# Evolution of ultraviolet vision in the largest avian radiation - the passerines

**DOI:** 10.1186/1471-2148-11-313

**Published:** 2011-10-24

**Authors:** Anders Ödeen, Olle Håstad, Per Alström

**Affiliations:** 1 Department of Animal Ecology, Uppsala University, Norbyvägen 18D, S-752 36 Uppsala, Sweden; 2 Department of Evolutionary Organismal Biology, Uppsala University, Norbyvägen 18A, S-752 36 Uppsala, Sweden; 3 Department of Anatomy, Physiology and Biochemistry, Swedish University of Agricultural Sciences, P.O. Box 7011, S-750 07, Uppsala, Sweden; 4 Swedish Species Information Centre, Swedish University of Agricultural Sciences, Box 7007, S-750 07 Uppsala, Sweden

## Abstract

**Background:**

Interspecific variation in avian colour vision falls into two discrete classes: violet sensitive (VS) and ultraviolet sensitive (UVS). They are characterised by the spectral sensitivity of the most shortwave sensitive of the four single cones, the SWS1, which is seemingly under direct control of as little as one amino acid substitution in the cone opsin protein. Changes in spectral sensitivity of the SWS1 are ecologically important, as they affect the abilities of birds to accurately assess potential mates, find food and minimise visibility of social signals to predators. Still, available data have indicated that shifts between classes are rare, with only four to five independent acquisitions of UV sensitivity in avian evolution.

**Results:**

We have classified a large sample of passeriform species as VS or UVS from genomic DNA and mapped the evolution of this character on a passerine phylogeny inferred from published molecular sequence data. Sequencing a small gene fragment has allowed us to trace the trait changing from one stable state to another through the radiation of the passeriform birds. Their ancestor is hypothesised to be UVS. In the subsequent radiation, colour vision changed between UVS and VS at least eight times.

**Conclusions:**

The phylogenetic distribution of SWS1 cone opsin types in Passeriformes reveals a much higher degree of complexity in avian colour vision evolution than what was previously indicated from the limited data available. Clades with variation in the colour vision system are nested among clades with a seemingly stable VS or UVS state, providing a rare opportunity to understand how an ecologically important trait under simple genetic control may co-evolve with, and be stabilised by, associated traits in a character complex.

## Background

**Figure 1:**
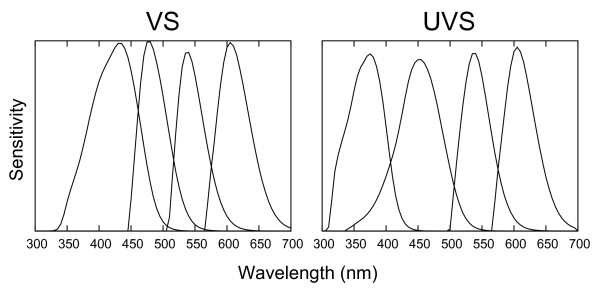
**Examples of spectral sensitivities for VS and UVS birds**. Normalised single cone spectral sensitivities (from left to right: SWS1, SWS2, MWS and LWS) of a VS bird (Indian Peafowl *Pavo cristatus*) [68] and a UVS bird (Eurasian Blue Tit *Cyanistes caeruleus*) [36] including the effects of ocular medium absorption. Human visible range is approximately 400-700 nm, with wavelengths shorter than 400 nm being ultraviolet.

Colour perception is one of the disciplines where birds excel. In general, interspecific variation in avian colour vision falls into two discrete classes, which are characterised by the spectral sensitivity of the most shortwave sensitive of the four single cones, the SWS1 [1] (the other, more long-wave sensitive being SWS2, MWS and LWS). The wavelength of maximum sensitivity (λ_max_) of the SWS1 ranges either from 355-380 nm in the 'ultraviolet sensitive' class (UVS) or from 402-426 nm in the 'violet sensitive' (VS) (reviewed by [2] and [3]). The UVS class is optimised for ultraviolet sensitivity, but the VS class also has some degree of sensitivity in the ultraviolet spectrum (Figure 1). Changes in spectral sensitivity of the SWS1 are ecologically important, as spectral tuning affects the abilities of birds to accurately assess the quality of potential mates [4-14], spot elusive prey or detect other food items [15-20] and minimise visibility of social signals in plumage coloration to predators [21].

Non-conservative substitutions in the SWS1 cone opsin protein that are located in the retinal binding pocket close enough to interact with the retinal chromophore may shift λ_max _from UVS to VS or vice versa [22,23]. Replacement of cysteine by serine in the 90^th ^amino acid (aa) position, the substitution Cys90Ser, or the reverse substitution, alone accounts for the whole shift (all aa residues named in this article are numbered according to bovine rhodopsin ([24]). Despite the simple nature of the mechanism, very few shifts have been described so far (e.g. review in [2]), suggesting that the SWS1 cone spectral sensitivity is under strong stabilising selection. The VS class is both ancestral and the most common to birds; its members are distributed throughout the avian phylogeny [25,26]. Four or five independent shifts to UVS are currently known: in shorebirds, Passerida passerines, parrots, the rhea and presumably trogons [[26] and references therein, [27-30]].

The most promising group of birds for further investigation into the spectral tuning of SWS1 cone opsins is clearly the passerines (Passeriformes). This is the most species rich of all avian orders and the only one except the shorebirds (Charadriiformes) from which both classes of spectral sensitivity are known. Every investigated member of the Passerida clade of passerines belongs to the UVS class [26,27,31-38] and it has appeared that all other passerines can be placed in the VS category (see [26,27,37,39] and Browne, *et al*. 2006 (GenBank only)). Recently, however, Ödeen *et al. *[40] argued that the evolution of ultraviolet sensitive vision in Passeriformes is more complex than a single VS to UVS opsin shift in an ancestor of Passerida, presenting molecular evidence for additional UV shifts from VS colour vision outside Passerida, in fairywrens, genus *Malurus*.

Since spectral tuning of the cone is under the genetic control of a few amino acid residues in the opsin protein, it is possible to quickly classify almost any bird as VS or UVS from a sample of genomic DNA [3,26]. The accuracy of this short-fragment genomic DNA approach in distinguishing UVS from VS species has been validated against all published MSP data [3]. We have employed this method to search for gross differences in spectral tuning in a larger sample of passerine species than has been investigated before. Our aims with this survey were to assess how stable SWS1 cone sensitivity has been in the course of passerine evolution and to trace the evolutionary sequence of shifts in spectral tuning. As the basis for the study, we have inferred a molecular phylogeny with sequence data from GenBank.

## Results

### Opsin sequencing

We amplified the target fragment of the SWS1 opsin gene in 56 passerine species from 30 families and one falconiform species, Northern Crested Caracara *Caracara cheriway *(Additional file 1). Cycle sequencing produced 50-107 bp long overlapping strands of identifiable nucleotides. Sequences have been deposited in the European Nucleotide Archive (ENA) (accession numbers in Additional file 1). Amino acid translations spanning the spectral tuning sites 86, 90 and 93 [22] are presented in Additional file 1. For unknown reasons, we failed to amplify the SWS1 opsin in two species: Blue Jewel-babbler *Ptilorrhoa caerulescens *(Psophodidae) and White-bellied Erpornis *Erpornis zantholeuca *(Vireonidae).

Position 90 held either serine or cysteine residues, signifying VS and UVS opsins, respectively. Neither phenylalanine nor any other aa residues suggesting a potential major shift in spectral tuning through a non-conservative substitution was present at site 86 (cf. [28]). However, the Rifleman *Acanthisitta chloris *holds leucine in spectral tuning site 93 (Leu93). This state is previously undescribed in birds and probably the result of a non-conservative substitution (see below).

### Phylogenetic reconstruction

**Figure 2:**
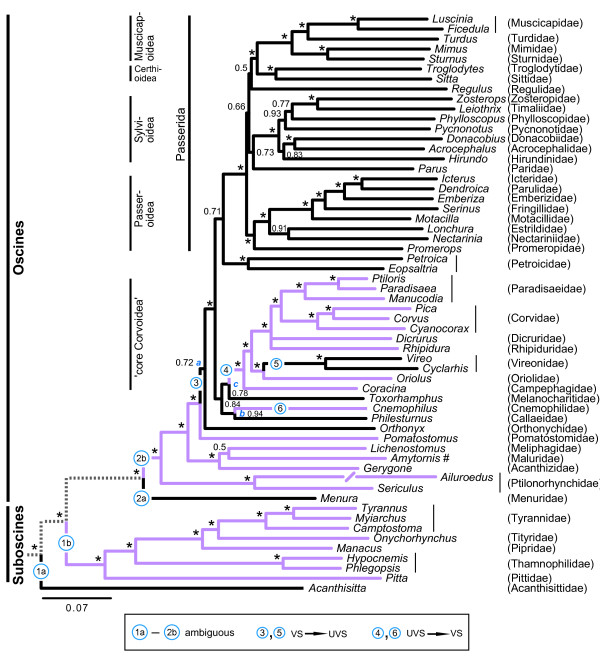
**Phylogenetic recontruction of SWS1 opsin evolution**. Majority rule (50%) consensus tree of passerines based on concatenated mitochondrial cytochrome *b *and ND2, nuclear myoglobin intron 2, ODC introns 6 to 7, TGFβ2 intron 5, and protein-coding nuclear c-myc exon 3, RAG-1 and RAG-2 sequences (> 9 kbp), inferred by Bayesian inference, analysed in eight partitions, with two parrots and two falconiforms as outgroup. Posterior probabilities given at nodes, * indicating ≥0.95. Long *Ailuroedus *branch truncated. VS/UVS optimisation represented by violet for VS, black for UVS and dotted for ambiguous. Transitions from one state to another are indicated by numbers; 1a and 1b, and 2a and 2b, respectively, represent uncertainties due to ambiguous ancestral state. *a*, *b *and *c *refer to insignificantly supported nodes discussed in the text. # Sister clade, genus *Malurus *(not included), contains both VS and UVS species [40].

The inferred tree (Figure 2) is mostly well resolved and, except for some short internal branches, well supported. It agrees well with previously published passerine trees based on fewer loci, such as a large-scale study of passerine relationships based on RAG-1 and -2 [41], as well as with more densely sampled studies of the Passerida based on ODC, myo and β-fibrinogen intron 5 [42] and of the 'core Corvoidea' based on ODC, myo, RAG-1 and -2, and ND2 [43].

The character optimisation infers a minimum of six changes, two from VS to UVS, two from UVS to VS and two with uncertain direction due to three nodes with ambiguous ancestral states near the root (Figure 2). The number of inferred transitions are reduced to five, if clades *b *and *c *in Figure 2, which are reconstructed with low support, are collapsed, and *Cnemophilus *is placed as sister to the 'core Corvoidea' clade (Additional file 2). On the contrary, if all 'basal' nodes with posterior probability < 0.95 are collapsed, eight transitions are inferred (Additional file 3). The ancestor to all passerines is reconstructed as UVS when only parrots (UVS) form the outgroup, whereas it is ambiguous when falconiforms (VS) are also included in the outgroup (as shown in Figure 2).

## Discussion

The evolution of colour vision in birds has apparently involved many more than the four to five independent shifts between SWS1 cone opsin types that previously available data have indicated. In Passeriformes, the picture is indeed more complex than a single VS to UVS opsin shift in an ancestor of Passerida; our phylogenetic trait mapping reveals more variation among the passerines than what is known from other birds combined. The class of colour vision of the most recent common ancestor to the passerines is ambiguous as a result of different states in parrots (UVS) and falconiforms (VS). As parrots are suggested to be the sister group to the passerines [44], the passerine ancestor is hypothesised to be UVS (as inferred when falconiforms are excluded from the optimisation analyses). In the subsequent passerine radiation, colour vision has changed between UVS and VS at least eight times, including two shifts in the family Maluridae (signified by # in Figure 2) [40].

It is interesting to note that two major clades, the Passerida/Petroicidae and the suboscines, are homogeneous with respect to colour vision (albeit different classes), whereas there is considerably more variation in the rest of the tree, particularly within Maluridae. It is not immediately apparent what the selective advantage of either VS or UVS might be, as both classes are present in taxa represented on all continents and in similar habitats. For example, the ancestor to the mostly forest-dwelling New World Vireonidae has changed from VS to UVS, whereas the mainly forest-inhabiting New World suboscines are VS. Moreover, the UVS Passerida/Petroicidae clade is mainly distributed in Afro-Eurasia and the New World, with few representatives in Australasia; the VS suboscines occur predominantly in the New World, with relatively few species in Africa, Madagascar, Asia and Australasia; the mostly VS, but also UVS, 'basalmost' oscines (*Menura*-*Orthonyx*) plus *Philesturnus*, *Cnemophilus *and *Toxorhampus *are chiefly Australasian; and the 'core Corvoidea' have representatives on all continents. The ancestral area of the passerines has been inferred to be eastern Gondwana, *i.e*. proto-Australasia [45,46], whereas the *Philesturnus*/*Cnemophilus*/*Toxorhampus*/'core Corvoidea' clade has been inferred to have originated in the proto-Papuan archipelago [43].

Unlike Maluridae (represented by *Amytornis *in Figure 2), none of the multi-sampled families from the present study are polytypic in gross tuning of the SWS1 opsin, but either contain VS or UVS taxa. In common with the Australian-Papuan Maluridae, the African-Eurasian genus *Motacilla *(wagtails) is rich in species and subspecies with strong differentiation in male plumage coloration. Yet, all the members of *Motacilla *sampled have base-pair identical SWS1 opsin gene fragments.

Unknown differences in other traits than SWS1 could explain why UVS-VS shifts are so unequally distributed between Passerida/Petroicidae and other passerines. Spectral tuning of the SWS1 cone opsin seems to be under strong stabilising selection in birds [30], likely partly due to gross spectral tuning shifts carrying costs, such as increased retinal photooxidation and ocular light scattering (reviewed in [47]). In addition, acute short wave colour vision is dependent on a complex of co-adapted physiological traits. Ultraviolet absorption by the ocular media is usually stronger in VS than in UVS species (reviewed in [48,49]), and the SWS2 cone class (sensitive to 'blue', λ_max _451-480 nm) is shifted toward shorter wavelengths in UVS than in VS birds (see review in [2]). A shortwave shift in λ_max _of a VS SWS1 pigment will produce a virtually negligible increase in UV sensitivity, unless it is accompanied by an increased UV transmission in the ocular media [48]. A shortwave shifted SWS1 will cause an uneven distribution of sensitivities across the spectrum, possibly deteriorating colour discriminability, if not followed by a shift of the SWS2 pigment towards the range vacated by SWS1. A plausible evolutionary scenario is that increased UV transmittance in an ancestor of passerines relaxed stabilising selection on SWS1 spectral tuning, allowing a VS to UVS shift of that opsin to reach fixation (see [48]) (the only close relative to passerines studied in this respect is one with a UV transparent ocular media: Budgerigar *Melopsittacus undulatus *[49]). Later, a shortwave shift in SWS2 spectral tuning in an ancestor of Passerida/Petroicidae may have improved spectral discriminability of UVS retinae and thereby reinstated stabilising selection on SWS1 spectral tuning. In agreement with this scenario, Passerida species where this cone has been examined by MSP mostly show shortwave shifted SWS2 λ_max _compared to VS species from other avian orders (see review [2]), but unfortunately the character state is not known from any VS or non-Passerida passerine. The realised spectral sensitivities of the two shortwave sensitive cones in non-Passerida passerines may be different from that of characteristic VS and UVS types (cf. review in [2]). Due to a general paucity of data on SWS2 λ_max _and ocular transmission, one can merely speculate at this point.

Shifts from UVS to VS in passerines seem to be controlled exclusively by the Cys90Ser substitution. Carvalho *et al. *[28] has reported a significant short wavelength tuning effect of substitution Ser86Phe, but we did not find phenylalanine in position 86 (Phe86) in any of the species we sequenced. The effects of all tuning site substitutions found in this study are known [22,23], with one exception: a leucine residue in the spectral tuning site 93 of the Rifleman *Acanthisitta chloris*. The most likely evolutionary event given the phylogeny in Figure 2 is the substitution Thr93Leu. This is a non-conservative substitution, changing from polar and mildly hydrophilic threonine to nonpolar, hydrophobic leucine. Site 93 is located in the retinal binding pocket, on the inner side of the opsin's alpha-helices close enough to interact with the retinal chromophore. Thr93Leu is therefore potentially important to spectral tuning (see [50]), but its effect in the avian SWS1 opsin is presently unknown.

The reconstruction of the evolution of colour vision in passerines is conditional on the true phylogeny having been inferred. The tree inferred here is based on a larger number of loci than any previous study of passerine relationships. As it is overall well supported by the data, and in good agreement with previous analyses of different datasets (e.g. [41-43], it is a well founded hypothesis of relationships. Although the tree has some nodes with low posterior probability, only two of these affect the interpretation of the evolution of colour vision (indicated by *a *and *b *in Figure 2). With respect to the poorly supported node *a*, switching position between *Pomatostomus *and *Orthonyx*, as is possible if this node is collapsed, would require one more step in the VS/UVS optimisation and result in eight further internal nodes having ambiguous states. Further collapsing of poorly supported 'basal' nodes leads to more steps in the UV optimisation. Accordingly, the opsin data lend further support to the present topology, and the favoured topology results in a more parsimonious optimisation than the alternative topologies. However, if the insignificantly supported nodes *b *and *c *are collapsed, and *Cnemophilus *is placed as sister to the 'core Corvoidea', one fewer change is required to explain the evolution of colour vision. More sequence data might resolve this issue in the future.

## Conclusions

Spectral tuning of the SWS1 cone opsin in birds is a trait of great ecological importance. It appears to be under strong stabilising selection and varies categorically between different clades in the avian phylogenetic tree. In the radiation of the very species rich order Passeriformes, sequencing of a small gene fragment allows us to map and trace the change from one stable state to the other. Clades with variation in the colour vision system are nested among clades with a seemingly stable VS or UVS state, providing a rare opportunity to understand how an ecologically important trait under simple genetic control may co-evolve with, and be stabilised by, associated traits in a character complex.

## Methods

### Opsin sequencing

We isolated genomic DNA from tissues and blood samples, which we took from live birds in the field or borrowed from museums and colleagues. As far as feasible we included representatives from clades of expected importance to tracing the radiation of passerines. As demonstrated in the fairywren case [40], closely related groups of passerine taxa might be polytypic with respect to spectral tuning of the SWS1 opsin. Acknowledging this possibility, we sampled multiple taxa in selected genera. We used a GeneMole^® ^automated nucleic acid extraction instrument (Mole Genetics) and the DNeasy Blood and Tissue Kit (QIAGEN) for the DNA isolation. Standard procedures were applied. With the same protocol and primers as are described in [3] and [26] we then amplified a fragment of the SWS1 opsin gene, containing the aa residues at positions 81-94, all located in the 2^nd ^α-helical transmembrane region.

We translated the DNA sequences into amino acids to identify the spectral tuning sites 86, 90, and 93 of SWS1 [22,23]. Then we calculated λ_max _from the tuning sites following *in vitro *changes in λ_max _reported by Wilkie *et al. *[22]. We assumed the effects of the key tuning sites to be additive. This assumption should provide reasonable approximations of λ_max _[51], although it disregards potential interactions between the tuning sites (see [52]).

### Phylogenetic reconstruction

A phylogeny was inferred from sequences in GenBank (Additional file 4). We made a search in GenBank for all falconiform, psittaciform and passeriform species for which we and others had sequenced the SWS1 opsin gene. We then selected eight loci for which sequences were available for a large proportion of these species or members of the same genus: the mitochondrial cytochrome *b *(cyt*b*) and NADH dehydrogenase II (ND2) genes, the nuclear myoglobin gene, intron 2 (myo), ornithine decarboxylase gene, introns 6 to 7 (ODC), transforming growth factor beta-2 gene, intron 5 (TGFβ2), and the protein-coding nuclear c-myc exon 3 and recombination-activating protein 1 and 2 genes (RAG-1, RAG-2). Sequences were aligned using the MUSCLE web server http://www.drive5.com/muscle; some manual adjustment was necessary for the non-coding sequences. The alignment is in Additional file 5.

The phylogeny was estimated by Bayesian inference using MrBayes 3.1.2 [53,54], with the sequences concatenated and partitioned by locus, using rate multipliers to allow different rates for the different partitions [55,56]. Ambiguous base pairs and indels were treated as missing data. As outgroups, we chose two falconiforms and two parrots, as these have been shown to be closely related to passerines [44,57]. Appropriate substitution models were determined based on the Bayesian Information Criterion [58] calculated by jModelTest version 0.1.1 [59]. For cyt*b*, ND2 and RAG-1, the best-fit model was the general time-reversible (GTR) model [60-62], assuming rate variation across sites according to a discrete gamma distribution with four rate categories (Γ; [63]) and an estimated proportion of invariant sites (I; [64]) (GTR+Γ+I). For the other loci, the Hasegawa-Kishino-Yano (HKY) model [65] plus Γ was selected (HKY+Γ), with the addition of I for myoglobin and c-myc (HKY+Γ+I). Posterior probabilities (PPs) were calculated in MrBayes using default priors. Four Metropolis-coupled MCMC chains with incremental heating temperature 0.1 were run for 7 × 10^6 ^generations and sampled every 1000 generations. Chain likelihood and other parameter values and effective sample sizes (> 200) were inspected in Tracer 1.5.0 [66]. The first 25% of the generations were discarded as 'burn-in', well after stationarity of most chain likelihood values had been established, and the posterior probability was estimated for the remaining generations. The analysis was run eight times, and the topologies and posterior probabilities were compared by eye and by the mean estimates and the corresponding standard errors.

Optimisation of the VS/UVS character was performed by parsimony in MacClade 4.08 [67], using default settings (polytomies treated as soft).

## List of abbreviations

λ_max_: wavelength of maximum absorbance; MSP: microspectrophotometry; SWS1: short-wavelength sensitive pigment, type one; VS: violet sensitive; UVS: ultraviolet sensitive.

## Authors' contributions

AÖ compiled the tissue material and carried out the genetic studies. AÖ and OH conceived of the study, interpreted the opsin results and compiled most of the molecular marker data. PA compiled some of the molecular marker data, performed sequence alignment and reconstructed the phylogeny. All authors participated in the trait state mapping, drafted the manuscript, and read and approved it.

## Supplementary Material

Additional file 1 **SWS1 opsin aa sequences and accession numbers**. SWS1 opsin amino acid (aa) sequences from passerine plus falconiform and psittaciform species analysed in the present study and previously. The spectral tuning aa sites 86, 90 and 93 (see text) are marked in bold, as are the ENA accession number of sequences new to this study. Type of SWS1 opsin is estimated from the tuning sites as either VS (violet sensitive) or UVS (UV-sensitive). The taxonomy follows the IOC World Bird List [69]. Numbers after species names signify the number of individuals sequenced. Information on geographic location for the new samples is available at ENA http://www.ebi.ac.uk/ena/data/view/< ACCESSION NUMBERS HE601811-HE601869 >. Click here for file

Additional file 2 **Alternative recontruction of SWS1 opsin evolution, 1**. Same tree as in Figure 2, but with clades *b *and *c*, which are reconstructed with low support, collapsed, and *Cnemophilus *placed as sister to the 'core Corvoidea' clade. VS/UVS optimisation represented by yellow for VS, blue for UVS, and barred for ambiguous. Click here for file

Additional file 3 **Alternative recontruction of SWS1 opsin evolution, 2**. Same tree as in Figure 2, but with all 'basal' nodes with posterior probability < 0.95 collapsed. VS/UVS optimisation represented by yellow for VS, blue for UVS, and barred for ambiguous. Click here for file

Additional file 4 **Sequences used for phylogenetic reconstruction**. GenBank numbers for sequences used for phylogenetic reconstruction. Click here for file

Additional file 5 **Sequence alignment**. Alignment of sequences used for phylogenetic reconstruction. Details in Additional file 4. Click here for file
