# Supplementary material for: Evolution of ultraviolet vision in the largest avian radiation - the passerines
Source: BMC Evol Biol. 2011 Oct 24;11:313. doi: 10.1186/1471-2148-11-313 (PMC3225180; doi:10.1186/1471-2148-11-313)
Supplement: Additional file 1 — SWS1 opsin aa sequences and accession numbers. SWS1 opsin amino acid (aa) sequences from passerine plus falconiform and psittaciform species analysed in the present study and previously. The spectral tuning aa sites 86, 90 and 93 (see text) are marked in bold, as are the ENA accession number of sequences new to this study. Type of SWS1 opsin is estimated from the tuning sites as either VS (violet sensitive) or UVS (UV-sensitive). The taxonomy follows the IOC World Bird List [69]. Numbers after species names signify the number of individuals sequenced. Information on geographic location for the new samples is available at ENA http://www.ebi.ac.uk/ena/data/view/< ACCESSION NUMBERS HE601811-HE601869 >. [file 1471-2148-11-313-S1.DOCX]

| Order | Family | Species | Common name | aa seq. 84-94 | Colour vision | Origin/Voucher^1^ | Tissue^1^ | Acc. no. | Reference |
| --- | --- | --- | --- | --- | --- | --- | --- | --- | --- |
| Falconiformes | Falcondiae | *Caracara cheriway* | Northern Crested Caracara | FI**S**YTF**S**VF**T**V | VS | FMNH 393524 |  | **HE601811** |  |
| Falconiformes | Falcondiae | *Falco peregrinus* | Peregrine Falcon | FI**S**CIF**S**VF**T**V | VS | NRM 956709 | A1995.6079 | AY227157 | [26] |
| Psittaciformes | Strigopidae | *Nestor notabilis* | Kea | FL**A**CIF**C**IF**T**V | UVS |  |  | HM150807 | [29] |
| Psittaciformes | Cacatuidae | *Calyptorhynchus latirostris* | Short-billed Black Cockatoo | FL**A**CIF**C**IF**T**V | UVS |  |  | HM150800 | [29] |
| Psittaciformes | Cacatuidae | *Eolophus roseicapilla* | Galah | FL**A**CIF**C**IF**T**V | UVS |  |  | HM150801 | [29] |
| Psittaciformes | Cacatuidae | *Cacatua galerita* | Sulphur-crested Cockatoo | FL**A**CIF**C**IF**T**V | UVS |  |  | HM150802 | [29] |
| Psittaciformes | Cacatuidae | *Cacatua moluccensis* (2) | Salmon-crested Cockatoo | FL**A**CIF**C**IF**T**V | UVS |  |  | HM150797,  HM150798 | [29] |
| Psittaciformes | Psittacidae | *Barnardius zonarius semitorquatus* | Australian Ringneck | FL**A**CIF**C**IF**T**V | UVS |  |  | HM150799 | [29] |
| Psittaciformes | Psittacidae | *Platycercus elegans* | Crimson Rosella | FL**A**CIF**C**IF**T**V | UVS |  |  | HM150794 | [29] |
| Psittaciformes | Psittacidae | *Melopsittacus undulatus* | Budgerigar | FL**A**CII**C**IF**T**V | UVS |  |  | Y11787 | [50] |
| Psittaciformes | Psittacidae | *Psittacus erithacus* | Grey Parrot | FL**A**CIF**C**IF**T**V | UVS | IBG, UU |  | AY227186 | [26] |
| Psittaciformes | Psittacidae | *Anodorhynchus hyacinthinus* | Hyacinth Macaw | FP**A**CIF**C**IF**T**V | UVS |  |  | HM150806 | [29] |
| Psittaciformes | Psittacidae | *Ara ararauna* | Blue-and-yellow Macaw | FL**A**CIF**C**IF**T**V | UVS |  |  | HM150803 | [29] |
| Psittaciformes | Psittacidae | *Ara macao* | Scarlet Macaw | FL**A**CIF**C**IF**T**V | UVS |  |  | HM150792 | [29] |
| Psittaciformes | Psittacidae | *Ara chloropterus* | Red-and-green Macaw | FL**A**CIF**C**IF**T**V | UVS |  |  | HM150805 | [29] |
| Psittaciformes | Psittacidae | *Amazona versicolor* | St. Lucia Amazon | FL**A**CIF**C**IF**T**V | UVS |  |  | HM150793 | [29] |
| Psittaciformes | Psittacidae | *Amazona guildingii* (2) | St. Vincent Amazon | FL**A**CIF**C**IF**T**V | UVS |  |  | HM150795,  HM150796 | [29] |
| Passeriformes | Acanthisittidae | *Acanthisitta chloris* | Rifleman | LL**C**CIL**C**VF**L**V | UVS | A.J. Baker | 6 | **HE601812** |  |
| Passeriformes | Pittidae | *Hydrornis elliotii* | Bar-bellied Pitta | FL**C**CIF**S**VF**T**V | VS | NRM 20047000 |  | **HE601813** |  |
| Passeriformes | Thamnophilidae | *Hypocnemis peruviana* | Peruvian Warbling Antbird | FL**C**CIF**S**VF**T**V | VS | CICRA, N. Seddon | 29.09.07 | GQ924590 | [39] |
| Passeriformes | Thamnophilidae | *Myrmeciza hemimelaena* | Southern Chestnut-tailed Antbird | FL**C**CIF**S**VF**T**V | VS | CICRA, N. Seddon | 22.11.07 | GQ924591 | [39] |
| Passeriformes | Thamnophilidae | *Phlegopsis nigromaculata* | Black-spotted Bare-eye | FL**C**CIF**S**VF**T**V | VS | CICRA, N. Seddon | 10.11.04 | GQ924592 | [39] |
| Passeriformes | Tyrannidae | *Camptostoma obsoletum* | Southern Beardless Tyrannulet | FM**C**CIF**S**VF**T**V | VS | NRM 937368 | CMS-018 | **HE601814** |  |
| Passeriformes | Tyrannidae | *Xolmis irupero* | White Monjita | FM**C**CIF**S**VF**T**V | VS | NRM 937384 | ICM-033 | **HE601815** |  |
| Passeriformes | Tyrannidae | *Tyrannus savana* | Fork-tailed Flycatcher | FM**C**CIF**S**VF**T**V | VS | NRM 937164 | AHN-039 | **HE601816** |  |
| Passeriformes | Tyrannidae | *Myiarchus tyrannulus* | Brown-crested Flycatcher | FM**C**CIF**S**VF**T**V | VS | NRM 937173 | AHN-048 | AY227183 | [26] |
| Passeriformes | Pipridae | *Manacus manacus* | White-bearded Manakin | FI**S**CIF**S**VF**T**V | VS | L. Shorey |  | AY227182 | [26] |
| Passeriformes | Tityridae | *Onychorhynchus coronatus* | Amazonian Royal Flycatcher | FF**C**CIF**S**VF**V**V | VS | UWBM 56019 | DAB 1082 | **HE601817** |  |
| Passeriformes | Menuridae | *Menura alberti* | Albert's Lyrebird | FF**C**CIF**C**VF**T**V | UVS | ANWC B47113 |  | **HE601818** |  |
| Passeriformes | Menuridae | *Menura novaehollandiae* | Superb Lyrebird | FF**C**CIF**C**VF**T**V | UVS | O. 70813 | EBU 37068 | **HE601819** |  |
| Passeriformes | Ptilonorhynchidae | *Ailuroedus crassirostris* | Green Catbird | FL**C**CIF**S**VF**T**V | VS | O. 71114 | EBU 39507 | HE588090 | [40] |
| Passeriformes | Ptilonorhynchidae | *Sericulus chrysocephalus* | Regent Bowerbird | FL**C**CIF**S**VF**T**V | VS | O. 70804 | EBU 38490 | HE588091 | [40] |
| Passeriformes | Ptilonorhynchidae | *Chlamydera nuchalis* | Great Bowerbird | FL**C**CIF**S**VF**T**V | VS | O. 65796 | EBU 10352 | HE588092 | [40] |
| Passeriformes | Maluridae | *Chenorhamphus grayi* | Broad-billed Fairywren | FL**C**CIF**C**IF**T**V | UVS | KUNHM 97993 | 7082 | HE588093 | [40] |
| Passeriformes | Maluridae | *Malurus cyanocephalus* | Emperor Fairywren | FL**C**CIF**S**VF**T**V | VS | KUNHM 87988 | 7564 | HE588094 | [40] |
| Passeriformes | Maluridae | *Malurus amabilis* | Lovely Fairywren | FL**C**CIF**C**IF**T**V | UVS | S. Pruett-Jones | LW01A | HE588095 | [40] |
| Passeriformes | Maluridae | *Malurus lamberti lamberti* | Variegated Fairywren | FL**C**CIF**C**IF**T**V | UVS | ANWC B46335 | 46335 | HE588097 | [40] |
| Passeriformes | Maluridae | *Malurus lamberti assimilis* | Purple-backed Fairywren | FL**C**CIF**C**IF**T**V | UVS | S. Pruett-Jones | VW104 | HE588096 | [40] |
| Passeriformes | Maluridae | *Malurus pulcherrimus* | Blue-breasted Fairywren | XL**C**CIF**C**IF**T**V | UVS | UWBM 60862 | PLG300 | HE588098 | [40] |
| Passeriformes | Maluridae | *Malurus elegans* | Red-winged Fairywren | FL**C**CIF**C**IF**T**V | UVS | WAM A27629 | A27629 | HE588099 | [40] |
| Passeriformes | Maluridae | *Malurus cyaneus* | Superb Fairywren | FL**C**CIF**C**IF**T**V | UVS | ANWC B34618 |  | HE588100 | [40] |
| Passeriformes | Maluridae | *Malurus splendens musgravi* | Turquoise Fairywren | FL**C**CIF**C**IF**T**V | UVS | ANSP 189635 | 10547 | HE588101 | [40] |
| Passeriformes | Maluridae | *Malurus splendens emmottorum* | Splendid Fairywren | FL**C**CIF**C**IF**T**V | UVS | UWBM 57545 | SAR7063 | HE588102 | [40] |
| Passeriformes | Maluridae | *Malurus splendens melanotus* (5) | Black-backed fairywren | FL**C**CIF**C**IF**T**V | UVS | S. Pruett-Jones | SW256, -268, -683 -727, -736 | HE588103-HE588107 | [40] |
| Passeriformes | Maluridae | *Malurus coronatus* (2) | Purple-crowned Fairywren | FL**C**CIF**S**VF**T**V | VS | S. Pruett-Jones | 2003–01, 2003–02 | HE588108,  HE588109 | [40] |
| Passeriformes | Maluridae | *Malurus alboscapulatus naimii* | White-shouldered Fairywren | FL**C**CIF**S**VF**T**V | VS | ANWC B26769 | MVM E121 | HE588110 | [40] |
| Passeriformes | Maluridae | *Malurus melanocephalus* (3) | Red-backed Fairywren | FL**C**CIF**S**VF**T**V | VS | ANWC B29594, B50894, J. Karubian | -, - ,RBW01 | HE588111-HE588113 | [40] |
| Passeriformes | Maluridae | *Malurus leucopterus edouardi* (3) | White-winged Fairywren | FL**C**CIF**S**VF**T**V | VS | S. Pruett-Jones | BW008, -13, -15 | HE588114-HE588116 | [40] |
| Passeriformes | Maluridae | *Malurus leucopterus leuconotus* (2) | Blue-and-white Fairywren | FL**C**CIF**S**VF**T**V | VS | S. Pruett-Jones | WW10, -14 | HE588117,  HE588118 | [40] |
| Passeriformes | Maluridae | *Malurus leucopterus leucopterus* (2) | Black-and-white Fairywren | FL**C**CIF**S**VF**T**V | VS | WAM A26804, -6 | A26804, -6 | HE588119,  HE588120 | [40] |
| Passeriformes | Maluridae | *Clytomyias insignis* | Orange-crowned Fairywren | FL**C**CIF**S**VF**T**V | VS |  | Kan4619 | HE588121 | [40] |
| Passeriformes | Maluridae | *Stipiturus mallee* | Mallee Emuwren | FL**C**CIF**S**VF**T**V | VS | S. Pruett-Jones | MEW1 | HE588122 | [40] |
| Passeriformes | Maluridae | *Amytornis barbatus* | Grey Grasswren | FL**C**CIF**S**VF**T**V | VS | ANWC B41788 |  | HE588123 | [40] |
| Passeriformes | Maluridae | *Amytornis striatus* | Striated Grasswren | FL**C**CIF**S**VS**T**V | VS | S. Pruett-Jones | SGW1 | HE588124 | [40] |
| Passeriformes | Meliphagidae | *Lichenostomus flavescens* | Yellow-tinted Honeyeater | FM**C**CIF**S**VF**T**V | VS | O. 66338 | EBU 10401 | GQ305955 | [27] |
| Passeriformes | Meliphagidae | *Philemon argenticeps* | Silver-crowned Friarbird | FM**C**CIF**S**VF**T**V | VS | O. 65825 | EBU 10283 | GQ305956 | [27] |
| Passeriformes | Meliphagidae | *Phylidonyris novaehollandiae* | New Holland Honeyeater | FM**C**CIF**S**VF**T**V | VS | O. 70854 | EBU 38188 | GQ305957 | [27] |
| Passeriformes | Meliphagidae | *Conopophila rufogularis* | Rufous-throated Honeyeater | FM**C**CIF**S**VF**T**V | VS | O. 65927 | EBU 10376 | GQ305958 | [27] |
| Passeriformes | Meliphagidae | *Acanthorhynchus tenuirostris* | Eastern Spinebill | FM**C**CIF**S**VF**T**V | VS | O. 70913 | EBU 38146 | GQ305959 | [27] |
| Passeriformes | Acanthizidae | *Gerygone igata* (2) | Grey Gerygone | FM**C**CIF**S**VF**T**V | VS | GW1, -2 |  | HM159130, HM159131 | Hauber & Chong (GenBank 2010) |
| Passeriformes | Pomatostomidae | *Pomatostomus temporalis* | Grey-crowned Babbler | FL**C**CIF**S**VF**T**V | VS | LSUMZ-B23626 |  | **HE601820** |  |
| Passeriformes | Pomatostomidae | *Pomatostomus ruficeps* | Chestnut-crowned Babbler | FL**C**CIF**S**VF**T**V | VS | LSUMZ-B23409 |  | **HE601821** |  |
| Passeriformes | Orthonychidae | *Orthonyx temminckii* | Australian Logrunner | FF**C**CIF**C**VF**T**V | UVS | UWBM 76694 | WBJ 3019 | **HE601822** |  |
| Passeriformes | Cnemophilidae | *Cnemophilus loriae* | Loria's Satinbird | LM**C**CIF**S**VF**T**V | VS | NRM 569572 |  | **HE601823** |  |
| Passeriformes | Melanocharitidae | *Toxorhamphus poliopterus* | Slaty-headed Longbill | LM**C**CVF**C**IF**T**V | UVS | NRM 543574 |  | **HE601824** |  |
| Passeriformes | Callaeidae | *Philesturnus carunculatus* | South Island Saddleback | LM**C**CVF**C**IF**T**V | UVS | PHCA |  | HM159129 | Hauber & Chong (GenBank 2010) |
| Passeriformes | Campephagidae | *Coracina novaehollandiae* | Black-faced Cuckooshrike | FL**C**CIF**S**VF**T**V | VS | ANWC B50378 |  | **HE601825** |  |
| Passeriformes | Vireonidae | *Cyclarhis gujanensis* | Rufous-browed Peppershrike | LM**C**CVF**C**IF**T**V | UVS | UWBM 77444 | VGR 283 | **HE601826** |  |
| Passeriformes | Vireonidae | *Vireo bellii* | Bell's Vireo | LM**C**CVF**C**IF**T**V | UVS | UWBM 81347 | VGR 877 | **HE601827** |  |
| Passeriformes | Oriolidae | *Oriolus oriolus* | Eurasian Golden Oriole | FL**C**CIF**S**VF**T**V | VS | NRM 20036563 |  | **HE601828** |  |
| Passeriformes | Dicruridae | *Dicrurus bracteatus* | Spangled Drongo | FL**C**CIF**S**VF**T**V | VS | ANWC B29889 |  | **HE601829** |  |
| Passeriformes | Rhipiduridae | *Rhipidura leucophrys* | Willie Wagtail | FL**C**CIF**S**VF**T**V | VS | ANWC B32827 |  | **HE601830** |  |
| Passeriformes | Rhipiduridae | *Rhipidura albiscapa* | Grey Fantail | FL**C**CIF**S**VF**T**V | VS | ANWC B50231 |  | **HE601831** |  |
| Passeriformes | Corvidae | *Cyanocorax chrysops* | Plush-crested Jay | FL**C**CIF**S**VF**T**V | VS | NRM 956690 | GFK-178 | **HE601832** |  |
| Passeriformes | Corvidae | *Garrulus glandarius* | Eurasian Jay | FL**C**CIF**S**VF**T**V | VS | P. Halvarsson, Dept. Population Biology, U.U. | 6188056 | **HE601833** |  |
| Passeriformes | Corvidae | *Pica pica* | Eurasian Magpie | FL**C**CIF**S**VF**T**V | VS | Uppsala kommun/Dept. Animal Ecology, UU | Hov17 | GQ305970 | [37] |
| Passeriformes | Corvidae | *Coloeus monedula* | Western Jackdaw | FL**C**CIF**S**VF**T**V | VS | Uppsala kommun |  | AY227177 | [26] |
| Passeriformes | Corvidae | *Corvus frugilegus* | Rook | FL**C**CIF**S**VF**T**V | VS |  |  | DQ451006 | Browne *et al.* (Genbank 2006) |
| Passeriformes | Corvidae | *Corvus cornix* | Hooded Crow | FM**C**CIF**S**VF**T**V | VS | Uppsala kommun |  | AY227176 | [26] |
| Passeriformes | Paradisaeidae | *Manucodia comrii* | Curl-crested Manucode | FL**C**CIF**S**VF**T**V | VS | O. 60598 | EBU 10071 | **HE601834** |  |
| Passeriformes | Paradisaeidae | *Ptiloris magnificus* | Magnificent Riflebird | FL**C**CIF**S**VF**T**V | VS | O. 64926 | EBU 11365 | **HE601835** |  |
| Passeriformes | Paradisaeidae | *Paradisaea rudolphi* | Blue Bird-of-paradise | FM**C**CIF**S**VF**X**V | VS | NRM 20046265 | Gelin-N28 | **HE601836** |  |
| Passeriformes | Petroicidae | *Eopsaltria australis* | Eastern Yellow Robin | LM**C**CIF**C**LF**T**V | UVS | O. 71121 | EBU 39591 | **HE601837** |  |
| Passeriformes | Petroicidae | *Microeca fascinans* | Jacky Winter | LM**C**CIF**C**LF**T**V | UVS | O. 65957 | EBU 10407 | **HE601838** |  |
| Passeriformes | Petroicidae | *Petroica rosea* | Rose Robin | LM**C**CIF**C**LF**T**V | UVS | O. 70044 | EBU 10234 | **HE601839** |  |
| Passeriformes | Petroicidae | *Petroica goodenovii* (2) | Red-capped Robin | LM**C**CIF**C**LF**T**V | UVS | D. Dowling | 274, 278 | **HE601840,**  **HE601841** |  |
| Passeriformes | Paridae | *Cyanistes caeruleus* | Eurasian Blue Tit | LM**C**CVF**C**IF**T**V | VS | N. Backström & S. Berlin | BT49 | FJ440638 | [3] |
| Passeriformes | Pycnonotidae | *Pycnonotus cafer* | Red-vented Bulbul | LM**M**CIF**C**IF**T**V | UVS | NRM 20036334 |  | **HE601842** |  |
| Passeriformes | Hirundinidae | *Hirundo rustica* | Barn Swallow | LM**M**CIF**C**IF**T**V | UVS | T. Sirotkin & A.Ö. | Fi15 | **HE601843** |  |
| Passeriformes | Phylloscopidae | *Phylloscopus trochilus* | Willow Warbler | LM**M**CIF**C**IF**T**V | UVS | M. Vila-Taboada, AÖ | Ups01 | AY227181 | [26] |
| Passeriformes | Acrocephalidae | *Acrocephalus stentoreus* | Clamorous Reed Warbler | LM**M**CIF**C**IF**T**V | UVS | C. Hemborg & A.Ö. | Eg10 | **HE601844** |  |
| Passeriformes | Acrocephalidae | *Acrocephalus schoenobaenus* | Sedge Warbler | LM**M**CIF**C**IF**T**V | UVS | M. Wilson, C. Hemborg, S. Ulfstrand & A.Ö. | Ug51 | **HE601845** |  |
| Passeriformes | Acrocephalidae | *Acrocephalus scirpaceus fuscus* | Eurasian Reed Warbler | LM**M**CIF**C**IF**T**V | UVS | M. Wilson, C. Hemborg, S. Ulfstrand & A.Ö. | Ug49 | **HE601846** |  |
| Passeriformes | Acrocephalidae | *Hippolais polyglotta* | Melodious Warbler | LM**M**CIF**C**IF**T**V | UVS | A. Lindström & A.Ö. | It20 | **HE601847** |  |
| Passeriformes | Donacobiidae | *Donacobius atricapilla* | Black-capped Donacobius | LM**M**CIF**C**IF**T**V | UVS | NRM 966966 | GFK-244 | **HE601848** |  |
| Passeriformes | Timaliidae | *Leiothrix lutea* | Red-billed Leiothrix | LM**M**CVF**C**IF**T**V | UVS | NRM 20026687 | VNM 2002-078 | FJ440645 | [3] |
| Passeriformes | Zosteropidae | *Zosterops japonicus* | Japanese White-eye | LM**M**CIF**C**IF**T**V | UVS | NRM 20026678 |  | GQ305960 | [27] |
| Passeriformes | Zosteropidae | *Zosterops senegalensis* | African Yellow White-eye | LM**M**CIF**C**IF**T**V | UVS | NRM 20066253 |  | GQ305961 | [27] |
| Passeriformes | Promeropidae | *Promerops gurneyi* | Gurney’s Sugarbird | LM**C**CVF**C**IF**T**V | UVS | UWBM 70395 | GAV 447 | GQ305965 | [27] |
| Passeriformes | Regulidae | *Regulus regulus* | Goldcrest | LM**C**CIF**C**IF**T**V | UVS | Dept. Animal Ecology, UU | 7.030218 | **HE601849** |  |
| Passeriformes | Troglodytidae | *Troglodytes aedon* | House Wren | LM**C**CIF**C**IF**T**V | UVS | N. Backström & S. Berlin | E3 | **HE601850** |  |
| Passeriformes | Sittidae | *Sitta europaea* (2) | Eurasian Nuthatch | LM**C**CIF**C**IF**T**V | UVS | J. Tomiuk | Kl207, -41 | **HE601851,**  **HE601852** |  |
| Passeriformes | Mimidae | *Mimus saturninus* | Chalk-browed Mockingbird | LM**C**CIF**C**IF**T**V | UVS | NRM 966912 | PER-190 | GQ305972 | [37] |
| Passeriformes | Sturnidae | *Acridotheres tristis* | Common Myna | LM**C**CIF**C**IF**T**V | UVS | NRM 20046711 |  | **HE601853** |  |
| Passeriformes | Sturnidae | *Sturnus vulgaris* | Common Starling | LM**C**CIF**C**IF**T**V | UVS | A. Lindström & A.Ö. | It16 | AY227180 | [26] |
| Passeriformes | Turdidae | *Turdus merula* | Common Blackbird | LM**C**CVF**C**IF**T**V | UVS | N. Backström & S. Berlin | TuMe2 | FJ440637 | [3] |
| Passeriformes | Turdidae | *Turdus iliacus* | Redwing | LM**C**CVF**C**IF**T**V | UVS | M. Vila-Taboada, A.Ö. | Ups02 | **HE601854** |  |
| Passeriformes | Muscicapidae | *Luscinia svecica* | Bluethroat | LM**C**CVF**C**IF**T**V | UVS | A. Lindström & A.Ö. | Fi21 | **HE601855** |  |
| Passeriformes | Muscicapidae | *Muscicapa aquatica* | Swamp Flycatcher | LM**C**CVF**C**IF**T**V | UVS | M. Wilson, C. Hemborg, S. Ulfstrand & A.Ö. | Ug40 | **HE601856** |  |
| Passeriformes | Muscicapidae | *Ficedula hypoleuca* | European Pied Flycatcher | LM**C**CVF**C**IF**T**V | UVS | A. Qvarnström | Kol36 | **HE601857** |  |
| Passeriformes | Muscicapidae | *Ficedula albicollis* | Collared Flycatcher | LM**C**CVF**C**IF**T**V | UVS | A. Qvarnström | Kol6 | **HE601858** |  |
| Passeriformes | Nectariniidae | *Chalcomitra senegalensis* | Scarlet-chested Sunbird | LM**C**CVF**C**IF**T**V | UVS | NRM 20056357 |  | GQ305963 | [27] |
| Passeriformes | Nectariniidae | *Cinnyris pulchellus* | Beautiful Sunbird | LM**C**CVF**C**IF**T**V | UVS | NRM 20076163 |  | GQ305964 | [27] |
| Passeriformes | Nectariniidae | *Aethopyga siparaja* | Crimson Sunbird | LM**C**CVF**C**IF**T**V | UVS | NRM 20026613 |  | GQ305962 | [27] |
| Passeriformes | Estrildidae | *Amadina fasciata* | Cut-throat Finch | LM**C**CVF**C**IF**T**V | UVS | ZMUC 118505 |  | FJ440639 | [3] |
| Passeriformes | Estrildidae | *Neochmia modesta* | Plum-headed Finch | LM**C**CVF**C**IF**T**V | UVS | ANWC 29034 |  | FJ440642 | [3] |
| Passeriformes | Estrildidae | *Taeniopygia guttata* | Zebra Finch | LM**C**CVF**C**IF**T**V | UVS |  |  | AF222331 | [23] |
| Passeriformes | Estrildidae | *Erythrura gouldiae* | Gouldian Finch | LM**C**CVF**C**IF**T**V | UVS | No voucher specimen | EBU 11103 | FJ440640 | [3] |
| Passeriformes | Estrildidae | *Lonchura maja* | White-headed Munia | LM**C**CVF**C**IF**T**V | UVS | ZMUC 118479 |  | FJ440641 | [3] |
| Passeriformes | Motacillidae | *Motacilla flava* (17) | Western Yellow wagtail | LM**C**CVF**C**IF**T**V | UVS | Various sources |  | **HE601859** |  |
| Passeriformes | Motacillidae | *Motacilla citreola citreola* | Citrine Wagtail | LM**C**CVF**C**IF**T**V | UVS | P. Chylarecki | M.cit.3 | **HE601860** |  |
| Passeriformes | Motacillidae | *Motacilla cinerea cinerea* | Grey Wagtail | LM**C**CVF**C**IF**T**V | UVS | T. Sirotkin & A.Ö. | Var03 | **HE601861** |  |
| Passeriformes | Motacillidae | *Motacilla alba alba* | White Wagtail | LM**C**CVF**C**IF**T**V | UVS | C. Hemborg | 3 | **HE601862** |  |
| Passeriformes | Motacillidae | *Motacilla aguimp* | African Pied Wagtail | LM**C**CVF**C**IF**T**V | UVS | M. Wilson & A.Ö. | Ug01 | **HE601863** |  |
| Passeriformes | Motacillidae | *Macronyx croceus* | Yellow-throated Longclaw | XM**C**CVF**C**IF**T**V | UVS | M. Wilson, C. Hemborg, S. Ulfstrand & A.Ö. | Ug59 | **HE601864** |  |
| Passeriformes | Motacillidae | *Anthus cervinus* | Red-throated Pipit | LM**C**CVF**C**IF**T**V | UVS | M. Björklund & A.Ö. | Is09 | **HE601865** |  |
| Passeriformes | Fringillidae | *Serinus canaria* | Atlantic Canary | LM**C**CVF**C**IF**T**V | UVS |  |  | AJ277922 | [34] |
| Passeriformes | Fringillidae | *Himatione sanguinea* | Apapane | LM**C**CVF**C**IF**T**V | UVS | UWBM 65834 | WLK 133 | GQ305968 | [27] |
| Passeriformes | Parulidae | *Dendroica coronata coronata* | Myrtle Warbler | LM**C**CVF**C**IF**T**V | UVS | FMNH 442959 |  | **HE601866** |  |
| Passeriformes | Icteridae | *Icterus galbula* | Baltimore Oriole | LM**C**CVF**C**IF**T**V | UVS | FMNH 442567 |  | GQ305968 | [27] |
| Passeriformes | Icteridae | *Sturnella superciliaris* | White-browed Blackbird | LM**C**CVF**C**IF**T**V | UVS | FMNH 330789 |  | **HE601867** |  |
| Passeriformes | Icteridae | *Sturnella neglecta* | Western Meadowlark | LM**C**CVF**C**IF**T**V | UVS | FMNH 341967 |  | **HE601868** |  |
| Passeriformes | Icteridae | *Xanthocephalus xanthocephalus* | Yellow-headed blackbird | LM**C**CVF**C**IF**T**V | UVS | FMNH 442337 |  | **HE601869** |  |
| Passeriformes | Icteridae | *Dolichonyx oryzivorus* (2) | Bobolink | LM**C**CVF**C**IF**T**V | UVS | UWBM 80613, -5 | EVL 750, -2 | FJ440643 | [3] |
| Passeriformes | Emberizidae | *Emberiza citrinella* | Yellowhammer | LM**C**CVF**C**IF**T**V | UVS | NRM 986194 |  | GQ305971 | [37] |
| Passeriformes | Thraupidae | *Cyanerpes cyaneus* | Red-legged Honeycreeper | LM**C**CVF**C**IF**T**V | UVS | FMNH 391637 |  | GQ305966 | [27] |

^1^Academy of Natural Sciences, Philadelphia (ANSP), Australian Museum (O., EBU), Australian National Wildlife Collection (ANWC), The Field Museum of Natural History, Chicago (FMNH), Los Amigos Biological Station (CICRA), Louisiana State University Museum of Natural Science (LSUMZ), Biology Education Centre at Uppsala University (IBG, UU), Museum of Victoria, Melbourne (MVM), Swedish Museum of Natural History (NRM), University Kansas Museum of Natural History (Kan, KUNHM), University of Washington, Burke Museum (UWBM), Western Australian Museum (WAM), Zoological Museum University of Copenhagen (ZMUC).
