# Supplementary figures and images for: Evolution of ultraviolet vision in the largest avian radiation - the passerines
Source: BMC Evol Biol. 2011 Oct 24;11:313. doi: 10.1186/1471-2148-11-313 (PMC3225180; doi:10.1186/1471-2148-11-313)

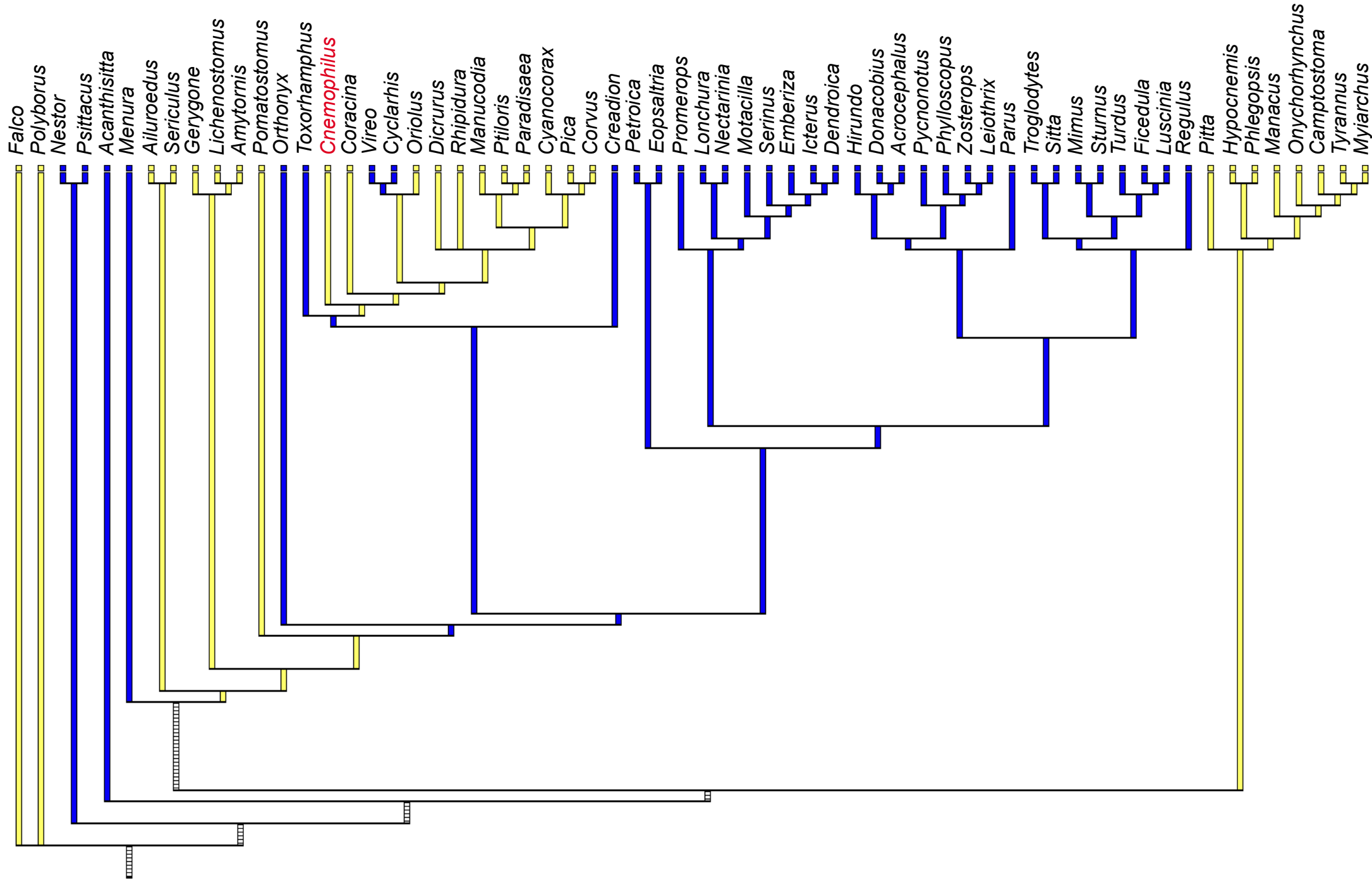

Supplement: Additional file 2 — Alternative recontruction of SWS1 opsin evolution, 1. Same tree as in Figure 2, but with clades b and c, which are reconstructed with low support, collapsed, and Cnemophilus placed as sister to the 'core Corvoidea' clade. VS/UVS optimisation represented by yellow for VS, blue for UVS, and barred for ambiguous. [file 1471-2148-11-313-S2.PDF]

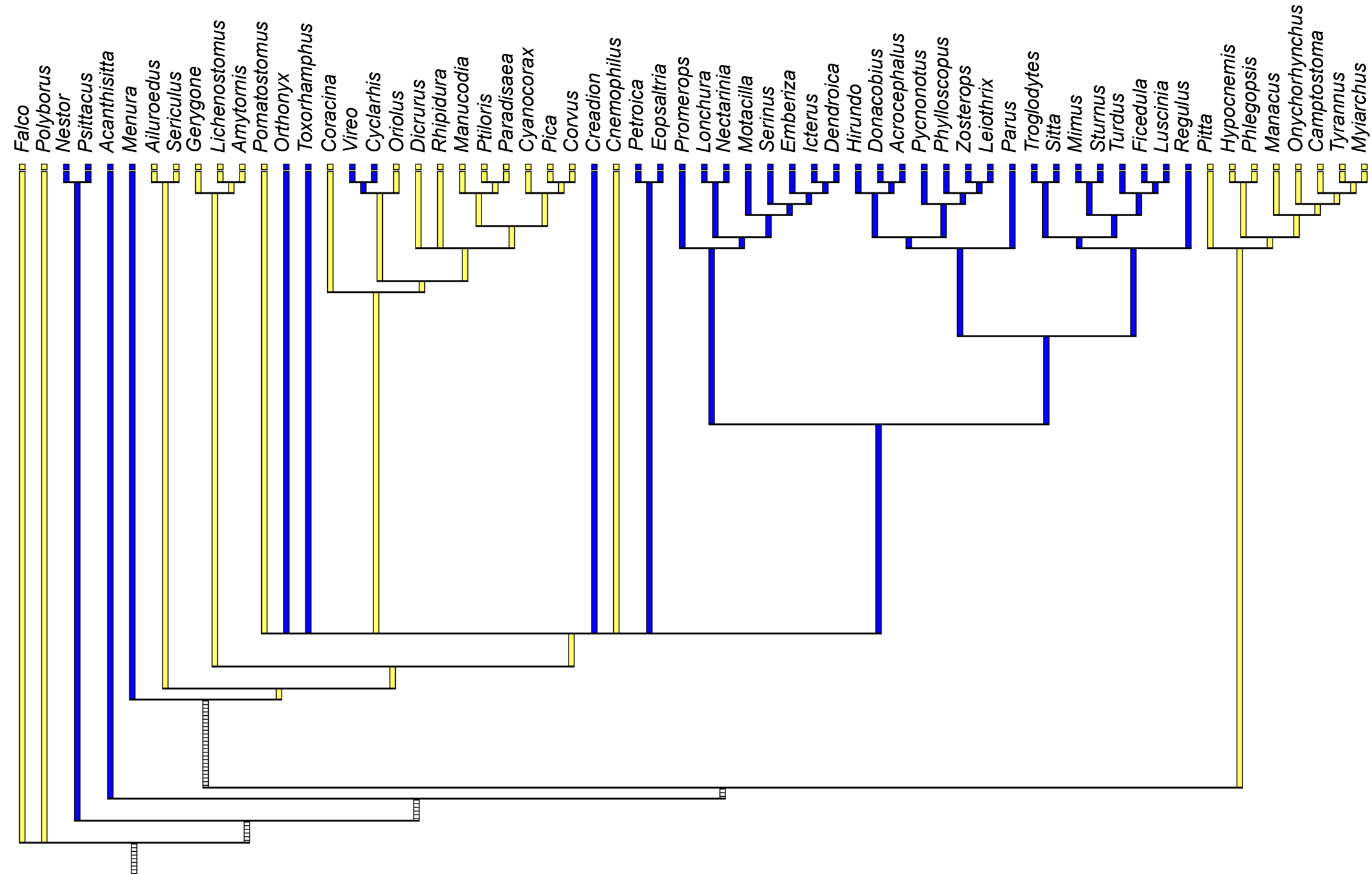

Supplement: Additional file 3 — Alternative recontruction of SWS1 opsin evolution, 2. Same tree as in Figure 2, but with all 'basal' nodes with posterior probability < 0.95 collapsed. VS/UVS optimisation represented by yellow for VS, blue for UVS, and barred for ambiguous. [file 1471-2148-11-313-S3.PDF]
