# Supplementary material for: Evolution of ultraviolet vision in the largest avian radiation - the passerines
Source: BMC Evol Biol. 2011 Oct 24;11:313. doi: 10.1186/1471-2148-11-313 (PMC3225180; doi:10.1186/1471-2148-11-313)
Supplement: Additional file 4 — Sequences used for phylogenetic reconstruction. GenBank numbers for sequences used for phylogenetic reconstruction. [file 1471-2148-11-313-S4.PDF]

Cytb
Polyborus plancus_GI_2231531
Falco_peregrinus_GI_5835946
Tyrannus_savana_GI_25988535
Myiarchus_tyrannulus_GI_25988533
Hypocnemis_cantator_GI_157098226
Phlegopsis_nigromaculata_GI_157098276
Manacus_manacus_GI_25988545
Acanthisitta_chloris_GI_40388199
Regulus_regulus_GI_62942306
Ailuroedus_crassirostris_GI_501125
Zosterops_japonicus_batanis_GI_117307457
Onychorhynchus_coronatus_GI_83031352
Nestor_notabilis_GI_33340943
Leiothrix_argentauris_GI_183585463
Troglodytes_aedon_GI_33867752
Pyrrhura_frontalis_GI_60391145
Luscinia_svecica_GI_32825659
Melopsittacus_undulatus_GI_134034920
Psittacus_erithacus_GI_84626823
Ficedula_hypoleuca_GI_13508727
Menura_novaehollandiae_GI_89255421
Pycnonotus_barbatus_FJ487857
Donacobius_atricapilla_GI_33867714
Hirundo_rustica_GI_71067721
Lichenostomus_flavus_GI_45385581
Cnemophilus_macgregorii_GI_6469738
Coracina_fimbriata_GI_6469734
Dicrurus_bracteatus_GI_225795341
Gerygone_fusca_GI_225795349
Ptiloris_magnificus_GI_34501182
Paradisaea_raggiana_GI_841455
Cyanocorax_chrysops_GI_1841722
Lonchura_cucullata_GI_18378130
Phylloscopus_trochilus_GI_76886185
Turdus_merula_intermedius_GI_187371362
Acrocephalus_scirpaceus_GI_164449763
Sitta_europaea_sinensis_GI_117307475
Mimus_saturninus_GI_34501130
Sturnus_vulgaris_GI_21304617
Creadion_carunculatus_GI_93213508
Petroica_rosea_GI_225795377
Eopsaltria_australis_GI_11139369
Serinus_canaria_GI_17467283
Icterus_galbula_GI_113196961
Motacilla_alba_GI_168805820
Emberiza_citrinella_GI_45685536
Pomatostomus_temporalis_GI_13395
Rhipidura_leucophrys_GI_239508637
Pica_pica_pica_GI_56069742

Manucodia_comrii_GI_840946
Corvus_corone_GI_5712221
Vireo_huttoni_GI_225795397
Dendroica_coronata_GI_206573675
Nectarinia_jugularis_GI_239508506
Amytornis_striatus_GI_45385681
Pitta_baudii_AY064280
Orthonyx_temminckii_AY064275
Sericulus_chrysocephalus_GI_501119
Cyclarhis_gujanensis_gujanensis_GI_14581485

ND2
Falco_peregrinus_GI_5835946
Tyrannus_savana_GU816828
Myiarchus_tuberculifer_FJ175973
Hypocnemis_cantator_GI_169409409
Phlegopsis_nigromaculata_GI_157094415
Manacus_manacus_GU985504
Acanthisitta_chloris_GI_40388199
Regulus_calendula_AY329435
Ailuroedus_crassirostris_GI_23499430
Zosterops_japonicus_GI_23297853
Onychorhynchus_coronatus_GI_83031438
Nestor_notabilis_GI_169787722
Leiothrix_argentauris_GI_113912235
Troglodytes_aedon_GI_39547756
Pyrrhura_picta_AY669444
Luscinia_svecica_HM640927
Melopsittacus_undulatus_GI_134034920
Psittacus_erithacus_GI_169787762
Menura_novaehollandiae_GI_89255421
Pycnonotus_barbatus_GQ369695
Hirundo_rustica_GI_77377885
Lichenostomus_flavus_GI_45385435
Coracina_novaehollandiae_GI_125487330
Dicrurus_bracteatus_GI_125487340
Gerygone_fusca_GU825901
Ptiloris_magnificus_GI_23499452
Paradisaea_raggiana_GI_168986781
Cyanocorax_chrysops_GI_114214848
Lonchura_cucullata_GI_19481416
Phylloscopus_trochilus_GI_19481464
Turdus_merula_GI_58618523
Acrocephalus_scirpaceus_GI_164449763
Sitta_europaea_GI_258674263
Mimus_saturninus_GI_149729348
Sturnus_vulgaris_GI_218684136
Creadion_carunculatus_GI_93213496
Parus_major_AY136587
Petroica_rosea_GI_260176910

Eopsaltria_australis_GI_260176866
Serinus_canaria_GI_169787772
Icterus_galbula_GI_6090645
Motacilla_alba_alboides_GI_56267682
Emberiza_citrinella_GI_256259814
Pomatostomus_temporalis_GI_23499446
Rhipidura_leucophrys_GI_256576249
Pica_pica_GI_125487162
Corvus_corone_GI_46562041
Vireo_leucophrys_AY030134
Camptostoma_obsoletum_GI_170877953
Promerops_cafer_GI_74101520
Oriolus_oriolus_GI_125487168
Dendroica_coronata_GI_55740182
Nectarinia_jugularis_GI_256576023
Amytornis_striatus_GI_45385529
Pitta_versicolor_EF501927
Orthonyx_temminckii_AY064755
Sericulus_chrysocephalus_EU341427
Cyclarhis_gujanensis_ochrocephala_GI_14581527

ODC
Polyborus_plancus_GI_156617609
Tyrannus_savana_GI_90658371
Myiarchus_tyrannulus_GI_90658335
Manacus_manacus_GI_90658333
Acanthisitta_chloris_GI_198449192
Regulus_regulus_GI_196052701
Ailuroedus_dentirostris_EU341453
Zosterops_japonicus_simplex_GI_229905435
Onychorhynchus_occidentalis_EU231853
Leiothrix_argentauris_GI_229905359
Troglodytes_aedon_GU816925
Pyrrhura_frontalis_GI_113120409
Luscinia_svecica_HM633741
Psittacus_erithacus_GU816898
Ficedula_hypoleuca_GI_196052635
Menura_novaehollandiae_GI_148970286
Pycnonotus_barbatus_FJ358086
Donacobius_atricapilla_EU680723
Hirundo_rustica_GI_149393725
Cnemophilus_loriae_GI_161015839
Coracina_novaehollandiae_GI_166209119
Dicrurus_bracteatus_GI_161015813
Ptiloris_magnificus_GI_198449210
Paradisaea_raggiana_GI_260585307
Phylloscopus_trochilus_UO
Turdus_merula_intermedius_GI_187481266
Acrocephalus_scirpaceus_GI_254968136
Sitta_europaea_GI_196052709

Mimus_saturninus_GI_196052663
Sturus_vulgaris_EU680769
Parus_major_EU680749
Eopsaltria_australis_GI_148970278
Icterus_galbula_GI_33321451
Motacilla_alba_alba PA/AÖ
Emberiza_citrinella_citrinella_GI_182894072
Pomatostomus_temporalis_GI_148970298
Rhipidura_rufifrons_EU272115
Manucodia_chalybatus_GI_260585251
Vireo_olivaceus_EU272110
Camptostoma_obsoletum_GI_165873992
Promerops_cafer_GI_196052689
Oriolus_oriolus_GI_196052671
Dendroica_virens_EU325828
Nectarinia_olivacea_GU816904
Toxorhamphus_poliopterus_GI_161015833
Pitta_guajana_GI_111182806
Orthonyx_temminckii_EF441244
Sericulus_chrysocephalus_EU341445
Cyclarhis_gujanensis_EU380435

Myo
Polyborus_plancus_GI_37622381
Falco_mexicanus_EU739964
Tyrannus_savana_GI_32967857
Myiarchus_tyrannulus_GI_90194278
Hypocnemis_cantator_GI_51104560
Phlegopsis_erythroptera_AY677026
Manacus_manacus_GI_34223550
Acanthisitta_chloris_GI_198449177
Regulus_regulus_GI_93211336
Ailuroedus_crassirostris_GI_23499414
Zosterops_japonicus_simplex_GI_253971575
Leiothrix_argentauris_GI_253971503
Pyrrhura_frontalis_GI_37622387
Luscinia_svecica_HM633605
Ficedula_hypoleuca_GI_34538531
Menura_novaehollandiae_GI_23499419
Pycnonotus_barbatus_FJ357985
Donacobius_atricapilla_GI_66876329
Hirundo_rustica_GI_23505414
Lichenostomus_penicillatus_GI_225795283
Cnemophilus_loriae_GI_161015801
Coracina_atriceps_GI_161015791
Dicrurus_bracteatus_GI_225795273
Gerygone_fusca_GI_225795279
Ptiloris_magnificus_GI_23499415
Cyanocorax_chrysops_GI_39979912
Phylloscopus_trochilus_GI_62735879

Turdus_merula_intermedius_GI_167369902
Acrocephalus_scirpaceus_FJ883111
Sitta_europaea_AY064257
Mimus_saturninus_GI_34538539
Sturnus_vulgaris_GI_34538575
Parus_major_AY228310
Petroica_rosea_GI_225795305
Eopsaltria_australis_GI_23499407
Serinus_canicollis_EU878718
Motacilla_alba_AY228307
Emberiza_citrinella_
Pomatostomus_temporalis_GI_23499405
Rhipidura_albiscapa_GI_225795315
Pica_pica_GI_39979924
Manucodia_ater_EU726218
Corvus_corone_GI_253971577
Vireo_huttoni_GI_225795321
Camptostoma_obsoletum_GI_165909109
Promerops_cafer_GI_196052409
Oriolus_oriolus_GI_34538589
Dendroica_coronata_GU932292
Nectarinia_olivacea_GU816931
Amytornis_striatus_GI_225795261
Pitta_guajana_GI_113196725
Toxorhamphus_poliopterus_GI_161015797
Orthonyx_temminckii_AY064728
Cyclarhis_gujanensis_EU380504

RAG-1
Polyborus_plancus_GI_42525312
Falco_peregrinus_AY461399
Tyrannus_savana_GI_241994383
Myiarchus_tyrannulus_GI_241994205
Hypocnemis_cantator_GI_221063185
Phlegopsis_nigromaculata_GI_221063215
Manacus_manacus_GI_241994185
Acanthisitta_chloris_GI_18845829
Regulus_calendula_AY057028
Ailuroedus_crassirostris_GI_23499368
Zosterops_japonicus_FJ358145
Onychorhynchus_coronatus_GI_241994249
Nestor_notabilis_GI_154371702
Leiothrix_argentauris_GI_253971662
Troglodytes_aedon_GI_18845955
Pyrrhura_frontalis_GI_37622373
Psittacus_erithacus_erithacus_GI_94483166
Ficedula_hypoleuca_GI_93211262
Menura_novaehollandiae_GI_18845887
Pycnonotus_barbatus_FJ358152
Donacobius_atricapilla_GI_32480970

Hirundo_rustica_GI_38324397
Lichenostomus_penicillatus_GI_225795433
Cnemophilus_loriae_GI_38324355
Coracina_novaehollandiae_GI_38324361
Dicrurus_bracteatus_GI_225795419
Gerygone_fusca_GI_225795427
Ptiloris_magnificus_GI_38324467
Paradisaea_raggiana_GI_18845907
Lonchura_cantans_GI_157672484
Phylloscopus_collybita_AY319997
Turdus_philomelos_AY307214
Acrocephalus_dumetorum_FJ358146
Sitta_europaea_GI_23499394
Mimus_saturninus_GI_18845130
Sturnus_vulgaris_GI_93211290
Creadion_carunculatus_GI_38324451
Parus_inornatus_AY057017
Petroica_rosea_GI_225795453
Eopsaltria_australis_GI_23499374
Icterus_parisorum_AY056998
Motacilla_cinerea_GI_18845893
Emberiza_schoeniclus_AY056992
Pomatostomus_temporalis_GI_23499384
Rhipidura_albiscapa_GI_225795465
Pica_pica_GI_157672456
Manucodia_chalybatus_GI_38324409
Corvus_corone_GI_19584569
Vireo_huttoni_GI_225795473
Camptostoma_obsoletum_GI_241994067
Promerops_cafer_GI_38324463
Oriolus_larvatus_AY057011
Amytornis_striatus_GI_225795403 (end) + Amytornis_striatus_GU825808
Pitta_guajana_GI_85822701
Toxorhamphus_novaeguineae_GI_18845951
Sericulus_chrysocephalus_GI_171850310

RAG-2
Tyrannus_savana_GI_241994743
Myiarchus_tyrannulus_GI_241994565
Hypocnemis_cantator_GI_221062965
Phlegopsis_nigromaculata_GI_221062995
Manacus_manacus_GI_241994545
Acanthisitta_chloris_GI_38324747
Regulus_calendula_AY443220
Ailuroedus_melanotis_AY443105
Zosterops_senegalensis_AY443247
Onychorhynchus_coronatus_GI_241994609
Nestor_notabilis_GI_154371704
Troglodytes_aedon_GI_38325025
Pyrrhura_melanura_EF517691

Psittacus_erithacus_GI_154371726
Ficedula_hypoleuca_GI_161019552
Menura_novaehollandiae_GI_38324885
Pycnonotus_barbatus_AY443219
Hirundo_rustica_GI_38324853
Cnemophilus_loriae_GI_38324789
Coracina_novaehollandiae_GI_38324799
Dicrurus_hottentottus_AY443141
Gerygone_fusca_GI_289584050
Ptiloris_magnificus_GI_38324977
Paradisaea_raggiana_GI_38324925
Lonchura_cantans_GI_161019580
Phylloscopus_collybita_AY799844
Turdus_philomelos_EF568274
Acrocephalus_newtoni_AY799825
Sitta_carolinensis_AY443227
Mimus_patagonicus_AY443173
Sturnus_vulgaris_GI_38325007
Creadion_carunculatus_GI_38324947
Parus_major_AY443197
Icterus_parisorum_AY443157
Motacilla_cinerea_GI_38324899
Emberiza_schoeniclus_AY443143
Pomatostomus_isidorei_AY443210
Rhipidura_hyperthra_AY443223
Pica_pica_GI_161019546
Manucodia_chalybatus_GI_38324871
Corvus_corone_GI_38324807
Vireo_philadelphia_AY443245
Camptostoma_obsoletum_GI_241994427
Promerops_cafer_GI_38324967
Oriolus_xanthonotus_AY443185
Nectarinia_olivacea_AY443180
Pitta_guajana_GI_85822629
Toxorhamphus_novaeguineae_GI_38325019
Orthonyx_temminckii_AY443187

c-myc
Polyborus_plancus_GI_37622357
Falco_rufigularis_DQ881899
Tyrannus_savana_GI_13183494
Myiarchus_tyrannulus_GI_25988479
Manacus_manacus_GI_25988485
Acanthisitta_chloris_GI_18845102
Regulus_regulus_GI_157672388
Ailuroedus_crassirostris_GI_23499346
Zosterops_poliogastrus_EF568223
Troglodytes_troglodytes_EF568213
Pyrrhura_frontalis_GI_37622361
Ficedula_hypoleuca_GI_157672364

Menura_novaehollandiae_GI_13183468
Pycnonotus_xanthopygos_EF568222
Hirundo_rustica_GI_21304761
Dicrurus_balicassius_AF377275
Ptiloris_magnificus_GI_23499364
Lonchura_cantans_GI_157672428
Turdus_philomelos_EF568203
Sitta_europaea_GI_21304755
Mimus_saturninus_GI_21304751
Sturnus_vulgaris_GI_21304749
Parus_caeruleus_EF568215
Petroica_rosea_GI_256532142
Eopsaltria_australis_GI_256532098
Serinus_canaria_GI_213735
Motacilla_alba_GI_157672418
Emberiza_citrinella_GI_157672446
Pomatostomus_temporalis_GI_23499360
Pica_pica_GI_157672336
Manucodia_keraudrenii_EF568196
Corvus_cornix_GI_21304769
Oriolus_oriolus_GI_21304773
Nectarinia_cuprea_EF568231
Pitta_baudii_AF295177
Orthonyx_temminckii_AY064286

TGFß2
Falco_peregrinus_GI_194246182
Tyrannus_tyrannus_EU737471
Manacus_manacus_EU522614
Acanthisitta_chloris_GI_193878438
Regulus_calendula_EU737446
Zosterops_japonicus_GI_220900551
Nestor_notabilis_GI_194246212
Pyrrhura_picta_EU660298
Psittacus_erithacus_GI_193878675
Ficedula_hypoleuca_GI_20514066
Menura_novaehollandiae_GI_193878595
Pycnonotus_barbatus_GU112572
Dicrurus_hottentottus_GQ145460
Cyanocorax_chrysops_GI_224593457
Turdus_falklandii_EU737469
Mimus_saturninus_GI_151336032
Sturnus_vulgaris_GI_151335998
Serinus_canaria_GI_194246258
Motacilla_aguimp_EU878633
Rhipidura_albiscapa_GI_256576385
Corvus_corone_GI_193878432
Dendroica_coronata_EU815801
Nectarinia_jugularis_GQ145313
Amytornis_striatus_GI_213386514

Pitta_guajana_GI_193878663
Sericulus_chrysocephalus_EU341482
