# Supplementary material for: Evolution of ultraviolet vision in the largest avian radiation - the passerines
Source: BMC Evol Biol. 2011 Oct 24;11:313. doi: 10.1186/1471-2148-11-313 (PMC3225180; doi:10.1186/1471-2148-11-313)
Supplement: Additional file 5 — Sequence alignment. Alignment of sequences used for phylogenetic reconstruction. Details in Additional file 4. [file 1471-2148-11-313-S5.PDF]

```
#NEXUS

Begin DATA;
Dimensions ntax=62 nchar=9082;
Format datatype=dna missing=? gap=- interleave=yes;
Matrix

[cytochrome b]
Polyborus_plancus
ATGGCCCCCAATATCCGAAAATCCCACCCCCTACTAAAAATAGTCAACAACTCCCTAATTGACCTTCCCACCCCCTCAAA
TATCTCCGCCTGATGAAACTTCGGATCTTTACTAGGTATCTGCCTTGTAACCCAAATCCTAACAGGCCTACTTCTAGCCA
TACACTACACTGCAGACACAAACCTAGCCTTCTCATCAGTCTCCCATACATGCCGAAACGTACAATACGGCTGACTAATC
CGCAATCTACACGCCAACGGAGCCTCACTTTTCTTCATCTGCATCTACATGCACATCGGACGAGGTATCTACTATGGCTC
CTACCTCTACAAAGAAACCTGAAACACAGGCATCATCCTCCTACTCACCCTAATAGCGACAGCCTTCGTGGGTTATGTCC
TACCCTGAGGACAAATATCCTTCTGAGGAGCTACAGTCATCACCAACTTATTTTCAGCCATTCCCTACATCGGCCAAACC
CTTGTCGAATGGGCCTGAGGAGGCTTCTCCGTGGATAACCCAACATTAACCCGATTCTTCGCCCTACACTTCCTACTGCC
ATTCCTAATCGCAGGCCTTACTCTAATCCACCTGACCTTCCTACACGAATCAGGCTCAAACAACCCCCTGGGAATCACAT
CAAACTGCGACAAAATCCCATTCCATCCCTACTTCTCCTCAAAAGATATCCTAGGATTCATACTCCTATACTTCCTACTA
ACAACCCTAGCCCTATTCTCCCCCAACCTCCTAGGAGACCCAGAAAACTTCACCCCAGCAAACCCCCTAGTCACACCTCC
CCATATCAAACCCGAATGATACTTCCTATTTGCCTACGCCATCCTACGCTCAATCCCCAACAAACTAGGCGGAGTACTAG
CACTTGCAGCTTCCATTCTAATCCTGTTCCTAAGCCCTTTCCTCCACAAATCCAAACAACGCACAATAACCTTCCGACCA
CTATCCCAAATACTATTCTGACTCCTAGTCACCAACCTACTTATCCTAACATGAATTGGCAGCCAACCCGTAGAACACCC
ATTCATCATTATCGGCCAACTAGCCTCCCTCACCTACTTCACAATTCTCCTAATCCTCCTCCCCCTCACCGGAGCCCTAG
AAAACAAAATCCTCAACTACTAA
Falco
ATGGCACCCAACATTCGAAAATCACACCCTTTAGTAAAAATAATCAATAACTCCCTAATTGACCTTCCCACTCCACCCAA
CATCTCCATATGATGAAACTTTGGATCCCTACTAGGAATCTGTCTAGCCACCCAAATCCTAACTGGCCTATTGCTAGCCA
TACACTACACAGCGGATACAACCCTAGCCTTTTCATCCGTCGCCCACACATGTCGAAACGTACAATACGGATGACTAATC
CGCAACCTACATGCCAACGGAGCATCCTTATTCTTCATCTGTATCTACATACACATTGGGCGAGGCATTTACTACGGCTC
TTATTTGTATAAAGAAACCTGGAATACAGGCATCATCCTTCTACTCACCCTAATAGCAACAGCCTTCGTCGGCTATGTAC
TACCCTGAGGACAAATATCATTCTGAGGAGCCACAGTCATTACCAACCTATTCTCAGCAATCCCATACATCGGCCAAACC
CTAGTCGAATGAGCTTGAGGGGGATTTTCAGTAGACAACCCAACACTGACCCGATTCTTCGCCCTACACTTCCTACTTCC
ATTCCTAATCGCAGGACTCACCCTAATCCACCTCACCTTCCTACATGAATCAGGCTCAAATAACCCCCTAGGAATCACAT
CAAATTGCGACAAAATCCCATTCCACCCATACTACTCTCTCAAAGATATCCTAGGATTTATACTCATATACCTGCCCCTA
ATAACCCTAGCCCTATTTACCCCAAACCTGCTAGGAGACCCAGAAAACTTTACACCAGCAAATCCCTTAGTCACCCCCCC
ACACATCAAACCAGAATGATACTTCCTATTTGCTTACGCCATCCTACGCTCAATCCCCAATAAACTGGGCGGAGTCCTAG
CACTAGCCGCCTCAGTACTAATCTTATTCCTGAGTCCACTACTTCACAAATCCAAACAACGCACAATAACCTTCCGCCCT
CTATCCCAGTCACTATTCTGACTTCTAGTCACCAACCTACTCATCCTAACCTGAGTAGGAAGCCAACCCGTTGAGCACCC
GTTCATCATCATTGGCCAACTAGCTTCACTCTCCTACTTCACAACCCTTCTAATCCTCCTCCCCCTTACCGGGGCCCTAG
AAAACAAAATCCTAAACTACTAA
Tyrannus
????????????????????????????????????????????????????????????????????????????????
????????????????????????????????????????????????????????????????GGCCTTCTTCTAGCAA
TACACTACACCGCAGACACTTCCCTAGCCTTCACTTCTGTTGCCCACACATGCCGAAATGTACAATTCGGCTGACTCCTA
CGAAACCTTCACGCAAACGGAGCATCTTTCTTTTTCATCTGCATCTACCTGCACATTGGACGAGGATTCTACTACGGGTC
TTACCTGTATAAAGAAACCTGAAACACCGGCGTTATCCTCCTATTGACTCTAATAGCAACCGCTTTCGTCGGCTACGTCC
TCCCATGAGGACAAATGTCTTTTTGAGGAGCTACAGTAATTACCAACCTATTCTCAGCGATCCCCTACATCGGCCAAACA
CTTGTAGAATGGGCCTGAGGTGGATTCTCTGTTGATAACCCTACACTCACCCGATTTTTCGCCCTCCACTTCCTCCTACC
ATTCGCAATTGCAGGTCTCACATTCATCCATCTAACCTTCCTGCATGAAACAGGATCAAACAACCCCCTCGGCATTCACT
CAGATTGTGATAAAATCCCATTCCATCCATACTTCTCTATAAAAGATATTCTAGGATTCATCATCCTCCTCCTCCCACTA
ATAACCCTAGCTATATTTTCACCCAACCTTCTAGGTGACCCTGAAAATTTCACGCCCGCCAACCCACTAACAACCCCTCC
CCACATCAAGCCAGAATGATATTTCCTATTCGCATATGCTATCCTACGATCCATTCCCAACAAACTCGGAGGAGTATTAG
CCCTCGCCGCCTCCGTCCTAGTACTATTTCTAGCCCCATTCCTCCACATGTCAAAGCAACGCACAATAACCTTCCGCCCT
CTTTCCCAATTCCTATTTTGAATCCTAGTAACTAACCTCCTCATCCTCACATGAATTGGTAGTCAACCAGTGGAACACCC
ATTCATCATTATCGGCCAACTAGCCTCATTCACTTACTTCACTATCATCCTCATTTTATTCCCAACTATCGGCATGCTAG
AAAATAAACTACTAAAACTTTAA
Myiarchus
????????????????????????????????????????????????????????????????????????????????
????????????????????????????????????????????????????????????????GGCCTCCTCCTAGCAA
TACACTACACTGCAGACACCTCCCTAGCTTTCACCTCAGTCGCCCACACATGCCGAAACGTCCAATTTGGTTGACTCATT
```

CGGAATCTCCATGCAAACGGAGCATCCCTCTTTTTATTTGCATTTATCTACACATCGGACGAGGATTCTACTATGGGTC
CTATCTGTATAAAGAAACCTGAAACACAGGTGTTATCCTCCTCCTAACCCTAATAGCAACTGCATTCGTCGGCTATGTTC
TCCCATGAGGACAAATATCATTCTGAGGCGCTACAGTAATCACTAACCTATTTTCAGCCATTCCCTATATCGGCCAAACA
CTCGTAGAATGAGCCTGAGGTGGATTCTCAGTCGACAACCCAACACTCACCCGATTCTTTGCCCTCCACTTCCTCCTACC
ATTTGCTATCGCAGGTATCACATTCATCCACCTTACATTTTTACACGAAACAGGATCAAACAATCCCCTAGGAATCTCCT
CAGACTGTGACAAAATCCCATTCCACCCATACTTCTCCACAAAAGACATCCTAGGCTTCATTATCCTCCTTCTCCCATTA
ATAACACTCGCCATATTTTTACCTAACCTTCTAGGCGACCCCGAAAATTTCACGCCCGCCAACCCACTAACAACTCCACC
CCATATCAAACCAGAGTGATATTTCCTTTTTGCATATGCCATTTTACGATCCATCCCTAATAAACTCGGAGGAGTCTTAG
CCCTCGCTGCCTCCGTCCTAGTTCTATTCCTAACCCCATTCCTACACATATCAAAACAACGCACAATAACCTTTCGTCCT
CTCTCCCAACTCCTATTCTGAATTCTAGTAACTAACCTCCTCATCCTTACATGAATTGGAAGCCAACCAGTAGAACATCC
ATTTATTATTATTGGCCAACTAGCCTCCTTCATCTACTTTACCATCCTCCTAATTCTATTTCCAATTATCGGAACACTAG
AAAACAAACTACTAAAATTCTAA
Hypocnemis_cantator
??
??????????????????TTTTGGATCCCTCCTTGGCATCTGCCTCATATCCCAAATCATTACCGGCTTACTAATAGCCA
TACATTACACAGCAGACACTACCCTAGCTTTCACATCAATTGCCCACACTTGCCGAAACGTCCAATTTGGATGACTAATC
CGAAACCTCCACGCAAACGGAGCTTCCCTATTCTTCATCTGCATCTACCTCCACATTGGACGAGGACTATACTATGGCTC
CTACCTATACAAAGATACCTGAAACACCGGAGTAATCCTACTACTAACTCTAATAGCAACAGCCTTCGTAGGTTACGTCC
TCCCCTGAGGACAAATATCATTTTGAGGAGCAACCGTCATCACCAACCTATTCTCAGCCATTCCCTACATCGGACAAACA
CTTGTAGAGTGGGCTTGAGGGGGCTTTTCAGTCGACAATCCAACCCTAACACGATTCTTTGCTCTCCACTTTTTACTCCC
CTTCATCATCGCAAGCCTTACACTCATCCACCTAACCTTCTTACACGAAACTGGATCAAACAATCCCCTAGGCATCTCAT
CAAACTGCGATAAAATCCCATTCCACCCCTATTTTTCCTCAAAAGACATCCTAGGCTTCATAGCCATATTCCTACCCCTC
ATATTCCTCACCATATTTTCACCCAACCTTCTAGGCGACCCAGAAAATTTACACCTGCAAACCCACTAGTGACCCCTCC
CCATATCAAACCCGAATGATACTTTCTATTCGCATATGCTATCTTACGATCTATCCCAAATAAACTAGGAGGAGTTCTCG
CCCTAGTTGCCTCCATCCTCATCCTATTCCTCATTCCCTTCCTACATAAATCGAAACAACGAACTATAACCTTCCGGCCC
CTCTCCCAACTCATATTCTGAACCTTAGTATCCAACCTCCTAATCCTGACATGAGTAGGCAGCCAACCAGTAGAACACCC
GTTCATTATCATCGGACAGCTAGCTTCACTCACCTACTTCTCAACTCTCCTAGTCCTATTCCCAATCATCGGAGCCCTGG
AGAACAAAATACTTAACCTCTAA
Phlegopsis
??
??????????????????TTTCGGATCCCTTCTTGGTATCTGCCTTATTACCCAAATTGTCACAGGCCTATTAATAGCAA
TGCACTACACAGCAGACACCACCCTAGCCTTTACATCAGTTGCCCACACTTGTCGAAATGTTCAATTTGGATGACTAATC
CGAAACCTCCATGCAAACGGAGCCTCAATATTCTTCATCTGCATCTATCTCCATATTGGACGAGGACTCTATTATGGCTC
CTACCTTTACAAAGAAACCTGAAACACCGGAGTAATCCTTCTACTAACTCTCATAGCAACAGCCTTCGTAGGATATGTCC
TCCCCTGAGGACAAATATCATTCTGAGGAGCAACCGTCATCACCAACTTATTCTCAGCAATCCCCTATATCGGACAAACA
CTCGTAGAATGGGCCTGAGGAGGATTTTCAGTCGACAATCCTACACTAACACGATTTTTCGCTCTCCACTTTCTGCTCCC
CTTTATCATCGCAGGCCTTACATTCATCCACTTAACTTTCCTGCACGAAACCGGATCAAACAACCCCTTAGGCATTCCAT
CAAACTGTGATAAAATTCCATTCCACCCCTACTTCTCCTCAAAAGACATTCTAGGCTTCATAGCCATACTCCTACCCCTC
ATATTCCTCGCCATATTCTCACCAAACCTCCTAGGCGACCCAGAAAATTTACACCCGCAAACCCATTAGTAACCCCTCC
CCATATCAAACCTGAATGATATTTTCTATTTGCATATGCCATCTTACGATCTATCCCAAACAAACTAGGAGGAGTCCTCG
CCCTCGCCGCCTCCGTCCTCGTCCTATTCCTAATTCCCTTCTTACATAAATCGAAACAACGAACAATAACCTTCCGGCCC
TTCTCCCAACTCATATTCTGAACCCTAGTATCTAACCTTCTAATTCTTACATGAGTGGGTAGCCAACCAGTAGAACATCC
ATTCATCATCATCGGCCAACTAGCTTCACTCACCTATTTTATAACCCTCCTAGTTCTATTTCCAATCATCGGAGCCTTAG
AGAACAAAATACTTAACCTCTAA
Manacus_manacus
??
??GGCCTCCTACTAGCAA
TACATTATACAGCGGATATCACCCTGGCATTCACGTCCGTCGCCCATACATGCCGAAATGTTCAATTCGGCTGACTAATC
CGAAACCTCCATGCAAACGGCGCCTCCTTCTTCTTCATATGCATTTACCTACACATTGGACGAGGATTCTACTATGGTTC
CTACCTATATAAAGAAACCTGAAACACAGGAGTTATTCTCCTCTTAACTCTAATAGCAACTGCCTTCGTAGGATATGTCC
TCCCATGAGGCCAAATATCATTCTGAGGCGCTACAGTAATTACTAACCTATTCTCAGCAATTCCTTACATTGGCCAAACA
CTTGTAGAATGAGCTTGAGGAGGATTCTCAGTTGACAACCCTACACTTACCCGGTTCTTTGCCCTCCACTTCCTCCTCCC
ATTCTTAATTGCAGGTCTCACCCTCATCCACTTAACTTTCTTACACGAAACAGGCTCAAACAACCCCCTAGGTATTTATT
CAGACTGCGACAAAATCCCATTCCACCCCTATTTCTTAATAAAAGATATTCTAGGCCTTTTGTTCCTCCTCATTCCTCTA
ATAACACTTGCCATATTCTCACCCAACCTCTTAGGAGATCCAGAAAATTTACACCTGCAAACCCATTAGTAACACCTCC
CCACATTAAACCTGAGTGGTACTTCCTATTTGCATATGCCATCCTACGATCAATCCCCAACAAATTAGGAGGAGTCCTAG
CCCTAGCCGCCTCCGTCTTAATTCTATTTCTAGCCCCATTCCTCCACATGTCAAAGCAACGAACTATAACCTTCCGACCT
CTCTCCCAACTTCTATTCTGAATCCTAGTAACAAATCTTTTAATCCTTACATGAATCGGTAGCCAACCAGTCGAACATCC
ATTTATTATCATCGGACAACTCGCCTCAACTACCTACTTCACAATCATTCTCATCTTATTCCCCATTACTAGTCTCCTAG
AAAATAAGATACTCAACCTCTAA

Acanthisitta_chloris
ATGGCCCCAAACATCCGCAAACACCACCCCCTCCTAAAAATAGTCAACGACTCCCTAATCGACCTACCAACCCCCTCAAA
CATTTCAGCCTGATGAAATTTCGGCTCCCTCTTAGGGATCTGCCTCATAACACAAATCATCACAGGCCTGCTACTAGCAA
CCCACTACACAGCGGACACCTCCTTAGCCTTCGCATCCGTTGCACACACATGCCGAAACGTCCAATTCGGCTGATTAATC
CGCAATCTCCATGCAAACGGAGCTTCATTCTTCTTTATCTGTATCTACCTACATATTGGACGAGGCCTCTATTACGGTTC
CTACCTATATAAAGAAACCTGAAACACAGGAGTCATCCTATTACTCACCCTCATAGCAACCGCCTTTGTCGGCTATGTCC
TTCCGTGAGGACAAATATCGTTCTGAGGAGCCACCGTCATCACCAACCTATTTTCAGCCATCCCCTACATCGGACAAACA
CTTGTAGAATGAGCCTGAGGCGGATTCTCAGTAGACAACCCCACCCTCACTCGATTCTTCGCCCTTCACTTCCTCCTCCC
CTTTGCAATCTCAGGACTAGTATTCATTCACCTAACCTTCCTCCACGAAACAGGATCAAACAACCCCCTGGGCATCTCCT
CAAACTGTGACAAAATCCCATTCCACCCATACTTCTCAACAAAAGACCTCCTAGGTTTCACAATCATATTTATTCCACTA
ATAACCCTAGCCCTATTCTCCCCTAACCTCTTAGGAGACCCAGAAAATTTCACCCCAGCAAACCCATTAGTCACACCCCC
CCACATCAAACCAGAGTGGTACTTCCTATTTGCTTACGCCATCCTCCGATCCATCCCCAACAAACTAGGAGGCGTCCTTG
CCCTCGCCGCCTCCATTCTAGTCCTCTTCCTCAGCCCCCTCCTACATAAATCAAAACAACGCACAATAACCTTCCGACCT
ATATCCCAACTACTATTCTGAATCCTAATCTCCAACCTCCTCATCCTCACATGAATCGGAAGCCAACCAGTTGAACATCC
CTTCATCATCATTGGCCAACTGGCCTCCCTAACCTACTTCACAACCCTCCTTATCCTATTCCCCATTATCGGAGCCCTCG
AAAACAAAATACTTAATCTCTAA
Regulus
ATGGCACCCAATCTACGTAAAAACCACCCTCTAATAAAAATCGTCAACGATGCTCTAATCGATCTCCCTACACCATCGAA
CATCTCATCATGATGAAACTTCGGCTCACTCTTAGGAATCTGCCTAGTAACACAAATCGTCACAGGACTATTACTGGCAA
TACACTACACAGCAGACACCAACCTAGCCTTCTCCTCCGTTTCACACATGTGCCGAAATGTCCAATTCGGCTGACTCATC
CGTAACCTTCATGCAAACGGAGCATCATTTTTCTTCATCTGCATCTACTTCCATATTGGCCGAGGATTTTACTACGGCTC
CTACCTAAATAAAGAAACTTGAAACGTAGGAGTAATCCTCCTCCTAGCACTAATAGCAACCGCTTTCGTAGGCTACGTCC
TACCATGAGGACAGATATCATTCTGAGGCGCAACAGTAATCACTAACCTATTTTCAGCCATCCCATACATTGGACAAACA
CTGGTAGAATGAGCCTGAGGTGGATTCTCTGTCGACAACCCAACTCTCACTCGATTCTTCGCTCTACACTTCCTACTACC
ATTCGTCATCGCAGGCCTCACTCTAGTCCACCTAACCTTCCTACACGAAACAGGATCTAACAATCCCCTAGGTATTCCAT
CAGACTGTGACAAAATCCCATTCCACCCCTACTACTCCACAAAAGACTTCCTAGGATTCCTGCTAATGTTTATTCCACTA
GCCGCCCTAGCCCTATTTTCACCAAACCTCCTAGGAGACCCAGAAAACTTCACTCCAGCTAACCCACTAGCAACACCACC
ACATATCAAACCTGAATGATACTTCCTATTTGCATATGCCATCCTACGCTCAATCCCAAACAAACTGGGGGGAGTCCTAG
CTCTAGCTGCATCAGTACTAGTCCTATTCCTCATGCCACTGCTACACACCTCTAAACTGCGCTCAATGACCTTCCGTCCG
CTGTCACAAATCCTGTTCTGAACCCTAGTAGCCAACCTCCTAATCCTAACCTGAGTCGGCAGCCAACCAGTTGAACATCC
ATTCATCATCATTGGACAACTAGCCTCATTCTCCTACTTCACCATTATTCTAGTTCTATTCCCACTGGCTGGTATCCTAG
AAAACAAAATACTGAAACTATAA
Ailuroedus
??
???????????????????TTTGGATCCCTTCTAGGAATCTGCCTAGTAATCCAAATCATCACAGGCCTACTACTAGCCA
CACATTACACTGCAGACACCAACCTAGCCTTCGCCTCAGTCGCACATATTTGCCGAGACGTACAGTTTGGTTGATTAATC
CGAAACCTACATGCAAACGGAGCTTCATTCTTCTTCATCTGCATCTACCTGCACATCGGACGAGGACTTTACTACGGCTC
ATACCTCAACAAAAAGACCTGAAACATTGGAGTAATCCTACTACTGCTCCTAATAGCAACTGCCTTCGTAGGCTA?????
?CCCCTGAGGACAAATATCATTCTGAGGGGCAACAGTCATTACCAACCTACTCTCAGCTATCCCATACATCGGACAAACA
CTTGTAGAATGGGCATGAGGCGGATTCTCAGTAGACAACCCGACACTGACCCGATTCTTCGCCCTACACTTCCTTCTACC
ATTTGTTATCGCAGGCCTCACACTAGTCCACCTTACATTCTTACACGAAACAGGATCCAACAACCC?CTAGGCATTCCAT
CAGACTG?GACAAAATTCCATTCCACCCATACTAC???????????ATTCTAGGATTCGCATTAATATTCACCTTACTA
GTTGCCATGGCCTTATTCTCCCCAAACATGCTTGGAGACCCAGAAAACTTCACCCCAGCCAACCC??????CACACCCCC
ACATATCAAACCAGAATGGTACTTCCTATTCGCATATGCAATCCTACGATCCATCCCCAACAAACTTGGAGGGGTCCTAG
CCCTGGCCGCATCCGTACTAGTGCTATTCCTAGTCCCCCTGCTCCATACAT??AAACAACGATCAATAACTTTCCGACCC
CTGTCCCAAATCCTATTCTGAACACTAGTTGCTAATCTACTAGTCTAT????????????????????????????????
??
???????????????????????
Zosterops
ATGGCTCTCAATCTTCGTAAAAACCACCCCTTACTAAAAATCATCAACGATTCCCTAATCGATCTTCCAACTCCATCAAA
CATCTCAACTTGATGAAACTTCGGATCACTCTTAGGCATCTGTCTAATCACACAAATCGTCACAGGCCTACTACTAGCTA
CACACTACACAGCAGATACCAACCTAGCCTTTGCTTCAGTAGCCCACACATGCCGAAATGTACAGTTCGGCTGACTAATC
CGCAACCTACATGCAAACGGCGCTTCATTCTTCTTCATCTGCATCTACTTCCACATCGGCCGAGGCTTTTATTATGGCTC
CTACCTAAATAAAGAAACCTGAAACGTAGGAGTTGTCCTTCTACTAGCCCTGATGGCCACCGCATTCGTAGGATACGTCC
TACCCTGAGGACAAATATCATTCTGAGGTGCCACAGTAATCACTAACCTATTCTCTGCAATCCCATACATCGGGCAAACT
TTAGTAGAATGAGCTTGAGGAGGATTCTCAGTAGACAACCCTACACTGACCCGATTCTTTGCTCTACACTTCCTACTTCC
TTTCGTAATCGCAGGCCTTACATTAGTCCACCTAACCCTACTGCACGAAACAGGATCAAACAACCCCCTTGGCATCCCAT
CAGACTGTGACAAAATCCCATTCCACCCATACTACTCTATCAAAGATATTCTAGGCTTCGCACTTATATTCACCGCATTA
GCCTCTGTAGCCCTATTCGCTCCTAACCTACTAGGGGACCCGGAAAATTTCACGCCCGCCAACCCCCTAGCAACACCACC
ACACATTAAGCCCGAATGATACTTCCTATTTGCTTACGCCATCCTACGATCTATCCCAAACAAACTGGGAGGCGTACTAG

CTCTAGCCGCCTCAGTGCTGGTCCTATTCCTAGTCCCACTATTACACACGTCCAAGCTACGTTCAATGACCTTCCGACCT
CTTTCACAAATCCTGTTCTGAACCCTAGTAGCTAACCTCCTTGTCCTCACCTGAGTAGGCAGCCAACCAGTTGAACACCC
ATTCATCATCATCGGTCAACTAGCCTCATTCACCTATTTCACCATCATTCTAGTCCTCTTCCCACTCGTATCCATCCTAG
AAAATAAACTACTCAAACTATAA
Onychorhynchus
??
??????????????????CTTTGGGTCCCTCCTAGGCATCTGCCTAATGACACAAATCATTACCGGCCTCCTTCTCGCAA
TACATTACACAGCAGATACTTCCCTAGCATTCACATCCGTAGCCCACACGTGCCGAAACGTTCAATTTGGCTGATTAATT
CGAAACCTTCATGCAAACGGAGCATCCTTCTTCTTCATCTGCATCTACCTTCATATCGGACGAGGATTCTACTACGGCTC
CTATCTCTATAAAGAGACCTGGAACACAGGAATTATCCTCCTCCTAACCCTAATAGCAACCGCCTTTGTCGGTTACGTTC
TCCCCTGGGGCCAAATATCATTCTGAGGTGCCACAGTAATTACCAACCTATTTTCTGCCATCCCGTACATCGGACAAACA
CTCGTAGAATGAGCCTGAGGGGGATTCTCTGTTGACAACCCCACGCTCACTCGATTCTTCGCTCTCCATTTTTTACTTCC
CTTTGCTATTGCAGGCCTTACATTCATCCACTTAACCTTCCTACACGAGACAGGCTCCAACAACCCACTCGGCATCTCCT
CAGACTGTGACAAAATTCCATTCCACCCGTACTTTTCCATAAAAGATGCTCTAGGCTTTATTCTCCTCCTCCTCCCACTA
ATAACCCTAGCTATATTCTCACCCAACCTCCTAGGAGACCCAGAAAACTTTACTCCAGCAAACCCACTAGTAACACCTCC
ACATATCAAACCCGAATGATACTTCCTATTTGCCTATGCAATCTTACGATCTATCCCAAACAAACTAGGTGGGGTCCTAG
CCCTTGCTGCCTCTGTCCTAGTCTTATTCCTAACCCCCTTCCTCCATATATCAAAACAACGCACAATAATCTTTCGCCCC
CTCTCTCAAATACTATTCTGAACCCTAGTAACTAATCTCCTCATTCTCACATGAATCGGTAGCCAACCAGTAGAACACCC
ATTTATCATCATTGGTCAACTAGCCTCCTTCTCTTACTTTATAATCCTTATTGTCCTATTCCCTATTATCGGAGCTTTAG
AGAACAAAATCCTCAACCTCTAA
Nestor_notabilis
??
??????????????????????????????CCTAGGCATTTGCTTAACAACCCAAATCCTAACAGGTCTACTCTTAGCCT
CCCATTATACTGCAGATACCTCTCTAGCTTTCTCCTCCGTGGCTAACATATGCCGAAATGTACAATACGGCTGACTAATC
CGCAACCTACACGCAAACGGAGCCTCATTCTTCTTTATCTGTATTTATCTACACATCGCCCGGGGCTTCTATTACGGCTC
ATATCTTTACAAAGAAACCTGAAACACAGGAGTTATCTTACTATTAACTCTCATAGCAACCGCTTTCGTTGGCTATGTTC
TCCCATGAGGCCAGATATCGTTCTGAGGTGCCACAGTTATTACCAACCTATTCTCTGCCATCCCATATATCGGACAAACC
TTAGTAGAATGAGCCTGAGGTGGATTCTCCGTAGACAATCCCACCCTGACCCGATTTTTCACTCTGCACTTCCTCCTTCC
ATTCATAATCACCAGCCTAGTACTTATCCACCTAACATTCCTACATGAATCAGGATCAAACAACCCCCTAGGAATCTCCT
CAAATTGTGACAAAATCCCATTCCACCCCTACTTCTCCCTAAAAGATCTTCTAGGATTTACAATCATATTTTTCCTACTC
ACCACCCTAGCCCTATTTTCCCCAAACCTGTTAGGAGATCCAGAAAACTTTACACCAGCAAATCCTTTAGTCACCCCACC
ACACATTAAACCAGAATGATACTTCCTGTTTGCATACGCAATCCTACGCTCAATTCCAAACAAACTGGGAGGAGTCCTAG
CCTTAGCGGCCTCTGTACTAATTCTATTCCTAA?CCCCCTTCTACACAAATCCAAACAGCGCACAATAGCCTTTCGTCCT
GCCTCTCAACTACTATTCTGAACTCTAGCCGCTAATCTATTCATCCTAACATGAGTAGGGAGTCAACCAGTAG???????
??
???????????????????????
Leiothrix_argentauris
??
??CTACTAGCAA
TACACTACTCAGCAGACACTAACCTGGCCTTCGAATCCGTTGCTCACACGTGCCGAAACGTCCAATATGGCTGACTAATT
CGAAACCTGCACGCGAACGGAGCCTCACTTTTCTTCATTTGTATCTACTTCCACATCGGCCGAGGACTCTACTACGGATC
TTACCTAAATAAAGAGACATGAAACATCGGAGTTGTACTATTACTTACACTTATAGCCACCGCCTTTGTTGGTTACGTAC
TGCCCTGAGGACAAATATCATTCTGAGCGGCTACAGTAATTACAAATCTGTTCTCAGCAATCCCTTACATCGGTCAAACC
CTAGTAGAATGACTGTGAGGAGGATTCTCAGTAGACAACCCCACCCTAACCCGATTTTTCGCCATCCACTTCCTCCTCCC
ATTCGTCATTGCAGGTCTCACGCTAGTCCACCTTACCCTCCTGCATGAAACAGGATCAAACAACCCCCTAGGAATCCCCT
CAGACTGCGATAAAATCCCATTCCATCCCTACTACACCATCAAAGACATCCTGGGATTCGTACTCATGTTCTCCCTACTA
GCTGCTTTAGCCCTATTCGCCCCAAACCTGCTAGGAGACCCAGAAAATTTCACACCAGCCAATCCCCTATCAACCCCACC
CCACATCAAACCCGAATGATACTTCCTATTCGCCTATGCTATCCTCCGATCTATCCCAAACAAACTAGGCGGAGTACTCG
CCCTAGCTGCCTCTGTACTAGTCCTATTCCTCATCCCCCTTCTCCATACATCTAAACTACGCTCAATAACTTTCCGCCCT
CTATCACAAATCCTATTCTGAACATTAGTAGCCAAC??
??
???????????????????????
Troglodytes
??
??????????????????CTTTGGGTCACTCCTAGGCCTTTGCCTAGTCACCCAAATCGTCACCGGCCTCTTGCTAGCCG
CACATTACACAGCAGATACATCCCTGGCTTTCAACTCCGTAGCTCACATGTGCCGAAACGTCCAATTTGGCTGACTAATC
CGTAACCTCCACGCAAACGGAGCATCCCTCTTCTTCATCTGCATCTACCTCCACATCGGCCGAGGATTCTACTATGGTTC
CTACCTCAACAAGGCAACCTGAAACGTCGGAGTCCTCCTCCTTTTAGCCCTCATAGCAACCGCCTTCGTAGGATATGTCC
TGCCCTGAGGCCAAATATCATTCTGAGGGGCTACAGTCATTACAAATCTATTCTCAGCAATTCCCTACATCGGCCAAACC
TTAGTAGAATGAGCGTGGGGGGGATTCTCAGTAGACAACCCAACCCTTACCCGCTTCTTCGCCCTCCACTTCCTCCTACC

CTTCCTCATCGCAGGACTAACACTCGTCCACCTCACCCTACTCCACGAGACAGGTTCAAACAACCCCCTAGGAATTCCTT
CAGACTGCGACAAAATCCCATTCCACCCATACTACTCAATCAAGGACTTACTGGGATTTGCCCTAATACTAATCTTACTA
GCCACCCTAGCCTTATTCTCACCCAACCTGCTAGGAGACCCAGAAAACTTCACACCAGCCAACCCCCTAGCCACACCCCC
GCACATTAAACCAGAATGATACTTCCTATTCGCATACGCTATCCTGCGATCCATCCCCAACAAACTAGGAGGAGTACTGG
CCCTAGCCGCCTCCGTCCTAGTCCTATTCCTAGTCCCCCTACTCCACACATCCAAACAACGCTCAATAACTTTCCGCCCT
ATCTCTCAAATCCTATTCTGAACCCTAGTAGCCAACCTACTCGTCCTAACCTGAGTCGGCAGCCAACCAGTCGAACACCC
CTTCATCATCATCGGACAACTGGCCTCCCTCTCATACTTCACAATCATTCTAGTTCTATTCCCTCTAGCAGCCATCCTAG
AAAATAAACTACTCAACTTATAA
Luscinia_svecica
??
????????CATGATGAAACTTCGGATCTCTATTAGGCATCTGCCTCCTCACACAAATCGTCACCGGTCTGTTACTAGCCA
CCCACTACACAGCAGACACATCCCTAGCCTTCAACTCAGTCGCCCACATATGCCGAAACGTACAATTCGGCTGACTAATT
CGAAACCTCCATGCAAACGGAGCCTCATTCTTCTTCATCTGCATTTACCTCCACATCGGCCGAGGATTCTACTATGGTTC
CTACCTATACAAAGAAACCTGAAACATCGGAGTAGTTCTCCTCCTAGTACTAATAGCAACCGCTTTCGTAGGCTATGTCC
TACCCTGAGGACAAATATCATTCTGAGGCGCTACAGTAATTACCAACCTATTCTCAGCAATCCCCTACATTGGTCAAACA
CTAGTAGAATGAGCCTGAGGCGGCTTCTCCGTAGACAACCCAACCCTGACCCGATTCTTCGCCCTACACTTCCTACTACC
ATTCCTCATCGTAGGCCTCACACTAGTTCACCTCACATTCCTGCACGAGACAGGATCGAACAACCCACTAGGCATTCCCG
CAGACTGCGACAAAATTCCATTCCACCCATACTACTCCACAAAAGACATCCTAGGCTTTGCACTCATACTCATCCTACTT
GTCTCCCTAGCCCTATTCTCCCCCAACCTACTARGAGACCCAGAAAACTTCACACCAGCTAACCCACTAGCAACACCCCC
CCACATTAAACCCGAATGGTACTTCTTATTTGCATATGCCATTCTACGCTCCATTCCAAACAAACTAGGAGGAGTACTAG
CCCTAATTGCCTCCATCCTAGTCCTATTCCTAACCCCACTACTACATACGTCTAAACAACGCTCACTGACTTTCCGCCCT
ATCTCACAAGTCCTATTCTGAGCCCTAGTCGCCAACCTATTTATCCTAACCTGAGTAGGAAGCCAACCAGTCGAACACCC
ATTCATCATTATCGGCCAACTAGCCTCCCTGTCATACTTCACAATCATTCTAATCCTATTCCCACTTGCGGSCATCCTAG
AGAAC??????????????????
Psittacus_erithacus
??
??TGCCTGGCAACCCAAATCCTAACCGGACTGCTCCTAGCCG
CCCACTACACCGCAGATACCACTCTAGCCTTCTCATCCGTAGCCAACACATGCCGAAACGTACAGTATGGGTGACTAATC
CGCAACCTACATGCAAACGGAGCCTCCCTCTTCTTTATCTGCATCTACCTCCACATTGCCCGAGGCTACTACTATGGTTC
ATACCTCTACAAGGAAACCTGAAACACAGGAATTATCCTCCTACTAACCCTGATAGCAACAGCCTTCGTTGGCTATGTCC
TACCCTGAGGCCAAATATCATTCTGAGGCGCCACAGTCATTACAAACCTATTCTCCGCAATCCCCTACATTGGACAGACC
CTAGTAGAGTGGGCCTGAGGCGGATTCTCAGTAGACAACCCAACCCTAACCCGATTCTTCACCCTACACTTTCTCCTCCC
ATTTATAATCACCAGCTTAGTCCTCATCCACCTAACCTTTCTACATGAGTCGGGATCAAACAACCCCCTAGGCCTCCCAT
CAAACTGCGACAAGATCCCATTCCACCCATACTTCTCCATAAAAGATCTACTAGGATTTATAATCATACTACTCCTACTC
ATATCCCTTGCCCTATTCTCCCCAAACCTACTAGGAGACCCAGAAAACTTCACCCCAGCAAACCCCCTAGTCACCCCCCC
ACATATCAAGCCAGAGTGATACTTCCTATTCGCATATGCCATCCTACGCTCAATCCCCAACAAACTAGGAGGGGTCCTAG
CCCTAACCGCCTCCGTACTTATCCTATTTCTAACCCCCCTCCTTCATAAATCCAAACAACGCACCATAGCTTTTCGCCCC
ATATCACAACTCCTATTCTGGGTACTAGCAGCCAACCTATTTGTCCTAACCTGGGTAGGAAGCCAACCCGTAGAACACCC
CTTCATCATCATCGGGCAGCTAGCCTCAATCACCTACTTTACCATTATCCTCATTCTATTCCCCATTACCTCCTCCCTAG
AAAACAAAATCCTTAAATAA???
Ficedula_hypoleuca
ATGGCCCTCAATCTTCGTAAAAACCACCCGCTATTCAAAACCATTAACGATGCCCTTATTGACCTCCCCACACCATCAAA
CATCTCAGCTTGATGAAACTTCGGGTCACTACTAGGCATCTGCCTGATCACACAAATCATCACCGGGCTGCTACTAGCCA
CTCACTACACAGCAGACACCTCCCTAGCCTTTAATTCAGTCGCCCACATATGCCGAAACGTACAGTTCGGCTGACTAATC
CGGAACCTCCACGCAAACGGAGCCTCCTTCTTCTTCATCTGCATTTACCTACACATCGGCCGAGGGTTCTACTACGGCTC
CTACCTGAACAAAGAAACCTGAAACGTCGGAGTTATCTTACTCCTCATCCTAATAGCAACCGCCTTCGTGGGATACGTCC
TCCCCTGAGGACAAATATCATTCTGAGGGGCTACAGTAATTACCAACCTATTCTCAGCAATCCCGTACATCGGCCAAACA
CTAGTAGAATGAGCCTGAGGTGGCTTCTCAGTAGACAACCCCACACTCACCCGATTCTTTGCCCTCCACTTCCTACTCCC
CTTCGTCATCGTAGGCATTACACTAGTCCACCTCACATTCCTACACGAAACAGGCTCAAACAATCCACTAGGAATCCCCG
CAGACTGCGACAAAATTCCATTCCACCCATACTACTCTACAAAAGACATTCTAGGGTTCGCACTCATGCTCATCCTCCTC
GTAGCCCTAGCCCTATTCTCCCCTAATCTCCTAGGAGACCCAGAAAACTTTACACCAGCCAACCCACTAGCTACGCCCCC
GCACATCAAACCCGAATGATACTTCCTATTCGCATATGCCATCCTACGTTCCATCCCAAATAAACTAGGAGGCGTACTAG
CACTAGCTGCATCCGTCCTAGTCCTATTCTTAGTACCACTACTCCACACATCTAAACAACGCTCACTAACCTTCCGACCC
ATCTCACAAATCCTGTTCTGAGCCCTAGTAGCCAACCTACTCGTCCTAACCTGAGTAGGGAGCCAACCAGTCGAACACCC
ATTCATCATCATCGGCCAATTAGCCTCCCTCTCCTACTTTACAATCATTCTCATCCTCTTCCCACTTGCAGCTGTACTAG
AGAACAAAATACTAAATCTCTAA
Menura_novaehollandiae
ATGGCCCTCAATCTTCGCAAACACCACCCTCTGATAAAAATCGTCAACGACTCTCTAATCGACCTTCCCACTCCATCAAA
CATCTCAATTTGATGAAACTTCGGATCCCTACTAGGAATCTGCCTCATTACTCAGATCGTCACTGGGTTATTACTAGCAA
TACACTACACTGCAGATACCTCCCTAGCCTTTGCCTCAGTAGCCCACACATGCCGTAACGTACAATTTGGATGACTAATC

CGAAATCTACACGCAAACGGAGCCTCACTATTCTTCATCTGCATCTACCTTCATATCGGCCGAGGAATCTACTACGGCTC
ATACCTATACAAAGAGACCTGAAACATTGGGGTAATCCTACTCCTAACACTAATAGCAACTGCCTTCGTAGGCTACGTCC
TCCCATGAGGACAAATGTCCTTCTGAGGGGCTACCGTAATTACTAACCTATTCTCAGCCATCCCCTACATCGGCCAAACA
CTAGTAGAATGAGCCTGAGGAGGATTCTCAGTAGACAACCCCACACTAACTCGATTCTTCACTCTTCACTTCCTCCTACC
TTTCGTAATCGTCGCTCTAACCTTAGTCCACCTGACCTTCCTTCACGAATCAGGCTCAAATAACCCGCTAGGCATCCCCT
CAGACTGTGACAAGATCCCGTTCCACCCCTACTACTCCACAAAAGACGTCCTAGGCTTTGCCCTAATACTACTACTCCTC
GCTACCCTAGCTCTATTCTCCCCAAACCTCCTAGGAGATCCAGAAAACTTCACCCCAGCGAACCCTCTAGTCACCCCACC
ACATATCAAACCTGAGTGATACTTCCTATTCGCATACGCTATCCTACGATCTATCCCCAACAAACTAGGAGGAGTACTAG
CCCTTGCTGCCTCCGTCCTAGTCCTATTCCTAATGCCATTCCTACATAAGTCAAAACAACGCTCAATGACTTTCCGCCCC
CTATCCCAAATCCTGTTCTGAACCCTAGTAGCCAACATCCTCATTCTAACATGAGTAGGAAGCCAACCAGTGGAACATCC
ATTCATCATCATTGGTCAATTAGCCTCATTCACCTACTTCGCCATCATTCTAATCCTACTACCCCTTGCAGGGGCCTTAG
AAAACAAACTACTCAAGCTCTAA
Pycnonotus
??GGCCCCCAATCTTCGTAAAAACCACCCACTACTAAAAACCATCAACGATTCCCTAATCGACCTTCCCACCCCATCAAA
CATCTCAATTTGATGAAACTTCGGATCCCTACTAGGCATCTGCCTCATTACACAAATCATCACAGGACTGCTACTAGCTA
TACACTATACAGCGGACACAGCCCTGGCATTCAACTCTGTAGCTCACATTTGCCGAAACGTCCAATTTGGCTGACTAATC
CGTAACCTACACGCAAACGGAGCCTCCCTATTCTTCATATGCATCTACTTACACATTGGCCGAGGAATTTACTACGGTTC
ATACCTAAACAAAGAAACCTGAAACGTAGGGGTAGTCCTACTCCTGGCCCTAATAGCAACTGCTTTCGTCGGCTACGTGC
TCCCCTGAGGACAGATATCATTCTGAGGGGCTACCGTAATCACAAACCTATTCTCGGCCATCCCATATATCGGCCAAACA
CTGGTAGAATGAGCATGAGGCGGATTCTCTGTAGACAACCCTACACTAACCCGATTCTTTGCTCTTCACTTCCTACTACC
ATTCCTCATTGCAGGACTCACACTAGTCCACCTCACCCTGCTACATGAAACAGGATCTAACAACCCCCTAGGAATTCCCT
CAGACTGTGATAAAATCCCATTCCACCCTTATTACTCCACAAAAGACATTCTAGGATTCATGCTAGTATTCATCCCCCTA
GCCGCCCTAGCCCTGTTTTCCCCCAACCTGCTAGGAGACCCAGAAAATTTCACACCAGCCAACCCCCTAGCCACACCCCC
ACATATCAAACCCGAATGATACTTCCTATTCGCCTACGCTATCCTCCGATCCATCCCAAACAAACTTGGAGGAGTACTAG
CACTTGCTGCTTCCGTCCTAGTTCTATTCCTAATCCCCCTACTACACACGTCCAAACTACGCTCAATAACATTCCGCCCC
CTATCACAAATCCTATTCTGAACACTAGTCGCTAACCTCCTAATCCTAACCTGAGTAGGAAGCCAACCAGTTGAACACCC
ATTCATCATCATCGGACAAATCGCTTCATTCTCCTACTTCACAATCATCCTAGTCCTATTCCCCATCGCATCCATCGTAG
AGAACAAAATACTCAAACTCTAA
Donacobius_atricapilla
??
?????????????????CTTCGGATCACTACTAGGCATTTGTCTAATCACACAAATCGTCACAGGCCTACTTCTAGCCA
TACACTACACAGCAGACACTTCCCTAGCCTTCGCTTCAGTAGCACATACATGCCGAAATGTCCAATTCGGCTGACTAATT
CGCAACCTACACGCAAACGGAGCCTCCTTCTTCTTCATTTGCATCTACTTCCACATCGGCCGAGGGTTCTACTACGGCTC
GTACCTAAACAAAGAAACCTGAAACGTTGGCGTAATTCTTCTTCTACTACTAATAGCAACCGCCTTCGTAGGATATGTCC
TACCATGAGGACAAATATCATTCTGAGGGGCTACAGTAATTACAAACCTATTCTCAGCAATCCCATACATCGGACAGACC
CTAGTGGAATGAGCCTGAGGAGGGTTCTCAGTAGACAACCCAACACTCACCCGATTCTTCGCCCTCCACTTCCTACTCCC
ATTCGTCATCGCAGGAGTAACGCTAGTCCACCTTACCCTGCTTCACGAAACAGGATCGAACAACCCCCTAGGAATCCCCT
CAGATTGCGACAAAATTCCATTCCACCCATACTACTCCGTGAAAGACATCCTCGGATTTGTACTTATGCTAGCCCTACTA
GCCTCCATAGCCCTATTTTCCCCAAACCTCCTCGGAGATCCAGAAAATTTCACTCCCGCCAACCCACTGGCTACACCACC
CCACATCAAACCCGAATGATACTTCCTATTCGCCTACGCAATCCTACGATCCATCCCAAACAAACTAGGAGGAGTCTTAG
CCCTGGCAGCCTCTGTACTAGTCCTATTCCTAATACCACTCCTACACACCTCCAAACTACGCTCAATAACATTCCGTCCA
CTGTCACAAATTCTCTTCTGAACACTAGTAGCAAACCTATTAGTCCTAACCTGAGTAGGGAGCCAACCAGTAGAACACCC
ATTCATCATCATTGGTCAACTAGCCTCATTATCCTACTTCACCATCATCCTAATTCTTTTCCCCCTCGTATCAATCTTAG
AGAACAAAATACTTAAAATCTAA
Hirundo_rustica
ATGGCCCCCAACCTTCGTAAAAACCACCCGCTACTGAAAATCATCAACGACTCCTTAATCGACCTGCCTACCCCATCAAA
CATCTCAACCTGATGAAACTTCGGCTCATTACTAGGACTATGCCTAGTCATACAAATCGTCACAGGCCTACTTCTAGCTA
CCCACTATACAGCAGATACCTCACTAGCCTTCGCCTCTGTAGCCCATATATGCCGAGACGTACAGTTTGGCTGACTTATC
CGAAACCTCCATGCAAACGGAGCCTCCTTCTTCTTCATCTGTATCTACCTACACATCGGACGAGGATTCTACTACGGATC
CTACCTAAACAAAGAAACTTGAAACGTCGGAGTAGTACTGCTACTAGCACTAATAGCCACGGCCTTCGTAGGCTACGTCC
TGCCCTGAGGACAAATATCATTCTGAGGGGCTACAGTAATCACGAACCTATTCTCAGCAATTCCGTACATCGGCCAAACA
CTTGTAGAATGAGCATGAGGAGGGTTCTCAGTAGACAACCCTACCCTAACCCGATTCTTCGCCCTACACTTCCTCCTCCC
ATTCGTCATCGCAGGACTGACCCTAGTACACTTAACCCTACTCCACGAAACAGGATCAAACAACCCACTAGGAATCCCCT
CAGACTGCGATAAAATCCCATTCCACCCATACTACTCCACAAAAGACATCCTAGGATTTATCATACTACTCATCGTACTA
GCTTCCCTAGCACTATTCTCCCCAAACCTCTTAGGTGACCCGGAAAACTTCACACCAGCCAACCCCCTGGCTACTCCACC
GCACATCAAACCCGAATGATACTTCCTATTCGCCTACGCCATCCTCCGATCCATCCCAAACAAACTAGGAGGAGTACTAG
CCCTAGCTGCCTCCGTCCTAGTATTATTCCTAATACCTCTACTCCACACCTCCAAACTGCGATCAATAACATTCCGACCA
CTATCACAAATCCTATTCTGGACCCTAGTCGCTAACTTACTTGTCCTAACCTGAGTAGGAAGCCAACCAGTAGAACACCC
CTTCATCATCATTGGACAACTAGCCTCGCTGTCCTACTTCACCATCATCCTGGTCCTATTTCCACTTGTCTCCATCCTAG
AAAACAAAATACTCAAACTCTAG

Lichenostomus
??
???????????????????TTCGGATCACTACTAGGCATCTGCCTAACAACCCAAATTGTCACAGGACTACTACTAGCCA
CGCACTATACAGCGGACACCTCCCTAGCTTTTTCATCTGTCGCCCACATATGCCGAGACGTCCAATTCGGCTGACTAATT
CGAAACCTCCATGCAAACGGAGCTTCCTTCTTCTTCATCTGCATCTACATCCACATCGGTCGAGGACTATACTACGGCTC
CTACCTAAACAAAGAAACCTGGAACATCGGAGTCGTGCTCCTCCTAACCCTAATAGCAACCGCTTTCGTAGGCTACGTCC
TGCCCTGAGGACAAATATCCTTCTGAGGGGCTACAGTAATTACCAACCTATTCTCAGCCATCCCTTACATCGGACAAACA
CTAGTAGAATGAGCCTGAGGAGGATTCTCAGTAGACAATCCCACCCTAACTCGATTCTTCGCCCTTCACTTCCTCCTTCC
CTTTGCCATTGCAGGCTTAACCCTCGTCCACCTGACATTCCTGCACGAAACCGGATCTAACAACCCCCTAGGAATCCCAT
CAGACTGCGACAAAATTCCATTCCATCCCTACTACTCAATCAAAGACATCCTAGGATTCGCAATAATGATCATTCTGCTA
GCCTCCCTAGCCCTATTTTCCCCTAACCTCCTAGGCGACCCGGAAAACTTCACGCCAGCCAACCCACTAGCCACTCCCCC
TCACATTAAACCCGAATGATACTTCCTATTTGCATACGCCATCCTCCGATCTATCCCCAATAAACTAGGAGGAGTCCTAG
CCCTAGCCGCCTCAATCCTCGTGCTTTTCCTAATCCCCCTACTACACAAATCCAAACAACGTTCCATAACATTCCGCCCT
CTGTCCCAAATCCTATTCTGAACCCTAGTCGCCAACCTCCTAGTCCTAACATGAGTAGGAAGCCAACCAGTTGAACACCC
ATTCATCATCATCGGCCAACTAGCATCCCTCAGCTACTTCACCATCATCCTAATTCTCTTCCCCCTGGCTAGCATGCTAG
AGAACAAAATACTAAAACTTTAA
Cnemophilus
ATGGCCCTCAATCTTCGTAAAAACCACCCACTGCTAAAAATAGTCAACGACTCCCTAATCGACCTTCCAACCCCCTCCAA
CATCTCAGGATGATGAAATTTCGGATCCCTTCTAGGAATCTGCCTAATCACACAAATTGTCACAGGATTACTCCTAGCCA
CACATTACACAGCAGACACCTCCTTAGCTTTCTCCTCCGTAGCACATATGTGCCGAAATGTCCAATTCGGCTGACTAATC
CGAAACCTACACGCAAACGGAGCCTCATTCTTCTTCATCTGCATCTACCTACACATCGGCCGAGGTTTCTACTACGGCTC
TTACTTAAATAAAGAAACCTGAAATGTAGGAGTTATCCTCCTACTAATCCTAATAGCAACCGCCTTCGTAGGTTACGTAC
TACCCTGAGGACAAATATCCTTCTGAGGGGCTACAGTAATCACTAACCTATTTTCAGCAATTCCATACATTGGTCAAACA
CTAGTAGAATGAGCCTGAGGAGGGTTCTCAGTAGACAATCCTACACTAACTCGATTCTTCGCCCTACACTTCCTCCTTCC
CTTTGTAATCGCAGGACTAACACTAGTCCATCTCACCTTCCTACACGAAACAGGATCTAACAATCCCCTAGGAATCCCCT
CAGACTGTGACAAAATCCCATTCCACCCATACTACTCCACAAAAGACATTCTAGGATTCGCACTACTATTCATCCCACTA
ATAGCCCTCACCCTATTCTCCCCTAACCTTCTAGGAGACCCAGAAAACTTCACGCCAGCCAACCCTCTAGCCACACCCCC
TCATATCAAACCAGAATGATACTTCCTATTCGCATACGCCATTCTCCGATCCATCCCCAACAAACTAGGAGGAGTTTTAG
CCCTAGCCGCCTCAGTGCTAGTCCTATTCCTCCTACCCCTCCTCCACGTATCCAAACAACGATCCATAACCTTCCGTCCA
CTCTCACAAATCCTGTTCTGAACCCTAGTTGCCAACCTCCTAATCCTAACATGAATTGGCAGCCAACCAGTAGAACACCC
ATTCATTATCATCGGACAGCTAGCCTCACTCTCCTACTTCACAATCATCCTGATCCTATTCCCCCTAGCAGCTGCCCTAG
AAAACAAGCTACTAAACCTATAA
Coracina
ATGGCCCTCAATCTACGTAAAAACCACCCCCTACTCAAAACTATTAACGATTCCCTCATCGACCTCCCTACTCCATCCAA
CATCTCAACCTGATGAAACTTCGGATCACTACTAGGCATTTGCCTCATCACTCAGATCATCACAGGCCTGCTACTAGCCA
CACACTACACAGCAGACACCACACTGGCCTTCAATTCCGTGGCCCACACCTGCCGAAACGTACAGTTCGGATGATTAATC
CGAAACCTCCACGCAAACGGAGCTTCTATGTTCTTCATCTGCATCTACCTCCACATCGGCCGAGGAATTTACTACGGCTC
CTACCTAAACAAAGAAACCTGAAATGTCGGTGTTATCCTACTACTCACTCTAATAGCAACTGCTTTCGTAGGCTACGTCC
TGCCTTGAGGACAAATATCCTTCTGAGGAGCGACAGTAATTACTAACCTACTCTCAGCAATCCCCTACATTGGTCCAACA
CTAGTCGAATGAGCGTGAGGAGGGTTTTCTGTAGACAACCCTACACTGACTCGATTCTTCGCCCTTCACTTCCTGCTCCC
ATTCGTAATCGTAGGATTAACACTAGTCCACTTAACCTTCCTACACGAGACAGGATCAAACAATCCACTTGGAATCTCAT
CCGACTGCGATAAAATCCCATTCCACCCTTACTATTCTATCAAAGACATTCTAGGATTCGCCCTAATACTTATCCTACTC
GTAGCTATAGCCCTATTTTCCCCAAACCTGCTAGGAGACCCAGAGAACTTTACACCAGCTAATCCCCTAGCCACACCTCC
TCATATCAAACCTGAATGATACTTCCTATTTGCATACGCCATCCTCCGATCCATCCCCAACAAACTGGGAGGAGTCCTAG
CTCTCGCTGCTTCTGTCCTAGTCCTATTCCTCATTCCACTACTGCACAAATCCAAACAACGATCCATAACATTCCGACCT
CTCTCACAAATCCTATTCTGAGCCCTAGTTGCTAACCTACTTATCCTCACATGAATCGGTAGCCAACCAGTCGAACATCC
ATTCATCATTATTGGACAATTAGCCTCATTTACTTACTTCACCATCATCCTAGTCCTATTTCCTCTTGCAAGCGTACTAG
AAAACAAACTACTAAATCCCTAA
Dicrurus
??
??GGACTACTACTAGCCA
TACATTACACAGCAGATACTAGCCTAGCTTTCACCTCTGTAGCACATACATGCCGAAACGTCCAATTTGGATGACTAATT
CGTAATTTACATGCAAACGGAGCTTCCTTTTTCTTCATTTGCATTTACCTACATATTGGCCGAGGATTCTACTACGGCTC
ATACCTAAACAAAGAAACCTGAAACATTGGAGTTATCCTACTCCTTACCTTAATAGCAACAGCCTTCGTCGGATATGTAC
TTCCCTGAGGACAAATATCATTCTGAGGCGCTACAGTAATCACTAATCTATTCTCAGCAATCCCTTATATTGGCCAAACA
CTAGTAGAATGAGCTTGAGGAGGTTTCTCAGTAGATAACCCCACACTAACTCGATTCTTCGCCCTTCACTTCTTACTACC
ATTTATCATTGCAGGCCTAACACTGGTACATCTAACATTCCTACACGAAACAGGCTCAAACAATCCACTTGGTATTTCTT
CGGATTGTGACAAAATCCCATTCCACCCATACTACTCAATCAAAGATATTCTAGGACTAGCACTAATACTAATCCCATTC
ATTGCCCTAGCATTATTTAATCCAAATCTCCTAGGAGATCCAGAAAATTTCACGCCCGCCAACCCCCTAGTCACCCCACC
CCATATTAAACCTGAATGATACTTCCTATTCGCATACGCCATCCTCCGATCAATTCCAAATAAACTAGGAGGAGTCCTAG

CCCTAGCCGCCTCAGTACTAATCCTATTCCTAATACCCCTACTACATACCTCTAAACAACGATCAATAACCTTCCGACCC
CTATCACAAGTCCTATTCTGAATCCTAGTCASTAACCTGCTTATCCTGACATGAGTTGGAAGCCAACCAGTCGAACACCC
ATTCATCATTATCGGCCAAGTAGCCTCATTCACCTATTTCATGATCATTCTAGTGCTATTCCCAGCTGCAAGCATCCTAG
AAAATAAAATACTGAAACTATAA
Gerygone_fusca
??
??GGTCTCCTACTAGCTA
TACACTACACAGCAGACACCACCCTAGCCTTCTCCTCCGTCGCCCACACATGCCGAAATGTCCAATTTGGCTGACTAATC
CGCAACCTCCATGCGAACGGAGCCTCATTCTTCTTCATCTGCATCTACTTTCACATCGGCCGAGGTTTTTATTACGGTTC
TTACCTAAATAAAGAAACCTGAAACATCGGAGTTGTACTCCTCCTAGCCCTAATAGCAACTGCTTTCGTGGGTTACGTCC
TCCCCTGAGGACAAATGTCATTCTGAGGGGCCACAGTAATTACTAACCTATTCTCGGCAATCCCCTACATCGGTCAAACT
CTAGTAGAATGAGCCTGAGGCGGATTCTCAGTAGATAACCCCACATTAACCCGATTCTTTGCCCTCCACTTCCTCCTCCC
ATTCGTGATCGCGGGCCTAACACTGGTTCACCTCACATTCCTTCACGAAACAGGCTCAAACAACCCCTTAGGAATCCCTT
CAGACTGCGATAAAATCCCATTCCACCCCTACTACTCTACAAAAGACATCCTAGGCTTCGCACTAATACTCTCCTCACTT
ACCGCCCTCGCTCTATTCTCACCCAACCTCCTAGGAGACCCAGAAAACTTTACCCCAGCAAACCCCCTAGCTACTCCCCC
ACACATCAAACCCGAATGATACTTCCTATTCGCCTACGCTATTCTCCGATCCATCCCTAACAAACTAGGAGGAGTCCTAG
CCCTAGCCGCTTCAATCCTCATTCTATTCCTCGTCCCCCTACTCCACAAATCCAAACAACGCTCAATAACCTTCCGTCCC
CTCTCTCAAATCCTATTCTGAACTCTAGTCGCTAACCTCCTAATTCTAACATGAGTCGGCAGCCAACCAGTTGAACACCC
ATTTATCATCATCGGCCAATTAGCCTCTATCTCCTACTTCACTATTATCCTTGTCCTATTCCCCCTAGTGAGCATCCTAG
AAAATAAACTACTAAAACTCTAA
Ptiloris_magnificus
??
??GGCCTACTGCTAGCAG
CACATTACACAGCAGACACCTCCCTAGCCTTCAGCTCCGTAGCTCACATATGCCGAGACGTCCAATTCGGATGACTAATC
CGAAACCTGCATGCAAACGGAGCCTCCATATTCTTCGTTTGCATCTACCTACATATCGGCCGAGGGTTCTACTACGGCTC
ATACCTCAACAAAGAAACCTGAAACGTTGGAGTAATCCTACTCCTAACCCTAATAGCAACAGCTTTCGTCGGATACGTCC
TTCCTTGAGGACAAATATCCTTCTGAGGTGCTACAGTCATTACTAACCTACTCTCAGCAATTCCATACATCGGGCAAACC
CTAGTAGAATGAGCTTGAGGAGGATTTTCAGTAGACAACCCTACACTAACCCGATTCTTTGCCCTCCACTTCCTCCTTCC
ATTCGTAATTGTAGGCCTGACACTAGTTCACCTGACATTCCTACACGAAACAGGATCAAACAACCCTCTCGGAATCCCAT
CAGATTGCGACAAAATTCCATTCCACCCCTACTACTCCATCAAAGACATCCTAGGATTCGCACTAATACTAACCCTGCTA
GCCGCCTTAGCACTATTCTCCCCAAACCTATTAGGAGACCCAGAAAACTTCACACCAGCCAATCCCCTAACCACACCCCC
TCATATCAAACCAGAATGATATTTCCTATTTGCATACGCTATCCTCCGATCCATCCCCAACAAACTAGGAGGAGTCCTAG
CTCTAGCTGCTTCAGTTTTAGTCCTATTCCTCATTCCTCTGCTTCACACATCCAAACAACGATCAATAACTTTCCGACCC
CTATCACAAATCCTATTCTGAATTCTAGTAACTAACCTACTAATTCTAACATGAGTAGGCAGC?????????????????
??
???????????????????????
Paradisaea_raggiana
ATGGCTCTCAATCTACGCAAAAACCACCCTCTACTAAAAATCATCAACGACTCTTTAATTGACCTTCCCACTCCATCAAA
CATCTCAATTTGATGAAACTTCGGATCTCTTCTAGGAATCTGCCTAGTAACACAAATTATCACAGGCCTGCTGCTAGCAG
CACATTACACAGCAGACACCTCCCTAGCTTTCAACTCTGTAGCCCACATGTGCCGAAATGTCCAATTTGGATGACTAATT
CGAAACCTACATGCAAACGGAGCTTCCTTATTCTTTATTTGCATCTACCTACACATCGGCCGAGGATTTTATTACGGCTC
ATACCTCAACAAAGAAACCTGAAACATCGGAGTAATCCTACTCCTAACCCTAATAGCAACAGCCTTCGTCGGATACGTCC
TCCCTTGAGGACAAATGTCCTTTTGAGGGGCTACAGTTATCACTAACCTATTCTCAGCAATTCCATACATTGGGCAAACC
CTAGTAGAATGAGCCTGAGGAGGTTTTTCAGTAGATAATCCCACACTAACCCGATTCTTCGCCCTACACTTCCTCCTCCC
ATTCGTAATCGCAGGCTTAACACTAGTTCACCTAACATTCCTACACGAAACAGGATCAAACAACCCCCTCGGAATCCCAT
CAGACTGCGACAAAATCCCATTCCACCCGTACTACTCCATCAAGGACATCCTAGGATTCGCACTAATGCTAATTTCACTC
GCCACTCTGGCACTATTCTCCCCAAACCTCCTAGGAGACCCAGAAAACTTTACTCCAGCCAACCCCCTGGCCACACCTCC
ACATATCAAACCAGAATGATATTTCCTATTTGCATACGCCATCCTTCGATCAATCCCTAACAAACTAGGAGGAGTCCTAG
CTCTAGCCGCCTCCGTATTAATTCTATTCCTTATCCCTCTACTCCACACATCAAAACAACGATCAATAACCTTCCGACCC
CTATCACAAATCCTATTCTGAATCCTAGTAACCGACCTATTAATCCTAACATGAGTAGGCAGCCAGCCAGTTGAACATCC
ATTCATTATTATTGGTCAACTAGCCTCATTCCTCTACTTCATAATCATCCTAGTCCTATTCCCCATCGTAGGTGCACTAG
AAAACAAACTACTCAATCTCTAA
Cyanocorax_chrysops
ATGGCCCTAAATCTACGTAAAAACCACCCTCTAATAAAAATCATCAACGATTCTCTAATCGATCTTCCTACTCCATCAAA
CATCTCAGCTTGATGATATTTCGGATACTTTCTAGGCATCTGCCTAATCACACAAATCATTACAGGCCTACTGCTAGCTA
TACACTACACAGCAGATACTTCCTATGCTTTTACATCCGTAGCCCACATGTGCCGAAACGTCCAATTTGGATGACTAATC
CGAAACCTCCATGCAAATGGAGCCTCCCTATTCTTCGTGTGTATCTACCTACACATCGGCCGAGGACTATACTATGGTTC
ATACCTAAACAAAGAAACCTGAAACATCGGAGTAATTCTCCTACTAACTCTTATAGCAACAGCCTTTGTTGGATATGTCC
TGCCTTGAGGACAAATATCCTTCTGAGGTGCCACAGTCATCACCAACCTTTTCTCAGCAATCCCATACATTGGACAAACA
CTAGTAGAATGACTCTGAGGAGGGTTTTCAGTAGATAACCCCACCCTCACCCGATTCTTTGCCTTCCACTTCCTACTCCC

ATTTGTTATCGTAGGAATAACATTAGTCCACCTAACCTTCCTACACGAAACTGGATCAAACAACCCCCTAGGAATTCCGT
CCGACTGTGACAAAATCCCATTCCACCCATACTACTCCATCAAAGACCTTCTAGGATTTGCACTAATACTAATCCTACTT
GTTACTCTAGCACTATTTAACCCAAACCTCCTAGGAGATCCAGAAAATTTCACGCCCGCCAACCCTCTGGCTACTCCCCC
ACACATCAAACCAGAATGATACTTCCTATTTGCATATGCCATTCTTCGATCAATTCCAAACAAACTAGGAGGAGTATTAG
CCCTAGCCGCCTCAGTCCTAGTTTTATTCCTCATTCCCCTATTACACGTCTCCAAACAACGTTCAATAACCTTCCGTCCC
CTATCCCAAATTCTATTCTGAACCCTAGTTGCAGACCTACTAATCCTAACATGAGTCGGGAGCCAGCCAGTCGAACACCC
ATTTATCATCATCGGCCAATTAGCTTCCTTAGCCTACTTTACAATCATTCTAATCCTATTCCCAATTGCGAGCGCACTAG
AAAACAAAATGCTCAACCTCTAA
Lonchura
??
???????????????????TTCGGGTCACTCCTAGGCATTTGCTTAATTACCCAAATTGTCACAGGCCTGCTGCTAGCTA
TGCACTACACAGCAGACACCTCCCTAGCCTTCTCCTCAGTAGCCCACATGTGCCGAGATGTACAATTTGGCTGACTAATC
CGCAACCTCCACGCTAACGGCGCCTCCTTCTTCTTCATCTGCATTTACCTCCACATCGGCCGAGGAATCTACTACGGCTC
ATACCTGTACAAAGAAACTTGAAACGTAGGAGTCATCCTCCTCCTAACCCTCATGGCTACCGCTTTCGTAGGGTACGTCC
TGCCCTGAGGACAAATATCATTCTGAGGAGCTACAGTAATCACAAACCTACTCTCAGCAATCCCCTATATCGGCCAAACG
CTAGTAGAATGAGCCTGAGGGGGATTCTCAGTAGATAACCCAACACTAACACGATTTTTCGCCCTCCATTTCCTACTCCC
ATTCGTCATCGCAGGACTAACACTAGTGCACCTCACCTTTCTACATGAAACAGGCTCAAACAACCCACTAGGTATCCCAT
CAGACTGCGACAAAATCCCCTTCCATCCATACTACACTACAAAAGACGTACTAGGATTCGCACTAATACTCTCCCTACTA
GCCTCACTAGCCCTATTTTCCCCAAACTCACTAGGAGACCCAGAAAACTTCACACCCGCTAATCCCCTAGTAACACCTCC
CCACATTAAACCCGAATGATACTTCCTATTCGCCTACGCTATCCTACGATCCATCCCAAACAAACTAGGAGGAGTCCTAG
CCCTACTCGCTTCAATCCTAGTCCTATTCATACTCCCCCTACTACATACATCCAAACTACGATCAATAACCTTCCGACCC
CTATCCCAAATCCTATTCTGAACCCTCGTCGCCAATGTCCTAGTACTAACCTGAGTGGGCAGC?????????????????
??
???????????????????????
Phylloscopus
??
???????????????????TTCGGATCCCTCCTAGGCATCTGCTTAATCACACAAATTGTCACAGGACTACTACTAGCCA
CACACTACACAGCAGATACATCCCTAGCATTCACTTCTGTCGCCCATATATGCCGAGACGTCCAATTCGGCTGACTAATC
CGCAACCTCCACGCAAATGGAGCTTCCTTTTTCTTTATTTGCATCTACTTCCACATCGGCCGAGGGTTCTACTACGGATC
ATACCTAAACAAAGAAACCTGGAACATTGGAGTCATTCTCCTACTAACACTTATAGCCACTGCCTTTGTCGGATACGTCC
TACCTTGAGGGCAAATATCATTCTGAGGGGCTACAGTAATTACAAACCTGTTCTCAGCAATCCCATACATTGGTCAAACA
CTAGTAGAATGAGCGTGGGGTGGATTCTCAGTAGACAACCCTACCCTAACTCGCTTCTTTGCCCTTCACTTCCTTCTCCC
ATTCGTCATCGCAGGACTTACCCTAGTCCACTTAACCCTACTACACGAAACAGGATCAAACAACCCACTAGGAATTCCAT
CAGACTGCGACAAAATCCCATTCCACCCCTACTACTCCACAAAAGACATCCTAGGCTTCGCACTCATACTAATCCTCCTT
GCTTCCCTAGCCCTATTCTCACCCAACCTACTAGGAGACCCAGAAAACTTCACACCAGCCAACCCTCTAGCCACACCCCC
ACATATCAAACCTGAATGATACTTCTTATTTGCTTACGCCATCTTACGATCAATCCCTAACAAACTGGGAGGCGTACTAG
CCCTGGCTGCTTCCGTATTAGTCCTCTTCCTCCTTCCTCTTCTCCACACATCCAAACTTCGCTCAATAACTTTCCGCCCC
CTATCACAAATTCTATTCTGAACTTTAGTAGCCAACCTCCTCATCCTAACCTGAGTAGGAAGCCAGCCAGTTGAACACCC
ATTTATTATTATTGGTCAACTAGCTTCCTTCACATACTTCACCATCATTCTAGTTCTCTTCCCTCTTGTATCCATCCTAG
AAAATAAAATACTCAAACTC???
Turdus
??
??????????????????????GGGTCACTACTAGGCATTTGCTTAATTACACAAATTATCACAGGCTTACTACTAGCCA
CGCATTACACAGCAGACACCTCCCTAGCCTTCAACTCAGTCGCCCACATATGCCGAAATGTCCAATTTGGCTGACTAATC
CGCAACCTCCATGCAAACGGAGCCTCAATATTCTTCATCTGCATTTACCTCCACATTGGCCGAGGGTTTTACTACGGCTC
GTATCTAAACAAAGAAACCTGAAACATCGGAGTTATTCTACTCCTAACCCTAATAGCAACTGCCTTCGTAGGCTACGTAC
TTCCCTGAGGACAAATGTCATTCTGAGGGGCTACAGTAATCACCAACCTATTCTCAGCAATCCCCTACATCGGCCAAACA
CTAGTAGAATGAGCCTGAGGGGGATTCTCAGTAGACAACCCCACACTGACGCGATTCTTCGCCCTCCACTTCCTCCTCCC
ATTCGTCATTGCAGGGCTCACACTAGTACATCTCACCTTCCTGCACGAAACAGGGTCTAACAACCCACTAGGAATCCCCG
CAGACTGCGATAAAATCCCCTTCCACCCTTACTACTCCACAAAAGACATCCTAGGATTTGCACTAATGCTCATCCTACTA
GTCTCCCTAGCCCTATTTTCCCCCAATGCACTCGGGGACCCAGAAAACTTCACGCCAGCCAACCCACTAGTCACACCCCC
TCACATCAAGCCTGAATGATACTTCCTATTCGCATACGCCATCCTTCGATCCATCCCAAACAAACTAGGAGGAGTATTAG
CACTAGCAGCCTCCGTCCTAGTCCTATTTCTCACCCCACTCCTACATAAATCGAAACAACGTTCAATAACCTTCCGACCC
CTGTCACAAATCCTATTCTGGGCCCTAGTAGCCAACCTCCTAATCCTTACTTGAGTAGGAAGCCAACCAGTTGAACACCC
GTTCATTATCATCGGCCAACTGGCCTCACTCTCCTACTTCACAATCATTCTAGTCCTATTTCCCCTCGCAGCCGTACTAG
AAAACAAAATGCTAAAACTC???
Acrocephalus
ATGGCCCTCAATCTTCGTAAAAACCACCCGCTAATAAAAATCGTCAACGACTCTCTAATCGACCTCCCCACCCCATCAAA
CATCTCAACTTGATGAAATTTTGGCTCACTACTAGGTATTTGCCTGATCACCCAAATCGTCACAGGACTTCTGCTAGCCA
TGCACTACACAGCAGACACCTCCCTAGCATTTGCCTCCGTCGCCCACGTATGCCGAGACGTACAATTTGGATGACTGATC

CGCAACCTCCACGCAAACGGAGCCTCTTTCTTCTTCATTTGCATTTACTTCCACATCGGCCGAGGGTTTTACTACGGATC
ATACTTGAACAAAGAAACCTGAAACATCGGCGTCATCCTCCTACTAACCCTCATAGCAACTGCCTTCGTAGGATACGTCC
TACCCTGAGGGCAGATATCATTCTGAGGAGCAACAGTAATCACAAACCTCTTCTCCGCCATCCCATACATCGGCCAAACA
CTAGTAGAATGAGCATGAGGAGGCTTTTCAGTAGACAACCCAACCCTAACCCGATTCTTTGCCATTCACTTCCTACTACC
GTTCATTATCGCAGGCCTGACCCTAGTACACCTAACCCTACTACACGAAACAGGGTCAAACAACCCACTAGGAATCCCAT
CAGACTGCGACAAAATTCCATTCCACCCCTACTACTCCGTAAAAGACATTCTAGGGTTTGCACTCATATTCATCCTTCTT
GCCTCCCTAGCCCTATTCTCACCCAACCTGCTAGGAGACCCAGAAAACTTCACGCCCGCCAACCCCCTAGCCACTCCCCC
GCACATCAAACCCGAATGATACTTCCTATTTGCATATGCTATCCTACGATCCATCCCAAACAAACTAGGGGGAGTACTAG
CACTTGCCGCCTCCGTTTTAGTTCTATTCCTCATGCCCCTCCTACACACATCCAAGCTGCGCTCAATGACATTCCGCCCT
CTCTCCCAAATCCTATTCTGAGCCCTAGTAGCCAACCTCCTCGTACTCACCTGAGTAGGCAGCCAACCAGTCGAACATCC
ATTCATCATCATCGGACAACTGGCCTCACTCAGCTATTTCACAATCATCCTGGTCCTCTTCCCCCTGGCATCCATCTTTG
AAAATAAACTCCTCAAACTATAA
Sitta
ATGGCACTCAATCTTCGTAAAACCCACCCATTAATAAAAGTCGTCAACGACGCCCTAATCGACCTCCCTACTCCATCAAA
CATCTCAATCTGATGAAATTTCGGATCGCTCCTAGGTATCTGCCTAATTACCCAAATCGTTACAGGCCTCCTATTAGCAA
CCCACTATACCGCAGACACCTCCCTAGCCTTCGCCTCAGTCGCCCACACCTGCCGAAACGTCCAATTCGGCTGACTCATC
CGCAACCTACACGCAAATGGAGCCTCATTCTTCTTCATCTGCATCTACCTACACATCGGCCGAGGCATCTACTACGGCTC
ATACCTAAACAAAGAAACCTGAAACGTAGGAGTCATCCTCCTCCTAGCCCTTATAGCCACCGCCTTTGTAGGCTACGTCC
TGCCCTGAGGACAAATATCATTTTGAGGAGCTACAGTAATTACCAACCTATTCTCTGCAATCCCCTACATCGGCCAAACA
CTAGTAGAGTGAGCCTGAGGCGGATTCTCAGTAGACAACCCCACACTAACACGATTCTTTGCCCTACACTTCCTCCTCCC
ATTCGTCATCGTAGGAGTCACACTAGTCCACCTCACCTTCCTCCACGAAACAGGCTCAAACAATCCACTAGGAATCCCCT
CAGACTGTGACAAAATCCCATTCCACCCATACTACTCCACAAAAGACATCCTAGGATTCGCTATCATGTTAATCCTTCTA
GTCACCCTTGCACTATTTTCTCCCAACCTGCTAGGAGACCCAGAAAACTTCACACCAGCCAACCCACTAGCCACACCCCC
ACACATCAAACCCGAATGATACTTCCTATTCGCATACGCCATTCTCCGATCCATCCCAAACAAACTAGGAGGAGTACTGG
CCCTAGCTGCTTCCGTCCTAGTCCTATTCCTCCTGCCCCTACTCCACACATCCAAACAACGCTCAATAACCTTCCGCCCA
CTCTCACAAATCCTCTTCTGAACCCTAGTAGCTGACCTCTTCATCCTAACCTGAGTAGGCAGCCAACCAGTCGAACACCC
ATTCATCATCATTGGCCAATTAGCCTCACTAGCCTACTTCACAATCATCCTAGTGCTCTTCCCACTCGCATCCCTCCTAG
AGAACAAACTACTCAAACTTTAA
Mimus
??
??GGCCTGCTACTAGCAA
CGCACTACACTGCAGACACCTCCCTAGCCTTCAACTCAGTAGCCCACATATGCCGAAACGTCCAATTTGGATGATTAATC
CGAAACCTGCACGCAAACGGAGCTTCATTCTTCTTCATCTGTATCTACCTGCACATCGGCCGAGGAATTTACTACGGATC
TTACTTAAACAAAGAAACCTGAAACATCGGAGTAATCCTCCTACTAACCCTAATAGCAACCGCCTTCGTAGGATACGTAC
TGCCCTGAGGACAAATATCATTCTGAGGGGCTACAGTAATCACTAATCTATTCTCTGCAATCCCCTACATCGGCCAAACA
CTAGTAGAATGAGCCTGGGGGGGATTCTCAGTAGACAACCCAACATTAACTCGATTCTTTGCCCTCCACTTCCTCCTCCC
ATTCGTCATTGCAGGACTAACACTAGTGCACCTCACCTTCCTACACGAAACAGGATCCAACAACCCCCTAGGAATCCCCT
CAGACTGCGACAAAATCCCATTCCACCCCTACTACACCGTAAAAGACATCCTAGGATTTGCACTAATAATCATCCTGCTA
GTATCCCTAGCACTATTCTCCCCCAACATACTAGGCGACCCAGAAAACTTCACCCCCGCCAACCCCCTAGCCACACCCCC
TCATATCAAACCTGAATGATACTTCCTATTTGCATACGCCATCCTCCGCTCCATCCCCAACAAACTAGGGGGAGTACTGG
CCCTTGCCGCTTCCGTGCTAGTCCTATTCCTAATACCTCTCCTCCATACATCCAAACTGCGCTCAATAACCTTCCGACCC
ATYTCACAAATCCTATTCTGAACTCTAGTCGCCAACCTCTTAGTCCTGACCTGAGTAGGCAGC?????????????????
??
???????????????????????
Sturnus_vulgaris
??GACGCCCTAATCGATCTCCCTACACCATCAAA
CATTTCGGCCTGATGAAACTTCGGATCCCTACTAGGCATCTGCCTAATTACACAAATCGTCACCGGCCTACTACTAGCCA
CACACTATACAGCAGACACCTCCCTAGCCTTCAACTCAGTAGCCCACATATGCCGAAACGTACAATTCGGCTGACTAATC
CGAAATCTACATGCAAACGGAGCATCATTCTTTTTCATCTGCATCTACCTACACATCGGACGAGGACTCTACTACGGATC
ATACCTAAACAAAGAGACCTGAAACGTCGGAGTAATTCTCCTACTAACGCTAATAGCAACAGCCTTCGTAGGATACGTAC
TGCCCTGAGGACAAATATCATTCTGGGGGGCAACAGTAATTACAAACCTATTTTCAGCAATCCCATACATCGGACAAACA
CTGGTAGAATGAGCCTGAGGAGGATTCTCAGTAGACAACCCCACACTAACCCGATTCTTCGCCTTGCACTTCCTACTGCC
ATTCCTCATTGTAGGGCTAACCCTAGTCCATCTAACCTTCCTACACGAAACAGGATCCAACAACCCACTAGGAATCCCCG
CAGACTGCGACAAAATCCCCTTCCACCCGTACTACTCTACAAAAGACATCCTAGGATTTGCACTAATACTCATTCTACTT
GTCTCCATAGCCCTATTCTCCCCAAACCTCCTAGGAGACCCAGAAAACTTTACACCAGCTAACCCCCTAGCCACACCCCC
TCACATTAAACCCGAATGATATTTCCTATTCGCATACGCCATTCTTCGATCCATCCCCAACAAACTAGGAGGAGTTCTCG
CCCTAGCCGCTTCCGTCTTAGTCCTATTCCTAATGCCTCTCCTCCACACCTCCAAACTACGCTCAATAACCTTCCGACCC
ATCTCGCAAGTCCTTTTCTGAACACTAGTTGCCAACCTCCTCATCCTCACCTGAGTGGGAAGCCAACCCGTTGAACACCC
ATTCATCATCATTGGCCAACTAGCCTCACTCTCCTATTTCACAATCATTCTAGTCCTA??????????????????????
???????????????????????

Creadion_carunculatus
??
??
????????????????????????TCGCCTTCACCTCCGTCGCCCACACCTGCCGAAACGTCCAATTCGGATGACTAATC
CGCAACCTACATGCAAACGGAGCCTCATTCTTTTTCATCTGCATCTACCTCCACATCGGCCGAGGCCTCTACTACGGCTC
ATACCTCAACAAAGAGACCTGAAACATTGGTGTCATCCTCCTCCTAACCCTTATAGCAACAGCCTTCGTAGGATACGTCC
TACCATGAGGACAAATATCCTTCTGAGGTGCTACAGTAATCACAAACCTCTTCTCAGCAATCCCATACATCGGCCAAACA
CTAGTAGAATGAGCCTGAGGTGGATTCTCAGTAGATAACCCAACACTAACCCGATTCTTCGCCCTCCACTTCCTCCTCCC
ATTCGTAATCGCAGGCCTGACACTAGTACATTTAACCTTCCTACACGAGACAGGCTCCAACAACCCGCTAGGAATCCCAT
CAGACTGCGACAAGATCCCATTCCACCCGTACTACTCCACAAAAGACATCCTAGGGTTCGCACTAATACTCATCCCACTT
GTCGCCCTAGCCCTATTCTCACCCAACCTCCTTGGAGACCCAGAAAACTTCACTCCAGCCAACCCCCTAGTCACACCACC
ACACATCAAACCCGAATGATACTTCCTATTCGCATACGCTATCCTACGCTCCATCCCAAACAAACTAGGCGGAGTCCTAG
CACTAGCCGCCTCCGTCCTTGTCCTCTTCCTCATCCCCCTCCTCCACAAGTCCAAACAAC????????????????????
??
??
???????????????????????
Parus
??
???TAACCCAAATCGTCACAGGCCTACTCCTAGCCA
TGCACTACACAGCAGACACCTCCCTGGCCTTCACCTCCGTTGCCCACACCTGCCGAAACGTTCAATTCGGCTGACTCATC
CGAAACCTCCACGCAAACGGAGCCTCCTTCTTCTTCATCTGCATCTACTTCCACATCGGACGAGGAATCTACTATGGCTC
TTACCTAAACAAAGAAACCTGAAACATCGGAGTTATCCTCCTCCTGACCCTCATAGCAACTGCATTCGTAGGCTACGTCC
TACCCTGAGGACAAATATCATTTTGAGGTGCTACAGTAATCACAAACCTATTCTCAGCAATCCCATACATCGGCCAAACA
CTAGTTGAATGAGCCTGAGGGGGATTCTCAGTAGACAACCCCACATTAACCCGATTCTTTGCCCTGCACTTCCTCCTACC
CTTCGTCATCGCAGGACTCACACTAGTCCATCTCACTTTCCTCCACGAAACAGGATCCAACAACCCCCTAGGAATCCCCT
CAGACTGCGACAAAATCCCATTCCACCCCTACTACTCCACAAAAGACATCCTAGGCTTCGCACTAATACTCATCATCCTC
GTCTCCCTAGCCCTATTCTCCCCCAACCTTTTAGGCGACCCAGAAAACTTCACTCCAGCAAACCCCCTATCCACCCCCCC
TCATATCAAACCCGAATGATACTTCCTATTCGCCTACGCCATCCTCCGATCCATCCCAAACAAACTAGGAGGAGTCCTGG
CCCTAGCCGCCTCCGTCCTAGTCCTATTCCTAATACCCCTACTCCACACATCTAAACAACGCTCAATAACCTTCCGACCC
CTATCTCAAGTCCTATTCTGAACCTTAGTCGCTAACCTCCTAATCCTGACCTGAGTAGGGAGCCAACCAGTCGAACATCC
ATTCATCATCATCGGCCAACTAGCCTCCCTATCCTACTTCACAATCATCCTAGTCCTATTTCCCCTTGCAGCCATCCTAG
AGAACAAAATCCTCAAACTTTAA
Petroica_rosea
ATGGCCCCAAACCTTCGTAAAAACCACCCTCTACTAAAAATCATCAACGACTCCCTAATTGACCTTCCCACACCATCTAA
CATCTCAACTTGATGAAACTTCGGCTCTCTCCTGGGCATCTGCCTAATCACACAAATTGTCACAGGACTTTTATTGGCCA
CACACTACACAGCAGACACTTCCTTAGCCTTCTCCTCCGTAGCCCACATATGCCGAAACGTCCAATTCGGATGACTGATC
CGAAACCTCCATGCAAACGGAGCTTCCTTCTTCTTCATCTGTATCTACCTGCACATCGGACGAGGCATTTACTACGGCTC
ATACCTAAACAAAGAAACCTGAAATATCGGAGTTATTCTCCTCCTAACCCTGATAGCAACTGCCTTCGTAGGCTACGTCC
TTCCATGAGGACAAATATCATTCTGAGGCGCTACAGTAATCACCAATCTATTTTCAGCCATCCCCTACATTGGCCAAACT
CTAGTAGAATGAGCATGAGGAGGCTTCTCAGTAGACAACCCCACTCTAACTCGATTTTTCGCTCTTCACTTCCTTCTGCC
ATTCGTAATCGCAGGCCTCACATTAGTCCACCTCACCTTCCTTCACGAAACAGGCTCAAATAACCCACTAGGCATTCCTT
CAGATTGCGACAAAATCCCATTCCACCCATACTACTCAACAAAAGACATTCTGGGCTTTGCCTTAATACTTATCCCACTC
ATTTCACTAGCTTTATTCTCCCCAAACCTGCTAGGAGACCCAGAAAATTTCACGCCGGCCAACCCC?TAGCCACACCTCC
TCACATCAAACCAGAGTGATATTTCCTATTTGCATACGCCATCCTACGATCCATCCCAAACAAACTAGGAGGCGTACTAG
CCCTAGCAGCCTCCGTCCTAGTCCTATTCCTCATACCTTTACTCCACACATCCAAACAACGCTCAATAACTTTCCGCCCC
CTCTCCCAAATTCTATTTTGAACTCTAGTAGCCAACCTCCTCATTCTTACTTGAGTGGGCAGCCAGCCAGTAGAACACCC
CTTCATTATCATCGGACAAGTAGCCTCATTCACCTACTTTGCCATCATTCTTGTCCTATTCCCCCTTGTATCACTCCTAG
AAAACAAACTACTAAAACTCTAA
Eopsaltria_australis
??
???CGTTACAGGCCTCCTACTAGCTG
CACACTACACAGCAGATACTTCCCTGGCCTTCACTTCTGTCGCCCACATATGCCGAAACGTCCAATTCGGCTGACTAATC
CGCAACCTCCACGCAAATGGAGCCTCATTCTTCTTTATCTGCATCTACTTTCACATCGGCCGTGGATTCTACTACGGCTC
ATACCTGAACAAAGAAACCTGAAACATTGGAGTTATCCTCCTACTAACCCTAATAGCAACCGCCTTCGTAGGGTACGTTC
TGCCCTGAGGACAAATATCCTTCTGAGGAGCTACAGTAATCACCAACCTATTCTCAGCTATCCCCTATATTGGTCAAACA
CTAGTAGAATGAGCTTGAGGGGGATTCTCCGTAGATAACCCCACACTCACCCGATTCTTCGCCCTTCACTTCCTCCTCCC
CTTCCTAATCGCAGGAATTACACTAGTCCATCTCACCTTCCTACACGAAACAGGATCAAACAACCCACTAGGAATCTCAT
CAGACTGCGACAAAATCCCATTTCACCCCTACTACTCAGTAAAAGACATCCTAGGATTTGTACTAATACTAATCCCACTA
GTTGCATTAGCACTATTCTCCCCAAATCTACTAGGAGACCCAGAAAACTTCACACCGGCCAACCCCCTAGCTACACCTCC
CCACATCAAACCCGAATGATACTTCCTATTTGCATATGCCATTCTACGATCTATCCCCAACAAACTTGGAGGGGTACTAG

CCCTGGCCGCCTCAGTCCTAGTCCTATTCCTCATTCCCCTACTTCACACATCCAAACAACGCTCAATAACCTTCCGCCCT
CTATCACAAATTC???
??
???????????????????????
Serinus
??
???????????????????TTCGGGTCTTTACTGGGCATCTGCCTAATCACCCAAATCGTTACAGGTCTTCTGCTAGCCA
CTCATTACACAGCAGATACCAACCTAGCCTTCTCCTCCGTAGCTCACATATGCCGCGACGTCCAATTCGGCTGACTAATC
CGCAACCTCCACGCAAACGGAGCCTCCTTCTTCTTCATTTGCATCTACCTACACATTGGCCGAGGAATCTACTACGGCTC
ATACCTAAACAAAGAAACCTGAAACATCGGAGTTATCCTACTATTAACCCTCATAGCAACCGCCTTCGTAGGTTACGTCC
TACCATGAGGCCAAATATCATTCTGAGGCGCTACAGTAATCACAAACCTATTCTCAGCAATCCCCTACATTGGACAAACA
CTAGTAGAATGAGCCTGAGGAGGATTCTCCGTAGACAACCCTACATTAACCCGCTTCTTTGCCCTCCACTTCCTACTCCC
ATTCGTTATCGTAGGCCTCACACTAGTTCACCTCACCTTCCTTCACGAAACAGGATCAAACAACCCAACAGGAGTTCCCT
CAGACTGCGACAAAATTCCATTCCACCCATACTACACCGTAAAAGACATTCTAGGTTTCGCACTAATAATCGCACTACTC
GTCTCCCTAGCCCTATTCTCCCCAAACCTCCTAGGAGACCCAGAAAACTTCACGCCAGCCAACCCCCTAGTAACTCCTCC
CCACATCAAACCCGAATGATACTTCCTATTCGCCTACGCCATCCTACGATCCATCCCAAACAAACTAGGAGGCGTACTAG
CCCTAGCCGCCTCAATCCTCGTACTATTCCTTATGCCCCTACTCCACACATCAAAACTACGATCAATAACTTTCCGTCCC
ATCTCACAAATCCTATTCTGAGCCCTAGTTGCAAACGTCCTCATCCTTACATGAGTAGGAAGCCAACCAGCAGAACACCC
ATTCATTATCATCGGCCAACTAGCCTCACTCTCATACTTCACAATCATTCTAGTCCTATTCCCCATCGCAGCCGCACTAG
AAAACAAACTCCTAAAACTCTAA
Icterus
??
??????????????????CTTTGGATCTCTACTGGGCGTCTGCTTAATCACTCAAATCGTCACAGGTCTTCTGCTAGCCA
TACACTACACAGCAGACACCAACCTAGCTTTCTCCTCCGTCGCTCACATATGCCGAGACGTACAATTCGGCTGACTCATC
CGCAACCTCCATGCAAACGGAGCCTCCTTCTTCTTCATCTGCATCTACCTTCACATCGGCCGAGGCATCTACTACGGCTC
ATACCTATACAAAGAAACCTGAAACATCGGAGTCATTCTCCTCCTAGCCCTCATAGCAACTGCCTTTGTAGGATACGTGC
TACCATGAGGCCAAATATCATTCTGAGGCGCCACCGTTATCACAAACCTATTCTCAGCTATCCCGTACATCGGACAAACA
CTAGTCGAATGAGCCTGAGGTGGCTTCTCCGTCGATAATCCCACACTGACCCGATTCTTTGCCCTTCACTTCCTCCTTCC
CTTCGTCATCGTAGGACTCACCCTCGTCCACCTCACCTTCCTGCACGAAACAGGCTCAAACAACCCTCTGGGCATCCCAT
CAGACTGCGACAAAATCCCTTTCCACCCGTACTACACCATTAAAGACGTCCTAGGATTTATTCTAATACTCTCCCTGCTC
GTCTCATTAGCCCTATTCTCCCCCAACCTCTTAGGGGACCCAGAAAACTTCACCCCAGCCAACCCCCTAGTCACCCCTCC
CCATATCAAACCCGAATGATATTTCCTATTTGCATACGCCATTCTTCGATCCATCCCAAACAAACTGGGAGGCGTATTAG
CCCTAGCCGCCTCAATTTTAGTCCTATTCCTCATACCATTACTTCACACATCAAAACTACGATCAATGACTTTCCGCCCT
CTATCACAAATCCTGTTCTGAGCCCTAGTAGCCAACGTCCTAATCCTAACCTGAGTGGCAGC???????????????????
??
???????????????????????
Motacilla
ATGGCCCCCAATCTTCGTAAAAACCACCAACTACTAAAAATCATCAATAATGCCCTAATTGACCTCCCCACACCATCAAA
CATCTCAACTTGATGAAACTTCGGGTCTCTTCTGGGCCTATGCCTAATCACCCAAATCGTTACAGGACTCCTGCTAGCCA
TACACTACACAGCAGACACCAACCTCGCCTTCTCCTCTGTAGCCCACATATGCCGAGACGTACAGTTCGGCTGACTCATC
CGCAACCTTCATGCAAACGGAGCCTCCTTCTTTTTCATCTGCATCTACCTACATATCGGCCGAGGACTTTACTACGGCTC
ATACCTGAACAAAGAAACCTGAAACATCGGAGTCATCCTCCTACTAACCCTAATAGCAACTGCCTTCGTAGGATATGTCC
TACCATGAGGCCAAATATCATTCTGAGGAGCTACCGTCATCACTAACCTATTCTCAGCAATCCCCTACATTGGGCAAACA
CTAGTAGAATGAGCCTGAGGGGGATTTTCAGTAGATAACCCCACATTAACCCGATTTTTCGCTCTCCATTTCCTCCTCCC
ATTCGTTATCGTAGGCCTCACATTAGTTCACCTCACCTTCCTTCACGAAACGGGATCAAACAACCCACTAGGCATCCCCC
CCGACTGCGACAAAATTCCTTTCCACCCATACTACACCATCAAAGACATCCTAGGATTCGCACTAATACTCTCCCTACTA
GTCGCCCTAGCTCTATTCTCCCCCAACCTCCTAGGGGACCCAGAAAACTTTACACCAGCCAACCCCCTAGTAACACCCCC
ACACATTAAACCAGAATGATACTTCCTATTTGCCTACGCTATCCTCCGATCCATCCCAAACAAACTAGGGGGTGTACTAG
CCCTAGCCGCCTCAATTCTCGTCCTATTCCTAACTCCACTACTTCATACATCAAAACTACGATCAATGACTTTCCGCCCT
ATCTCACAAATCTTATTCTGAGCCCTAGTAACCAACGTCCTCATCCTAACCTGAGTAGGCAGCCAACCAGTAGAACACCC
ATTCATCATCATTGGGCAACTAGCCTCATTCACCTACTTCCTAATCATTCTAGTCCTATTCCCCCTTGCAGGTCTCCTAG
AGAACAAGATCCTCAAACTCTAA
Emberiza
ATGGCCCTCAACCTTCGTAAAAACCACCGAATCCTAAAAATCATCAATGACGCCCTAATTGATCTCCCAGCACCATCAAA
CATTTCAACATGATGAAACTTCGGGTCCCTACTAGGCATCTGCCTAATCACCCAAATCATCACAGGCCTACTGCTAGCCA
TACACTACACAGCAGACACCAACCTGGCCTTCTCTTCTGTCGCCCACATATGCCGAGACGTCCAATTCGGCTGACTCATC
CGCAACCTACACGCAAACGGAGCTTCTTTCTTCTTCATCTGCATCTACCTACACATCGGCCGAGGCCTCTACTACGGCTC
CTACCTATACAAAGAAACCTGAAACATCGGAGTCATCCTACTCCTTATTCTCATAGCAACTGCCTTCGTAGGATATGTCC
TACCATGAGGACAAATATCATTCTGAGGAGCTACCGTAATTACAAACCTATTCTCAGCCATCCCCTACATCGGACAAACA
CTAGTAGAGTGAGCCTGAGGCGGATTCTCTGTCGACAACCCCACACTAACCCGATTCTTCGCTCTCCACTTCCTACTCCC

CTTCGTCATCGTAGGGCTCACCCTTGTTCACCTCACCTTCTTACACGAAACAGGCTCAAACAACCCACTAGGCATTCCCT
CAGACTGTGACAAAATCCCATTCCACCCATACTACACCATCAAAGATATCCTAGGATTTATACTCCTACTATCCCTACTC
GTCTCACTAGCTCTATTCTCCCCCAACCTCCTAGGTGACCCAGAAAACTTCACGCCTGCCAACCCCCTAGTCACTCCCCC
ACACATCAAACCCGAGTGATACTTCCTATTCGCCTACGCCATCCTCCGATCCATCCCAAACAAACTGGGAGGCGTACTAG
CCCTAGCTGCCTCAATCCTCGTTCTATTCCTCGTCCCCCTACTACATACATCAAAACTACGATCAATAACTTTCCGACCC
CTATCACAGATCCTATTCTGATCGCTAGTCGCCAACATCCTAGTCCTAACCTGAGTAGGCAGCCAACCAGTAGAACACCC
CTTCATCATCATTGGCCAACTAGCCTCACTCACGTATTTCACAATTATTCTAGTCCTATTCCCCCTTGCGGCACTCCTAG
AAAACAAACTACTCAAACTTTAA
Pomatostomus
??
???????????????????TTTGGGCTCCTTCTAGGAATCTGCCTAATCGTACAAATCGTCACAGGCCTGCTCCTAGCTG
CACACTACACAGCTGACACCTCCCTAGCCTTTGCCTCCGTAGCCCACATATGCCGCAACGTACAATTCGGATGACTAATC
CGAAACCTACATGCTAACGGAGCTTCATTCTTTTTCATTTGCATCTACCTGCATATTGGACGAGGACTCTACTACGGCTC
CTACTTAAACAAAGAAACCTGAAACATTGGAGTCATCCTGCTCCTAACCCTAATAGCAACTGCCTTTGTCGGCTACGTCC
TCCCCTGAGGACAAATATCATTCTGAGGGGCTACAGTCATCACAAATCTATTCTCAGCAATTCCATACATTGGCCAAACC
CTAGTAGAATGAGCCTGAGGAGGATTCTCAGTAGACAACCCCACACTAACCCGTTTCTTCGCCCTTCACTTCCTCCTACC
GTTCGTTATCGCAGGCCTCACACTAGTCCACCTAACCTTCTTACACGAAACAGGATCAAACAACCCCCTAGGAATCCCCT
CAGACTGCGACAAAATCCCGTTCCACCCCTACTACTCCACAAAAGACATACTAGGATTCGCACTCATACTTATCCCTCTC
ATCACCCTAGCCCTATTCTCACCCAACCTACTAGGTGACCCAGAAAATTTCACACCAGCAAACCCCCTAGCTACCCCTCC
ACACATTAAACCAGAATGATACTTCCTATTTGCATATGCCATTCTCCGATCCATTCCAAACAAACTGGGAGGGGTCCTAG
CCCTCGCTGCCTCCGTACTAGTACTATTCCTAATTCCATTACTACACACCTCTAAAGCCCGCTCAATAACCTTCCGTCCC
CTATCCCAAATCCTGTTCTGAACTCTAGTCGCCAACCTACTCGTACTAACATGAGTCGGCAGC?????????????????
??
???????????????????????
Rhipidura
ATGGCCCTCAACCTACGTAAAAATCATCCCGTCCTAAAAATCGTCAACGACGCCTTAATCGATCTTCCCACTCCATCCAA
CATTTCAACCTGATGAAACTTTGGGTCACTCCTAGGCATTTGCCTAGTTACACAGATCGTTACAGGCTTACTACTAGCCA
TACATTATACAGCAGATACTTCCCTAGCTTTCAACTCTGTAGCCCATATATGCCGAAACGTACAATTCGGATGACTAATC
CGAAATCTGCACGCAAACGGAGCTTCCTTCTTCTTCATCTGCATCTACCTACATATCGGCCGAGGATTTTACTACGGCTC
ATATTTAAACAAAGAAACCTGAAATATCGGAGTAATCCTACTACTAGCCCTAATAGCAACTGCTTTCGTAGGATACGTCC
TACCATGAGGACAAATATCATTCTGAGGAGCAACAGTAATTACTAACCTATTCTCAGCAATCCCATACATTGGACAAACA
CTAGTAGAATGAGCCTGAGGAGGATTCTCAGTAGACAACCCAACATTAACCCGATTCTTTGCCCTACACTTCCTCCTCCC
TTTCGTTATTGCAGGACTAACGCTAGTCCACCTCACTTTCCTACATGAAACAGGATCTAACAACCCTCTAGGCATTCCCT
CGGACTGCGACAAAATCCCATTCCACCCCTACTACTCTATCAAAGACATCCTAGGATTCGCACTAATGCTAATCCCACTA
ATCACTTTAGCACTATTCTCCCCAAACCTCCTAGGAGATCCAGAAAATTTCACGCCCGCCAACCCTCTAGCAACACCTCC
CCATATCAAACCTGAGTGATATTTCTTATTCGCATACGCCATCCTCCGATCCATCCCTAACAAACTAGGAGGAGTCCTAG
CCTTAGCTGCCTCAGTACTAGTCCTATTCTTAGCACCACTACTTCACAAATCCAAACAACGATCAATAACTTTCCGACCA
TTATCACAAATCCTATTCTGAGCTCTAGTTGCCAACTTACTTATCTTAACATGAGTTGGCAGCCAACCAGTCGAACACCC
ATTCATTATTATCGGACAACTAGCCTCACTCAGCTACTTCACAATCATTCTAGTACTATTCCCCATCGCAAACGTACTAG
AAAACAAATTACTAAACCTCTAA
Pica_pica
ATGGCCCTAAATCTACGCAAAAACCACCCCCTACTAAAAATCATCAATGACTCCTTAGTCGACCTTCCCACTCCATCAAA
CATCTCAGCTTGATGAAACTTCGGATCCCTACTAGGCATCTGCCTAATTACACAAATCATTACAGGCTTACTACTAGCCA
TACATTATACAGCAGACACCTCCCTAGCTTTTGCCTCAGTATCCCACATATGCCGCAATGTACAATTCGGATGACTAATC
CGAAATCTCCATGCAAACGGAGCCTCCTTCTTCTTCATCTGTATTTATCTACACATCGGCCGAGGATTCTACTACGGATC
ATACCTAAACAAAGAAACCTGAAACATCGGAGTAATCCTTCTCCTAACTCTAATAGCAACTGCTTTCGTAGGATATGTCC
TGCCATGAGGCCAAATATCCTTCTGAGGAGCTACGGTTATTACCAACCTATTCTCAGCAATCCCATACATTGGACAAACA
CTGGTAGAATGACTATGAGGAGGATTTTCAGTAGACAACCCCACACTAACCCGATTTTTTGCCTTTCACTTCCTACTACC
CTTTGTAATCGCAGGCCTAACGCTAGTCCACCTAACCTTCCTACATGAAACAGGCTCAAACAATCCACTAGGAATCCCCT
CAAACTGCGATAAAATCCCATTCCACCCCTACTACTCCATCAAAGACCTACTAGGATTCGCACTAATACTCGCCCTACTT
GCTACTATAGCACTATTCTCCCCAAACCTCCTAGGAGACCCAGAAAACTTCACACCTGCCAATCCTCTAGCCACACCTCC
CCATATCAAACCTGAATGATACTTCCTATTTGCTTATGCCATCCTCCGATCTATCCCAAATAAATTAGGAGGAGTCCTAG
CCCTAGCTGCTTCAGTCCTAATCCTATTCTTAATGCCCCTGCTCCATGTCTCCAAACAACGATCCATGACTTTCCGACCC
CTATCACAAATCTTATTTTGAACCCTAGTCACCGACCTCCTTATCCTAACATGAATCGGAAGCCAGCCAGTCGAACACCC
ATTCATTATCATCGGCCAATTAGCTTCGTTCGCCTACTTCACAATCATCCTTGTCCTATTTCCCCTTGTGAGTGCGCTAG
AAAGCAAACTACTCAACCTCTAA
Manucodia
ATGGCCCTCAACTTACGTAAAAACCACCCTGTACTAAAAACCATCAACAACGCTCTAATCGACCTCCCCACTCCATCAAA
CATCTCAATTTGATGAAACTTCGGCTCCCTATTAGGCATCTGCCTCATCACACAAATTATCACAGGCCTACTGCTAGCCA
TACATTACACAGCAGACACTTCCCTAGCATTCGCCTCTGTAGCCCATATCTGCCGAAACGTACAATTCGGATGACTAATC

CGAAACCTGCACGCAAACGGAGCCTCATTCTTCTTCATTTGCATCTACCTCCATATCGGCCGAGGAATCTACTACGGTTC
CTACCTAAATAAAAAAACCTGAAACATTGGAGTAATCCTACTTCTAACTTTAATAGCAACTGCCTTCGTTGGCTACGTAC
TACCCTGAGGACAAATATCATTCTGAGGGGCTACAGTTATCACCAACCTATTCTCAGCCATCCCATACATTGGCCAAACA
CTAGTAGAATGAGCTTGAGGAGGATTCTCAGTAGACAACCCTACACTAACCCGATTTTTTGCCCTACACTTCCTACTCCC
ATTTGTAATCGCCGGACTAACACTAGTACACCTCACCTTCCTACATGAAACAGGCTCAAACAACCCTCTTGGAATCCCAT
CAGACTGCGACAAAATCCCATTTCACCCATACTATACCATCAAAGACATTCTAGGATTTGCATTAATAATCATCCCATTC
GTTACCCTAGCATTATTCTCCCCAAACCTCCTAGGAGACCCAGAAAATTTCACGCCAGCCAACCCTCTAGTCACACCTCC
ACATATCAAACCAGAATGATACTTCCTATTTGCTTACGCCATTCTACGGTCAATCCCCAACAAACTAGGAGGAGTCCTAG
CCCTAGCCGCTTCAGTCCTAGTCCTATTCCTCCTTCCTCTCCTCCACACATCCAAACATCGCTCAATAACATTCCGACCC
CTATCACAAATCCTGTTTTGAACCCTAGTCGCCAACCTGCTCATCCTGACATGAGTGGGCAGCCAACCAGTCGAGCACCC
GTTCATTATCATTGGACAGGTAGCCTCATTCACCTACTTCTTAATTATTCTAGTCCTATTCCCAATTGCAAGTGTACTAG
AAAACAAAATACTTAAAATTTAA
Corvus_corone
??
???ATCATCACAGGTCTGCTACTAGCTA
TACACTACACAGCAGACACTTCTCTAGCCTTCACCTCCGTAGCCCACACATGCCGAAACGTACAATTTGGATGACTAATT
CGAAACCTCCACGCAAACGGAGCCTCCTTCTTCTTCATTTGCATCTACCTACACATCGGCCGAGGATTTTATTACGGTTC
ATACCTAAACAAAGAAACCTGAAACATCGGAGTAATTCTTCTCCTAACACTAATAGCAACCGCCTTCGTAGGGTACGTCC
TACCCTGAGGACAAATATCCTTCTGAGGAGCAACAGTCATCACAAATCTCTTCTCAGCAATCCCATACATCGGACAAACA
CTAGTAGAATGACTATGAGGCGGGTTCTCAGTAGACAACCCAACACTAACCCGATTTTTTGCCTTCCACTTCCTCCTCCC
ATTCGTAATAGCAGGACTAACACTAGTACACCTAACCTTCCTTCACGAAACAGGCTCAAATAATCCACTAGGAATTCCCT
CAGACTGTGACAAAATTCCATTCCATCCTTATTACTCCATCAAAGACCTACTAGGATTCGCACTAATACTCATCCCATTC
ATTGCCCTGGCTCTATTCTCCCCAAACCTCCTAGGAGACCCAGAAAATTTCACGCCCGCCAACCCTCTAGCCACACCTCC
ACACATTAAACCTGAATGATACTTCCTATTTGCATACGCCATTCTTCGATCCATCCCAAACAAACTAGGAGGAGTCCTGG
CCCTAGCCGCCTCAGTCCTAGTCCTATTCCTACTGCCTTTCCTCCACGTTTCTAAACAACGCTCCATAACTTTCCGACCC
CTATCTCAAATCCTATTCTGAATTCTAGTTGCAGACCTATTCATCCTAACATGAGTAGGAAGC?????????????????
??
???????????????????????
Vireo
??
??GGCCTCCTACTAGCCA
TGCACTACACCGCAGACACCTCCCTAGCCTTCGCTTCCGTGGCCCACACATGCCGAAACGTGCAATACGGATGACTAATC
CGAAACCTCCATGCAAACGGAGCCTCCTTCTTCTTCATCTGCATCTACCTGCATATTGGACGAGGGCTATACTACGGCTC
ATACCTAAACAAAGAAACCTGAAATGTAGGGGTAATCCTCCTACTGACTCTAATAGCAACTGCCTTCGTAGGGTACGTCC
TACCATGAGGACAGATATCGTTCTGAGGGGCCACAGTAATTACCAACCTATTCTCAGCAATCCCATACATTGGACAAACA
CTTGTAGAATGAGCCTGAGGGGGGTTTTCAGTAGACAACCCAACACTAACTCGATTCTTCGCTCTGCACTTCCTCCTACC
CTTCGTAATCGCAGGACTAACACTAGTACATCTAACCTTCCTCCACGAAACAGGGTCAAACAACCCCCTAGGAATTCCCT
CAGACTGCGACAAAATCCCATTCCACCCCTACTACACCATCAAAGACATTCTAGGGTTCGCCCTAATACTAACTGCCCTC
GTATCCCTAGCCCTATTCTCCCCAAACCTCCTAGGAGACCCAGAAAACTTCACGCCTGCCAACCCCCTAGCCACCCCACC
ACACATCAAACCCGAATGATACTTCCTATTCGCATACGCAATCCTACGATCAATCCCAAACAAACTAGGAGGAGTCCTAG
CCCTAGCCGCCTCCATTCTAGTCCTATTCCTAATACCACTCCTACACACCTCCAAACAACGCTCAATGACTTTCCGCCCC
CTATCACAAATCCTCTTCTGAGTACTAGTCGCCAACCTCCTAATTCTGACATGAATCGGCAGCCAACCAGTCGAACAACC
ATTCATCATCATCGGACAACTGGCCTCACTAAGCTACTTCACAATTATTCTAGTCCTATTCCCCATTGCAGGGATGCTAG
AAAACAAACTACTAAAGCTCTAA
Camptostoma_obsoletum
??
??
??
??
??
??
??
??
??
??
??
??
??
??
???????????????????????

Promerops_cafer
??
??
??
??
??
??
??
??
??
??
??
??
??
??
???????????????????????
Oriolus
??
??
??
??
??
??
??
??
??
??
??
??
??
??
???????????????????????
Dendroica
ATGGCCCTCAATCTTCGTAAAAATCACCAAATCCTCAAAATCATCAACGACGCCCTAATTGACCTCCCAGCACCATCAAA
CATTTCTACCTGATGAAACTTTGGATCTCTATTAGGCATCTGCCTAATTACCCAAATCGTTACAGGTCTTCTACTAGCTA
TACACTACACAGCGGACACTAACCTTGCCTTTTCCTCCGTCGCCCACATATGCCGAGACGTACAATTCGGCTGACTCATC
CGCAACCTACACGCAAACGGAGCCTCCTTCTTCTTCATCTGCATCTACTTACACATCGGTCGAGGACTATACTACGGCTC
ATACCTGAACAAAGAAACCTGAAATATCGGAGTTATTCTCCTCCTAACTCTCATAGCAACTGCTTTCGTAGGATACGTAC
TGCCATGAGGCCAAATATCATTCTGAGGTGCTACCGTAATTACAAACCTATTCTCAGCAATCCCCTACATCGGACAAACA
TTAGTAGAATGAGCCTGAGGAGGATTCTCCGTTGACAATCCCACACTAACCCGATTCTTCGCCCTCCACTTCCTCCTCCC
ATTCGTCATCGTAGGACTCACCCTAGTTCACCTAACCTTCCTCCACGAAACAGGATCAAACAACCCACTAGGCATCCCAT
CAGACTGCGACAAAATCCCTTTCCACCCTTACTACACTATCAAAGACATCCTAGGATTTGTACTAATACTCTCTCTGCTC
GTCTCACTAGCCCTATTCGCCCCTAACCTTCTAGGCGACCCAGAAAACTTCACCCCAGCCAACCCCCTAGTCACTCCACC
CCACATCAAACCTGAATGATATTTCCTATTTGCATACGCCATCCTCCGATCCATCCCAAACAAACTAGGAGGTGTCCTAG
CCTTAGCCGCCTCCATCCTAGTCCTATTCCTAACCCCACTACTACACACATCAAAACTACGATCAATAACCTTCCGTCCC
CTATCACAAATCCTATTCTGGACCCTAGTCGCAAACGTCCTCATCCTAACCTGAGTAGGCAGCCAACCAGTAGAACACCC
ATTCATCATCATCGGCCAACTAGCCTCATTCTCATACTTCATGATCATCCTAGTTCTATTCCCCCTCGCAGCCGCTTTAG
AGAACAAACTTCTCAAACTCTAA
Nectarinia
ATGGCCCTCAATCTTCGTAAAAACCACCGAATCCTAAAAGTCATCAACGACGCCCTAATTGACCTACCAACACCATCAAA
CATTTCAGTCTGATGAAACTTCGGATCCCTCCTAGGCATCTGCCTAGTCACTCAAATCGTCACAGGCCTGCTACTAGCCA
TACACTACACAGCAGACACCTCCCTAGCCTTCTCCTCCGTAGCACACATATGCCGAAACGTCCAATTTGGCTGACTAATC
CGCAACCTTCACGCAAACGGAGCCTCCTTTTTCTTCATCTGCATCTACCTACACATTGGCCGAGGAATTTACTACGGCTC
ATACCTATACAAGGAAACCTGAAACATCGGAGTTATTCTCCTACTGATCCTAATAGCAACCGCATTCGTAGGATACGTAC
TGCCCTGAGGACAAATATCCTTCTGAGGCGCTACAGTAATCACAAACCTATTCTCAGCAATCCCCTACATTGGCCAAACA
CTAGTAGAATGAGCCTGAGGGGGATTCTCCGTAGACAACCCCACACTAACACGATTCTTTGCTCTTCATTTCCTCCTACC
ATTCGTCATCGCAGGCCTCACACTAGTCCACCTCACCTTCCTACACGAGACAGGATCAAACAACCCTACGGGCATTCCAT
CAGATTGCGACAAGATCCCCTTCCATCCGTACTATACTACAAAAGACATCCTAGGGTTCGTACTTATACTCCTAACACTC
GCTGCCCTAGCCCTATTCTCCCCCAACCTCCTAGGTGACCCAGAAAACTTCACACCCGCCAACCCCCTGGTCACTCCTCC
CCACATCAAACCCGAATGATACTTCCTATTTGCCTACGCTATCCTCCGATCCATCCCAAACAAACTCGGAGGAGTCCTAG

CCCTAGCCGCCTCCATCCTAGTCCTATTCCTACTCCCCCTACTCCACACATCAAAACTGCGCTCAATAACATTCCGCCCC
CTATCACAAATCCTATTCTGAGCCCTAGTCGCCAACATTATAATTCTCACCTGAGTAGGCAGCCAACCAGTCGAACACCC
GTTCATCATCATCGGACAGGTAGCCTCCCTCTCCTACTTCACCATTATCCTAGTCCTATTCCCCCTAGTTTCCATCCTAG
AAAACAAACTACTCAAACTTTAA
Amytornis_striatus
??
???????????????????TTCGGATCTCTCCTAGGCATTTGCCTTATCACCCAAATCGTCACAGGCCTCCTGCTAGCTA
CACACTACACAGCAGACACCTCCCTGGCCTTCTCCTCCGTCGCCCATACATGCCGCAACGTCCAATTCGGATGACTCATC
CGCAACCTACATGCTAATGGGGCCTCACTCTTCTTCTTCTGCATTTACCTCCACATTGGCCGAGGTTTCTATTACGGCTC
CTACCTGAATAAAGAAACCTGAAACATCGGAGTTATCCTACTTCTAACCCTCATAGCAACCGCCTTCGTAGGCTATGTCC
TGCCCTGAGGCCAAATATCATTCTGAGGGGCTACAGTAATTACAAATCTATTCTCAGCAATCCCCTACATCGGACAAACA
CTAGTAGAGTGAGCCTGAGGCGGATTCTCAGTAGATAATCCTACACTCACCCGATTCTTTGCCCTCCACTTCCTCCTTCC
ATTCGTAATCGCAGGCCTCACACTAGTCCACCTCACACTCCTACACGAAACCGGCTCAAACAACCCCCTAGGAATCCCCT
CGGACTGCGACAAAATCCCATTCCACCCATACTACACTACAAAAGACATTCTAGGCTTTGCCCTAATACTTCTATCACTC
GCGGGCCTGGCCCTATTTTCCCCAAATCTCCTAGGAGACCCAGAAAACTTCACGCCAGCTAACCCCCTAGCCACACCTCC
CCACATCAAACCCGAATGATACTTCCTATTCGCATATGCTATCCTTCGATCCATCCCCAACAAACTAGGCGGAGTCCTAG
CCCTAGCTGCCTCCGTCCTAGTCCTATTCTTAATCCCCTTCCTACATGCATCCAAACAACGCTCAATGACTTTCCGCCCA
TTATCACAAATCCTATTTTGAATCCTGGTCACCAACCTCCTCGTCCTAACATGAGTTGGCAGCCAACCAGTCGAACACCC
ATTCATTATCATTGGACAACTGGCATCCTTCTCCTACTTTACTATCATCTTAATCCTGTTCCCAGTGGTAAGCGTCGTAG
AAAATAAACTACTCAACCTCTAA
Pitta
??
??
???????????????????????????????????TCCGTAACCCACACATGCCGAAACGTCCAATTCGGCTGACTGATC
CGAAACCTCCACGCCAACGGAGCTTCAGCCTTCTTCATCTGCATCTATCTCCATATCGGACGCGGACTTTACTACGGCTC
CTACCTATACAAAGAAACATGAAACACAGGAGTCATCCTCCTCCTCACCCTAATAGCAACCGCCTTCGTCGGCTATGTCC
TCCCATGAGGCCAAATATCCTTCTGAGGGGCCACAGTCATCACCAACCTATTTTCAGCCATCCCATATATTGGACAAACA
TTAGTAGAATGAGCCTGAGGAGGGTTCTCAGTAGACAACCCAACACTTACACGATTCTTCGCCCTACACTTCCTCCTCCC
ATTCATCATCGCAGGACTCACACTCATCCACCTCACTTTCCTACATGAAACAGGCTCAAACAACCCCCTAGGAATCCCGT
CAGATTGCGACAAAATCCCATTCCACCCATACTTCTGCATAAAAGACATTGTAGGCTTCATAATAATACTTCTGCCACTA
GCATCTCTAGCCATATTCTCACCCAACTTCTTAGGCGACCCAGAAAACTTCACACCTGCCAACCCCCTAGTAACCCCACC
CCACATCAAACCTGAATGATACTTCTTATTTGCATACGCTATCCTACGCTCCATCCCTAACAAACTAGGAGGCGTCCTCG
CCCTAGCTGCCTCAGTCCTAATCTTATTCCTCATACCCTTCCTACACAAATCTAAACAGCGAACAATAACCTTTCGCCCA
TTATCACAACTACTGTACTGAACCCTAGTAGCCAACCTCCTCATTCTTACATGAGTGGGCAGC?????????????????
??
???????????????????????
Toxorhamphus
??
??
??
??
??
??
??
??
??
??
??
??
??
??
???????????????????????
Orthonyx_temminckii
??
??
???????????????????????????????????TCCGTCTCCCACATCTGCCGAGACGTGCAATTCGGCTGACTGATC
CGCAACCTCCACGCCAACGGAGCCTCCTTATTCTTCATCTGCATCTACCTCCACATCGGCCGCGGATTCTACTACGGCTC
ATACCTAAACAAAGAGACCTGAAACGTCGGAGTAGTTCTCTTACTAGCCCTAATAGCAACTGCCTTCGTTGGTTATGTAC
TTCCCTGAGGACAAATATCCTTTTGAGGTGCTACAGTCATCACAAACCTATTCTCAGCAATCCCATACATCGGCCAAACA
TTAGTAGAATGGGCCTGAGGAGGATTTTCAGTAGACAACCCAACATTAACCCGATTCTTCACCCTACACTTCCTCCTACC

CTTCCTTATCGTAGGGTTAACATTAGTCCACCTGACCTTCCTACACGAGACAGGATCAAACAACCCCCTAGGAATCCCTT
CAGACTGCGATAAAATTCCGTTCCACCCATACTACTCTACAAAAGACATCCTAGGCTTCGTATTAATACTCACCCTGCTA
GCCTCCCTAGCCCTATTCTCCCCTAACTTCCTAGGAGACCCAGAAAACTTCACGCCCGCCAATCCTCTATCAACACCCCC
TCACATCAAACCAGAATGGTACTTCCTATTCGCATACGCCATCCTCCGATCCATTCCCAACAAACTAGGAGGCGTACTAG
CCTTAGCCGCCTCCGTCCTAGTACTATTCCTCCTCCCCCTCCTACACAAATCGAAACAACGATCAATAACCTTCCGACCC
CTATCCCAAATTCTATTCTGAGCCCTAGTGGCCAACTTACTCATCCTAACATGAGTCGGCAGC?????????????????
??
???????????????????????
Sericulus_chrysocephalus
??
????????????????????TTCGGATCCCTACTAGGAATCTGCCTAGTAATACAAATTGTCACCGGCTTCTACTTGCCA
TACACTACACGGCAGACACTAACCTAGCCTTTGCCTCCGTAGCCCACACATGCCGAAATGTACAGTTCGGGTGACTAATC
CGAAACTTACATGCAAACGGGGCCTCATTATTCTTTATCTGCATCTACCTACACATTGGACGAGGAATCTACTACGGATC
TTACCTAAACAAAGAAACTTGAAACATAGGGGTGATCCTCCTACTAGCCCTTATAGCAACCGCCTTCGTAGGATACGTCC
TCCCCTGAGGACAAATATCATTCTGAGGCGCTACAGTCATTACAAACCTATTCTCAGCCATCCCATACATTGGACAAACC
CTTGTAGAATGAGCACGAGGGGGATTCTCTGTAGATAACCCAACACT?????????CTTCGCCCTACATTTCCTCCTTCC
ATTTGTCATTGTAGGCCTCACACTAGTCCATCTCACATTCCTACACGAAACGGGATCCAACAACCCCCTAGGCATCCCAT
CGGACTGCGACAAAATTCCATTCCACCCATACTACACCACAAAAGACATTCTAGGATTCGCGCTAATACTCACCCTACTA
GTCTCCATAGCCCTATTTTCCCCTAACTTACTTGGAGACCCAGAAAACTTCACCCCAGCTAACCCTCTCTCCACGCCACC
CCATATTAAACCCGAATGATACTTCCTATTC?GATACGCTATCCTACGATCAATTCCCAACAAACTGGGAGGAGTCCTAG
CTCTAATCGCATCAATTCTAGTACTATTTTTCCTACCCCTAATACAC?CAT??AAACAACGC?CAATAACTTT??GTCCC
CTA???
??
???????????????????????
Cyclarhis_gujanensis
ATGGCCCTCAATCTTCGCAAAAACCACCCCCTACTAAAAATTGTAAACGACTCCCTAATCGATCTACCAACCCCATCAAA
CATCTCAACCTGATGAAACTTTGGCTCCCTTCTAGGAATCTGCCTAGTCACACAAATCATCACAGGACTACTACTAGCCA
CACACTACACAGCCGACACCTCCCTAGCATTCAATTCAGTAGCCCACACATGCCGAAATGTACAGTTCGGATGACTAATC
CGAAACATTCACGCAAACGGGGCCTCATTCTTCTTCATCTGCATCTACCTACACATCGGCCGAGGACTATACTACGGCTC
ATATCTAAACAAAGAAACCTGAAACATTGGAGTCATCCTACTACTAACCCTAATAGCAACTGCCTTCGTAGGATATGTCC
TACCATGAGGACAAATATCCTTCTGAGGTGCTACAGTAATCACAAACCTATTCTCAGCAATTCCGTACATCGGACAAACA
CTAGTAGAATGAGCCTGAGGAGGATTCTCAGTAGACAACCCAACACTAACCCGATTCTTCGCTCTACACTTCCTACTACC
ATTCGTCATTGCAGGACTAACACTAGTTCACCTAACATTCCTACATGAAACAGGATCAAATAACCCACTAGGAATCCCAT
CAGACTGCGACAAAATCCCGTTCCACCCCTACTACTCCATCAAAGACATCCTAGGGTTTGCCCTAATACTTATCTCACTA
ACTGCCCTAGCCCTATTCTCCCCAAACCTACTAGGAGACCCAGAAAACTTCACACCGGCCAACCCCCTAGCTACCCCACC
ACATATCAAACCTGAATGATACTTCCTATTCGCGTACGCTATCCTACGATCAATCCCAAATAAACTTGGAGGAGTACTGG
CCCTAGCTGCCTCCATCCTAGTTCTATTCCTAATACCACTCCTTCACACATCCAAACAACGATCAATAACCTTCCGACCC
CTATCACAAATCCTATTCTGAACCCTAGTCGCCAACTTACTAATCCTAACCTGAATTGGCAGCCAACCAGTTGAACAACC
ATTCATCATTATTGGACAACTAGCCTCACTCAGCTACTTCACAATCATCCTAGTCCTATTCCCAATCGCAGGACTACTAG
AAAACAAAATACTAAAACTATAA

[ND2]
Polyborus_plancus
??
??
??
??
??
??
??
??
??
??
??
??
??
?
Falco
ATGAGCCCCCTCACAAAACTAATATTCTCCTTAAGCCTTCTACTGGGAACAACCATTACAATTTCAAGCAACCACTGAAT

ACTAGCTTGAACTGGGTTAGAAATCAACACCCTTGCCATTATCCCCTTCATTTCAAAATCACATCACCCTCGAGCTGTCG
AAGCTGCAATCAAATACTTTCTCGTCCAAGCAACCGCCTCCGCACTAATCCTTTTCTCGAGCACAATCAACGCACAGCTC
ACCGGACAATGGGACATTACCCAATTAACCCAACCAACAGCCTCCCTCCTACTGACTATAGCAGTAGCAATAAAACTAGG
ACTAGTACCCTTCCATTTCTGATTTCCAGAGGTCATGCAAGGCTCACCCATAACCACCGCCCTACTCCTCTCCACATTAA
TAAAACTCCCCCCCATCACTATTTTATTCTTAACTGCCCCCTCACTAAACCCGTCTCTGCTAACAACCATAGCCATCGCA
TCCGCAGCTGTCGGAGGATGAATAGGACTCAACCAGACACAAATTCGAAAAATCCTAGCCTTCTCATCAATCGCCCACCT
GGGCTGAATAACCATTATTCTCATATACAACCCTAAACTAACAATAATAACCTTCTACTTATACTCCCTAATAACTGCCT
CCATTTTCTTAACCCTCAACACAACCAACTCATTAAAACTATCCACAATAATAACCTCCTGAACAAAAATACCAGCACTA
AACACATCCCTAATACTAGCACTGTTATCCCTAGCTGGCCTACCTCCACTCACTGGCTTCTTACCAAAATGACTCATCAT
TCAAGAATTAACTAAACAAGAGATATCAACCACAGCCATAATTATCTCAATACTCTCCCTACTAGGCTTATTTTTCTACC
TACGCCTTGCATATTGCTCTACAATCACACTTCCACCTAACACCACAAATTTCATAAAACAATGATACAGTAACAAACCT
ACAAACACGCCAACCGCTGCCCTAATTTCCCTATCAATCCTTCTCCTCCCCCTTTCTCCCACAATCTTAACCATCACCTA
A
Tyrannus
ATGAATCCCCAGGCTAAACTCATTTTCTCCATAAGCCTTGTGCTAGGAACAACCATCACAATTTCAAGCAACCATTGAAT
AATAGCATGAACCGGACTTGAAATTAATACCTTAGCCATCCTTCCTTTAATCTCAAAATCCCACCACCCACGGGCCATTG
AAGCCTCAACTAAATACTTCTTAGTCCAAGCAGCTGCTTCAACATTACTTCTCTTCTCCAGCATATCTAACGCTTGATTT
ACCGGCCAATGGGACATTACCCAGCTTACTCACCCAGCATCATGCATATTACTAACGGCTGCAATCTCAATAAAACTAGG
CCTGGTTCCATTCCACTTCTGATTCCCAGAAGTACTGCAGGGCTCTTCTCTTATAACAAGTCTACTACTTGCCACAATCA
TAAAATTCCCCCCAACCATTCTCCTCTTTCTGACCTCTCCTTCACTCAACCCTACCCTGCTATCCGTAATAGCCATTGCT
TCTGCAGCTTTAGGAGGCTGAATAGGTCTCAACCAAACCCAAATTCGCAAAATCATAGCCTTCTCATCCATCTCTCATCT
AGGCTGAATAACTATTATCCTCATTTATAATCCCAAACTTATACTAATCACCTTCTATCTCTATTCTCTTACAACAAGCG
CCATCTTCTTTGCCCTCAACTCAACCAACACCCTAAAACTATCCACTCTTATAACCATATGATCCAAAATCCCCATTCTA
GCTACAACTCTCATACTCACCCTCTTATCCCTTGCAGGCCTTCCCCCATTAACTGGATTCCTCCCCAAATGACTAATCAT
CCAAGAGCTAACTAAACAAGAACTCACTGCTACAGCAACTATTATCGCTCTACTCTCCCTCCTAGGACTATTCTTCTACC
TCCGCCTTGCCTACTGCGCAACTATCACCCTCCCACCTAACTCCGCTAACCACATAAAACAATGACAGACTAGCAAGACT
GTCAACTCATTAACTTCTACACTCATCACACTATCAATTATACTCCTGCCCTTATCCCCTACAATCCTCACAATCCCATA
G
Myiarchus
ATGAACCCTCAGGCTAAACTCATTTTCTTCATAAGCCTCCTCTTGGGAACAACCATCACAATTTCAAGCAATCACTGAAT
AATGGCATGAACTGGACTTGAAATTAACACCTTAGCTATTCTTCCCCTAATCTCAAAATCCCATCATCCACGAGCCATTG
AAGCTTCAACTAAATACTTCCTAGTCCAAGCAACTGCCTCAACACTACTTCTTTTCTCTAGCGCCTCCAATGCATGATCC
ACCGGCCAATGGGACATCACTCAACTCACCCATCCAGTATCATGCATCCTACTAACAACTGCAATCTCAATAAAACTAGG
TCTGGTCCCATTTCACTTTTGATTTCCAGAAATCCTTCAAGGCTCTTCCCTAACAACAAGCCTTCTACTAGCCACAATCA
TAAAATTCCCTCCAACCATTCTCCTTCTCCTCATCTCCCCCTCACTCAACCCTACTCTGTTATCTATGATGGCCATTGCT
TCCGCAGCCCTAGGGGGCTGAATAGGACTCAACCAAACCCAAGTCCGTAAAATCATAGCCTTCTCATCCATTTCCCACCT
GGGCTGAATAATTATTATCCTCATTTACAGCCCCAAACTTACACTAATCACTTTCTACATCTATTCCTTAACTACAGCCG
CTATCTTCTTCACCCTAAACTCTACTAATACTCTAAAACTATCAACTCTAATAACTATATGATCCAAAATCCCCACACTA
GCCGCAACCCTCATACTCACCCTCCTATCTCTTGCAGGCCTCCCACCTTTAACTGGATTCCTCCCCAAATGACTAATCAT
TCAAGAATTAACTAAACAAGAACTAACAACCACAGCAACCATCATTACCCTTCTTTCCCTCCTAGGACTTTTCTTCTATC
TCCGCCTTGCCTACTGTGCAACAATCACTCTTCCCCCAAACTCTGTTAACCACATAAAGCAATGACACACTAGCAAATCC
ATTGATATATTAACTTCCACCCTCATCACACTATCAATTATTCTCCTACCTTTATCACCTACAATCCTCACAATCCCATA
G
Hypocnemis_cantator
ATGAACCCCCAAGCCAAACTACTTTCCATCACAAGCTTATTTCTAGGAACAACTATCACAATCTCAAGTAACCACTGAAT
GATAGCATGAACTGGGCTAGAAATTAATACTTTGGCTATCCTCCCCCTCATTTCAAAATCTCATCACCCACGAGCCATCG
AAGCCGCAACAAAATATTTCCTAGTCCAAGCAGCTGCCTCAGCACTACTCTTATTTTCCAGCATAACCAACGCATGGTAT
ACCGGACAATGAGACATTACCCAACTCACCCATCCCACATCATGTCTACTTCTTACTACAGCCATTTCAATTAAACTAGG
ATTAGTCCCATTCCACTTCTGATTCCCAGAAATCCTCCAAGGTACATCCCTGATCACAGGCCTCCTACTAGCAACTGCTC
TTAAATTCCCACCAACTACACTACTTTTACTCACATCCTCTTCACTCAACCCCACTCTACTAGCCCTCATAGCAATTGCC
TCAACAGCCCTAGGTGGTTGAACTGGACTGAACCAAACCCAAACACGCAAAATCTTAGCCTTTTCCTCCATCTCCCACTT
AGGCTGAATAACCATTATTCTCGTATATAACCCCAAACTCACCTTAATTACCTTCTTCCTCTACTGCCTAATAACTATCC
CCATTTTCCTTACCCTCAACACAATCAAAACCCTAAAACTAACTACGATATCAACCGCATGGACAAAAATCCCCTCACTA
ACTGCAACCCTAATACTCATACTCCTGTCATTAGCAGGACTACCCCCACTAACAGGTTTCTTACCAAAATGGCTAATTAT
TCAAGAACTAACCAAACAAGAAATAACCCCAATAGCAACAATCATTGCCCTATTATCTTTACTCGGACTTTTCTTCTATC
TACGCCTTGCCTACTGTGCCACAATCACCCTACCTCCCAACTCTACTAACTACATAAAACAGTGACAAACCAATAAATCA
GCAAACATGACTATTACAACTCTTATCTCCCTATCCACTATACTCTTACCCCTCTCCCCCATGATCTCCACAATTCCTTA
G
Phlegopsis
ATGAGCCCTCTAGCCAAATTACTTTCCATAGCAAGTCTATTCTTAGGAACAACCATCGCAATCTCAAGCAACCACTGAAT

AATAGCCTGAGCCGGATTAGAAATTAACACCCTAGCCATCCTCCCCCTTATTTCAAAATCCCACCACCCACGAGCCATCG
AAGCCGCAACAAAATACTTCCTAGTCCAAGCAGCTGCCTCAGCATTACTCTTATTTTCTAGCATAACTAATGCATGATAT
ACTGGACAATGAAACATTACACAACTCACCCACCCTACATCATGCCTACTTCTCACAACAGCTATCTCAATCAAACTAGG
ATTAGTCCCATTCCACTTCTGATTCCCAGAAATCCTCCAAGGCACATCCCTAACCACTGGCCTCCTATTAGCAACAATCC
TAAAATTTCCACCAACCACATTATTCCTACTCACATCACCCTCACTTAACCCTACCTTATTAACCTTTATAGCAATCGCT
TCAACAGCCCTAGGTGGCTGAGCCGGATTAAACCAAACCCAAACTCGCAAAATCCTAGCCTTTTCTTCTATCTCCCATTT
GGGCTGAATAACAATTATCCTTATATATAATCCTAAGCTCACCTTAATCACCTTCTACCTCTACTGTTTAATAACCATCC
CCATTTTTCTTACCCTCAACACAATCAAAACCTTGAAACTAACCACAATAACAACTGCTTGAACAAAAATCCCCTCACTA
ACTGCAACTCTTATACTCATACTCCTATCATTAGCAGGATTACCCCCACTAACAGGCTTTTTACCAAAATGATTAATTAT
TCAAGAACTGACCAAACAAGAAATGACTCTAACAGCAACAATTATTGCCCTACTATCCCTGTTAGGACTCTTCTTCTACC
TACGCCTTGCATACTGTGCCACAATCACTCTTCCCCCCAACTCCACTAACCATATAAAACAATGACAAACCAATAAATCG
ACGAATATAACTATTACAACCTTAATCACCCTATCCACTATATTACTACCCCTCTCCCCCATGATCTTTACAACCCCTTA
G
Manacus_manacus
ATGAACCCCCAAGCTAAACTAATCTCCACAATAAGCCTGCTCTTAGGAACCTCAATCACAATTTCAAGTAACCACTGAAT
AATAGCATGAGTTGGCTTAGAAATTAACACTTTAGCTATTCTTCCATTAATCTCTAAAAGCCACCATCCACGAGCCATTG
AAGCCTCCACTAAATACTTTCTAACCCAGGCAGCCGCATCCACGCTACTTCTATTCTCCAGCATGACCAATGCATGATTC
TCTGGCCAATGAGACATCACCCAACTAACCCACCCCATATCATGCATACTACTAACTATTGCAATCTCAATTAAGCTAGG
CTTAGTCCCATTTCATTTCTGATTTCCAGAAGTTCTTCAAGGCTCAACTATGACAACATGCCTGCTACTAGCCACAGTCA
TAAAATTTCCACCAACCACACTATTCTACCTAACATCCCCTTCACTCAACCCAACTCTCCTATCTTCAATAGCCATTGCT
TCCGCTGCCCTAGGTGGCTGAATAGGACTTAACCAAACCCAAACTCGCAAAATTTTAGCTTTCTCATCTATCGCGCACCT
GGGATGAATAACCATTATTCTCAACTACAACCCCAATCTAACTTTAATTACGTTCTATCTCTATACTGTAACAACTTCTG
CCATCTTCCTCATCCTTAACACAACCAATACCCTAAAACTATCCACTGTTATATCCACATGAACCAAAATCCCATCACTC
ATCACAATCCTTATGCTCACCCTTCTCTCCCTCGCAGGTCTTCCTCCCCTAACAGGATTCCTACCCAAGTGGTTAATTAT
TCAAGAACTCACAAAACAAGAACTAACAGTACTAGCCACAGCTATTAGCCTACTCTCTTTACTTGGACTTTTCTTCTACC
TACGACTGGCCTACTGTGCAACCATCACACTACCCCCAAACTTCACCAACTACATAAAACAGTGAAAAACTAACAAACCA
ACTAACACCATAACCACAACCTTTACCTCCCTATCAATTATGCTCCTGCCACTATCTCCCATAGTACTCGCAATCCCCTA
G
Acanthisitta_chloris
ATGAACCCCCAAGCAAAATTAATCTCCCTTACTAGCCTACTCCTAGGCACAACCATCACAATCTCCAGCAATCACTGAAT
AATGGCCTGAACCGGCCTAGAAATCAACACTCTTGCTATTCTCCCTCTCATTTCAAAATCCCACCACCCCCGAGCCATTG
AGGCCACAATCAAATACTTCCTAGTACAAGCCTCTGCATCAGCACTAATTCTATTTTCTAGCACAATTAATGCTTGGACT
ACCGGCCAATGAGATATCACCCAACTAACCCACCCTCTAGCCTGCCTACTACTAACAACAGCAATTGCAATAAAACTAGG
ACTTGTCCCATTCCACTTCTGATTCCCAGAAGTCTTACAAGGCTCATCCCTGACCACTGCCCTCCTGCTCTCCACGGCCA
TGAAACTCCCTCCACTCGTTCTCCTCACCCTTTCACACCCATCACTCAACCCCCTACTACTAATCACCATGGCCGTTGCC
TCAGCAGCCCTAGGGGGATGAATAGGACTAAACCAAACCCAAACTCGCAAAATCCTGGCTTTCTCCTCCATCTCCCATCT
GGGGTGAATGGCTGCTATCAYCGTCTTTAACCCAAAACTCTCACTATTAACTTTCTACCTGTACGCTTTAATAACCTCCT
CTACATTCCTTACCCTTAACACAATAAAAACCCTCAAACTATCAACAATAATAACTGCATGGACAAAAACCCCTGCCCTA
AGCATAACCCTCATGCTCACCCTCCTCTCCCTAGCAGGACTCCCCCCACTAACAGGCTTCCTCCCAAAATGAATAATTAT
CCAAGAGCTAACTAAACAAGAAATAACCCCTGCAGCCACAATCATAGCCCTCCTGTCCCTGTTAAGCCTATTTTTCTACC
TCCGCCTGACATACTGCGCAACAATCACTCTGCCCCCAAACTCCGCAAATCACATAAAACAATGACGCATTAGCAAATCA
ACAAACCCCCTCCTCGCTCCCCTTCTAGCCCCGTCCATTATTCTACTTCCTCTCTCCCCCATAATCCCTGCAATTACCTA
G
Regulus
ATGAGCCCACAGGCAAAACTCGTCTTTACCTTAAGCTTAATTCTGGGTACAACCATAACAATTTCAAGCAACCATTGAGT
AATAGCTTGGATTGGCCTTGAAATCAATACCCTAGCCATTCTCCCTCTGATCTCAAAATCCCACCACCCACGGGCTATCG
AAGCCGCAACCAAATACTTCCTAGTCCAAGCAGCCGCCTCCGCCCTAGTCCTATTCTCGAGTATAACCAATGCATGAGAT
ACTGGACAATGAGACATTACCCAATTAACACATCCAATATCATGCATAGTATTAACATCAGCCATTGCAATAAAACTAGG
ACTAGTCCCCTTCCACTTCTGATTCCCAGAAGTCCTACAAGGATCCCCCTTAATCACTGGCCTACTGCTATCCACAATCA
TAAAATTCCCTCCAATTACCCTGCTCTTCATAACTTCACAATCATTAAACCCAATACTATTAACCACTATGGCTATCCTA
TCCGCAGCCCTCGGAGGATGAATAGGCCTAAACCAAACACAAACCCGAAAAATCCTAGCATTCTCCTCTATTTCCCATCT
AGGCTGAATAGCAGTCATCATCATGTACAACCCCAAACTCACTTTACTGAACTTTTACTTATACGCCCTAATAACTGCAA
CCGTATTCCTTATCCTAAACACAATTAAGGCCATAAAACTATCAACACTGATAACTACATGAACTAAAACTCCATCATTA
AACACAATACTACTACTAATTATACTCTCCCTAGCAGGTCTTCCACCATTAACAGGATTCCTGCCAAAATGACTGATCAT
TCAAGAACTAACCAAACAAGACATGACTCCAACAGCAACAATCATCTCACTCCTATCTCTACTAAGCCTATTCTTCTACC
TACGCCTTGCCTACTGTGCTACCATCACACTTCCTCCTCACACCTCAAATCATATAAAACAATGGCATATTAACAAGCCA
ACTCACCCCATAATCGCAATCCTAACTGTTATCTCCCTAATACTACTCCCTGTTGCCCCCATAATCATCACCGCAATTTA
A
Ailuroedus
GTGAACCCTCAGGCAAAACTAATCTTCACCGTTAGTCTACTCATAGGGACCACAATCACAGTCACGAGCAACCATTGAAT

TACAGCCTGAGCAGGACTCGAAATTAATACACTTGCCGTCCTCCCGCTCATTTCAAAGTCCCACCACCCACGGGCCATCG
AGGCCGCAACAAAATACTTCCTAACCCAAGCCACTGCTTCAGCCCTAATCCTATTCTCCAGCATGGCCAATGCATGGCAG
ACCGGGCAATGAGACATTATGCAACTTACCAGCCCCGTACCTTGCACCATCTTCACAGCAGCCATCGCAATAAAACTAGG
CCTAGTGCCATTTCACTTCTGACTCCCCGAGGTCCTCCAAGGCTCCTCCCTAATCACTGGCCTAATCCTATCCACCATCA
TAAAATTTCCCCCTATCACCCTTATATTCCTCACCTCAAAATCCCTAGACCCTACTCTATTAACCACCATGGCAATCCTG
TCAACGGTCCTAGGGGGATGAATGGGCCTAAACCAAACACAAGTCCGGAAGATCCTGGCCTTCTCATCCATCTCGCACTT
GGGCTGAATAACAATCGTTCTAGTCTACAACCCAAAGCTAACCATACTGAACTTCTACCTGTACTCCGTAATAACCGCCT
CTGCCTTCCTTACCCTACATGCCATAAAAACCCTAAAACTCCAAACACTAATAACTTCGTGGGCAAAGTCCCCAGCATTA
AGCGCCATATTCTTCCTCACACTTTTATCCTTGGCGGGCCTCCCTCCCCTAACCGGATTTATGCCAAAATGACTGATCAT
TCAAGAGCTCACAAAACAAGACATGGCCCTAGCATCAATCATTATCTCACTCGCATCCCTACTAGGGCTATTCTTCTACC
TACGCCTCCGATTCTGCG????AATCACCTTACCACCGCACACCACCAACCACATAAAACAGTGACGTACCAACAAACCA
ATCAGCGCCGTAGTGGCTATCCTAAGCACTCTCACCATGGCCCTCTTACCCATCTCCCCCATA????????????????
?
Zosterops
GTGACCCCCCAAGCAAAACTAATTTTTACTACCAGCCTCCTACTAGGAACAACCATTACAATTTCAAGCAACCATTGAAT
CATAGCCTGAGCCGGCCTTGAAATTAACACTCTAGCCATTCTCCCTATAATCTCAAAATCCCACCACCCCCGATCTGTTG
AAGCCGCAACCAAATATTTCCTAACCCAAGCAGCTGCCTCTGCATTAGTGCTATTTTCCAGCATAACCAACGCATGACAC
ACAGGCCAATGAGACATTACTCAACTAACCCATCCAACATCATCTCTTATCCTAACATCCGCCATCGCAATAAAACTGGG
ACTGGTCCCATTCCATTTCTGATTCCCCGAAGTCCTACAAGGCTCCCCCCTCACTACTGGGCTACTCCTATCCACAGTCA
TAAAACTCCCACCAATTACCCTACTCTTCATAACATCACCTTCCCTAAACCCCACACTACTCACATGCATAGCCGTACTC
TCAACAGCCTTGGGCGGCTGAATAGGACTAAACCAAACACAAATCCGAAAAATCCTAGCTTTCTCCTCCATCTCCCACCT
AGGTTGAATAGCCATCATTATCGCTTATAACCCCAAACTTACACTATTAAACTTCTACCTATACACCCTAATAACTACAG
CCGTATTCCTAACTTTAAACACAATTAAAACCCCCAACCTATCTACCCTGATAACAACATGAACAAAAATCCCCTCACTA
AACGCTATGCTCTTCTTAACCCTACTATCCCTAGCAGGACTTCCACCACTAACCGGCTTCCTACCTAAATGACTCATTAT
CCAAGAACTCACTAAGCAAGACATAATCCCAGCAGCAACAGCCATTTCCCTCCTATCCCTACTAGGACTATTCTTCTACC
TACGCCTTGCATACTGTGCCACAATCACACTCCCACCACACACCACAAACCATATGAAGCGATGACACATCAATAAACCA
ACCAGCACTACAATTGCCATCCTAATAGTTCTATCAACCACACTTCTTCCTATCTCTCCTATGATTACAACCATCATCTA
A
Onychorhynchus
ATGAACCCCCAAGCCAAACTTGTCTCCGCCATTAGCCTGTTCATAGGAACAGCCATTACAATCTCAAGCAATCATTGAAT
AATAGCATGAACCGGCCTTGAGGTCAACACCCTAGCCATCCTACCTCTAATTTCAAAATCCCACCACCCCCGAGCCGTCG
AAGCCGCAACCAAGTACTTCCTCGTCCAAGCAGCTGCTTCCACGCTACTCCTCTTTTCCAGCACCGTCAATGCATGACAC
ACTGGACAGTGGGACATCACCCAACTCACCCACCCTACATCCTGCATCCTACTGACTGTTGCAATTTCAATAAAACTCGG
CCTAGTCCCATTTCACTTCTGATTTCCAGAAGTACTTCAAGGTTCATCCTTATCAACCAGCCTTTTACTAGCCACAGTTA
TGAAACTCCCCCCAACTACACTTCTATTCCTCACATCCCCCTCACTCAATCCAACCCTACTATCTACGATAGCCATTGCC
TCCACAGCCCTAGGAGGCTGAATAGGTCTAAACCAAACTCAAATCCGTAAAATCCTAGCTTTCTCCTCTATCTCCCACCT
AGGTTGAATAACCATCATCCTAATCCACAGCCCCAAGCTTACCCTGATTACCTTCTACTTATACTCACTAACAACGGCTG
CTATTTTCTCAATTCTCAGCACAACCAATACCCTGAAATTGTCCACCATAATAACTTCATGAACAAAAATCCCCTCCCTA
ACTGCAATACTCATAGTTGCACTCTTATCCCTCGCCGGACTTCCACCATTGACAGGCTTTCTACCCAAATGACTTATTAT
TCAAGAACTCACTAAACAAGAGCTCACCGTCATTGCAACAATAATTGCCCTACTATCCTTACTCGGACTGTTCTTCTACC
TTCGCCTAGCTTATTGTGCAACAATTACACTCCCTCCCAACTCTACTAACCACATAAAACAATGACAAATCACCAAGTCA
GTAGACATCCCTACCACACTCCTAGCTACACTATCAGTCATGCTTCTTCCACTATCACCCATAATTCCCACAATCCTCTA
G
Nestor_notabilis
ATGAACCCCCATGCAAAACTCATCCTCATCCTAAGTCTTCTCCTAGGAACAACAATCACAATCTCAAGTAATCATTGAGT
GACAATGTGAGCTGGATTAGAAATCAACACCTTAGCCATCATCCCCCTAATCTCAAAATCCCACCACCCTCGGGCTATCG
AGGCAACAATTAAATACTTCCTAGTACAAGCAATAGCCTCAACACTAGTTCTCTTTTCAAGCATAACCAATGCCTGATGT
ACCGGACAGTGAGACATTACCCAACTCACCAACCCCCCGTCATGTCTTCTCCTAACTACTGCGGTTGCTATCAAATTAGG
GCTAGCCCCCTTCCACTTCTGATTCCCAGAAGTACTTCAAGGCACATCCCTCATCACAGCTATGCTACTATCAACAGTAA
TAAAACTTCCACCCACCATCATCTTATTTCTTACCTCACACTCACTCAACTCTACCCTACTTACCAGCCTGGCCATCGTA
TCAACTGCCCTAGGTGGTTGAATAGGACTTAACCAAACACAAACCCGAAAAATCTTAGCCTTCTCATCTATCTCCCACCT
CGGCTGAATAACCATTATCATTATCTACAACCCAAAACTTACTCTCCTAGCCTTCTACATCTACGTTATAATAACTACCT
CAATCTTCCTCACCCTAAACACAACTAACACTCTAAATCTATCAATGCTAACGACCTCATGAACTAAAACTCCTACACTA
AATACAACCCTTATACTCACCCTACTATCCTTAGCCGGCCTTCCCCCATTATCAGGCTTCTTACCAAAATGGCTCATCAT
CCAGGAACTCATCAAGCAAGAAATGACTACAACAGCCACAGTCATCTCCATACTCTCACTTCTCGGACTTTTCTTCTACC
TACGTCTAGCATACTGCTCAACAATTACACTTCCTCCCAACCCCTCAAACAAAATAAAACAATGACTCTTTAAAAAGCCA
ACCAGTGTCCTAGCCTCTATCCTTACCTCCTTATCCATCCTACTCCTCCCACTCTCCCCCATAATCCTCATCGCCACCTA
A
Leiothrix_argentauris
??????????????AAACTAGTCTTCATCTCTAGCCTCCTACTAGGCTCAACAATCACAATCTCGAGCAATCACTGAAT

CACGGCCTGGGCCGGCCTGGAAATTAATACCCTAGCCGTCCTCCCTATAATCTCAAAATCCCACCATCCTCGAGCCATTG
AAGCTGCGACCAAGTACTTTCTAACCCAAGCAACTGCCTCAACACTAGTGCTATTCGCCAGCATAACCAACGCATGATAC
ACTGGACAATGGGACATCACCCAAATAACTCACCCAACATCATGTCTAATTCTCACATCAGCTATCGCAATAAAACTAGG
ACTAGTCCCATTCCACTTTTGATTCCCAGAAGTACTCCAAGGATCCCCCCTTATAACTGGCCTCCTCCTCTCCACAGTAA
TAAAATTCCCACCCATCACGCTGTTCTTCATAACATCACCATCCCTAAACCAAACCCTACTAACACTCATGGCCATCATA
TCCGTAGCTGTGGGAGGATGAATGGGACTCAACCAAACACAGATCCGAAAAATCCTAGCATTCTCCTCTATCTCGCACCT
AGGCTGAATAACTATTATCATCTCCTACAACCCTAAACTCACCCTATTAAACTTCTACCTATACATCCTAATAACTGCAG
CCGTTTTCCTAACTTTAAACTCAATTAAAACCTTAAAACTGTCTACACTAATAACCACATGAACAGAAACCCCTGCACTA
AGCGCTATACTCCTCCTAACAATGCTCTCCCTCGCAGGACTACCACCCTTAACAGGATTCTTACCCAAATGACTCATCAT
CCAAGAACTAACTAAACAAGACATAGCTGCAGTAGCAGTGATAATCTCAATTCTCTCCCTGCTGAGCCTATTCTTCTACC
TCCGCCTAGCATACTGCGCCACAATTACACTCCCCCCTCATACCACAAACCACATCAAACAGTGACGCACTAACACCCCC
ATCAACACCATAATCGCCATCATAACCGTCACATCAACCTCACTCCTCCCTATCTCCCCAATAATCCCCACCATCATC??
?
Troglodytes
ATGAACCCCCAAGCAAAACTAGTCTTCACCATCAGCCTCCTTCTAGGAACAACCATTACCGTTTCAAGCAACCACTGAGT
TTTAGCCTGAACTGGCCTTGAAATCAACACCTTAGCCATCCTACCCCTAATCTCAAAATCTCACCATCCCCGAGCCATTG
AAGCCGCAACTAAATACTTCCTAGTTCAAGCAGCTGCCTCCACCCTAGTCTTATTCTCCAGCATAACCAATGCATGACAC
ACCGGACAATGAGACATCACCCAACTAACAAACCCCACCTCATCCCTAATCTTGACCACAGCCCTTGCAATAAAACTAGG
TCTAGTCCCATTCCACTTCTGATTCCCAGAAGTCCTTCAAGGCTCCTCCCTCCCCGTTGGTCTACTCCTATCAACAGCCA
TAAAATTTCCACCCATCACCCTACTATACATAACCTCGCCCTCACTAAACCCAACCTTACTAGTCACCATAGCCCTCCTC
TCCACAGCCCTAGGAGGATGAATGGGCCTAAATCAGACCCAAACTCGAAAAATCCTAGCCTTCTCCTCAATCTCCCACCT
AGGATGAATGGCCATTATTATCTCTTATAACCCCAAACTCACCCTACTAAACTTCTACCTATACGCCCTCATAACTGCAG
CAGTATTCCTCACATTAAATTCAACAAAAACCTTAAATCTGCCTACTCTAATAACCTCCTGAACAAAAACCCCAGCACTA
AACGCAATATTAATACTAACACTCCTATCCCTGGCAGGCCTCCCCCCTCTAACAGGATTCCTCCCAAAATGACTCATCAT
CCAAGAACTAACCAAACAAGAAATAGCCCCAGCGGCAACCATCATCTCCCTCCTATCTCTACTCGGCCTATTCTTCTACC
TCCGCCTCGCATACTGCGCAACAATCACCCTACCACCACACACTACCAACCACATGAAACAATGACATACCAACAAACCA
ACTAGCCCTATAGTAGCTATCTTAACCACCATGTCCCTTACACTTCTCCCAGTCTCCCCCTTAATCCTCCCCATCGTCTA
A
Luscinia_svecica
????ACCCCCAAGCAAAACTTATCTTTACCATTAGCCTGCTCTTAGGAACCACCATTACAATCTCAAGCAACCACTGAAT
CATAGCCTGAACCGGCCTCGAAATCAATACTCTGGCCATCCTCCCCCTAATCTCCAAATCCCACCATCCACGAGCCATCG
AAGCTGCAACCAAATACTTTCTAGTCCAAGCTGCCGCCTCTACCCTAATCCTATTCTCTAGCATAACCAATGCATGACAA
ACCGGTCAGTGAGACATCACCCAACTGACTTGCCCAACATCATGCCTAATCTTAACTGCAGCCATTGCAATAAAACTAGG
ACTAGCCCCTTTCCACTTTTGATTCCCCGAAGTATTACAAGGCACCTCTCTAACCACTGGCCTCCTCCTATCCACAGCCA
TGAAATTCCCCCCAATAACACTCTTCTTCATGACTTCCCAATCATTAAACCCAACCCTATTAACCATCATAGCCATTCTC
TCTGCTGCTCTGGGAGGATGAATAGGCCTAAATCAAACACAAACTCGAAAAATCCTAGCCTTTTCATCTATCTCCCACCT
GGGTTGAATGGCTATTATCATTATCTATAGCCCTAAACTAGCTCTACTAAACTTCTACCTGTACGTCTTAATAACTGCAG
CTGTATTCCTAACCCTAAACTCAATCAACACCCTAAAACTATCCACACTCATGACTACATGAACAAAAACCCCAGCATTA
AGTGCAACCCTGATATTAACCCTTCTCTCCCTTGCAGGTCTCCCCCCTCTAACAGGCTTCCTTCCTAAATGACTAATCAT
CCAAGAACTAACAAAACAAGAAATAGCCCCAACAGCAACTATTATTGCCCTTCTCTCCCTATTAAGCCTATTCTTCTACC
TCCGCCTAGCATACTGTGCAACAATCACCCTTCCCCCACACACTACAAACCACATAAAACAATGACACACTAACAAGCCA
ACCAATATCCTAGTGGCCATCCTAACCACCATGTCCATCATCCTTCTACCAATCTCACCAATAATCCTCACCATTGTGTA
A
Psittacus_erithacus
ATGAGCCCCCTTGCAAAACTCATCTCCACTACAAGCCTCCTACTAGGGACCACAATCACAATCACAAGCAACCACTGAGC
CATAGCCTGAACCGGACTAGAAATCAACACCCTTGCTATCATCCCCCTAATCTCAAAGTCCCACCACCCACGGGCTATCG
AAGCTACAACCAAATACTTCCTAGTACAAGCAGCCGCCTCAACACTAGTCCTGTTCTCAAGCATAACCAACGCATGAGCC
ACCGGACAATGAGACATTACCCAGCTCACCCACCCACCGTCATGTCTACTACTAACCACTGCCATTGCCATTAAACTAGG
CCTAACCCCATTCCACTTCTGATTTCCAGAGGTATTACAAGGATCCTCCCTCACCACCGCCCTGCTCCTCTCAACACTAA
TAAAACTTCCACCAACCACTATCCTACTCCTCACATCACACTCACTAAGCCCCACACTACTCACCGCCATAGCAATCATA
TCCATCGGCCTAGGCGGCTGAATAGGACTCAACCAAACACAAATCCGAAAAATCATAGCCTTCTCATCCATTTCACACCT
AGGTTGAATAACCACTATCATTATCTACAACCCCAAACTAACTCTACTAACCTTCTACATCTACAGCCTAATAACTACCT
CCATCTTCCTCACCCTAGACACAGCCAAGACCTTAAAACTAACCACACTAATAACCTCATGAACCAAAATCCCTATACTA
ACCACAACCCTTATACTGGCACTCCTATCGCTCGCAGGCCTCCCTCCACTAACAGGCTTTCTACCCAAGTGGCTCATCAT
TCAAGAACTCACAAAGCAAGAGATAATCGTAACAGCTACAATTATCTCCCTACTCTCATTGCTAGGGCTCTTCTTCTACC
TACGCCTAGCATACTGCTCAACAGTCACCCTCCCTCCGAACTCCTCAAACAAAATAAAACAGTGATCCACCAAAAACCCA
ACCAACACTCTAATTCCCATACTCACCTCTCTATCTACCCTACTTCTACCTCTCTCCCCTATAATCCCCACCATCACTTA
A
Ficedula_hypoleuca
?????CCCCCAAGCAAACCTAGTCTTTATCACCAGCCTATTTCTAGGAACCACCATTACAGTCTCAAGCAACCACTGAGT

TATGGCCTGGGCCGGACTCGAGCTAAACACCCTAGCTATCCTACCCCTTATCTCAAAATCCCACCACCCGCGGGCCATCG
AAGCCGCAACCAAGTACTTCCTAGTCCAAGCAGCCGCCTCCACCCTAATCTTATTCTCTAGCATAACCAATGCATGACAG
ACTGGACAGTGGGACATTTCTCAGCTAACCTGCCCCGTGTCATGCCTGATCCTAACCACGGCCATTGCAATAAAACTAGG
GCTAGCTCCCTTCCACTTCTGATTCCCCGAAGTGCTTCAAGGCTCTTCCCTAATCACTGGCCTCCTCCTATCCACAGCCA
TAAAGTTCCCTCCAATCACACTACTCTACATGACCTCCCAATCACTAAATCCAACCCTGCTAGTCACTATAGCCATCCTT
TCTGCCGCCCTAGGAGGATGAATAGGCCTAAACCAGACACAAACCCGAAAAATCCTAGCCTTCTCATCCATCTCCCACCT
AGGATGAATAGCCATCATCATTGTCTACAGCCCCAAACTAGCCCTACTAAACTTTTACCTGTATGCTCTAATGACCGCAG
CCGCATTCCTAACTCTAAACTCAATCAACACCCTGAAACTATCTACGCTTATAACTACATGAACAAAAACCCCAGCACTA
AGCGCAGCCCTAATATTAACCCTTCTCTCCCTTGCAGGCCTCCCCCCTCTAACAGGCTTCCTCCCCAAATGACTGATCAT
CCAAGAACTAACTAAACAAGAAATAGCCCCAGCAGCAACACTCATTGCCCTTCTCTCCCTACTAAGCCTATTTTTCTACC
TACGCCTCGCGTACTGCGCAACTATCACGCTTCCCCCACATACCACAAACCATATGAAACAGTGGCACACTAATAAACCA
ACCAGCATCCTAATCGCCATTCTAACCACCATATCCATCACCCTCCTGCCAGCATCACCTATA?????????????????
?
Menura_novaehollandiae
ATGAACCCCCAAGCAAAACTAATCTTCATCCTTAGCCTATTCCTGGGGACAGCCATCACCCTATCAAGTAGTCACTGGGT
CATAGCCTGAACCGGCCTAGAAATCAACACACTTGCCATCCTCCCGCTCATTGCAAAGTCACACCACCCACGAGCCATTG
AAGCCGCAACTAAATACTTCCTAGTACAAGCAGCTGCCTCAGCCCTAGTACTTTTCTCTAGCACAACCAATGCACTATAT
ACCGGACAGTGAGATATCACTCAAATAACCCACCCTACCTCATGCTTACTAATAACAGCAGCCATCGCAACAAAACTAGG
CCTAGTACCTTTCCACTTCTGATTCCCAGAAGTACTCCAAGGATCTTCCTTAACCACCGGCCTATTACTATCCACAGTCA
TAAAATTTCCCCCAATCACCTTACTCTACATAACCTCCCACTCACTTAATCCCACGCTACTAGCAACCATGGCTATCCTC
TCAACAGCCATTGGCGGTTGAATAGGCCTAAACCAGACACAAATCCGAAAAATCCTTGCTTTCTCCTCTATCTCACACCT
TGGCTGAATAGCCATCATTCTAGTATATAACCCCAAACTCACTCTCCTCAACTTCTATCTATACGCCCTAATAACTGCAA
CCGTATTCCTCACCCTAAAAACGATAAAAACACTAAAACTATCCTCACTAATAACTGCATGAGCAAAAACCCCAGCATTA
AGCGCAACCCTCATACTAATTCTACTGTCCTTAGCTGGTCTCCCCCCACTAACAGGCTTCATACCAAAATGACTAATCAT
TCAAGAACTAACTAAGCAGGATATAGCCCCCACAGCAATAATCATCGCCCTACTCTCTCTATTAAGCCTATTCTTCTACC
TACGCCTGGCATACTGTGCAACAATCACCCTTCCCCCACACACCACAAACCACATGAAACGATGGCACGTTAACGAGCCA
GTCAGCCCTATTATTGCCATCCTTGCAACCCTGTCCCTCACCCTTTTACCGCTCTCCCCCATAGTCCTTACCATCTTCTA
A
Pycnonotus
ATGAACCCCCAAGCAAAATTAATCTTTACCATCAGCTTATTACTAGGAACAACCATAACAATCTCAAGCAACCACTGAAT
CATAGCCTGAGCTGGTCTCGAAATCAACACTCTAGCCGTTCTCCCAATGATCTCCAAATCCCACCACCCACGAGCCATTG
AAGCCGCTACCAAGTACTTTCTAACCCAAGCAACAGCCTCAGCCCTTGTCCTATTCTCCAGTATGACTAACGCATGATAT
ACTGGACAATGAGACATTACCCAACTCACCCACCCAATGTCATCCTTAATTTTAACCTCGGCCATTGCAATGAAATTAGG
ACTGGTTCCATTCCACTTTTGATTTCCTGAAGTACTACAAGGGTCCCCCCTTACCACTGGCCTTCTTTTATCCACAATAA
TAAAATTCCCTCCAATTACGCTACTATTCATAACATCCCCATCACTAAACTCAACTCTACTAACCTGCATAGCCATTCTC
TCTACCGCCTTAGGAGGATGAATGGGACTAAACCAAACACAAATCCGAAAAATCCTAGCCTTCTCCTCCATCTCCCACCT
AGGATGAATGGCTATTATCATCACCTACAACCCCAAACTTACACTACTAAACTTCTACCTCTACTCACTAATAACCGCAA
CCGTATTCTTAACCCTAAACTCAATCAAAGTCTCAAAACTATCTACTTTAATGACCACATGAACAAAAACCCCACCACTA
AGCGCCACTTTACTTTTAACACTATTATCCCTAGCAGGCCTCCCTCCTCTAACGGGATTCCTACCCAAATGACTCATCAT
CCAAGAACTAACCAAACAAAGCATAGCCGTCGCAGCAACCACAATTTCCCTCCTTTCACTACTAAGCCTATTCTTCTACC
TCCGCCTCGCATACTGTGCAACAATCACACTCCCCCCGCACACTACCAACCACATAAAACAATGACATACCAATAAACCA
ACCAACGCTCTAATCGCAATCTTAACCACTACATCCATTATGCTCCTCCCCATCTCCCCTCTACTATCCTCCATCACGTA
A
Donacobius_atricapilla
??
??
??
??
??
??
??
??
??
??
??
??
??
?
Hirundo_rustica
ATGAACCCCCAAGCTAAACTAATATTTGCCCTAAGCCTGTTGTTAGGAACAACCATTACCATCACAAGCAACCACTGAAT

CATAGCCTGAGCCGGGCTCGAGATCAATACCCTCTCCATTCTACCACTGATCGCAAAATCCCACCACCCACGAGCCATTG
AAGCCGCAACTAAATACTTTATAACCCAAGCAACCGCCTCAGCCCTAGTCCTATTCTCCAGCATAACTAACGCCTGACAC
ACTGGACAATGAGACATCACCCAAATAACACACACAACTTCATGCTTAATCCTAACCTCAGCCATTGCTATAAAACTAGG
AATAGTACCATTTCACTTCTGATTTCCAGAGGTCCTTCAAGGGTCCCCTATCATCACCGGTCTTATCCTATCCACTATCA
TAAAACTCCCCCCTATTGCACTACTATACATGACATCCCACTCACTAAACCCAACATTACTAACCCTAATGGCCATCATA
TCTGCGGCCCTAGGAGGATGAATAGGACTTAACCAAACACAAATCCGAAAAATCTTGGCATTCTCCTCCATCTCACACCT
AGGATGAATAGCCATCATTATCCCATTCAGCCCAAAACTCACTATACTAAACTTCTACCTATATGTCTTAATAACCTCAG
CCATTTTCCTCACCATAAATACAGTCAAAGTACTAAAGCTATCAACACTAATAACCTCATGAACAAAAGCACCAGCACTA
AATACAATACTACTACTGACCCTACTCTCACTGGCAGGCCTACCTCCCCTAACAGGCTTCCTACCTAAATGACTCATTAT
CCAAGAACTGACTAAACAAAATATAGCTCCAACAGCAATTATTATTTCACTATTATCTCTACTCGGCCTATTCTTCTATC
TACGCCTCGCATACTGCGCAACTATTACACTCCCCCCACACACCACAAACCACATGAAGCAGTGACATACCTACAAACCA
ACCAGCACTTTAATTGCCATCTCGATTGTCATATCTATCATACTTCTCCCAATCTCCCCCATAATCCCTACCATCCTATA
A
Lichenostomus
ATGAGCCCCCAGGCAAAACTAATCTTCGTCACCAGCCTACTCCTAGGATCAACAATCACAATCTCAAGCAACCACTGAAT
TATGGCCTGAACCGGCCTTGAGATCAACACACTAGCCATTCTCCCCATAATCTCAAAATCACACCACCCGCGCGCCATTG
AGGCAGCAACCAAATATTTCCTAGTCCAAGCAGCCGCCTCCACCCTCGTTCTGTTTTCTAGCATAACTAACGCATGGCAT
ACTGGACAGTGGGATATTGCACAGCTAACACACCCAACATCCTGCCTGATCCTGACAGCAGCCGTTGCCATAAAACTAGG
ACTAGTCCCATTCCACTTCTGATTCCCTGAAGTCCTCCAAGGCACTTCCCTCACCACCAGCCTCCTACTATCAACGGTCA
TAAAATTTCCACCGATTACCCTACTATACCTCACATCTAGCTCATTAAACCCTACACTGCTAACTACCCTGGCTGTGCTA
TCCACAGCCCTGGGAGGATGAATAGGCCTTAACCAGACGCAAATCCGCAAAATCCTGGCTTTCTCCTCCATCTCACATCT
AGGCTGAATGGCCATTATTCTCGCCTACAACCCTAAACTTACTTTACTTAACTTCTACCTGTACAGCCTAATGACTGCGG
CCGTATTCCTCACCCTAAACAGCATAAAAGCGCTAAAACTATCAACATTAATAACTGCATGGACAAAAGCCCCATCCCTC
AGCACTATTCTCATTATCGTATTGCTATCCCTAGCTGGTCTTCCCCCTTTAACAGGCTTCCTACCAAAGTGACTCATCAT
CCAGGAACTTACTAAACAAGACATAGCTCTCACAGCAATACTCATTTCCCTACTATCCTTACTCAGCCTATTCTTCTATC
TTCGCCTAGCATACTGTACAGCAATCACACTCCCCCCTCACACGACAAATCACATGAAACTATGGCACATTAACAAACCA
ACCAATGTTTCAATCGCCATCCTAGCCACCCTCTCCCTCACTCTCCTCCCCCTCTCTCCCGTACTACTTGCTATTGTTTA
?
Cnemophilus
??
??
??
??
??
??
??
??
??
??
??
??
??
?
Coracina
????CCCCCCAAGCAAAACTAATTTTTACCACTAGCCTTCTACTAGGAACTACCATTACAATTTCAAGCAACCATTGAGT
TATAGCCTGAACCGGCCTTGAAATCAACACACTAGCCATCTTACCATTAATCTCAAAATCCCACCATCCTCGAGCCATTG
AAGCCGCAACCAAATACTTTCTAGTGCAAGCTGCTGCTTCAACCCTGGTCCTGTTCTCTAGTATAACCAATGCATGACAT
TCAGGACAATGAGACATCACTCAACTAACTCACCCAGTGTCATGCCTTATTCTAACCGCAGCCATCTCAATAAAATTAGG
ACTGGTACCATTCCACTTCTGATTCCCAGAAGTACTTCAAGGTTCTCCCCTAATCACTGGCCTTATTCTATCAACAGTGA
TGAAATTCCCACCAATTACATTGCTCTACATAACATCCCAGTCACTAAACCCCACACTACTAGTAACTATAGCTATCCTT
TCTGCAGCCCTAGGAGGATGAATGGGCCTAAACCAAACACAAACCCGAAAAATTATGGCCTTTTCATCCATTTCACACCT
AGGCTGAATGACCATCATCATCATCTACAGCCCCAAACTAGCACTACTCAACTTCTACTTATACGCCATAATAACCGCAG
CCGTATTCCTAACCCTTAACTCAATCAAAGCCCTAAAACTAACAACCCTAATAACAGCATGAACGAAAGCACCATCACTC
AGTGCAATCCTCCTACTCACCCTCCTATCACTTGCCGGCCTCCCCCCCTTTAACAGGATTCCTCCCAAAATGACTCATCAT
CCAAGAATTAACTAAACAAGGCCTAGCTCCTGCAGCAATAACCATTTCACTTCTCTCCCTACTAGGCCTATTTTTCTACC
TACGCCTCGCATATTGCGCAACAATTACACTTCCCCCTCACACTACGAACCACATAAAACAATGGCACATCAACAAACCA
GTCAATCCTCTAATTGCCATTCTAACAACCATATCCATTGCCCTACTTCCCATCTCCCCCATGATCCTCACCATTGTCTA
A
Dicrurus
ATGAACCCCCAAGCAAAAATAATTTTCATCACTAGTATACTGCTAGGGACAACTATTACAATCACAAGCAACCATTGAGT

TATAGCCTGAACAGGACTTGAAATCAACACACTAGCCATTTTACCATTAATCTCAAAATCTCACCACCCCCGGGCCGTTG
AAGCTGCAACTAAGTACTTCTTAGTACAAGCAGCTGCCTCAACCCTAGTACTATTTTCAAGTATAACCAACGCATGACAT
ACCGGCCAATGGGACATTACTCAGCTGACTCATTCAACCTCATGCCTAATCCTAACTGCGGCCATTTCAATTAAACTGGG
ACTAGTGCCATTCCACTTCTGATTCCCAGAAGTACTTCAAGGTTCATCCCTGACCACAGGTCTCCTGCTATCAACAATCC
TAAAACTCCCACCAATTACTTTACTATTTATAACCTCCCAGTCACTAAATCCAACATTACTAACAACTATAGCCATCCTG
TCTGCAGCTATCGGAGGATGAATAGGACTTAACCAAACACAAATCCGAAAAATCCTAGCCTTCTCCTCTATCTCACACCT
AGGCTGAATAACCATTATCCTCGTATACAACCCTAAACTCACATTACTGAATTTCTACCTATATACTATAATGACTTCAA
CCGTATTTCTAACCCTAAACTCAATAAAAGTCCTAAAACTTTCCACGCTAATAACTGCATGAACAAAAACACCATCACTT
AGTGCAATCTCCCTACTAACACTTATATCTTTAGCCGGCCTCCCCCCATTAACTGGATTCCTCCCAAAATGACTAATCAT
CCAAGAACTCACCATGCAAGAAATAGCCCCAGCAGCGATAATTATATCACTACTCTCTTTACTAGGACTATTCTTCTACC
TACGAGTTGCATACTGCGCTACAATTACACTCCCACCACACACCACAAACCACATAAAACAATGACATACCAATAAACCA
GTAAGTATAATAATCGCCATTTTAACCACTATATCAATTATGCTTCTTCCCATCTCTCCAATAATCCTTGCCATTATCTA
A
Gerygone_fusca
ATGAATCCCCAGGCAAAACTAGTTTTCATTATCAGCCTGCTTTTAGGAACAACTATTACTATCTCAAGTAACCACTGAGT
CATAGCCTGAACAGGCCTCGAAATCAACACACTCGCCGTCCTACCCCTAATCTCGAAATCTCACCACCCCCGAGCCATCG
AAGCCGCAACCAAATACTTTCTAGTCCAAGCAGCTGCCTCTGCATTAGTACTATTCTCCAGCATAACCAACGCATGACAA
ACCGGACAGTGGGACATCACCCAACTAACCGACCCCACAGCATGCCTAGTACTAACCGCAGCCATTGCAATAAAACTAGG
ACTGGTACCATTTCACTTCTGATTCCCAGAAGTACTTCAAGGCACTTCCTTAACAACCGGGCTCCTACTATCAACGGCCA
TAAAGTTCCCTCCAATCACACTACTCTTCCTAACCTCGCCCTCACTTAATCCTACATTACTAACTACAATGGCCCTTYTA
TCCGCAGCCCTAGGAGGATGAATAGGACTTAA?CCAACCACAAACTCGCAAATCCTAGCCTTTTCTCCATTTTCCCATCT
AGGTTGAATGGCCATCATCTTAGCCTCCAACCCAAAACTCACCCTGCTCAACTTTTACCTCTACACTTCGATAACCGCTG
CCGTATTCCTTACCCTAAATACAATAAAAGCCCTTAAACTCTCCACACTAATAACCGCATGAACCAAAACTCCCTCTCTC
AGCGCTATACTCCTTCTTACACTACTCTCCCTAGCTGGCCTTCCCCCTCTAACAGGCTTTCTCCCTAAGTGAGCTATCAT
CCAAGAACTAACCAAACAAGGCCTAGCGCCAGCAGCAATAGTAATCTCCCTCCTCTCCTTACTAAGCCTATTCTTCTATC
TCCGCCTTGCATACTGCACAGCAATTACACTCCCCCCACATACTACAAATCATATAAAACAATGACACGTCAACAAACCA
ACTAGTGTCCTAATTGCTAT??
?
Ptiloris_magnificus
ATGAACCCCCAAGCAAAACTAGTCTTTACTATTAGCCTGATCCTAGGGACGACAATCACAATCTCAAGCAACCACTGAGT
CATGGCCTGAGCCGGTCTTGAAATCAACACTCTCGCCATCTTACCCCTAATCTCAAAATCCCACCACCCCCGAGCCATCG
AAGCCGCAACCAAATATTTCTTAGTACAGGCAGCTGCTTCTACTTTGGTACTATTCTCCAGCATAACCAACGCATGACAC
ACTGGACAATGAGATATCACTCAACTGACCCACCCAGCATCATGCCTGGTAATTACTGCAGCCATCTCAATAAAACTAGG
ACTGGTGCCATTCCACTTCTGATTCCCAGAAGTACTTCAAGGGTCTTCCTTAACTACTGGTCTCCTATTATCTACAATCA
TAAAATTCCCACCAATTACCCTACTGTTTATAATCTCACAATCCCTAAACCCTACACTACTAACAGCCATGGCTATTCTC
TCCGCCGCCCTCGGAGGATGAATAGGACTAAACCAGACACAAATCCGAAAAATCATAGCCTTCTCTTCTATCTCCCATCT
AGGATGAATGGCTATCATCATCATCTACAACCCAAAACTTACCCTACTCAACTTCTACCTATACACTCTAATAACCGCAG
CCGTATTCCTAACCTTTAACTCAATAAAAGTACTAAAACTATCAACGTTAATAACCGCATGAACAAAAGCACCTTCACTT
AGTGCAATTCTCCTACTAACACTCCTATCTTTAGCCGGCCTCCCCCCTCTGACGGGTTTCCTCCCAAAATGACTCATTAT
TCAAGAACTAACCAAACAGGACATAGCCCCGGCGGCGGCAATTATTTCACTACT??CCCTATTAGGCCTATTCTTCTACC
TCCGCTTAGCATACTGTGCAACAATCACACTTCCGCCTCACACCACAAACCACATAAAACAGTGGCATGTCAACAAACCA
ATTAACATCTCAATTGCTGTTCTAACCACATTGTCCATCATGCTCCTTCCCATCTCCCCAATA?????????????????
?
Paradisaea_raggiana
?TGAACCCCCAAGCAAAACTAATTTTTACTATCAGCCTGGCCCTAGGAACAACAATCACAATCTCAAGCAATCACTGAGT
CATAGCCTGAACCGGCCTTGAAATCAACACTCTCGCCGTCTTGCCCCTAATTTCAAAATCTCACCACCCCCGAGCCATTG
AGGCCGCAACTAAATACTTTCTAGTACAAGCAGCCGCTTCCACTCTAGTGCTATTTTCTAGCATAACTAACGCCTGACAC
ACCGGACAATGAGATATCACTCAACTAACCCACCCAGTGTCATGTCTAGTACTTACCGCAGCCATTTCAATAAAACTAGG
CCTAGTGCCATTCCACTTTTGATTCCCAGAAGTACTGCAAGGATCTTCTTTAACCACTGGCCTTCTACTATCTACAATCA
TGAAATTCCCACCAATTACCCTACTATTCATAATTTCTCAATCCCTAAATCCAACACTACTAACAACCATAGCTATTCTT
TCTGCTGCCCTAGGAGGATGAATGGGACTAAACCAAACACAAATCCGAAAAATTATGGCCTTCTCCTCTATCTCTCACCT
AGGATGAATGGCTATCATCATCATTTACAACCCAAAACTAACTCTACTTAACTTCTACCTATACACCCTAATAACTGCAG
CTGTATTCCTGACTTTTAACTCAATAAAAGTACTAAAACTATCAACATTAATAACCGCATGGACAAAAGCACCCTCACTT
AGTGCAATTCTCCTACTAACGCTCTTATCTTTAGCCGGCCTGCCCCCTCTGACAGGTTTCCTCCCAAAATGACTCATTAT
TCAAGAACTAACTAAACAGGAAATAGCCCCAGCAGCAGCAAGCATT??????????????????????????????????
??
??
?
Cyanocorax_chrysops
ATGAACCCCCAAGCAAAGTCAATTTTTATTATTAGCTTACTCCTAGGGACAACTATCACAATCTCAAGTAACCACTGAGT

AATGGCCTGAACCGGCCTTGAAATCAACACACTCGCTATTTTACCCCTAATCTCAAAATCCCACCACCCCCGGGCCATCG
AAGCAGCTACTAAATATTTCTTAGTACAAGCAGCTGCCTCAACCCTAGTTCTATTTTCCAGCATAACCAATGCCTGACAC
ACTGGACAATGAGACATCACTCAAATGACCCATCCAACATCCTCATTAATCCTAACTGCAGCTATTTCAATAAAACTAGG
ATTAGTACCATTCCATTTTTGATTCCCAGAGGTGATGCAAGGCTCATCCCTTATCACTGGACTTATCCTATCCACAGTAA
TAAAATTCCCACCAATTACCCTATTATTTATAACCTCCCACTCACTAAACCCCACACTAATAACCACCATGGCTATTCTC
TCCGTGGCCTTAGGAGGATGAATAGGACTAAACCAAACACAAATTCGAAAAATCATAGCCTTTTCCTCCATTGCTCACCT
GGGCTGAATAGCTATCATCATTATCTACCACCCAAAACTATCCCTACTAAACTTCTATCTGTACGTCATAATTACTGCCG
CCGTATTCCTAAGCCTAAACTCAATGAAAGTCCTAAAACTATCAACGCTAATGACTGCATGAACAAAGGCACCTTCACTT
AGCACAATCCTCCTACTAACACTTCTATCCCTGGCCGGCCTCCCCCCTCTGACCGGTTTCCTCCCAAAATGACTAATTAT
TCAAGAGCTAACAAAGCAGGACATAACCCCATCAGCAATAATCATCTCACTTCTGTCATTACTAGGGTTATTCTTCTACC
TTCGCCTCGCATACTGTGCAACAATCACCCTACCCCCTCACACTACTAATCACATAAAACAATGACATACTAATAAGTCC
ATCAGCCCCCTAGTCGCTGTCCTAACAACCCTTTCTATTATGCTCCTCCCAATTTCCCCTATACTTACCACCATCGTCTA
A
Lonchura
ATGAACCCCCAAGCAAAACTAATCTTCACCATAAGCTTACTACTAGGCACTACTATTACAATCTCAAGTAACCATTGAAT
CATAGCCTGAGCCGGGCTAGAAATCAACACACTTGCCATCCTGCCATTAATCTCAAAGTCCCACCACCCCCGTGCCATCG
AAGCCGCAACCAAGTACTTCCTGACTCAAGCAGCCGCTTCGGCCCTAGTTCTGTTCTCCAGCATAACCAATGCATGACAC
ACCGGACAATGAGACATCACCCAACTAACCAACCCAACATCCTGCCTAATCCTAACCTCTGCAATTGCAATAAAACTAGG
CCTAGTACCCTTCCACTTCTGATTCCCAGAAGTACTACAAGGCTCCCCCCTAACCACCGGACTTATTCTATCCACAGCCA
TAAAACTTCCCCCCATGACCCTACTCTTCATAACTTCACCCTCCCTAAACTCCACTCTCCTAGTCACCATAGCTATTCTC
TCAACAGCCCTGGGAGGGTGAATAGGGTTAAATCAAACACAAACCCGAAAAATCCTAGCCTTCTCATCTATCTCTCACCT
AGGATGAATAGCCATCATCATTACGTACAACCCTAAACTAACCCTACTAAATTTCTATCTATATGCACTAATAACTGCAA
CCGTATTCCTGACCCTAAACTCAATTAAAGTCCTAAAGCTATCCACCCTCATAACTGCATGAACAAAAGCCCCATCACTA
AGTGCCATACTACTGCTAACTCTGCTATCACTTGCAGGATTACCTCCCCTAACAGGATTCCTCCCAAAATGACTGATCAT
CCAAGAACTAACCAAACAAGGTATGGCCTCAGCAGCCACAATCATCTCTCTACTATCACTACTAGGGCTTTTCTTCTACC
TTCGACTTGCATACTGCGCAACTATTACACTTCCCCCCCACACTGTCAACCATATAAAACAATGACACATTAACAAGCCG
ATCAACACTGTAATTGCTGTCCTAACCTCCCTGTCTATCACCCTACTCCCAATTGCCCCAATAATCCTCACCACTATCTA
A
Phylloscopus
ATGAACCCCCAAGCAAAACTAATTTTTACCATCAGCCTCCTACTAGGAACAACCATCACAATCTCAAGCAACCACTGAAT
CATGGCCTGAGCCGGCCTTGAAATTAACACCCTCGCTATCCTTCCACTAATCTCCAAATCCCACCACCCCCGAGCTATTG
AAGCCGCAACTAAATACTTCCTAGTTCAAGCAACTGCCTCCACTTTACTACTATTCTCCAGCATAACCAACGCATGACAT
ACTGGACAATGAGACATTACCCAATTAACCCACCCCATCTCCTGCCTAATTCTAACCTCTGCCATTGCAATAAAACTAGG
ACTAGTCCCATTCCACTTTTGATTCCCCGAAGTCCTGCAAGGAGCGCCACTCACAACCGGACTACTCTTAGCTACAGTCA
TAAAATTCCCCCCAATCACACTATTCTTTTTAACCTCCCACTCCCTAAACCCAACTCTGCTAACTTGCATAGCCATCCTT
TCTACCGCCCTAGGTGGATGAATAGGACTTAACCAAACCCAAATTCGAAAAATCCTGGCTTTCTCCTCCATCGCCCACTT
AGGCTGAATGACCATTGTTATCTCTTTCGACCCAAAACTAACCCTACTGAACTTCTACCTCTACAGCCTCATGACCGCAG
CCACTTTCCTAGCCCTAAACTCAATCAAAGCTTTAAAACTAGCAACTCTCATAACAACATGAACAAAAGCTCCCTCACTA
AACGCAATACTATTCTTAACCCTGCTCTCTCTAGCAGGCCTGCCCCCTCTCACAGGCTTCCTGCCCAAATGAATAATTAT
TCAAGAACTAACTAAGCAAAGCATAGCCCCAGCAGCAACCATAATCTCCCTCTTATCCCTACTAGGCCTATTCTTTTACC
TCCGCCTTGCATACTGCACCACAATCACACTGCCCCCCCACACCACAAATCACATGAAGCTATGACACACTAACAAACCA
ACCCACGTCTCGGTCGCCATTTTAACCACCCTATCTACTATACTCTTACCTATCTCTCCTATAATATACCCTCTATTGTA
A
Turdus
???AACCCACAAGCAAAACTAATTTGCACCATAAGCCTCCTCCTAGGGTCAACTATCACGCTCTCGAGCAACCACTGAGT
CACAGCTTGAACTGGGCTTGAAATCAACACCCTAGCCATCCTTCCACTAATCGCCAAGTCCCACCACCCACGATCAATCG
AAGCTGCAACTAAATATTTCCTAGTCCAAGCTGCTGCCTCTGCCCTAATCCTATTCTCCAGCATAACCAACGCATGATAC
ACGGGACAGTGAGACATCACTCAACTGACCTGCCCAGCCTCATGCCTGGTCCTGACCGCAGCCATCGCAATAAAACTCGG
ACTAGCCCCATTCCATTTCTGATTCCCCGAAGTTCTGCAAGGTTGCTCCCTAACCACCGGCCTACTCCTATCCACAGCCA
TGAAGTTCCCACCAATCGCATTATTCCTCATAACCTCACAATCCCTAAACCCAACCCCACTTATCACCATGGCCATTCTA
TCTGCAGCCTTAGGGGGATGGATAGGACTCAACCAAACCCAAATTCGAAAAATCTTAGCCTTCTCATCCATCTCCCATCT
AGGCTGAATGACCATCATCCTCGTCTACAGCCCAAAACTAGCCCTGCTAAACTTCTACTTATACGTAACAATAACTGCAG
CTGTTTTTCTAGCCCTAAACTCAATCAAAACTCTAAGCCTCTCCACACTAATAACCACCTGAACAAAAACCCCAGCATTA
AGCGCAATGTTAATGCTTACTCTACTTTCACTCGCAGGACTCCCGCCCCTAACGGGCTTCCTGCCCAAATGACTTATTAT
TCAAGAACTAACCAAGCAAAGTATAGCCCCAGCAGCAACAATCATAGCCCTCCTCTCCCTACTAAATCTGTTCTTCTACC
TTCGACTCGCATACTGCGCAACAATCACACTTCCACCTCACAGCACTAACCACATAAAACGATGACACATTAACAAACCA
GTCAGCCCCCTAGTCGCCATTTTAACCACCACATCCCTTACCCTTCTCCCAATTTCACCCATAATCTTCGCTATTGTC??
?
Acrocephalus
ATGAACCCCCAGGCAAAACTAGTGTTCATCGCTAGCCTCTTTCTAGGGACAATAATTACAATCTCAAGCAATCATTGAAT

CACAGCTTGAACAGGACTTGAGATCAACACCCTCGCTGTTTTACCCCTAATCTCAAAATCCCACCACCCCCGAGCCATTG
AGGCTGCAACCAAATACTTCTTAGCACAAGCAACCGCTTCTGCCCTGGTCCTATTCTCCAGCATAACCAATGCATGACAC
ACTGGACAATGGGACATCACCCAACTAACTCACCCAGTATCATGCCTGCTTCTAACCTCGGCCATTGCAATAAAACTCGG
ACTGGTACCGTTTCACTTCTGATTCCCAGAGGTACTTCAAGGCACCAGCCTAACCACCGGCCTGCTGCTATCCACAGCCA
TAAAATTCCCACCAATCACACTACTCTTCATGACATCCGCATCATTAAACCCAACAGTACTCACCTGCATAGCCGTACTA
TCTGCGGCCCTGGGCGGCTGAATGGGCCTAAACCAGACACAAATTCGAAAAATCCTAGCCTTCTCCTCCATCTCCCACCT
AGGCTGAATAACCATTATTATCCCCTACAGCCCCAAACTCACCCTACTAAACTTCTACCTATACGCCCTAATAACTGCAG
CCGTATTTCTCACTCTAAATTCAATAAAAGTATCAAAACTATCTACCTTAATGACAGCTTGGACAAAAATCCCCTCCCTA
AACGCAATGCTTCTACTAACCCTGCTCTCCCTAGCAGGGCTTCCACCAATAACAGGATTCCTGCCAAAATGACTCATCAT
CCAGGAACTAACCAAACAAGACATGGCCCCCGCAGCGACAATGATCTCCCTACTATCCCTACTAAGCCTATTCTTCTACC
TCCGCCTTGCGTACTGCACAACGATCACACTCCCACCACACACCCACAAACCACATAAAACAATGACACACTAGCAAGGCA
ACCAGCACCCTAGTCGCCTCCCTAGTCACCATATCAATCACACTGCTCCCTATCTCCCCCATCATTTACATCATCGTCTA
A
Sitta
ATGAAC??CCAAGCAAAACAAATCTTCGCCACCAGCCTACTCATAGGAACCACCATTACAATCTCAAGCAACCACTGAAT
CCTAGCCTGAACCGGCCTCGAAATCAACACCCTAGCCATCCTGCCTCTAATCTCAAAATCACACCACCCCCGCGCCATCG
AAGCTGCAACCAAATACTTCCTAGTACAAGCTGCAGCCTCAGCCCTAGTATTATTCTCAAGCATAACCAACGCTTGACAC
ACCGGACAATGAGATATCACCCAATTAACACACCCTACCTCATGCCTAATCCTAACCTCAGCAATCGCAATAAAACTAGG
CCTAGCCCCATTCCACTTCTGATTTCCAGAAGTCCTCCAAGGCTCCCCACTCACCACAGGCCTTCTCCTATCCACTGCCA
TAAAATTCCCACCAATCACCCTACTATACATAACCTCCCAATCACTAAACCCAGCCCTACTAACCACCATAGCCATCCTG
TCCGCAGCCCTAGGCGGGTGAATAGGCCTAAATCAAACACAAACCCGAAAAATCTTAGCCTTCTCCTCAATCTCCCACTT
AGGCTGAATAGCTATCATCATCACATACAGCCCAAAACTTGCTCTACTGAACTTCTACCTATACACCCTAATAACCGCAG
CAGCATTCCTCACCCTAAACTCAATAAAAGTACTAAAACTATCCACCCTAATAACCTCCTGATCAAAAACACCAACACTC
AGCGCAATACTACTACTAACACTACTCTCTTTAGCAGGCCTCCCCCCACTAACAGGATTCCTGCCAAAGTGACTTATCAT
CGAGGAACTAACCAAACAAGAAATAGCCCCAACAGCAACAATCATCTCACTCCTCTCTCTCTTAAGTCTATTCTTCTACC
TACGGCTAGCATACTGCTCAACAATTACACTACCCCCCCACACCACAAACCACATAAAACGATGGCACACTAACAAACCC
GTCAACGCCTCAATCGCCATCCTAACAACCCTATCTATCATACTCCTCCCCATCTCCCCCATCATCCTCACCATCATGTA
A
Mimus
ATGAACCCCCAAGCAAAACTAGTCTTTACCACCAGCCTAATCCTAGGGTCAACTATCACAATCTCGAGCAACCACTGAAT
TACAGCCTGAGCCGGGCTTGAAATCAACACCCTAGCAGTTCTACCACTAATCTCAAAGTCTCACCACCCCCGAGCCATCG
AAGCAGCAACCAAATACTTCCTAGTACAAGCAGCCGCCTCCGCCCTAATCCTGTTCTCTGGCATAACCAACGCATGAAAC
ACAGGACAATGAGACATTACCCAACTAACCTGCCCAACGTCATGTACAATCCTGACCACAGCCATTGCTATAAAACTAGG
ACTGGCTCCCTTCCACTTCTGGTTCCCAGAAGTACTCCAAGGCTGCTCCCTCCTCACAGGACTCCTCCTATCCACAGCCA
TAAAATTCCCACCAATCGTCCTCCTATTCATAACCTCCCAATCATTAAACCCCACCCTACTAACCACCATAGCCATCCTA
TCTGCAGCCCTAGGAGGATGGGCCGGACTGAATCAAACCCAAACTCGAAAAATCCTGGCCTTTTCATCCATCTCCCACCT
AGGTTGAATAGCCGCCATCGTCATCTACAGCCCTAAACTAGCCCTACTAAACTTCTACCTATATGCCATAATGACTGCAG
CCGTATTCCTCACCCTAAACTCAATCAAAACTCTAAAACTATCTACACTAATGACCACATGAACCAAAACCCCAGCACTA
AGTGCGATACTAATACTAACCCTACTTTCCCTGGCAGGACTTCCCCCTCTAACAGGATTTCTCCCCAAATGACTTATCAT
CCAAGAACTAACCAAACAAGAAACAATCCCAACCGCAGTGGCCATAGCCCTCCTCTCCCTACTAAGCCTATTCTTCTACC
TACGCCTCGCATACTGCGCAACAATCACCCTCCCCCCACACACTACAAACCACATAAAACAGTGGCACACAAACAAACCA
ATCAGCCCCATAGTTGCTATCCTAGTCACCATGTCCATCGTTCTCCTGCCCATATCCCCTACAATCCTCACCCTAGTCTA
A
Sturnus_vulgaris
ATGAACCCCCAAGCAAAACTAGTCTTTACCCTTAGCCTACTTCTAGGATCAACTATCACAATCTCGAGTAACCACTGAAT
TATAGCCTGGACTGGACTTGAAATCAATACCCTAGCGATCCTACCGCTAATCTCAAAATCGCACCACCCCCGAGCCATTG
AAGCTGCAACCAAGTACTTTCTAGTACAAGCAGCCGCCTCCACCCTACTCCTATTCTCCAGCATGACTAACGCATGACAC
ACTGGACAATGAGACATCACTCAACTAACCTGCCCAGTATCCTGCGTAATCCTTACTGCAGCCATTGCAATAAAATTAGG
ACTTGCTCCGTTCCACTTCTGATTCCCCGAAGTACTACAAGGCTGCCACCTCACCACCGGCCTACTCCTGTCCACAGTCA
TGAAATTCCCACCAATCACACTCCTCTATATGACCTCCCAATCACTAAACCCCTCCCTACTAACCGCCATAGCCCTCCTA
TCCGCAGCTGTGGGAGGATGAATGGGACTCAACCAAACGCAAACCCGAAAAATCCTTGCCTTCTCATCTATCTCCCACTT
AGGCTGAATAGCAATTGTCATCGCCTACAGCCCCAAACTAGCCCTACTCAACTTCTATTTATACGTAATAATAACCGCCG
CCGTATTCCTCACCCTAAACACAATTAAAACGCTAAAACTGTCCACACTAATAACCACATGAGCAAAAACCCCAGCTCTG
AGCGCGATACTAATACTGACTTTACTCTCCCTAGCAGGTTTACCCCCTCTGACCGGCTTCCTTCCTAAATGACTGATTAT
TCAAGAACTGGTCAATCAAGAAATAGCCCCAACAGCAACAATCATCGCCCTACTGTCCTTATTAGGTCTATTCTTCTACC
TCCGACTCGCATACTGTGCAACAATCACACTTCCCCCACATACGACAAACCACATAAAACAGTGGTATACCAACAAACCT
ACCAACACCTTAGTCGCCATTCTGGTCACAATATCCATCATCCTCCTACCCATCTCCCCTATACTCCTCACTATTGTCTA
A
Creadion_carunculatus
??

??
??
??
??
??
????????????????????????????TCAATCAGACACAAATTCGAAAAATCATAGCTTTCTCTTCCATTTCTCATCT
AGGCTGAATATCTATTGTCATCGTCTACAACCCCAAACTCACCCTACTCAATTTCTACCTGTACGCAATAATAACCACAG
CCACATTCCTCACCCTAAACTCCATAAAAGTCCTCAAACTATCAACGCTCATAACTGCATGAGCCAAAGCACCCTCCCTC
AGCGCAATACTAATGCTAACCCTGCTCTCCCTAGCCGGTCTTCCCCCTCTAACAGGCTTCCTCCCAAAATGACTCATCAT
TCAAGAGCTAACTAAACAAAGTATAGCTCCTGTGGCTATAATCCTATCCCTCCTCTCACTACTAGGACTATTCTTCTACC
TCCGATTAGCAT??
??
?
Parus
ATGAACCCCCAGGCAAAACTAATCTTCATCACCAGCCTACTCCTGGGCACAACCATCACGCTCTCGAGTAACCACTGAAT
CACAGCCTGAACAGGCCTTGAAATCAACACACTTGCCATCCTACCCCTTATCTCAAAATCCCACCACCCACGAGCAATCG
AAGCCGCCACTAAATACTTCCTAGTACAAGCAACCGCCTCCACACTAGTCCTATTCGCCAGCATAACCAACGCATGAGCC
ACCGGACAATGAGATATCACCCAACTAACCCACCCAGCATCCTGCATAATCCTAACTTCAGCCCTAGCAATAAAACTAGG
CCTAGTACCATTCCACTTTTGATTCCCCGAAGTACTCCAAGGCTCCCCCCTTATCACAGGCCTCCTCCTATCCACAGCCA
TAAAATTCCCACCAATCACCCTCCTCTTCATAACCTCCCCATCGCTCAACCCCATCTTACTCACCACCATGGCCATCCTC
TCCGCAGCCCTAGGAGGCTGAATGGGCCTAAACCAAACCCAAACCCGAAAAATTATAGCCTTCTCCTCCATCTCTCACCT
AGGTTGAATAGCCATAATCCTAGTCTACGATCCCAAACTCACCCTACTAAACTTCTACCTATATACCCTAATCACTGCAG
CCGTATTCCTAACTCTAAACACCATCAAAGCCCTAAACCTATCCACACTAATAACTACATGAACAAAAACCCCATCACTA
AGCGCAACCCTCCTATTAACCCTACTATCCCTTGCAGGACTTCCCCCTCTAACAGGATTCCTACCAAAATGAATAATTAT
CCAAGAACTAACCAAACAAGACATAACACCCACAGCAACGATCATGGCCCTACTCTCACTATTAAGCCTATTCTTCTACC
TCCGCCTTGCATACTGCGCAACAATCACACTCCCCCCACACACCACAAACCACATAAAACTATGACACACCCACAAACCA
ACTAATGCCTCAGTGGCCCTCCTAGTCACCATATCCACCCTACTCCTACCCATCTCACCTATGATCCACACCATTGTCTA
A
Petroica_rosea
?????????????????????ATTTTCGCCGCCAGTCTCCTCCTAGGAACAACCATCACAGTCTCAAGCAATCATTGAAT
CATAGCCTGAGCCGGCCTAGAAATCAACACATTAGCCATCCTGCCCCTAATCTCAAAATCCCACCACCCCCGAGCCATCG
AAGCTGCAACCAAGTACTTTCTAGTCCA?GCAACCGCCTCAACCCTGCTCCTATTTGCTAGCACGGTCAACGCATGGTCT
ACTGGTCAATGAGATATCACCCAACTAACCCACCCCATCTCTTGCGCAATCCTAACCGCAGCCATTGCAATAAAACTAGG
ACTAGTCCCATTTCACTTCTGATTCCCAGAGGTTCTTCAAGGCTCCCCCCTAATCACCGGCCTCCTCCTATCCACAGCCC
TAAAACTCCCACCCATTGCACTTTTTTACATAACCCACCACTCACTAGACACCACCCTACTAACCACCATAGCCATTCTT
TCCGTGGCCGTAGGGGGCTGAATAGGACTAAATCAGACACAAATCCGAAAAATCCTCGCCTTCTCCTCCATCTCACACCT
GGGCTGAATAGCCATCATCATTGCCTACCACCCAAAACTAACTCTACTAAACTTTTACCTGTACTCACTAATAACTGCAA
CCGTATTCCTCACTTTCCACTCAACCAAAACTTTAAAACTATCAACACTAATAACCTCCTGAACAAAAACACCAGCCCTA
AACGCAGCACTCCTGCTAACACTACTCTCCCTAGCAGGCCTCCCTCCCTTAACAGGCTTCCTCCCAAAATGACTCATCAT
CCAGAAACTAACAAACAAGAACATGGCCCCCGCGGCGGCAAAAATTTCTATGCTCCCCCTACTAGGTCTATTCTTCTACC
TTCGCCTTGCTTACTGTGCCACAATCACACTTCCACCTCACACTACCAACCACATGAAACAATGACACACCAATAAGCCA
ATCCCCTCCTTAATCGCTACTCTAATAACCATGTCCACCCTACTTCTCCCTATCTCCCCTACA?????????????????
?
Eopsaltria_australis
?????????????????????ATCTTC?CCATCAGCCTTATCCTAGGGACAACTATTACGATCTCAAGCAATCACTGAAT
TATAGCCTGAGCCGGCCTTGAAATTAACACACTAGCCATCTTGCCCCTAATCTCAAAATCCCATCACCCTCGAGCCATCG
AGGCCGCAACTAAATACTTCCTAACCCAAGCAGCTGCCTCCACATTACTATTATTCTCCAGCATGAACAACGCGTGATAC
ACCGGACAATGAGATATTACCCAACTAACACACCCCACTTCATGCCTAGTACTAACCACAGCCATTTCGATAAAACTAGG
ACTAGTCCCATTCCACTTCTGATTCCCAGAAGTCATACAAGGCACCTCTCTAATTATCGGCCTCCTACTGTCCACAGCCA
TAAAATTTCCACCAATCACCCTCCTCTATATAACCTCACCATCCCTAAATCCTACACTACTAACCACCCTAGCCATTTTA
TCCGTAGCCGTAGGCGGTTGAATAGGATTAAATCAAACACAAATCCGAAAAATCATAGCCTTCTCCTCCATCTCCCATCT
AGGATGAATGGCTATTGTGATCAGCTACAACCCCAAACTCACTCTACTAAACTTCTACTTATATACCATAATAACCGCAG
CTGTATTTCTTACCCTTAATTCAATTAAAGCCCTAAAATTGCCAACATTACTAACCTCATGAACAAAATCACCAGCACTA
AACACAGTCCTTCTACTTACACTACTTTCCCTAGCCGGCCTCCCACCACTAACAGGCTTCCTCCCCAAATGATTTATTAT
CCAAGAGCTAACCAAACAGGACATAGCCCCCGCAGCAACAGTAATCTCCCTACTATCCCTACTAAGCCTATTCTTTTACC
TTCGCCTTGCATACTGCGCAACAATCACGCTTCCCCCTCACACTACAAATCATGTAAAACAATGGCGTACCAACAAACCA
GTAAACCTCTCCATCGCCATCCTGACCACCCTGTCCCTAGCCTTACTCCCCATATCACCTATA?????????????????
?
Serinus
ATGAATCCACAGGCAAAACTAATCTTTACAATTAGTCTGCTCCTAGGAACTACCATCACCATCTCAAGCAACCATTGAAT

TATGGCCTGAGCCGGCCTAGAAATCAACACGCTTGCCATCTTACCCCTAATCTCAAAATCCCACCATCCACGATCCATTG
AGGCAGCCACTAAATACTTCCTAACCCAAGCAGCTGCCTCAACTCTAGTACTATTCTCCAGCATAACTAACGCATGACAT
ACCGGACAATGAGACATCACCCAACTCTCCCACCCTACATCAAGCCTAATCTTAACCTCGGCAATTGCTATAAAACTAGG
CCTAGTTCCGTTTCACTTCTGATTTCCAGAAGTATTACAGGGTTCCCCCTCTCCACCGGCCTCATCCTATCTACTATCA
TAAAACTTCCCCCAATCTCTCTCCTATATATAACATCCCCATCACTAAACCCCACACTCCTAACCACCTTAGCTATCCTA
TCAGCAGCTATCGGCGGATGAATAGGACTTAACCAAACACAGATCCGAAAAATCCTAGCCTTCTCTTCCATCTCCCATCT
AGGATGAATAACAATCATTATCATCTATAATCCTAAACTCACCCTTCTCAACTTCTACCTATATACTATAATAACCGCAG
CTGTCTTCCTAACCCTGAACTCAATAAAAGTACTAAAACTATCTACCCTAATAACAGCATGAACTAAAGTTCCATCACTG
AACGCAATACTACTCCTAACCCTACTATCCCTTGCAGGTCTACCTCCCCTAACAGGATTCCTACCCAAATGACTCATCAT
CCAAGAACTAACCAAACAAGAAATAATCCCTGCAGCTACACTCATTTCCCTTCTCTCTCTATTAAGCCTATTCTTCTACC
TCCGTCTTGCATACTGTACCACAATTACACTCCCACCACATACTACAAACCACATAAAACAATGACGTACCGGCAAATCA
ACCCACACCCTGATTGCCATCCTAACCACAATATCTGTCATTCTCCTTCCCATTTCACCTATAATCCTCACCATCATTTA
A
Icterus
ATGAATCCCATAGCAAGCCTGATCTTCACCACTAGCTTACTCCTAGGAACAACTATCACTATCTCAAGCAACCACTGAAT
CATAGCCTGAACTGGACTCGAGATTAACACACTCGCCATCCTCCCACTAATCTCAAAATCCCACCATCCACGAGCCATTG
AAGCTGCCACTAAGTACTTCCTAACTCAAGCAACTGCCTCTGCCCTTGTTCTATTCTCTAGCATGACCAATGCATGGGAC
ACCGGACAATGGGACATCACCCAACTTACCCACCCAACATCCTGCCTAATCCTCACCTCGGCGGTCGCAATAAAACTAGG
ACTAGTACCATTCCACTTCTGATTCCCAGAAGTCCTCCAAGGCTCTCCCCTAACCACTGGCCTACTCTTATCTACTATCA
TAAAACTACCACCAATCGCATTACTCTATATAACCTCCCCCTCACTAAGCCCTACACTCCTAACTACCCTGGCTATCCTC
TCAGTAGCCCTAGGAGGATGAATAGGCCTCAATCAAACACAAGTCCGAAAAATTCTGGCCTTCTCCTCTATTTCCCACCT
AGGATGAATAACAATTATTATCATCTACAACCCTAAACTCACTCTCCTCAACTTCTACCTATACGCTGTAATAACTGCAA
CTGTCTTCCTTACCCTAAACACAATCAAAGTCCTAAAGCTATCCACCCTAATAGCTGCGTGGGCTAAAACCCCAGCCCTA
AGCGCGATACTGCTCCTAACCCTACTCTCCCTCGCAGGCCTTCCCCCTCTAACAGGATTCCTACCCAAATGATTCATCAT
CCAAGAACTAACTAAACAGGACATAGCCCCTGCAGCCACACTCATTTCCCTCCTCTCCCTACTAAGCCTCTTCTTCTACC
TCCGACTCGCATACTGTGCAACCATCACACTCCCCCCGCACACTACAAACCACATAAAACAATGACGCACTAACAAACCA
ACCAACGTTACAATTGCCATCTTAACCACTGTGTCCCTCGTGCTTCTCCCTGTCTCCCCTATAATCCTCACCATCATCTA
A
Motacilla
ATGAATCCCCAGGCAAAACTAATTTTCATCACTAGCCTACTCCTAGGAACTACCATCACAATCTCGAGCAACCACTGAAT
CATGGCCTGGGCCGGCCTGGAAATTAACACACTAGCCATTTTACCGCTAATCTCAAAATCCCACCACCCGCGGGCCATTG
AAGCCGCTACTAAGTATTTCCTAGTACAAGCAGCCGCTTCTGCCCTAGTCCTATTCTCCAGTATGACTAACGCATGATGC
ACGGGACAATGAGACATTACCCAACTCACCCACCCAACATCATGCCTAATCCTAACTTCAGCTATCGCAATAAAACTAGG
ACTAGTACCCTTCCATTTCTGATTCCCAGAAGTACTGCAAGGCTCCCCTCTTACCACCGGCCTCCTGCTATCCACTGCCA
TAAAACTCCCGCCAATAACACTATTATACATAACTTCACCCTCGCTAAACCCCACATTACTAACCACCATAGCCATCCTT
TCAACTGCCTTAGGGGGATGAATAGGACTCAACCAGACACAAATCCGAAAAATTCTAGCTTTCTCATCCATTTCCCACCT
AGGCTGAATAGCAATTATCCTCGCCTACAACCCTAAACTCACCCTTCTCAACTTCTACCTATATGCACTAATAACTACAA
CTGTATTCCTTACCATAAACTCAATGAAAGTCCTAAAACTATCTACTCTAATAACAGCATGAACCAAAATACCATCACTA
AACGCAATACTGCTCCTAACTCTACTCTCACTTGCAGGACTCCCCCCTCTAACGGGATTCCTCCCCAAATGACTCATCAT
CCAAGAACTAACCAAACAAGACATGGCCCCAGCAGCTACAATCATCTCACTCCTCTCCCTACTAAGCCTGTTCTTCTACC
TCCGCTTAGCCTACTGCACAACAATCACACTCCCCCCACACGCTACAAACCACATGAAACAGTGACACACCCACAAACCA
ACCCCAACACTAATCGCCATCCTAACCACTATGTCCGTTACCCTACTGCCTGCCTCCCCCATAATTCTCGCTATCATCTA
A
Emberiza
ATGA?CCCCCAAGCAAACCTAATCTTCATCATCAGCCTAATCCTGGGGACAACCATCACTATTTCAAGCAACCATTGAAT
CATGGCTTGAGCAGGCCTTGAAATCAACACGCTTGCCATTCTCCCATTAATCTCAAAATCCCACCACCCGCGGGCCATTG
AAGCTGCTACTAAATATTTCCTCACCCAAGCAGCCGCCTCCACTCTTCTCCTATTCTCCAGCATAACCAACGCATGGCAT
ACCGGACAGTGAGATATTACCCAGCTCACTTACCCAACATCCTGCCTAATCCTCACTTCAGCAATCGCAATAAAATTGGG
ACTAGTACCATTCCACTTCTGATTCCCAGAAGTACTCCAAGGTTCCCCCCTCACCACCGGCCTTCTCCTATCCACCCTTA
TAAAACTCCCTCCAATTGCACTATTATACATAACCTCCGCCTCACTTAATCCTACCCTCTTAACAACCCTGGCCATCCTT
TCGACAGCCTTTGGAGGATGAATGGGCCTCAACCAAACACAAATCCGAAAAATCCTAGCCTTTTCTTCCATCTCCCACCT
AGGTTGAATAGCAGTTATCATTATTTACAACCCCAAACTCACGCTCCTCAATTTCTACCTATACAGCATGATAACCGCAT
CCGTATTCCTTGCCCTAAACACAATAAAAGTATCAAAGCTATCCACGCTGATGACTGCATGAACCAAAATCCCGTCCCTA
AACGCAATACTACTACTAGCCCTCCTCTCCCTTGCGGGCCTCCCCCCTCTAACAGGATTCCTGCCTAAATGACTCATCAT
CCAAGAACTAACCAAACAGGATATAGCCCCAACCGCCACACTCATTTCCCTCTTATCCCTGCTGAGCCTATTCTTCTACT
TACGACTCACATACTGCACAACCATTACACTTCCCCCACACACTACAAATCACATAAAACAGTGACGCACTAACAAACCA
ACCAACATCACAATTGCCGTACTGACTACCATGGCCGTCATACTCCTCCCTATTTCACCTATAATTCTACC?????????
?
Pomatostomus
ATGAACCCCCAAGCAAAACTAATTTTTGCTACCAGCCTCGTTCTAGGCTCCACCATTACAATCTCAAGCAACCACTGAAT

TACAGCCTGAGCTGGACTAGAAATTAACACACTCGCCATCCTCCCCTTAATCTCAAAATCCCATCATCCCCGAGCTATTG
AGGCTGCAACTAAATACTTCCTAGTACAAGCCGCTGCCTCCGCCCTAATCTTATTTTCTAGCATAACTAATGCATGACAC
ACTGGACAATGGGACATCACACAGATAACTCACCCAATTGCATGCCTGACTTTAACTGCAGCTCTTGCAATAAAACTAGG
CCTGGCACCATTCCACTTTTGATTTCCAGAAGTCCTCCAAGGCTCTCCTCTCACCATTGGCCTCCTTCTGTCAACAGTTA
TAAAACTCCCACCAATTGCCCTACTATACTTAACCTCTGCCTCACTCAACCAAACCCTGCTAACTACCATAGCTCTTCTT
TCCACAGCCTTAGGCGGATGAATAGGACTCAACCAAACACAAACTCGAAAAATTTTAGCTTTCTCATCTATTTCACACCT
AGGCTGAATAACCGCCATTATTGCCTACAACCCCAAGCTCACCCTACTCAACTTCTACCTATACACTATTATAACTGCAG
CTGTCTTCTTAACCCTCAACACAACTAAAGCCCTCAAACTATCAACACTAATAACCTCATACACAAAGGCACCCTTACTG
AATGCTGTCTTACTTCTAACATTACTCTCTTTAGCCGGCCTTCCTCCTTTTACAGGCTTTCTTCCCAAATGGCTCATCAT
CCAGGAACTAACTAAACAAGACATGGCTATTGTAGCAACAGCTATTTCTCTCCTCTCCCTACTAGGACTGTTCTTCTACC
TACGTCTTGCATACTGCGCTACAATCACACTCCCCCCTCACACCACAAACCACATGAAACAGTGACACACTAACAAACCA
ACTAACGTGCTAGTTGCCATTTTAACCGCCCTCTCCCTCACCCTCCTTCCCCTCTCTCCTCCA?????????????????
?
Rhipidura
ATGAATCCCCAAGCAAAACTAATCTTTACTGTAAGCCTGCTCCTGGGAACAACCATTACCATTTCAAGTAATCATTGAAT
CGCGGCCTGGGCCGGCCTTGAAATTAACACACTCGCCGTTTTACCCCTAATCTCAAAATCTCATCACCCTCGAGCCATTG
AAGCTGCAACAAAGTGCTTTCTAGTACAAGCAGCTGCCTCCGCCCTAGTCCTATTTTCAAGCATAACCAATGCATGACAT
ACTGGACAATGAGATATTACCCAATTAACCCACCCAATTTCTTCTTCAATCTTGACTACGGCCATTTCAATCAAACTAGG
ACTAGTTCCATTCCACTTCTGATTTCCAGAAGTACTTCAAGGCTCTTCTCTAATTACTGGCCTTATCCTATCAACAGCTG
TAAAATTTCCACCAATTACCCTACTTTACATGACCTCCCACTCACTCAACCCAGAACTCCTCACTGTTATAGCAATTCTC
TCTGTTGCCCTAGGGGGATGAATAGGACTAAATCAAACACAAATCCGAAAAATTCTAGCCTTTTCCTCTATCTCCCACCT
AGGCTGAATGGCTATCATCATCACCTATAACCCCAAACTTACTCTACTAAATTTTTACCTGTACTCCTTAATGACAGCAG
CTGTGTTCCTAACCCTGAATTCAATAAAAGTCCTAAAACTAACAACATTAATAACCACCTGAACAAAATCACCTTCACTT
AGCTCTATCTTCCTACTAACACTATTATCTTTAGCCGGCCTGCCCCCCTTAACAGGATTCCTCCCAAAATGACTCATTAT
TGAAGAACTAACTAAACAAGACATAGTCCCAACAGCAATAATCCTGTCGCTACTATCCCTCTTGGGACTATTCTTCTACC
TACGCCTTGCATATTGTGCAACCATTACACTACCCCCTCACACTACAAACCATATGAAAAAATGGCATACCAATAAACCC
GTAAACATCTCAATTGCCATTCTGACCACCATGTCCATCATGCTTCTTCCCATTTCACCTATAATCCTTACCATAGTTTA
A
Pica_pica
ATGAATCCCCAAGCAAAACTAATCTTCATCATCAGCCTATTTCTGGGAACTACCATCACAATCTCAAGCAACCATTGAGT
CATAGCTTGAACCGGCCTTGAAATTAACACACTTGCCATCTTGCCCCTAATCTCAAAATCTCACCACCCCCGGGCCGTTG
AAGCAGCAACCAAATACTTCCTAGTACAAGCAACTGCTTCAACCTTAGTACTATTTTCTAGCATAACTAACGCATGATAC
ACAGGACAATGAGACATTACCCAACTAACCCACTCAACATCCTCCCTAATCCTAACTGCAGCCATTTCAATGAAACTAGG
CCTAGCCCCATTCCACTTCTGATTCCCAGAAGTACTCCAAGGCTCTCCACTCACCACCGGCCTCCTCTTATCCACAGTCA
TAAAATTCCCACCAATTACCCTACTTTTTATAACTTCCCAGTCCCTCAACCCTTCTCTATTAACCATTCTCGCTATTCTC
TCAGTAGCCATAGGAGGATGAATGGGGCTAAATCAAACACAAACCCGAAAAATTATAGCCTTTTCCTCCATTGCCCATTT
AGGATGAATAACTATTATCCTTATTTATTACCCAAAACTCACACTACTTAATTTCTACCTATACGCTATAATAACCGCTG
CCGTATTCCTAACCCTAAACTCAATAAAAGTCCTGAAACTATCAACATTGATGACTGCATGAACAAAAGCACCTTCACTC
AGCACAATTCTCCTACTAACACTCCTATCCTTAGCCGGCCTCCCCCCCTCTAACCGGCTTCCTCCCAAAATGACTCATTAT
CCAAGAGCTAACTAAACAGGACATAGCCCCAGCAGCAATGATCATTTCACTTCTATCTCTCCTAGGACTCTTCTTCTACC
TTCGCCTAGCCTACTGTGCAACAATTACACTCCCACCCCACACAACAAACCACATAAAACAATGGCATACTAACAAGCCG
ATTAACCCATCAATCGCCGTTTTAACCACTCTCTCCATCATGCTCCTCCCAATTTCCCCTATACTCGCCACTCTAGTTTA
A
Manucodia
??
??
??
??
??
??
??
??
??
??
??
??
??
?
Corvus_corone
?????CCCCCAAGCAAAACTAATTTTTGTTACTA?CCTACTCCTAGGAACAACCATCACAATTTCAAGCAACCATTGAAT

TATGGCCTGAACCGGCCTTGAAATCAACACACTCGCTATCTTACCCCTGATTTCAAAATCTCACCACCCCCGAGCCATTG
AGGCAGCAACTAAATACTTTCTAGTTCAAGCAACTGCCTCAACCCTAGTACTATTCTCCAGCATAACTAATGCATGGTAC
ACAGGACAGTGGGATATTACCCAACTAACCCATCCAACATCCTCCCTAATCCTAACTGCAGCCATTTCAATGAAGCTAGG
ACTAGTGCCCTTCCACTTTTGATTCCCAGAAGTCCTACAAGGCTCCCCCATCATTACAGGCCTTCTCCTATCCACAGTCA
TAAAATTCCCACCAATTACCCTACTCTATATAACTTCCCTATCCCTAAATCCTACACTACTGACTACCCTAGCTATTCTT
TCCGTGGCCCTAGGAGGCTGAATAGGACTGAACCAGACACAAACCCGAAAAATCATGGCCTTCTCCTCTATCTCGCACCT
AGGCTGAATGGCCATTATCCTGATCTACTACCCTAAACTGACTCTTCTCAACTTCTACCTATATGCCATAATAACCGCTG
CCGTATTCCTAACCCTAAACTCAATAAAAGTCCTAAATCTATCTACACTAATAACTGCATGAACAAAAGCACCTTCACTT
AGCACAATTCTCCTACTAACACTCCTATCCCTAGCCGGCCTCCCCCCTCTAACTGGCTTCCTCCCAAAATGACTCATTAT
TCAAGAGCTAACCAAACAAGACATGGCCCCAGCAGCAATCATTATCTCCCTTCTATCACTGCTGGGCCTCTTCTTCTATC
TTCGCCTTGCATATTGTGCAACAATCACACTCCCACCACACACAACAAACCACATGAAACAGTGGCATACCAACAAACCA
ATCAATCCCTCAATCGCTGTTCTAACCACCCTGTCCATCATGCTCCTCCCAATTTCTCCCATACTTACTACCATTATCT?
?
Vireo
ATGAACCCCCAAGCAAAGCTAATCTTCGTATCCAGCCTAATACTAGGTACTACCCTAACAATCTCTAGCAACCATTGAAT
TACAGCCTGAGCGGGACTCGAAATTAACACACTCTCTATCTTACCCCTAATCTCAAAATCTCACCATCCGCGGGCAATTG
AAGCTGCAACCAAGTACTTCCTAGTTCAAGCAGCCGCCTCCGCCCTCGTCCTATTTTCCGGGATGACCAACGCTTGACAC
ACCGGACAATGAGACATTACTCAACTAACCCACCCGATATCATGCCTAGTCCTTACCGCAGCCCTTTCAATAAAACTAGG
ACTAGTCCCATTCCACTTCTGATTCCCAGAAGTACTACAAGGCTCCCCCCTAACCACAGGACTCATCCTATCAACGGTCA
TAAAACTTCCTCCAATCGCCCTACTCTTCATGACTTCCCACTCACTTAACCCCACAGTACTAACCACCCTGGCAATCCTA
TCCGTAGCTCTAGGAGGATGAATAGGCCTAAACCAAACACAAATCCGCAAAATCATAGCTTTCTCCTCCATTTCACACCT
AGGCTGAATAACCATTATCATCGTCTACAACCCTAAACTAACCCTACTTAACTTCTACCTATACGCTATAATAACCGCTG
CCGTATTCCTAACCTTCAACTCAATTAAAACCCTAAAACTATCCACCCTAATAACCACATGAACAAAAACACCCTCACTT
AGCATAATCCTCCTACTAGTACTCATATCACTCGCCGGCCTCCCGCCCCTCACAGGGTTCCTACCTAAATGACTCATCAT
CCAGGAACTAACTAATCAACATATAGCCCCAGCTGCAACAGCCGTCTCCCTACTCTCCCTGCTAAGCCTATTCTTCTATC
TACGACTGGCCTACTGCGCAACAATTACCCTACCACCACACACTACAAACCACATGAAACAGTGACACAGTGCCAAACCC
ATTAACCTATCAGTCGCTATCCTGGCCACCGCATCCACCATGATGCTACCCATCTCCCCCATGATCTTCACCGCCATCTA
A
Camptostoma_obsoletum
ATGAACCCCCAAGCTAAACTCATTTTCTCTATGAGCCTTTTTCTAGGGACAACCATTACAATTTCAAGTAACCATTGAAT
AATAGCATGGACCGGCCTTGAAATTAATACTTTAGCTATTCTCCCTTTAATCTCAAAATCACACCATCCACGAGCTACTG
AAGCCTCAACTAAGTACTTCTTAACCCAAGCAACTGCATCAACATTACTCCTATTCTCCAGCATGTCCAACGCATGATTT
ACTGGCCAATGGGATATTACTCAACTCACTCACCCAATATCATGTATACTACTCACTACCGCTATTTCAATAAAACTAGG
CCTAGTCCCATTCCACTTCTGATTYCCAGAAGTCCTTCAAGGTTCCTCCCTAATAACAAGCTTACTACTAGCAACACTCA
TAAAATTTCCCCCAACCATCCTCCTCTACCTAACATCCTCATCACTTAATCCTTCTCTACTATTCATAATAGCTATTGCT
TCAGCAGCTTTAGGAGGTTGAATGGGACTAAACCAAACCCAAGTCCGAAAAATCCTAGCTTTTTCATCTATCTCCCACCT
AGGCTGAATAATTATTATTCTCATTTATAATCCCAAACTAGCACTAATTACTTTCTATCTTTACTCTCTAACTACTGCAG
CCATTTTTTTGCCCTTAATTCTACTAATACCTTAAAACTATCGACCTTAATAACTGCATGATCTAAAGCCCCCACACTT
ACTGCAACCCTCATACTTGCCCTTCTATCTCTCGCAGGCCTTCCTCCTTTAACCGGATTTCTACCCAAATGACTAATTAT
CCAAGAACTCACTAAACAAGGAGTAACAGCAACAGCAACTATCATTTCCCTACTTTCCCTCCTAGGCTTATTCTTTTATC
TTCGTCTCGCCTATTGTGCGACAATCACTATCCCTCCAAACTCCATTAATCACATAAAACAATGACAAACTAACAAAACC
ATTAACACACTAACCTCATCCCTCACTACACTATCAATCATGCTTCTACCTCTATCACCTACAATTCTTACAATCCCATA
G
Promerops_cafer
ATGAACCCCCAGGCTAAACTAATTTTTATCATCAGCCTAATTCTAGGGACAACCATTGCAATCACAAGTAACCACTGGGT
CACAGCCTGAGCCGGCCTCGAAATCAACACACTAGCCATTTTACCCTTAATCTCAAAATCGCACCACCCGCGAGCCGTCG
AAGCTGCAACCAAGTACTTCCTGGTCCAAGCAGCCGCCTCCGCCCTAGTCCTATTTTCCAGCGTAACCAACGCCCTATGA
ACGGGACAGTGGGATATCACCCAACTCACCCAACCCATTTCCTGCCTAATTCTAACCTCAGCTATTGCAATAAAACTCGG
CTTAGCCCCATTCCACTTCTGATTTCCAGAAGTACTTCAAGGATCCCCTCTCATTACGGGCCTACTCCTATCAACAATCA
TAAAACTACCCCCCATCGCTCTGCTATACATAACCTCCTTCTCCCTGAATCCAACCCTACTAACGATCATGGCCATCCTG
TCCGTTGCCCTAGGAGGATGAATGGGCCTAAACCAAACACAAACCCGAAAAATCCTGGCCTTCTCCTCCATCGCCCACCT
AGGCTGAATAGCCATCATCATCATTTACAACCCTAAACTCACTCTCCTAAACTTCTACCTCTACACCATAATAACCGCTG
CAGTATTCCTCACCATAAACTCCACCAACGTCCTAAAACTATCCACACTAATAACAACGTGAACAAAATCCCCCCCACTA
AACATAATACTACTACTAACCCTCCTATCCCTAGCAGGTCTACCCCCACTAACAGGATTCCTACCAAAATGAATAATCGT
CCAAGAACTAACCAAACAAAGCATAGCCCCCACAGCAGCAATCCTATCTATCCTATCCCTTCTAGGCCTGTTCTTCTACC
TCCGCCTAGCATATTGCGCGACAATCACACTCCCCCCTCACACCACCAACCACATAAAACAGTGACGGACCAACAAACCC
ATTAACACTTCAATCGCTATCCTAATAGTAGCATCCACCATAATACTCCCCATCTCCCCCATGATTATCACCACCATCTA
A
Oriolus
ATGAACCCCCAAGCAAAACTAATCTTTATCTTCAGCCTGCTCCTAGGATCTGCCATTACAATCTCGAGCAACCACTGAGT

TATAGCCTGAACTGGACTTGAAATCAACACATTAGCTGTTTTACCCCTAATCTCAAAATCTCACCACCCCCGAGCTATCG
AAGCTGCAACTAAGTACTTCCTGGTACAGGCAGCTGCTTCAACCCTAGTCCTATTCTCCAGCATAACCAACGCATGACAC
ACAGGACAATGAGATATCACACAACTAACACATCCAACATCGTCATTAATCCTAACCGCAGCAATTTCAATAAAACTAGG
GCTGGTACCATTCCACTTCTGATTCCCAGAAGTACTTCAAGGGTCGTCTCTAACCACAGGGCTCCTTTTATCAACAGTTA
TAAAATTTCCACCAATCACCCTACTCTACATAACCTCCTCATCATTAAACCCAACTTTACTCACCACTATAGCCATCCTA
TCTACAGCCTTAGGAGGATGAATAGGGCTGAACCAAACACAAACCCGAAAGATCATGGCTTTCTCCTCAATCTCCCACTT
AGGATGAATGGCCATTATCCTTATCTACTGCCCCAAACTAACCCTACTCAACTTTTTCCTATACACACTAATGACTGCAA
CTGTATTTCTGACCTTCAACTCAATGAAGGCCCTAAAACTATCCACACTAATAACCGCATGGGCAAAAACACCTTCACTT
AGCACAATGCTCCTACTGACCCTGCTGTCACTTGCCGGCCTCCCCCCTCTTACCGGCTTCCTCCCAAAGTGACTCATCAT
CCAAGAACTAACTAAGCAGGACATGATTACGGCAGCAGTGTCTATATCACTGCTCTCTCTACTAGGACTGTTTTTCTACC
TGCGCCTGGCATACTGCGCAACAATTACCCTGCCCCCTCACACTACAAACCACATAAAACAATGACGCAACAACAAACCA
GTAAGCCCCCTGATCGCCGTCCTTACTGCCGTGTCAATTATGCTCCTTCCCATGTCCCCAATAATAGCCTACACCATCTA
A
Dendroica
ATTAACCCTCAAGCAAATCTAATTTTCATCGCCAGCCTGCTCCTAGGAACAACCATTACCATTTCAAGCAACCACTGAGT
CATAGCCTGAACCGGCCTTGAAATCAACACGCTTGCCATCCTCCCCCTAATCTCAAAATCTCATCATCCACGAGCCATTG
AAGCTGCCACTAAATACTTCCTTACACAAGCAGCTGCTTCCGCCCTTGTCTTATTCTCCAGCATAACCAACGCATGACAC
ACCGGACAATGAGACATCACCCAACTCACTCACCCAACATCCTCCTTAATTCTAACCTCAGCAATCGCAATAAAACTAGG
TCTAGTGCCATTCCACTTCTGATTCCCAGAAGTACTCCAAGGCTCCCCCCTGACTACCGGCCTCCTCCTATCAACCATTA
TAAAACTCCCCCCAATTACACTACTCTACATGACTTCCCCATCATTAAACCCCACCCTACTAACCACCCTTGCCATTCTC
TCAACAGCCCTAGGAGGATGAATGGGGCTTAATCAAACACAAGTCCGAAAAATCTTAGCTTTCTCCTCCATCTCCCATCT
AGGCTGAATAGCAATCATCATCATCTATAACCCTAAACTCACTCTCCTTAACTTCTACCTATACGCCATAATAACTGCAA
CCATCTTCCTCACCCTGAATTCAATCAAAGTACTAAAACTATCTACCCTCATAACTGCATGAACCAAAGTTCCAGCCATA
AACGCAATACTACTCCTAACCCTACTCTCCCTTGCAGGCCTACCTCCACTAACAGGTTTCTTACCCAAATGACTTATTAT
TCAAGAATTAACTAAACAAGACATAGCCCCAGCAGCCACACTTATTTCCCTACTCTCCCTACTAAGTCTTTTCTTCTATC
TCCGACTCGCATACTGCACAACCATTACACTCCCACCTCATACTACAAATCACATGAAGCAATGACGTACCAACAAATCA
ACCAGCATCACAATCGCTATCCTGACAACCATATCCGTCATACTTCTCCCCATCTCCCCTATGATTCTTACCATTGTTTA
A
Nectarinia
????????CAAGCAAAACTAGTATTCGCCTCCAGCCTCATTCTCGGAACGACCATCACAATAACAAGCAACCATTGAAT
CCTAGCCTGGACTGGATTGGAAATCAACACATTAGCTATCCTCCCCCTGATCTCAAAATCTCACCATCCACGGGCCATTG
AAGCTGCAACCAAGTACTTTCTGACCCAAGCAGCCGCCTCCGCCCTAGTACTATTCTCCAGCCTAACCAACGCATGACAC
ACAGGACAATGAGATATCACCCAACTAACCCACCCGACATCATGCCTGATTCTCACCTCGGCAATCTCAATAAAATTAGG
ACTAGTCCCCTTCCACTTTTGATTCCCAGAAGTACTCCAAGGCTCTCCCCTAACCACAGGCCTTCTACTATCCACAATCA
TAAAATTACCCCCTCTAACATTACTCTACATAACATCCCCGTCATTAAGCCCAACTATCCTAGTTACCATGGCCATCCTC
TCCGCTGCCATGGGAGGATGAATAGGTCTAAACCAGACACAAATTCGAAAGATCATAGCCTTCTCCTCTATCTCCCATCT
AGGATGAATAGCCATCATCCTCACTTACGACCCTAAACTCACTCTACTAAACTTCTACCTGTACTCGCTAATAACTGCAG
CTGTGTTTCTTACCCTGAACACAATCAAAGTGCTAAAACTCTCTACATTAATGACTGCATGGACCAAGGTCCCCTCACTA
AACGCGATACTATTACTAACCCTGCTCTCCCTAGCAGGCCTTCCCCCTCTAACCGGATTCCTTCCTAAATGACTTATCAT
CCAAGAACTAACTAAACAAGACATAGCCCCAGCGGCAACAGCGATCGCTCTGCTATCCCTACTAAGCCTGTTCTTCTACC
TCCGCCTTGCATACTGTACAACAATCACCCTACCACCCCACACCACAAACCACATAAAACAATGACGCACTAACAAACCA
ACTAACCCTCTAATTGCCATCCTAACAACACTCTCCATCACCCTGCTACCTATTGCCCCCATGATCTACACCTATCTTTA
A
Amytornis_striatus
ATGAACCCCCAAGCAAAACTAATCTTCACCATTAGCCTAATTCTAGGAACAACCATTACAATTTCAAGCAACCACTGAGT
TATGGCCTGAACTGGCCTTGAAATCAACACACTAGCCATTCTCCCCCTAATCTCAAAATCCCATCACCCCCGAGCAATCG
AGGCAGCAACTAAATACTTCCTAGTCCAAGCAGCCGCCTCAACCCTAGTACTATTCTCCAGCATAACCAACGCATGACAC
ACCGGACAGTGAGATATCACCCAGCTAACACACCCAGCCTCATCCACAGTACTAACCACAGCTATTGCAATCAAACTAGG
CTTAGCCCCATTCCACTTTTGATTCCCAGAGGTCCTTCAAGGATCCTCACTAACCACCAGTCTCCTCCTGTCAACAGTCA
TAAAATTCCCCCCCATCACCCTACTCTTCCTAACCGCACACTCACTTAACCCAACACTGCTAACAACCATAGCCATCCTG
TCTGCAGCGCTAGGGGGATGAATAGGACTAAACCAGACACAAATCCGTAAAATCCTAGCCTTCTCCTCCATTTCCCACCT
AGGATGAATAGCCATTATCATCACCTACAACCCTAAACTTACCCTACTAAATTTCTATCTATACAGCCTAATGACAGCAG
CCGTCTTCCTCACCCTCAATACAATAAAAGTCCTAAAACTCTCCACACTAATAACCTCATGAACAAAAATCCCTTCCCTT
AGCGCAATCCTCCTACTTACACTCCTATCTCTCGCTGGCCTCCCGCCCCTCACAGGATTCCTGCCCAAATGACTGATTAT
CCAAGAACTAACTAAACAAGAAATAGCCCCAGCAGCAACAGTCATCTCCCTCCTGTCCCTACTCAGCCTCTTCTTCTACC
TCCGCCTAGCATACTGCACGACCATTACACTCCCTCCCCATACTACTAACCACGTCAAGCAATGACACACTAACAAGCCA
ACTAGCCTCCTAATCGCCCTCCTAACCACTCTCTCCCTCATACTCCTGCCACTCTCCCCCATAATTCTTACCATGGTCTA
?
Pitta
ATGAACCCCCAAGCTAAATTAATTTCCTTATCTAGTCTCGTCCTGGGAACAACCCTTACAATCTCAAGCAACCATTGAAT

CATAGCATGAGCAGGCCTARAAATTAACACCATCGCCATCCTCCCTCTAATCGCTAAACCCCACCACCCTCGAGCAATCG
AAGCTACAACCAAATACTTCCTTGTACAGGCCTCTGCCTCCACATTACTCCTACTCTCCAGCACTATCAACGCATGACTT
ACTGGACAATGAGACATCACCCACCTAACCCACCCCCTGCCCTCTGCACTCCTAACCATAGCAATTGCAATAAAACTAGG
ACTAGTCCCCTTCCACTTCTGATTCCCAGAAGTCCTACAAGGCTCCTCTTTAACTACAGGCCTACTTTTATCTACTGCCT
TAAAATTTCCCCCAGTCACCCTCCTATTACTTACAACTCCCTCCCTCAGCCCAACCCTACTAACCTCGCTAGCCATCCTC
TCAACTGCCCTAGGAGGCTGAATAGGCCTCAACCAAACCCAAATCCGCAAAATCCTTGCCTTCTCCTCCATCTCCCACAT
AGGCTGAATAGTCATTATCATTATCTACACTCCAAAGCTCACCCTACTAACCTTCTATTTATACACCATAATAACCACTA
CCGTCTTCCTCACCATCAGCACAACCAATATCCTAAAACTATCAACAATAATAACCACATGAACAAAAATCCCCCCACTA
ACAGCAACCCTAATACTAACCCTCCTCTCCCTAGCCGGTCTTCCCCCTCTCTCAGGCTTCCTACCCAAATGACTCATTCT
ACAAGAACTCACTAAACAGGAAATAACTACAACAGCCACCATCATCGCTCTCCTCTCTCTACTCAGCCTATTCTTCTACC
TCCGTCTCGCATACTGTGCCACAATCACACTACCCCCTAACTCCTCAAACCACATAAAACTATGACAAACAGACAAAAAA
GTTAACACCACAACCCCAACCCTCACCACTTTAAGCACACTACTCCTACCGCTCTCACCAATAATCCTTACAATCCCCTA
A
Toxorhamphus
??
??
??
??
??
??
??
??
??
??
??
??
??
?
Orthonyx_temminckii
GTGAACCCACGGGCAAAACTAATCTTCATCACTAGCCTGTTCCTAGGTACATCCATCACAATCTCAAGTAGCCACTGAAT
CATAGCCTGAACCGGGCTCGAAATTAACACACTCGCTGTCCTCCCCCTAATCTCAAAATCCCACCACCCCCGAGCCATCG
AAGCCGCTACCAAGTACTTTCTAGTCCAAGCAGCTGCTTCCACCCTAGTACTATTCTCTAGCATGACCAATGCCTGATTC
ACCGGGCAATGGGACATTACCCAACTAACCCACCCCTTGTCCTGCCTCATCCTGACTACAGCAGTCGCAATAAAACTTGG
CCTGGCCCCATTCCATTTCTGATTCCCAGAAGTACTTGAAGGCTCCTCCCTGACCACAGGACTGTTACTGTCCACAGCCA
TGAAGTTACCCCCAACCTCCTTACTCTTCATAACCTCCCAGTCGCTTAACCCCCCTCTACTGACCGGGATGGCCATCCTA
TCAGCAGCCCTAGGCGGATGAATAGGACTGAACCAGACACAAATCCGAAAAATCCTGGCCTTCTCATCCATCTCCCACTT
AGGCTGAATAACTATTATCCTCCTTTACAACCCAAAACTAACCCTTCTTAACTTCTACCTCTATTGCCTGATCACTGCAA
CAGTATTCCTCACCCTCAACACAGCCAAAGCCACAAAACTTCCAACACTACTAACCGCATGAACAAAAACCCCACCCCTA
AGTGCAATGCTCCTACTGACCCTACTCTCCCTAGGCGGCCTCCCCCCTCTGACAGGATTCCTGCCAAAACTATTAATCGT
CCAAGAACTAACCAAACAAGACCTAGCCCCAACTGCAACTCTCATCTCCATGCTCTCCCTACTTAGCCTATTCTTCTACC
TGCGCCTTGCCTACTGCACAGCAATTACTCTCCCCCCTCACACCACAAACCACATGAAACAATGACATGTCAACAAACCC
ACCAACACCCTAATTGCTATCATAACCGTCCTCTCCATCTCTTCCCTTCCCCTCTCACCCATA?????????????????
?
Sericulus_chrysocephalus
ATGAATCCCCAGGCAAAATTAATCTTTGTCATTAGCCTAATTTTAGGGACTACCATTACAATTTCAAGCAACCACTGAGT
TATGGCTTGAACAGGACTAGAAATTAACACACTAGCTATTCTACCACTTATCTCAAAATCTCACCACCCACGAGCCATCG
AAGCTGCAACAAAGTATTTTCTAACCCAAGCAGCTGCCTCGGCCCTAGTACTGTTTTCAAGTATATCCAATGCATGACAA
ACTGGACAATGAGATATCACTCAACTTACCGACCCTATATCATGTACTATTTTTACAGCCGCCATTGCAATCAAATTGGG
CCTTGTACCATTCCACTTTTGATTTCCAGAAGTACTACAAGGTTCTCCCCTAACAACTGGACTAGCCCTAGCCACCATCA
TAAAACTACCCCCCATCACCCTAATATTTATAACCTCTGGATCACTAAATCCAATCATACTAACTACCATAGCCATTATA
TCCACAGCCCTAGGAGGATGAATAGGACTAAACCAAACACAAATCCGCAAAATCCTAGCCTTTTCATCCATCTCACACCT
TGGATGGATGACAATCATTCTTATCTACAACCCAAAACTAACCCTACTGAACTTCTACCTATACACTGTAATAACCGCAG
CCACCTTCCTAACCCTAAACTCTATAAAAGCCCTAAAATTATCAACACTGATAACTTCATGAACAAAATCTCCAAC????
??
??
??
??
?
Cyclarhis_gujanensis
ATGAACCCCCAAGCAAAACTGGTATTCGTACTTAGTCTAATTCTAGGAACCACCCTCACAATTTCCAGCAACCACTGAAT

TATAGCTTGAGCAGGACTTGAAATCAACACACTAGCCATCCTACCATTAATCTCAAAATCCCACCACCCACGGGCTATCG
AAGCCGCAACTAAATACTTTCTAGTACAAGCAGCTGCTTCTACTCTAGTACTATTCTCAGGAATAACCAACGCCCTGCAC
ACAGGACAATGAGACATTACTCAGCTAACCCATCCAACATCCTGCCTTATCCTCACTGCAGCCCTATCAATAAAACTAGG
TCTAGTGCCATTCCACTTCTGATTTCCAGAAGTCCTACAAGGCTCTACACTAACCACAGGACTTCTCCTATCAACAGTCA
TGAAACTGCCCCCTATTACCCTACTATTCATAACCCACCATTCACTAAACCCTGCTCTACTAGCCACTCTAGCCATCATG
TCAGTAGCCCTAGGGGGGTGAATAGGACTAAACCAAACACAAATCCGAAAAATCATGGCCTTCTCATCCATCTCACACCT
AGGATGAATAGCCGTAATCATCATCTACAGCCCTAAACTAACCCTACTAAACTTCTACCTATATGCCATAATAACTGCAT
CTGTATTCATAACCCTCAATACAATCAAAGCCCTGAAACTATCAACCCTAATAACAGTATGGGCAAAAACACCATCACTT
AACATGATTCTCCTACTAGTACTCATATCACTTGCCGGCCTGCCCCCATTAACAGGCTTTCTCCCAAAATGACTAATCAT
CCAAGAACTAACTAAACAGCACATAGCCCCAACAGCAACTATCCTATCCATATTATCACTACTAGGGCTATTCTTCTACC
TCCGACTGGCCTACTGCGCAACAATTACACTACCACCCCACACTACAAACCACATGAAACAGTGACACAACAACAAGCCC
ATCCACCCATCAATTGCCATTCTGGCCACCCTATCTATTGCACTACTGCCCCTCTCCCCAACTATCTCCGCCATTATTTA
A

[ODC]
Polyborus_plancus
TAAATTTGGAGCTACACTTAAGACTAGCAGGCTGCTTCTGGAGCGTGCAAAAGAACTTGACCTTGCCATCATTGGAGTTA
GGTGAG-TTGATAAT--CTCAAAACACAAAATACA------------------GT--CATATTAAA-
TAAAATTTCTTTRAAC------------------------GGTCTACCTGACCATAGAGG----
TGCCTTTGCATATGGTGACATGAGT--TCTGTATAAACTTCTTGAATAACTTGCCAAATAGCAACTGATA-TTGTGTA-
TCTCTTTGTAGTTTCCATGTTGGAAGTGGATGTACGGACCCAGAGACCTTTGTTCAAGCCATTTCTGATGCCCGTTGTGT
GTTTGATATGGGAGTAAGTCT-AGCTCTACTTTCTCTGGAA-CTACTGCTCAACTGTTGTGGCAA-------AAC---
AATCACGTGTACAACTGTTATGGAGTTAAAA--------------GCTAGCTAAAGTCACTAATTTCA---
CRTTGGAATTTTTGGGGTCGT-GAT---
-----------------------------GGCTTGCTTT-GATTGGTTCTKC-AAAGTTCATC-TAC------AGATGC-
ACTAAAATCGGCAGCTCAAACTC-AAGTGAC-TA-------GAAAATGACAAGTTAGCTC-
TGTTCCAAGTATTAGTGAAACTACTTTATT-----GTTTTAGCTTAGAAAGTAATGTAAACTAAGAACTGC---------
------TTTCTTGACAGTGTTGCTGTTAATCTCA-TTCTAGGCTGAACTTGGCTTCAATATGTATCTGCTCGAT
Falco
??????TGGAGCTACACTTAAGACTAGCCGGCTGCTTCTGGAGCGTGCAAAAGAACTTGACCTTGCCATCATTGGAGTTA
GGTGAGTTTGATAAT--GTCAAATTACAAAATACATTAAATATTATCATCTTTAT--CATATTAAA------
TACCTTTAAAT------------------------GGTCTACCTGAC--TAGAGA----
TGCCTTTGCATGTGGAGACTTAAGT--TTGGTATAAACTTCTCAAATAACTTGCCAAATAGCAACTGATA-CAGTGTT-
TCTCTTTGTAGTTTCCATGTTGGAAGTGGATGTACAGACCCAGAGACCTTTGTCCAAGCAATTTCTGATGCCCGTTGTGT
GTTTGATATGGGAGTAAGTCT-AGTTCTACTTTCTCTGGAA-TTACTGCTCAACTGTTGTGGCAA-------AAC---
AATCATGTATACAACTGTTAGGGGGTTAAAA--------------GCTAGCTAAAGTAACTAATTTCA---
AGTTGGAGTTT--GAGGTCAT-GAT---
-----------------------------AGCTTGCATT-GATCAGTTCTGC-AAAGTTCATC-TAC------AGATGC-
ACTGAAATCAGCAGCTCCAAATC-AAGTGGC-TATTAATTTGAAAATGACGAGTTAGCTC-
TGTACCAATTATTAGTGAAATTACTTTATT-----GGTTTAGTGTAGAAAGTAATGTAAACTAAGAACTGC---------
------TTTCTTGACAGTGTTGCTGTTAATTTCA-TTTTAGGCTGAACTTGGCTTCAGTATGTAT?????????
Tyrannus
??????TGGAGCTACACTTAAGACTAGCAGGCTTCTCCTGGAGCGTGCAAAAGAACTTGAGCTTGCCATTGTTGGAGTTA
GGTGAG-TTGATAAT--GTCAAA---------ATA------------------GT--CATATTAAA-
TAAGTTTTCTTTGAAT------------------------AGTCTACCTAAGGACAGAGA----
TGCCTTTACATGTGGTGACATGAGT--TTATACAAGACTTYCTGACTAATTTGSCAAGTAGCAACTGATA-TTTTATA-
TCTTTTCACAGYTTCCATGTTGGAAGTGGATGTACAGACCCAGAGACCTTTGTTCAAGCCATTTCTGATGCCCGCTGTGT
GTTTGATATGGGAGTAAGTCT-AATTCTACTTTCTCTGGAA-CTACTGCTCRMCTGTTGTATTGATCAAGTTGTG-
TTAATCAAGTGTGAATATGTTACAGGGTTAAAAAA------------GCTGACTAA-GTCAATGATTTCA---
TGTTGGAATTTTCGAGGGCAT-GAT---
-----------------------------RGCTTACTTT-GACCAGTTTGGC-AAAACTCATC-TAC------AGATGC-
ACTAAAATTGGCAGCTCATACTC-AAGTGAC-TATT-AATTGAATATGATGAGTTATCTC-
GGCTCCAAATATTAGTGAAATGACTTCACATTAC-CTTTTAAGCTAAAATG----ATAAATTAACAACTGA---------
------TTTCTTGACCCTGTTGCCATTAATCTCA-TTTTAGGCTGAACTTGGCTTYGATATGTAT?????????
Myiarchus
??????TGGAGCTACACTTAAGACTAGCAGGCTTCTCCTGGAGCGTGCAAAAGAACTTGAGCTTGCCATTGTTGGAGTGA
GGTGAG-TTGATAAT--GTCAAA---------ATG------------------GT--CATATTAAA-
TAAGTTTTCTTTGAAT------------------------AGTCTACCTAAGGACAGAGA----
TGCCTTTACATGTGGTGACATGAGT--TTATACAAGACTTCCTGACTAATTTGCCAAGTAGCAAATGATA-TTTTATA-

TCTTTYCACAGTTTCCATGTTGGAAGCGGATGTACAGACCCAGAGACTTTTGTTCAAGCCATTTCTGATGCCCGCTGTGT
GTTTGATATGGGAGTAAGTCT-AATTCTACTTTCTCTGGAA-CTACTGCTCAACTGTTGTGTTGATCAAGTTGTG-
TTAATCAAGTGTGAATATGTTACAGGGTTAAAAAA------------GCTGACTAA-GTCAGTGATTTCA---
TGTTGGGATTTTCGAGGGCAT-GAT---
----------------------------GGCTTACTTT-GACCAGTTTGGC-AAAACTCATC-TAC------AGATGC-
ACTAAAATTGGCAGCTCATACTC-AAGTGAC-TATT-AATTGAATATGATAAGTTATCTC-
TGCTCCAAATATTAGTGAAATGATTTCACATTAC-CTTTTAAGCTAAAATG----ATAAATTAACAACTGA---------
------TTTCTTGACCATGTTGCCATTCATCTCA-TTTTAGGCTGAACTTGGCTTCAATATGTAT?????????
Hypocnemis_cantator
??
??
??
??
??
??
??
??
??
??
??
?????????????
Phlegopsis
??
??
??
??
??
??
??
??
??
??
??
?????????????
Manacus_manacus
??????TGGAGCTACATTGAAGACTAGCAGGCTTCTTCTGGAACGTGCAAAAGAACTTGAGCTTGCTATTGTTGGAGTTA
GGTGAG-TTGATAAT--GTCAAA---------ATA------------------GT--CATATTAAA-
GAAGTTTTCTTTAAAT------------------------AGTCTACCTAAGGACAGAGA----
TGCCTTTGCATGTGGTGACACGAGT--TTATACAAGACTTCTTGACTAATTTGCCAAGTAGCAACTGATA-TTTTTA-
TCTTTTCACAGTTTCCATGTTGGAAGTGGATGTACAGACCCAGAGACCTTTGTTCAAGCCATTTCTGATGCCCGCTGTGT
GTTTGATATGGGAGTAAGTCT-AATTCTACTTTCTCTGGAA-TTACTGATCAACTGTTGTGGCAA-------AAG-
TTAATCAAGTGTGAATGTGTTACGGGGTTAAAAAA------------GCTGGCTAA-GTCACTGATTTCA---
TGTTGGAATTTTTGAGGGCTT-GAT---
----------------------------GGCTTACTTT-GACCAATTTGGT-AAAACTCATC-TAC------AGATGC-
ACTAAAATTGGCAGCTCCTACTG-AAGTGAC-TATT-ACTTGAATATGATGAGTTATCTC-
TGTTCCAAATATTAATGAAATGACTTCACATTAC-CTTTTAAGCTAAAATG----ATAAATTAACAACTGA---------
------TTTCTTGACCATGTTGCCAATAATCTCA-TTTTAGGCTGAACTTGGCTTCAATATGTAT?????????
Acanthisitta_chloris
??GCGGGCAAAAGAACTTGACCTTGCCATTGTGGGAGTTA
GGTGAG-TGGATAAC-TTTTTTTGT-------ATG------------------AC--TGTATTTTGGTA--
TTTTCTCTAAAT------------------------ACTCTACTTGACAATAGAGG----
TGCCTTTGCATCTGGTGACATGACT--TTATACAAAACTTCTTGACTAATTTGCCAAATAGCAACTGATA-TTTTCTA-
TCTTTTTGTAGTTTCCATGTTGGAAGTGGATGTACAGACCCGGAGACCTTTGTTCAGGCCATT?????????????????
?????ATATGGGAGTAAGTCTGAATTCTGCTTTCTCTGGAA-GTACTGCCAGACTGTTGTAGCAC-------AAC-
TGAATGAAGAGTAAAACTGTTATGGGGTTAAAA--------------GCTAGCTAA-
GTCACTGATTTCATGTTGTTGGAATTT--GAGGTCAT-GAT---------------------------------------
---GACTCCTTT-GGCCAGTTTGGC-AAAA--CATC-
TAT------GGATGC-GCTAAAATTGGCAGCTCAAACTC-AAGTGAC-TATT-ACTTGAACA--CTGAGTTAGCTC-
TGTTCTAAGTAT--ATGAAATGACTTCACATTAC-TTTTTAAGCTAAAATG----ATAAATTAAGAACTGG---------
------TTTGTTGACAGTGTTGCCATT-ATCTCA-TTTT???????????????????????????????????

Regulus
TAAATTTGGAGCTACACTTAAGACTAGCAGGCTTCTTCTGGAGCGTGCAAAAGAACTTGATCTTGCCATCGTTGGAGTTA
GGTGAG-CTGACAGT--ATCAAAAC-------ACA------------------GT--CATATTAAA-
TAAGTTTTTTTAAAT-------------------------GGTCTGCCTGATAATAGAGG----
TGCCTTTGCATGTGATGACCTGAGT--TTGTA-GAAACTTCTTGACTAATTTGCCAAATAGCGACTGATG-TTTTGTT-
TCTTTTTGTAGTTTCCATGTAGGAAGTGGATGTACAGACCCAGAGACCTTTGTTCAGGCCATTTCTGATGCCCGCTGTGT
GTTTGATATGGGAGTAAGTCT-AGTTCTACTTTTTCTGGAA-CTACTGTTCAACTGTGGTGGGAA-------AAC-
TGAACGAGATGAACAACTGTTATGGGGTTAAAG--------------GC-AGCTRT-
GTCACTGACTTCATGTTGCTGGAAATTTTTAGGTTCT-GGT---------------------------------------
--AGCTCACTGT-GACCAGCTTGGC-AAAACTCGTC-
TAC------AGATGT-GCTAAAATTTGCAATTGAAACTT-AAATAAC-TGTT-ACTTGAAC---GTGTGTTAGCTC-
TGTTTCAAGTGTAAATGAAATAACTTCACATTAC-TTCTTAAGCTAAAATG----
ATGAATTATAAACTGATATAAGTTATAAACTTTTCTTGACAGTGTGGCTGTTAATCTCA-
TTTTAGGCTGAACTTGGCTTCGATATGTATCTGCTTGAT
Ailuroedus
??
??
??
??
??
??
??CT-GGT----------------------------------
---GTCTTACTTT-GACCAGTTTGGC-
AAAACTCATC-TAC------AGATGC-ACTAAAATTGGCAGCTCAAATGC-AAATGAC-??TT-
ACTTGAACATGATGAGTTAGCTG-CGTTCCAAGTATTAATGAAA??????????TTGC-CTTTTAAGCTAAGATG----
GTAAATTATAAAT???
?????????????????????????????????????
Zosterops
TAAATTTGGAGCTACGCTTAAGACTAGCAGGCTTCTTCTGGAGCGTGCAAAAGAACTTGACCTTGCCATCGTTGGAGTTA
GGTGAG-CTGACAGT--ATCAAAAT-------ACA------------------GT--TGTATTAAA-
TAAGCTTTCTTTAAAT------------------------GCTTTACCTGACAATAGAGA----
TGGCTTTGCTTGTGATGACATGAGT--TTATATAAAACTTCTTGATTAATTTGCCAAGTATCAACTGATA-TTTTGTA-
TCTTTTTGTAGTTTCCATGTTGGAAGTGGATGTACAGACCCAGAGACCTTTGTTCAAGCCATTTCTGATGCCCGCTGTGT
GTTTGATATGGGAGTAAGTCT-AGTTCTACTTTATCTGGAA-CTACTTCTCAGCTGTTGTGGCAA-------AAC-
TGAATGAAGTGTACAACTGTTATGGGGTTAAAA------------------GCTAA-
GTCACTGACTTCATGTTGTTGGGATTTTTTAGGTTCT-GGT---------------------------------------
--AGCTTACTTT-GACCAGCTTGGC-AAAACTCATC-
TAC------AGATGC-ACTAAAATTGGCAGTTCAAACTC-AAATAAC-TGTT-ACTTGAAC---ATGTGTTAGCTC-
TGTTTCAAGTGTTAATGAAATTAATTTACATTAC-TTTTTAAGCTAAAACA----
ATAAATTATGAACTGATATAAGTTATAAAC-TTTTCTGACAGTGTTCCTGTTAATCTCA-
TTTTAGGCTGAACTTGGCTTCAATATGTATCTGCTTGAT
Onychorhynchus
??????TGGAGCTACACTTAAGACTAGCAGGCTTCTCCTGGAGCGTGCAAAAGAACTTGAGCTTGCCATTGTTGGAGTTA
GGTGAG-TTAATAT--GTCAAA----------ATA------------------GT-------CAAG-
TAAGTTTTCTTTGAT-------------------------AGTCTACCTAAGGACAGAGA----TGCCTTTGC-------
---ATGAGT--TTATACAAGAGTTCTTGACCAATTTGCCTAGTAGCAACTGATA-TTTTGTA-
TCTTTTCACAGTTTCCATGTTGGAAGTGGATGTACGGACCCAGAGACATTTGTTCAAGCCATTTCTGATGCCCGCTGTGT
GTTTGATATGGGAGTAAGGCT-AATTCTTCTTTCTCTGGAA-CTACTGCTCAACTGTTGTGTTGATCAAGTTGTG-
TTAATCAAGTGTGAATATGTTACGGGGTTAAAA----------------AAACTAA-GTCAATGATTTCA---
TGTTGGAATTTTTGAGGGCAT-GAT---
----------------------------GGCTTTCTTT-GACCAGTTTGGT-AAAACTCGTA-TAC------AGATGC-
ACTGAAATTGGTAGCTTGTACTC-AAGTGAC-TATC-ACTTGAATGTGATGAGTTAGCTC-
TGCTCCAAGTATTAGTGAAATAACTTTACATTAC-CTTTTAAGCTAAAATG----ATAAATTAACAGCTGA---------
------TTTCTTGACCGTGTTGCCATTAATTTCA-TTTTAGGCTGAACTTGGCTTCAGTATGTAT?????????
Nestor_notabilis
??
??
??
??
??

??
??
??
??
??
??
?????????????
Leiothrix_argentauris
TAAATTTGGAGCTACACTTAAGACTAGCAGGCTTCTTCTGGAGCGTGCAAAAGAACTTGACCTTGCCATTGTTGGAGTTA
GGTGAG-CTGACAGT--ATCAAAAC-------ACA------------------GT--TGTATTAAA-
TAAGTTTTCTTTATAT------------------------GCTCTACCTGGCAATAGAGA----
TGCCTTTGCATGTGRTGACATGAGR--TTTGTGTAAACTTCTTGATTAATTTGCCAAATATTAACTGATA-TTTTGTA-
TTTTTTTGTAGTTTCCATGTTGGAAGTGGATGTACAGACCCAGAGACCTTTGTCCAAGCCATTTCTGATGCCCGCTGTGT
GTTTGATATGGGAGTAAGTCT-AGTTCTACTTTATCTGGAA-CTATGGCTCAACTGTTGTGGCAA-------AAC-
TGAATGAAGTGTATAACTGTTATGGGGTTAAAA--------------GCTAGCTAA-
GTCACTGACTTCGTGTTGTTGGAATTTTTTAGGTTCT-AGT---------------------------------------
--AGCTTACTTT-GACCAGTTTGGC-AAAACTCATC-
TAC------AGATGC-ACTAAAATTGGCAGCTCAAACTC-AAATRAC-TGTT-ACTTGAAC---ATGTGCTAGCTC-
TGTTTCAGGTGTTAATGAAATGACCTTACATTAC-GTTTTAAGCTAAAATG----
ATAAATTATCAACTGATATAAATTACAAACTTTTCTTGACAGTGTTGCTGTTAATCTCA-
TTTTAGGCTGAACTTGGCTTCAATATGTATCTGCTTGAT
Troglodytes
??????TGGAGCTACACTTAAGACTAGCAGGCTTCTTCTGGAGCGTGCTAAAGAACTTGACCTTGCCATCGTTGGAGTTA
GGTGAG-CTGACAGT--ATCAAAAC-------ACA------------------GT--CATATTAAA-
TAGGTTTTCTTTAAAT------------------------TGTTTGCCTGGCAATAGAGA----
TGCTTTTGCATGTGATGATATGAGT--TTATACAAGACTTCTTGACTAATTTGCCAAATAGCAACTGATA-TTTTGTA-
TCTTTTTGTAGTTTCCATGTTGGAAGTGGATGTACAGACCCAGAGACCTTTGTTCAAGCCATTTCTGATGCCCGCTGTGT
GTTTGATATGGGAGTAAGTCT-AGTTCTACTTTTTCTGGAA-CTGCTGCTCAACTGTGGTGGCAA-------AAC-
TGAATGAAGTGTACAACTGTTGTGGGGTTAAAA--------------GCTAGC----ATCGCTGACTTGA---
TGTTGGAATTTTTTAGGTCCT-GGT---
----------------------------AATTTACTTA-GG-CAGCTTGGC-AAAACTCATC-TAC------AGATGC-
ACTAAAATTGGCAGCTCAAACTC-AAGTGAC-TGTT-ATTTGAAT---ATGTGTTAGCTA-AG-----
AGTTTTAATTAAATGACTTCAGGTTACTTTTTTATGCTAAAATG----
ATAAATTATAAGCTGATATAAATGATAAACTTTTCTTGACAGTGTTGCTGTTAATCTTA-
TTTTAGGCTGAACTTGGCTTCGATATGTAT?????????
Luscinia_svecica
TAAATTTGGAGCTACACTTAAGACTAGCAGGCTTCTTCTGGAGCGTGCAAAAGAACTTGACCTTGCCATTGTTGGAGTTA
GGTGAG-CTGACAGT--ACCAAAAC-------ACA------------------GTTTTATATTAAA-
TAAGTTTTCTTTAAT-------------------------GGTCTAGCTGACAATAGAGA----
TGCTTTTGCATGTGATGACATGAGT--TTATACAAAATTTCTTGACTAATTTGCCAAATAGCAAGTGATA-TTTTGTA-
TCTTTTTGTAGTTTCCATGTTGGAAGTGGATGTACAGACCCAGAGACCTTTGTCCAAGCCATTTCTGATGCCCGCTGTGT
GTTTGATATGGGAGTAAGTCT-AGTTCTGTTTTCTCTGGAA-CTACTGCTCAACTGTTGTGGCAA-------AAC-
TGAATGAAGTGTACAACTGTTATGGGGCAAAAA--------------GCTAGCTAA-GTCACTGACTTAG------
TGGAATTTTTTGGGTGCT-GGT--
-------------------------AGCTTACTTT-GACCAGCTTGGC-AAAACTCATC-TACTCATTTAGATGC-
ACTAAAACTGGTAGCTCAGTCTC-AAAAAAC-TGTT-ACTTGAAC---ATGTGTTAGCTC-
TGTTTCAAGTATTAATGAAATGAATTTACATTAC-TTTTTAAGCTAAAATG----ATAAATTATAAACT-----------
------TTTTTTGACAGTGTTGCTGTTAATCTCA-TTTTAGGCTGAACTTGGCTTCAATATGTATCTGCTTGAT
Psittacus_erithacus
??????TGGAGCTACGCTTAAGACTAGCAGGCTTCTTCTGGAGCGTGCAAAAGAGCTTGACCTTGCCATCATTGGAGTAA
GGTGCA-TTGATAG---ATCACTAT-------GCA------------------GT--CATAGTAAA-
TAAGATTTATTTAAGY------------------------GGTATACCTGAC---AGAGG----
TGACTCTGCATATGATGACTGGAGT--TTTGAATGAACTTCTTTACAAATTTGTCAAATAACAACTGATA-TTTTGTA-
TCTCTTCATAGTTTCCACGTTGGAAGTGGATGTACAGACCCAGAGACCTTTGTCCAAGCCATTTCTGATGCCCGCTGTGT
GTTTGATATGGGAGTAAGTCC-AGTTCTACTTTCTCTGGAA-CTACTGATCAACTGCTGTGGCAA-------
AACCTGAATCAAGTGTAAAACTGTTGTGGGGTTAAAA--------------GCTAGCCAA-
GCTGCTGATTTCATTTGTTGGAATTTTTGAGGTCAT-GAG--
--GGCTTACTTA-GATCAGTTCTGC-AAACCTCATT-
TAC------AAATGT-GCTAAAATCAGCAGCTCAAACTC-AAGTGACTTATTAACTTGAACACGATGAATTAGTTG-

TGTTCCAAGTATTAATGAAATGGCTTTACATCAC-TTTTTAAGCTACAATG----ATAAACTAAGAGCTGC---------
------TTTTGTGACAGTGTTGCTGTTAATCTCA-TTTTAGGCTGAACTTGGCTTCAATATGTAT?????????
Ficedula_hypoleuca
TAAATTTGGAGCTACACTTAAGACTAGCAGGCTTCTTCTGGAGCGTGCAAAAGAACTTGACCTTGCCATTGTTGGAGTTA
GGTGAG-CTGACAGT--ACCAAAAC-------ACA------------------GTTGTATATTAGA-
TAAGTTTTCTTTAAT-------------------------GGTCTAGCTGACAATGGGGA----
TGYTTTTGCATGTGATGACATGAGT--TTATACAAAATTTCTTGACTAATTTGCCGAATAGCAAGTGATA-TTTTGTA-
TCTTTTTGTAGTTTCCATGTTGGAAGTGGATGTACAGACCCAGAGACCTTTGTCCAAGCCATTTCTGATGCCCGCTGTGT
GTTCGATATGGGAGTAAGTCT-GGTTCTACTTTCTCTGGAG-CTGCTGCTCAATTGTTGTGGCAA-------AAC-
TGAATGAAGTGTACAACTGTTATGGGGCAAAAA------------------GCTAA-GTCACTGACTTAG------
TGGAATTTTTTGGGTGCT-GGT--
-------------------------AGCGTACTTT-GATCACCTTGGC-AAAACGCATC-TACTCATTTAGATGC-
AYTAAAATTGGTAGCTCAGTCTC-AAAAAAC-TGTT-ACTTGAGC---ATGTGTTAGCTC-
TGTTTCAAGTATTAATGAAATGAATTTACATTAC-TTTTTAAGCTGAAATG----ATAAATTATAAACT-----------
------TTTTTTGACAGTGTTGCTGTTAATCTCA-TTWTAGGCTGAACTTGGCTTCAATATGTATCTGCTTGAT
Menura_novaehollandiae
??GCGTGCAAAAGAACTTGACCTTGCCATTGTTGGAGTTA
GGTGAG-CTGACAAT--ATCAAAAC-------ACA------------------GT--CATATTAAA-
TAATTTTTCTTTAAAT------------------------GGTCTGCCTTATGGTAGAGA----
TGCCTTTGCATGTGGTTACATGAGT--TTATACAAAACTTCTTTACTAATTTGCCTAATAGCAGCTGATA-GTTCGTG-
TCTTTTTGCAGTTTCCATGTTGGAAGTGGATGTACAGACCCAGAGACTTTTGTTCAAGCCATTTCTGATGCCCGCTGTGT
GTTCGATATGGGAGTAAGTCT-AGTTCTACTTTCTCTGGAA-CTACTGCTCAGCTGTTGTGGGAA-------AAC-
TGAATGAAGTGTACAACTGTTAGAGGGTTAAAA--------------GCTAGCTAA-
GTTGCTGATTTCATGTGGTTGGAATTTTTGAGGTCCT-GAT---------------------------------------
--GACTTACTTT-GACCAGTTTGGC-AAAACTCATC-
TGC------AGATGC-ACTAAAATTGGCAGCTCAAACTC-AAACGAC-TGTT-AGTTGAACACAATGAATTAGTGG-
TGCTCCAAGTATTAATTAAATGACTTCACATTAC-CTTTTAAGCTTAAATG----ATAAATTATAAACTAA---------
------TTTCTTGACAGTGTTGCTGTTACTCTCA-TTTT???????????????????????????????????
Pycnonotus
TAAATTTGGAGCCACACTTAAGACTAGCAGGCTTCTTCTGGAGCGTGCAAAGGAACTTGACCTTGCCATTGTTGGAGTTA
GGTGAG-CTGACAGT--ATCAAAAC--
AGTCTTCTTTAAAT------------------------GGTCTGCCTGACAGTAGAGA----
TGCCTTTGCATGTGATGACATGAGT--TTATATAAAACTTCTTAACTAATTTGCCAAATAGCAACTGACA-TTTTCTA-
TCTTTCTGTAGTTTCCATGTTGGAAGTGGCTGTACAGACCCAGAGACCTTTGTTCAAGCCATTTCTGATGCCCGCTGTGT
GTTTGATATGGGAGTAAGTTT-AGTTCTGCTTTATTTGGAA-CTACTGCTCAACTGTTGTGGTAA-------AAC-
TGAAYGAGGTGTACAATTGTTATGGAGTTAAAA--------------GCTAGCGAA-
GTCACTGAATTCATGTTGTTGGAATTTTTTAGGTCCTTGTT---------------------------------------
--AGCTTACTTT-GACCAGCTTGGCAAAAACTCATC-
TAC------AGATGC-ACTAGAATTGGCAGCTCAAACTC-AAATGAC-TGTT-ACTTGAAC---ATGTGTTAGCTC-
TGTTTCAAGTGTTAATGAAATGACTTTACATTAC-TTTTTAAGCTAAAATG----
ATAAATTATGAACTGATGTAAATTATAAACTTTTCTTGACAGTGTTGCTGTTAATCTCA-
TTTTAGGCTGAACTTGGCTTCAATATGTATCTGCTTGAT
Donacobius_atricapilla
TAAATTTGGAGCTACACTTAAGACCAGCAGGCTTCTTCTGGAGCGTGCAAAAGAACTTGACCTTGCCATTGTTGGAGTTA
GGTGAG-CTGACAGT--ATCAAAAC-------GCA------------------GT--TGTACTAAA-
TAAGTTTTCTTTAAAT------------------------GGTCT----------AGAGA----
TACCTTTGCTTGTGGTGACATGAGT--TTATACAAAACTTCTTGAGTAATTTGTCAAATAGCAACTGATA-TTTTGTA-
TCTTTTGGTAGCTTCCATGTTGGAAGTGGATGTACAGACCCAGAGACCTTTGTTCAAGCCATTTCTGATGCCCGCTGTGT
GTTTGATATGGGAGTAAGTCT-AGTTCTACTTTCTCTGGAA-CTACTGTTCAACTGTTGTGGCAA-------AAC-
TGAATGACGTGTACAACTGTTAAGGGATTAAAA--------------GCTAGCTAA-
GTCACTGACTTCGTGTTGTTGGAATTTTTTAGGTCCT-GGT---------------------------------------
--AGCTTACTTA-GACCAGCTTGGC-AAAACTCATC-
TAT------AGATGC-RCTAAAATTGGCAGCTCAAACTC-AAATGAC-TGTT-ACCTGAAC---ATGTGTTAGCTC-
TGTTTCAAGTGTTAATGAAATGACTGTACATTAC-TGTTTAAGCTAAAATG----
ATAAATTATGAGCTGATATAATATATAAACGTTTCTTGACAATGTTGCTGTTAATCTCA-
TTTTAGGCTGAACTTGGCTTCAATATGTATCTGCTTGAT
Hirundo_rustica
TAAATTTGGAGCTACACTKAAGACTAGCAGACTTCTTCTGGAGCGTGCAAAAGAGCTTGACCTTGCCATTGTTGGAGTTA
GGTAAG-CTGACAAT--ATCAAAAC-------ACA------------------GT--YGTATTAAA-T--
GTTTTCTTTAAAT------------------------

GGTCTACCTGACAATAGAGAGAGGTGCCTTTACACATGATGACATGGG---
TTATACAAAACTTCTCGACTACTTTGCCAAATCGCAACTGATA-TTTTGTA-
TCCTTTTGTAGTTTCCATGTTGGCAGTGGATGTACAGACCCAGAGACGTTTGTTCAAGCCATTTCTGATGCCCGCTGTGT
GTTTGATATGGGAGTAAGTCT-AGTTCTATTTTCTCTGGAA-CTGCTGCTCAACTGTTGTGGCAA-------AAC-
TGAGTAAAATGTACAACTGTTAYGGGGTTAAAA--------------GCTAGCTAA-GTCACTGACTTCA---
TGTTGGAATTTTTTAGGTCCT-GGT---
----------------------------AGCTTACTTTGGACCAACTTGGC-AAAACTTGTC-TAT------AGATGC-
ASTAAAATTGACAGCTTAAACTC-AAATGAC-TGTT-ACTTRAAC-----GTCTTAGCTC-
TGTTTCAAGTATTAATGAGGTGACTTTACATTAC-TTTTTAGGCTAAAATG----
GTAAATTATGAATTGATATAAATTATGAGCTTTTCTTGACAGTGTTGCTGTTAATCTCA-
TTTTAGGCTGAACTTGGCTTCAGTATGTATCTGCTGGAT
Lichenostomus
??
??
??
??
??
??
??
??
??
??
??
?????????????
Cnemophilus
??GCGTGCGAAAGAACTTGACCTTGCCATTGTTGGAGTTA
GGTGAG-CTGATGAT--ATCAAAAC-------ACA------------------GT--CGTATTAAA-
TAAGTTTTCTTTAAAT------------------------GGTGTACCTGAAAATAGAGA----
TGCCTTTGCATGTGATGACATGAGT--TTATACAAAACTTCTTGACTAATTTGCCGCATAGCAACTGATA-GTTTGTA-
TCTTTTTGTAGTTTCCACGTTGGAAGTGGATGTACAGACCCAGAGACCTTTGTTCAAGCCATT?????????????????
?????ATATGGGAGTAAGTCT-AGTTCTACTTTTTCTGGAC-CTGCTGCTCAGCTGTTGTGGCAA-------AAC-
TGAATGAAGTGTACACCTTTTACGGCATTAAAA--------------
GCTATCTAAGGTCACTGACTTCGTGTGGTTGGAATTTTTGAGGTCCT-GGT-----------------------------
--AGCTTACTTT-GACGAGCTTGGC-
AAAACTCATC-TAC------AGATGC-TCTAAAAGTGGCAGCTCAAACTC-AAATGAC-TATC-ACTTGAAC---
ATGAGTTAGCTC-TGTTTCAAGTGTTAATGAAATTACTTCACATTAC-ATTTTATGCWAAAATG----
ATAAATTATAAACTGATATAAATTGTAAACTTTTCTTGACAGTGTTGCTGTTAATCTCA-
TTTT???????????????????????????????????
Coracina
??GCGTGCGAAAGAACTTGAGCTTGCCATTGTTGGAGTTA
GGTGAG-CTGACAGT--ATCAAAAC-------ACA------------------GT--CACGTTAAA-
TGAGTTTTCTTTAACT------------------------GGTCTACCTGACAATAGAGA----
TGCCTTTGCATGTGGTGACGTGAGT--TAACACAAAACTTCTTGACTAATTTGCCAAATAGCAACTGATA-GTTTGTA-
TCTTTTTATAGTTTCCATGTTGGAAGTGGATGCACAGACCCAGAGACCTTTGTTCAAGCCATTTCTGATGCCCGCTGTGT
GTTTGATATGGGAGTAAGTCT-AGTTCTACTTTCTCTGGAA-CTACTGCTCAACTGTTGTGGCAA-------AAC-
TGAATGAAGTGTACAACTTCTATGGGGTTAAAA--------------GCTAACTAA-
GTCACTGACTTCATGTTGTTGGAATTTTTG--------AGT---------------------------------------
--AGCTTACTTT-GACCAGCTTGGC-AAAACTCATC-
TAC------AGATGC-ACTAAAATTGGCAGCTCAAACTC-AGATGAC-TATT-ACTTGAAC---ATGAGTTAGCTC-
TGTTTCAAGTGTTAATGAAATTACTTAACATTAC-TTTTTAAGCTAAAATG----ATAAGTTATAAACTGG---------
------TTTCTTGACAGTGTTGCTGTTAATCTCA-TTTT???????????????????????????????????
Dicrurus
??GCGTGCAAAAGAACTTGACCTTGCCATTGTTGGAGTTA
GGTGAG-CTGACAGT--ATCAAAGC-------ACA------------------GT--CATATTAAA-
TAAGTTTTTTATAAGC------------------------GGTCTATCTGATAATAGAGA----
TGCCTTTGCTTGTGGTGGCGTGAGT--TTATACAAAACTTCTTGACTAATTTGCCAAATAGCAACAGATA-ATTTGTA-
TCTTTTTGTAGTTTCCATGTTGGAAGTGGATGTACAGACCCAGAGACCTTTGTTCAAGCCATTTCTGATGCCCGCTGTGT
GTTTGATATGGGAGTAAGTCT-AGTTCTACTTTTTCTGGAA-CTACTGCTCAACTGTTGTGGCAA-------AGC-
TGAATGAAGTGTACAACTTTTATGGGGTTAAAA--------------GCTAGCTAA-
GTCACTGACTTCATGTTGTTGGAACTTTTGAGGTCCT-GGT---------------------------------------

--AGCTTACTTT-GACCACCTTGGC-AAAACTCATC-
GAC------TGATGC-TCTAAAATTGGCAACTCAAATTC-AAATGAC-TGTT-ACTTGAAC---ATGAGTTAGCTC-
TGTTTCAAGTGTTAATGAAATGACTTAACATTAC-TTTTTAAGCTAAAATG----ATAAATTATAAACTGG---------
------TTTTTTGACAGTGTTGCTGTTAATCTCA-TTTT???????????????????????????????????
Gerygone_fusca
??
??
??
??
??
??
??
??
??
??
??
?????????????
Ptiloris_magnificus
??GCGTGCAAAAGAACTTGACCTTGCCATTGTTGGAGTTA
GGTGAG-CTGACAGT--ATCAAAAC-------ACA------------------GT--CATATTAAA-TA--
TTTTTTATAAGT------------------------GGTCTACCTGACAATAGAGA----
TGCCTTTGCTTGTGGTGGCATGAAT--TTATACAAAACTTC-TGACTAATTTGCGAAATAGCAACTTATA-ATTTATA-
TCTTTTTGTAGTTTCCATGTTGGAAGTGGATGCACAGACCCAGAGACCTTTGTTCAAGCCATT?????????????????
?????ATATGGGAGTAAGTCT-AATTCTGCTTTTTCTGGAA-CTGCTGCTCAACTGTTGTGGCAA-------AGC-
TGAATGAAGTGTACAATTTTTATGGGATTAAAAGCTAAGCTTTAGTGACTAGCTAA-
GTCACTGACTTCGTGTTGTTGGAATTTT----GTCCT-GGT---------------------------------------
--AGCTTACTTT-GACCAGCTTGGC-AAAATTCACC-
TAC------TGAGGC-ACTAAAATTGGCAACTCAAACTC-AAATGAC-TGTT-ACTTGAAC---ATGAGTTACCTC-
TGTTTCAAGTGTTAATGAAATGACTCAACATTAC-TTTTTAAGCTAAAATG----ATAAATTATAAACTGA---------
------TTTTTTGACAGTGTTGCTGTTAATCTCA-TTTT???????????????????????????????????
Paradisaea_raggiana
??GCGTGCAAAAGAACTTGACCTTGCCATTGTTGGAGTTA
GGTGAG-CTGACAGT--GTCAAAAC-------ACA------------------GT--CATATTAAA-TA--
TTTTTTATAAGT------------------------GGTCTACTTGACAATAGAGA----
TGCCTTTGCTTGTGGTGGCATGAAT--TTATACAAAACTTC-TGACTAATTTGCGAAATAGCAACTGATA-ATTTGTA-
TCTTTTTGTAGTTTCCATGTTGGAAGTGGATGCACAGACCCAGAGACCTTTGTTCAAGCCATTTCTGATGCCCGCTGTGT
GTTCGATATGGGAGTAAGTCT-AATTCTGCTTTTTCTGGAG-CTGCTGCTCAACTGTTGTGGCAA-------AGC-
TGAATGAAGTGTACAATTTTTATGGGATTAAAAACTAAACTTTAGTGACTAGCTAA-
GTCACTGACTTCGTGTTGTTGGAAYTTT----GTCCT-GAT---------------------------------------
--AGCTTACTTT-GACCAGCTTGGC-AAAACTCACC-
TAC------TGATGC-ACTAAAATTGGCAACTCAAACTC-AAATGAC-TGTT-ACTTGAGC---ATGAGTTACCTC-
TGTTTCAAGTGTTAATGAAATGACTCAACATTAC-TTTTTAAGCTAAAATG----ATAAATTATAAACTGA---------
------TTTTTTGACAGTGTTGCTGTTAATCTCA-TTTT???????????????????????????????????
Cyanocorax_chrysops
??
??
??
??
??
??
??
??
??
??
??
?????????????
Lonchura
??
??
??

??
??
??
??
??
??
??
??
?????????????
Phylloscopus
??????????????????????????GCAGGCTTCTTCTGGAGCGTGCAAAAGAGCTTGACCTTGCCATTGTTGGAGTTA
GGTGAG-CTGACAGT--CTCAAA---------ACA------------------GT--TGTATTAAA-
TAAGTTTTCTTTAAAT------------------------GCTCTGCCTGACAATACAGA----
TGCCTTTGCATGTGTTGGCATTTGT--TTATACAAAATTTCTTGATTAATTTGCCAAATGTCRCCTGATA-TGTTGTA-
TCTTTTTGTAGTTTCCATGTTGGAAGTGGATGTACAGACCCAGAGACCTTTGTTCAAGCCATTTCTGATGCCCGCTGTGT
GTTTGATATGGGAGTAAGTCT-AGTTCTACTTTATCTGGAACCTACTGCTCAACTGTTGTGGCAA-------AAC-
TGAATGAAGTGTACGACTGCTATGGGGTTAAA---------------RCTAGCTAA-
GTCACTGACTTCATGTTGTTGGCATTTTTTATGTCCT-GGT---------------------------------------
--AGCTTACTTT-GACCAGCTTGGC-AAAATTCATC-
TAC------AGATGC-ACTAAAATGGGCAGCACAAACTC-AAATGAC-TGTT-ACTTGAAC---ATGTGTTAGCTC-
TGTTTYAAGTGTT----AAATGACTTTACAATAG-TTTTTAAGCTAAAATG----
ACAAATTATGTACTGATATAAATTATAAATTTTTCTTGACAGTGTTCCTCTTAATCTCA-
TTTTAGGCTGAACTCGGCTTCAATATGTATCTGCTTGAT
Turdus
TAAATTTGGAGCTACACTTAAGACTAGCAGGCTTCTTCTGGAGCGTGCAAAAGAGCTTGACCTTGCTATTGTTGGAGTTA
GGTGAG-CTGACAGT--ACCAAAAC-------ACA------------------GT--TGTATTAAA-
TAAATTTTCATTGAT-------------------------GGTCTAGCTGACAATAGAGA----
TGCGTTTGCATGTGATGACATGAGT----ATACAAAACTTCTTGACTAATTTGCCAAATATCAACTGATATTTTTGTA-
TCTTATTGTAGTTTCCACGTTGGAAGTGGATGTACAGACCCAGAGACCTTTGTTCAAGCCATTTCTGATGCCCGCTGTGT
GTTTGATATGGGAGTAAGTCT-AGTTCTACTTTCTCTGGAG-CTACTGCTCAACCGTTGTGGCAA-------AAC-
TGAATGAAGTGTACAACTGCTATGGGGTTAAAA--------------GCTAGCTAA---
AGCTGAGTTCATGCTGTTGGAATTTTTTAGGTTCT-GGT---
--AGCTTACTTT-GACCAGCTTGGC-AAAACTCATG-
TAC------AGATGC-ACAAAAACTGGCAGCTTAAACTC-AAAAAAC-TGTT-CCTTGAAC---ATGTGTTAGCTC-
TGTTTCAATTGTTAATGAAATGACTTCACATTAC-TTTTTAAGCTAAAATG----ATAAATTATGAACT-----------
------TTTTTTGACAGTGTTGCTGTTAATCTCA-TTTTAGGCTGAACTTGGCTTCAATATGTACCTGCTTGAT
Acrocephalus
??????????????????????ACTAGCAGGCTTCTTCTGGAGCGTGCAAAAGAACTTGACCTTGCCATTGTTGGAGTTA
GGTGAG-CTGACAGT--ATCAAA---------ACA------------------GT--
TGTATTAAATTAAGTTTTCTTTAAAT------------------------GGTCTACCTGACCACAGAGA----
TGCCTTTGCATGTGATGACATGAGT--TTATACAAAACTTCTTGACTAATTTGCCAAATAGCAACTGATA-TTTTGTA-
TGTTTTTGTAGTTTCCATGTTGGAAGTGGATGTACAGACCCAGAGACCTTTGTTCAAGCCATTTCTGATGCCCGCTGTGT
GTTTGATATGGGAGTAAGTCT-AGTTCTACTTTTTCTGGAA-CTACTGCTTAACTGTTGTGGCAA-------TAC-
TGAATGAAGTGTACAACTGTTATGGGGTTAAAA--------------GCTAGCTAA-GTC???GACTT-----
TGTTAGAATTTTTTAGATCCT-GGT---
----------------------------AGCTTACTTT-GATCAGCTTGGC-AAAACTCATC-TAC------AGATGC-
ACTAAAATTGGCAGGTCAAACTT---------TGTT-ACTTGAAC---ATG?GTTAGCTC-
TGTTTCAAGTGTTAATGAAATTACTTGACATTAC-
TTTTTAAG??
???????????????????????????????????????
Sitta
TAAATTTGGGGCTACACTTAAGACTAGCAGGCTTCTTCTGGAGCGTGCTAAAGAACTTGACCTTGCCATTGTTGGAGTTA
GGTGAG-CTGACAGT--ATCAGAAC-------ACC------------------GT--CATATTGAA-
TAAGTTTTCTTTAAAT------------------------GGTCTACCTGACAATAGAGA----
TGCTTTTGCATGTGATAA---GAGT--TTATAGAAAATTTCTTGACTGATTTGTCAAATAGCAACTGATA-
TTTTGTATTTTTTTTGTAGTTTCCATGTTGGAAGTGGATGTACTGACCCAGAGACCTTTGTTCAAGCCATTTCTGATGCC
CGCTGTGTGTTTGATATGGGAGTAAGTCT-AGTTCTACTTTATCTGGAA-CTACTGTTCAATTATAGTGGCAA-------
AAC-TGAATGAAGTGTACAGCTGTTATGGGGTCAAAA--------------ACTAACTAA-
GTCAGTGACTTCATGTTGCTGAAATTTTTTAGGTCCT-GGT---------------------------------------
--AGCTTATTTC-AACCAGCTTGGC-AAAACTCATC-

TAC------AGATGC-ACTAAAATTGGCAGCTCAAATTC-AAGTGAC-TGTT-ACTTGGAC---ATGTGTTATCTC-
TGTTTTAAGTGTTAATTAAAT----------------TTTAAGCTAAAATG----
ATAAATTATAAACTGATATAACTTATAAACTTTTCTTGACAGTGTTGCTGTTAATCTCA-
TTTTAGGCTGAACTTGGCTTCAATATGTATCTGCTTGAT
Mimus
TAAATTTGGAGCTACACTTAAGACTAGCAGGCTTCTTCTGGAGCGTGCAAAAGAGCTTGACCTTGCCATTGTTGGAGTTA
GGTGAG-CCAACAGT--ACCAAAAC-------ACA------------------GT--TGTATTAAA-
TAAGTTTTCTTTAAT-------------------------GGTCTGCCTGACAATAGAGG----
TGCCTTCGCATGTGATGACATGAGT--TTATATAAAACTTCTTGACTAACTAACCAAATAGCAACTGATA-YTTTGTA-
TCTTTTTGTAGTTTCCATGTTGGAAGTGGATGTACAGACCCAGAGACCTTTGTTCAAGCCATTTCTGATGCCCGCTGTGT
GTTTGATATGGGAGTAAGTCT-AKTTCTACTTTCTCTGGAA-CTACYGCTCCACTGTTGTGGCAA-------AAC-
TGAATGAAGTGTGCAACTGTTATGGGCTTAAAA--------------GCTAGCTAA-
GTCACTGACTTCATATTGTTGGAWTTTTTTAGATCCT-GGT---------------------------------------
--AGCTTACTTT-GATCAGGTTGGC-AAAACTCATC-
TAC------AGATGC-ACTAAAACTGTCAGCTCAAACTCAAAAAAAC-TGTT-GCTTGAGC---ATGTGTTAGCTC-
TGTTTCAAGTGTTAATGACATTTCGTCATGTTAC-TTTTCAAGCTAAAATG----ATACATTATAAACT-----------
------TTTCTTGACAGTGTTCCTGTTCATGTCA-TTTTAGGCTGAACTTGGCTTCAATATGTATCTGCTTGAT
Sturnus_vulgaris
TAAATTTGGAGCTACACTTAAGACTAGCAGGCTTCTTCTGGAGCGTGCAAAAGAGCTTGACCTTGCCATTGTTGGAGTTA
GGTGAG-CTGACAGTACACAAAAAC-------ACA------------------GT--TATATTAAA-
TAAGTTTTCTTTAAT-------------------------GGTCWATCTGACTAYAGAGA----
TGCCTTTGCATGTGATGACATGAGT--TTATATAAAACTTCTTGACTAATTAGCCAAATAGCAACTGATA-TTTTGTA-
TCTTTTTGTAGTTTCCATGTTGGAAGTGGATGTACAGACCCAGAGACCTTTGTTCAAGCCATTTCTGATGCCCGCTGTGT
GTTTGATATGGGAGTAAGTCT-AGTTCTACTTTCTCTGGAA-CTACTGCTCAACTGTTGTCACAA-------AAC-
TGAATGAAGTCTGCAACTGTTATGGGGTTAAAA--------------TCTGGCTAA-
GTCACTGACTTCATATTGTTGGAATTTTTTAGGTCCT-GGT---------------------------------------
--AGCTTACTTT-GACCAGCTTGGT-AAAATTCATC-
CAC------AGATGC-ACTAAAATTAGCAGTTCAAACTC--AAAAAC-TGTT-GCGTGAAC---ATGTGTTAGCTC-
TGCTTCTAGTGTTAATGAAACTTCTTCATGTTAC-TTTTTAAGCTAAAATG----ATACGTTATAAACT-----------
------TTTCTTGACAGTGTTGCTGTTAATGTC--TTTTAGGCTGAACTTGGCTTCAATATGTATCTGCTTGAT
Creadion_caruncculatus
??
??
??
??
??
??
??
??
??
??
??
?????????????
Parus
TAAATTTGGGGCTACACTTAAGACTAGCAGGCTTCTTCTGGAGCGTGCAAAAGAACTTGACCTTGCCATTGTTGGAGTTA
GGTGAG-CTGACAGT--ATCAAAAC-------GCA------------------GT--AGTATTGAA-
TAAGTTTTCTTCAAAT------------------------GGTCTACCTGACAATAGAGA----
TGCCTTTGCATGTGATGACATGAGT--TTATACAAAACTTCTTGATTAATTTGCCAAATAGCAACTGATA-TTTTGTA-
TCTTTTTGTAGTTTCCATGTTGGAAGTGGATGTACAGACCCAGAGACCTTTGTTCAAGCCATTTCTGATGCCCGCTGTGT
GTTCGATATGGGAGTAAGTCT-AATTCTACTTTCTCTGAAA-CTACTGCTCAACTGTTGTGGCAA-------AAC-
TGAATGAAGTGTACAACTATTCTGGGGTTAAAA--------------GCTAGCTGA-
GTCACTGGCTTCATGTTGTTGGAATTTTTTAGGTCTT-GTT---------------------------------------
--AGCTTACTTT--ACCAGCTTGGC-AAAACTCATC-
TAC------TGATGCAACTAAAATTGGCAGCTTAAACTC-AAATGAC-TGTT-ACTTGAAA---ATGTGTTAGCTC-
TGTTTCAAGTGTTAATGAAATTACTTCACATTAG-TTTTTAAGATAAAATG----ATAAATTATAAA----------
TGATAAACTTTTCCTGACAGTGTTGCTCTTAATCTCA-TTCTAGGCTGAACTTGGCTTCAGTATGTATCTGCTTGAT
Petroica_rosea
??
??
??

??
??
??
??
??
??
??
??
?????????????
Eopsaltria_australis
??GCGTGCAAAAGAACTTGACCTTGCCATTGTTGGAGTTA
GGTGAG-CTGACAGC--ACCAAAGC-------ACA------------------GT--CATATTAAA-
TAAGTTGTCTTTAAAT------------------------GGTTCACCTGACAATAAAGA----
TGCCTTTGCATGTGATGACATGAGT--TWATACAAAAYTTCTTGACTAATTTGCCAAATAGCAACTGATA-GTTTGTA-
TCTTTTTGTAGTTTCCATGTTGGAAGTGGATGTACAGACCCAGAGACCTTTGTTCAAGCCATTTCTGATGCCCGCTGTGT
GTTTGATATGGGAGTAAGTCT-GGTTCTGCTTTCTCTGGAG-CTACTGCTCAACTGCTGTGGCAA-------AAC-
TGAAWGAAGTGTACAACTTCTATGGGGTTAAAA--------------GCTAGCTAA-
GTCACTGACTTCATGTCATTGGAATTTTTGAGGTTCT-GGT---------------------------------------
--AGCTT---------------GGC-
AAAACTCATCTTAC------AGATGC-AGTAAAATTGGCAGCTCAAACTC-AAATGAC-TGTT-ACTTGAAC---
ATGAGTTAGCTG-TGTTTCAAGTGTTAATGAAATGACTTCACATTAC-TTTTTAAGCTAAAATG----
ATAAATTATAAACTGATACAAATGATAAACTTTTCTTGACAGTGTTGCTGTTAATCTCA-
TTTT???????????????????????????????????
Serinus
??
??
??
??
??
??
??
??
??
??
??
?????????????
Icterus
TAAATTTGGAGCTACACTTAAGACCAGCAGGCTTCTTCTGGAGCGTGCAAAAGAACTTGACCTTGCCATTGTTGGAGTTA
GGTGAG-CTGACAGT--ATCAAAAC-------GCA------------------GT--TGTACTAAA-
TAAATTTCCTTTAAATTTCCTTTAAACATCTCTATTGTCAGGTCTACCTGACGATAGAGA----
TGCCTTTGCATGTGATGACGTGAGT--TTATACAAAATTTCTTGAGTAATTTGCCAGATAGCAACTGATA-TTTTGTA-
TCTTTTTGTAGTTTCCATGTTGGAAGTGGATGTACAGACCCAGAGACCTTTGTTCAAGCCATTTCTGATGCCCGCTGTGT
GTTTGATATGGGAGTAAGTCT-AGTTCTACTTTCTCTGGAA-CTACTGCTCAACTGTTGTGGCAA-------ACC-
TGAATGAAGTGTACAACTGTTATGGGGTTAAAA--------------GCTAGCTAA-
GTCACTGACTTGATGTTGTTGGAATTTTTTAGGTCCT-GGT---------------------------------------
--AGCTTACTTT-GACCAGCTTGG--AGAACTAAAG-
AACTAAATTTGAAGA-ACTAAAATTGGCAGCTCAAACTC-AAATTAC-TGTT-ACTTGAAC---ATGTGTTAGCTC-
TATTTCAAGTGTTAATGAAATGACTTCACGTTAC-TTTTAAAGCTAAAATG----
GTAAATTATAAACTGACATAAATTATAAACTTTTCTTGACAGTGTTGCTCTTTGTTTCA-
TTTTAGGCTGAACTTGGCTTCAATATGTATCTGCTTGAT
Motacilla
??????????????????????????????GCTTCTTCTGGAGCGTGCWAA?GAACTTGAYCTTGCCATTGTTGGAGTTA
GGTGAG-CTGACWAT--ATCAAAAC-------ACA------------------GT--CATACTAAA-
AAAGTTTCCTTTAAAA------------------------GGTCSACCTGACAATAGATA----
TGCCTTTGCTTGTGATGACATGGGT--TTATGCAAAAATTATTGAGTAATTTGCCAAATAGCAACTGATA-TTTTTTA-
TCTTTTTGTAGTTTCCATGTTGGAAGTGGATGTACAGACCCAGAGACCTTTGTTCAAGCCATTTCTGATGCTCGTTGTGT
GTTTGATATGGGAGTAAGTCT-AGTTCTACTTTCTCTGGAA-CTACGGCTCAACTATTGTGGCAA-------AAC-
TGAATGAAGYGTACAACTGTTATGGGGTTAAAA--------------GCCAGCTAA-
GTCACTGACTTGATGTTGTTGGAATTTTTTAGGTCCT-GGT---------------------------------------
--AGCTTACTTT-GACCAGCTTGGC-AAAA--------

----------------CTAAAATTGGCAGCTCAAACTC-AAATTAC-TGTT-ACTTGAAC---ACGTGTTASCTC-
TGTTTCAAGTGTTAATGAAATGACTTCAGATTAC-TTTTTAAGCTAAAATG----
ATAAATTATMCACTGACATAAATTGTAAACTTTTCTTGACAGTSTTGCTGTTCATTT???????????????????????
???????????????????
Emberiza
TAAATTTGGAGCTACACTTAAGACCAGCAGGCTTCTTCTGGAGCGTGCAAAAGAGCTTGACCTTGCCATTGTTGGAGTTA
GGTGAG-CTGACAGT--ATCAAAAC-------ACA------------------GT--TGGACTAAA-
TAAATTTCTTTTAAAT------------------------GGTCTACCTGACAGTAGAGA----
TGCCTTTGCGTGTGATGACATGAGT--TTATACAAAACTTCTTGAGTMATTTGCCAAATAGCAACTGATA-TTTTGTA-
TCTTTTTGTAGTTTCCATGTTGGAAGTGGATGTACAGACCCAGAGACCTTTGTTCAAGCCATTTCTGATGCCCGCTGTGT
GTTTGATATGGGAGTAAGTCT-AGTTTACTTTCTCTGGAA-CTCCTGCTCAACTGTTGTGGCAA-------GAC-
TGAGTGAAGTGTACAACTATTATGGGGTTAAAA--------------GCTAGCTAA-GTC------
TTGATGTTGTTGGAATTTTTAGGTCCT-GGT---
-----------------------------------AGCTTACTTT-GACCAGCTTGGC-AGAA-----------------
-------CTAAAATTGGCAGCTCAAACTCAAAATTAC-TGTT-ACTTGAAC---ATGTGTTAGCTC-
TGTTTCAAGTGTTAATGAAATGTCTTCACATTAC-TTTTTAAGCTAAAATG----ATAAATTATAAACT-----------
------TTTCTTGACAGTATTGCTGTTCATTTCA-TTTTAGGCTGAACTTGGCTTCAATATGTATCTGCTTGAT
Pomatostomus
??GCGTGCAAAAGAACTTGACCTTGCCATTGTTGGAGTTA
GGTGAG-TTGACAAC--ACCAAAAC-------GCA------------------GT--CATATGAAA-
TAAGTTTTCTTTAAAT------------------------GGTCTGCCTGACAATAGAGA----
TGCCTTTGCATGTGGTGACATGAGT--TTATACAAAACTTCTTGACTAATTTGCCAAATAGCAACTGATA-GTTTGTA-
TCTTTTTGTAGTTTCCATGTTGGAAGTGGATGTACAGACCCAGAGACCTTTGTTCAAGCCATTTCTGATTCCCGCTGTGT
GTTTGATATGGGAGTAAGTCT-AGTTCTATTTTCTCTGGAA-CTACTGCTCAACTGTTGTGGCAA-------AAC-
TGAATGAAGTGCACAACTGTTATGTGGTTAAAA--------------GCTACCTAA-
GTCACTGATTTCATGTTGTTGGAATTTTTGAGGTCCT-GGT---------------------------------------
--AGCTTACTTT-GACCAGCTTGGC-AAAACTCATC-
TAC------AGATGC-ACTAAAATTGGCAGCTCAARCTC-AAATGAC-TATT-ACTTGAAC---ATGACTTAGCTC-
TGTTTCAAGTGTTAATGAAATGACTCCACATTAT-TTTTTAAGCTCAAATG----ATAAATTATAAACTGA---------
------TTTCTTGACAGTGCTGCTGTTAATCTCA-TTTT???????????????????????????????????
Rhipidura
??GCGTGCAAAAGAACTTGACCTTGCCATTGTTGGAGTTA
GGTGAG-CTGACAGT--ATCAAAAC-------GCA------------------GT--CATATTAAA-
TAAGTCTTTTATAGAT------------------------GGTCTACCTGACAATAGAGA----
TGCCTTTGCTTGTGGTGGCATGAGC------------CTTCTTGACTGATTTGCCAGATAGCAACTGATA-ATCTGTA-
TCTTTTGGTAGTTTCCATGTTGGAAGTGGATGTACAGACCCAGAGACCTTTGTTCAAGCCATTTCTGATGCCCGCTGTGT
GTTYGATATGGGAGTAAGTCT-AGTTCTACTTTTTCTGGAA-TTACTGCTYAACTGTTGTGGCAA-------GGC-
TGATTGAAGTGT-------TTATGGGATTAAAA--------------GCTAGCTAA-
GTCACTGACTTCATGTTGTTGGAATTTTTGAGGTCCT-
GGTAAAGTTTTGTCAGTAGATAAGTTTTGCCAAGCAAAAGTTATCTAGTTATCAAGTTTTAGTGCATCAGTAGGTAAGTT
TTGTCAAGGTTATTTT-GACCAGCTTGGC-AAAACTTATC-TAC------TGATGC-
ACTAAAATTGGCAACTCAAACTC-AAATGAC-TGTT-ATTTGAAC---ATGAGTTAGCTC-
TGTTTCAAGTGTAAATGAAATG-----------------------AAAATG----ATAAGTTATAAACTGR---------
------TTTTTTGACAGTGTTGCTGTTAATCTCA-TTTT???????????????????????????????????
Pica_pica
??
??
??
??
??
??
??
??
??
??
??
?????????????
Manucodia
??GCGTGCAAAAGAACTTGACCTTGCAATTGTTGGAGTTA
GGTGAG-CTGACAGT--ATCAAAAC-------ACA------------------GT--CATATTAAA-TA--

TTTTTTATAAGT------------------------GGTCTACCTGACAATAGAGA----
TGTCTTTGCTTGTGGTGGCATGAGT--TTATACAAAACTTC-TGATTAATTTGCCAAATAACAACTGATA-ATTTGTA-
TCTTTTTGTAGTTTCCATGTTGGAAGTGGATGCACAGACCCAGAGACCTTTGTTCAAGCCATT?????????????????
?????ATATGGGAGTAAGTCT-AGTTCTGCTTTTCCTGGAA-CTGCTGCTCAACTGTTGTGGCAA-------AGC-
TGAATGAAGTGTACAATTTTTATGGGGTTAAAAGCTAAGCTTTAGTGACTAGCTAA-
GTCACTGACTTCATGTTGTTGGAATTTT----GTCCT-GGT---------------------------------------
--AGCTTACTTT-GACCAGCTTGGC-AAAACTCAGC-
GAC------TGATGC-ACTAAAATTGGCAACTCAAACTC-AAATGAC-TGTT-ACTTGAAC---ATGAGTTAGCTC-
TGTTTCAAGTGTTAACGAAATGGTTTAACGTTAC-TTTTTAAGCTAAAATG-----------ATAAACTGA---------
------TTTTTTGACAGTGTTGCTGTTAATCTCA-TTGT???????????????????????????????????
Corvus_corone
TAAATTTGGAGCTACACTTAAGACTAGCAGGCTTCTTCTGGAGCGTGCAAAAGAACTTGACCTTGCCATTGTTGGAGTTA
GGTGAG-CTGACAGT--ATCAAAAC-------ACA------------------GT--CATATTAAA-TAAGTTT------
-----------------------------------TAACAATAGAGA----TGCCTTTGCTTGTGGTGGCATGAGT--
TTATACAAGACTTCTTGACTAATTTGCCAAATAGCAACTGATA-ATTTGTR-
TCTTTTTGTAGTTTCCATGTTGGAAGTGGATGTACAGACCCAGAGACCTTTGTTCAAGCTATTTCTGATGCCCGCTGTGT
GTTCGATATGGGAGTAAGTCT-
AGTTCT??
??
??
??ACTAAAATTGGCAACTC-------AAATGAC-
TGTT-AATTGAAC---ATGAGTTAGCTC-TGTTTCAAGTGTTAACAAAATGACTTAACATTAC-
TTTTTAAGCTAAAATG----ATAAATTATAAACTGA---------------TTTTTTGACAGTATTGTTGCTAATCTCA-
TTTTAGGCTGAACTTGGCTTCAATATGTATCTGCTTGAT
Vireo
??GCGTGCAAAAGAACTTGACCTTGCCATTGTTGGAGTTA
GGTGAG-CTGACAGT--ATCAAA---------ACA------------------GT--CATATTTAA-
TAAGTTTTCTTTAAGT------------------------GGTCTACCTGACAATAGAGA----
TGTCTTTGCATGTGCTGACATGAGT--TTATACAAAAYGTCTTGACTGATATGSCAAATAGCAACTGATA-GTGTGTA-
TCTTTTTGTAGTTTCCATGTTGGAAGTGGATGTACAGACCCAGAGACCTTTGTTCAAGCCATTTCTGATGCCCGCTGTGT
GTTTGATATGGGAGTAAGTCT-AGTTCTACTTTCTCTGGAA-CTACTGCTCAACTATTRTGGCAA-------AAC-
TGAATGAAGTGTACAACTTCTATGGGGTTAAAA--------------GCTAGCTAA-GTCACTGACTTCA---
TGTTGGAATTTTTGAGGTCCT-GGT---
----------------------------AGCTTACTTT-GACCAGTTCAAC-AAAACTCATC-TAC------AGATGC-
ACTAAAATTGGCAGCTCAAACTC-AAATGAC-TATT-ACTTGAAC---ATGAGTTAGCTC-
TGTTTCAGGTGTTAATGAAATTACTTCACATTAC-TTTTTAAGCTAAAATG----ATAAATTATAAACTGA---------
------TTTCTTGACAGTGTTGCTGTTAATCTCA-TTTT???????????????????????????????????
Camptostoma_obsoletum
??????TGGAGCTACACTTAAGACCCGCAGGCTTCTCCTGGAGCGTGCAAAAGAACTTGAGCTTGCCATTGTTGGAGTTA
GGTGAG-TTGATAAT--GTCAAA---------ATA------------------GT--CATATAATC-
TTAGTTTTTCTTAAAA------------------------TGTTTTAGTAAGGACAGAGA----
TGCCTTTACATGTGGTGACATGAGT--TTATACAAGACTTCCTGACTAATTTGCCAAGTAGCAACTGATA-TTTTGTA-
TCTTTTCACAGTTTCCATGTTGGAAGTGGATGTACAGACCCAGAGACCTTTGTTCAAGCCATTTCTGATGCCCGCTGTGT
GTTTGATATGGGAGTAAGTCT-AATTCTAYTTTCTCTGGAA-CTACTGCTCAACT--TGTGTTGATCRARYTGTG-
TTAATCAAGTGTGAATATGTTACAGGGTTAAAAAA------------GCTGACTAA-GTCAATGATTTCA---
TGTTGGAATTTTTGAGGRCAT-GAK---
----------------------------GGCTTACTTT-GACCAGTTTGGC-AAAACTCATC-TAC------AGATGC-
ACTAAAATTGGCAGCTCRTACTC-AAGTGAC-TATT-ACTTGAATATGATGAGTTRTCTC-
TGCTCCAAATACTAGTGAAATGACTTCACATTAC-CTTTTAAGCTAAAATG----ATAAATTAACAACTGA---------
------TTTCTTGACCATGTTGCCATTAATCTCA-TTTTAGGCTGAACTTGGCTTCAGTATGTAT?????????
Promerops_cafer
TAAATTTGGAGCTACACTTAAGACTAGCAGGCTTCTTCTGGAGCGTGCAAAAGAACTTGACCTTGCCATTGTTGGAGTTA
GGTGAG-CTGACAGT--ATCAAAAC-------ACA------------------GT--CACATTAAA-
TAAGTTTTCTTTAAAT------------------------GGTCTGCCTGATAATTGAGA----
TGCCTTTTCATGTGATGACCTAGGT--TTATACAAAACTTCTTGACTAATTTGCTAAATAGCAACTGATA-TTTTGTA-
TCTTTCTGTAGTTTCCATGTTGGAAGTGGATGTACAGACCCAGAGACCTTTGTTCAAGCCATTTCTGATGCCCGCTGTGT
GTTTGATATGGGAGTAAGTCT-AGTTCTACTTTCTCTGGAA-CTACTGCTCAACTGTTGTGGCAA-------AAC-
TAAATGAAGCGTATAAGTGTTACGGGGTTAAA-----------------------A-GTCACTGATTTGA---
TGTTGGAATTTTTGGGTCCT-GGT--
----------------------------AGCTTATTTT-GACCAGCTTGGC-ACAA------------------------

CTAAAATTGGCAGCTCAAATGC-AAATGAC-TGTT-ACTTGAAC---ATGTGTTAGCTC-
TGTTTCAAGTGTTAATGAAATTAT------TTAT-TTTTTAAGCTAAAATG----
AGAAATTATAAACTGATACAAATTATAAACTTTTCTTGACAGTTTTGCTGTTAATCTCA-
TTTTAGGCTGAACTTGGCTTCAGTATGTATCTGCTTGAT
Oriolus
TAAATTTGGAGCTACACTGAAGACTAGCAGGCTTCTTCTGGAGCGTGCGAAAGAACTTGACCTTGCCATTGTTGGAGTTA
GGTGAG-CTGACAGT--ATCAAAAC-------ACA------------------GT--CATATTAAA-
TAAGTTCTCTTTAAAAT-----------------------GGTCTRTATGACGATAGAGA----
TGCCTTTGCTTGTGGTGACATGAGT--TTATACAAAATTTCTTGCCTAATTTGCCAAATAAAAACTGATA-GTTTGTA-
TCTTTTTGTAGTTTCCACGTTGGAAGTGGATGCACAGACCCAGAGACCTTTGTTCAAGCCATTTCTGATGCCCGCTGTGT
GTTTGATATGGGAGTAAGTCT-AGTTCTACTTTCTCTGGAG-CTACCGCTCAGCTGTTGTGGCAA-------AAC-
TGAATGATGTGTACAATTGTTACGGGGTTAAAA--------------GCTAGCTAA-
GTCACTGACTTCGTGTTGTTGGAATTTTTGAGGTCCT-GGT---------------------------------------
--AGCTTACTTT--ACCAGCTTGGC-AAAACTTATC-
TAC------AGATGC-ACTAAAATTGGCAGCTCAAACTC-AAATGAC-TATT-ACTTGAAC---ATGAATTAGCTC-
TGTATCAAGTGTTAATGAAATTACTTAATGTTAC-TTTTTAAGCTAAAATG----ATAAATTATGAACTGG---------
------TTTCTTGACAGTGTTGCCATTAATCTCATTTTTAGGCTGAACTTGGCTTCAATATGTATCTGCTTGAT
Dendroica
??
??
??
??A-
TTTTTTTGTAGTTTCCATGTTGGAAGTGGATGTACAGACCCAGAGACCTTTGTTCAAGCCATTTCTGATGCCCGCTGTGT
GTTTGATATGGGAGTGAGTGT-AGTTCTACTTTCTCTGGAA-CTATGGGTCAACTACTGTGGCAA-------AAC-
TAAATGAAGTGTACAACTGTTATGGGGTTAAAA--------------GCTAGCTAA-
GTCACTGACTTGATGTTGATCGAATTTTTTAGGTCCTGGGT---------------------------------------
--AGCTTACTTT-GATCAGCTTGGC-AGAA--------
----------------CTAAAATTGGCAGCTCAAACTC-AAATTAC-TGTT-ACTTGAAC---ATGTGTTAGCTC-
TATTTCAAGTTTTAATGAAATGATTTCTTGTTAC-TTTTAAAGCTAAAATG----
ATAAATTATAAACTGACATAAATTATAAACTTTTCTTGACAGTGTTGCTGTTCATTTCA-
TTTTAGGCTGAACTTGGCTTCAATATGTATCTGCTTGAT
Nectarinia
??????TGGAGCTACACTTAAGACTAGCAGGCTTCTTCTGGAGCGTGCAAAAGAACTTGACCTTGCTATTGTTGGAGTTA
GGTGAG-CTGACAGT--ATCAAAAT-------ACA------------------GT--CCTATTAAA-
TCAGTTTTCTTTAAAT------------------------GGTCTACCTGACAATAGAGA----
TGCCTTTGCATGTGATGACATGAGT--TTATGCAAAATTTCTCGACTAATTTGCTAAATAGCAACTGATA-TTTTGTA-
TCTTTTTGTAGTTTCCATGTTGGAAGTGGATGTACAGACCCAGAGACCTTTGTTCAAGCCATTTCTGATGCCCGCTGTGT
GTTTGATATGGGAGTAAGTCT-AGTTCTACTTTCTCTTGAA-CTCCTGCTCAACTGTTGTGGTAT-------AAC-
TGAATCAAGTGTACAACTGTTAAGGAGTTAAAA--------------GCTAGCTAA-
GTCACTGACTTGATGTTGTTGGAATTTTTTAGGTCCT-GGT---------------------------------------
--AGCTCACTTT-GACCAGCTTGGC-AAAA--------
----------------GTAAAATTGGCAGCTGAAACTC-AAATGAC-TCTT-ACTTGAAC---CTGTGTTAGCTT-
TGTTTCAAGTGTTAATGAAATGACTTTACATTAC-TTTTTAAGCTAAAATG----
ATAAATTATAAACTGATATAAATTATAAACTTCTCTTGACAGTGTTGATGTTAATCTCA-
TTTTAGGCTGAACTTGGCTTCAGTATGTAC?????????
Amytornis_striatus
??
??
??
??
??
??
??
??
??
??
??
?????????????
Pitta
??GCGTGCAAAAGAGCTTGACCTTGCCATTGTTGGGGTTA

GGTGAG-TTGATAAT--GTCAAAGC-------ACA------------------GT--CATATTAAA-
TAACTTTTCTTTAAAT------------------------GGTCTACATCATGATAGAGA----
TGCCTTTGCAGGTAGTGACATGAGTTTTTATACAAGACTTCATGACTGATTTGCCAAATAGCAGCTGATA-TTTTGTA-
TCTTTTCATAGTTTCCATGTTGGAAGTGGATGCACAGACCCAGAGACATTTGTTCAAGCCATT????????????????
?????ATATGGGAGTAAGTCC-AATTCTGTCTTCTCTGGAA-TTACTTGCTTTTTTGTGTGTCAA-------AAC-
TGAATCAAGTGTGAAGCTGTTACAGGGTTAAAA--------------GCTGGCCAA-GTCACTGGCTTCA---
TGTTGGAATTTGTGA---
----------------------------GGCTTACTTT-GACCACTTTGGC-AAAACTCATC-TCC------AGATGC-
AGTAAAATTGGCAGCTTAAACTC-AGATGAC-TGTT-ACTTGAGCATCACGA--
TAACTCATGTTCCAAGTATTAGTGAAATGATTTCACATTAC-TTTTTAGCTAAAATG----ATAAATGAACAACTGA--
--------------TTTCTTGACAGTGTTGC-ATTAATTTCA-
TTTT????????????????????????????????????
Toxorhamphus
???GCGTGCTAAAGAACTTGACCTTGCCATTGTTGGAGTTA
GGTGAG-CTGACAAT--ATCAAAAC-------ACA------------------GT--CATATTAAG-
TAAGTTTTCTTTAAAT------------------------GGTCTACCTGACAATAGAGA----
TGCCTTTGCATGTGATGACGGGAGT--TTATACAAAACTTCTTGACTAATTTGCCAAATAGTAACTGATA-GTTTGTA-
TCTTTTTGTAGTTTCCATGTTGGAAGTGGATGTACAGACCCAGAGACCTT?????????????????????????????
?????ATATGGGAGTAAGTCT-AGTTCTACTTTCTCTGGAA-CTACTGCTCAACTGCTGTGGCAA-------AAC-
TGAATGAAGTGTACATCTTTTATGGGGTTAAAA--------------GCTTGCTAA-
GTCACTGACTTCATGTTGTTGGAATTTTTTAGGTCCT-GGT---------------------------------------
--AACTTACTTT-GACCAGCTTGGC-AAAACTCACC-
TAC------AGATGC-ACTAAAATTGGCAGCTCAAACTC-AAATGAC-TATT-ACCTGAAC---ATGAGTTAGCTC-
TGTTTCAAGTGTTAATGAAATGACTTCACATTAC-TTTTTAAGCTAAAATG----
ATAAATTGTAAAATGATGTAAATTACAAACTTTTCTTGACAGTGTTGCTGTTAATCTCA-
TTTT????????????????????????????????????
Orthonyx_temminckii
???GCGTGCAAAAGAACTTGACCTTGCCATTGTTGGAGTTA
GGTGAG-CTGCCAAT--ATCAAAAC-------CCA------------------GT--CATACTAAA-
TAAGTTTTCTTTAAAT------------------------GGTCTACCTGACAAGAGAGA----TGCCTT-
GCATGTGATGGCATGAGT--TTATACAAAACTTCTTGACTAATTTGCCAAATAGCAGCTGATA-GTTTGTA-
TCTTTTTGTAGTTTCCATGTTGGAAGTGGATGTACAGACCCAGAGACCTTTGTTCAAGCCATTTCTGATGCCCGCTGTGT
GTTTGATATGGGAGTAAGTCT-AATTCTACTTTCTCTGGAA-CTACTGCTCAACT-----GGCAA-------AAC-
TGAATGAAGTGTACAACTTTTATGTGGTTAAAA--------------GCTAGCTAA-
GTCGCTGACTTCATGTTGTTGGAATTTTTGATGTCCT-GAT---------------------------------------
--AGCGTACTTT-GACCAGCTTGGG-AAAACTCACC-
TAC------AGATGC-ACTAAAATTGGCAGCTCAAACTC-AAATTAC-TATT-ACTGGAAC---ATAAGTTAGCTC-
TGTTTCAAGTTTTAATGAAATGACTTCACATTAC-TTTTTAAGCTAAAATG----ATAAATTATAAACTGA---------
------TTTCTTGACAGTGTTGCTGTTAATCTGA-TTTT????????????????????????????????????
Sericulus_chrysocephalus
??
??
??
??
??
??
???CT-GGT----------------
--GGCTTACTTT-
GTCCTGTTTGGC-AAAACTCA?TTACA------AGATGC-ACTAAAATTGGCAGCTCAAATGC-AAATGAG-??TT-
ACTTGAACATGATGAGTTAGCTG-TATTCCAAGTATTAATGAAA??????????TTGC-CTTTTAAGCTAAAATG----
GTAAATTATAAAC???
???????????????????
Cyclarhis_gujanensis
???GCGTGCAAAAGAACTTGACCTTGCCATTGTTGGAGTTA
GGTGAG-CTGACAGT--ATCAAA---------ACA------------------GT--CATATTTAA-
TAAGTTTTCTTTAAGT------------------------GGTCTACCTGACAATAGAGA----
TGCCTTTGCTTGTGCTGACATGAGT--TTATACAAAACGTCTTGACTGATATGCCAAATAGCAACTGATA-GTTTGTA-
TCTTTTTGTAGTTTCCATGTTGGAAGTGGATGTACAGACCCAGAGACCTTTGTTCAAGCCATTTCTGATGCCCGCTGTGT
GTTTGATATGGGAGTAAGTCT-AGTTCTACTTTCTCTGGAA-CTACTGCTCAACTATTGTGGCAA-------AAC-
TGAATGAAGTGTACAACTTCTATGGGGTTAAAA--------------GCTAGCTAA-GTCACTGACTTCC---

TGTTGGAATTTTTGAGGTCCT-GGT--
----------------------------AGCTTACTTT-GACCAGTTCAGC-AAAACTCATC-TAC------AGATAC-
ACTAAAATTGGCAGCTCAAACTC-AAATGAC-TGTT-ACTTGAAC---ATGAGTTAGCTC-
TGTTTCAGGTGTTAATGAAATGACTTCACATTAC-TTTTTAAGCTAAAATG----ATAAATTATAAACTGA---------
------TTTCTTTACGGTGTTGCTGTTAATCTCA-TTTT???????????????????????????????????

[myoglobin]
Polyborus_plancus CAAATATCTGGAGGTATGGAAAAGGGCAGG-AAATCTTGGTATC-T--------
-GATGTGTGGTGAATGTGT--GCAAGA-CAGCTATGTGAGAGCTGTGCTTTTATTTACTGATGGCCAGTTGGACTTC-
AGTGAGCCCTCCCTCAAGTCCAAGGTCTCTGTGTACATGCAGGAGGAGGCACAGAAAAAGGGCTCATGGTATGCAAATGG
TATGT------GAATATCCAAGTTTAGATTTCCCATTCCAAACA-
CCACATGCAGTCTGACCAATCCTCGACAATAATAAACC-AGCTCAT-GCAGCCTCTGCATGCCTGGGAAACTA-
CATTACA-TAAGAACTATC---AGCG--------GCTGGACACAAGGGATGTA--CAATTTTAAAGTAAGCCCT-
GGAGGATCCATTGGAGACCAAGACCCACAAAACTAAGTGTTGTACAAACACAGGGCATGAATTTT-
CAGCCTTAATAGATGAGAT-AGACAAG-
AGTGGGAAGGGCCATGGTCTACTCAAGGTCATGAAGCAGATTGGCGTCACAGCTAGGAATAGAGCCCAGCTCTTCTGCCC
AGCCCAGGCTCCTTGCATGCTAGACCTCGCTGTCTCTCCAGAGACTGTGGGAAGGGCTACTGAATTAGCTCGGAGATATT
TCCAGGCTCTGCAACCTATTTCTTAGTCTGGCTTTTCTGAAAATAAACCTTGAGTGTCCCTTCAG--TTT-
TTTTTCTCTCTTTCCTTCCTCACAGTTCATTTCTG
Falco CAAATATCTGGAGGTATGGAAAAGGGCAGG-AAATGTTGGTGTC-T--------
-GATGTGTAGTGAATGTGT--GCAAGA-CAGCTATGTGAGAGCTGTGCTTTTTTTTACTGATGGCTGCTTGGACTCC-
AGTGAGCCCTCCCTCAAGTCCAAGGTCTCTGTGTATATGCAGGAGGAGGCACAGAAAAAGGGTTCATGGTATGCAAATGG
TATGT------GAATCTCCAAGTTTAGATTTCCCATTCCAAACA-
CCACATGCAGTCTGACCAATCCCTGACAATAATAAACC-AGCCCGT-GCAGCCTCTGCATGCCTGGGAAACAA-
CATTACA-TAAGAACTATC---AGTG--------GCTGGACACAAGGGATGTA--CAATTTTAGAGTAAGCCCC-
GGAGGATCCATTGGAGACCAAGACCCACAAAACTAAGTGTTGTACAAACACAGGGCATGAATTTT-
CAGCCTTAATGGATGAGAT-AGACAAG-
AGTGGGAAGGGCCATGGTCTACTCAAGGTCATGAAGCAGATCAGCGTCACAGCTAGGAATAGAGCCCAGTTCTTCTGCCC
AGCCCAGGCTCCTTGCATGCTAGACCTCGCTGTCTCTCCAGAGACTGTGGGAAGGGCTACTGAATTAGCTCGGAGATATT
TCCAGGCTCTGCAACCTATTTCTTAGTCTGGCTTTTCTGAAAATAAACCTTGAGTGTCCCTTCAGTTTTCTTTCTCTCT
CTCTCCTTCCTCACAGTTCATTTCTG
Tyrannus CAAATATCTGGAGGTATGGAAAAGAGCAAG-GAATCTTGGTGTC-T--------
-GATGCGCAGTGAATGTGT--GTAAGA-CAGCTATGTGAGAGCTGTGCTTTTATTTACCAATGACTAGTTGGACTTC-
AGTGAGTTCTCCCTCAAGATCAAGGTCTCTGTGTACAGGCAGCAGGAGGCACAA--
AAAGGGGTAATGGTATGCGAATGTTATGT------GAATATCCAAGTTTAGATTTCCCATTCCAAACA-
CCACACACAGTCTGACTAACCCTTGACAACAATAAACC-AACCCAT-GCAGCCTCTGCATGCCTGGGAAGCTG-
TATTACA-TAAGACTTGTC---AGTG--------GCTGGACACATGGGACGTA--CAATTTTAGAGCAAGCCCT-
AGAGGATCCATTGGAGACCAAAACCCATAAAACTAAGTGTTATACAAACACACGGCATGAATTTT-
TGTTCTTAATGGATGAGGC-
AGACAAAAAGTGGGAAGGGCCATGGTCTGCTCAAGGTCATGAAGCAGCTCAGTGTCGGAGCTAGGAATAGAGCCCAGTTC
TTGTGCCTAGCCCAGGTTCCTTGCATGCTAAACTTCACTGTCTCTCCAGAGACTGCACAGAGGGCTACTGAATTAGCTCA
GAGATATTTCCAGGCTCTGCAACCTATTTCTTAGTCTGGCTTTTCTGAAAATAAACCTTGAGTGTCCCTTCAG-TTTT-
TCTCTCTCTCTTTCCTTCCTCACAGTTCATTTCTG
Myiarchus CAAATATCTGGAGGTATGGAAAAGAGCAAG-GAATCTTGGTGTC-T--------
-GATGTGCAGTGAATGTGT--GTAAGA-CAGCTATGTGAGAGCTGTGCTTTTATTTAC-AATGACTAGTTGGACTTC-
AGTGAGTTCTCCCTCAAGATCAAGGTCTCTGTGTACAGGCAGCAGGAGGCACAA--
AAAGGGGTAATGGTATGCGAATGTTATGT------GAATATCCAAGTTTAGATTTCCCATTCCAAACA-
CCACACACAGTCTGACTAACCCTTGACAACAATAAACC-AACCCAT-GCAGCCTCTGCATGCCTGGGAAGCTG-
TATTACA-TAAGACCTGTC---AGTG--------GCTGGACACATGGGACATA--CAATTTTAGAGCAAGCCCT-
AGAGGATCCATTGGAGACCAAAACCCATAAAACTAAGTGTTATACAAACACACGGCATGAATTTT-
CGTTCTTAATGGATGAGGC-
AGACAAAAAGTGGGAAGGGCTATGGTCTGCTCAAGGTCATGAAGCAGCTCAGCGTCGGAGCTAGGAATAGAGCCCAGTTC
TTGTGCCTAGCCCAGGTTCCTTGCATGCTAAACTTCACTGTCTCTCCAGAGACTGCACAGAGGGCTACTGAATTAGCTCA
GAGATATTTCCAGGCTCTGCAACCTATTTCTCAGTCTGGCTTTTCTGAAAATAAACCTTGAGTGTCCCTTCAG-TTTT-
TCTCTCTCTCTTTCCTTCCTCACAGTTCATTTCTG
Hypocnemis_cantator
??GAATGTGT--
GCAAGA-CCGCTATGTGAGAGTTTGCTTTAATTTATTGATGACTAGTTGGACTTC-
TGTGAGTTCTTCCTCAAGTCCAAGGTCTCTGTGTACAGGC????????GCCCAG--

AAAGGGCTCATGGTACGTGAATGGTATGT------GAATATCCAAGTTTAGATTTCCCACTCCAAACA-
TCACACACAGTCTGACTAACCCTTGACAACAATAAACC-AACCCAG-GCAGCCTCTGCATGCCTGGGAAACTA-
TATTACA-TAAGACCTGTC---AGTG--------GCTGGACACATGGGATGTA--TAATTTTAGAGTAAACCCT-
GGAGGATCCATTGGAGACCAAGACCCATAAAACTAAGTGTTATACAAACATATGGCATGAATTTT-
CAGTCTTAATGGATGAGGA-
AGACAAAAAGTGGACAGGGCCATGGTCTTCTCAAGGTCATGAAGCAGATCAGCATCAGAGCTAGGAGAGGAGCCCAGTTC
TTCTGCATACCCCAGGCTCCTTGCATACTAAACCTCACTGTCTCTCCAGAGACTGCACGGAGGGCTACTGAATTAGCTCA
GAGATATTTCCAGGCTCTGCAACCTATTTCTTAGTCTGGCTTTTCTGAAAATAAACCTTGAGTGTCCCTTCAG-TTTT-
TCTCTCTCTCTTTCCTTCCTCACAGTTCATTTCTG
Phlegopsis   CAAATATCTGGAGGTATGGAAAAGGACAAG-RAATATTGGTGTC-T--------
-GGTGTGTAGTGAATGTGT--GCAAGA-CAGCTATGTGAGAGTTTTGCTTTAATTTATTGATGACTAGTTGGACTTC-
TGTGACTTCTCCCTCAAGTCCAAGGTCTCTGTGTACAGGCAGCAGGAGGCACAG--
AAAGGGCTCATAGTATGTGAATGGTATGT------GAATATCCAAGTTTAGATTTCCCATTCCAAACA-
TCACACACAGTCTGACTAACCCTGGACAACAATAAACC-AACCCAT-GCAGCCTCTGCATGCCTGGGTAACTA-
TATTACA-TAAGACCTGTC---AGTG--------GCTGGACACATGGGACATA--TAATTTTAGAGTAAACCCT-
GGAGGATCCATTGGAGACCAAGACCCATAAAACTAAGTGTTATACAAACATATGGCATGAATTTT-
CAGTCTTAATGGATGAGGA-
AGACAAAAGGTGGACAGGGCCATGGTCTTCTCAAGGTCATGAAGCAGATCAGCATCAGAGCTAGGAGAGGAGCCCATTTC
TTCTGTGTAGCCCAGGCTCCTTGCATACTAAACCTCACTGTCTCTCCAGAGACTGCACAGAGGGCTACTGAATTAGCTCA
GAGATATTTCCAGGCTCTGCAACCTATTTCTTAGTCTGGCTTKTCTGAAAATAAACCTTGAGCGTCCCTTCAGTTTTTCT
TTCTCTCT--TTCCTTCCTCACAGTTCATTTCTG
Manacus_manacus   CAAATATCTGGAGGTATGGAAAAGGGCAAG-GAATCTTGGTGTC-T--------
-GAAGCGCAGTGAATGTGT--GTAAGA-CATCTATGTGAGAGCTGTGCTTTTATTTACCAATGACTAGTTGGACTTC-
AGGGAGTTCTCCCTCAAGACCAAAGTCTYTGTGTACAGGCAGCAGGAGGCACAG--
AAAGGGCTAATGGCATGCGAATGGTATGT------GAATATCCAAATTTAGATTTCCCATTGCAAACA-
CCACACACAGTCTGACTAACCCTTGACAACAACAAACC-AACCCAT-GCAGCCTCTGCATGCCTGGGAAGCTG-
TATTACA-TAAGACCTGTC---AGTG--------GCTGGACACATGGGACATA--CAATTTTATAGGAAGCCCT-
AGAGGATCCACTGGAAAGCAAAACCCATAAAACTAAATATTATACAAACATACGGCATGAATTTT-
CGGTCTTAATGGATGAGGC-
AGACAAAAAGTGGGAAGGGCCATGGTCTGCTCAAGGTCATGAAGCAGATCAGTGTCAGAGCTAGGAATAGAGCCCAGTTC
TTCTGCCTAGGCCAGGTTCCTTGCATACTAAACTTCACTGTCTCTCCAGAGACTTCACAGAGGGCTACTGAATTAGCTCA
GAGATATTTCCAGGCTCTGCAACCTATTTCTTAGTCTGGTTTTTCTGAAAATAAACCTTGAGTGTCCCTTCAG-TTTT-
TCTCTCTCTCTTTCCTTCCTCACAGTTCATTTCTG
Acanthisitta_chloris   GAAATATCTGGAGGTATGGAAAAGGGCAGG-GAACCTTGATGTC-T--------
-GATGTGCAGCGAATGTGT--GCAAGA-TAACTATGTGAGAGTTGTCCTTTGATTTACTGATGACCAATTGGACTTC-
AGTGAGCTCTCCTTGAAGTCCAAGGTCTCTGTGTACATGCAGGAGGAGGCACAG--
AAAGAGCTCATGGTATTTGAATGGTATGC------GAATATCCAAGTTTAGATTTCCCATTCCAAACA-
CCACATGCAATCCGACCAACCCTTGACAACAATAAACC-TGTCCAT-GCATTCTCTGCATGGCTGGRAAACTA-
CATTACA-TAAGAGCTGCC---AGTG--------GCTGGACACAAGGGACGTA--CAATTTTAGAGCGAGCCCT-
GGAGGATCCATTGGAGACCAAGACCCATAAGACTAAGTGTTATACAAACATAGCTCATGAATTTT-
CAGTCTTAATGGATGAGGC-
AGACAAAAAGTGGGAAGGGCCATGGTCTGCTCAAGGTCATGGAGCAGATCAGCGTCAGAGCTAGGAATAGAGCCCGGTTC
TTCTGCCTAGCCCAGGCTCCTTGCATACTAGACCTCACTGTCTCTCCAGAGACTGCAGGAAGGGCTACTGAATTAGCTCG
AAGATATTTCCAGGCTCTGCAACCTATTTCTTAGTCTGGCTTTTCTGAAAATAAACCTTGAGTGTCCCTTCAT-TTTT-
TCTCTCTCTCTTTCCTTCCTCACAGTTCATTTCTG
Regulus   CAAATATCTTGAGGTATGGAAACGGGCAAG-GAGTCTCAGTGTT-T--------
-GATGTGTAGCAAATGTGTGGGCAAGA-CAGCTGTGTGAGGGTTGGGCTTTCATTTACTGATGACTAGTTGGACTTC-
ACTGAGCTCTCCCTCAAGTCCAAGGTCTGTGTGTACAAGCAGGAGGAGGCACAG--
AAAGGGCTCATGGTATGCAAATGGTATGTCCAGTGAAATATCCAAGTTYACATTTCCCATTCCAAACA-
CCACACACAGTCTGATCAACTCTTGACAACAATAAACC-AGCCAAT-GCAGCCTATGCATGCCTGGGAAACTG-
TATTACA-TAAGGACTGTC---AGTG--------ACTGGACACAAGGGACATA--CAATTTTAGAGTAAGCCCT-
GGAGGATCCATTGGAGACAAAGACCCATAAAACTACGTGTTATACAAGTACAGGGCATGAATTTG-
CATTCTTAATGGATAARGC-
AGACAAAAAGTGGAAAGGGCCGTCGTCTACTCAAGGTCATGAAGCAGATCAGCATCAGAGCTAGGAATAGAGCCCAGTGC
TTCTGCCTAGCCTAGGCTCCTTGCATACTAGACCTCACTGTCTCTCCAGAGGCTGCAGAAAGGCCTACTGAATTAGCTCA
GAGATATTTCCAGGCTCTGCAACCTATTTCTCAGTCTGGCTTTTCTGAAAATAAACCTTGAGTGTCCCTTCAG-TTTT-
TCTTTTTCTCTTTCCTTCCTCTCAGTTCATTTCTG
Ailuroedus   CAAATACCTGGAGGTATGGAAAAGGGCAGG-GAGTCTCTG?GTC-T--------
-AATGTCTAGTGAATGTGT--GCAAGA-TAGCTATGTGAGAGTTGTGCTTTTATTTACTGATGACTAGTTGGACTTC-
AGTGAGCTCTCCCTCAAGTCCACGTTGTGTGTGTACAAACAGGAGGAGGCACAG--

AAAGGGTTCATGGTATGCAAATGGTATGT------GAATATCCAAGTTTAGATTTCCCATTCCAAACA-
CCACACACAGTGTGACCAACCCTTGACAACAATAAACC-AGCCCAT-GCAGCCTATGCATGCCTGGGAAACAA-
TATTACG-TAAAGACTGTC---AGTGACTGACTGTCAGGACACAAGGGACATATGCAATTTTAGAGTAAGCCCT-
GGAGGATCCATTGGAGACCAAGACCCATAAAACTCAGTGTTATACAAACACAGGGCATGAATTTG-
CACTCTTAATGGATGAGGC-
AGACAAAAAGTGGGAAGGGCCATGGTCTACTCAAGGTCGTGAAGCAGATCAGTGTCAGAGCTAGGAACAGAGCCCAGTGC
TTCTGCCTAGCCCAGGCTCCTTGCATACTAGACCTCACTGTCTCTCCAGAGACTGTGGGAAGGGCTACTGAATTAGCTCA
GAGATATTTCCAGGCTCTGCAACCTATTTCTTAGTCTGGCTTTTCTGAAAATAAACCTTGAATGTCCCTTCAG-TTTT-
TCTTTCTCTCTTTCCTTCCTCACAGTTCATTTCTG
Zosterops TAAATATCTGGAGGTATGGAAAAGGGCAGG-GAGTTTTGGTGTC-T--------
-GATATGTAGTGAATGTGT--GCAAGA-CAGCCATGTGAGAGTTTGGCTTTCATTTACTGATGACTAGTTGGACTTC-
AGTGAG-TCTCCCTCAAGTCCAAGGTCTGTGTGTACAAGCA---GGAGACCCAG--
AAAGGGCTCATGGTATGCAAATGGTATGT------GAATATCCAAGTTTAGATTTCCCATTCCAACCA-
CCACACATAGTCTGAACAACCCTTGACAACAATAAACC-AGCCCAT-ATAGCCTATGCATGCCTGGGAAACTG-
TATTACA-TAAGGACTGTC---AGTGCACAGTGCACAGGACACAAGGGACATA--CAATTTTAGAGTAAATCCT-
GGAGGATCCATTGGAGACCAAGACCCATAAAACTAAGTGTTATACAAACACAGGGTATGAATTTG-
CAGTCTTAATGGATGA-GC-
AGACAAAAAGTGGAAAGGGCCATGGTCTACTCAAGGTCATGAAGCAGATCAATGTCGGAGCTAGGAATAGAGCCCAGTTC
TTCTGCCTAGCCCAGGCTCCTTACATACTAGACCTCACTGTCTCTCCAGAGGCTGCAGGAAGACCTACTGAATTAGCTCA
GAGATATTTCCAGGCTCTGCAACCTATTTCTTAGTCTGGCTTTTCTGAAAATAAACCGTGAGTGTCCCTTCAG-TTTT-
TCTTTCTCTCTTTCCTTCCTCTCAGTTCATTTCGG
Onychorhynchus CAAATATCTGGAGGTATGGAAAAGGGCAAG-GAATCTTGGCGTC-T--------
-GATGTGCAGTGAATGTGT--GTAGGA-CAGCTATGTGAGAGCTCTGCTTTTCTTTACTGATGGCTGGTTGGGCTTC-
AGTGAGTTCTCCCTCAAAACCAAGGTCTCTGTGTACAGGCAGCAGGAGGCACAG--
AAAGGGCTAATGGTATGCGAATGTTATGT------GAATATCCAAGTTTAGATTTCCCATTCCAAACA-
CCACACACAGTCTGAATAACCCTTGACAACAATAAACC-AACCCAT-GCAGCCTCTGCATGCCTGGGAAGCTG-
TATTATA-TAAGACCTGTC---AGTG--------GCTGGACAAATGGGACATA--CAATTTTAGAGTAAGCCCT-
GGAGGATCCATTGGAGACCAAAACCCATAAAACTAAGTGTTATACAAATACACGGCATGAATTTT-
CGGTCTTAATGGATGAGGC-AGACAGAA-
GTGGGAAGGGCCATGGTCTACTCAAGGTCATGAAGCAGATCAGCATCAGAGCTAGGAATAGAGCCCAGTTCTTCTGCCTA
GCCCAGGTTCCTTGCATACTAAACTTCACTGTCTCTCCAGAGACTGCATGGAGGGCTACTGAATTAGCTCAGAGATATTT
CCAGGCTCTGCAACCTATTTCTTAGTCTGGCTTTTCTGAAAATAAACCTTGAGTGTCCCTTCAGTTTTTCTCTCTCTCTC
TTTCCTTCCTCACAGTTCATTTCTG
Nestor_notabilis
??
??
??
??
??
??
??
??
??
??
Leiothrix_argentauris CAAATATCTGGAGGTATGGAAAAGGGCAGG-GAGTCTCAGTGTC-T--------
-GATATGTAGTGAATGTAT--GCAAGA-CAGCCATGTGAGAGTTGGGCTTTCAATTACTGATGACTAGTTGGACTTC-
AGTGAGCTC--
ATGGTATGCAAATGGTATGT------GACTATCCAAGTTTAGATTTCCCATTCCAACCA-
CCACACACAATCTGACCAACCCTTGACAACAATAAACT-AGCCCAT-GCAGCCTATGCATGCCTGGGAAACTG-
TATTATA-TAAGGACTGTC---AGTG--------ACTGGACACAAGGGACATA--CAATTTTAGAGTAAACCCT-
GGAGGATCCATTGGAGACCAAGACCCATAAAACTAAGTGTTATACAAACACAGGGTATGAATTTG-
CAGTCTTAACGGATGA-GC-
AGACAAAAAGTGGAAAGGGCCATGGTCCACTCAAGGTCATGAAGCAGATCAACGTCAGAGCTAGGAATAGAGCCCAATGC
TTCTGCCTAGCCCAGGCTYCTTACATACTAGACCTCACTGTCTCTCCAGAGGCTGCAGGAAGGCCTACTGAATTAGCTCA
GAGATATTTCCAGGCTCTGCAACCTATTTCTTAGTCTGGCTTTTCTGAAAATAAACCTTGAGTGTCCCTTCAG-TTTT-
TCTTTCTCTCTTTCCTTCCTCTCAGTTCATTTCGG
Troglodytes CAAATATCTGGAGGTATGGAAAAGGGCAGG-GAGTCTCAGTGTC-TA-------
-GATGTGTAGTAAATGTGT--GCAA---CAGCTATGGGAGGGTTGGGCTTTCATTTACTGATGACTAGTTGGGCTTT-
ATTGAGCTTCTCCTCAAGTCCGAGGTCTGTGTATACAAGCAGGAGGAGGCACAG--AAAGGGCTCATGGTATGT------
-----------GAATATCCGAGTTTAAATTTCCCATTCCAAACA-

CCACGCACAGTCTGACCAGCCCTTGACAACAACAAACC-AGCCCAT-GCAC---------GCCTGGGAAACTG-
TATTACA-TAAGGACTGTC---AGTG--------ACTGGACACAGGGGACATA--CAATTTTAGAGTAAGCCCT-
GGAGGATCCATTGGAGACTTAGACCCATAAAACTAAGTGTTATACAAACACAGGGCATGAATTTG-
CAGTCTTAATGGATGAGGC-
AGACAAAAAGTGGAAAGGGCCATGGTCTACTCAAGGACATGAAGCAGATCAGCGTCAGAGCTAGGAATAGAGCCCAGTGC
TTCTGCCTAGCCCAGGCTCCTTGCATCCTAGACCTCTCTGTCTCTCCAGAGGCTGCAGGAAGGCCTACTGAATTAGCTCG
GAGATATTTCCAGGTTCTGCAACCTATTTCTTAGTCTGACTTTTCTGAAAATAAACCTGTAGTGTCCCTTCAGTTTTTCT
TTCTCTCT---TCCTTCCTCTCAGTTCATTTCTG
Luscinia_svecica ????????????GTATGGAAAAAGGCAGG-GAGTCTCAGTGTC-T--------
-GATGTCTAGTGAATGCAT--GCAAGA-CAGCTGTGTGAGAGTTGGGCTTTCATTTACTGATGGCTAGTTGGACTTC-
CGTGAGCTCTCCCTCAAGTCCAAGGTCTGTGTGTAAAAGTAGGAGGAAGCACAG--
AAAGGGCTCATGGCATGTAAATGATATGT------GAATATCCAAGTTTAGATTTCCTATTCCAAACA-
CCACACACAGTCTGACCAACCCTTGACAACAATAAAGC-AGCCCAT-GCAGCCTAAGCATGCCTGGGAAACTG-
TATTACA-TGAGGACTGTC---AGTG--------ACCGAACACAAGGGACATG--CAATTTTAGAGTAAACCCT-
GGAGAATCCATTGGAGACCAAGACCCATAAAACTAAGTGTTATACAAACACAGGGCATGAATTTG-
CAGTCTTCATGGATGAGGC-
AGACAAAAAGTGGAAAGGGCCATGGTCTATTCAAGGTCATGAAGCAGATCAATGTCAGAGCTATGAATA-
AGCCCAGTGCTTCTGCCTAGCCCAGGCTCCTTGCATACTAGACCTCACTGTCTCTCCAGAGGCTGAAGGAAGGCCTACTG
AATTAGCTCGGAGATATTTCCAGGCTCTGCAACCTATTTCTTAGTCTGGCTTTTCTGAAAATAAACCATGAGCGTCCCTT
CAG??
Psittacus_erithacus CAAATACCTGGAGGTATGGAAAAGGGCAGG-GAACCTTCTTGTC-T--------
-GATGTGTAGTGAATGTGT--GCAAGC-CGGCTATGTGAGAGTTGCCCTTT-ATTTACTGATGGCTAGTTGGACTTC-
AGTGAGCTCTCCCTCAAGTCCAGTGTCTCTGTGTACAGGCAAGAGGAGGCATGG--
AAAGGGCTTATGGTATGTGAATGGTATGT------GACTATCCAAGTTTAGATTTCCCATTCCAAACA-
CCACACRCAGTCTGACCAACCCTTGACAATAATAAACC-AGCCTAT-GCAGCCTCTGCATGCCTGGGAAACTG-
CATTACA-TAACAGCTGTC---AGTG--------ACTGGATAGCAGGGACATA--CAGTTTTAGAGTAAGCCCT-
GGGGGATCTATTGGAGACCAAGACCCACAAAAGTAAGTGT---ACAAACACAGGGCATGAATTTT-
CATCCTTAATGGATTAGGC-
AGACAAAGAGTAGGAAGGTCCACAATCTATTCAAGGTCAGGAAGTAGATCAGTGTCAGAGCTAGGAATAGAGTCCAGTTC
TTCTGCCCAGC-
TAARCTCCTTGCATGCTAGACCTCACTGTCTCTCCAGAGACTGTGGGAAGGGCTACTGAATTAGCTTGGAGATATTTCCA
GGCTCAGCAACCTATTTCTTAGTCTGGCTTTTCTGAAAATAAACCCTGAGTGTCCCTTCAGCTTTTCTCTCTCTCTCTTT
CCTTCCTCACAGTTCATTTCCG
Ficedula_hypoleuca CAAATATCTGGAGGTATGGAAAAGGGCAGG-GAGTCTCAGTGTC-T--------
-GATGTCTAGTGAATGCAT--GCAAGA-CAGCTATRTGAGAGTTGGGCTTTCATTTACTGATGACTAGTTGGACTTC-
AGTGAGCTCTCCCTCAAGTCCAAGGTCTGTGTGTAAAAGTAGGAGGAGGCACAG--AAAGGGCTCAAGG---
GCGAATRGTATGT------GAATATCCAAGATTAGATTTCCCATTCCAAACA-
GCACATACAGTCTGACCAGCCCTTCACAACAATAAACC-AGCCCAT-GCAGCCTATGCGTGCCTGGGAAACTG-
TATTACA-TAAGGACTGTC---AGTG--------ACCAGACACAAGGGACATG--CAATTTTAGAGTAAACCCT-
GGAGAATCCATTGGAGACCAAGACCCATAAAACTAAGTGTTACACAAACACAGGGCATGAATTTG-
CAGTCTTAATGGATGAGGC-
AGACAAAAAGTGGAAAGGGCCATGGTCTACTCAAGGTCATGAAGCAGATCAGTGTCAGAGCTATGAATA-
AGCCCAATGCTTCTGCCTAGCCCAGGCTCCTTGCATACTAGACCTCACTGTCTCTCCAGAGGCTGAAGGAAGGCCTACTG
AATTAGCTCGGAGATATTTCCAGGCTCTGCAACCTATTTCTTAGTCTGGCTTTTCTGAAAATAAACCATGAGCGTCCCTT
CAG-TTTT-TCTTTCTCTCTTTCCTTCCTCGCAGTTCATTTCTG
Menura_novaehollandiae CAAATATCTGGAGGTATGGAAAAGGGCAGG-GAGTCTCAGTGTCTT--------
-GATGTGTAGTGAATGTGT--GCAAGA-CAG?----GGAGAGTTGTGCTTTTATTTACTGATGACTAGTTGGACTTC-
AGTGAGCTCTCCCTCAAGTCCAGGGCCTGTGTGTACAAGCAGGAGGAGGCACAG--
AAAGGGCTCATGGTATGCAAATGGTATGT------GAATATCAAAGTTTAGACTTCCCATTCCAAACA-
CCACACACAGTCTGACCAACCCGTGACAACAATAAACC-AGACCAT-GAAGCCTATGCATGCCTGGGAAACTA-
TATTACA-TAAGAACTGTCACAAGTG--------ACGGAACACAAGAGACATA--
CCATTTTACAGTAAGCCCTAGGAGGATCCATTGGAGACCAAGACCCATAAAACTAAGTGTTATACAAGCACAGGCCATGA
ATTTG-CAGTCTTAATGGATGAAGC-
AGACAAAAAGTGGGAAGGGCTATGGTCTACTCAAGGTCATGAAGAAGATCAGCGTCAGAGCTAGGAATAGAGCCCAGTTC
CTCTGCCTAGCCTGGGCTCCTTGCATACTAGACCTCACTGTCTCTCCAGAGACTGT?G?AAGGGCTACTGAATTAGCTTG
GAGATATTTCCAGGCTCTGCAACCTATTTCTTAGTCTGGCTTTTCTGAAAATAAACCTTAAGTGTCCCGTCAG-TTTT-
TCTTTCTCTCTTTCCTTCCTCACAGTTCATTTCTG
Pycnonotus CAAATATCTGGAGGTATGGAAAAGGGCAGG-GAGTCTTAGTGTC-T--------
-GATGTGTAGTGAATGTGT--GCAAGA-CAGCTATGTGAGAGTTGGGCTTTCATTTACTGGTGACTAGTTGGACTTC-
AGTGAGCTCTCCCTCAAGTCCAAAGTCTGTGTACACAAGCA---GGAGGCACAG--

AAAGGGCTCATGGCATGCAAATGGTATGT------GAATATCCAAGTTTAGATTTCCCATTCCAGCCA-CCAC----
AGTCTGACCAACCCTTGGCAACAATAAACC-TGCCCAT-GCAGCCTATGCATGCCTGGGAAACTG-TATTACA-
TAAGGACTGTC---AGTG--------ACTGAACACAAGGGACATA--CAATTTTAGAGTAAACCCT-
GGAGGATGCATTGGAGACCAAGATCCATAAAACTAAGTGTTATACAAACACAGGGCATGAATTTG-
CAGCCCTAATGGATGAGGC-
AGACAAAAAGTGGAAAGGGCCATGGTCTACTCAAGGTCATGAAGCACATCAGCGTCAGAGCTAGGAATAGAGCCCAGTGC
TTCTGCCTAGCCCAGGCTCCTTACATACTAGACCTCACTGTCTCTCCAGAGGCTGCAGGAAGGCCTACTGAATTAGCTCA
GAGATATTTCCAGACTCTACAACCTATTTCTTAGTCTGGCTTTTCTGAAAATAAACCTTGAGTGTCCTTTCAG-TTTT-
TCTTTTTCTCTTTCCTTCCTCTTAGTTCATTTCTG
Donacobius_atricapilla   TAAATATCTGGAGGTATGGAAAAGGGCAGG-GAGTCTCAGTGTC-T--------
-GATATGTAGTGAATGTGT--GCAAGA-CAGATGTGTGAGGGTTAGGCTTTCATTTACTCATGACTAGTTGGATTTC-
AGTGAGCACTCCCTCAAGTCCAAGTTCTGTRTATACAAGCA---GGAAGCACAG--
AAATGGCTCATGGTATGCAAATGGTATGT------GAATATCCAAGTTTAGATTTCCCATTCCAACCA-
CCACACACAGTCTGACCAACCTTTGACAACAATAAACC-AGCCCAT-GCAGCCTACACATGCCTGRGAAACTG-
TATTACA-TGAGGACTGTC---AGTG--------ACTGGACACAAGGGACATA--CAATTTTAGAGTAAACCCT-
GGAGGATCCATTGGAGACCAAGACCCATAAAACTAAGTGTTATACAAACACAGGGCATGAATTTG-
CAGTCTTAATGGATGAGGC-
AGACAAAAAGTGGGAAGGGCCATGGTCTACTCAAGGTCAAGAAGCAGATCAGCATCAGAGCTAGGAATAGAGCCCAGTGC
TTCTGCCTAGCCCAGGCTCTTTACATACTAGACCTCACTGTCTCTCCAGAGGCTGCAAGAGGGCCTACTGAATTAGCTCA
GAGATATTTCCAGGCTTTGCAACCTATTTCTTAGTCTGGCTTTTCTGAAAATAAACCTTGAGTGTCCCATTAG-TTTT-
TCTTTCTCTCTTTCCTTTCTCTCAGTTCATTTCTG
Hirundo_rustica   CAAATATCTGGAGGTACGGAAAAGGGCAGG-GAGTCTTAGTGTC-T--------
-GATGTGTAGTGAATATGT--GCAAGA-CAGCTATGTGAGAGTTGGGCTTTCATTTATTGATGACTAGTTGGACTTC-
AGTGAGCTCCCCCTCAAGTCCAAGGTCTGTGTGTACAAACA---GGAAGCACAG--
AAAGGGCTCACGGTATGCAAATGGTATGT------GAATATCCAAGTTTAGATTTCCCATTCCAGCCA-CCAC----
AGTCTGACCAACCCTTGACGACAATAAACC-AGCCCAT-GCAGCCTATGCCTGCCTGGGAAACTG-TATTACA-
TAAGGACTGTC---AGTG--------ACTGGACACAAGGGACATA--CAATTTTAGAGCAAACCCG-
GGAGGATCCATTGGAGACCAAGACCCACAAAACTAAGTGTTATACAAACAGAGGGCATGAATTTG-
CAGTCTTAATGGATGAGGM-AGACAAAAAGTGGAAAAGGC-
ATGGTCTACTCAAGGTCATGAAGCAGATCAGCGTCAGAGCTAGGAATAGAGCCCAGTGCTTCTGCCTAGCCCAGGCTCCT
TACATAGTAGACCTCACTGTCCCTCCAGAGGCTGCAGGAAGGCCTACTGAATTAGCTCAGAGATATTTCCAGGCTCTGCA
ACCTATTTTTAGTCTGGCTTTTCCGAAAATAAACCTTGAGTGTCCCTTCAG-TTTT-
TCTTTCTCTCTTTCCTTCCTCTCAGTTCATTTCTG
Lichenostomus   CAAATATCTGGAGGTATGGAAAAGGGCAGG-GAGTCTCAGTGTC-T--------
-GATRTGTAGTGAATGTGT--GCCAGA-CAGCTATRTGAGAGC-ATGCCTTTACTTACTGATGACTAGCTGGACTTC-
AGTGAGCTTTCCCTCAAGTCCAAGGTCTGTGTGTACAAACAGGAGGAGGCACAG--
AAAGGGCTCATGGTATGCAAATAGTATGT------GACCATCCAAGTTTAGATTTCCCATTCCAGACA-
CCACATATAGTCTGACTAGCCCTTGACAACAACAAACC-AGCCCAT-GCAGCCTGTGCATGCCTGGGAAACTG-
TATTRCA-TAAGGACTGTC---AGTG--------ACTGGACRCAAGGGACGCA--CAATTTTAGAGTAAGCCCT-
GGAGGATCCATTGGAGACCAAGACCCATAAAACTAAGTGTTACACAAACACATGGCATGAATTTG-
CAGTCTTAATGGATGAGGC-
AGATAAAAAGTGGGAAGGGCCATGGTCTACTCAAGGTCGTGAAGCAGATCAGYGTCAGAGCTAGGAATAGAGCCCAGTGC
TTCTGCCTAGCCCAGGCTCCTTGGATACTAGACCTCACTGTCTCTCCAGAGACTCTAGGAAGGGCTACTGAATTAGCTCG
GAGATATTTCCAGGCTCCGCAACCTATTTCTTAGTCTGGCTTTTCTGAAAATAAACCCTGAGTGTCCCTTCAG-TTTT-
TCTTTCTCTCTTTCCTTCCTCACAGTTCATTTCTG
Cnemophilus   ????????????GTATGGAAAAGGGCAGG-G-GTCTCAGTGTT-T--------
-GATGTGTAGTGAATGTGT--GCAAGA-CAGCTAT-TGAGAGTTGGGCTTTTATTTACTGATGTCTAGTTGGACTTC-
AGTGAGCTCTCCCTCAAGTCCAAGGTTTGTGTGTACAAGCAGGAGGATGCACGG--
AAAGGGCGCATGGTATGCAAATGGTATGT------GAATATCCAAGTTTAGATTTCCTATTCCCAACA-
CTACACACAGCCTGACCAACCTCTGACAACAACAAACC-AGCCCAT-GCAGCCTATGGATGCCTGGGAAACTG-
CATTACA-TAAGGCCTGTC---AGTG--------ATTGGACACAAGGGACATA--CAATTTTAGAGTAAGCCCT-
GGAGGATCCGTTGGAGACCAAGACCCATAAAACTAAGTGTTATACAAACACAGGGCATGAATTTG-
CAGTCTTAATGGATGAGGC-
AGACAAAAAGTGGGAAGGCCCATGGTCTAYTCAAGGTCATGAAACAGATCAGCGTCAGAGCTAGGAATAGAGCCCAGTGC
TTCTGCCTAGCCCAGGCTCCTTGCATACTAGACCTCACTGTCTCTCCAGAGACTGCAGGAAGGGCTACTGAATTAGCTCG
GAGATATTTCCAGGCACTGCAACCTATTTCTTAGTCTGGCTTTTCTGAAAATAAACCTTGAGTGTCCCTTCAG-TTTT-
TCTTTCTCTCTTTCCTTCCTCACAGTTCATTTCTG
Coracina   ????????????GTATGGAAAAGCGCAGG-GAGTCTCAGTATC-T--------
-GATGTATAGTGAATGTGT--GCAAGA-CAGCTATGTGAGAGTTGTGCTTTTATTTACTGATGACTAGTTGGACTTC-
AGTGAGCTTTCCCTCAAGTCCAAGGTCTGTGTGTACAAGCAGGAGGAGGCACAG--

AAAGGGCTCATGGTATGCAAATGGTATGT------GAAAATCCAAGTTTAGATTTCCCATTCCAAACA-
YCACAGACAGTCTGACCAGCTCTTGACAACACTAAACC-AGCCCAT-GCAGCCTATGCATGCCTGGGAAACTG-
TATTACA-TAAGGACTGTC---AGTG--------ACTGGACACAAGGGACAAA--CAGTTTTAGAGTAAGCCCT-
GGAGGATCCACTGGAGRCTGAGACCCATAAAACGAAGTGTTATACAAACACAGGGCATGAATTTG-
CAGTCTTCATGGATGAGGC-
AGACAAAAAGTGGGAGGGGCCACGGTCTACTCAAGGTCATGAAGCAGATCAGCATCAGAGCTAGGAATAGAGCCCAGTGC
TTCTGCCTAGCCCAGGCTCCTTGCATGCTAGACCTCACTGTCTCTCCAGAGACTGCAGGAAGGGCTACTGAATTAGCTCG
GAGATATTTCCAGGCTCTGCAACCTATTTCTTAGTCTGGCTTTTCTGAAAATAAACCTTGAGTGTCCCTTCAG-TTTT-
TCTTTCTCTCTTTCCTTCCTCACAGTTCATTTCTG
Dicrurus CAAATATCTGGAGGTATGGAAAAGGGCAGG-GAGTCTCAGTGTC-T--------
-GATGTGTMGTGAATGTGT--GCAAGA-CAGCTATGTGAGAGTTGTGCTTCTATTTACTGATGACTTGTTGGACTTC-
AGTGAGCTCTCCCTCAAGTCCAAGGTCTGTGTGTACAAGCAGGAGGAGGCACAG--
AAAGGGCTCATGGTATGCAAATGGTATGT------GAATATCCAAGTTTAGATTTCCCATTCCAAACA-
CCACACACAGTCTGACCAGCCCTTGACAACAATAAACC-AGCCCAT-GCAGCCTATGCATGCCTGGGACGCTG-
TGTTACA-TAAGGCCTGTC---AGTG--------ACTGGACACGAGGGACATA--CAATTTTAGAGTAAGCCCT-
GGAGGATCCATTGGAGACCAAGACCCATAAAACGAAGTGTTATACAAACACAGGGCATGAATTTG-
CAGACTTAATGGATGAGGC-
AGACAAAAAGTGGGAGGGGCCATGGTCTACTCAAGGTCATGAAGCAGATCAGCGTCAGAGCTAGGCATAGAGCCCAGTGC
TTCTGCCTAGCCCAGGCCCCTTGCATACTAGACCTCACTGTCTCTCCAGAGGCTGCAGGAAGGGCTACTGAATTAGCTCA
GAGATATTTCCAGGCTCTRCAACCTATTTCTTAGTCTGGCTTTTCTGAAAATAAACCTTGAATGTCCCTTCAGTTTTTCT
TT----CTCTTTCCTTCCTCACAGTTCATTTCTG
Gerygone_fusca CAAATATCTGGAGGTATKGAAAAGGGCAGG-GAGTCTCAGTGTC-T--------
-GATGTGTAGTGAACGTGT--GCAAGA-CAGCTATGTGAGAATTGTGCTTTTATTTCCTGCTGACTAGTTGGACTTC-
AGTGAGCTCTCCCTCAAGTCCAAGGTCTGTGTGTACAAGCAGGAGGAGGCACAG--
AAAGGGCTCATGGTATGCAAACAGTATGT------GAACATCCAAGTTTAGATTTCCCGTTCCAAACA-
CCWYGTACAGTCTGACCAGCCCYTGACAACAATAAACC-AGCCCAT-GCAGCCTAGGCATGCCTGGGAAACTG-
TATTACA-TAAGGACTGTC---AGTG--------ACTGGACACAAGGGACATA--CAATTTTAGAGTAAGCCCT-
GGAGGATCCATGGGAGACCAAGACCCATAAAACTAAGTGTTATACAAACACATGGCATGAATTTG-
CAGTCTTAATGGATGAGGC-
AGACAAAAAGTGKGAAGGGCCATGGTCTACTCAAGGTCGTGAAGCACATCAGCGTCAGAGCTAGGAATAGAGCCCAGTGC
TTCTGCCTAGCCCAGGCTCCTTGCATACTAGACCTCACTGTCTCTCCAGAGACTGCAGGAAGGGCTACTGAATTAGCTCG
GAGATATTTCCAGGCTCYGCAACCTATTTCTTAGTCTGGCTTTTCTGAAAATAAACCTTGAGTGTCCCTTCAGTTTTT-
TCTTTCTCTCTTTCCTTCCTCACAGTTCATTTCTG
Ptiloris_magnificus CAAATATCTGGAGGTATGGAAAAGGGCAGG-GAGTCTCAGTGTC-T--------
-GATGTGTAGTGAATGTGT--GCAAGA-CAGCTACGTGAGAGTTGTGCTTTTATTTACTGATGACTAGTTGGACTTC-
AA----------CTCAAGTCCAAGGTCTGTGTGTACAAGCAGATGGAGGCACAG--
AAAGGGTTCATGGTATGCAAATGGTATGT------GAATATCCAAGTTTAGCTTTCCCATTCCAGACA-
CCACACACAGTCTGACCAGCCCTTGACAACAATAAACC-AGCCCAT-GCAGCCTATGCATGCCTGGGAAACTG-
TATTACA-TAAGGCCCGTC---AGTG--------ACTGGACACAAGGGACATA--CAATTTTAGAGTAAGCCCT-
GGAGGATCCATTGGAGACCAAGACCCATAAAACTAAGTGTTATACAAACACAGGGCATGAATTTG-
CAGACTTAATGGATGAGGC-
AGATGAAAAGTGGGAGGGGCCATGGTCTACTCAAGGTCATGAAGCAGATCAGCGTCAGAGCTAAGCATAGAGCCCAGTGG
TTCTGCCTAGCCCAGGCTCCTTGCATACTAGACCTCACTGTCTCTCCAGAGGCTGCAGGAAGGGCTACTGAATTAGCTCG
GAGATATTTCCAGGCTCTGCAACCTATTTCTTAGTCTGGCTTTTCTGAAAATAAACCTTGAGTGTCCCTTCAGTTTTTCT
TT--CTCTCTTTCCTTCCTCACAGTTCATTTCTG
Paradisaea_raggiana
??
??
??
??
??
??
??
??
??
??
Cyanocorax_chrysops CAAATATCTGGAGGTATGGAAAAGGGCAGG-GAGTCTCAGTGTC-T--------
-GATGTGTAGTGAATGTGT--GCAAGA-CAGCCATGTGAGAGTTGTGCTTTTATTTACTGATGACTAGTTGGACTTC-
AGTGAGCTCTCCCTCACGTCCAAGGTCTGTGTGTACAAGCAGGAGGAGGCACAG--
AAAGGGCTCATGGTATGCAAACGGTACAT------GAATATCCAAGTTTAAA-TTCCCATTCCAAACA-

CCACACACAGTCTGACCAGCCCTTGACAACAATAAACC-AGCCCAT-GCAGCCTACGCATGCCTGGGAAACTC-
TATTACA-TAAGGCCTGTC---AGTG--------ACCRGACACAAGGGACATA--CAATTTTAGAGTAAGCCCT-
GGAGGATCCATTGGAGACCAAGACCCATAAAACTAAGTGTTATACAAACACAGGACATGAATTTG-
CAGACTTAATGGATGAGGC-
AGACAAAAAGTGGGAGGGGCCATGGTCTACTCAAGGTCATGAAGCAGATCAGCATCAGAGCTAGGCGGAGAGCCCAGTGC
TTCTGCCTAGCCCAGGCTCCTTGCATACTAGACCTCACTGTCTCTCCAGAGGCTGCAGGGAGGGCTACTGAATTAGCTCG
GAGATATTTCCAGGCTCTCCAACCTATTTCTTAGTCTGGCTTTTCTGAAAATAAACCTTGAGTGTCCCTTCAG-TTTT-
TCTTTCTCTCTTTCCTTCCTCACAGTTCATTTCTG
Lonchura
??
??
??
??
??
??
??
??
??
??
Phylloscopus   CAAATACCTGGAGGTATGGAAAAGGGCAGG-GAGTCTTAGTGTC-T--------
-GATGTGTAATGAATGTGT--GCAAGA-CAGCTATGTGAGAGTTGGGCTTTCATTTACTGATGACTAGTTGCACTTC-
AGTGAGCTCTCTCTCAAGTCCAAGGTCTGTGTGTACAAACA---GAAGGCACAG--
AACGGGCTCATGGTATGCAAATGGTATGT------GAATATCCAAGTTTAGATTTCCCTTTCCAAACA-CCAC----
AGTCTGGCCAACCCTTGACAACAATAAACC-AGCCCAT-TTAGCCTGTGCATGCCTGGGAAACTG-TATTACA-
TAAGGACTGTC---AGTG--------ACTGGACACAAGGGACATA--CAATTTTAGAGTAAACCCT-
GGAGGATCCATTGGAGACCAAGACCCATAAAACTAAGTGTTATACAAACATAGGGCATGAATTTG-
CAGTCTTAATGGATGAGGC-
AGAGAAAAAGTAGAAAGGGCCATGGTCTACTCAAGGTCATGAAGCAGATGAGCGTCAGAGCTAGGAATAGAGTCCA-
TGCTTTTGCCTAGCCCAGGCTTCTTACATACTAGACCTCACTGTCTCTCCAGAGGCTGCAGGAAGGCCTACTGAATTAGC
TCAGAGATATTTCCAG-CTCTGCAACCTATTTCTTAGTCTGGCTTTTCTGAAAATAAACCTTGAGTGTCCCTTCAG-
TTTT-TCTTTCTCTCTTTCCTTCCTCTCAGTTCATTTCTG
Turdus   ?????????????GTATGGAAAAGGGCAGGAGAGTCTCAGTGTC-T--------
-GATGCATAGTGAATGCAT--GCAAGA-CAGCTATGTGAGAGTTGGACTTTCATTTACTGATGACTGGTTGGACTTC-
AGTGAGCTCTCCCTCAAGTCCAAGGTCTGTGTGTAAAAGTAGGAGGAGGCACAG--
AAAGGGTTCATGGCATGCAAATAGTATGT------GAATATCCAAGTTTAGATTTCCCATTCCAAACA-
CCACACACAGTCTGACCAACCCTTGACAACAATAAACC-ATCCCAT-GCAGCCTATGCATGCCTGGGAAACTG-
TATTACA-TAAGGACTGTC---AGTG--------ACCGGACACAAGGGACATG--CAATTTTAGAGTAAACCCT-
GGAGAATCCATTGGAGACCAAGACCCATAAAACTAAGTGTYATACAAACACAGGGCATGAATTTG-
CAGTCTTAATGGATGAGGC-
AGACAAGAAGTGGAAAGGGCCATGGTCTACTCAAGGTCATGAAGCAGATCAGCATCAGAGCTATGAATA-
AGCCCAGTGTTTCTGCCTAGCCCAGGCTCCTTGCATACTAGACCTCACTGTCTCTCCAGAGGCTGAAGGAAGGCCTACTG
AATTAGCTCGGAGATATTTCCAGGCTCTGCAACCTATTTCTTAGTCTGGCTTTTCTGAAAATAAACCATGAGTGTCCCTT
CAG-TTTT-TCTTTCTCTCTTTCCTTCCTCTCAGTTCATTTCTG
Acrocephalus   ?????????????GTATG-AAAAGGGCAGG-GAATCTCAGTGTC-T--------
-GATATGCAGTGAATGTGT--GCAAGA-CAGCTATGTGAGAGTTAGGCTTTCATTTACTGATGACTAGTTGGACTTC-
AGTGAGCTCTCCCTCAAGTCCAAGGTCTGTGTATACAAGCAGGAGG---CACAG--
AAAGGGCTCATGGTATGCAAATTGTATGT------GAATATCCAAGTTTAGATTTCC------AACCA-
CCACACACAGTCCGACCAGTCTTTGACAACACTAAACC-AGCCCAT-GCAGCCTATGCATGCCTGGGACACTG-
TATTACA-TAAGGACTGTT---GGTG--------ACTGGGCACAAGGGACATA--CAATTTTAGAGTAAATCCT-
GGAGGATCCATTGGAGACCAAGACCCATAAAATTAAGTGTTATACAAACACAGGGAATGAATTTG-
CAGTCTTAATGGACGAGGC-
AGACAAAAAGTGGAAAGGGCCATGGTCTACTCAAGGTCATGAAGCAGATCAGTGTCAGAGCTAGGAACAGAGCCCAGTGC
TTCTTCCTAGCCCAGGCTCCTTACATACTAGACCTCACTGTCTCTCCAGAGGCTGCAGGAGAGCCTACTGAATTAGCTCA
GAGATATTTCCAGGCTCTGCAACCTATTTCTTAGTCTGGCTTTTCTGGAAATAAACCTTGAGTGTTCCTTCAGTTTTTCT
TT--CTCTCTTTCTTTTCTCTCAG??????????
Sitta   CAAATATCTGGAGGTATGGAAAAGAGCAGG-GAGTCTCAGTGTC-T--------
-GATGTGTAGTGAATGTGT--GCAAGG-CAGCTATGTGAGAGTTGGGCTTTCATTTACTGATGACTAGTTGGACTTC-
AGTGAGCTCTCCCTCAAGTCCAAGGTCTGTGTATACAAGCAGGAGGAGGCACAA--
AAAGGGCTCATGGTATGCAAATGGTATGT------GAATATCCAAGTTTAGATTTCCCATTCCAAACA-
CCACATGCAGTCTGACCAACCCTTGACAACAATAAACC-AGCCCCT-GCAGCCTATGCATGCCTCAGAAACTG-

TATTACA-TAAGGACTGTC---AGTG--------ACTGGACACAGGGGATATC--CAATTTTAGAGTAAGCCCT-
GGAGGATCCATTGGAGACCAAGACCCATAAAACTAAGTGTTATACAAACACAGCGCATGAATTTG-
CAGTCTTGATGGATGAGGC-
AGACAAAAAGTGGAAAGGGCCATGGTCTACTCAAGGTCAGGAAGGAGATCAGCATCAGAGCTAGGAATAGAGCCCAGTGC
TTCTGCCTAGCCCAGGCGCCTT-
CATCCTAGACCTCAGTGTCTCTCCAGAGGCTGCAGGAAGGCCTACTGAATTAGCTCGGAGATATTTCCAGGTTCTGCAAC
CTATTTCTTAGTCTGGCTTTTCTGAAAATAAACCTTGAGTGTCCCTTCAGTTTTTCTTT--
CTCTCTTTCCTCCCTCTCAGTTCATTTCTG
Mimus   CAAATATCTGGAGGTATGGAAAAAGATCGG-GAGTATCAGTGTC-T--------
-GATGTGTAGTGAATGTGT--GCAAGA-CAGCTATGTGAGAGTTGGGTTTTCA--------TGACTAGTTGGATTTC-
AGTGAGCTCTCCCTCAAGTCCAAGGTCTATGTGCAAAAGTAGGAGGAGGCACAG--
AAAGGGCTCATGGCATGCAAATGATATGT------GAATGTCCAAGTTTAGATTTACTATTCCAAACA-
TCACATACAGTCTGACCAACCCTTGACAACAATAAACC-AGCCCAT-GCAGCCTGTGCATGCCTGGGAAAGCG-
TATTACA-TAAGGACTGTC---AGTG--------ACTGGACACAAGGGACATT--CAATTTTAGAGTAAGCCCT-
GGAGGATCCATTGGAGACCAAGACCCATAAAAT----TGTTATACAAACACAAGGCATGAATTTG-
CAGTCTTAATGGATGAGGC-
AGACAAAAAGTGGAAAGGGCCATGCTCTACTCAAGGTTATGAAGCATATCAGCGTCAGAGCTATGAATA-
AGCCCAGTGCTTCTGCCAAACCCAGGTTCCTTGCATACTAGACCTCACTGTCTCTCCAGAGGCTGCAGGAAGGCCTACTG
AATTAGCTCGGAGATATTTCCAGGCTCTGCAACCTATTTCTTAGTCTGGATTTTCTGAAAATAAACCTTGAGTGTCCCTT
CAG-TTTT-TCTTTCTCTCTTTCCTTCCTCTCAGTTCATTTCTG
Sturnus_vulgaris   CAAATATCTGGAGGTATGGAAAAGGACAGG-GAGTCTCAGTATC-T--------
-GATGTGTAGTGAATGTGT--GCAAGA-CAGCTATGTGAGAGTTGGGCTTTCATTTACAGATGAGTAGTTGGAYTTC-
AGTGAGCTCTCCCTCAAGTCCAAGGTCTGTGTGTAAAAGTAGGAGGAGGCACAG--
AAAGGGCTCATGGCATGCAAATGGTATGT------GAATATCCAAGTTTAGATTTCCCATTCCAAACA-
CCACATACAGTCTGACCAATCCTTGACAACAGTAAACC-AGCTCAT-GCA----------GCCTGGGAAACCG-
TATTACA-TAAGGACTGTC---AGTG--------ACCGGACATAAGGGACATA--CAATTTTAGAGTAAGCCCT-
GGAGGATCTATTGGAGACCAAGACCCATAAAATTAAGTGTTATACAAACACAGGGCATGAATTTG-
CAGTCTTAATGGATGAGGC-
AGACAAAAAGTAGAAAGGGCCATGGTCTGCTCAAGGTCATGAAGCAGATCAGTGTCAGAGCTATGAATA-
AGCCCAGTGCTTCTGCCTAGCCCAGGCTCCTTGCATACTAGACCTCACTGTCTCTCCAGAGGCTGCAGGAAGGCCTACTG
AATTAGCTCGGAGATATTTCCAGGCTCTGCAACCTATTTCTTAGTCTGGATTTTCTGAAAATAAACCTTGAGTGTCCCTT
CAG-TTTT-TCTTTCTCTCTTTCCTTCCTCTCAGTTCATTTCTG
Creadion_caruncu latus
??
??
??
??
??
??
??
??
??
??
Parus   CAAATATCTGGAGGTATGGAAGAGGGCAAG-GAGTCTCAGTGTC-T--------
-GATGTGTAGTGAATGTGT--GCAAGA-CAGCTGTGTGAGAGTTGGGCTTTCATTTACTGATGACTAGTTGGACTTC-
AGTGAGCTCTCCCTCAAGTCCAATGTCTGTGTGTACAAGCAGGAGGAGACACAG--
AAAAAGCTCACAGTATGCAAATGGTATGT------GAATATCCAAGTTTAGATTTCCCATTCCAAATA-
CCACACACAGTCTGACCAACCCTTGGCAACAATAAACC-AGCCCAT-GCAGCCTATGCATGCCTGAGAAACTG-
CATTACA-TAAGGACTGTC---AGTG--------ACTGGACACAAGGGACATA--CAGTTTTAGAGTAAGCCCT-
GGGGAATCCATTGCAGACCAAGACCCATAAAACT----GTTATACAAACACAGGACATGAATTTG-
CCGTCTTAATGGATGAGGC-
AGACAAAAAGTGGAAAGGGCCATGGTCTGCTCAAGGTTATGAAGCAGATAAGTGTCAGAGCTAGGAATAGAGCCCAGTAC
TTCTGCCTAGCCCAGGCTCCTTGCATAGTAGACCTCACTGTCTCTCCAGAGGCTGCAGGAAGGCCTACTGAATTAGCTCG
GAGATATTTCCAGGCTCTGCAACCTATTTCTTAGTCTGGCTTTTCTGAAAATAAACCTTGAGTGTCCCTTCAGTTTTTCT
TT--CTCTCTTTCCTTCCTCTCAGTTCATTTCTG
Petroica_rosea   CAAATATCTGGAGGTATGGAAAAGGGCAGG-GAGTCTCAATGTC-T--------
-GACATGTAGTGAATGTGT--ACAAGA-CAGCTATGTGAGAGTTGGGCTATTATTTACTGATGACTAGTTGGACTTC-
AGTGAGCTCTCCCTCAAGCCCAAGGTCTGTGCGTACAAGCAGGAGGAGGCACAG--
AAAGGGCTCATGATATGCAAATGGTATGT------GAATATCCAAGTTTAGATTTCCCATTCCAAATA-
CCACACACAGTCTGACCAACCCTTGACAACAATAAACC-AGCCCAT-GCAGCCTATGCATGTCTGGGAAACTG-

TATTACA-TAAAGCCTG-----------------ACTGGACACAAGGGACATA--CAATTTTAGAGTAAGCCCT-
GGAGGATCCATTGGAGACCAAGACCCATAAAACTAAGTGTTATAAAAACACCGGGCATGAATTTG-
CAGTCTTAATGGATGAGGC-
AGACAAAACGTGGGAAGGGCCATGGTCTACCCAAGGTCATGAAGCAGATCAGTGTCAGAGCTAGGAATAGAGCCCAGCAC
TTCTGCCTAGCCCAGGCTCCTTGCATACTAGACCTCACTGTCTCTCCAGAGGCTGCAGGAAGGGCTACCGAATTAGCTCG
GAGATATTTCCAGGCTCTGCAACCTATTTCTTAGTCTGGCTTTTCTGAAAATAAACCCTGAGTGTCCCTTCAG-TTTT-
TCTTTCTCTCTTTCCTTCCTCACAGTTCATTTCTG
Eopsaltria_australis   CAAATATCTGGAGGTATGGAAAAGGGCAGG-GAGTCTCAGCGTC-T--------
-GACATGTAGTGAATGTGT--GCAAGA-CAGCTATGTGAGAGTTGGGCTTTTATTTAATGATCACTAGTTGGACTTC-
AGTGAGCTCTCCCTGGAGTCCAAGGTCTGTGTGTACAAACATGAGGAGGCACAG--
AAAGGGCTCATGGTATGCAAATGGTATGT------GAATATCCAAGTTTAGATTTCCCATTCCAAACA-
GCACACACAGTGTGACCAACCCTTGACAACAATAAACT-GGCCCAT-GCAGCCAATGCATGCCTGGGAAACTG-
TATTACA-TAAGGCCTGTC---AGTG--------ACTGGACACAAGGGACATA--CAATTTTGGAGTAAGCCCT-
GGAGGATCCATTGGAGACCAAGACCCATAAAACTAAGCGTTATACAAACACAGGGCATGAATTTT-
CAGTCTTAATGGATGAGGC-
AGACAAAAAGAGGGAAGGGCCATGATCTACTCAAGGTCATGAAGCAGATCAGCGTCAGAGCTAGGAATAGAGCCCAGCGC
TTCTGCCTAGCCCAGGCTCCCTGCATACTAGACCTCACTGTCTCTCCAGAGGCTGCAGGAAGGGCTACTGAATTAGCTCG
GAGATATTTCCAGGCTCTGCAACCTATTTCTTAGTCTGGTTTTTCTGAAAATAAACCTTGAGTGTCCCTTCAG-TTTT-
TCTTTCTCTCTTTTGTTCCTCACAGTTCATTTCTG
Serinus   ?????????????GTATGGAAAAGGGCAAG-AAGCTTCAGTGTC-T--------
-GATGTGCAGTGAATGTGT--GCAAGA-CAGCTGTGTGAGAGTTGGGCTTTCATTTATTGATGACTAGTTGGACTTC-
AGTGAGCTCTCCCTCAAGTCCA-GG---GTGTGTACAAGCAGTAGGAGGCACAG--AAAGGG-------
TATGCAAATGGTATGT------GAATATCCAAGTTTAGATTTCCCATTCCAAACA-
CCACACACAGTCTGACCAACCCTTGACAGCAATAAACC-AGCCCAT-GCAGCCTATGCATGCCTGGGAAACTG-
TATTACA-TAAGGACTGTC---AGTG--------ACAGGACACAAGGGACATA--CAATTTTAGAGTAAGCCCT-
GGAGGATCTATTGGAGACCAAGACCCATAAAACTAAGTGTTACACAAACACAGGCCATGAATTTG-
CAGTCTTAATGGATGACGC-
CGACAAAAAGTGGAAAGGGCCATGGCCTACTCAAGGTCATGAAGCAGATCAGCATCAGAGCTAGGAATAGAGCCCAGTGC
TTCTGCCTAGCCCAGACTCATTGCATACTAGACCTCACTGTCTCTCCAGAGGCTGCAGGAAGGCCTGCTGAATTAGCTCA
GAGATATTTCCAGGCTCTGCAACCTATTTCTTAGTCTGGCTTTTCTGAAAATAAACCTTGAGTGTCCCTTCAG???????
??????????????????????????????????
Icterus
??
??
??
??
??
??
??
??
??
??
Motacilla   ?????????????GTATGGAAAAGGGCA?G-GAGTCTCACTGTC-T--------
-GATGTGTACTGAATGTGT--GCAAGA-CAGCTGTGTGAGAGTTGGGCTTTCATTTATTGATGACTGGTTGGACTTC-
ATTGAGCTCTCCCTCAAGTCCAGGGTCTGTGTGTACAAGCAGGAGGAGGCACAG--
AAAGGGCTCATGGTATGCAAATGGTATGT------GAATATCCAAGTTTAGATTTCCCATTCCAAACA-
CCCCATGCAGTCTGACCAGCTCTTGACAACAATAAACC-AGCCCAT-GCAGCCTATGCGTGCCTGGGAAACTG-
TATTACA-AAAGGACTGTC---AGTG--------ACTGGACACAAGGGACATA--CAATTTAAGAGTAAGCCCT-
GGAGGACCCATTGGAGACCAAGA-CCATAAAACTACGTGTTATACTAACACAGGGCATGAATTTG-
CAGTCTTAATGGATGAGGC-
TGACAAAAAGTGGAAAGGGTCATGGTCTACTCAAGGTCACGAAGCAGATCAGCGTCAGAGCTAGGAATAGAGCCCAGTGC
TTCTGCCTAGCCCAGACTCC--------------
TCACTGTCTCTCCAGAGGCTGCAGAAAGGCCTACTGAATTAGCTCAGAGATATTTCCAGGCTCTGCAACCTATTTCTTAG
TCTGGCTTTTCTGAAAATAAACCTTGAGTGTCCCTTCAG-TTTT-TCTTTCTCTCTTTCCTTCCTCACAGTTCATTTCTG
Emberiza   CAAATATCTGGAGGTACGGAAAAGGGCAGA-GAGTCTCAGAGTC-T-
CAGTGTCTCATGTGTAGTAWATGTGT--GCAAGA-
CAGCTGTATGAGAGTTGGGCTTTCATTTATTGATGACTAGTTGGACTTC-
AGTGAGCTCTCCCTCAAGTCCAGGGTCTGTGTGTACAAGCAAGAGGAGGCACAG--
AAAGGGCTCATGGTATGCAAATGGTATGT------GAATATCCAAGTTTAGATTTCCCATTCCAAACA-
CCACACGCAGTCTGACCAACCCTTGACAACAATAAACC-AGCCCAT-GCAGCCTATGCATGCCTGGGAAACTG-

TATTACA-TAAGAACTCTC---AGTG--------ACTGGACACAAGGGACATA--AAATTTTTGAGTAAGCCCT-
GGAGGATCCATTGGATMCYWRGAKCCATAAAAGT----GTTATACAAACACAGGGCATGAATTTG-
CAGTCTAAATGGATGAGGC-
CGACAAAAAGTGGAAAGGGCCATGGTCTACTCAAGGTCATGAAGCAGATCAGCGTCAGAGCTAGGAATAGAGCCCAGTGC
TTCTGCCTAGCCCAGACTCCTTGCATACTAGACCTCACTGTCTCTCCAGAGGCTGCAGGGAGGCCTACTGAACTAGCTCA
AAGATATTTCCAGGCTCTGCAACCTATTTCTTAGTCTGGCTTCTCTGAAAATAAACCTTGAGTGTCCCTTCAGTTTTTCT
TT--CTCTCTTTCCTTCCTCACAGTTCATTTCTG
Pomatostomus CAAATATCTGGAGGTACGGAAAAGGTCAGG-GAGCCTCAGTGTC-T--------
-GATGTGTAGTGAATGTGT--GCAAGA-CAGCTATGTGAGAGTTGGGGTTTTATTTACTGATGACGAGTTGGCCTTC-
AGTGAGCTGTCTCTCAAGTCCAAGGTCTGTGTGTACAAGCAGGAGGAGGCACAA--
AAAGGGCTCATGGCATGCAAATGGTATGT------GAATATCCAAGTTTAGATTTCCTATTCCAAACA-
CCACACACAGTTTGACCAACCCTTGACAACAACAAACC-AGCCCAA-GAAGCCTATGCATGCCTTGGAAACTG-
TATTACA-TAAGGACTGTC---AGTG--------ACTGGACACAAGGAACATA--CAATTTTAGAGTAAACCCT-
GGAGGATCCATTGGAGACCAAGACCCATAAAACTAA--GTTATACAAACACAGGGTATGAATTTT-
CAGTCTTAATGGATAAGGC-
AGACAAAAAGTGGGAGGGGCCATGGCCTACTCAAGGTCATGAAGGAGATCAGCGTCAGAGCTAGGAATAGAGCCCAGTGC
TTCTGCCTAGCCCAGGCTCCTTGCACACTAGACCTCACCACCTCTCCAGAGACTGCAGGAAGGGCTACTGAATTAGCTCG
GAGATATTTCCAGGCTCTGCAACCTATTTCTTAGTCTGGCTTTTCTGAAAATAAACCCTGAGTGTCCCTTCAG-TTTT-
TCTTTCTCTCTTTCCTTCCCCACAGTTCATTTCTG
Rhipidura CAAATATCTGGAGGTATGGAAAAGGGCAGG-GAGTCTCAGTGTC-T--------
-GATGTGCAGTGAATGTGT--GCAAGA-CAGCAATGCGAGAGTTGTGCTTTTATTTACTGAAGACTAGTTGGACTTT-
AGTGAGCTCTCCCTCAAGTCCAGGGTTTGTGTGCACAAGCAGGCGGAGGCACAG--
AAAGGGCTCATGTTATGCAAATGGTATGT------GAATATCCAAGTTTAGATTTCCCATTCCAAACA-
TCACACACAGTCTGACCAGCCCTTGACAACAATAAACC-AGCCCAT-GCAGTCTATGCATGCCTGGGAAACTG-
TATTACA-TAAGGCCTGTC---AGCG--------ACTGGACACAAGGGACATA--CAATTTTAGAGTAAGCCCT-
GGAGGATCCATTGGAGACCAAGACCCATAAAACTAAGTGTTATACAAACACAGGGCATGAATTTG-
CAGACTTAATGGGTGAGGC-
AGACAAAAAGTGGGAAGGGCCATGGTCTACTCAAGGTCATGAAGCAGATCAGCGTCAGAGCTAGGCATAGAGCCCAGTGC
TTCTGCCTAGCCCAGGCTCCTTGCATGCTAGACCTCACTGTCTCTCCAGAGGCTGCAGGAAGGGCTACTGAATTAGCTCG
GAGATATTTCCAGGCTCTGCAACCTATTTCTTAGTCTGGCTTTTCTGAAAATAAACCTTGAGTGTCCCTTCAG-TTAT-
TCTTTCTCTCTTTCTTTCCTCACAGTTCATTTCTG
Pica_pica CAAATATCTGGAGGTATGGAAAAGGGCAGG-GAATTTCAGTGTC-T--------
-GATGTGTGGTGAATGTGT--GCAAGA-CAGCCATGTGAGAGTTGTGCTTTTATTTACTGATGACTAGTTGGACTTC-
AGTGAGCTCTCCCTCACGTCCAAGGTCTGTGTGTACAAGCAGCAGGAGGCACAG--
AAAGGGTTCATGGTATGCAAATAGTACGT------GAACATCCAAGTTTAGA-TTCCCATTCCAAAC-----
CACAGAGTCTGACCAGCCCTTGACAACAATAAACC-AGCCCAT-GCAGCCTATGCATGCCTGGGAAACTG-TATTACA-
TAAGGCCTGTC---AGTG--------ACTGGACGCCAGGGACATA--CAATTTTAGAGTAAGCCCT-
GGAGGATCCATTGGAGACCAAGACCCATAAAACTAAGTGTTAYACAAACACCGGGCATGAATTTG-
CAGACTTAATGGATGAGGC-
AGACAAAAAGTGGGAGGGGCCATGGTCTACTCAAGGTCATGAAGCAGATCAGCATCAGAGCTAGGCAGAGAGCCCAATGC
TTCTGCCTAGCCCAGGCTCCTTGCATACTAGACCTCACTGTCTCTCCAGAGGCTGCAGGGAGGGCTACTGAATTAGCTCG
GAGATATTTCCAGGCTCTCCAACCTATTTCTTAGTCTGGCTTTTCTGAAAATAAACCCTGAGTGTCCTTTCAG-TTTT-
TCTTTCTCTCTTTCCTTCCTCACAGTTCATTTCTG
Manucodia CAAATATCTGGAGGTATGGAAAAGGGCAGG-GAGTCTCAGTGTC-T--------
-GATGTGTAGTGAATGTGT--GCAAGA-CAGCTACGTGAGAGTTGTGCTTTTATTTACTGATGACTAGTTGGACTTC-
AATGAGCTCTCCCTCAAGTCCAAGGTCTGTGTGTACAAGCAGATGGAGGCACAA--
AAAGGGGTCATGGTATGCAAATGGTATGT------GAATATCCAAGTTTAGATTTCCCATTCCAGACA-
CCACACACAGTCTGACCAGCCCTTGACAACATTAAACC-AGCCCAT-GCAGCCTATGCATGCCTGGGAAACTG-
TATTACA-TAAGGCCTGTC---AGTG--------ACTGGACACAAGGGACATA--CAATTTTAGAGTAAGCCCT-
GGAGGATCCATTGGAGACCAAGACCCATAAAACTAAGCGTTATACAAACACAGGGCATGAATTTG-
CAGACTTAATGGATGAGGC-
AGACAAAAAGTGGGAGGGGCCATGGTCTACTCAAGGTCATGAAGCAGATCAGTGTCAGAGCTAAGTACAGAGCCCAGTGC
TTCTGCCCAGCCCAGGCTCCTTGCATACTAGACCTCACTGTCTCTCCAGAGGTTGCAGGAAGGGCTACTGAATTAGCTCA
GAGATATTTCCAGGCTCTGCAACCTATTTCTTAGTCTGGCTTTTCTGAAAATAAACCTTGAGTGTCCCTTCAGTTTTTCT
TTCTCTCT--TTCCTTCCTCACAGTTCATTTCTG
Corvus_corone CAAATATCTGGAGGTATGGAAAAGGGCAGG-GAATCTCAGTGTC-T--------
-GATGTGTGGTGAATGTGT--GCAAGA-CAGCCATGTGAGAGCTGTGCTTTTATTTACTGATGACTAGTTGGACTTC-
MGTGAGCTCTCCCTCACGTCCAAGGTCTGTGTGTACAAGCAGCAGGAGGCACAG--
AAAGGGCTCATGGTATGCAAATGGTACGT------GAATATCCAAGTTTAGA-TYCCCATTCCAAACA-
TCACACACAGTCTGACCAGCCCTTGACAACAATAAACC-AGACCAT-GCAGCCTATGCATGCCTGGGAAACTG-

TATTACA-TAAGGCCTGTC---AGTG--------ACTGGACGCAAGGGACATA--CAATTTTAGAGTAAGCCCT-
GGAGGATCCATTGGAGACCAAGACCCATAAAACTAAGTGTTATACAAACACTGGGCATGAATTTG-
CAGACTTAATGGATGAGGC-
AGACAAAAAGTGGGAGGGGCCATGGTCTACTCAAGGTCATGAAGCAGATCAGCATCAGAGCTAGGCAGAGAGCCCAGTGC
TTCTGCCTAGCCCAGGCTCCTTGCATACTAGACCTCACTGTCTCTCCAGAGGCTGCAGGGAGGGCTACTGAATTAGCTCG
GAGATATTTCCAGGCTCTCCAACCTATTTCTTAGTCTGGCTTTTCTGAAAATAAACCTTGAGTGTCCCTTCAGTTTTTCT
TT--CTCTCTTTCCTTCCTCACAGTTCATTTCTG
Vireo   CAAATATCTGGAGGTATGGAGAAGGGCAGT-GAGTCTCAGTGTC-T--------
-GATGTGTAGTGAATGTGT--GCAAGA-CAGCTATGTGAGAGTTGTGCTTTTATTTACTGATGA--------ACTTC-
AGTGAGCTCTCCCTTGAGTCCAAGGTCTGTGTGTACAAGCAGGAGGAGGCACAG--
AAAGGGCTCATGGTATGCAAATGGTATGT------GAAAATCCAAGTTTAGATTTCCCGTTCCAAACA-
CCGCACACAGTCTGACCAGCCCTTGACAGCAATAAACC-AGCCCAT-GCAGCCTATGCATGCCTGGGAAACTG-
TATTACA-TAAGGCCTGTC---AGTG--------ACTGGACACAAGGGACGTA--CAATTTTAGAGTAAGCCCT-
GGAGGATCCACTGGAGACCAAGACCCATAAAATTAAGTGTTATACAAACACAGGGCATGAATTTG-
CAGACTTAATGGATGAGGC-
AGACAAAAAGTGGGAGGGGCCATGGTCTACTCAAGGTCACGAAGCAGATCAGCGTCAGAGCTAGGAATAGAGCCCAGTGC
TTCTGCCTAGCCCAGGCTCCTTGCATACTAGACCTCACTGTCTCTCCAGAGGCTGCAAGAAGGGCTACTGAATTAGCTTG
GAGATATTTCCAGGCTCTGCAACCTATTTCTTAGTCTGGCTTTTCTGAAAATAAACCTCGAGTATCCCTTCAG-TTTT-
TCTTTCTCTCTTTCCTTCCTCACAGTTCATTTCTG
Camptostoma_obsoletum
??
??
??
??
??
??
??
??
??
??
Promerops_cafer   TAAATATCTGGAGGTATGGAAAAGGGCAGG-GAGTCTCATTGTC-T--------
-GATGTGTAGTGAATGTGT--GCAAGA-CAGCTGTGTGAGAGTTGGTCTTTCATTTATTGATGACTAGTTGGACTTC-
AGTGAGCTCTCCCTCAAGTCCAAGGTCTGTGTGTACAAGCAGGAGGAGGCACAG--
AAARGGTTCATGGTATGCAAATGGTATGT------GAATATCCAAGTTTAGATTTCCYATTCCAAGCA-
CCACACACAGTCTGACCAACCCTTGACAACAATAAACC-AACCCAT-GCAGCCTATGCATGCCTGGGAAACTG-
TATTACA-TAAGGACTGTC---AGTG--------ACTGGGCACAAGGGACATA--CAATTTTAGAGTAAGCCCT-
GGAGGATTCCTTGGAGACCAAGACCCATAAAACTAAGTGTTATACAAACACAGGGCATGAATTTG-
CAGTGTTAATGGATGAGGC-
AGACAAAAAGTGGAAAGGGCCATGGTCTACTCAAGGTCATGAAGCAGATCAGCGTCAGAGCTAGGAATAGAGMCCAGTGC
TTCTGCCTAGCCCAGGCTCCTTGCACACTAGACCTCACTGTCTCTCCAGAGGCTGCAGGAAGGCCTACTGAATTAGCTTG
GAGATATTTCCAGGCTCTGCAACCTATTTCTTAGTCTGGCTTTTCTGAAAATAAACCTTGAGTGTCCCTTCAG--TTT-
TCTTTCTCTCTTTCCTTCCTCACAGTTCATTTCTG
Oriolus   CAAATATCTGGAGGTATGGAAAAGGGCA---GAGTCTCAGTGTC-T--------
-GATGTGTAGTGAATGTGT--GCAAGA-CAGCTATGTGAGAGCTGTGCTTTTATTTACTGATGACTAGTTGGACTTC-
AGTGAGCTCTCTCRCAAGTCCAAGGTCTGTGTGTACAAGCAGGAGGAGGCACAG--
AAAGGCCTCATGGTATGCAAATGGTATGT------GAATATCCAAGTTTAGATTTCCCATTCCAAACA-
CCACACACAGTCTGACCAGCTCTTGACAGCAATAAACC-AGCCCAT-GCAGCCTTTGCATGCCTGGGAAACTG-
TATTACA-TAAGCCCTGTC---AGTG--------ACTGGACACAAGGGACATA--CAATTTTAGAGTAAGCCCT-
GGAGGATCCATTGGAGACCAAGACCCATAAAATGAAGTGTTATACAAACACAGGGCATGAATTTG-
CAGACTTAATGGATGAGGC-
AGACAAAAAGTGGGAGGGGCCATGGTCTACTCAAGGTCATGAAGCAGATCAGCGTCAGAGCTAGGAATAGAGCCCAGTGC
TTCTGCCTAGCCCAGGCTCCTTGCATGCTAGACCTCACTGTCTCTCCAGAGGCTGCAGGAAGGGCTACTGAATTAGCTCA
GAGATATTTCCAGGCTCTGCAACCTATTTCCTAGTCTGGCTTTTCTGAAAATAAACGTTGAGTGTCCCTTCAGTTTTTCT
TT--CTCTCTTTCCTTCCTCACAGTTCATCTCTG
Dendroica   ?????????GGAGGTATGGAAAAGG-AAGA-GATTCTCAGTGTC-T--------
-GATGTGTAGTGAATGTGT--GTAAGA-CAGCTGTGTGAGAGTTGGGCTTTCATTTATTGATGACTTGTTGTACTTC-
AGTGAGCTCTCCCTCAASTCCAGGGTCTGTGTGTACAAGCAGGAGGAGGCACAG--
AAAGGGCTCATGGTATGCAAATGGTATGT------GAATATCCAAGTTTAGATTTCCCACTCCAAACA-
CCACACGCAGTCTGACCAACCCTTGACAACAATAAACC-AGCCCAT-GCAGCCTATGCATGCCTGGGAAACCG-
TATTACA-TAAGGACTGTY---GGTG--------ACTGGACACAAGGGACATA--CAATTTTAGAGTAAGCCCT-

GGAGGATCCATTGGAGACCAAGACCCATAAAAGTAAGTGTTATACAAACACAGGGCATGAATTTG-
CAGTCTTGATGGATGAGGC-
TGACAAAAGGTGGAAAGGGTCATGGTCTACTCAAGGTCATGAAGCAGATCAGCGTCAGAGCTAGGAATAGAGCCCAGTGC
TTCTGCCTAGCTCAGACTCCTTGCCTACTAGACCTCACTGTCTCTCCAGAGGCTGCAGGAAGGCCTACTGAATTAGCTCA
GAGATATTTCCAGGCTCTGCAACCTATTTCTTAGTCTGGTTTTTCTGAAAATAAACCTTGAGTGTCCCTTCAGTTTTTCT
TT--CTCTCTTTCCTTCCTCATAGTTCATTTCTG
Nectarinia CAAATATCTGGAGGTATGGAAAAGGGCAGG-GAGTCTTGGGGTC-TA-------
-GATGTGTAGTGAATGTGT--GCAAGA-CAGCTATGTGAGAGTTGGACTTCCATTTACTGATGACTAGTTGGACTTC-
AGTGAGCTCTCCTTGAAGTCCAAGGTCTGTGTGTATAAGCAGGAGGAGGCACAG--
AAAGGGCTCACGGTATTCAAATGGTATGT------GAATATCCAAGTTTAGATTTCCCATTCCAAACA-
CCACACACAGTCTGACCAACCCTTGACAACAATAAACC-AGCACAT-GCAGCCTATGCGTGCCTGGGAAACTA-
CATTACA-TAAGGACTGTC---AGTG--------ACTGGACACAAGGGACATA--CAATTTTAGAGTAAGCCCT-
GGAGGATCCATTGGAGACCAAGACCCATAAAACTAAGTGTTATAGAAACACAGGACATGAATTTG-
CAGTCTTAATGGATGAGGC-
AGACAAAAAGTGGAAAGGGCCATGGTMTCCTCAAGGTCATGAAGCAGATCAGCGTCAGAGCTAGGAATAGAGCCCAGTGC
TTCTGCCTAGCCCAGACTCCTTGCATACTAGACCTCACTGTCTCTCCAGAGGCTGCAGGAAGGCCTACTGAATTAGCTCG
GAGATATTTCCAGGCTCTGCAACCTATTTCTTAGTCTGGCTTTTCTGAAAATAAACCCTGAGTGTCCCTTCAGTTTTTCT
TT--CTCTCTTTCCTTCCTCACAGTTCATTTCTG
Amytornis_striatus CAAGTATCTGGAGGTATGGAAAAGGGCAGG-GAGTCTCAGTGTC-T--------
-GATGTATAGTGAATGTGT--GCAAGA-CAGCTGTGTGAGAGTTGTGCTTTTATTTACTGATGGCTAATTGGACTTC-
AGTGAGCTCTCCCTCCAGTCCGAGCTCTGTGTGTGCAAGCAGGAGGAGGCACAA--AAAGACTTCATGGTATGC------
------------AAATATCCAAGTTTAGATTTCCCATTCCAAACA-
CCACATACAATGTGACCAGCCCTTGACAACAATAAACC-AGCCCAT-GCCTCCTRTGCATGCCTGRGAAACTG-
TATTACA-TAAGGGCTGTC---AGTG---------ATGGACACAAGGGACATA--YAATTTTAGAGTAAGCCCT-
GGAGGATCCATTGGAGACCAAGACCCATAAAACTAAGTATTATACAAACACATGGCATGAATTTG-
CAGTCTTAATGGATGAGGC-
AGACAAAAAGTGGGAAGGGCCATGGTCTACTCAAGGTCRTGAAGCAKATCAATGYYAGAGCTAGGAATAGAGCCCAGTGC
TTCTACCTAGCCCAGGCTCCTTGCATATTAGACCTCACTGTCTCTCYAGAGACTGTAGGAAGGGCTACTGAATTAGCTCR
GAGATATTTCCAGGCTCTGCAACCTATTTCTTAGTCTGGCTTTTCTGAAAATAAACCCTGAGTGTCCCTTCAG-TTTT-
TCTTTCTCTCTTTCCTTCCTCACAGTTCATTT???
Pitta CAAATATCTGGAGGTATGGAAAAGGGCAGG-GCATCTTGGTGCC-T--------
-GATGTATAGTGGACGTGT--GCAAGA-CAACTATATGAGAGTTGTACTTTTATTTACTGATGTCTAATTGAATTTC-
AGTGAGCTCTCCTTCCAGTTCAAGGTCTCTGT--ACAGACAGCAGGAAGCGCAG--AAAGGGCTCATGGTATGT------
------------GAATATCCAAGTTTAGATTTCCCACTCCAAACA-
CCACGTGCAGTCTGAGCAAGTCTTGACAACAATAAACC-AACCCAT-GCTGCCTCTGCATGCCTGGGAAACTA-
TATTGCA-TAAGACCTGTT---TGTG--------GCTGGACACAAAAGGTATA--CAATTTTAGAGTAAGCCCT-
GGAGGAGACATTGGAGACCAAGACCCATAAAACTAAGTGTTATACAAACACAGGGCATGAATTTT-
TGGTCTTAATGGATAAGGT---------
GGTGGTAAGGGCCATGGTCTACTCAAGGTCATGAAGCAGATCAGCATCAAAGCTAGGAATAGAGCCCAGTCCTTCTGCCT
AGCCCAGGCTCCTTGCATACTAGACCTCACTGTCTCTCGAGGGACTATGGGGAGGGCTACTGAAATAGCTCAGAGATATT
TCCAGGCTCTGCAACCTATTTCTTAGTCTGGCTTTTCTGAAAATTAACCTTGAGTGTCCCTGTAG-TTTT-
TTTCTCTCTCTTTCCTTCCTCACAGTTCATTTCTG
Toxorhamphus ?????????????GTATGGAAAAGGGCAGG-GAGTCTCAGTGTC-T--------
-GATGTGTAGTTAATGTGT--GCAAGA-CAGCTCTGTGAGAGTTGGGCTTTTATTTACTGATGACTAATTGGACTTC-
AGTGAGCTCTCCCTCAAGTCCAAGGTCTGTGTGTACAAGCAGGAGGAGGCACAG--
AAAGGGCTCATGGTATGCAAATGGTATGT------GAATACCCAAGTTTAGACTTCCCATTCCAAACA-
CCACACACAGTCTGACCAGCCCTTGACAACAATAAACC-AGCCCAT-GCAGCCTATGCATGCCTGGGAAACTG-
TATTACA-TAAGGACTGTC---AATG--------ACTGGGCACAAGGGACATA--CAATTTTAGAGTAAGCCCT-
GGAGGATCCAGTGGAGACCAAGACCCATAA------GCGTTATAGAAACACAGGGCATGAATTTG-
CAGTCTTAGTGGATGAGGC-
AGACAAAAAGTGGGAGGGGCCATGGTCTACTCAAGGTCATGAAGCAGATCAGCGTCAGAGCTAGGAATAGAGCCCAGTGC
TTCTGCCTAGCCCAGGCTCCTTGCATACTAGACCTCACTGTCTCTCCAGAGGCTGCAGGAAGGGCTACTGAATTAGCTCG
GAGATATTTCCAGGCTCTGCAACCTATTTCTTAGTCTGGCTTTTCTGAAAATAAACCCTGAGTGTCCCTTCAG-TTTT-
TCTTTCTCTCTTTCCTTCCTCACAGTTCATTTCTG
Orthonyx_temminckii CAAATATCTGGAGGTATGGAAAAGGGCAGG-GAGTCTCAGTGCC-T--------
-GATGTGTAGTGAATGTGT--GCAAGA-CAGCTATGTGAGTGTTGCGTTTTTATTTACTGATGGCTAGTTGGACTTC-
AGTGAACTCTTCATCAAGTCCAAGGTCTGTGTGTACAAGCAGGAGGAGGCACAA--
AAAGGGCTCATGGTATGCAAATGGTATGT------GAATATCCAAGTTTAGATTTCCCACTCCAAACA-
CCACACACAGTCTGACCAACCTTTGACAACAATAAACC-AGCCCAT-GCAGCCTATGCACGCCTGGGAAACTG-
TGTTACA-TAAGGACTGTC---GGTG--------ACTGGACACAAGGGACATA--CAATTTTAGAGTAAGCCCT-

GGGGGATCCATGGGAGACCAAGACCCGTAAAACTAAGTGTTATACAAACACAGGGCATGAATTTG-
CAGTCTTAATGGATGAGGC-
AGACAAAAAGTGGGAGGGGCCATGGTCTACTCAAGGTCATGAAGCAGATCAGCGTCAGAGCTAGGAATAGAGCCCAGTGC
TTCTGCCTAGCCCAGGCTCCTTGCATACTAGACCTCACTGTCTCTCCAGAGACTGCAGGAAGGGCTACTGAATTAGCTCG
GAGATATTTCCAGGCTCTGCAACCTATTTCTTAGTCTGGCTTTTCTGAAAATAAATCCTGAGTGTCCCTTCAGTTTTTCT
TTCTCTCT--TTCCTTCCTCGCAGTTCATTTCTG
Sericulus_chrysocephalus
??
??
??
??
??
??
??
??
??
??
Cyclarhis_gujanensis   ?????????????GTATGGAGAAGGGCAGT-GAGTCTCGGTGTC-T--------
-GATGTGTAGTGAATGTGT--GCAAGA-CAGCTATGTGAGAGTTGTGCTTTTATTTACTGATGACTAGTTGGACTTC-
AGTGAGCTCTCCCTCGAGTCCAAGGTCTGTGTGTACAAGCAGGAGGAGGCACAG--
AAAGGGCTCATGGTATGCAAATGGTGTGT------GAAAATCCAAGTTTAGATTTCCCATTCCAAACA-
CCGCACACAGTCTGACCAGCCCTTGACAGCAATAAACC-AGCCCAT-GCAGCCTATGCATGCCTGGGAAACTG-
TATTACA-TAAGGCCTGTC---AGTG--------ACTGGACACAAGGGATGTA--CAATTTTAGAGTAAGCCCT-
GGAGGATCCATTGGAGACCAAGACCCATAAAATTAAGTGTTATACAAACACAGGGCATGAATTTG-
CAGACTTAATGGATGAGGC-
AGACAAAAAGTGGGAGGGGCCACGGTCTACTCAAGGTCATGAAGCAGATAAGCGTCAGAGCTAGGAATAGAGCCCAGTGC
TTCTGCCTAGCCCAGGCTCCTTGCATACTAGACCTCACTGTCTCTCCAGAGGCTGCAGGAAGGGCTACTGAATTAGCTTG
GAGATATTTCCAGGCTCTGCAACCTATTTCTTAGTCTGGCTTTTCTGAAAATAAACCTCGAGTATCCCTTCAGTTTTTCT
TTCTCTCT--TTCCTTCCTCACA???????????

[RAG-1]
Polyborus_plancus   TAAAGTGAGAACATTTGAAAAAACACCTT-------------------------
----
CTGATGACAGCCAGCACATAAACAAAGCCCAGGCAGAAGAGGTCACTTCTTCAAACAAAGAAACCATACTGCGTAAAGAT
GAAGCAGTGGCGAGAGGAGAAAAGATGGAGTTAATAGGCAATAGGCAGGCACTTGAGAAAGATGCCAATGACATGAAAAC
ACAAGACAATAAAGCTCATCAGAACAATCTGAAGCAACTTTGCCGCATCTGTGGAGTTTCATTTAAAACTGATTGTTACA
GGAGAAGTCATCCAGTGCATGGGCCGGTGGATGATGAAACTCTGTGGCTTCTGAGAAAGAAAGAGAAAAAAGCAACCTCT
TGGCCAGATCTTATCGCTAAGGTTTTCAAAATTGATGTGCGAGGAGATGTTGATACTATCCATCCCACTCGATTTTGTCA
CAATTGCTGGAGTATTATCCATAGAAAATTCAGTAATACTCCATGTGAAGTATATTTTCCTAGGAACAGCACAATGGATT
GGCAACCCCACTCCCCAAACTGTGATGTGTGCCACACTACCAAGCGGGGAGTCAAGAGAAAAAGCCAGCCACCCAGTGCA
CAACATGGCAAACGTATGAAGACCATTGTGGAATGTGCTCGAATAAACAGAGCTGTAAAGAAC------------
CAAGCACAGATAAACAACAAAAATTTAATGAAAGAGATTGTCAATTGCAAGAATACACATCTCAGCACCAAGCTGCTTGC
AGTTGATTACCCAGTAGATTTCATTAAATCCATTTCTTGCCAGATCTGTGAGCATATTTTGGCAGATCCAGTGGAAACAA
CATGTAGACACTTGTTTTGCAGAACTTGCATCCTTAAATGTATCAAGGTTATGGCAGCTACTGCCCATCCTGCTGGTAT
CCTTGCTTCCCTACTGATCTGGTAACCCCAGTGAAGTCCTTCCTGAACATCCTTGATAGCCTGGGTATAAGATGCCCTGT
AAAGGAATGTGATGAAGAGATCTTGCATGGAAAATATGGCCAACACCTCTCCAGCCACAAGGAGATGAAAGACAGAGGGC
TCTACAGCCACATAAATAAAGGTGGCCGACCAAGGCAGCATCTCCTGTCTTTAACCAGGAGAGCTCAGAAACATCGTCTG
AGAGAACTGAAACGTCAAGTCAAGGCTTTTGCTGAGAAAGAAGAGGGCGGCGATATAAAGGCTGTATGCATGACATTGTT
CCTGCTAGCTTTAAGAGCAAAAAATGAACACAGACAAGCAGATGAATTGGAGGCTATAATGCAAGGGAGGGGATCTGGAC
TTCACCCTGCTGTCTGCCTGGCAATCCGAGTCAACACGTTTCTCAGCTGTAGCCAATATCATAAAATGTATAGAACAGTA
AAAGCTGTCACTGGGAGGCAGATCTTCCAGCCTTTGCATGCTCTTCGCACTGCTGAGAAAGCCCTTCTACCAGGTTATCA
TCCATTTGAGTGGAAACCTCCCTTGAAAAATGTATCCACTAACACAGAAGTGGGAATTATAGATGGACTATCAGGATTGC
CACACTCAATTGATGACTACCCAGTAGACACAATTGCAAAGAGATTTCGATATGATGCAGCCTTGGTTTGTGCCTTAAAG
GACATGGAGGAGGAGATCTTGGAAGGCATGAAAGCAAAAAACGTGGATGACTATTTGAATGGTCCCTTCACTGTGGTAGT
AAAAGAGTCCTGTGATGGAATGGGAGATGTCAGCGAGAAGCACGGAGGTGGGCCTGCTGTCCCAGAGAAGGCCGTTCGCT
TTTCTTTCACTGTCATGAACATCGCTGTAGCACAAGGGAATGAAAGCAAGAGGATCTTTGAAGAAGTAAAACCCAATTCG
GAGTTGTGTTGCAAGCCCTTGTGCCTTATGCTGGCTGATGAATCTGATCATGAAACTCTGACGGCAATCCTGAGTCCCCT
CATAGCAGAAAGAGAGGCTATGAAAAACAGTGAACTGCTACTTGAAATGGGAGGCATCCTGAGAACATTCAAATTCATCT
TTAGGGGTACAGGATATGATGAGAAACTCGTGCGGGAAGTGGAAGGACTGGAGGCCTCAGGTTCCACTTACATTTGTACC

CTGTGCGATGCAACCCGCCTGGAGGCTTCCCAGAATTTGGTCTTCCACTCCATAACCAGGAGCCATGCTGAAAATCTGGA
GCGATATGAAATATGGAGGTCCAACCCATATCACGAATCTGTTGATGAACTCCGTGACAGAGTGAAGGGTGTTTCAGCCA
AACCTTTCATTGAGACCGTTCCCTCCATAGATGCGTTGCACTGTGACATTGGCAATGCAACAGAATTCTACAGGATATTC
CAGATGGAGATCGGTGAAGTTTACAAGAATCCTGATGTGTCTAAAGAGGAGAGGAAGAGGTGGCAGTTGACGCTTGACAA
ACACCTCAGGAAGAAGATGAACTTGAAGCCTATGATGAGGATGAGTGGAAATTTTGCTAGAAAGCTCATGTCCAAAGAGA
CAGTAGAGGCAGTATGTGAATTAATAAAGTGTGAGGAAAGGCATGAAGCCCTAAAAGAACTAATGGACCTTTATCTGAAG
ATGAAGCCAGTGTGGCGATCCTCATGCCCTGCTAAGGAGTGTCCAGAATTGCTGTGCCAGTATAGCTACAATTCACAGCG
TTTTGCTGAGCTTCTGTCTACAAAGTTCAAGTACAGATATGAGGGCAAGATTACAAATTATTTCCACAAAACCCTTGCTC
ATGTTCCTGAAATCATTGAAAGAGATGGGTCCATTGGGGCCTGGGCAAGTGAAGGAAATGAGTCTGGAAACAAACTGTTT
AGGAGGTTCCGAAAAATGAATGCCA???????????????????????????????????
Falco   TAAAGTGAGAACATTTGAAAAAACACCTT-------------------------
----
CTGATGACAGCCAGCACATAAACAAAGCCCAGGCAGAAGAGGTCACTTCTACAAACAAAGAAATCATACTGCATAAAGAT
GAAGCAGTGCTGAGAGGAGAAAAGATGGAGTTAATAGGCAGTAGGCAGGCACTTGAGAAAGATGCCAACGACATGRAAAC
AGAAGACAATAAAGCTCATCAGAACAATCTGAAGCAACTTTGCCGCATCTGTGGAGTTTCATTTAAAACTGATTGTTACA
GGAGGAGTCATCCAGTGCACGGACCAGTGGATGATGAAACTCTGTGGCTTCTGAGAAAGAAAGAGAAAAAAGCAACCTCT
TGGCCAGATCTTATCGCTAAGGTTTTCAAAATTGATGTGCGAGGAGATGTTGATACTATCCATCCCACTCGATTTTGTCA
CAATTGCTGGAGTATTATCCATAGAAAATTCAGTAATACTCTATGTGAAGTATACTTTCCAAGGAACAGCACAATGGATT
GGCAACCCCACTCCCCAAACTGTGATGTGTGCCACACTACCAAGCGTGGAGTCAAGAGAAAAAGTCAGCCATCCAGCGCA
CAGCATGGCAAACGTATGAAGACCGTTGCAGAATGTGCTCGAATAAACAGAGCTGTAAAGAAC------------
CAAGCACAGATAAACAACAAAAGTTTAATGAAAGAGATTGTCAATTGCAAGACTATACATCTCAGCACCAAGCTGCTTGC
AGTTGATTACCCAGTAGATTTCATTAAATCCATTTCTTGCCAGGTCTGTGAGCATATTCTGGCAGATCCAGTGGAAACAA
CATGTAGACACTTGTTTTGCAGAACTTGCATCCTTAAATGTATCAAGGCTATGGGCAGCTATTGCCCATCCTGCTGGTAT
CCTTGCTTCCCTACTGATCTGGTAACCCCAGTGAAATCCTTCCTGAACATCCTTGATAACCTGAGTATAAGATGCCCTGT
AAAGGAATGTGATGAAGAGATCTTGCATGGAAAATATAGCCAACACCTCTCCAGCCACARGGAGATGAAAGACAGAGGGC
TCTACAGCCATATAAATAAAGGTGGCCGCCCAAGGCAGCATCTCCTGTCTCTGACCAGGAGAGCTCAGAAACATCGTCTG
AGAGAACTGAAACGTCAAGTCAAGGCTTTTGCTGAGAAAGAAGAAGGCGGTGATATAAAGGCTGTATGCATGACATTGTT
CCTGCTAGCTTTAAGAGCAAAAAATGAACACAGACAAGCAGACGAATTGGAGGCTATAATGCAAGGGAGGGGATCTGGAC
TTCACCCTGCTGTCTGCCTGGCAATCCGAGTCAACACGTTTCTCAGCTGTAGCCAATATCATAAAATGTATAGAACAGTA
AAAGCTGTCACTGGGAGACAGATCTTCCAGCCTTTGCACGCTCTTCGCACTGCTGAGAAAGCCCTTCTACCAGGTTATCA
TCCATTTGAGTGGAAACCTCCCTTGAAAAATGTATCCACTAACACAGAAGTGGGAATTATAGATGGACTATCAGGATTGC
CACACTCAATTGATGACTACCCAGTAGACACAATTGCAAAGAGATTTCGATATGATGCAGCCCTGGTTTGTGCCTTAAAG
GACATGGAGGAGGAGATTTTGGAAGGCATGAAAGCAAAAAATGTGGATGACTATTTGAATGGTCCCTTCACTGTGGTAGT
AAAAGAGTCCTGTGATGGAATGGGAGATGTCAGCGAGAAGCACGGAGGTGGGCCTGCTGTCCCAGAGAAGGCCGTTCGCT
TTTCTTTCACAGTCATGAACATTGCTATAGCACAAGGGAATGAAAGTAAGAGGATCTTTGAAGAAGTAAAACCCAATTCA
GAGTTGTGTTGCAAGCCCTTGTGCCTTATGCTGGCTGATGAATCTGATCATGAAACTCTGACGGCAATCCTGAGTCCCCT
CATAGCAGAAAGAGAGGCTATGAAAACCAGTGAACTGCTACTTGAAATGGGAGGAATCCTGAGAACATTCAAATTCATCT
TTAGGGGTACAGGATATGATGAGAAACTCGTGCGGGAAGTGGAAGGGCTGGAGGCCTCAGGTTCCACGTACATTTGTACC
CTGTGCGATGCAACCCGCCTGGAGGCGTCCCAGAATTTGGTCTTCCACTCCATAACCAGGAGCCATGCTGAAAATCTGGA
GCGATATGAAATATGGAGGTCCAACCCATATCATGAATCTGTTGATGAGCTCCGTGACAGAGTGAAGGGTGTTTCAGCCA
AACCTTTCATTGAGACCGTTCCCTCCATAGATGCGTTGCACTGTGACATTGGCAATGCAACAGAATTCTACAGGATATTC
CAGATGGAGATTGGTGAAGTTTACAAGAATCCTGATGTGTCTAAAGAGGAGAGGAAGAGGTGGCAGTTGACGCTTGACAA
ACACCTCAGGAAGAAGATGAACTTGAAGCCTATGATGAGGATGAGTGGAAATTTTGCTAGAAAGCTCATGTCCAAAGAGA
CAGTAGAGGCAGTATGTGAATTAATAAAGTGTGAGGAAAGGCATGAAGCCCTAAAAGAGCTAATGGACCTTTATCTGAAG
ATGAAGCCAGTGTGGCGATCCTCATGCCCTGCTAAGGAGTGTCCAGAATTGCTGTGCCAGTATAGCTACAATTCACAGCG
TTTTGCTGAGCTTCTGTCTACAAAGTTCAAGTACAGATATGAGGGCAAGATTACAAATTATTTCCACAAAACCCTTGCTC
ATGTTCCTGAAATCATTGAAAGAGATGGGTCCATTGGGGCCTGGGCAAGTGAAGGAAATGAGTCTGGAAACAAACTGTTT
AGGAGGTTCCGAAAAATGAATGCCA???????????????????????????????????
Tyrannus   TAAAGTGCGATCATTTGAAAAAACAA----------------------------
----
CTGATGACAGGCAGCACATAAGCAAAGATCAGGCAGAAGGGATTGCTTCTTCAAACAAAGAAATCATACTGCATAAAGAT
AAAGCAGTGTCAAGAGAAGAAAAGATGGAGTTAATGGGCAATAGGCAGGCACTTGAAGAAGAGGACCATGACATGAAAAC
ACGAGACAATAGAGCTCATCAGAACAATCTGAAGCAACTTTGTCGCATCTGTGGAGTTTCATTTAAAACTGATTGCTACA
AGAGAACTCATCCAGTGCATGGGCCTGTGGATGATGAAACTCTGTGTCTTCTGAGAAAGAAAGAAAAAAAAAGCAACCTCT
TGGCCAGATCTTATAGCTAAGGTGTTCAAGATTGATGTGCGAGGGGATGTTGATACTGTCCATCCCACTCGGTTTTGTCA
CAACTGTTGGAGTATTATCCATAGAAAATACAGTAATACTCTRTGTGAGGTATATTTTCCTAGGAACAGCACCATGGAGT
GGCAGCCTCACTCCCCAAACTGTGAYGTATGCCATACTACCAGTCGAGGGGTCAAGAGAAAAAGCCAGACACCAAGTGTA
CAGCAAGGCAAACGTGTGAAGACCACTGTGGAACGTGCTCGACTAAACAGAGGAGTAAAGAAC------------
CAGGCAAAGATAAACAACAAAAATTTAATGAAAGAGATTGTCAATTGCAAGAATATACATCTCAGCACCAAGCTGCTTGC
TGTTGATTACCCAGTAGATTTCATTAAATCAATTTCTTGCCAGATTTGTGAGCATATTTTGGCAGATCCAGTGGAAACAA

CATGCAGACACTTGTTTTGCAGAACTTGCATCCTTAAATGTATCAAGGTTATGGGCAGCTATTGCCCCTCCTGCTGGTAT
CCTTGCTTCCCRACTGATCTGGTAACCCCAGTGAAATCCTTCCTGAACATCCTTGATAGCCTGGGTATAAGATGCCCTGT
AAAGGAATGTGATGAAGAGATTTTGCATGGAAAATATGGCCAACACCTCTCCAACCACAAGGAGATGAAAGATAGAGAGC
TCTATAGCTATGTGAATAAAGGTGGCCGACCAAGGCAGCATCTCCTGTCTTTGACAAGGAGAGCTCAGAAACATCGTCTG
AGGGAACTGAAACGTCAAGTCAAGGCTTTTGCCGAGAAAGAAGAGGGTGGTGATATAAAGGCTGTATGCATGACTTTGTT
CCTTCTAGCTTTAAGAGCAAAAAACGAACACAGACAAGCAGATGAATTGGAGGCTATAATGCAAGGGAGGGGATCTGGAC
TTCATCCTGCTGTCTGTCTGGCAATCCGAGTTAACACATTTCTCAGCTGTAGCCAGTACCATAAAATGTATAGAACTGTA
AAAGCTGTCACTGGCAGGCAGATCTTCCAGCCCTTGCACGCTCTTCGCACTGCTGAGAAAGCCCTCCTACCAGGTTATCA
CCCATTTGAGTGGAAACCTCCCTTGAAAAACGTATCCACTAATACAGAAGTAGGMATTATAGATGGTCTATCAGGATTGC
CACTTTCAGTTGATGACTACCCAGTAGACACAATTGCAAAGAGATTTCGGTATGATGCAGCCTTGGTTTGTGCCTTAAAG
GACATGGAGGAGGAGATCTTGGAAGGCATGAAAGCAAAAAATCTGGACGATTATTTGAATGGCCCCTTCACAGTGGTAAT
AAAAGAGTCCTGTGATGGAATGGGAGATGTCAGTGAGAAGCATGGAAGTGGGCCTGCTGTCCCAGAGAAGGCTGTTCGCT
TTTCTTTCACAATTATGAACATTGCTATAGCACATGAGAATGAAAGCAAGAGGATCTTTGAGGAAATAAAGCCAAATTCA
GAGTTGTGTTGCAAGCCCTTGTGCCTCATGCTGGCTGATGAATCAGATCATGAAACTCTAACAGCAATCCTGAGCCCCCT
CATAGCAGAAAGAGAGGCTATGAAAAACAGTGAACTGCTGCTTGAAATGGGAGGCATCCTGAGAACATTCAGATTTGTCT
TTAGGGGTACAGGGTATGATGAGAAACTTGTGCGGGAAGTGGAAGGGCTGGAGGCCTCAGGTTCCACTTACATTTGTACC
CTGTGTGATGCAACTCGCTTGGAGGCATCCCAGAATTTGGTCTTCCACTCCATAACCAGGAGCCATGCTGAAAATCTGGA
GCGATATGAAATATGGAGGTCCAACCCATACCATGAATCTGTTGATGAGCTCCGTGACAGAGTGAAGGGTGTTTCAGCCA
AACCTTTTATTGAGACTGTTCCCTCCATAGATGCGTTGCACTGTGACATTGGCAATGCAACAGAATTCTACAGAATTTTC
CAGATGGAGATTGGTGAACTTTACAAGAACCCTGATGTGTCTAAAGAGGAGAGGAAGAGGTGGCAGTTGACTCTTGACAA
ACACCTCAGGAAGAAGATGAACTTGAAGCCTATGATGAAGATGAGTGGAAATTTTGCTAGAAAGCTCATGTCCAAAGAGG
CAGTAGAGGCAGTATGTGAATTAATAAAGTGTGAGGAAAGGCATGAAGCCCTAAAAGAACTAATGGACCTTTATCTGAAG
ATGAAGCCAGTGTGGCGATCCTCATGCCCTGCCAAGGAGTGCCCGGAACTGCTGTGCCAGTATAGCTACAATTCACAGCG
TTTTGCTGAGCTCTTATCTACAAAGTTCAAGTACAGATATGAAGGCAAGATTACAAATTATTTCCACAAAACACTTGCTC
ATGTTCCTGAAATCATTGAAAGAGATGGGTCTATAGGGGCCTGGGCAAGTGAAGGAAATGAGTCTGGAAACAAACTGTTT
AGGAGATTCCGAAAAATGAATGCCAGGCAGTCCAAATGCTATGAGATGGAGGATGTCTTG
Myiarchus   TAAAGTGCGATCATTTGAAAAAACAA----------------------------
----
CTGATGACAGGCAGCACATAAGCAAAGATCAGGCAGAAGGGATTGCTTCTTCAAACAAAGAAATCATACTGCATAAAGAT
AAAGCAGTGTCAAGAGAAGAAAAGATGGAGTTAATGGGCAATAGGCAGGCACTTGAGGAAGAGGCCCATGACATGAAAAC
ACAAGACAATAGAGCTCATCAGAACAATCTGAAGCAACTTTGTCGCATCTGTGGAGTTTCATTTAAAACTGATTGCTACA
AGAGAACTCATCCAGTGCATGGGCCGGTGGATGATGAAACTCTGTGTCTTCTGAGAAAGAAAGAAAAAAAAGCAACCTCT
TGGCCAGATCTTATAGCTAAGGTGTTCAAGATTGATGTGCGAGGGGATGTTGATACTGTCCATCCCACTCGGTTTTGTCA
CAACTGTTGGAGTATTATCCATAGGAAATACAGTAGTACTCTATGTGAGGTATATTTTCCTAGGAACAGCACCATGGAGT
GGCAACCTCACTCCCCAAACTGTGATGTATGCCATACTACCAGTCGAGGGGTCAAGAGAAAAAGCCAGCCACCAAGTGTA
CAGCAAGGCAAACGTGTGAAAACCATTGTGGAACGCGCTCGACTAAACAGAGGTGTAAAGAAC------------
CAGGCACAGATAAACAACAAAAATTTAATGAAAGAGATTGTCAATTGCAAGAATATACATCTCAGCACCAAGCTGCTTGC
TGTTGATTACCCAGTAGATTTCATTAAATCAATTTCTTGCCAGATTTGTGAGCATATTTTGGCAGATCCAGTGGAAACAA
CATGCAGACACTTGTTTTGCAGAACTTGTATCCTTAAATGTATCAAGGTTATGGGCAGCTATTGCCCCTCCTGCTGGTAT
CCTTGCTTCCCGACTGATCTGGTAACCCCAGTGAAATCCTTCCTGAACATCCTTGATAGCCTGGGTATAAGATGCCCTGT
AAAGGAATGTGATGAAGAGATTTTGCATGGAAAATATGGCCAACACCTCTCCAACCACAAGGAGATGAAAGATAGAGAGC
TCTATAGCTATGTAAATAAAGGTGGCCGACCAAGGCAGCATCTCCTGTCTTTGACAAGGAGAGCTCAGAAACATCGTCTG
AGGGAACTGAAACGTCAAGTCAAGGCTTTTGCCGAGAAAGAAGAGGGTGGTGATATAAAGGCTGTATGCATGACTTTGTT
CCTTCTAGCTTTAAGAGCAAAAAACGAACACAGACAAGCAGATGAATTGGAGGCTATAATGCAAGGGAGGGGATCTGGAC
TTCATCCTGCTGTCTGTCTGGCAATCCGAGTTAACACGTTTCTCAGCTGTAGCCAGTACCATAAAATGTATAGAACTGTA
AAAGCTGTCACTGGGAGGCAGATCTTCCAGCCCTTGCATGCTCTTCGCACTGCTGAGAAAGCCCTCCTACCAGGTTATCA
CCCATTTGAGTGGAAACCTCCCTTGAAAAACGTATCTACTAATACAGAAGTAGGAATTATAGATGGTCTATCAGGATTGC
CACTTTCAGTTGATGACTACCCAGTAGACACAATTGCAAAGAGATTTCGATATGATGCAGCCTTGGTTTGTGCCTTAAAG
GACATGGAGGAGGAGATCTTGGAAGGCATGAAAGCAAAAAATCTGGACGACTATTTGAATGGCCCCTTCACTGTGGTAAT
AAAAGAGTCCTGTGATGGAATGGGAGATGTCAGTGAGAAGCATGGAAGTGGGCCTGCTGTCCCAGAGAAGGCTGTTCGCT
TTTCTTTCACAATTATGAACATTGCTATAGCACATGGGAATGAAAGCAAGAGGATCTTTGAGGAAATAAAGCCAAATTCA
GAGTTGTGTTGCAAGCCCTTGTGCCTTATGCTGGCTGATGAATCAGATCATGAAACTCTAACAGCAATCCTGAGCCCCCT
CATAGCAGAAAGAGAGGCTATGAAAAACAGTGAACTGCTGCTTGAAATGGGAGGCATCCTGAGAACATTCAGATTTGTCT
TTAGGGGTACAGGGTATGATGAGAAACTTGTGCGGGAAGTRGAAGGGCTGGAGGCCTCAGGTTCCACTTACATTTGTACT
CTGTGTGATGCAACTCGCTTGGAGGCATCCCAGAATTTGGTCTTCCACTCCATAACCAGGAGCCATGCTGAAAATCTGGA
GCGATATGAAATATGGAGGTCCAACCCATACCATGAATCTGTTGATGAGCTCCGTGACAGAGTGAAGGGTGTTTCAGCCA
AACCTTTTATTGAGACTGTTCCCTCCATAGATGCATTGCACTGTGACATTGGCAATGCAACAGAATTCTACAGGATTTTC
CAGATGGAGATTGGTGAACTTTACAAGAACCCTGATGTGTCTAAAGAGGAGAGGAAGAGGTGGCAGTTGACTCTTGACAA
ACACCTCAGGAAGAAGATGAACTTGAAGCCTATGATGAAGATGAGTGGAAATTTTGCTAGAAAGCTCATGTCCAAAGAGG
CAGTAGAGGCAGTATGTGAATTAATAAAGTGTGAGGAAAGGCATGAAGCCCTAAAAGAACTAATGGACCTTTATCTGAAG

ATGAAGCCAGTGTGGCGATCCTCATGCCCTGCCAAGGAGTGCCCAGAACTGCTGTGCCAGTATAGCTACAATTCACAGCG
TTTTGCTGAGCTCTTATCTACAAAGTTCAAGTACAGATATGAAGGCAAGATTACAAATTATTTCCACAAAACACTTGCTC
ATGTTCCTGAAATCATTGAAAGAGATGGGTCCATAGGGGCCTGGGCAAGTGAAGGAAATGAGTCTGGAAACAAACTGTTT
AGGAGATTCCGAAAAATGAATGCCAGGCAGTCCAAATTCTATGAGATGGAGGATGTCTTG
Hypocnemis_cantator TAAAGTGCGATCATTTGAAAAAACACCTT-------------------------
----
CTGATGACAGGCAGCACATAAACAAAGATCAGGCAGAAGGGGTTGCTTCTTCAAACAAAGAAATCATACTGCACACAGAT
GAAGCAGTGCCAAGAAGAGAAAAGATGGAGTTAATGGGCAATAGGCAGGCCCTTGAGAAAGAGGCCTGT-----------
----
GACAATAGAGCTCATCAGAACAATCTGAAACAACTTTGCCGCATCTGTGGAGTTTCATTTAAAACTGATTGTTACAAGAG
AACTCATCCAGTGCATGGGCCGGTGGATGATGAAACTCTGTGGCTTTTGAGAAAGAAAGAAAAAAAAGCAACCTCTTGGC
CAGATCTTATTGCTAAGGTTTTCAAGATTGATGTGCGAGAGGATGTTGATACTATCCATCCCACTCGATTTTGTCACAAC
TGTTGGAGTATTATCCATAGAAAATTCAGTAATACTCTAGGTGAAGTATATTTTCCTAGGAACAGCACAATGGAGTGGCA
ACCTCACTCCCCAAACTGTGATGTATGCCATACTACCACTCGAGGGGTCAAGAGAAAAAGCCAGCCACCAAGTGTACAGC
ATGGCAAACGTGCGAAGACCATTGTGGAACGTGCTCGACTAAACAGAGGTGTGAAGAAC------------
CAAGCACAGATAAACAACAAAAATTTAATGAAAGAGATTGTCAATTGCAAGAATACACATCTCAGCACCAAGCTGCTTGC
AGTTGATTACCCAATAGATTTCATTAAATCAATTTCTTGCCAGATTTGTGATCATATTTTGGCAGATCCAGTGGAAACAA
CATGCAGACACTTATTTTGCAGGACTTGCATCCTTAAATGTATTAAGGTTATGGGCAGCTATTGCCCCTCCTGCTGGTAT
CCTTGCTTCCCTACTGATCTGGTGACACCAGTGAAATCCTTCCTGAACATCCTTGATAGCCTGGGTATAAGATGCCCTGT
AAAGGAATGTGATGAAGAGATCTTGCATGGAAAATATGGCCAACACATCTCCAGCCATAAGGAGAAGAAAGATAGAGAGC
TCTATAGCCACATAAATAAAGGTGGCCGACCAAGGCAGCATCTCCTATCTTTGACAAGGAGAGCTCAGAAACATCGTCTG
AGGGAACTGAAACGTCAAGTCAGGGCTTTTGCTGAGAAAGAAGAGGGTGGTGATATAAAGGCTGTATGCATGACTTTGTT
CCTTCTAGCTTTAAGAGCGAAAAATGAACACAAACAAGCAGATGAACTGGAGGCTATAATGCAAGGGAGAGGATCTGGAC
TTCATCCTGCTGTCTGTCTGGCAATCCGAGTCAACACATTTCTCAGCTGTAGCCAGTATCATAAAATGTACAGAACTGTA
AAAGCTGTCACTGGGAGGCAGATCTTCCAGCCCTTGCATGCTCTTCGCACTGCTGAGAAAGCCCTCCTACCAGGCTATCA
CCCATTTGAGTGGAAACCTCCCTTGAAAAATGTATCCACTAATACAGAAGTGGGAATTATAGATGGATTATCAGGACTGC
CACTCTCAATCGATGACTACCCAGTAGACACAATTGCAAAGAGATTTCGATATGATGCAGCCTTGGTTTGTGCTTTAAAG
GACATGGAGGAGGAGATCTTGGAAGGCATGAAAGCAAAAAATCTGGAYGACTATTTGAATGGTCCCTTCACTGTGGTAGT
AAAAGAGTCCTGTGATGGAATGGGAGATGTCAGTGAGAAGCATGGAAGTGGGCCTGCTGTCCCAGAGAAGGCTGTTCGCT
TTTCATTCACAGTTATGAACATCGCTATTGCAAATGGGAATGAAAGCCAGAGAATCTTTGAGGAAGTAAAGCCCAATTCA
GAGTTGTGTTGCAAACCCTTATGCCTTATGCTGGCTGATGAATCAGATCATGAAACTCTGACAGCAATCCTGAGCCCACT
CATAGCAGAGAGAGAGAGGCTATGAAAAACAGTGAACTATTGCTTGAAATGGGAGGCATCCTGAGAACATTCAGATTTGTCT
TCAGGGGTACAGGATATGATGAAAAACTTGTACGGGAAGTGGAAGGGCTGGAGGCCTCAGGTTCCACTTACATTTGTACC
CTTTGTGATGCAACTCGCTTGGAGGCATCCCAGAATTTGGTCTTCCACTCCATAACGAGGAGCCATGCTGAAAATCTAGA
ACGATATGAGATATGGAGGTCCAACCCATATCACGAATCTGTTGATGAGCTCCGTGACAGAGTGAAGGGTGTTTCAGCCA
AACCTTTTATTGAGACTGTTCCCTCCATAGATGCATTGCACTGTGACATTGGCAATGCAGCAGAATTCTACAGGATTTTC
CAGATGGAGATTGGTGAACTTTACAAGAATCCTGACGTTTCTAAAGAGGAGAGGAAGAGGTGGCAGTTGACTCTTGACAA
ACACCTCAGGAAGAAGATGAACTTGAAGCCTATGATGAGGATGACTGGAAATTTTGCTAGAAAGCTTATGTCCAAAGAGA
CAGTAGAGGCAGTATGTGAATTAATAAAGTGTGAGGAAAGGCATGAAGCCCTAAAAGAACTAATGGACCTTTATCTGAAG
ATGAAGCCAGTGTGGCGATCCTCATGCCCTGCCAAGGAGTGCCCAGAACTGCTGTGCCAGTATAGCTACAATTCACAGCG
TTTTGCTGAGCTCTTATCTACAAAGTTCAAGTACAGATATGAAGGCAAGATTACAAATTATTTCCATAAAACACTTGCTC
ATGTTCCTGAAATCATTGAAAGAGATGGGTCCATTGGGGCCTGGGCAAGTGAAGGAAATGAGTCTGGAAACAAACTGTTT
AGGAGGTTCCGAAAAATGAATGCCAGGCAGTCCAAATGCTATGAGATGGAGGATGTCTTG
Phlegopsis TAAAGTGCGATCATTTGAGAAAACATCCT-------------------------
----
CTGATGACAGGCAGCACATAAACAAAGATCAGGCAGAAGGGGTTGCTTCTTCAAACAAAGAAATCATACTGCACACAGAT
GAAGCAGTGCCAAGAACAGAAAAGATGGAGTTAATGGGCAATAGGCAGGCCCTTGAGAAAGAGGCCCAT-----------
----
GACAATAGAGCTCATCAGAACAATCTGAAACAACTTTGCCGCATCTGTGGAGTTTCATTTAAAACTGATTGTTACAAGAG
AACTCATCCAGTGCATGGGCCAGTGGATGATGAAACTCTSTGGCTTCTGAGAAAGAAAGAAAAAAAAGCAACCTCTTGGC
CAGATCTTATCGCTAAGGTTTTCAAGATTGATGTGCGAGGGGATGTTGATACTATCCATCCCACTCGATTTTGTCACAAC
TGTTGGAGTATTATCCATAGAAAATTCAGTAATACTCTAGGTGAAGTATATTTTCCTAGGAACAGCACAATGGAGTGGCA
ACCTCACTCCCCAAACTGTGATGTATGCCATACTACCACTCGAGGGATCAAGAGAAGAAGCCAGCCACCAAGTGTACAGC
ATGGCAAACGTGCAAAGACCATTGTGGAACATRTTCGACTAAACAGAGGCGTGAAGAAC------------
CAAGCACAGATAAACAACAAAAATTTAATGAAAGAGATTGTCARTTGCAAGAATACACATCTCAGCACCAAGCTGCTTGC
AGTTGATTACCCAATTGATTTCATTAAATCAATTTCTTGCCAGATTTGTGATCATATTTTGGCAGATCCAGTGGAAACAA
CATGCAGACACTTATTTTGCAGGACTTGCATCCTTAAATGTATCAAGGTTATGGGCAGCTATTGCCCCTCCTGCTGGTAT
CCTTGCTTCCCTACTGATCTGGTGACACCAGTGAAATCCTTCCTGAACATCCTTGATAGCCTGGGTATAAGATGCCCTGT
AAAGGAATGTGATGAAGAGATCTTGCATGGAAAATATGGCCAACACATCTCCAGCCACAAGGAGAAGAAAGATAGAGAGC
TCTATAGCCACATAAATAAAGGTGGCCGACCAAGGCAGCATCTCCTGTCTTTGACAAGGAGAGCTCAGAAACATCGTCTG

AGGGAACTGAAACGTCAAGTCAGGGCTTTTGCTGAGAAAGAAGAGGGTGGTGATATAAAGGCTGTATGCATGACTTTGTT
CCTTCTAGCTTTAAGAGCGAAAAATGAACACAAACAAGCAGATGAATTGGAGGCTATAATGCAAGGGAGAGGATCTGGAC
TTCATCCTGCTGTCTGTCTGGCAATCCGAGTCAACACATTTCTCAGCTGTAGCCAGTATCATAAAATGTACAGAACTGTA
AAAGCTGTCACTGGGAGGCAGATTTTCCAGCCCTTGCATGCTCTTCGCACTGCTGAGAAAGCCCTCCTACCAGGCTATCA
CCCATTTGAGTGGAAACCTCCCTTGAAAAATGTATCCACTAATACAGAAGTGGGAATTATAGATGGATTATCAGGACTGT
CACTCTCAATCGATGACTACCCAGTAGACACAATTGCAAAGAGATTTCGATATGATGCAGCCTTGGTTTGTGCCTTAAAG
GACATGGAGGAGGAGATCTTGGAAGGCATGAAAGCAAAAAATCTGGACGACTATTTGAATGGTCCCTTCACTGTGGTAGT
AAAAGAGTCCTGTGATGGAATGGGAGATGTCAGTGAGAAGCATGGAAGTGGGCCTGCTGTCCCAGAGAAGGCTGTTCGCT
TTTCATTCACAGTTATGAACATCGCTATTGCAAATGGGAATGAAAGCCAGAGAATCTTTGAGGAAGTAAAGCCCAATTCA
GAGTTGTGTTGCAAACCCTTATGCCTTATGCTGGCTGATGAATCAGATCATGAAACTCTGACAGCAATCCTGAGCCCACT
CATAGCAGAAAGAGAGGCTATGAAAAACAGTGARCTATTGCTTGAAATGGGAGGCATCCTGAGAACATTCAGATTTGTCT
TTAGGGGTACAGGATATGATGAAAAACTTGTACGGGAAGTGGAAGGGCTGGAGGCCTCAGGTTCCACTTACATTTGTACC
CTCTGTGATGCAACTCGCTTGGAGGCATCCCAGAATTTGGTCTTCCACTCCATAACGAGGAGCCATGCAGAAAATCTGGA
ACGATATGAAATATGGAGGTCCAACCCAYATCACGAATCTGTTGATGAGCTCCGTGACAGAGTGAAGGGTGTTTCAGCCA
AACCTTTTATTGAGACTGTTCCCTCCATAGATGCATTGCACTGTGACATTGGCAATGCAGCAGAATTCTACAGGATTTTC
CAGATGGAGATTGGTGAACTTTACAAGAATCCTGACGTGTCTAAAGAGGAGAGGAAGAGGTGGCAGTTGACTCTTGACAA
ACACCTCAGGAAGAAGATGAACTTGAAGCCTATGATGAGGATGACTGGAAATTTTGCTAGAAAGCTTATGTCCAAAGAGA
CAGTAGAGGCAGTATGTGAATTAATAAAGTGTGAGGAAAGGCATGAAGCCCTAAAAGAACTAATGGATCTTTATCTGAAG
ATGAAGCCTGTGTGGCGATCCTCATGCCCTGCCAAGGAGTGCCCAGAACTGCTGTGCCAGTATAGCTACAATTCACAACG
TTTTGCTGAGCTCTTATCTACAAAGTTCAAGTACAGATATGAAGGCAAGATTACAAATTATTTCCATAAAACACTTGCTC
ATGTTCCTGAAATCATTGAAAGAGATGGGTCCATTGGGGCCTGGGCAAGTGAAGGAAATGAGTCTGGAAACAAACTGTTT
AGGAGGTTTCGAAAAATGAATGCCAGGCAGTCCAAATGCTATGAGATGGAGGATGTCTTG
Manacus_manacus TAAAGTGCGATCATCTGAAAAAATGCCCT-------------------------
----
CTGATGACAGGCAGCACATAAACAAAGATCAGGCAGAAGGGATTGCTTCTTCAAACAAAGAAATCATACTGCATAAAGAT
GAAACAGTGTCAAGAGAWGAAAAGATGGAGTTAATGACCAATAGGCAGGCACTTGAGAAAGAGRCCCATGACATGAAAAC
ACGAGACAATAGAGCTCATCAGAACAATCTGAAGCAACTTTGTCGCATCTGTGGAGTTTCATTTAAAACTGATTGCTACA
AGAGAACTCATCCAGTGCATGGGCCGGTGGATGATGAAACTCTGTGTCTTCTGAGAAAGAAAGAAAAAAAAGCAACCTCT
TGGCCAGATCTTATTGCTAAGGTTTTCAAGATTGATGTGCGAGGGGATGTTGATACTATCCATCCCACTCGGTTTTGTCA
CAACTGTTGGAGTATTATCCATAGAAAATACAGTAATACCCTATGTGAGGTATATTTTCCTAGGAACAGCACGATGGAGT
GGCAACCTCACTCCCCAAACTGTGATGTATGCCATACTACCAGTCGAGGGGTCAAGAGAAAAAGCCARCCATCAAGTGTA
CAACAAGGCAAACGTGTGAAGACCATTGTGGAACGTGCTCGACTAAACAGAGGCGTAAAGAAC------------
CAGGCACAGATAAACAACAAAAATTTAATGAAAGAGATTGTCAATTGCAAGAATATACATCTCAGCACCAAGCTGCTTGC
AGTTGATTACCCAGTAGATTTCATTAAATCAATTTCTTGCCAGATTTGTGAGCATATTTTGGCAGATCCAGTGGAAACAA
CATGCAGACACTTGTTTTGCAGAACTTGCATCCTTAAATGTATCAAGGTTATGGGCAGCTATTGCCCCTCCTGCTGGTAT
CCTTGCTTCCCTACTGATCTGGTAACCCCAGTGAAATCCTTCCTGAACATCCTTGATAGCCTGGGTATAAGATGCCCTGT
AAAGGAATGTGATGAAGAGATTTTGCATGGAAAATATGGCCAACACCTCTCCAACCACAAGGAGATGAAAGATAGAGAGC
TCTATAGCTACATAAATAAAGGTGGCCGACCAAGGCAGCATCTCCTATCTTTGACAAGGAGAGCTCAGAAACATCGTCTA
AGGGAACTGAAACGTCAAGTCAAGGCTTTTGCTGAGAAAGAAGAGGGTGGTGATATAAAGGCTGTATGCATGACTTTGTT
CCTTCTAGCTTTAAGAGCAAAAAATGAACACAGAAAAGCAGATGAATTGGAGGCTATAATGCAAGGGAGGGGATCTGGAC
TTCATCCTGCTGTCTGTCTGGCAATCCGAGTCAACACATTTCTCAGCTGTAGCCAGTACCATAAAATGTATAGAACTGTA
AAAGCTGTCACTGGGAGGCAGATCTTCCAACCCTTGCATGCTCTTCGCACTGCTGAGAAAGCCCTCCTACCAGGTTATCA
CCCATTTGAATGGAAACCTCCCTTGAAAAATGTATCCACTAATACAGAGGTAGGAATTATAGACGGTCTATCAGGATTGC
CACTCTCAGTTGATGACTACCCAGTAGACACAATTGCAAAGAGATTTCGATATGATGCAGCCTTGGTTTGTGCCTTAAAG
GACATGGAGGAGGAGATCTTGGAAGGCATGAAAGCAAAACATCTGGACGACTATTTGAATGGTCCCTTCACYGTGGTAAT
AAAAGAGTCCTGTGATGGAATGGGAGATGTCAGTGAGAAGCATGGAAGCGGGCCTGCTGTCCCAGAGAAGGCTGTTCGCT
TTTCTTTCACAGTTATGAACATTGCTATAGCACATGGGAATGAAAGCAAGAGGATCTTTGAGGAAGTAAAGCCAAATTCA
GAGTTGTGTTGCAAGCCCTTGTGCCTTATGCTGGCTGACGAATCAGATCATGAAACTCTAACGGCAATCCTGAGCCCCCT
CATAGCAGAAAGAGAGGCTATGAAAAACAGTGAACTGCTRCTTGAAATGGGAGGCATCCTGAGAACATTCAGATTTGTCT
TTAGGGGTACAGGATATGATGAGAAACTTGTGCGGGAAGTGGAAGGGCTGGAGGCCTCAGGTTCCACTTACATTTGTACC
CTGTGTGATGCAACCCGCTTGGAGGCATCCCAGAATTTGGTCTTCCACTCCATAACCAGGAGCCATGCTGAAAATCTGGA
GCGATATGAAATATGGAGGTCCAACCCATATCACGAATCTGTTGATGAGCTCCGTGACAGAGTGAAAGGTGTTTCAGCCA
AACCTTTTATTGAGACCGTTCCCTCCATAGATGCATTGCACTGTGACATTGGCAATGCAACAGAGTTCTACAGGATTTTC
CAGATGGAGATTGGTGAACTTTACAAGAATCCTGATGTGTCTAAAGAGGAGAGGAAGAGGTGGCAGTTGACTCTTGACAA
ACACCTCAGGAAGAAGATGAACTTGAAGCCTATGATGAAGATGAGTGGAAATTTTGCTAGAAAGCTCATGTCCAAAGAGG
CAGTAGAGGCAGTATGTGAATTAATAAAGTGTGAGGAAAGGCATGAAGCCCTAAAAGAACTAATGGACCTCTATCTGAAG
ATGAAGCCAGTGTGGCGATCCTCATGCCCTACCAAGGAGTGCCCAGAACTGCTGTGCCAGTATAGCTACAATTCACAGCG
TTTTGCTGAGCTCTTATCTACAAAGTTCAAGTACAGATATGAAGGCAAGATTACAAATTATTTCCACAAAACACTTGCTC
ATGTTCCTGAAATCATTGAAAGAGATGGATCCATAGGGGCCTGGGCAAGTGAAGGAAATGAGTCTGGAAACAAACTGTTT
AGGAGGTTCCGAAAAATGAATGCCAGGCAGTCCAAATTCTATGAGATGGAGGATGTCTTG

Acanthisitta_chloris TAAAGTGCGATCGTTTGATAAAACACCCT------------------------
----
CCGATGGCAACCAGCACATAAACAAAGATCAGGCAGAAGAGGTTGCTTCTTCAAACAAAGAAATCATACTGCATGAAGAT
GAAGTGGTGCCAAGAGGAGAAGGGATGGAGTTAATGGGCAACAGGGAGGCAGTCAAGAAAGATGCCCATGACATGAAGAC
ACAAGACAACAGAGATCACCAGAGCAATCTGGAGCAACTTTGCCGCATCTGTGGAGTTTCATTTAAAACTGATTGTTACA
AGAGAACTTATCCAGTACATGGGCCAGTGGATGATGAAACTCTGTGGCTCCTGAGAAAGAAAGAAAAAACAGCAACCTCT
TGGCCAGATCTTATAGCTAAGGTTTTTAAGATTGATGTGCGAGGGGATGTYGATACTATCCATCCCACTCGATTTTGTCA
CAAYTGCTGGAGTATTATCCACAGGAAATTCAGTAATACWCTATGTGAAGTATATTTTCCTAGGAACAGCACAGTGGAGT
GGCAGCCTCACTCTCCAAACTGTGATGTGTGCCATACTACCCGACAAGGAGTCAAGAGAAAAAACCAACAACCGAGAGTG
CAACATGGCAAACGTGTGAAGACCATTGTGGAATGTGCTCGACTAAACAGAGGTATAAAGAAC------------
CAAGCACAGATAAACAACAAAAATTTAATGAAAGAGATTGTCAATTGCAAAAATATACATCTCAGCACCAAGATGCTTAC
AGTTGATTACCCAGAGGATTTCATTAAATCCTTATCTTGCCAGATTTGTGAACATATTTTGGCAGATCCAGTGGAAACAA
CATGCAGACACTTGTTTTGCAGAACTTGCATCCTTAAATGTATCAAGGTTATGGGCAGCTATTGTCCTTCCTGCTGGTAT
CCTTGTTTTCCTACTGATCTGGAAATACCAGTGAAATCCTTCCTGAACACCCTTGATAGCCTGAGTATAAGATGCCCTGT
AAAAGACTGTGATGAAGAAATCAGGTATGGAAAATATAGCCAACACCTCTCCAGCCACAAGGAGATGAAAGATAGAGAGC
TCTACATCCACATAAATAAAGGTGGCAGACCGAGGCAGCATCTCCTAACTTTGACCAGGAGAGCTCAGAAACATCGCCTG
AGGGAACTGAAACGTCAAGTCAAGGCTTTTGCTGAGAAAGAAGAGGGTGGTGATATAAAGGCTGTATGCATGACTTTGTT
CCTGCTAGCTTTAAGAGCAAAAAATGAACACAGACAAGCAGATGAACTGGAGGCTATAATGCAAGGGAGGGGATCTGGAC
TTCACCCTGCTGTCTGCCTGGCAATTCGAATCAACACATTTCTCAGCTGTAGCCAGTATCATAAAATGTATAGAACAGTA
AAAGCTGTCACTGGGAGGCAAATCTTCCAGCCTTTGCATTCTCTTCGCACTGCTGAGAAAGCTCTCCTGCCAGGTTATCA
TCCATTTGAGTGGAAACCTCCTTTGAAAAATGTATCCACTAACACAGAAGTGGGAATTATAGATGGGCTATCAGGATTGC
CACTCTCAATTGATGACTACCCAGTAGACACAATTGCAAAGAGATTTCGATATGATGCAGCCTTGGTTTGTGCCTTAAAG
GACATGGAGGAGGAGATCTTGGAAGGCATGAAAGCAAAACACCTGGATGACTATTTGAATGGTCCCTTCACTGTGGTAGT
CAAAGAGTCCTGTGATGGAATGGGAGATGTCAGTGAGAAGCATGGCAGTGGGCCTGCTGTCCCAGAGAAGGCTGTTCGCT
TTTCTTTCACAGTCATGAGCATTGCTATAGCACAGGGGAATGAAAACAAGAGGATCTTTGAGGAAGGAAAGCCCAATTCA
GAGTTGTGTTGCAAGCCCTTGTGCCTTATGCTGGCTGACGAATCAGATCATGAAACTCTGACAGCGATCCTGAGCCCCCT
CATTGCAGAAAGAGAGGCTATGAAAAGCAGTGAACTGCTGCTTGAAATGGGAGGTATCCTGAGAACATTCAGGTTCATCT
TTAGAGGTACAGGATATGATGAGAAACTCGTGCGGGAAGTGGAAGGGCTGGAGGCCTCAGGTTCCACTTACATTTGCACC
CTGTGTGATGCAACCCGCTTGGAGGCATCCCAGAATTTGGTCTTCCACTCCATCACCAGGAGTCATGCTGAAAATCTGGA
GCGATATGAAATTTGGAGGTCCAACCCATATCATGAATCTGTTGATGAGCTCCGCAACAGAGTGAAGGGTGTTTCGGCCA
AACCTTTTATTGAGACTGTTCCCTCTATAGATGCATTGCACTGTGACATTGGCAATGCAGCAGAATTCTACAGGATTTTC
CAGATGGAGATCGGTGAACTTTACAAGAATCCTGATGTGTCTAAAGAGGAGAGGAAGAGGTGGCAGTTGGCTCTTGACAA
ACACCTCAGGAAGAAGATGAACTTGAAGCCTATGCTGAGGATGAGTGGGAATTTTGCTAGAAAGCTCATGTCCAAAGAGA
CAGTAGAGGCAGTATGTGAATTAATAAAGTGTGAGGAAAGACATGAAGCCCTAAAAGAACTAATGGACCTTTATCTGAAG
ATGAAGCCAGTGTGGCGATCCTCATGCCCTGCCAAGGAGTGCCCTGAACTGCTGTGCCAGTATAGCTACAATTCACAGCG
TTTTGCTGAGCTCTTATCTACAAAGTTCAAGTACAGATATGAAGGCAAGATTACAAATTATTTCCACAAAACACTTGCTC
AYGTTCCTGAAATCATTGAAAGAGATGGGTCTATTGGGGCCTGGGCAAGTGAAGGAAATGAGTCTGGTAACAAACTGTTT
AGGAGGTTCCGAAAAATGAATGCCAGGCAGTCCAAATGCTATGAGATGGAGGATGTCTTG
Regulus TAAATTACGATCATTTGAAAAAACACACT------------------------
----
CTGATGACAGCCAGAACATAAACAAAKATCAGGCAGAAGAGGCTGTTTCTTCAAACAAAGAAGTCATCCTGCATAAAGAT
GAAGCAGTGCCAAGAGGAGAAAAGATGGAGTTAATGGGCAGGAGGCAGGGACTTGAGGAAGATGCCCATGCCATGAAAAC
ACAGGACAATAGAGCTCATCAGAACAATCTGAAGCAACTCTGCCGCATCTGTGGAGTTTCATTTAAAACTGATTGTTACA
AGAGAACTTACCCAGTGCATGGGCCAGTGGATGATGAAACTCTGTGGCTTCTGAGAAAGAAAGAAAAAACAGCAACCTCT
TGGCCYGATCTTATTGCTAAGGTTTTCAAGATTGATGTGCGAGGGGATGTCGATACTATCCATCCCACTCGATTTTGTCA
CAACTGCTGGAGTATTATACATAGAAAATTCAGTAATACTCTATGTGAAGTATATTTTCCTAGGAACAGCACAATGGAGT
GGCAACCCCATTCCCCCAGCTGTGACGTGTGCCATACTACCAGACRAGGAGTCAAGAGAAAAAGTCAGCCCCCRAGAGTA
CTACATGGCAAACGTGTCAAAACCACTGGGGAACGTGCTCAGCTAAACAGAGGGGTAAAGAAC------------
CAAGCACAGATAAACAACAAAAATTTAATGAAAGAGATTGTCAATTGCAAGGATAGACATCTAAACACCAAGCTGCTTGC
AGTAGATTACCCAGTAGATTTCATTAAATCTGTTTCTTGCCAGATTTGTGATCATATTTTGGCAGATCCAGTGGAAACAA
CATGCAGACACTTGTTTTGCAGAACTTGCATCCTTAAATGTATCAGGATTATGGGCAGCTATTGCCCCTCCTGCTGGTAT
CCTTGCTTTCCTCCTGATCTGGTAACCCCAGTGAAATCCTTCCTGAACATCCTCRATAACCTGATTATWAGATGCCCTGT
AAAAGAGTGTGATGAGGAGATCTTGCATGGAAAATATGGCCAACACCTCTCTGGCCACAAGGAGATGAAAGATGGAGAGC
TCTATAGCTACATAAATAAAGGTGGCCGACCRAGGCAGCATCTCCTGTCTTTGACMAGGAGAGCTCAGAAACATCGTCTG
AGGGAACTGAAACGTCAAGTTAAGGCTTTTGCTGAGAAAGAAGAGGGTGGTGATATAAAGGCTGTATGCATGACTTTGTT
CCTGCTAGCTTTGAGAGCAAAAAATGAACACAAACAAGCAGATGAACTGGAGGCTATAATGCAAGGGAGGGGATCTGGAC
TTCATCCTGCTGTCTGTCTGGCCATCCGAATCAACACGTTTCTCAGCTGTAGTCAGTATCATAAAATGTACAGAACAGTA
AAAGCTGTCTCTGGGAGACAAATCTTCCAGCCTTTGCATGCTCTTCGCACTGCCGAGAAAGCCCTCCTACCAGGTTATCA
CCCATTTGAGTGGAAACCTCCCCTGAAAAATGTATCCAATAACACAGAAGTGGGAATTATAGATGGACTATCAGGACTGC
CACTCTCAATTGATGATTACCCAGTAGACACAATTGCAAAGAGATTCCGATATGATGCAGCCTTGGTTTGTGCCTTAAAG

GACATGGAGGAGGAGATCTTGGAAGGCATGAAAGCAAAAAGCCTGGACGACTATTTGAATGGCCCCTTCACTGTGGTAGT
AAAAGAGTCCTGTGATGGAATGGGAGATGTCAGTGAGAAGCATGGAAGTGGGCCTGCTGTCCCAGAGAAAGCTGTTCGCT
TTTCTTTCACTGTCATGAACATTTCTATAGCACATGGGAGTGAAAGCAAGAGAATCTTTGAGGAAGTAAAGCCCAATTCA
GAGTTGTGCTGTAAGCCCTTGTGCCTTATGCTGGCTGATGAATCAGATCATGAAACTCTGACAGCAATCCTGAGCCCCCT
CATAGCAGAAAGAGAAGCTATGAAAAACAGTGAACTGCTGCTTGAAATGGGAGGCATCCTGAGAACATTTAGATTTGTCT
TTAGGGGTACAGGATATGATGAGAAACTCGTGCGGGAGGTGGAAGGGCTGGAAGCTTCAGGTTCCACTTACATTTGTACC
CTGTGTGATGCAACCCGCTTGGAGGCATCCCAGAATCTGGTCTTCCACTCCATAACCAGGAGCCATGCTCAAAATCTGGA
GCGATATGAAATGTGGAGGTCCAACCCATATCATGAGTCTGTTGATGAGCTCCGTGACAGAGTGAAGGGTGTCTCAGCCA
AACCTTTTATTGAGACTGTTCCCTCCATAGATGCACTGCACTGTGACATTGGCAATGCAACAGAATTCTACAGGATTTTC
CAGATGGAAATTGGTGAACTTTACAAGAATCCTGATGCATCTAAAGAGGAGAGGAAAAGGTGGCAGTTGACTCTTGACAA
ACACCTCAGGAAGAAGATGAACTTGAAGCCTATGTTGAAGATGAGTGGAAATTTTGCTAGAAAGCTCATGTCCAAAGAGA
CTGTAGAGGCAGTATGTGAATTAATAAAGTGTGAGGAAAGGCATGAAGCCCTAAAGGAACTAATGGACCTTTATCTGAAT
ATGAAGCCAGTGTGGCGATCCTCATGCCCTGCCAAAGAGTGCCCAGAACTGCTGTGCCAGTATAGCTACAATTCACAGCG
TTTTGCAGAGCTCTTATCTACAAAATTCAAATACAGATATGATGGCAAGATTACAAATTACTTCCACAAAACACTTGCTC
ATGTTCCAGAAATCATTGAAAGAGATGGGTCCATTGGGGCCTGGGCAAGTGAAGGAAATGAGTCTGGAAACAAACTGTTT
AGGAGGTTCCGAAAAATGAATGCCAGGCAGTCCAAAGTCTATGAGATGGAGGATGTCTTG
Ailuroedus
??
??
??
??
??
??
??
??
??
??
??
??
??ATAAGATGCC
CTGTAAAGGAATGTGATGAAGAGATCCTGCATGGAAAATACAGCAAACACCTCTCCAGCCAT---
GAGATGAAAGATAGAGAGCTCTATAGCTACATAAATAAAGGTGGCCGACCAAGGCAGCACCTCCTGACTTTGACAAGGAG
AGCTCAGAAACATCGTCTGAGGGAACTGAAACGTCAAGTCAAGGCTTTTGCTGAGAAAGAAGAGGGTGGTGATATAAAGG
CTGTATGCATGACTTTGTTCCTGCTAGCTTTGAGAGCAAAAAATGAACACAAACAAGCAGATGAATTGGAGGCTATAATG
AAAGGGAGGGGATCTGGACTTCATCCTGCCGTCTGTCTGGCAATCCGAGTCAACACATTTCTCAGCTGTAGTCAGTATCA
TAAAATGTATAGAACAGTAAAAGCTGTCACTGGGAGGCAGATCTTCCAGCCTTTGCATGCTCTTCGCACTGCTGAGAAAG
CCCTCCTGCCAGGTTACCACCCATTCGAGTGGAAACCTCCCTTGAAAAATGTATCCACTAACACAGAAGTGGGAATTATA
GATGGACTATCAGGATTGCCACTCTCAATTGATGACTACCCAGTAGACACAATTGCAAAGAGATTTCGATATGATGCAGC
CTTGGTTTGTGCCTTAAAGGACATGGAGGAGGAGATCTTGGAAGGCTTGAAAGCAAAAAATCTGGATGACTATTTGAATG
GCCCCTTCACGGTGGTAGTAAAAGAGTCCTGTGATGGAATGGGGGACGTCAGTGAAAAGCACGGAAGTGGGCCTGCTGTC
CCAGAGAAGGCTGTTCGCTTTTCTTTCACAGTCATGAACATTTCTATAGCACATGGGAATGAAAGCAAGAGGATTTTTGA
GGAACTAAAGCCCAATTCAGAGTTGTGCTGCAAGCCCTTGTGCCTTATGCTGGCT?????????????????????????
??
??
??
??
??
??
??
??
??
??
??
???
Zosterops   TAAATTGCCATCATTTGAAAAAACACCCT-------------------------
----
CTGATGACAGCCAGCACATAAACAAAGACCAGGCAGAAGAGGCTGTTTCTTCAAACAAAGAATTCATCCTGCATAAAGAT
GAAACAGTGCCAAGAGGAGAAAAGATGGAGTTAATGGGCAATAGGCAGGGACTTGAGGAAGATGCCCATGCCATGAAAAC
ACAAGACAATAGAGCTCATCAGAACAATCTGGAGCAACTCTGCCGCATCTGTGGAGTTTCATTTAAAACTGATTGTTACA
AGAGAACTTACCCAGTGCATGGGCCAGTGGATGATGAAACTCTGTTGCTTCTGAGAAAGAAAGAAAAAACAGCAACCTCT

TGGCCAGATCTTATTGCTAAGGTTTTCAAGATAGATGTGCGAGGGGATGTCGATACTATCCATCCCACTCGATTTTGTCA
CAACTGTTGGAGTATTATACACAGAAAATTCAGTAATACTCCATGTGAAGTATATTTTCCTAGGAACAGCACAATGGAGT
GGCAACCCCATTCCCCAAACTGTGATGTGTGCCATACTACCAGACGAGGAGTCAAGAGAAAGAAACAGCCTGCAAGTGTA
CAATCTGGCAAACGTGTCAAAACCACTGGGCAACGTGCTCAGCTAAACAGAGGTGTAAAGAAC------------
CAAGCACAGATAAACAACAAAAATTTAATTAAAGAGATTATCAATTGCAAGGATATACATCTCAGCATCAAGCTGCTTGC
AATTGATTACCCAGTAGATTTCATTAAATCCATTTCTTGCCAGATTTGTGATCATATTTTGGCAGATCCAGTGGAAACAA
CATGCAGACACTTGTTTTGCAGAACTTGCATCCTTAAATGTATCAGGGTTATGGGCAGCTATTGCCCCTCCTGCTGGTAT
CCTTGCTTTCCTACAGATCTGGTTACCCCAGTGAAATCCTTCCTGAACATCCTTGATAACTTAAGTATAAGATGCCCCGT
AAAGGAATGTGATGAAGAGATCTTGCATGGAAAATACAGCCAACACCTCTCTCGTCACA?GGAGATGAAAGATGGGGAGC
TCCATAGCTACATAAATAAAGGTGGCCGACCAAGGCAGCACCTCCTCT?TTTGACAAGGAGAGCTCAGAAACATCGT?TG
AGG?AA?TGAAACGTCAAGTCAAGGCTTTTGCTGAGAAAGAAGAGGGTGGTGATATAAAGGCTGTATGCATGACTTTGTT
CCTGCTAGCTTTGAGAGCAAAAAATGAACACAAACAAGCAG?TGAACTGGAGGCTATAATGCAAGGGAGAGGATCTGGAC
TTCATCCTGCTGTCTGTCTGGCCATC?GAATCAACACGTTTCTCAG?TGCAGTCAGTACCATAAAATGTATAGAACAGTA
AAAGCTGTCTCTGGGAGGCAGCTCTTCTAGCCTCTGCATGCTCTTCGCACTGCTGAGAAAGCCCTCCTACCAGGTTATCA
CCCATTTGAGTGGAAACCTCCCCTGAAAAATGTATCCACTAACACAGAAGTGGGAATTATAGATGGACTATCAGGACTGC
CACTCTCAATTGATGATTACCCAGTAGACACAATTGCAAAGAGATTCCGATATGATGCAGCCTTAGTTTGTGCCTTAAAA
GACATGGAGGAGGAGATCTTGGAAGGCATGAAAGCAAAAAACCTGGACGACTATTTGAGTGGCCCCTTCACTGTGGTAGT
AAAAGAGTCCTGTGATGGAATGGGAGATGTCAGTGAAAAGCATGGAAGTGGGCCTGCTGTCCCAGAGAAGGCTGTTCGCT
TTTCCTTCACAGTCATGAACATTTCTATATCACATGAGAATGAAAGCAAGAGGATCTTTGAGGAAGTAAAGCCCAATTCA
GAGTTGTGCTGTAAGCCCTTATGCCTAATGCTGGCAGATGAATCAGATCATGAAACGCTGACAGCAATCCTGAGCCCCCT
CATAGCAGAAAGAGAGGCTATGAAAAACAGTGAACTGCTGCTTGAAGTGGGAGGCATCCTGAGAACATTTAGATTTGTTT
TTAGGGGTACAGGATATGATGAGAAACTCGTGCGGGAAGTGGAAGGGCTGGAGGCCTCAGGTTCCACTTACATTTGTACC
TTGTGTGATGCAACCCGCTCGGAGGCATCCCAGAATCTGGTCTTCCATTCCATAACCAGGAGCCATGCTGAAAATCTGGA
GCGATATGAAATATGGAGGTCCAACCCATTTCATGAGTCTGTTGATGAGCTCCGTGACAGAGTGAAGGGTGTTTCAGCCA
AACCTTTTATTGAGACTGTCCCCTCCATAGATGCATTGCACTGTGACATTGGCAATGCAACAGAATTCTACAGGATTTTC
CAGATGGAGATTGGTGAACTTTACAAGAATCCTGATGTATCTAAAGAGGAGAAGAAGAGGTGGCAGTTGGCTCTTGACAA
ACACCTCAGGAAGAAGATGAACTTGAAGCCTATGTTGAAAATGAGTGGAAATTTTGCTAGAAAGCTTATGTCCAAAGAGA
CTGTAGAAGCAGTATGTGAATTAATAAAGTGTGAGGAAAGGCATGAAGCCCTGAAGGAACTAATGGACCTTTATCTGAAA
ATGAAGCCAGTGTGGCGATCCTCATGCCCTGCCAAGGAGTGCCCAGAACTGCTGTGCCAGTATAGCTATAATTCACAGCG
TTTTGCAGAACTCTTATCTACAAAGTTCAAGTACAGATATGAGGGCAAGATTACAAATTATTTCCATAAAACACTTGCTC
ATGTTCCTGAAATCATTGAAAGAGATGGGTCCATTGGGGCCTGGGCAAGTGAAGGAAATGAGTCTGGAAACAAACTGTTT
AGGAGGTTCCGAAAAATGAATGCCAGGCAGTCCAAAGTCTATGAGATGGAGGATGTCTTG
Onychorhynchus   TAAAGTACGATCATTTGAAAAAACGCCTT-------------------------
----
CTGATGACAGGCAGCACAAAAACAAAGATCAGGCAGAAGGGATTGCTTCTTCAAACAAAGAAATCATACTGCATAAAGAT
AAAGCAGTGTCAAGAGAAGAAAAGATGGAGTTAATGGGCAATAGGCAGGCACTTGAGAAAGAGGCCCATGACCTGAAAAC
AAGAGACAATAGAGCTCATCAGAACAATCTGAAGCAACTCTGTCGCATCTGTGGAGTTTCATTTAAAACTGATTGCTACA
AGAGAACTCATCCAGTGCACGGGCCAGTGGATGATGAAACTCTGTGTCTTCTGAGAAAGAAAGAAAAAAAAGCAACCTCT
TGGCCAGATCTTATCTCTAAGGTTTTCAAGATTGATGTGCGAGGGGATGTTGATACTATCCATCCCACTCGGTTTTGTCA
CAACTGTTGGAGTATTATCCATAGAAAATACAGTAATACTCTATGTGAAGTATATTTTCCTAGGAACAGCACCATGGAGT
GGCAACCTCACTCCCCAAACTGTGATGTATGCCATAGTACCAGTCGAGGGATCAAGAGAAAAAGCCAGCCACCAAGTGTA
CAACAAGGCAAACGTGTGAAGACCACTGTGGAACGTGCTCAACTAAACAGAGGTGTAAAGAAC------------
CAGGCACAGATAAACAACAAAAATTTAATGAAAGAGATTGTCAATTGTAAGAATATACATCTCAGCACCAAGCTGCTTGC
AGTTGATTACCCAGTAGATTTCATTACATCAATTTCTTGCCAGATTTGTGAGCATATTTTGGCAGATCCAGTGGAAACAA
CATGCAGACACTTGTTTTGCAGAACTTGCATCCTTAAATGTATCAAGGTTATGGGCAGCTATTGCCCCTCCTGCTGGTAT
CCTTGCTTCCCTACTGATCTGGTAACCCCAGTGAAATCCTTCCTGAACATCCTTGATAGCCTGGCTATAAGATGTCCTGT
CAAGGAATGTGATGAAGAGATTTTGCATGGAAAATACAGCCAACACCTCTCCAACCACAACGAGATGAAAGATAGAGAGC
TCTATAGCTACATAAATAAAGGTGGCCGACCAAGGCAGCATCTCCTATCTTTGACAAGGAGAGCTCAGAAACATCGTCTG
AGGGAGCTGAAACGTCAAGTCAAGGCTTTTKCTGAGAAAGAAGAGGGTGGTGATATAAAGGCTGTATGCATGACTTTGTT
CCTTCTAGCTTTAAGAGCGAAAAACGAACACAGACAAGCAGATGAATTGGAGGCTATAATGCAAGGGAGGGGGTCTGGAC
TTCATCCTGCTGTCTGTCTGGCAATCCGAGTCAACACGTTTCTCAGCTGTAGCCAGTACCATAAAATGTATAGAACTGTA
AAAGCTGTCACCGGGAGGCAGATCTTCCAGCCCTTGCATGCTCTTCGCACTGCTGAGAAAGCCCTCCTACCAGGTTATCA
CCCATTTGAGTGGAAACCTCCCTTGAAAAATGTATCCACTAATACAGAAGTAGGAATTATAGATGGGCTATCAGGATTGC
CGCTCTCCGTTGATGACTACCCAGTAGACACAATTGCAAAGAGATTCCGATATGATGCAGCCTTGGTTTGTGCCTTAAAG
GACATGGAGGAGGAGATCTTAGAAGGCATGAAAGCAAAAAATCTGGATGACTATTTGAATGGTCCCTTCACTGTGGTAAT
AAAAGAGTCCTGTGATGGAATGGGAGATGTCAGCGAGAAGCATGGAAGTGGGCCTGCTGTCCCAGAGAAGGCTGTTCGCT
TTTCTTTCACAGTTATGAACATTGCTATAGCACATGGGAATGAAAGCAAGAGGATCTTTGAGGAAGTAAAGCCAAATTCA
GAGTTGTGTTGCAAGCCCTTGTGCCTTATGCTGGCTGACGAATCGGATCATGAAACTCTAACAGCAATCCTGAGCCCCCT
CATAGCAGAAAGAGAGGCTATGAAAAACAGTGAACTGCTGCTTGAAATGGGAGGCATCCTGAGAACATTCAGATTTGTCT
TTAGGGGTACAGGATATGATGAGAAACTTGTGCGGGAAGTGGAAGGGCTGGAAGCCTCAGGTTCCACTTACATTTGTACC

CTGTGTGATGCAACCCGCTTGGAGGCATCTCAGAATTTGGTCTTCCACTCCATAACCAGGAGCCAYGCTGAAAATCTGGA
GCGATATGAAATATGGAGGTCCAACCCATATCATGAATCTGTTGATGAGCTCCGTGACAGAGTGAAGGGTGTTTCAGCCA
AACCTTTTATTGAGACTGTTCCCTCCATAGATGCATTGCACTGTGACATTGGCAATGCAACAGAATTCTACAGGATTTTC
CAGATGGAGGTTGGTGAGCTTTACAAGAATCCTGATGTGTCTAAAGAGGAGAGAAAGAGGTGGCAGTTGACTCTTGACAA
ACACCTCAGGAAGAAGATGAACTTGAAGCCTATGATGAAGATGAGTGGAAATTTTGCTAGAAAGCTCATGTCCAAAGAGG
CAGTAGAGGCAGTATGTGAATTAATAAAGTGTGAGGAAAGGCATGAAGCCCTAAAAGAACTAATGGACCTTTATCTGAAG
ATGAAGCCAGTGTGGCGATCCTCATGCCCTGCTAAGGAGTGCCCAGAACTGCTGTGCCAGTATAGCTACAATTCACAGCG
TTTTGCTGAGCTCTTATCTACTAAGTTCAAATACAGATATGAAGGCAAGATTACAAATTATTTCCATAAAACACTTGCTC
ATGTTCCTGAAATCATTGAAAGAGATGGGTCTATAGGGGCCTGGGCAAGTGAAGGAAATGAGTCTGGAAACAAACTGTTC
AGGAGGTTCCGAAAAATGAATGCCAGGCAGTCCAAATTCTATGAGATGGAGGATGTCTTG
Nestor_notabilis
??
??GCTTCTTCAAACAAAGAAATCATACTG
CATAAAGATGAAGCAGTGCCAAGAGGAGA------------
AATGGGCAATAGGCAGGCACTTGAGAATGATGACAAGAACATGAAAACACAAGACAATAAAGCTCATCAGAACAATCTGA
AGCAACTTTGCCGCATCTGTGGAGTTTCATTTAAAACTGATCGTTACAAGAGAACTCACCCAGTGCATGGGCCGGTGGAT
GATGAAACTCTGCGGCTTCTGAGAAAGAGAGAGAAAAAAGCAACCTCTTGGCCGGATCTTATCTCTAAGGTTTTTAAGAT
TGATGTGCGAGGGGATGTTGATACTATCCATCCCACTCGATTTTGTCACAATTGCTGGAGCATTATCCATAGAAAATTCA
GTAATACTCCATATAAAGTATGTTTTCCTAGGAACAGCACGAAGGAGTGGCAACGCCACTCCCCAAACTGTGATGTGTGC
TGCACAACCCGTCAGGGAGTCAAGAGAAAAAGCCAGTCATGCAGCATACAACATGGCAAACGTGCAAAGACTGTTGCAGA
ATGTGCTCAATTAAACAGAAGCGTAAAGAAC------------CAAGCACAGATAAAC---
AAAAATTTAATGAAAGAGATTGTTAGTTGCAAGAATATACATCTCAGCACCAAGCTTCTTGCAGTTGATTACCCTGTAGA
TTTCATTAAATCTATCTCTTGCCAGATCTGTGAGCATATTTTGGCAGATCCAGTGGAAACATCATGTAGGCACTTGTTTT
GCAGAACTTGCATCCTTCAATGTATCAAGGTTACGGGCAGCTATTGCCCCTCCTGCTGGTATCCTTGCTTCCCTACTGAT
CTGGTAACCCCAGTGAAATCCTTCCTTAATATCCTTGATAATCTGGGTATAAGATGCCCTGTAAAGGAATGTGATGAAGA
GATCTTGCATGAAAAATATGGTCAACACCTCTCCAGCCACAAGGAAATGAAAGATAGAGAGCTCTACAGCCACATAAATA
AAGGTGGCCGACCAAGGCAGCATCTCCTGTCTTTGACCAGGAGAGCTCAGAAACATCGTCTGAGAGAACTGAAGCGTCAA
GTCAAAGCTTTTGCTGAGAAAGAAGAGGGAGGTGATATAAAGTCTGTATGCATGACTTTGTTCCTGCTAGCTTTAAGAGC
AAAAAACGAACACCGACAAGCAGATGAGTTGGAGGCTATAATGCAAGGGAGGGGATCTGGACTTCACCCTGCGGTCTGCT
TGGCAATCCGAGTCAACACATTTCTCAGCTGTAGCCAGTATCATAAAATGTATAGAACAGTAAAAGCTGTTACTGGTAGG
CAGATTTTTCAGCCTTTGCATGCTCTTCGCACTGCTGAGAAAGCCCTCCTGCCAGGTTATCATCCATTTGAGTGGAAACC
TCCCTTGAAAAATGTATCCACCAACACGGAAGTGGGAATTATAGATGGACTATCAGGATTGCCACTCTCTATTGATGACT
ACCCAGTAGACACAATTGCAAAGAGATTTCGGTATGATGCAGCCTTGGTTTGTGCCTTAAAGGACATGGAGGAGGAGATC
TTGGAAGGCATTAAAGCAAAAAACCTGGATGATTATTTGAACGGTCCCTTCACCGTGGTAGTAAAAGAGTCCTGTGATGG
AATGGGAGATGTCAGTGAGAAGCATGGAAGTGGACCTGCTGTCCCAGAGAAGGCTGTTCGCTTTTCTTTCACAGTCATGA
ACATTGCT?????????????????????????????????????GAAGTAAAGCCCAATTCAGAGCTGTGTTGCAAGCCC
TTGTGCCTTATGCTAGCTGATGAATCGGATCATGAAACTCTGACAGCAATCCTGAGCCCCCTCATAGCAGAAAGAGAGGC
TATGAAAAACAGTGAACTGCTTCTTGAAATGGGAGGCATCCTAAGAACATTCAAATTCGTCTTTAGGGGTACAGGATATG
ACGAGAAACTCGTGCGGGAAGTGGAAGGGCTGGAGGCCTCAGGTTCCACTTACATTTGTACCCTGTGTGATGCAACCCGC
CTGGAGGCATCCCAGAATGTGGTCTTCCACTCCATAACCAGGAGCCATGCTGAAAATCTGGAGCGATATGAAATATGGAG
GTCCAACCCATATCATGAATCTGTTGATAAGCTCCGTGACAGAGTGAAGGGTGTTTCAGCCAAACCTTTTATTGAGACCG
TTCCCTCCATAGATGCGTTGCACTGCGACATTGGAAATGCAGCAGAATTCTATAGGATTTTCCAGATGGAGATCTGTGAA
GTTTATAAGAATCCTGATGTGTCTAAAGAGGAAAGGAAGAGGTGGCAGTTGGCTCTTGACAAGCACCTCAGGAAGAAGAT
GAACTTGAAGCCTATGATGAGGATGAGCGGAAATTTTGCTAGAAAGCTCATGTCCAAAGAGACAGTAGAGGCAGTATGTG
AATTAATAAAGTGTGAAGAAAGGCATGAGGCCCTAAAAGAACTAATGGACCTTTATCTGAAGATGAAGCCAGTGTGGCGA
TCCTCATGCCCTGCCAAGGAATGTCCAGAATTGCTGTGCCAGTATAGCTATAATTCACAGCGTTTTGCCGAGCTCCTATC
TACAAAGTTCAAGTACAGATATGAAGGCAAGATTACAAATTATTTCCACAAAACCCTTGCTCATGTTCCTGAAATCATTG
AAAGAGATGGGTCCATTGGGGCCTGGGCAAGTGAAGGAAAT??
???
Leiothrix_argentauris CAAATTGCGATCATTTGAAAAAACACCCT-------------------------
----
CTGAAGACAGCCAGCACATAAACAAAGACCAGACAGAAGAGGCTGTTTCTTCAAACAAAGAATTCATCCTGCGTAAAGAT
GAAGCAGTGCCAAGAGGAGAAGAGATGGAGTTAACGGGCAATAGGCAGGGACTTGAGGAAGATGCCCATGCCGTGAAAAC
ACAAGACAACAGAGCTCATCAGAACAATCTGAACCAACTCTGCCGCATCTGTGGAGTTTCATTTAAAACTGATGGTTACA
AGAGAACTTACCCAGTGCATGGGCCAGTGGATGAGGAAACTCTGTTGCTTCTGAGAAAGAAAGAAAAAACAGCAACCTCT
TGGCCAGATCTGATTGCTAAGGTTTTCAAGATTGATGTGCGAGGGGATGTTGATACTATCCATCCCACTCGATTTTGTCA
CAACTGTTGGAGTATTATACACAGAAAATTCAGTAATACTCCATGTGAAGTATATTTTCCTAGGAACAGCACAATGGAGT
GGCAACCCCATTCCCCAAACTGTGATGTGTGCCATACTACCAGACGAGGAGTCAAGAGAAAGAAACAGCCCCCAAATGTA
CAGTCTGGCAAACGTGTCAAAACCACTGGGCAACGTGCTGGGCTAAAYAGAGGTGTAAAGAAG------------
CAAGCACAGATAAACAACAAAAATTTAATTAAAGAGATTATCAATTGCAAGGATATACATCTCAGCACCAAGCTGCTTGC

AATTGATTACCCAGTAGATTTCATTAAATCCATTTCTTGCCAGATTTGTGATCATATTTTGGCAGATCCAGTGGAAACAA
CATGCAGACACCTGTTTTGCAGAACTTGCATCCTTAAATGTATCAGGGTTATGGGCAGCTATTGCCCCACCTGCTGGTAT
CCTTGCTTTCCTACTGATCTGGTTACCCCAGTGAAATCCTTCCTGAACATCCTTGATAACCTAAGTATAAGATGCCCTGT
AAAGGAATGTGATGAAGAGATCTTGCATGGAAAATATGGCCAACATCTCTCTGGTCACAAGGAGATGAAAGATGGAGAGG
TCTATAGCTACATAAATAAAGGTGGCCGACCGAGGCTGCACCTCCTGTCTTTGACCAGGAGAGCTCAAAAACATCGTCTG
AGGGAACTGAAACGTCAAGTCAAGGCTTTTGCTGAGAAAGAAGAGGGTGGTGATATAAAGGCTGTATGCATGACTTTGTT
CCTGCTAGCTTTGAGAGCCAAAAATGAACACAAACAAGCAGATGAACTGGAGGCTATAATGCAAGGGAGGGGATCTGGAC
TTCATCCTGCTGTCTGTCTGGCCATCCGAATCAACACGTTTCTCAGCTGTAGTCAGTACCATAAAATGTATAGAACAGTA
AAAGCTGTCTCTGGGAGGCAGATCTTCCAGCCTTTGCATGCCCTTCGCACTGCTGAGAAAGCCCTCCTACCAGGTTATCA
CCCATTTGAGTGGAAACCTCCCCTGAAAAATGTATCCACTAACACAGAAGTGGGAATTATAGATGGACTATCAGGACTGC
CACTCTCAATTGATGATTACCCAGTAGACACAATTGCAAAGAGATTCCGATATGATGCAGCCTTAGTTTGTGCCTTAAAA
GACATGGAGGAGGAGATCTTGGAAGGCATGAAAGCAAAAAACCTGGACGACTATTTGAGTGGCCCCTTCACTGTGGTAGT
AAAAGAGTCCTGTGAYGGAATGGGAGATGTCAGTGAGAAGCATGGAAGTGGGCCTGCTGTCCCAGAGAAGGCTGTTCGCT
TTTCCTTCACAGTCATGAACATTTCTATAGCACATGRGAATGAAAGCAAGAGGATCTTTGAGGAAGTAAAGCCCAATTCA
GAGTTGTGCTGTAAGCCCTTATGCCTTATGCTGGCTGATGAATCAGATCATGAAACTCTGACAGCAATCCTGAGCCCCCT
CATAGCAGAAAGAGAGGCTATGAAGAACAGTGAACTGTTGCTTGAAATGGGAGGCATCCTGAGAACATTTAGATTTGTCT
TTAGGGGTACAGGGTATGATGAGAAACTTGTGCGRGAAGTAGAAGGGCTGGAGGCCTCAGGTTCCACTTACATTTGTACC
CTGTGTGATGCAACCCGCTTGGAGGCATCCCAGAATCTGGTCTTCCACTCCATAACCAGGAGCCATGCTGAAAATCTAGA
GCGATATGAAATATGGAGGTCTAACCCATATCACGAGTCTGTTGATGAGCTCCGTGACAGAGTGAAGGGTGTTTCAGCCA
AACCTTTTATTGAGACTGTTCCCTCCATAGATGCATTGCACTGCGACATTGGCAATGCAACAGAATTCTACAGGATTTTC
CAGATGGAGATTGGGGAACTTTATAAGAATCCTGATGTGTCTAAAGAGGAGAGGAAGAGGTGGCAGTTGGCTCTTGACAA
ACACCTCAGGAAGAAGATGAACTTGAAGCCTATGTTGAAGATGAGTGGAAATTTTGCTAGGAAGCTCATGTCCAAAGAGA
CTGTAGAGGCAGTATGTGAATTAATAAAGTGTGAGGAAAGGCATGAAGCCCTAAAGGAACTAATGGACCTTTATCTGAAG
ATGAAGCCAGTGTGGCGATCCTCATGCCCTGCCAAGGAGTGTCCAGAACTGCTGTGCCAGTATAGCTACAATTCACAGCG
TTTTGCAGAACTCTTATCCACAAAGTTCAAGTACAGATATGAGGGCAAGATTACAAATTATTTCCACAAAACACTTGCTC
ATGTTCCTGAAATCATTGAAAGAGATGGGTCCATTGGGGCCTGGGCAAGCGAAGGAAATGAGTCTGGAAACAAACTGTTT
AGGAGGTTTCGAAAAATGAATGCCAGGCAGTCCAAAGTCTATGAGATGGAGGATGTCTTG
Troglodytes TAAATTGCGATCATTTGAAAAAACACCCT------------------------
----
CTGATGACAGCCAGCACATAAACAAAGATCAGGCAGAAGAGGCTGTTTCTTCACACAAGGAATTCATCCTGC---
AAGATGAAGCAGTGCCAAGAGGAGAAAAGRTGRAGTTAACAGGCAACAGGCAGGGGCTAGAGGAAGATGCCCATGCCATG
AAAACACAAGACAATAGAGCTCATCAGGACAATCTGAAGCAACTCTGTCGCATCTGTGGAGTTTCATTTAAAACTGATTG
CCACAAGAGAACTTACCCAGTGCATGGGCCAGTGGATGATGAAACTCTGTGGCTTCTGAGAAAGAAAGAAAAAACAGCAA
CYTCTTGGCCAGATCTTATTGCTAAGGTTTTCAAGATTGATGTGCGAGGGGATGTCGATACTATCCATCCCACTCAATTT
TGTCACAATTGTTGGAGTATTATACATAGCAAATTCAGTAATACTCTATGTGAAGTATATTTTCCCAGGAACAGCACAAT
GGAGTGGCAACCCCATTCCCCAAACTGTAATGTGTGCCATACTACTAGACGAGGAGTCAAGAGAAAAAGCCAGCCTGCAA
RTGTTCAACGTGGCAAACGTGTCAAAACCACTGTG------------TTAAACAGAGGTGTAAAGAAC------------
CAAGCACAGATAAACAAYAAAMATTTAATGAAAGAGATTGTCAGTTGCAAGGATGTACATCTCAGCACCAAGCTGCTTGC
AGTTGATTACCCACTAGATTTCATTAAATCCATTTCTTGCCAGATTTGTGATCATATTTTGGCAGATCCAGTGGAAACAA
CATGCAGACACTTGTTTTGCAGAACTTGCATCCTTAAATGTATCAGGGTTATGGGCAGCTATTGCCCCTCCTGCTGGTAT
CCTTGCTTTCCTACGGATCTGGTAACCCCAGTGAAATCCTTCCTGAACATCCTTGATAACCTGAGTATAAGATGCCCTGT
AAAGGAATGCGATGAAGAGATTTTGCATGGAAAATATGGTCAACACCTCTCTGGTCACAAGGAGASAAAAGATGGAGAGC
TCTATAGCTACATAAACAAAGGTGGCCGACCGAGGCTGCACCTCTTGTCTTTGACAAGGAGAGCTCAGAAACATCGTCTG
AGGGAACTGAAACGTCAAGTCAARGCTTTTGCTGAGAAAGAAGAGGGYGGTGATATAAAGGCTGTATGCATGACTTTGTT
CCTGCTAGCTTTGAGAGCAAAAAATGAACACAAACAAGCAGATGAACTGGAAGCTATAATGCAAGGGAGGGGATCTGGAC
TTCATCCGGCTGTCTGTCTGGCCATCCGAATCAACACGTTTCTCAGCTGTAGTCAGTATCATAAAATGTAYAGGACAGTA
AAAGCTGTCACGGGGAGGCAGATCTTCCAGCCTTTGCATGCTCTTCGCACTGCTGAGAAAGCCCTCCTACCRGGTTATCA
CCCGTTTGAATGGAAACCTCCCCTGAAAAATGTATCCACTAACACYGAAGTGGGAATTATAGATGGACTATCAGGACTGC
CGCACTCAATCGATGATTACCCAGTAGAAACAATTGCAAAGAGATTCCGATATGATGCAGCCTTGGTTTGTGCCTTAAAG
GACATGGAGGAGGAGATCTTGGAAGGCATGAAAGCAAAAAAYCTGGATGACTATTTGAATGGTCCCTTCACTGTGGTAGT
AAAAGAGTCCTGTGATGGAATGGGAGATGTCAGTGAGAAGCATGGAAGTGGGCCTGCTGTCCCAGAGAAGGCCGTTCGCT
TTTCTTTCACAGTCATGAACATTTCTATAGCACATGGGAATGAAAGCACGAGGATCTTTGAGGAGGTAAAGCCCAATTCA
GAGTTGTGCTGTAAACCCTTGTGCCTTATGCTGGCTGATGAATCAGATCATGAAACTCTGACAGCAATCCTGAGCCCYCT
CATAGCAGAAAGAGAGGCTATGAAAAGCAGTGAACTGCTGCTTGAAATGGGAGGCATCCTGAGAACATTTAGATTCGTCT
TTAGGGGCACAGGATATGATGAGAAACTTCTGCGGGAAGTGGAAGGGCTGGAGGCCGCAGGTTCCACCTACATTTGTACC
TTGTGTGATGCAACCCGCTTGGAGGCATCCCAGAATCTGGTCTTCCACTCCATAACCAGGAGCCATGCTGAAAATCTGGA
GCGATACGAAATATGGAGGTCCAACCCATACCACGAGTCTGTTGATGAGCTCCGTGACAGAGTGAAGGGTGTTTCAGCCA
AACCTTTTATTGAGACTGTTCCCTCCATAGATGCATTGCACTGTGACATTGGCAATGCAACAGAATTTTACAGGATTTTC
CAGATGGAGATTGGTGAACTTTACAAGAATCCTGATGTGTCTAAAGAGGAGAGGAAGAGGTGGCAGTTGACTCTTGACAA
ACACCTCAGGAAGAAGATGAACTTGAAGCCTATGTTGAAGATGAGTGGAAATTTTGCTAGAAAGCTCATGTCCAAAGAGA

CTGTAGAGGCAGTATGTGAATTAATAAAGTGTGAGGAAAGGCATGAAGCCCTAAAAGAACTAATGGACCTTTATCTGAAG
ATGAAGCCAGTGTGGCGATCCTCATGCCCTGCCAAGGAGTGCCCAGAACTGCTGTGCCAGTATAGCTACAATTCACAGCG
TTTTGCAGAGCTCTTATCTACAAAGTTCAAGTACAGATATGAGGGCAAGATTACAAATTATTTCCATAAAACACTTGCTC
ATGTCCCTGAAATCATTGAAAGAGATGGGTCCATTGGGGCCTGGGCAAGCGAAGGAAATGAGTCTGGAAACAAACTGTTT
AGGAGGTTCCGAAAAATGAATGCCAGGCAGTCCAAAGTCTATGAGATGGAGGATGTCTTG
Luscinia_svecica
??
??
??
??
??
??
??
??
??
??
??
??
??
??
??
??
??
??
??
??
??
??
??
??
??
??
??
??
??
??
??
??
??
??
??
??
??
??
Psittacus_erithacus   TAAAGTGAGATCATTTGAAAAACTGCCTT------------------------
-----------------------------------CTGATGACAA---------AAACAAGGGTAAGGCAGAA---
ATTGCTTCTTCAAACAAAGAAATCATACTGTGTAAAGATGAAGCAGTGCCGAGAGAAGAAAACATGGACTTAATGGGCAA
TGGGCAGGCACTTGAGAATGATGGCAAAAAAATGAAAACACAAGACAATAAAGCTCATCAGAACAATCTGAAGCAACTTT
GCCGCATCTGTGGAGTTTCATTTAAAACTGATTGTTATAAGAAAACTCACCCAGTGCATGGGCCAGTGGATGATGAAACT
CTGTGGCTTTTGAGGAAGAGAGAGAAAAAAGCAACCTCTTGGCCAGATCTTATCTCTAAGGTTTTTAAGATTGATGTGCG
AGGGGATGTTGATACTATCCATCCCACTCGATTTTGTCATGATTGTTGGAGCATTATCCATAGAAAATTCAGTAATACTC
CATATAAAGTATGTTTTTCTAGGAATAGCACGAAGGAGTGGCAACGCCACTCTCCAAACTGTGATGTGTGCCACACTACC
CGTCAGGGAGTCAAGAGAAAAAGCCAGCCACCCAGTGTACAATATGGCAAACCTGCAAAGACCGTTGCAGAATGTGCTCA
AATAAACAGAAATGTAAAGAAC------------CAAGCACAGATAAAC---
AAAAATTTAATGAAAGAGATTGTCAGTTGCAAGAATATACATCTCAGTACCAAGCTCCTTGCAGTTAATTACCCCATAGA
TTTCATTAAATCTATCTCTTGTCAGATCTGTGAGCATATTTTGGCAGATCCAGTGGAAACATCATGTAGGCACTTGTTTT
GCAGAACCTGCATCCTTAAATGTTTCAAGGTTATGGGCAGCTATTGTCCCTCCTGCTGGTATCCTTGCTTCCCTACTGAT
CTGGTCACCCCAGTGAAATCCTTCCTGAACATCCTTGATAATCTGGGTATAAGATGCACTGTAAAGGAATGTGWTGAAGA
GATCTTGCATGAAAAATATGGTCAACACCTCTCCAGCCACAAGGAGATGAAAGATAGAGAGCTGTACAGCCACATAAATA
AAGGTGGCCGACCAAGGCAGCATCTCCTGTCTTTGACCAGGAGAGCTCAGAAACATCGTCTGAGAGAACTGAAGCGCCAA
GTCAAGGCTTTTGCTGAAAAAGAAGAGGGAGGAGATATAAAATCTGTATGCATGACTTTGTTTCTGCTAGCTTTAAGAGC

AAAAAATGAACACCGACAAGCAGATGAATTGGAGGCTATAATGCAAGGGAGGGGATATGGACTTCACCCTGCGGTCTGCT
TGGCAATCAGAGTCAACACGTTCCTCAGCTGTAGCCAGTATCATAAAATGTATAGAACAGTAAAGGCTGTCACTGGAAGG
CAGATCTTTCAGCCTTTGCATGCTCTTCGCACTGCTGAGAAAGCCCTCCTACCAGGTTATCATCCATTTGAGTGGAAACC
TCCCTTAAAAAATGTATCCACTAACACAGAAGTGGGAATTATAGACGGGTTATCAGGATTGCCACTATCAATTGATGACT
ACCCAGTAAACACAATTACAAAGAGGTTTCGGTATGATGCAGCCTTGGTTTGTGCCTTAAAGGACATGGAGGAGGAGATT
TTGGAAGGCATGAAAGTGAAAAACCTGGATGACTATTTGAACGGTCCCTTCACTGTGGTAGTGAAAGAGTCCTGTGATGG
AATGGGAGATGTCAGTGAAAAGCATGGAAGTGGGCCTGCTGTCCCAGAGAAGGCTGTTCGCTTTTCATTCACAGTGATGA
ACATTGCTATAGCACATGGGAATGAAAACAAGAGGATCTTTGAAGAAGTAAAGCCCAATTCAGAGCTGTGTTGCAAGCCC
TTGTGCCTTATGCTGGCTGACGAATCAGATCATGAAACTCTGACGGCAATCCTGAGCCCCCTCATAGCAGAAAGAGAGGC
TATGAAAAACAGTGAACTGCTGCTTGAAATGGGAGGCATCCTGAGAACATTCAAATTCATCTTTAGGGGTACAGGGTATG
ATGAGAAACTTGTGCGGGAAGTGGAAGGGCTGGAGGCCTCAGGTTCCACTTACATTTGTACCCTGTGTGACGCAACCCGT
CTGGAGGCATCCAAGAATGTGGTCTTCCACTCCATAACCAGGAGCCATGCTGAAAATCTGGAGCGATATGAAATATGGAG
GTCCAACCCATATCATGAATCTGTTGATAAGCTCCGTGACAGAGTGAAGGGTGTTTCAGCCAAACCTTTTATAGAGACCG
TTCCCTCCATAGATGCGTTGCACTGTGACATTGGAAATGCAGCAGAATTCTACAGGATTTTCCACATGGAGATCTGTGAA
GTTTATAAGAATCCTGATGTGTCTAAAGAGGAGAGGAAGAGGTGGCAGTTGGCTCTTGACAAACACCTCAGGAAGAAGAT
GAACTTGAAGCCTATGATGAGGATGAGTGGAAATTTTGCTAGAAAGCTCATGTCCAAAGAGACAGTAGAGGCAGTATGTG
AATTAATAAAGTGTGAAGAAAGGCATGAGGCCTTAAAAGAACTAATGGACCTTTATCTGAAGATGAAGCCAGTGTGGCGA
TCCTCATGCCCTGCCAAGGAATGTCCAGAATTGCTGTGCCAGTATAGCTATAATTCACAGCGTTTTGCCGAGCTCCTATC
TACAAAGTTCAAGTACAGATATGAAGGCAAGATTACGAATTATTTCCACAAAACCCTTGCTCATGTTCCTGAAATCATTG
AAAGAGATGGGTCCATTGGGGCCTGGGCAAGTGAAGGAAATGAGTCTGGAAACAAACTGTTTAGGAGGTTCCGAAAAATG
AATGCCAGACAGTCCAAATGCTATGAGATGGAGGATGTCTTG
Ficedula_hypoleuca TAAATTGCGATCATTTGAAAAAACACCCT-------------------------
----
CTGAAGACAGCCAGCACATAAACAAAGATCAGGCAGAAGAGGCTGTTTCTTCAAACAAAGAATTCATTCTGCATAAAGAT
GAAGCAGTGCCAGTAGGAGAAAAGATGGAGTTAATGGGCAATAGGCAGGCACTTGAGGAAGATGCCCATGCCATGAAAAC
AAAAGACGATAGAGCTCATCAGCACAATCTGAAGCATCTCTGCCGCATCTGTGGAGTTTCATTTAAAACTGATTCTTACA
AGAAAACTTACCCAGTGCATGGGCCAGTGGATGATGAAACTCTGAGGCTTCTGAGAAAGAAAGAAAAAACAGCAACCTCA
TGGCCAGATCTTATTGCTAAGGTTTTCAAGATTGATGTGCGAGGGGATGTCGATACTATCCATCCCACTCAGTTTTGTCA
CAATTGTTGGAGTATTATACATAGCAAATACAGTAACACTCTATGTGAAGTATATTTTCCTAGAAACAGCACAATGGAGT
GGCAACCCCATTCCCCAAACTGTGATGTGTGCCATACTACCAGAAGAGGAGTCAAGAGAAAAAGCCAGCCCCCAAGTGTA
CAACGTGGCAAACGTGTCAAAACCACCGGGGAACGTGCTCAGCTAAACAGAAGTGTAAAGAAC------------
CAAGCACAGATAAACAACAAGAATTTAATGAAAGAGATTGTCAACTGCAAGGATATACATCTCAGCACCAAGCTGCTTGC
AGTTGATTACCCACCAGATTTCATTAAATCCATTTCTTGCCAGATTTGTGATCATATTTTGGCAGATCCAGTGGAAACAA
CATGCAGACACTTGTTTTGCAGAACCTGCATCCTTAAATGTATCAGGGTTATGGGCAGCTATTGCCCCTCCTGCTGGTAT
CCTTGCTTTCCTACTGATCTGGTAACCCCAGTGAAATCCTTCCTGAACATCCTTGATAACCTGGGTATAAGATGCCCTGT
AAAGGAATGTGATGAAGAGATCATGCATGGAAAGTATGGCCAACACCTCTCTGGCCACAAGGAGATGAAAGACAGAGAGC
TCCACAGCTACATAAATAAAGGTGGCCGACCGAGGCAGCACCTGCTGTCCCTGACCAGGAGAGCTCAGAAACATCGTCTG
AGGGAACTGAAAC?CCAAGTCAAGGCTTTTGCTGAGAAAGAAGAGGGCGGTGATATAAAGGCTGTATGCATGACTTTGTT
CTTGCTAGCTTTGAGAGCAAAAAATGAACACAAACAAGCAGATGAACTGGAGGCTATAATGCAAGGGAGGGGATCTGGAC
TTCATCCCGCTGTCTGTCTGGCCATCCGAATCAACACGTTTCTCAGCTGTAGTCAGTATCATAAAATGTACAGAACAGTA
AAAGCTGTCACTGGGAGGCAGATCTTCCAGCCTTTGCACGCTCTTCGCACTGCTGAGAAAGCCCTCCTCCCAGGGTATCA
CCCATTTGAGTGGAATCCTCCCCTGAAAATCGTATCCATTAACACAGAAGTGGGAATTATAGATGGACTGTCAGTACTTC
CACTCTCAATTGATGATTACCCAGTGGACACAATTGCAAAGAGATTCCGATATGATGCAGCCTTGGTTTGTGCCATAAAG
GTCATGGAAGAGGAGATCTTGGAAGGCATGAAAGCAAAAAACCTGGATGACTATTTGAATGGCCCCTTCACGGTGGTAAT
AAAAGAGTCCTGTGATGGAATGGGAGATGTCAGTGAGAAGCATGGAAGCGGGCCTGCTGTTCCAGAGAAGGCTGTTCGCT
TTTCTTTCACAGTCATGAACATYTYTATAGCACATGGGAACGAAAGCAAGAGGATCTTTGAGGAAGTAAAGCCCAATTCA
GAGCTGTGCTGTAAGCCCTTGTGCCTTATGCTGGCTGATGAATCAGATCATGAAACTCTGACAGCAATCCTGAGCCCCCT
CATTGCAGAAAGAGAGGCTATGAAAAACAGCGAACTGCTGCTTGAAATGGGAGGCATCCTGAGGACATTTAGATTCGTCT
TTAGGGGTACAGGATATGACGAGAAACTCGTGCGGGAAGTGGAAGGGCTGGAGGCCTCAGGCTCCACGTACATTTGTACC
CTGTGTGATGCCACCCGCTTGGAGGCATCCCAGAATCTGGTCTTCCACTCCATAACCAGGAGCCACGCTGAAAACCTGGA
GCGATATGAAATATGGAGGTCCAACCCGTACCACGAGTCTGTTGATGAGCTCCGGGACAGAGTGAAGGGTGTTTCAGCCA
AACCTTTTATTGAGACTGTTCCCTCCATAGATGCATTGCACTGTGACATTGGCAATGCAACGGAATTCTACAGGATCTTC
CAGATGGAGATTGGTGAACTTTACAAGAATCCTGACGTGTCCAAGGAGGAGAGGAAGAGGTGGCAGCTGACTCTTGACAA
ACACCTCAGGAAGAAGATGAACCTGAAGCCTATGCTGAAGATGAGTGGAAATTTTGCAAGAAAGCTCATGTCCAAAGAGA
CTGTAGAGGCAGTATGTGAATTAATAACGTGTGAGGAAAGGCACGAAGCCCTAAAAGAACTAATGGACCTTTACCTGAAG
ATGAAGCCGGTGTGGCGATCCTCATGCCCCGCCAAGGAGTGCCCAGAACTGCTGTGCCAGTACAGCTACAATTCACAGCG
TTTTGCAGAGCTCTTGTCTACAAAGTTCAAGTACAGATATGAGGGCAAGATTACAAATTATTTCCACAAAACACTTGCTC
ATGTTCCTGAAATCATTGAAAGAGATGGGTCCATTGGGGCCTGGGCAAGCGAAGGGAATGAGTCYGGAAACAAGCTGTTT
AGGAGGTTCCGAAAAATGAATGCCAGGCAGTCCAAAGTCTATGAGATGGAGGATGTCTTG

Menura_novaehollandiae TAAATTGCGATCATTTGAAAAAACACCCT-------------------------
----
CTGATGACAGCCAACACATAAACAAAGATCAGGCACAAGAGGCTGCTTCTTCAAATGAAGAATTAATCCTGCATAAAGAT
GAAGCAGTGCCAAGAGGAGAAAAGATGGAGTTAATGGACAATAGGCAGGGACTTGAGAAAGATGCCCATGACATGAAAAC
ACAAGACAATAGAGCTCATCAGAACAGGCTGAAGCAACTTTGCCGCATCTGTGGAGTTTCATTTAAAACTGATTGTTACA
AGAGAACTCACCCAGTGCATGGGCCGGTGGATGATGAAACTATGTGGCTTCTGAGAAAGAAAGAAAAAAAAGCAACCTCT
TGGCCAGATCTTATCTCTAAGGTGTTCAAGATTGATGTGCGAGGCGATGTCGATACTATCCATCCCACTCGATTTTGTCA
CAACTGTTGGAGTATTATACATAGAAAATTCAGTAATACTCTATGTGAAGTATATTTTCCTAGGAACAGCACAATGGAGT
GGCAACCCCACTCCCCAAACTGTGATGTGTGCCGTACTACCAGCCGAGGAGTCAAGAGAAAAAGCCAGCCCCCAAGCGTA
CAACACAGCAAACGTGTCAAAACCACTGGGGAACGTGCTCGACTCAACAGAGGTGTAAAGAAC------------
CAAGCACAGATAAACAACAAAAATTTAATGAAAGAGATTGTCAATTGCAAGAATATACATCTCAGCACCAAGCTGCTTGC
AGTTGATTATCCAGTAGATTTCATTAAATCCATTTCTTGCCAGATTTGTGAGCATGTTTTGGCAGATCCTGTGGAAACAA
CATGCAGACACTTGTTTTGCAGAACTTGCATCCTTAAGTGTATCAGGGTTATGGGCAGCTATTGCCCCTCCTGCTGGTAT
CCTTGCTTCCCTACTGATCTGGTAACCCCAGTGAAATCCTTCCTGAACATCCTTGATAACCTGTGTATAAGATGCCCTAT
ACAGGAATGTGATCAAGAGATCTTGCATGGAAAATATGGCCAACACCTCTCCAGCCACAAGGAGATGAAGGATAGAGAGC
TCTATAGCTACATAAATAAAGGTGGTCGACCGAGGCAGCACCTCCTGTCTTTGACAAGGAGAGCTCAGAAACATCGTCTG
AGGGAGCTGAAACGTCAAGTCAAGGCTTTTGCTGAGAAAGAAGAGGGTGGTGATATAAAGGCTGTATGCATGACTTTGTT
CCTGCTAGCTTTGAGGGCAAAAAATGAACACAAACAAGCAGATGAATTGGAGGCTATAATGCAAGGGAAGGGATCTGGAC
TTCATCCTGCTGTCTGTCTGGCAATCCGAGTCAACACGTTTCTCAGCTGTAGTCAGTATCATAAAATGTATAGAACAGTA
AAAGCTGTCTCTGGGAGGCAGATCTTCCAGCCTTTGCATGCTCTTCGCACTGCTGAGAAAGCCCTCCTGCCAGGTTATCA
CCCATTCGAGTGGAAACCTCCCTTAAAAAATGTATCCACAAACACAGAAGTGGGCATTATAGATGGACTATCAGGATTGC
CACTCTCTATTGATGACTATCCAGTGGACACAATTGCAAAGAGATTTCGCTATGATGCAGCCTTGGTTTGTGCCTTAAAG
GACATGGAGGAGGAGATCTTGGAAGGCATGAAAGCAAAAAACCTGGACGACTATTTGAATGGCCCCTTCACTGTGGTAGT
AAAAGAGTCCTGTGACGGAATGGGAGATGTCAGTGAGAAGCATGGAAGTGGGCCTGCTGTCCCAGAGAAGGCTGTTCGCT
TCTCTTTCACAGTCATGAACATTTCTGTAGCACATGAGAACGAAAGCAAGAGGATCTTTGAGGAAGTAAAGCCCAATTCA
GAGTTGTGTTGTAAGCCCTTGTGCCTTATGCTCGCTGATGAATCTGATCATGAAATTCTGACTGCAATCCTGAGCCCCCT
CATCGCAGAAAGAGAGGCTATGAAAAGCAGTGAACTGCTGCTTGAAATGGGAGGCATCCTGAGATCCTTCAGATTCATCT
TTAGGGGTACAGGATATGATGAGAAACTCGTGCGGGAAGTGGAAGGGCTGGAGGCCTCAGGTTCCACTTACATTTGTACC
CTGTGCGATGCAACCCGCTTGGAGGCGTCCCAGAATGTGGTCTTCCACTCCATAACCAGGAGCCATGCTGAAAATCTGGA
GCGATATGAAATATGGAGGTCCAACCCTTACCACGAATCTGTTGACGACCTCCGTGACAGAGTGAAGGGTGTTTCAGCCA
AACCTTTTATTGAGACCGTTCCCTCCATAGATGCATTGCACTGCGACATTGGCAATGCAACAGAATTCTACAGGATTTTC
CAGATGGAGATTGGTGAACTTTACAAGAATCCTGACGTGTCCAAAGAGGAGAGGAAGCGGTGGCAGTTGACTCTTGATAA
ACACCTCAGGAAGAAGATGAACTTGAAACCTATGCTGAGAATGACTGGAAATTTTGCTAGAAAGCTCATGTCCAAAGAGA
CAGTAGAGGCAGTATGTGAATTGATAAAGTGTGAGGAAAGGCATGAAGCCCTAAAAGAATTAATGGACCTTTATCTGAAG
ATGAAGCCAGTGTGGCGATCCTCATGCCCTGCCAAGGAGTGCCCAGAACTGCTGTGCCAGTACAGCTACAATTCACAGCG
CTTTGCAGAGCTCTTATCTACCAAGTTCAAGTACAGATATGAAGGCAAGATTACAAATTATTTCCACAAAACGCTTGCTC
ATGTTCCTGAAATCATTGAAAGAGATGGGTCCATTGGGGCCTGGGCAAGTGAAGGAAATGAGTCTGGAAACAAACTGTTT
AGGAGGTTCCGAAAAATGAATGCCAGGCAGTCCAAATGCTATGAGATGGAAGATGTCTTG
Pycnonotus CAAATTGCCATCATTTGAAAAAACACCCT-------------------------
----
CTGATGACAGCCAGCACATAAACAAAGACCAGGCAGAAGAGACTGTTTCTTCAAACAAAGAATTCATCCYGCGTAAAGAT
GAAGCGGTACTAAGAGGAGAAAAGATGGAGTTAACAGGCAATAGGCAGGAACTTGAGGAAGACGCCCATGCCATGAAAAC
ACAAGACAACAGAGCTCATCAGAACAATCTGAAGGAACTCTGTCGCATCTGTGGAGTTTCATTTAAAACTGATTGTAACA
AGAGAACTTACCCAGTCCATGGACCAGTAGATGATGAAACTCTGTCACTTTTGAGAAAGAAAGAAAAAACAGCAACCTCT
TGGCCAGATCTTATTGCTAAGGTTTTCAAGATTGATGTGCGAGGGGATGTCGATACTATCCATCCCACTCGATTTTGTCA
CAACTGCTGGAGTATTATACACAGAAAATTCAGTAATACTCCATGTGAAGTATATTTTCCTAGGAACAGCACAATGGAGT
GGCAACCCCATTCCCCAAACTGTGATGTGTGCCATACTACCAGACGAGGAGTCAAGAGAAAAAACCAGCCCCCAAGTGTA
CAACGTGGCAAACGTGTCAAAACCACTGGGGAACGTGCTCGGCTCAACAGAGGTGTAAAGAAC------------
CAAGCACAGATAAACAACAAAAATTTAATTAAAGAGATTGTCAGTTGCAAGGATATACATCTCAGCACCAAGCTGCTTGC
AATTGATTACCCACTAGATTTCATTAAATCCATCTCCTGCCAGATTTGTGATCATATTTTGGCAGATCCAGTGGAAACAA
CATGCAGACACTTGTTTTGCAGGACTTGCATCCTTAAATGTATCAGGGTTATGGGCAGCTATTGCCCCTCCTGCTGGTAT
CCTTGCTTTCCTACTGATCTGGTTACCCCAGTGAAATCCTTCCTGAACATCCTTGATAACTTGAGTATAAAATGCCCTGT
AAAGGAATGTGATGAAGAGATCTTGCATGGAAAATACGGCCAACACCTCTCTAGCCACAAGGAGATGAAAGACAGAGAGC
TCTATAGCTACATAAATAAAGGTGGCCGACCGAGGCAACACCTCCTGTCTTTGACGAGGAGAGCTCAGAAACATCGTCTG
AGGGAACTGAAACGTCAAGTCAAGACTTTTGCTGAGAAAGAAGAGGGTGGTGATATAAAGGCCGTATGCATGACTTTGTT
CCTGCTAGCTTTGAGAGCAAAAAATGAACACAAACAAGCAGATGAACTGGAGGCTATAATGCAAGGGAGGGGATCTGGAC
TCCATCCTGCTGTCTGTCTGGCCATCCGAATCAACACGTTTCTCAGCTGTAGTCAGTACCATAAAATGTATAGAACAGTA
AAAGCTGTCTCTGGGAGGCAGATCTTCCAGCCTTTGCATGCTCTTCGCACTGCTGAGAAAGCCCTACTACCAGGTTACCA
CCCATTTGAGTGGAAACCTCCCCTCAAAAATGTATCCGCTAACACAGAAGTGGGAATTATAGATGGACTGTCAGGACTGC
CACTCTCAATTGATGATTACCCGGTAGACACAATTGCAAAGAGATTCCGATATGATGCAGCCTTGGTTTGTGCCTTAAAA

GACATGGAGGAGGAGATCTTGGAAGGCATGAAAGCAAAGAACCTGGATGACTATTTGAATGGCCCTTTCACTGTGGTAGT
AAAAGAGTCCTGTGATGGAATGGGAGATGTCAGTGAGAAGCATGGAAGTGGGCCTGCTGTCCCAGAGAAGGCTGTTCGCT
TCTCCTTCACAGTCATGAACATTTCTATAGCATGTGGGAATGAAAGCAAGAGGATCTTTGAGGAAGTAAAGCCCAATTCA
GAGTTGTGCTGTAAGCCCTTGTGCCTTATGCTGGCTGATGAATCAGATCATGAAACTCTGACAGCAATCCTGAGCCCCCT
CATAGCAGAAAGAGAGGCTATGAAAAACAGTGAACTGCTGCTTGAAATGAGAGGCATCCTGAGAACATTTAAATTTGTCT
TCAGGGGTACAGGATATGATGAAAAACTCGTGCGGGAAGTGGAAGGGCTGGAGGCCTCAGGTTCCACTTACATTTGTACC
TTGTGTGATGCCACCCGTTTGGAGGCATCCCAGAATCTGGTCTTCCACTCCATAACCAGGAGCCATGCTGAAAATCTGGA
GAGATATGAAATATGGAGGTCCAACCCGTATCACGAATCTGTTGATGAGCTCCGTGACAGAGTGAAGGGTGTTTCAGCCA
AACCTTTTATTGAGACTGTTCCCTCCATAGATGCATTGCACTGTGACATTGGCAATGCAACAGAATTCTACAGGATTTTC
CAGATGGAGATTGGTGAACTTTACAAGAATCCTGATGTGTCTAAAGAGGAGAGGAAGAGGTGGCAGTTGGCTCTTGACAA
ACACCTCAGGAAGAAGATGAACTTGAAGCCTATGTTGAAGATGAGTGGAAATTTTGCTAGAAAGCTCATGTCCAAAGAGA
CTGTAGAGGCAGTATGTGAATTAATAAAATGTGAGGAAAGGCATGAAGCCCTGAAAGAACTAATGGACCTTTATCTGAAG
ATGAAGCCAGTGTGGCGATCGTCATGCCCTGCCAAGGAGTGCCCAGAACTGCTGTGCCAGTATAGCTACAATTCACAGCG
CTTTGCAGAACTCTTATCTACAAAGTTCAAGTACAGATATGAGGGCAAGATTACAAATTATTTCCACAAAACACTTGCTC
ATGTTCCTGAAATAATTGAAAGAGATGGGTCCATTGGGGCCTGGGCAAGTGAAGGAAATGAGTCTGGAAACAAACTGTTT
AGGAGGTTCCGAAAAATGAATGCCAGGCAGTCCAAAGTCTATGAGATGGAGGATGTCTTG
Donacobius_atricapilla TAAATTGCGGTCATTTGAAAAAACACCCT-------------------------
----
CTGATGACAGCCAGCACATAAACAAGGATCAGGCAGAAGAGGCTGTTTCTTCAAACAAGGAGTTCATCCTGCATAAAGAT
GAAGCAGTGCCAAGAGGAGAAAAGATGGAGTTAATGGGCAATAGGCAGGGACTTGAGGAAGATGCCCATGCCATGAAAGC
ACAAGACAATAGAGCTCATCAGAACAATCTGAAGCAACTCTGCCGCATCTGTGGAGTTTCATTTAAAACTGATTGTGACA
AGAGAACTTACCCAGTGCATGGGCCAGTGGATAATGAAACTCTGTGGCTTCTGAGAAAGAAAGAAAAAACAGCAACCTCT
TGGCCAGATCTTATTGCAAAGGTTTTCAAGATTGATGTGCGAGGGGATGTCGATACTATCCATCCCACTAGATTTTGTCA
CAACTGTTGGAGTATTATACACAGAAAATTCAGTAATACTCTGTGTGAAGTATATTTTCGTAGGAACAGCACAATGGAGT
GGCAACCCCATGCCCCAAACTGTGATGTGTGCCGTACCACCAGACGAGGAGTCAAGAGAAAAAAACAGCCTCCAAGTGTA
CAACATGGCAAACGTGTCAAAACCACTGGGGAACGTGGTCGGCTAAACAGAGGTGTAAAGAAC------------
CAAGCACAGATAAACAACAAAAATTTAATGAAAGAGATTGTCAATTGCAAGGATATACATCTCAGCACCAAGCTGCTTGC
AATTGATTACCCTGTAGATTTCATTAAATCCATCTCTTGCCAGATTTGTGATCATATTTTGGCAGACCCAGTGGAAACAA
CATGCAGACACTTGTTTTGCAGAACTTGCATCCTTAAATGTATCAGGGTTATGGGCAGCTATTGCCCCTCCTGCTGGTAT
CCTTGCTTTCCTACTGATCTAGTTACCCCAGTGAAATCCTTCCTGAACATCCTTGATARCTTGAGTATAAGATGCCCTGT
AAAGGAATGTGATGAAGAGACCTTGCATGGAAAATATGGCCAACACCTCTCCAGCCACAAGGAGATGAAAGATAGAGAGC
TCTACAGCTACATAAATAAAGGTGGCCGACCAAGGCAGCACCTCCTGTCTTTGACGAGGAGAGCTCAGAAACATCGTCTG
AGGGAACTGAAACGTCAAGTCAAGGCTTTTGCTGAGAAAGAAGAGGGTGGTGATATAAAGGCTGTGTGCATGACTTTGTT
CCTGCTAGCTTTGAGAGCAAAAAATGAACACAAACAAGCAGATGAACTGGAGGCTATAATGCAAGGGAGGGGATCTGGAC
TTCATCCTGCTGTCTGTCTGGCCATCCGAATCAACACGTTTCTCAGCTGTAGTCAGTATCATAAAATGTATAGAACAGTG
AAAGCTGTCTCTGGGAGGCAGATCTTCCAACCTTTGCATGCTCTTCGCACTGCTGAGAAAGCCCTCCTACCAGGTTATCA
CCCATTTGAGTGGAAACCTCCCCTGAAAAATGTATCTGCCAACACAGAGGTGGGAATTATAGATGGCCTATCAGGACTGC
CACTCTCAATTGATGATTACCCAGTAGACACAATTGCAAAGAGATTCCGATATGATGCAGCCCTGGTTTGTGCCTTAAAA
GACATGGAGGAGGAGATCTTGGAAGGCATGAAAGCAAAAAGCCTGGACGACTATTTGAATGGCCCCTTCACTGTGGTAAT
AAAAGAGTCCTGTGACGGAATGGGAGATGTCAGTGAGAAGCATGGAAGTGGGCCTGCTGTCCCAGAGAAGGCTGTTCGCT
TTTCCTTCACAGTGATGAACATTTCTATAGCACATGGGAACGAAAGCAAGAGGATCTTTGAGGAGGTAAAGCCCAATTCA
GAGTTGTGCTGTAAGCCCTTGTGCCTTATGCTGGCTGATGAATCAGATCATGAAACTCTGACAGCAATCCTGAGCCCCCT
CATAGCAGAAAGAGAGGCTATGAAAAACAGTGAACTGCTGCTTGAAATGGGAGGCATCCTGAGAACATTTAGATTTGTCT
TTAGGGGTACAGGATATGATGAAAAACTTGTGCGAGAAGTAGAAGGGCTGGAGGCTTCTGGTTCCACTTATATTTGTACC
TTGTGTGATGCAACCCGACTGGAGGCATCCCAGAATCTGGTCTTCCACTCCATAACCAGGAGCCATGCTGAAAATCTGGA
GCGATATGAAATATGGAGGTCCAATCCATATCACGAGTCTGTTGATGAGCTCCGTGACAGAGTGAAGGGTGTTTCAGCCA
AACCTTTTATTGAGACTGTTCCCTCCATAGACGCATTGCACTGCGACATTGGCAATGCAACAGAATTCTACAGGATTTTC
CAGATGGAGATTGGTGAACTTTACAAGAATCCTGACGTGTCTAAAGAGGAGAGGAAGAGGTGGCAGTTGGCTCTTGACAA
ACACCTCAGGAAGAAGATGAACTTGAAGCCTATGTTGAAGATGAGTGGAAATTTTGCTAGAAAGCTCATGTCCAAAGAGA
CTGTAGAGGCAGTATGTGAATTAATAAAGTGTGAGGAAAGGCATGAAGCTCTAAAAGAACTAATGGACCTTTATCTGAAG
ATGAAGCCAGTGTGGCGATCCTCATGCCCTGCCAAGGAGTGCCCAGAACTGCTGTGCCAGTATAGCTACAATTCACAGCG
TTTTGCAGAACTCTTATCTACAAAGTTCAAGTATAGATATGAGGGCAAGATTACAAATTATTTCCACAAAACACTTGCTC
ATGTTCCTGAAATCATTGAAAGAGATGGGTCCATTGGCGCCTGGGCAAGCGAAGGAAATGAGTCTGGAAACAAACTGTTT
AGGAGGTTCCGAAAAATGAATGCCAGACAGTCCAAAGTCTATGAGATGGAGGATGTCTTG
Hirundo_rustica TAAATTGCGATCATTTGAAAAAACACCCT-------------------------
----
CAGATGACCGCCAGCACATAAACAAAGATCAGGCAGAAGAGGCTGTTTCTTCAAACAAAGAATTGACCCTGCATAAAGAT
GAAGCAGTGTCAAGAGGAGAAAAGATGGARTTAACGGGCAATAGGCATGGACTTGAGGAAGATGTCCATGCCATGAAAAC
ACAAGACAATAGAGCTCATCAGAACAATCTGAAGCAACTCTGCCGCATCTGTGGAGTTTCATTTAAAACTGATGGTCACA
AGAGAACTTACCCAGTGCACGGGCCAGTGGATGACGAAACTCTGAGGCTTCTGAGAAAGAAAGAAAAAACAGCAACCTCT

TGGCCAGACCTTATTGGTAAGGTTTTCAAGATTGATGTGMGAGGGGATGTCGATACTATCCATCCCACTCGATTTTGTCA
CAACTGTTGGAGTATTATACACAGAAAATTCAGTAATACTCTATGTGAAGTATATTTTCCTAGGAACAGCACAATGGAGT
GGCAACCCCATTCCCCAAACTGTGATGTGTGCCATACTACCAGGCGAGGAGTCAAGAGAAAAAGACAGCCCCCAWGTGTG
CAACATGGCAAACGTGTCAAAACCACTGGGGAACGTACTCGGCTAAACAGAGGTGTAAAGAAC------------
CAAGCACAGATAAACAACAAAAATTTAATTAAAGAGATTGTCAATTGCAAAGATATACACCTCAGCACCAAGCTGCTTGC
AATTGATTACCCAGTAGATTTCATTAAATCCATTTCTTGCCAGATTTGTGATCATATTTTGGCAGATCCAGTGGAAACAA
CATGCCGACACTTGTTTTGCAGAACTTGCATCCTTAAATGTATCAGGGTTATGGGCAGCTATTGCCCCTCCTGCTGGTAT
CCTTGCTTTCCTACTGATCTGGTTACCCCAGTGAAATCCTTCCAGAACATCCTTGATAACTTGRGTATAAGATGCCCTGT
AAAAGAATGTGATGAAGAGATCTTGCATGGAAAATATGGCCAGCACCTTTCCAGCCACAGGGAGATGAAAGATAGAGAGC
TCTATAGCTACATAAATAAAGGTGGCCGACCGAGGCAGCACCTCCTGTCTTTGACGAGGAGAGCTCAGAAACATCGTCTG
AGGGAACTGAAACGTCAAGTCAAGACTTTTGCTGAGAAAGAAGAGGGTGGTGATATAAAGGCTGTATGCATGACTTTGTT
CCTGCTAGCTTTGAGAGCAAAAAATGAACACAAACAAGCAGACGAACTGGAGGCTATAATGCAAGGGAGGGGATCTGGAC
TTCATCCTGCTGTATGTCTGGCCATCCGAATCAACACGTTTCTCAGCTGCAGTCAGTATCATAAAATGTATAGAACAGTA
AAAGCTGTCAGTGGGAGGCAGATCTTCCAGCCTTTGCATGCTCTTCGCACWGCTGAGAAAGCCCTCCTACCAGGTTATCA
CCCATTTGAGTGGAAACCTCCCCTGAAAAATGTATCCACTAACACAGAAGTGGGAATTATAGATGGACTATCAGGACTGC
CACTCTCAATTGAYGATTACCCAGTAGACACAATTGCAAAGAGGTTCCGATATGATGCAGCCTTGGTTTGTGCCTTAAAA
GACATGGAGGAGGAGATCTTGGAAGGCATGAAAGCAAAAAACCTGGATGACTATTTGAATGGCCCCTTCACTGTGGTAGT
AAAAGAGTCCTGTGACGGAATGGGAGATGTCAGTGAGAAGCATGGAAGTGGGCCTACTGTCCCAGAGAAGGCTGTTCGCT
TTTCCTTCACAGTCATGAACATTTCTATAGCACAAGGGAATGAAAGCAAGAGGATCTTTGAGGAAGTAAAGCCCAATTCA
GAGTTGTGCTGTAAGCCCTTGTGCCTTATGCTGGCTGATGAATCAGATCATGAAACTCTGACAGCAATCCTGAGTCCCCT
CATAGCAGAACGAGAGGCTATGAAGAACAGTGAACTCCTGCTTGAAATGGGAGGCATCCTGAGAACATTTAGATTTGTCT
TCAGGGGTACAGGATATGATGAGAAACTCGTGCGGGAAGTGGAAGGGCTGGAGGCCTCAGGTTCCACTTACATTTGTACC
CTGTGTGACGCAACCCGCTTGGAGGCATCCCAGAATCTGGTCTTCCACTCCATAACCAGGAGCCATGCTGAAAATCTGGA
GCGATATGAAATATGGAGGTCCAACCCATATCATGAGTCTGTTGATGAGCTCCGTGACAGAGTGAAGGGTGTTTCAGCCA
AACCTTTTATTGAGACTGTTCCCTCCATAGATGCATTGCACTGCGACATTGGAAATGCAACAGAATTCTACAGGATTTTC
CAGATGGAGATTGGTGAACTTTACAAAAATCCTGACGTGTCTAAAGAGGAGAGGAAGAGGTGGCAGTTGGCTCTTGACAA
ACACCTCAGGAAGAAGATGAACTTGAAGCCTATGTTGAAGATGAGTGGAAATTTTGCTAGAAAGCTCATGTCCAAAGAGA
CTGTAGAGGCAGTGTGTGAATTAATAAAATGTGAGGAAAGGCATGAAGCCCTAAAAGAACTAATGGATCTTTATCTGAAG
ATGAAGCCAGTGTGGCGATCCTCATGCCCTGCCAAGGAGTGCCCAGAACTGCTGTGCCAGTATAGCTACAATTCACAGCG
TTTTGCAGAACTCTTATCTACAAAGTTCAAATACAGATATGAGGGCAAGATTACAAATTATTTCCACAAAACACTTGCTC
ATGTTCCTGAAATCATTGAAAGAGATGGGTCCATTGGGGCCTGGGCAAGTGAAGGAAATGAGTCTGGAAACAAACTGTTT
AGAAGGTTCCGAAAAATGAATGCCAGGCAGTCCAAAGTCTATGAGATGGAGGATGTCTTG
Lichenostomus
??
??
??
??
??
??
??
??
??
??
??
??
??ATAAGATGCC
CTGTAAAGGAATGTGATGAAGAGATCTTGCATGGAAAATATGGCCAGCATCTCTCCAGCCACAAGGAGATGAAAGATAGA
GAGCTCTACAGCAACATAAATAAAGGTGGCCGACCGAGGCAGCACCTCCTGTCTTTGACAAGGAGAGCTCAGAAACATCG
TCTGAGGGAACTGAAACGTCATGTCAAGGCGTTTGCTGAGAAAGAAGAGGGTGGTGATATAAAGGCTGTATGCATGACTT
TGTTCCTGCTAGCTTTGAGAGCAAAAAATGAACACAAACAAGCAGATGAACTGGAGGCTATAATGCAGGGGAGGGGATCT
GGACTTCATCCTGCTGTCTGTCTGGCAATCCGAGTCAACACATTTCTCAGCTGTAGTCAGTATCATAAAATGTACAGAAC
AGTGAAAGCTGTCTCTGGGAGGCAGATCTTCCAGCCTTTGCATGCTCTTCGCACTGCTGAGAAAGCCCTCCTACCAGGTT
ATCACCCATTTGAGTGGAAACCTCCCTTGAAAAATGTATCCACTAACACAGAAGTGGGAATTATAGATGGACTATCAGGA
CTGCCACTCTCAATTGATGACTACCCAGTAGACACAATTGCAAAGAGATTTCGATATGATGCAGCCTTGGTTTGTGCCTT
AAAGGACATGGAGGAGGAGATCTTGGAAGGCATGAAAGCAAAAAACCTGGACAACTATTTGGATGGCCCCTTCACTGTGG
TAGTGAAAGAGTCCTGTGATGGAATGGGAGATGTCAGTGAGAAGCATGGAAGTGGGCCTGCTGTCCCGGAGAAGGCTGTT
CGCTTTTCTTTCACAGTCATGAACATTTCTATTGCACATGGGAATGAAAGCACAAGGATCTTTGAGGAAGTAAAGCCCAA
TTCAGAGTTGTGCTGT??
??
??

???
???
???
???
???
???
???
???
???
???
Cnemophilus TAAATTGCGATCATTTGAAAAAACATCCT---------------------------
----
CTGATGACAGCCAGCACATAAACAAAGATCAGGCAGAAGAGGCTGTTTCTTCAAACAAAGAATTAATCCTGCATAAAGAT
GAAGCAGTGCCAAGAGGAGAAAAGATGGTGTTAATGGGCAATGGGCAGGGACTTGAGGAAGATGCCCGTGCCATGAAAAT
ACAAGACAAYAGAGCTCATCAGAACAATTTGAAGCAACTCTGCCGCATCTGTGGAGTTTCATTTAAAACTGATTGTTACA
AGAGAACTTACCCAGTGCATGGACCAGTGGATGATGAAACACTGTGCCTTCTGAGAAAGAAAGAAAAAACAGCAACCTCT
TGGCCAGATCTTATTGCTAAGGTTTTCAAGATTGATGTGCGAGGGGATGTTGATACCATCCATCCCACTCGATTTTGTCA
CAACTGTTGGAGTATTATACATAGAAAATTCAGTAATACTTTATGTGAAGTATATTTTCCTAGGAACAGCACAATGGAGT
GGCAACCCCACTCCCCAAACTGTGATGTGTGCCAGACTACCAGACGAGGAGTCAAGAGAAAAAGCCAGCCCCCAAATGTA
CAACATGGCAAACGTGTCAAAACCACTGCGGAACGTACTCGGCTAAACAGAGGTGTAAAGAAC------------
CAAGCACAGACAAACAACAAAAATTTAATGAAAGAGATTGTCAATTGCAAGGATATCCATCTCAGCACCAAGCTGCTTGC
AGTTGATTACCCAGTAGATTTCATTAAATCCATTTCTTGCCAGATTTGTGATCATATTTTGGCAGATCCAGTGGAAACAA
CATGCAGACACTTGTTTTGCAGAACTTGCATCCTTAAATGTATCAGGGTTATGGGCAGCTATTGCCCCTCCTGCTGGTAT
CCTTGCTTTCCTACCGATCTGGTAACTCCAGTGAAATCCTTCCTAAGCATCCTTGATAACCTGAGTATAAGATGYCCTGT
AAAGGAATGCGATGAAGAGATCTTGCATGGAAAATATGGCCAACACCTCTCCAGCCACAAGGAGATGAAAGATAGAGAGC
TCTATAGCTACATAAATAAAGGTGGCCGACCAAGGCAGCACCTCCTGTCTTTGACGAGGAGAGCTCAGAAACATCGTCTG
AGGGAACTGAAACGTCAAGTCAAGGCTTTTGCTGAGAAAGAAGAGGGCGGTGATATAAAGGCTGTATGCATGACTTTGTT
CCTGCTAGCTTTGAGAGCAAAAAATGAACACAAACAAGCAGATGAACTGGAGGCTATAATGCAAGGGAGGGGATCTGGAC
TTCATCCTGCTGTCTGTCTGGCCATCCGAATCAACACATTTCTCAGCTGTAGTCAGTATCATAAAATGTATAGAACAGTA
AAAGCTGTCACTGGGAGGCAGATCTTCCAGCCTTTGCATGCTCTTCGCACTGCTGAGAAAGCCCTCCTACCAGGTTATCA
CCCGTTTGAGTGGAAACCTCCCTTGAAAAATGTATCCACTAACACAGAAGTGGGAATTATAGATGGACTATCAGGACTGC
CACTCTCAATTGACGACTACCCAGTAGACACAATTGCAAAGAGATTTCGATATGATGCGGCCTTGGTTTGTGCCTTAAAG
GATATGGAGGAGGAGATCTTGGAAGGCATGAAAGCAAAAAACCTGGATGACTATTTGAATGGCCCCTTCACTGTGGTAGT
AAAAGAGTCCTGTGATGGAATGGGAGATGTCAGTGAGAAGCATGGAAGTGGGCCTGCTGTCCCAGAGAAGGCTGTTCGCT
TTTCTTTCACAGTCATGAATATTTCTATAGCACATGGGAATGAAAGCACGAGGATCTTTGAGGAAGTAAAGCCCAATTCA
GAGTTGTGCTGTAAGCCCTTGTGCCTTATGCTGGCTGATGAATCAGATCATGAAACTCTGACAGCAATCCTGAGCCCCCT
CATAGCAGAAAGAGAGGCTATGAAAAACAGTGAACTGCTGCTTGAAATGGGAGGCATCCTGAGAACATTTAGATTCATCT
TTAGGGGTACGGGATATGATGAGAAACTCGTGCGGGAAGTGGAAGGGCTGGAGGCCTCAGGTTCCACTTACATTTGTACT
CTGTGTGATGCAACTCGCTTGGAGGCATCCCAGAATCTGGTCTTCCACTCCATAACGAGGAGCCACGCTGAAAATCTGGA
GCGATATGAAATATGGAGGTCCAACCCATATCACGAATCTGTTGATGAGCTCCGTGACAGAGTGAAGGGTGTTTCAGCCA
AACCTTTTATTGAGACTGTTCCCTCCATAGATGCATTGCACTGCGACATTGGCAATGCGACAGAATTCTACAGGATTTTC
CAGATGGAGATTGGTGAACTTTACAAGAATCCTGAYGCGTCTAAAGAGGARAGGAAGAGGTGGCAGTTGACTCTTGACAA
ACACCTCAGGAAGAAGATGAACTTGAARCCTATGTTGAAGATGAGTGGAAATTTTGCTAGAAAGCTCATGTCCAAAGAGA
CTGTAGAGGCWGTATGTGAATTAATAAAGTGTGAGGAAAGGCATGAGGCAYTAAAAGAACTAATGGACCTTTATCTGAAG
ATGAAGCCAGTGTGGCGATCCTCATGCCCTGCCAAGGAGTGCCCAGAACTGTTGTGCCAGTACAGCTACAACTCACAGCG
TTTTGCAGAGCTCTTGTCTACRAAGTTCAAGTACAGATATGAAGGCAAGATTACAAATTATTTCCACAAAACACTTGCTC
ATGTTCCTGAAATCATTGAAAGAGATGGGTCCATTGGGGCCTGGGCGAGTGAAGGAAATGAGTCTGGAAACAAACTGTTT
AGGAGGTTCCGAAAAATGAATGCCAGGCAGTCCAAAGTCTATGAGATGGAGGATGTCTTG
Coracina TAAATTGCGATCATTTGAAAAAACACCCT---------------------------
----
CTGATGACAGCCAGCACATAAACAAAGATCAGGCAGAAGAGGCTGTTTCTTCAAACAAAGAATTAATCCTGCATAAAGAT
GAAGCAGTGCCAAGAGGAGAAAAGATGGAGTTAACGGGCAATAGGCAGGGACTTGAGGAAGATGCCCATGCCATGGAAAC
ACAAGACAATASAGCTCATCAGAACAATTTGAAGCAACTCTGCCGCATCTGTGGAGTTTCATTTAAAACTGATTGTTACA
AGAGAACTTACCCAGTGCACGGGCCAGTGGATGATGAAACTCTGTGGCTTCTCAGAAAGAAAGAAAAAACAGCAACCTCT
TGGCCAGACCTTATTGCTAAAGTTTTCAAGATTGATGTGCGAGGGGATGTCGATACTATCCATCCCACTCAATTTTGTCA
CAACTGTTGGAGTATTATACATAGAAAATTCAGTAATACTCTATGTGAAGTATATTTTCCTAGGAACAGCACAATGGAGT
GGCAACCGCACTCCCCAAAATGTGATGTGTGCCACACTACCAGACGAGGAGTCAAGAGAAAAAGCCAGCCCCCAAGTGTA
CAACGTCGCAAACGTGTCAAAACCACTGGGGAACGTGCTCGGCTAAACAGGAGAGTAAAGAAC------------
CAAGCACAGATAAACAACAAAAATTTAATGAAAGAGATTGTCAATTGCAAGGATATACATCTCAGCACCAAGCTCCTTGC
AGTTGATTACCCAGTAGATTTCATTAAATCCATTTCTTGCCAGATTTGTGATCATATTTTGGCAGATCCAGTGGAAACAA

CATGCAGACACTTGTTCTGCAGAACTTGCATCCTTAAATGTATCAGGGTTATGGGCAGCTATTGCCCTTCCTGCTGGTAT
CCTTGCTTTCCTACTGATCTGGTAACCCCAGTCAAATCCTTCCTGAACATCCTTGATAACCTGAGTATAAGATGCCCTGT
AAAGGAATGTGATGAAGAGATCTTGCATGGAAAATATGGCCAACACCTCTCCAGCCACAAAGAGATGAAAGATAGAGAGC
TCTATAGCTACATAAATAAAGGTGGCCGACCGAGGCAGCACCTCCTGTCTTTGACGAGGAGAGCTCAGAAACATCGTCTG
AGGGAGCTGAAACGTCAAGTCAAGGCTTTTGCTGAGAAAGAAGAGGGTGGTGATATAAAGGCTGTATGCATGACTTTRTT
CTTGCTAGCTTTGAGAGCAAAAAATGAACACAAACAAGCAGATGAACTAGAGGCTATAATGCAAGGGAGGGGATCTGGAC
TTCATCCTGCTGTCTGTCTGGCCATCCGAATCAACACGTTTCTCAGCTGTAGTCAATATCACAAAATGTATCGAACAGTA
AAAGCTGTCACTGGGAGGCAGATCTTCCAGCCTTTGCATGCTCTTCGCACTGCTGAGAAAGCCCTCCTACCAGGTTATCA
CCCATTCGAGTGGAAACCTCCCTTGAAAAATGTATCCACTAACACAGAAGTGGGAATTATAGATGGACTGTCAGGACTGC
CACTCTCAATTGATGACTACCCAGTAGACACAATTGCAAAGAGATTTCGATATGATGCGGCCTTGGTTTGTGCCTTAAAG
GACATGGAGGAGGAGATCTTGGAAGGCATGAAAGCAAAAAACCTGGACGACTATTTGAATGGCCCCTTCACTGTGGTAGT
AAAAGAGTCCTGTGATGGAATGGGAGATGTCAGTGAGAAGCATGGGAGTGGACCTGCTGTCCCAGAGAAGGCTGTTCGCT
TTTCTTTCACAGTCATGAACATTTCTATAGCACATGGGAATGAAAGCAAGAGGATCTTTGAGGAATTAAAGCCCAATTCA
GAGTTGTGCTGTAAGCCCTTGTGCCTTATGCTGGCTGACGAATCAGATCACGAAACTCTGACGGCAATCCTGAGCCCCCT
CATAGCAGAAAGAGAGGCTATGAAAAACAGTGAACTGCTGCTTGAAATGGGAGGCATCCTGAGAACATTTAGATTTGTCT
TTAGGGGTACAGGATATGATGAGAAACTTGTGCGGGAAGTGGAAGGGCTGGAGGCTTCAGGTTCCACTTACATTTGTACC
CTGTGTGATGCAACCCGTTTGGAGGCATCCCAGAATCTGGTCTTCCACTCCATAACCAGGAGCCATGCTGAAAATCTGGA
GCGATATGAAATATGGAGGTCCAACCCATATCATGAATCTGTTGATGAGCTCCGTGACAGAGTGAAGGGTGTTTCAGCCA
AACCTTTTATTGAGACTGTTCCCTCCATAGATGCATTGCACTGCGATATTGGCAATGCAACAGAATTCTACAGGATTTTC
CAGATGGAGATTGGTGAACTTTACAAGAATCCTGACGTGTCTAAAGAGGAGAGGAAGAGGTGGCAGTTGACTCTTGACAA
ACATCTCAGGAAGAAGATGAACTTGAAGCCCATGTTGAAGATGAGTGGAAATTTTGCTAGAAAGCTCATGTCCAAAGAGA
CCGTAGAGGCAGTATGTGAATTAATAAAATGTGAGGAAAGGCATGAAGCCCTAAAAGAACTAATGGACCTTTATCTGAAG
ATGAAGCCAGTGTGGCGATCCTCATGCCCTGCCAAGGAGTGCCCAGAACTGCTGTGCCAGTATAGCTACAATTCACAGCG
TTTTGCGGAGCTCTTATCTACAAAGTTCAAGTACAGATATGAGAGCAAGATTACAAATTATTTCCACAAAACGCTTGCTC
ATGTTCCTGAAATCATTGAAAGAGATGGGTCCATTGGGGCCTGGGCAAGTGAAGGAAATGAGTCTGGAAACAAACTGTTT
AGGAGGTTCCGAAAAATGAATGCCAGGCAGTCCAAAGTCTATGAGATGGAGGATGTCTTG
Dicrurus
??
??
??
??
??
??
??
??
??
??
??
??
??ATAAGATGCC
CTGTAAAGGAATGTGATGAAGAGATCTTGCATGGAAAATATGGCCAACACCTCTCCAGCCACAAGGAGATGAAAGATAGA
GAGCTCTATAGCTACGTAAATAAAGGTGGCCGACCGAGGCAGCACCTCCTGTCTTTGACGAGGAGAGCTCAGAAACATCG
TCTGAGAGAACTGAAACGTCAAGTTAAGGCTTTTGCTGAGAAAGAAGAGGGCGGTGATATAAAGGCTGTATGCATGACTT
TGTTCCTGCTAGCTTTGAGAGCAAAAAATGAACACAAACAAGCAGATGAACTGGAGGCTATAATGCAAGGGAGGGGATCT
GGACTTCATCCTGCTGTCTGTCTGGCCATCCGAATCAACACGTTTCTCAGCTGTAGTCAGTATCATAAAATGTATAGAAC
GGTAAAAGCTGTCACTGGGAGGCAGATCTTCCAGCCTTTGCATGCTCTTCGCACTGCTGAGAAAGCCCTCCTACCAGGTT
ATCACCCATTTGAGTGGAAACCTCCCTTGAAAAATGTATCCACTAACACAGAAGTGGGAATTATAGATGGACTATCAGGT
CTGCCACTCTCAGTTGATGACTACCCAGTAGACACAATTGCAAAGAGATTTCGATATGATGCGGCCTTGGTTTGTGCCTT
AAAGGACATGGAGGAGGAGATCTTGGAAGGCATGAAAGCAAAAAACCTGGATGACTATTTGAATGGCCCCTTCACTGTGG
TAGTAAAAGAGTCCTGTGATGGAATGGGAGATGTCAGTGAGAAGCATGGAAGTGGGCCTGCTGTCCCAGAGAAGGCTGTT
CGCTTTTCTTTCACAGTCATGAACATTTCTATAGCACAGGGGAATGAAAGCAAGAGGATCTTTGAGGAGGTAAAGCCCAA
TTCA?AGTTGTGCTGC??
??
??
??
??
??
??
??
??

??
??
??
??
Gerygone_fusca
??
??
??
??
??
??
??
??
??
??
??
??
??ATAAGATGTC
CTGTAAAGGAATGTGATGAAGAGATCTTGCATGGGAAATATGGCCAACACCTCTCCAGCCACAAAGAGATGAAAAGTGGA
GAGCTCTGCAGCTATATAAATAAAGGTGGCCGACCGAGGCAGCACCTCCTGTCTTTGACGAGGAGAGCTCAGAAACATCG
TCTGAGGGAACTGAAACGTCAAGTCAAGGCTTTTGCTGAGAAAGAAGAGGGTGGTGATATAAAGGCTGTATGCATGACTT
TGTTCCTGCTAGCTTTGAGAGCAAAAAATGAACACAAACAAGCAGATGAACTAGAGGCTATAATGCAAGGGAGGGGATCT
GGACTTCATCCTGCTGTCTGTCTGGCAATCCGAATCAACACATTTCTCAGCTGTAGTCAGTATCACAAAATGTACAGAAC
AGTAAAAGCTGTCACTGGGAGGCAGATCTTCCAGCCTCTGCATGCTCTTCGCACTGCTGAGAAAGCCCTCCTACCAGGTT
ATCACCCATTCGAGTGGAAACCTCCCCTGAAAAATGTATCCACTAACACAGAAGTGGGAATTATAGATGGACTATCAGGA
CTGCCACTCTCAATTGATGACTACCCAGTGGACACAATTGCAAAGAGATTTCGATATGATGCAGCCTTGGTTTGTGCCTT
AAAGGACATGGAGGAGGAGATCTTGGAAGGCATGAAAGCAAAAAACCTGGATGACTATTTGAACGGCCCTTTCACTGTAG
TAGTGAAAGAGTCCTGTGATGGAATGGGAGATGTCAGTGAGAAGCACGGAAGYGGGCCTGCTGTGCCAGAGAAGGCTGTT
CGCTTTTCTTTCACAGTCATGAACATTTCTATAGCACATGGGAATGAAAGCACGAGGATCTTTGAGGAAGTAAAGCCCAA
TTCAGAGTTGTGCTGT??
??
??
??
??
??
??
??
??
??
??
??
??
Ptiloris_magnificus   TAAATTGCGATCATTTGAAAAAACACCCT-------------------------
----
CTAATGACAGCCAGCACATAAACAAAGATCTGGCAGAAGAGGCTGTTTCTTCAAACAAAGAATTCATCCTGCATAAAGAT
GAAGCAGTGCCAAGAGGAGAAAAGATGGAGTTAACGGGCAATAGGCAGGGACTTGAGGAAGATGCCCATGCCATGCAAAC
ACAAGACAATAGAGCTCATCAAAACAATTTGAAGGAACTCTGCCGCATCTGTGGAGTTTCATTTAAAACTGATTGTTCCA
AGAGGACTTACCCAGTGCATGGGCCAGTGGATGATGAAACTCTGTGGCTTCTGAGAAAGAAAGAAAAAACAGCAACCTCT
TGGCCAGACCTTATTGCTAAGGTTTTCAAGATTGATGTGCGAGGGGATGTTGATACTATCCATCCCACTCAATTTTGTCA
CAACTGTTGGAGTATTATACATAGAAAATTCAGTAATACTCTATGTGAAGTATATTTTCCTAGGAACAGCACAATGGAGT
GGCAACCGCACTCCCCGAACTGTGACGTGTGCCATACTACCAGACGAGGAGTCAAGAGAAAAAGCCAGCCCCGAAGTGTA
CAACRTGGCAAACGTGTCAAAACCACTGGAGAACGTGCTCGGCTAAACAGAGGTGTAAAGAAC------------
CAAGCACAAATAAACAACAAAAATTTAATGAAGGAGATTGTCAATTGCAAGGATATACATCTCAGCACCAAGCTGCTTGC
AGTTGATTACCCAGTAGATTTCATTAAATCCATTTCTTGCCAGATTTGCGATCATATTTTGGCAGATCCAGTGGAAACAA
CATGCAGACACTTGTTTTGCAGAACTTGCATCCTTAAATGTGTCAGGGTTATGGGCAGCTATTGCCCCTCCTGCTGGTAT
CCTTGCTTTCCTACTGATCTGGTAACCCCAGTGAAATCCTTCCTGAACATCCTTGATAACCTGAGTATAAGATGCCCTGT
AAAGGAATGTGATGAAGAGATCTTGCATGGAAAATATGGCCAACACCTCTCCAGCCACAAGGAGATGAAAGATAGAGAGC
TCTATAGCTACATAAATAAAGGTGGCCGACCGAGGCAGCACCTCCTGTCTTTGACGAGGAGAGCTCAGAAACATCGTCTG
AGGGAGCTGAAACGTCAAGTCAAGGCTTTTGCTGAGAAAGAAGAGGGTGGTGATATAAAGGCTGTATGCATGACTTTGTT
CCTGCTAGCTTTGAGAGCAAAAAATGAACACAAACAAGCAGATGAACTGGAGGCTATAATGCAAGGGAGGGGATCTGGAC

TTCATCCTGCTGTCTGTCTGGCCATCCGAATCAACACTTTTCTCAGCTGTAGTCAGTATCATAAAATGTATAGAACAGTA
AAAGCTGTCAGTGGGAGGCAGATCTTCCAGCCTTTGCATGCTCTTCGCACTGCTGAGAAAGCCCTCCTACCAGGTTATCA
CCCATTTGAGTGGAAACCTCCMTTGAAAAATGTATCCACTAACACAGAAGTGGGAATTATAGATGGACTATCAGGACTGC
CACTCTCAATTGATGACTACCCAGTAGACACAATTGCAAAGAGATTTCGATATGATGCAGCCTTGGTTTGTGCCTTAAAG
GACATGGAGGAGGAGATCTTGGAAGGCATGAAAGCAAAAAACCTGGATGACTACTTGAACGGCCCCTTCACTGTGGTAGT
GAAAGAGTCSTGTGATGGAATGGGAGATGTCAGTGAGAAGCATGGAAGTGGGCCTGCTGTCCCAGAGAAGGCTGTTCGCT
TTTCTTTCACAGTCATGAACATTTCTGTAGTACAGGGGAATGAAAGCAAGAGGATCTTTGAGGAAGTAAAGCCCAATTCA
GARTTGTGCTGCAAGCCCTTGTGCCTTATGCTGGCTGACGAATCAGATCATGAAACTCTGACRGCAATCCTGAGCCCCCT
CATAGCAGAAAGAGAGGCTATGAAAAACAGTGAACTGCTGCTTGAAATGGGAGGCATCCTGAGAACATTTAGATTCATCT
TTAGGGGTACAGGATATGATGAGAAACTCGTGCGGGAAGTGGAAGGGCTGGAGGCCTCAGGTTCCACTTACATTTGTACC
CTGTGCGATGCAACCCGCTTGGAGGCGTCCCAGAATCTGGTCTTCCACTCCATAACCAGGAGCCATGCTGAAAATCTGGA
GCGATATGAAATATGGAGGTCCAACCCATATCACGAATCTGTTGATGAGCTCCGTGACAGAGTGAAGGGTGTTTCAGCCA
AACCTTTTATTGAGACCGTTCCCTCCATAGATGCATTGCACTGCGACATTGGCAATGCAACAGAATTCTACAGGATTTTC
CAGATGGAGATTGGTGAACTTTACAAGAATCCTGACGTGTCTAAAGAGGAGAGGAAGAGGTGGCAGCTGACTCTTGACAA
ACACCTCAGGAAGAAGATGAACTTGAAGCCTATGTTGAAGATGAGTGGAAATTTTGCTAGAAAGCTCATGTCCAAAGAGA
CCGTAGAGGCAGTATGTGAATTAATAAAGTGTGAGGAAAGGCATGAAGCCCTAAAAGAACTAATGGACCTTTATCTGAAG
ATGAAGCCAGTGTGGCGATCCTCATGCCCTGCCAAGGAGTGCCCAGAACTGCTGTGCCAGTATAGCTACAATTCACAGCG
TTTTGCRGAGCTCTTATCTACAAAGTTCAAATACAGATATGAGGGCAAGATTACAAATTATTTCCACAAAACACTTGCTC
ATGTTCCAGAAATCATTGAAAGAGATGGGTCCATTGGGGCCTGGGCAAGTGAAGGAAATGAGTCTGGAAACAAACTGTTC
AGGAGGTTCCGAAAAATGAACGCCAGGCAGTCCAAAGTCTATGAGATGGAGGATGTCTTG
Paradisaea_raggiana   TAAATTGCGATCATTTGAAAAAACACCCT-------------------------
----
CTAATGACAGCCAGCACATAAACAAAGATCTGGCAGAAGAGGCTGTTTCTTCAAACAAAGAATTCATCCTGCATAAAGAT
GAAGCAGTGCCAAGAGGAGAAAAGATGCAGTTAACGGGCAATAGGCAGGGACTTGAGGAAGATGCCCATGCCATGCAAAC
ACAAGACAATAGAGCTCATCAAAACAATTTGAAGGAACTCTGCCGCATCTGTGGAGTTTCATTTAAAACTGATTGTTCCA
AGAGGACTTACCCAGTGCATGGGCCAGTGGATGATGAAACTCTGTGGCTTCTGAGAAAGAAAGAAAAAACAGCAACCTCT
TGGCCAGACCTTATTGCTAAGGTTTTCAAGATTGATGTGCGAGGGGATGTTGATACTATCCATCCCACTCAATTCTGTCA
CAACTGTTGGAGTATTATACATAGAAAATTCAGTAATACTCTATGTGAAGTATATTTTCCTAGGAACAGCACAATGGAGT
GGCAACCGCACTCCCCAAACTGTGACGTGTGCCATACTACCAGACGAGGAGTTAAGAGAAAAAGCCAGCCCCGAAGTGTA
CAACGTGGCAAACGTGTCAAAACCACTGGAGAACGTGCTCGGCTAAACAGAGGTGTAAAGAAC------------
CAAGCACAAATAAACAACAAAAATTTAATGAAGGAGATTGTCAATTGCAAGGATATACATCTCAGCACCAAGCTGCTTGC
AGTTGATTACCCAGTAGATTTCATTAAATCCATTTCTTGCCAGATTTGCGATCATATTTTGGCAGATCCAGTGGAAACAA
CATGCAGACACTTGTTTTGCAGAACTTGCATCCTTAAATGTGTCAGGGTTATGGGCAGCTATTGCCCCTCCTGCTGGTAT
CCTTGCTTTCCTACTGATCTGGTAACCCCAGTGAAATCCTTCCTGAACATCCTTGATAACCTGAGTATAAGATGCCCTGT
AAAGGAATGTGATGAAGAGATCTTGCATGGAAAATATGGCCAACACCTCTCCAGCCACAAGGAGATGAAAGATAGAGAGC
TCTATAGCTACATAAATAAAGGTGGCCGACCGAGGCAGCACCTCCTGTCTTTGACGAGGAGAGCTCAGAAACATCGTCTG
AGGGAGCTGAAACGTCAAGTCAAGGCTTTTGCTGAGAAAGAAGAGGGTGGYGATATAAAGGCTGTATGCATGACTTTGTT
CCTGCTAGCTTTGAGAGCAAAAAATGAACACAAACAAGCAGATGAACTGGAGGCTATAATGCAAGGGAGGGGATCTGGAC
TTCATCCTGCTGTCTGTCTGGCCATCCGAATCAACACTTTTCTCAGCTGTAGTCAGTATCATAAAATGTATAGAACAGTA
AAAGCTGTCAGTGGGAGGCAGATCTTCCAGCCTTTGCATGCTCTTCGCACTGCTGAGAAAGCCCTCCTACCAGGTTATCA
CCCATTTGAGTGGAAACCTCCCTTGAAAAATGTATCCACCAACACAGAAGTGGGAATTATAGATGGACTATCAGGACTGC
CACTCTCAATTGATGACTACCCAGTAGACACAATTGCAAAGAGATTTCGATATGATGCAGCCTTGGTTTGTGCCTTAAAG
GACATGGAGGAGGAGATCTTGGAAGGCATGAAAGCAAAAAACCTGGATGACTACTTGAACGGCCCCTTCACTGTGGTAGT
GAAAGAGTCCTGTGATGGAATGGGAGATGTCAGTGAGAAGCATGGAAGTGGGCCTGCTGTCCCAGAGAAGGCTGTTCGCT
TTTCTTTCACAGTCATGAACATTTCTATAGCACAGGGGAATGAAAGCAAGAGGATCTTTGAGGAAGTAAAGCCCAATTCA
GAGTTGTGCTGCAAGCCCTTGTGCCTTATGCTGGCTGACGAATCAGATCATGAAACTCTGACGGCAATCCTGAGCCCCCT
CATAGCAGAAAGAGAGGCTATGAAAAACAGTGAACTGCTGCTTGAAATGGGAGGCATCCTGAGAACATTTAGATTCATCT
TTAGGGGTACAGGATATGATGAGAAACTCGTGCGGGAAGTGGAAGGGCTGGAGGCCTCAGGTTCCACTTACATTTGTACC
CTGTGCGATGCAACCCGCTTGGAGGCGTCCCAGAATCTGGTCTTCCACTCCATAACCAGGAGCCATGCTGAAAATCTGGA
GCGATATGAAATATGGAGGTCCAAYCCATATCACGAATCTGTTGATGAGCTCCGTGACAGAGTGAAGGGTGTTTCAGCCA
AACCTTTTATTGAGACCGTTCCCTCCATAGATGCATTGCACTGCGACATTGGCAATGCAACAGAATTCTACAGGATTTTC
CAGATGGAGATTGGTGAACTTTACAAGAATCCTGACGTGTCTAAAGAGGAGAGGAAGAGGTGGCAGCTGACTCTTGACAA
ACACCTCAGGAAGAAGATGAACTTGAAGCCTATGTTGAAGATGAGTGGAAATTTTGCTAGAAAGCTCATGTCCAAAGAGA
CCGTAGAGGCAGTATGTGAATTAATAAAGTGTGAGGAAAGGCATGAAGCCCTAAAAGAACTAATGGACCTTTATCTGAAG
ATGAAGCCAGTGTGGCGATCCTCATGCCCTGCCAAGGAGTGCCCAGAACTGCTGTGCCAGTATAGCTACAATTCACAGCG
TTTTGCRGAGCTCTTATCTACAAAGTTCAAATACAGATATGAGGGCAAGATTACAAATTATTTCCACAAAACACTTGCTC
ATGTTCCAGAAATCATTGAAAGAGATGGGTCCATTGGGGCCTGGGCAAGTGAAGGAAATGAGTCTGGAAACAAACTGTTC
AGGAGGTTCCGAAAAATGAACGCCAGGCAGTCCAAAGTCTATGAGATGGAGGATGTCTTG
Cyanocorax_chrysops
??

??
??
??
??
??
??
??
??
??
??
??
??
??
??
??
??
??
??
??
??
??
??
??
??
??
??
??
??
??
??
??
??
??
??
??
??
??
Lonchura
??
??
??
??
??
??
??
??
??
??
??
??
??
??
??
??
??
???TAGTCAGTATCATAAAATGTATAGAAC
AGTGAAAGCTGTCACTGGGAGGCAGATCTTCCAGCCTTTGCATGCTCTTCGCACTGCTGAGAAAGCCCTCCTCCCAGGTT
ATCACCCATTTGAGTGGAAACCTCCCCTGAAAAATGTATCCACTAACACAGAAGTGGGAATTATAGATGGACTATCAGGA
CTGCCGCTCTCAATTGATGATTACCCAGTAGACACAATTGCAAAGAGATTCCGATATGATGCAGCCTTGGTTTGTGCCTT
AAAGGACATGGAGGAGGAGATCTTGGAAGGCCTGAAAGCAAAAAAACCTGGACGACTATTTGAATGGCCCCTTCACTGTGG
TAGTAAAAGAGTCCTGTGATGGAATGGGAGATGTCAGTGAGAAGCATGGAAGTGGGCCTGCTGTCCCAGAGAAGGCTGTT

CGCTTTTCTTTCACAGTCATGAACATTTCTATAGAACATGGGAATGAAAGCAAGAGGATTTTTGAGGAAGTAAAGCCCAA
TTCAGAATTGTGCTGTAAGCCCTTGTGCCTTATGCTGGCTGATGAATCAGATCATGAAACTCTGACAGCAA?????????
??
??
??
??
??
??
??
??
??
??
??
??
Phylloscopus TAAATTGCGATCATTTGAAACAACACCCT-------------------------
----
CTGATGACAGCCGGCACATAAACAAAGATCAGGCAGAAGAGGCTGTTTCTTCAAACAAAGAATTCATCCTGCATAAAGAT
GAAGCAGTGCCAGGAGGAGAAAAGATGGAGTTGACRGGCAATAGGCAGAGACTGGAGGAAGATGCCCATGCCATGAAAAC
RCAAGACAACAGAGCTCATCAGAACAATCTGAAGCAACTCTGCCGCATCTGTGGAGTTTCATTCAAAACTGATTGTTACA
AGAGAACTTACCCAGTGCAYGGGCCAGTGGATGACGAAACTCTGTGGCTTCTGAGAAAGAAAGAAAAAACAGCAACCTCT
TGGCCAGATCTTATTGCTAAAGTTTTCAAGATTGATGTGCGAGGGGATGTCGATACTATCCATCCCACTCGATTTTGTCA
CAACTGTTGGAGTATTATACAGAGAAAATTCAGTAATACTCCATGTGAAGTATATTTTCCTAGGAACAGCACAATGGAGT
GGCAACCCCATTCCCCAAACTGTGATGTGTGCCATACTACCAGACGAGGAGTCAAGAGAAAAAAACAGCCCCCAAGTGTA
CAACGTGGTAAACGTGTCAAAACCACTGGGGAACGTGCTCGGCTAAACAGAGGTGTAAAGAAC------------
CAAGCACAGATAAACAACAAAAATTTAATTAAAGAGATTGTCAGTTGCAAGGATATACATCTCAGCACCAAGCTGCTTGC
AATTGACTACCCAGTAGATTTCATTAAATCCATCTCTTGCCAGATTTGTGATCATATTTTGGCAGATCCAGTGGAAACAA
CATGCAGACACTTGTTTTGCAGAACTTGCATCCTTAAATGTATCAGGGTTATGGGCAGCTATTGCCCCTCCTGCTGGTAT
CCTTGCTTTCCTACTGATCTGGTTACCCCAGTGAAATCCTTCCAGAACATCCTTGATAACTTGAGGATAAGATGCCCTGT
AAAGGAATGTGGTGAAGAGATCTTGCATGGAAAATATGGCCAACACCTCTCAGGTCACAAGGAGATGAAAGATAGAGAGC
TCCATAGCTACATAAATAAAGGTGGCCGACCAAGGCAGCACCTCCTGTCCTTAACTAGGAGAGCTCAGAAACATCGCCTG
AGGGAACTGAAACGTCAAGTCAAGGCTTTTGCTGAGAAAGAAGAGGGTGGTGATATAAAGGCTGTATGCATGACTTTGTT
TCTACTAGCTTTGAGAGCAAAAAATGAACACAAACAAGCAGATGAACTGGAGGCTATAATGCAGGGGAGGGGATCTGGAC
TTCATCCTGCTGTCTGTCTGGCCATCCGAATCAACACGTTTCTCAGCTGTAGTCAGTATCATAAAATGTACAGAACAGTA
AAAGCTGTCACTGGGAGGCARATCTTTCAGCCTTTGCATTCTCTTCGCACTGCTGAGAAAGCCCTCCTACCAGGTTATCA
CCCGTTCGAGTGGAGACCTCCCCTGAAAAATGTATCCACTAACACAGAAGTGGGAATTATAGATGGACTATCAGGACTGC
CACTCTCAATTGATGATTACCCAGTAGACACAATTGCAAAGAGATTCCGATATGATGCAGCCCTGGTTTGTGCCTTAAAA
GACATGGAGGAGGAGATCTTGGAAGGCATGAAGGAAAAAAACCTGGATGACTATTTGAATGGTCCCTTCACTGTGGTAGT
AAAAGAGTCCTGTGATGGAATGGGAGATGTCAGTGAGAAGCATGGAAGTGGGCCTGCTGTCCCAGAGAAGGCTGTTCGCT
TTTCCTTCACAGTCATGAACATTTCTATAGCACATGGGAATGAAAGCAAGAGGATTTTTGAGGAAGTAAAGCCCAATTCA
GAGTTGTGCTGTAAGCCCTTGTGCCTTATGCTGGCTGATGAATCAGATCATGAAACTCTGACAGCAATCCTGAGTCCCCT
CATAGCAGAAAGAGAGGCTATGAAAAACAGTGAACTGCTGCTTGAAATGGGAGGCATCCTGAGAACATTCAGATTTGTCT
TTAGGGGTACAGGATATGATGAGAAACTGGTGCGGGAAGTGGAAGGGCTGGAGGCCTCAGGTTCCACTTACATTTGTACC
CTGTGTGATGCAACCCGCTTGGAGGCATCCCAGAATCTGGTCTTTCACTCCATAACCAGGAGCCATGCTGAAAATCTGGA
GCGATATGAAATATGGAGGTCCAACCCATATCACGAGTCTGTTGATGAGCTCCGTGACAGAGTGAAGGGTGTTTCAGCCA
AGCCTTTTATTGAGACTGTTCCCTCCATAGATGCATTGCACTGCGACATTGGCAATGCCACAGAATTCTACAGGATTTTC
CAGATGGAGATTGGTGAACTTTACAAGAATCCTGACGTGTCTAAAGAGGAGAGGAAGAGGTGGCAGTTGGCTCTTGACAA
ACACCTCAGGAAGAAGATGAACTTGAAGCCTATGTTGAAGATGAGTGGAAATTTTGCTAGAAAGCTCATGTCCAAAGAGA
CTGTAGAGGCAGTATGTGAATTAATAAAGTGTGAGGAAAGGCATGAAGCCCTGAAAGAACTAATGGACCTTTATCTGAAG
ATGAAGCCAGTGTGGCGATCCTCATGCCCTGCYAAGGAGTGCCCAGAACTGCTGTGCCAGTATAGCTACAATTCACAGCG
TTTTGCAGAACTCTTATCTACAAAGTTCAAGTATAGATATGAGGGCAAGATTACAAATTATTTCCACAAAACACTTGCTC
ATGTTCCTGAAATCATTGAAAGAGATGGGTCCATYGGGGCCTGGGCAAGTGAAGGAAATGAGTCTGGAAACAAACTGTTT
AGGAGGTTCCGAAAAATGAATGCCAGGCAGTCCAAAGTCTATGAGATGGAAGATGTCTTG
Turdus TAAATTGCGATCATTTGAAAAAACACCCT-------------------------
----
CTGATGATGGCCAGCACATAAACAAAGATCAGGCAGAAGAGGCTGTTTCTTCAAACAAAGAATTCATTCTGCATGAAGAT
GAAGCAGTGCCAAGTGGAGAAAAGATGGAGCTAACAGGCAATAGGCAGGGACTTGAGGAAGATGCCCATGCCATGAAAAC
ACAAGACAATAGAGCTCATCAGAACAATCTGAAGCAACTCTGCCGCATCTGTGGAGTTTCATTTAAAACAGATTGTTACA
AGAAAACTTACCCAGTGCATGGGCCAGTGGATGATGAAACTCTGAAGCTTCTGAGAAAGAAAGAAAAAACAGCAACCTCT
TGGCCAGACCTTATTGCTAAGGTTTTCAGGATTGATGTGCGAGGGGATGTCGATACTATCCATCCCACCCACTTTTGTCA
CAACTGTTGGAGTATTATACACAACAAATTCAGTAATACTCTGTGTGAAGTATATTTTCCTAGGAACAGCACAATGCAGT

GGCAACCCCATTCCCCAAACTGTGATGTGTGCCATACTACCAGAAGAGGAGTCAAGAGAAAAAGCCAGCCCCCAAGTGTA
GCGCGTGGCAAACGTGTCAAAACCACTGGGGAACATGCTCAGCTAAACAGAGGTGCAAAGAAC------------
CAAGCACAGATAAACAACAAAAATTTAATGAAAGAGATTGTCAATTGCAAGGATATACACCTCAGCACCAAGCTGCTTGC
AGTTGATTACCCACCGGATTTCATTAAATCCATTTCTTGCCAGATTTGTGATCATATTTTGGCAGATCCAGTGGAAACAA
CATGCAGACACTTGTTTTGCAGAACTTGCATCCTTAAATGTATCAGGGTTATGGGCAGCTATTGCCCCTCCTGCTGGTAT
CCTTGCTTTCCCACTGACCTGGTAACCCCAGTAAAATCCTTCTTGAACATCCTTGATAACCTGAGTATAAGATGCCCTGT
AAAGGAATGTGATGAAGAGATCTTGCATGGAAAGTATGGCCAACACCTCTCTGGCCACAAGGAGATAAAAGACAGAGAGC
TCCACAGCTACATAAATAAAGGTGGCCGACCGAGGCAGCACCTCCTGTCCCTGACGAGGAGAGCTCAGAAACATCGTCTG
AGGGAGCTGAAACGCCAGGTCAAGGCTTTTGCTGAGAAAGAAGAGGGCGGTGACATAAAGGCTGTATGCATGACTTTGTT
CCTGCTAGCTTTGAGAGCAAAAAATGAACACAAACAAGCAGATGAACTGGAGGCTATAATGCAAGGGAAGGGATCTGGAC
TTCATCCTGCTGTCTGTCTGGCCATCCGAATCAACACGTTTCTCAGCTGTAGTCAGTATCACAAAATGTACAGAACAGTA
AAAGCTGTCACAGGAAGGCAGATCTTCCAGCCTTTGCATGCTCTTCGCACTGCTGAGAAAGCCCTCCTACCAGGTTATCA
TCCATTTGAGTGGAATCCTCCCCTGAAAAATGTATCCACTAACACAGAAGTGGGAATTATAGATGGACTATCAGGACTGC
CACTCTCAATTGATGATTACCCAGTAGACACAATTGCAAAGAGATTCCGATATGATGCAGCCTTGGTTTGTGCCTTAAAG
GACATGGAGGAGGAGATCTTGGAAGGCATGAAAGCAAAAAACCTGGATGACTATTTGACTGGCCCCTTTACTGTGGTAAT
AAAAGAGTCCTGTGATGGAATGGGAGATGTCAGTGAGAAGCATGGAAGTGGGCCTGCTGTCCCAGAGAAGGCTGTTCGCT
TTTCTTTCACAGTCATGAACATTTCTATAGCACATGGGAATGAAAACAAGAGGATCTTTGAGGAATTAAAGCCCAATTCA
GAGTTGTGCTGTAAGCCCTTATGCCTTATGCTGGCTGATGAATCAGACCATGAAACTCTGACAGCAATCCTGAGCCCCCT
CATAGCAGAAAGAGAGGCTATGAAAAACAGTGAACTGCTGCTTGAGATGGGAGGCATCCTGAGAACATTTAGATTCGTCT
TTAGGGGTACAGGATATGATGAGAAACTCGTGAGGCAAGTGGAAGGGCTGGAGGCCTCAGGGTCCACTTACATTTGTACC
CTGTGTGATGCCACCCGCTTGGAGGCATCCCAGAACCTGGTCTTGCACTCCATAACCCGGAGCCATGCTGAAAACCTGGC
ACGATATGAAATATGGAGGTCCAACCCATATCACGAGTCAGTCGATGAGCTCCGTGACAGAGTGAAGGGTGTTTCAGCCA
AACCTTTTATCGAGACTGTCCCCTCCATAGATGCATTGCATTGTGACATTGGCAATGCAACAGAGTTTTACAGGATCTTC
CAGATGGAGATCGGTGAGCTTTACAAGAATCCTGATGTGTCTAAGGAGGAGAGGAAGAGGTGGCAGCTGACTCTGGACAA
ACATCTCAGGAAGAAGATGAACTTGAAGCCTATGTTGAAGATGAGTGGAAATTTTGCTAGAAAGCTCATGTCCAAAGAGA
CTGTAGAGGCAGTATGTGAATTAATAACATGTGAGGAAAGGCATGAAGCCCTAAAAGAACTAATGGACCTTTATCTGAAG
ATGAAGCCAGTGTGGCGATCCTCATGCCCTTCCAAGGAGTGCCCAGAACTGCTGTGCCAATATAGCTACAATTCACAGCG
TTTTGCAGAGCTCTTATCTACAAAGTTCAAGTACAGATATGAGGGCAAGATTACAAATTATTTCCACAAAACACTTGCTC
ATGTTCCTGAAATCATTGAAAGAGATGGGTCTATTGGGGCCTGGGCAAGTGAAGGAAATGAGTCTGGAAACAAACTGTTT
AGGAGGTTCCGAAAAATGAATGCCAGGCAGTCCAAAGTCTATGAGATGGAGGATGTCTTG
Acrocephalus ????????????????????????CCCT------------------------
----
CTGATGACAGCCAGCACATAAACAAAGATCAGGCAGAAGAGGCTGTTTCTTCAAACAAAGAATTCATCCTGCATAAAGAT
GAAGCAGAGCCAAAAGGCGAAAAGATGGAGTTAACGGGCAATAGGCAGGGACTTGAGGAAGATGCCCATGCCATGAAAAC
ACAAGACAATAGAGCTCATCAGAACAATCTGAAGCAACTCTGCCGCATCTGTGGAGTTTCATTTAAAACTGATTGTTACA
AGAAAACTTATCCAGTGCATGGGCCAGTGGATGATGAAACTCTGTGCCTTCTGAGAAAGAAAGAAAAAACAGCAACCTCT
TGGCCAGATCTTATTGCTAAGGTTTTCAAGATTGATGTGCGAGGGGATGTTGATACTATCCATCCCACTCGATTTTGTCA
TAACTGTTGGAGTATTATACACAGAAAATTCAGTAATACTCTATGCGAAGTATATTTTCCTAGGAACAGCACAATGCAGT
GGCAACCCCATTCCCCAAACTGTGATGTGTGCCATACTACCAGACGAGGAGTCAAGAGAAAAAACCATCCCCCAAGTGTA
CAACGTGGCAAACGTGTCAAAACCACTGGGGAACGTCCTTGGCTAAACAGAGGTGTAAAGAAC------------
CAAGCACATATAAACAACAAAAATTTAATTAAAGAGATTGTCAATTGCAAGGATATACATCTCAGCACCAAGCTTCTTGC
AATTGATTACCCAGTAGATTTCATTAAATCCATTTCTTGCCAGATTTGTGATCATATTTTGGCAGATCCAGTGGAAACAA
CATGCAGACACTTATTTTGCAGAACTTGCATCCTTAAATGTATCAGGGTTATGGGCAGCTATTGCCCCTCCTGCTGGTAT
CCTTGCTTTCCTACTGATCTGGTTACCCCAGTGAAATCCTTCCTAAACATCCTTGATAACTTGAGTATAAAATGCCCTGT
AAAGGAATGTGATGAAGAGATCTTGCATGGAAAATACGGCCAACACCTCTCCAGCCACAAGGAGATGAAGGATGGAGAAC
TCTACAGCTACATAAATAAAGGTGGCCGACCGAGGCAGCACCTCCTGTCTTTGACGAGGAGAGCTCAGAAACATCGTCTG
AGGGAACTGAAACGTCAAGTCAAGGCTTTTGCTGAGAAAGAAGAGGGTGGTGATATAAAGGCTGTATGCATGACTTTGTT
CCTGCTAGCTTTGAGAGCAAAAAATGAACACAAACAAGCAGATGAACTGGAGGCTATAATGCAAGGGAAGGGATCTGGAC
TTCATCCCGCTGTCTGTCTGGCCATCCGAATCAACACATTTCTCAGCTGTAGTCAGTATCATAAAATGTATAGAACAGTA
AAAGCTGTCACTGGGAGGCAGATCTTCCAGCCTTTGCATTCTCTTCGCACTGCTGAGAAAGCCCTCCTACCAGGTTATCA
CCCATTTGAGTGGAAACCTCCCCTGAAAAATGTATCCACTAACACAGAAGTGGGAATTATAGATGGACTATCAGGACTGC
CACTCTCCATTGATGATTACCCAGTAGACACAATTGCAAAGAGATTCCGATATGATGCAGCCCTGGTTTGTGCCTTAAAA
GACATGGAGGAGGAGATCTTGGAAGGCATGAAAGAAAAAAACCTGGACGACTATTTGAATGGSCCCTTCACCGTGGTAGT
AAAAGAGTCCTGTGACGGAATGGGAGATGTCAGTGAGAAGCATGGAAGTGGGCCTGCTGTCCCAGAGAAGGCCGTTCGCT
TTTCCTTCACAGTCATGAACATTTCTATAGCACATGGGAATGAAAGCAAGAGGATCTTTGAGGAAGTAAAGCCTAATTCA
GAGTTGTGCTGTAAGCCCTTGTGCCTTATGCTGGCTGATGAATCAGATCATGAAACTTTGACGGCAATCCTGAGCCCCCT
CATAGCAGAAAGAGAGGCTATGAAAAACAGTGAACTGCTGCTTGAAATGGGAGGCATCCTGAGAACATTTAGATTTGTCT
TTAGGGGTACAGGGTATGATGAGAAACTTGTGCGGGAAGTGGAAGGGCTGGAGGCCTCAGGTTCCACTTACATTTGTACC
CTGTGTGATGCCACCCGCTTGGAGGCATCCCAGAATCTGGTCTTCCACTCCATAACCAGGAGCCATGCTGAAAATCTGGA
GCGATATGAAATATGGAGGTCCAACCCATACCACGAGTCTGTTGATGAGCTCCGTGACAGAGTGAAGGGTGTTTCAGCCA

AACCTTTTATTGAGACTGTTCCCTCCATAGATGCATTGCACTGCGACATTGGCAATGCGACAGAATTCTACAGGATTTTC
CAGATGGAGATTGGTGAACTTTACAAGAATCCTGACGTGTCTAAAGAGGAGAGGAAGAGGTGGCAGTTGGCTCTTGACAA
ACACCTCAGGAAGAAGATGAACTTGAAGCCTATGTTGAAAATGAGTGGCAATTTTGCTAGAAAGCTTATGTCCAAAGAGA
CTGTAGAGGCAGTATGTGAATTAATAAAGTGTGAGGAAAGGCATGAAGCCCTAAAAGAACTAATGGACCTTTATCTGAAG
ATGAAGCCAGTGTGGCGATCCTCATGTCCTGCCAAAGAATGCCCAGAACTGCTGTGCCAGTATAGCTACAATTCACAGCG
TTTTGCAGAACTCTTATCTACAAAGTTCAAGTACAGATATGAGGGCAAGATTACAAATTATTTCCACAAAACACTTGCTC
ATGTTCCCGAGATCATTGAAAGAGATGGGTCCATTGGGGCCTGGGCAAGCGAAGGAAATGAGTCTGGAAACAAACTGTTT
AGGAGGTTCCGAAAAATGAATGCCAGGCAGTCCAAAGT??????????????????????
Sitta
??
??
??
??
??
??
??
??
??
??
??
??
??ATCAGATGCC
CTGTAAAGGAATGTGATGAAGAGATTTTGCATGGGAAATATGGCCAACACCTCTCTGGCCACAAGGAGATGAAAGATAGA
GAGCTCTGTAGCTACATAAATAAAGGAGGCCGGCCGAGGCAGCACCTGCTGTCTTTGACAAGGAGAGCTCAGAAACATCG
TCTGAGGGAACTGAAACGCCAAGTCAAGGCTTTTGCTGAGAAAGAAGAGGGTGGTGACATAAAGGCTGTATGCATGACTT
TGTTCCTGCTAGCTTTGAGAGCCAAAAATGAGCACAAACAAGCAGATGAACTGGAGGCTATAATGCAAGGGAGGGGATCT
GGACTTCACCCTGCTGTCTGCCTGGCCATCCGAATCAACACGTTCCTCAGCTGTAGTCAGTACCATAAAATGTACAGAAC
AGTGAAAGCTGTCACTGGGAGGCAGATCTTCCAGCCTTTGCATGCCCTCCGCACTGCTGAGAAAGCCCTCCTACCTGGTT
ATCACCCATTYGAGTGGAAACCCCCCCTGAAAAATGTATCCACTAACACAGAGGTGGGAATTATAGATGGGCTGTCAGGG
CTGCCACTCTCCATTGATGATTACCCAGTAGACACGATTGCAAAGAGATTCCGCTACGACGCAGCCTTGGTTTGTGCCTT
AAAGGACATGGAGGACGAGATCCTGGAAGGCATGAAAGCAAAAAACCTGGATGACTATTTGAATGGCCCCTTCACTGTGG
TAGTAAAAGAGTCCTGTGATGGGATGGGAGATGTCAGTGAGAAGCATGGAAGCGGGCCTGCTGTCCCAGAGAAGGCTGTT
CGCTTTTCTTTCACAGTCATGAACATTTCTGTAGCACATGGCAGCGAAAGCAAGAGGATCTTTGAGGAAGCAAAGCCCAA
TTCAGAGTTGTGCTGTAAGCCCTTGTGCCTTATGCTGGCT??
??
??
??
??
??
??
??
??
??
??
??
??
Mimus
??
??
??
??
??
??
??
??
??
??
??
??
??ATAAGATGCC

```
CTGTACAGGAATGTGATGAAGAGATCTTGCATGGAAAGTATGGCCAACACCTCTCTGGCCACAAGGAGATGAAAGATGGA
GAGCTCCATAGCTACATAAATAAAGGTGGCCGACCAAGGCAGCACCTCCTGTCCCTTACAAGGAGAGCTCAGAAACATCG
CCTGAGGGAACTGAAACGTCAAGTCAAGGCTTTTGCTGAGAAAGAAGAGGGTGGTGATATAAAGGCTGTATGCATGACTT
TGTTCCTGCTAGCTTTGAGAGCAAAAAATGAACACAAACAAGCAGATGAACTGGAGGCTATAATGCAAGGGAGGGGATCW
GGACTTCATCCTGCTGTCTGTCTGGCCATCCGAATCAACACGTTTCTCAGCTGTAGTCAGTATCATAAGATGTACAGAAC
AGTAAAAGCTGTCACTGGGAGGCAGATCTTCCAGCCTCTGCATGCTCTTCGCACTGCTGAGAAAGCCCTCCTACCAGGCT
ATCACCCATTTGAGTGGAATCCTCCCCTGAAAAACGTATCCACTAACACTGAAGTGGGAATTATAGATGGACTATCAGGA
CTGCCACTCTCAATTGATGATTACCCAGTAGACACAATTGCAAAGAGGTTCCGATATGATGCAGCCTTGGTTTGTGCCTT
AAAGGACATGGAGGAAGAGATCTTGGAAGGCATGAAAGCAAAAAACCTGGATGACTATTTGAATGGCCCTTTCACTGTGG
TAATAAAAGAGTCCTGTGATGGAATGGGAGATGTCAGTGAGAAGCATGGAAGTGGGCCTGCTGTCCCAGAGAAAGCTGTT
CGCTTTTCTTTCACAGTCATGAACATCTCTATAACACAAGGGAATGAAAGCAAGAGGATTTTTGAGGAAGTAAAGCCCAA
TTCAGAGTTGTGCTGTAAGCCCTTGTGCCTTATGCTGGCT????????????????????????????????????????
????????????????????????????????????????????????????????????????????????????????
????????????????????????????????????????????????????????????????????????????????
????????????????????????????????????????????????????????????????????????????????
????????????????????????????????????????????????????????????????????????????????
????????????????????????????????????????????????????????????????????????????????
????????????????????????????????????????????????????????????????????????????????
????????????????????????????????????????????????????????????????????????????????
????????????????????????????????????????????????????????????????????????????????
????????????????????????????????????????????????????????????????????????????????
????????????????????????????????????????????????????????????????????????????????
????????????????????????????????????????????????????????????????????????????????
????????????????????????????????????????????????????????????????
Sturnus_vulgaris          TAAATTGCCATCATTTGAAAAAACACCCT-------------------------
-----------------------------------
CTGATGACAGCCAGCACATAAACAAAGATCAGGCAGAAGAGGCTGTTTCTTCAAACGAAGAATTCATTCTGCATAAAGAT
GAAGCAGTGCCAAGAGGAGAAAAGATGGAGTCAACAAGCAATAGGCAGGCACTTGAGGAAGATGYCCATGCTGTGAAAAT
ACAAGACAATAGAGTTCATCAGAACAATCTGAAGCAACTCTGCCGCATCTGTGGAGTTTCATTTAAAACTGATTGTTACA
AGAAAACTTACCCAGTGCATGGGCCAGTGGATGATGAAACTCTGAGGCTTCTGAGAAAGAAAGAAAAAACAGCAACCTCT
TGGCCAGACCTTATTGCTAAGGTTTTCAAGATTGATGTGCGAGGGGATGTTGATACAATCCATCCCACTCAATTCTGTCA
CAACTGTTGGAGTATTATACATAGCAAATTCAGTAATACTCCATGTGAAGTATATTTTCCTAGGAACAGCACAATGGAGT
GGAAACCCCATTCCCCAAACTGTGATGTGTGCCATACTACCAGAARAGGAGTCAAGAGAAAAAGCCAGCCTCCAAATGTA
CAACGTGGCAAACGTGCCAAAACCACCAGGGAACATGCTCAGCTAAACAGAGGTGTAAAGAAC------------
CAAGCACAGATAAACAACAAAAATTTAATGAAAGAGATTGTCAATTGCAAGGATATACATCTCAGCACCAAGCTGCTGGC
GGTTGATTACCCACTAGATTTCATTAAATCCATTTCTTGCCAGATTTGTGATCATATTTTGGCAGATCCGGTGGAAACAA
CATGCAGACACTTGTTTTGCAGAACTTGCATCCTTAAATGTATCAGGGTTATGGGCAGCTATTGCCCCTCCTGCTGGTAT
CCTTGCTTTCCTACTGATCTGGTAACCCCAGTGAAATCCTTCCTGAACATCCTYGATAACCTGAGTATAAGATGCCCTGT
AAAGGAATGTGATGAAGAGATCTTGCATGGAAAGTATGGCCAACACCTCTCTGGCCACAAGGAGATGAAAGAGGGAGAGC
TCCACAGCTACATAAATAAAGGTGGCCGACCAAGGCAGCACCTCCTGTCTCTGACGAGGAGAGCTCAGAAACATCGTCTG
AGGGAACTGAAACGTCAAGTCAAGGCTTTTGCTGAGAAGGAAGAGGGCGGTGATATAAAGGCTGTATGCATGACTTTGTT
CCTGCTGGCTCTGAGAGCAAAAAATGAACACAAACAAGCAGATGAACTGGAGGCTATAATGCAAGGGAGGGGATCTGGAC
TTCATCCCGCTGTCTGTCTGGCCATCCGAATCAACACGTTTCTCAGCTGCAGTCAGTATCATAAAATGTACAGAACAGTA
AAAGCTGTCACTGGCAGGCAGATCTTCCAGCCTTTGCATGCCCTTCGCACTGCTGAGAAAGCCCTCCTACCAGGCTATCA
CCCATTTGAGTGGAATCCTCCCCTGAAAAACGTATCCACTAACACAGAAGTGGGAATTATAGATGGACTATCAGGACTGC
CCCTCTCAGTTGATGATTACCCAGTAGACACAATTGCAAAGAGATTCCGATATGATGCAGCCTTGGTTTGTGCCTTAAAG
GACATGGAGGAGGAGATCTTGGAAGGTATGAAAGCAAAAAACCTGGACGACTATTTGAATGGCCCCTTCACTGTGGTAAT
AAAAGAGTCCTGTGATGGAATGGGAGATGTCAGTGAGAAGCATGGAAGTGGGCCTGCTGTCCCAGAGAAGGCTGTTCGCT
TTTCTTTCACAGTCATGAACATCTCTATAACACATGGGAACGAAAGCAAGAGGATCTTTGAGGAAGTAAAGCCCAATTCA
GAGTTGTGCTGTAAGCCCTTGTGCCTTATGCTGGCTGATGAATCAGATCACGAAACTCTGACAGCAATCCTGAGCCCCCT
CATAGCAGAAAGAGAGGCTATGAAAAACAGTGAACTGCTGCTTGAAATGGGAGGCATCCTGAGAACATTTAGATTCGTCT
TTAGGGGTACAGGATATGATGAGAAACTCGTGCGGGAAGTGGAAGGGCTGGAGGCCTCAGGTTCCACTTACATTTGTACC
CTGTGTGATGCCACCCGCTTGGAGGCATCCCAGAACCTGGTCTTCCACTCCATAACCAGGAGCCACGCTGAAAATCTGGA
GCGGTATGAAATATGGAGGTCCAACCCCTACCACGAGTCTGTGGATGAGCTCCGTCACAGAGTGAAGGGTGTTTCAGCCA
AACCTTTTATTGAGACTGTTCCCTCCATAGATGCATTGCACTGTGACATTGGCAATGCAACAGAATTCTACAGGATTTTC
CAGATGGAGATTGGGGAACTTTACAAGAATCCTGACGTGTCTAAGGAGGAGAGGAAGAGGTGGCAGTTGACTCTTGACAA
ACACCTCAGGAAGAAGATGAACTTGAAGCCTATGTTGAAGATGAGYGGAAATTTTGCTAGAAAGCTCATGTCCAAAGAGA
CTGTAGAGGCAGTATGTGAATTAATAACATGTGAGGAAAGGCATGAAGCTCTAAAAGAACTAATGGACCTTTATCTGAAG
ATGAAGCCAGTGTGGCGATCCTCRTGCCCTGCCAAGGAGTGCCCAGAACTGCTGTGCCAGTATAGCTACAATTCACAGCG
TTTTGCAGAGCTCTTATCTACAAAGTTCAAGTACAGATATGAGGGCAAGATTACAAATTATTTCCACAAAACACTTGCTC
```

ATGTCCCCGAAATCATTGAAAGAGATGGGTCCATTGGGGCCTGGGCAAGCGAAGGAAACGAGTCTGGAAACAAACTGTTC
AGGAGGTTCCGAAAAATGAATGCCAGGCAGTCCAAAGTCTATGAGCTGGAGGATGTCTTG
Creadion_carunculatus TAAATTGCGATCATTTGAAAAAACACCCT-------------------------
----
CTGATGACAGCCAGCACATAAACAAAGACCAGGCAGAAGAGGCTGTTTCTTCAAACGAAGAATTCATCCTCCATAAAGAT
GAAGCAGTGCCAGGAGGAGAAAAGATGGAGTTAACGGGCAATAGGCAGGGACTTGAGGAACATGCCCATGCCATGAAAAC
ACAAGACAATAGAGCTCATCAGAACAATTTGAAGCAACTCTGCCGCATCTGTGGAGTTTTATTTAAAACTGATGGTTACA
ATAGAACTTACCCAGTGCATGGGCCAGTGGATGATGAAACTCTGTGTCTTCTGAGAAAGAAAGAAAAAACAGCAACCTCT
TGGCCAGATCTTATTGCTAAGGTTTTTAAGATTGATGTGCGAGGGGATGTTGATACTATCCATCCCACTCGATTTTGTCA
TAACTGTTGGAGTATTATAAATAGAAAATTCAGTAATACTCTATGTGAAGTATATTTTCCTAGGAACAGCACAATGGAGT
GGCAACCCCACTCCCCAAACTGTGATGTCTGCCATACTACCAGACGAGGAGTCAAGAGAAAAAGCCAGCCCCCAAGTGTA
CAACGTGGCAAACGTGTCAAAACCACTGGGGAACGCGCTCGGCTAAACAGAGGTATAAAGAAC------------
CAAGCACAGATAAACAATAAAAATTTAATGAAAGAGATTGTCAATTGCAAGGATATACATCTCGGCACCAAGCTGCTTGC
CGTTGATTACCCGGTAGATTTCATTAAATCCATTTCTTGCCAGATTTGTGATCATATTTTGGCAGATCCAGTGGAAACAA
CATGCAGACACTTGTTTTGCAGAACTTGCATCCTTAAAGGTATCAGGGTTATGGGCAGCTATTGCCCCTCCTGCTGGTAT
CCTTGCTTTCCTACTGATCTGGTAACCCCAGTGAAATCCTTCCTGAACATCCTCGATAACCTGAGTATAAGATGCCCTGT
AAAGGAATGTGATGAAGAGATCTTGCATGGAAAATATGGCCAACACCTCTCCAGCCACAAKGAGATGAAAGATAGAGAGC
TCTACAGCTACATAAATAAAGGTGGCCGACCGAGGCAGCACCTCCTGTCTTTGACGAGGAGAGCTCAGAAACATCGTCTG
AGGGAACTGAAACGTCAAGTCAAGGCTTTTGCTGAGAAAGAAGAGGGCGGTGATATAAAAGCTGTATGCATGACTTTGTT
CCTGCTAGCTTTGAGAGCAAAAAATGAACACAAACAAGCAGATGAACTGGAGGCTATAATGCAAGGGAGGGGATCTGGAC
TTCATCCTGCTGTCTGTCTGGCCATCCGAATCAACACATTTCTCAGCTGTAGTCAGTATCATAAAATGTATAGAACAGTA
AAAGCTGTCACTGGGAGGCAGATCTTCCAGCCTTTGCATGCTCTTCGCACTGCTGAGAAAGCCCTTCTACCAGGTTATCA
CCCATTTGAGTGGAAACCTCCCTTGAAAAATGTATCCACTAACACAGAAGTGGGAATTATAGATGGACTATCAGGACTGC
CACTCTCAATTGATGACTACCCAGTAGACACAATTGCAAAGAGATTTCGATATGATGCGGCCTTGGTTTGTGCCTTAAAG
GACATGGAGGAGGAGATCTTGGAAGGCATGAAAGCAAAAAACCTGGATGACTATTTGAATGGCCCCTTCACTGTGGTAGT
AAAAGAGTCCTGTGATGGAATGGGAGATGTCAGTGAGAAGCATGGAAGTGGGCCTGCAGTCCCAGAGAAGGCTGTTCGCT
TTTCTTTCACAGTCATGAACATTTCTATAGCACATGGGAATGAAAGCAAGAGGATCTTTGAGGAAGTAAAGCCCAATTCA
GAGTTGTGCTGTAAGCCCTTGTGCCTTATGCTGGCTGACGAATCTGATCACGAAACTCTGACAGCAATCCTGAGCCCCCT
CATAGCAGAGCGAGAGGCTATGAAAAACAGTGAACTGCTGCTTGAAATGGGAGGTATCTTGAGAACATTTAGATTCATCT
TTAGGGGTACAGGGTATGATGAGAAACTCCTGAGGGAAGTGGAAGGGCTGGAGGCCTCAGGTTCCACTTACATTTGTACC
CTGTGTGATGCAACCCGCTTGGAGGCATCCCAGAATCTCGTCTTCCACTCCATAACCAGGAGCCACGCTGAAAATCTGGA
GCGATATGAAATATGGAGGTCCAACCCATATCACGAATCTGTTGATGAGCTCCGTGACAGAGTGAAGGGTGTTTCAGCCA
AACCTTTCATTGAGACTGTTCCCTCCATAGATGCATTGCACTGCGACATTGGCAATGCAACAGAATTCTACAGGATTTTC
CAGATGGAGATTGGTGAACTTTATAAGAATCCTGATGTGTCTAAAGAGGAGAGGAAGAGGTGGCAGTTGACTCTTGACAA
ACACCTCAGGAAGAAGATGAACTTGAAGCCTATGTTGAAGATGAGTGGAAATTTTGCTAGAAAGCTCATGTCCAAAGAGA
CTGTAGAGGCAGTATGTGAATTAATAAAGTGTGAGGAAAGGCATGAAGCCCTAAAAGAACTAATGGACCTTTATCTGAAG
ATGAAGCCAGTGTGGCGATCCTCATGCCCTGCCAAGGAGTGCCCAGAACTGTTGTGCCAGTATAGCTACAATTCACAGCG
TTTTGCGGAGCTCTTATCTACAAAGTTCAAGTACAGATATGAAGGCAAGATTACAAATTATTTCCACAAAACACTTGCCC
ATGTTCCTGAAATCATTGAAAGAGATGGGTCTATTGGGGCCTGGGCAAGTGAAGGAAATGAGTCTGGAAACAAACTGTTT
AGGAGGTTCCGAAAAATGAATGCCAGGCAGTCCAAAGTCTATGAGATGGAGGATGTCTTG
Parus TAAATTGCCATCATTTGAAAAAACACCCT-------------------------

CTGATGACAGCCAGCTCATAAGCAAAGATCAGGCAGAAGAGGCTGTTTCTTCAAACAAAGAATTCATCCTGCATAAAGAT
GAAGAAGTGCCAAGAGGAGAAAAGATGGAGTTAACATGCAATAGGCAGGGTCTTGAGGAAGATGCCCATGCCATGAAAAC
ACARGACAATAGAGTGCATCAGACTAATCTGAAGCAATTCTGCCGCATCTGTGGAGTTTCATTTAAAACTGATTGCTACA
AGAGAACCTACCCAGTGCATGGGCCAGTGGATGATGAAACTCTGTGCCTTCTGAGAAAGAAAGAAAAAACAGCGACCTCT
TGGCCAGATCTTATTGCTAAGGTTTTCAAGATTGATGTGCGAGGGGATGTCGATACTATTCATCCCACTCAATTTTGTCA
CAACTGTTGGAGTATTATACATGGAAAATTCAGTAATACTCTATGTGAAGTATATTTTCCTAGGAACAGCGCAATGGAGT
GGCAACCCCATTCCTCAAACTGTGATGTGTGCCATACTGCCAGACGAGGAGTCAAGAGAAAAAGCCAGCCCCCAAGTGTA
CAACGTGGTAAACGTGTCAAAACCACTGGAGAACGTGCTCGGCTAAACAGAGGTGTAAAGAAC------------
CAAGCACAGATAAACAACAAAAATTTAATGAAAGACATTGTCAATTGCAAGGATATACATCTCAGCACCAAGCTGCTTTC
AATTGATTACCCAGTAGATTTCATTAAATCCATTTCTTGCCAGATTTGTGATCATATTTTGGCAGATCCATTGGAAACAA
CGTGCAGACACTTGTTTTGCAGAACTTGCATCCTTAAATGTATCAGGGTTATGGGCAGCTATTGCCCCTCCTGCTGGTAT
CCTTGCTTTCCTACTGATCTGGTAACCCCAGTGAAATCCTTCCTGAACATCCTTGATAACCTGACTATAAGATGCCCTGT
AAAGGAATGTGATGAAGAGATCTTGCACGGAAAATACGGCCAACACCTCTCTGGTCACAAGGAGATGAAAGATAGAGAGC
TCTGTAGCTACACCAATAAAGGCGGTCGACCAAGGCAGCACCTCCTGTCCTTGACGAGGAGAGCTCAGAAACATCGTCTG
AGGGAACTGAAACGTCAAGTCAAGGCTTTTGCTGAGAAAGAAGAGGGAGGTGATATAAAGGCTGTATGCATGACTTTGTT
CCTGCTAGCTTTGAGAGCAAAAAATGAACACAAACAAGCAGATGAACTGGAGGCTATAATGCAAGGGAGGGGATCTGGAC
TTCATCCCGCTGTCTGTTTGGCCATCCGAATCAACACGTTTCTCAGCTGTAGTCAGTATCATAAAATGTATAGAACAGTA
AAAGCTGTCACCGGGAGGCAGATCTTCCAGCCTTTGCATGCTCTTCGCACTGCTGAGAAAGCTCTCTTACCAGGTTATCA

CCCATTTGAGTGGAAACCTCCCCTGAAAAATGTATCCACTAACACAGAAGTGGGAATTATAGATGGACTATCAGGACTGC
CACTGTCAATTGATGATTACCCAGTAGACACAATTGCAAAGAGATTCCGATATGATGCAGCCTTGGTTTGTGCCTTAAAG
GACATGGAGGAGGAGATCTTGGAAGGCATGAAAGCAAAAAACCTGGACGACTATTTGAATGGCCCCTTCACTGTTGTAGT
AAAAGAGTCCTGTGATGGAATGGGAGACGTCAGTGAAAAGCATGGAAGTGGGCCTGCTGTCCCTGAGAAGGCTGTTCGAT
TTTCTTTCACAGTCATGAACATTTCTGTAGCACATGGGAATGAAAGCAAGAGGATCTTTGAGGAAGTAAAGCCCAATTCA
GAGTTGTGCTGTAAGCCCTTGTGCCTTATGCTGGCTGATGAATCAGATCATGAAACTCTAACAGCAATTCTGAGCCCCCT
CATAGCAGAAAGAGAGGCTATGAAAAACAGTGAGCTGCTGCTTGAAATGGGAGGCATCCTGAGAACATTTAGGTTCATCT
TTAGGGGTACAGGTTATGATGAGAAACTCGTGCGGGAAGTGGAAGGACTGGAGGCCTCAGGTTCCACTTACATTTGTACC
CTGTGCGATGCAACCCGCTTGGAGGCATCCCAGAATCTGGTCTTCCACTCCATAACCAGGAGCCATGCTGAAAATCTGGA
GCGATATGAAATATGGAGGTCCAACCCATATCACGAGTCTGTTGATGAGCTCCGTGACAGAGTGAAGGGTGTTTCAGCCA
AACCTTTTATTGAGACTGTTCCCTCCATAGATGCATTGCACTGCGACATTGGCAATGCAACAGAATTCTACAGGATTTTC
CAGATGGAGATTGGTGAACTTTACAAGAATCCCGATGTGTCTAAAGAGGAGAGGAAGAGGTGGCAGTTGACTCTTGACAA
ACACCTCAGGAAGAAGATGAACCTGAAGCCTATGTTGAAGATGAGTGGAAATTTTGCTAGAAAGCTTATGTCCAAAGAGA
CTGTAGAGGCAGTATGCGAATTAATAAAGTGTGAGGAAAGGCATGAAGCCCTAAAAGAACTAATGGACCTTTATCTGAAG
ATGAAGCCAGTGTGGCGGTCCTCATGCCCTGCCAAGGAGTGCCCAGAACTGCTGTGCCAGTACAGCTACAATTCACAGCG
TTTTGCAGAACTCTTATCTACAAAGTTCAAGTACAGGTATGAGGGCAAGATTACAAATTATTTCCACAAAACACTTGCTC
ATGTTCCTGAAATCATTGAAAGAGATGGGTCCATTGGAGCCTGGGCAAGTGAAGGAAATGAGTCCGGAAACAAACTGTTT
AGGAGGTTCCGAAAAATGAATGCCAGGCAGTCCAAAGTCTATGAGATGGAGGATGTCTTG
Petroica_rosea
??
??
??
??
??
??
??
??
??
??
??
??
???ATAAGATGTC
CTGTGAAGGAATGTGATGAAGAGATCTTGCATGGAAAATATGGCCAACACCTCTCCAGCCACAAGGAGATGAAGGACGGA
GAGGTCTACAGCTACATCAATAAAGGTGGTCGGCCGAGGCAGCACCTCCTGTCTTTGACCAGGAGAGCTCAGAAACATCG
TCTGAGGGAACTGAAACGTCAAGTCAAGGCTTTTGCTGAGAAAGAAGAGGGTGGTGATATAAAGGCTGTATGCATGACTT
TGTTCCTGCTAGCTTTGAGAGCAAAAAATGAACACAAACAAGCAGATGAACTGGAGGCTATAATGCAAGGGAAGGGATCT
GGACTTCATCCTGCTGTCTGTCTGGCCATCCGAATCAACACGTTTCTCAGCTGTAGTCAGTATCATAAAATGTATAGAAC
AGTAAAAGCTGTCTCTGGGAGGCAGATCTTCCAGCCTTTGCATGCTCTTCGTACTGCTGAGAAAGCCCTCCTACCAGGTT
ATCACCCCTTTGAGTGGAAACCTCCCCTGAAAAATGTATCCACTAACACAGAAGTGGGAATTATAGATGGACTATCAGGA
CTGCCACTCTCAATTGATGACTACCCAGTAGACACAATTGCAAAGAGATTTCGATACGATGCAGCCTTGGTTTGTGCCTT
AAAGGACATGGAGGAGGAGATCTTGGAAGGCATGAAAGCAAAAAACCTAGATGACTATTTGAATGGCCCCTTCACAGTCA
TAGTAAAAGAGTCCTGTGATGGAATGGGAGATGTCAGTGAGAAGCATGGAAGTGGGCCTGCTGTCCCAGAGAAGGCTGTT
CGCTTTTCTTTCACAATCATGAATATTTCTATAGCACATGGGAATGAAAGCAAAAGGATCTTTGAGGAATTAAAGCCCAA
TTCAGAGTTGTGCTGT??
??
??
??
??
??
??
??
??
??
??
??
??
Eopsaltria_australis
??
??
??

??
??
??
??
??
??
??
??
??
??ATAAGATGTC
CTGTAAAGGAATGTGATGAAGAGATCCTGCATGGAAAATATGGCCAACACCTCTCCAGCCACAAGGAGATGAAAGACGGA
GAGGTCTACAGTTACATAAATAAAGGTGGCCGACCAAGGCAGCACCTCCTGTCTTTGACAAGGAGAGCTCAGAAGCATCG
CCTGAGGGAACTGAAACGTCAAGTCAAGGCTTTTGCTGAGAAAGAAGAAGGTGGTGATATAAAGGCTGTATGCATGACTT
TATTCCTGCTAGCTTTGAGAGCAAAAAATGAACACAAACAAGCAGATGAACTGGAGGCTATAATGCAAGGGAAGGGATCT
GGACTTCATCCTGCTGTCTGTCTGGCCATCCGAATCAACACGTTTCTCAGCTGTAGCCAGTATCATAAAATGTATAGAAC
AGTAAAAGCTGTCTCTGGGAGGCAGATCTTCCAGCCTTTGCATGCTCTTCGCACTGCTGAGAAAGCCCTCCTACCAGGTT
ATCACCCATTTGAGTGGAAACCTCCCCTGAAAAACGTATCCACTAACACAGAAGTGGGAATTATAGATGGACTATCAGGA
CTGCCACTCTCAATTGATGACTACCCAGTAGACACAATTGCAAAGAGATTTCGGTATGATGCAGCCTTGGTTTGTGCCTT
AAAGGATATGGAAGAGGAGATCTTGGAAGGCATGAAATTAAGAAACCTGGATGACTATTTGAATGGCCCCTTCACTGTGG
TAGTAAAAGAGTCCTGTGATGGAATGGGAGATGTCAGTGAGAAGCATGGAAGTGGGCCTGCAGTCCCAGAGAAGGCTGTT
CGCTTTTCTTTCACAGTCATGAACATTTCTATAGCACACGGGAATGAAAGCAAGAGGATCTTTGAGGAAGTAAAGCCCAA
TTCAGAGTTGTGCTGTAAGCCCTTGTGCCTTATGCTGGCT??
??
??
??
??
??
??
??
??
??
??
??
??
Serinus
??
??
??
??
??
??
??
??
??
??
??
??
??
??
??
??
??
??
??
??
??
??
??
??
??

??
??
??
??
??
??
??
??
??
??
??
??
Icterus   TAAATTGCGATCATTCCAAAAAACAGCCT-------------------------
----
CTGATGACAGCCAGCACATACACAAAGATCAGGCAGAAGAGGCTGTTTCCTCAAACAAAGAAATCATCCTGCATGAAGAT
GAAGCAGTGCCAAGAGGAGAAAAGATGGAGTTAACGGGCAATAGGCAGGGACTTGAGGAAGATGCCCATGCCATGAAAAT
ACAAGACAACAGAGTTCATCAGAACAATCTGAAGCAACTCTGCCGCATCTGTGGGGTTTCATTTAAAACTGATTGTTCCA
AGAGAACTTACCCAGTGCATGGGCCAGTGGATGATGAAACTCTTTGGCTTCTGAGAAAGAAAGAAAAAACAGCAACCTCT
TGGCCAGATCTTATTGCTAAGGTTTTCAAGATTGATGTGCGAGGGGATGTTGACACYATCCATCCCACTCGATTTTGTCA
CAACTGTTGGAGTATTATACATAGAAAATTCAGTAATACTCTATGTGAAGTGTATTTTCCTAGGAACAGCACAATGGAGT
GGCAACCCCATTCCCCAAACTGTGATGTTTGCCACACTACCAGACGAGGAGTCAAGAGAAAAAGCCAGCCTCCAAGTGTG
CAACATGGCAAACGTGTAAAAAAGACTGGGGAACGTGCTCAGCTAAACAGAGGTGTAAAGAACCAACATATCAAACAAGC
ACAGATAAACAACAAAAATTTAATGAAAGAGATCGTCAATTGCAAGGATATACATCTCAGCATCAAGCTGCTTGTAGTTG
ATTACCCAGTAGATTTCATTAAATCCATTTCTTGCCAGATTTGTGATCATATTTTGGCAGATCCAGTGGAAACAACGTGC
AGGCACTTGTTTTGCAGAACTTGCATCCTTAAATGTATCAGGGTTATGGGCAGCTATTGCCCCTCCTGCTGGTATCCTTG
CTTTCCAACTGATCTGGTAACCCCAGTGAAATCCTTCCTGAACATCCTTGATAATYTGAGTATAAGATGCCCTGTAAAGG
AATGTGATGAAGAGATCTCACATGGAAAATATGGCCAACACCTTTCTGGCCACAAGGAGATGAAAGAAGGAGAGCTCTAT
AGCTACATCAATAAAGGTGGTCGACCGAGGCAGCACCTCCTGTCTTTGACGAGGAGAGCTCAGAAACATCGTCTGAGGGA
GCTGAAACGTCAAGTCAAGGCTTTTGCTGAGAAAGAAGAGGGCGGTGATATAAAGGCGGTGTGCATGACTTTGTTCCTGC
TAGCTCTGAGAGCAAAAAATGAACACAAACAAGCAGATGAACTGGAGGCTATAATGCAAGGCAGAGGATCTGGACTTCAT
CCTGCTGTCTGTCTGGCCATCCGAATCAACACGTTTCTCAGCTGTAGTCAGTATCATAAAATGTATAGAACAGTAAAAGC
TGTCACTGGGAGGCAGATCTTCCAGCCTTTGCATGCTCTTCGCACTGCTGAGAAAGCCCTCCTACCAGGTTATCACCCCT
TTGAGTGGAAACCTCCCCTGAAAAATGTATCCACTAACACAGAAGTGGGAATTATAGATGGATTATCAGGACTGCCACTC
TCAATTGATGATTACCCAGTAGACACAATTGCAAAGAGATTCCGATACGATGCAGCCTTGGTTTGTGCCTTAAAGGACAT
GGAGGAGGAGATCTTGGAAGGCATGAAAGCAAAAAACCTGGATGACTATTTGAATGGCCCCTTCACTGTGGTAGTAAAAG
AGTCCTGTGATGGAATGGGAGATGTCAGTGAGAAGCACGGAAGTGGGCCTGCTGTCCCAGAGAAGGCTGTTCGCTTTTCC
TTCACAGTCATGAACATTTCTATAGCACATGGGAATGAAAGCAAGAGGATCTTTGAGGAAGTAAAGCCCAATTCAGAGTT
GTGCTGTAAGCCCTTGTGCCTTATGCTGGCTGATGAATCAGATCATGAAACTCTGACAGCAATCCTGAGCCCCCTCATAG
CAGAAAGAGAGGCTATAAAAAACAGTGAACTGCTGCTTGAAATGGGAGGCATCCTGAGAACATTTAGATTCATCTTTAGG
GGTACAGGATATGATGAGAAACTCTTGAGGGAAGTGGAAGGGCTTGAGGCCTCAGGTTCCACTTATATTTGTACCCTGTG
TGATGCAACCCGCCTGGAGGCATCCCAGAATCTGGTCTTCCACTCCATAACCAGGAGCCATGCTGAAAATCTGGAGCGAT
ATGAAATATGGAGGTCCAACCCATACCACGAGTCTGTTGATGAGCTCCGTGACAGAGTGAAGGGTGTTTCAGCCAAACCT
TTTATTGAGACTGTTCCCTCCATAGATGCACTGCACTGCGACATTGGCAATGCAACAGAATTCTACAGGATCTTCCAGAT
GGAGATTGGTGAACTTTACAAGAATCCTGATGTGTCTAAAGAGGAGAGGAAGAGGTGGCAGTTAACTCTTGACAAACACC
TCAGGAAGAAGATGAACTTGAAGCCTATGCTGAAGATGAGTGGAAATTTTGCCAGAAAGCTCATGTCCAAAGAGACTGTA
GAGGCAGTATGTGAATTAATTAAGTGTGAGGAAAGGCATGAAGCCCTAAAAGAACTAATGGACCTTTATCTGAAGATGAA
GCCAGTGTGGCGATCCTCATGCCCTGCCAAGGAGTGCCCAGAACTGCTGTGCCAGTATAGCTTCAATTCACAGCGTTTTG
CAGAGCTCTTATCTACAAAGTTCAAGTATAGATATGAGGGCAAGATTACAAATTATTTCCACAAAACGCTTGCTCATGTT
CCTGAAATCATTGAAAGAGATGGGTCCATTGGGGCCTGGGCAAGCGAAGGAAATGAGTCTGGAAACAAACTGTTTAGGAG
GTTCCGAAAAATGAATGCCAGGCAGTCCAAGGTCTATGAGATGGAGGATGTCTTG
Motacilla   TAAATTGCGATCATTTGAAAAAACACCCT-------------------------
----
CTGATGACAGCCAGCACATACACAAAGATCAGGCAGAAGAGGCTGTTTCTTCAAACAAAGAAATCATCCTGCATGAAGAT
GAAGCAGTGCCAAGAGGAGAAAAGATGGAGTTAACAGGCAATATGCAGGGACTTGAGGAAGATGCT------
GTGAAAACACAAGACAATAGAGCTCATCAGAACAATCTGAAGCAACTCTGCCGCATCTGTGGGGTTTCATTTAAAACTGA
TTGTTCCAAGAGAACTTACCCAGTGCATGGGCCAGTGGATGATGAAACTCTTTGGCTTCTGAGAAAGAAAGAAAAAACAG
CAACCTCTTGGCCAGATCTTATTGCTAAGGTTTTCAAGATTGATGTGCGAGGGGATGTTGACACTATCCATCCCACTCAC
TTTTGTCACAACTGCTGGAGTATTATACATAGAAAATTTAGTAATACTCTATGTGAAGTGTATTTTCCTAGGAACAGCAC
AATGGAGTGGCAACCCCATTCCCCAAACTGTGATGTCTGCCATACTACCAAACGAGGAGTCAAGAGAAAAAGCCAGCCCC
CAAGTGTGCAACGGGGCAAACGTGTAAAAGCCACTGGGGAACRTGCTCAGCTAAACAGAGGTATAAAGAACCAGCAACTC

AAACAAGCACAGATAAACAACAAAAATTTAATGAAAGAGATTGTCAATTGCAAGGATATACATCTCAGCACCAAGCTGCT
TGTAGTTGATTACCCAGTAGATTTCATTAAATCCATTTCTTGCCAGGTTTGTGATCATATTTTGGCAGATCCAGTGGAAA
CAACATGCAGACACTTGTTTTGYAGAACTTGCATCCTTAAATGTATCAGGGTTATGGGCAGCTATTGCCCCTCCTGCTGG
TATCCTTGCTTTCCTACTGATCTGGTAACCCCAGTGAAATCCTTCCTGAATATTCTTGATAATCTGAGTATAAGATGCCC
TGTAAAGGAATGTGATGA?GAGATCTTGCATGGAAAATATGGCCAACACCTCTCTGGCCACAAGGAGATGAAAGAAGGAG
AGCTCTATAGCTACATCAATAAAGGTGGCCGACCGAGGCAGCACCTCCTGTCTTTGACAAGGAGAGCTCAGAAACATCGT
CTGAGGGAGCTGAAACGTCAAGTCAAGGCTTTTGCTGAGAAAGAAGAGGGTGGTGATATAAAGGCTGTATGCATGACTTT
GTTCCTGCTAGCTTTGAGAGCAAAAAATGAACACAAACAAGCAGATGAACTGGAGGCTATAATGCAAGGGAGGGGATCTG
GACTTCATCCTGCTGTCTGTCTGGCCATCCGAATCAACACGTTTCTCAGCTGTAGTCAGTATCATAAAATGTACAGAACG
GTAAAAGCTGTCACTGGGAGGCAGATCTTCCAGCCTTTGCATGCTCTTCGCACTGCTGAGAAAGCCCTCCTACCAGGTTA
TCACCCATTTGAGTGGAAACCTCCCCTGAAAAATGTATCCACTAACACAGAAGTGGGAATTATAGATGGACTATCAGGAC
TGCCACTCTCAATTGATGATTATCCAGTAGACACAATTGCAAAGAGATTCCGATATGATGCAGCCTTGGTTTGTGCCTTA
AAAGACATGGAGGAGGAGATCTTGGAAGGCATGAAAGCAAAAAACCTGGATGATTATTTGAATGGCCCCTTCACTGTGGT
AGTAAAAGAGTCCTGTGATGGAATGGGAGATGTCAGTGAGAAGCATGGAAGTGGGCCTGCTGTCCCAGAGAAGGCTGTTC
GCTTTTCTTTCACAGTCATGAACATTTCTATAGCACATGGGAATGAAAGCAAGAGGATCTTTGAGGAAGTAAAGCCCAAT
TCAGAGTTGTGCTGCAAGCCCTTGTGCCTTATGYTGGCTGATGAATCAGATCATGAAACTCTGACAGCAATCCTGAGCCC
CCTCATAGCAGAAAGAGAGGCTATGAAAAACAGTGAACTGCTGCTTGAAATGGGAGGCATCCTGAGAACATTTAGATTCA
TCTTTAGGGGTACAGGATATGATGAGAAACTCTTRCGGGAAGTGGAAGGGCTGGAGGCCTCAGGTTCCACTTATATTTGT
ACCCTGTGTGATGCAACCCGCTTGGAGGCATCCCAGAACCTGGTCTTCCACTCCATAACCAGGAGCCATGCTGAAAATCT
GGAGCGATATGAAATATGGAGGTCCAACCCATATCACGAGTCTGTTGATGAGCTCCGTGACAGAGTGAAGGGTGTTTCAG
CCAAACCTTTTATTGAGACTGTTCCCTCCATAGATGCATTGCACTGCGACATTGGGAATGCAACAGAATTCTACAGGATT
TTCCAGATGGAGATTGGTGAACTTTACAAGAATCCTGACGTGTCKAAAGAGGAGAGGAAGAGGTGGCAGTTGACTCTTGA
CAAACATCTCAGGAAGAAGATGAACTTGAAGCCTATGCTGAAGATGAGTGGAAATTTTGCTAGAAAGCTCATGTCCAAAG
AGACTGTAGAGGCAGTATGTGAATTAATTAGGTGTGAGGAAAGGCATGAAGCCCTWAAAGAACTAATGGACCTTTATCTG
AAGATGAAGCCAGTGTGGCGATCCTCRTGCCCTGCCAAGGAGTGCCCAGAACTGCTGTGCCAGTATAGCTACAATTCACA
GCGTTTTGCAGAGCTTTTATCTACAAAGTTCAAGTACAGATATGAGGGCAAGATTACAAATTATTTCCACAAAACGCTTG
CTCATGTTCCTGAAATCATTGAAAGAGATGGGTCCATTGGGGCCTGGGCAAGCGAAGGAAATGAGTCTGGAAACAAACTG
TTTAGGAGGTTCCGAAAAATGAATGCCAGGCAGTCCAAGGTCTATGAGATGGAGGATGTCTTG
Emberiza TAAATTGCGATCATTTGAAAAAACAGCCT-------------------------
----
CTGATGACAGCCAGCACATACACAAAGATCAGGCAGAAGAGGCTGTTTCTTCAAACAAAGAAATCATCCTGCATGAAGAT
GAAGCAGTGACAAGAGGAGAAAAGATGGAGTTAATGGGCAATAGGCAGGGACTTGAGGAAGATGCCCATGCCATGAAAAC
ACAAGAAAATAGAGTTCATCAGAACAATCTGAAGCAACTCTGCCGCATCTGTGGGGTTTCATTCAAAACTGATTKTTCCA
AGAGAACTYACCCAGTGCATGGGCCAGTGGATGATGAAACTCTTTGGCTTCTGAGAAAGAAAGAAAAAACAGCAACCTCT
TGGCCAGATCTTATTGCTAAGGTTTTCAAGGTTGATGTGCGAGGGGATGTTGACACTACCCATCCCACTCGATTTTGTCA
CAACTGTTGGAGTATTATACACAGAAAATTCAGTAATACTCTATGTGAAGTGTATTTTCCTAGGAACAGCACAATGGAGT
GGCAACCCCATTCCCCAAACTGTGATGTCTGCCATACTACCAGACGAGGAGTCAAGAGAAAAAGCCAGCCCCCAAGTGTG
CAACGTGGCAAACGTGTCAAAGCCACTGGGGAACGTGCTCAGCTAAACAGAGGTGTAAAGAACCAACATCTCAAACAAGC
ACAGATAAACAACAAAAATTTAATGAAAGAGATTGTCAATTGCAAGGATATACATCTCAGCACCAAGCTGCTTGTAGTTG
ATTACCCAGTAGATTTCATTAAATCCATTTCTTGCCAGATTTGTGATCATATTTTGGCAGATCCAGTGGAAACAACATGC
AGACACTTATTTTGCAGAACTTGCATCCTTAAATGTATCAGGGTTATGGGCAGCTATTGCCCCTCCTGCTGGTATCCTTG
CTTTCCTACTGATCTGGTWACCCCAGTGAAATCCTTCCTGAACATCCTTGATAATCTGACTATAAGATGCCCTGTAAAGG
AATGTGATGAAGAGATCCTGCATGGAAAATATGGCAAACACCTTTCTGGCCACAAGGAGATGAAAGAAGSAGAGCTCTAT
AGCTACATCAATAAAGGTGGCCGACCGAGGCAGCACCTCCTGTCTTTGACGAGGAGAGCTCAGAAACATCGTCTGAGGGA
GCTGAAACGTCAAGTCAAGGCTTTTGCTGAGAAAGAAGAGGGCGGTGATATAAAGGCTGTATGCATGACTTTGTTCCTGC
TAGCTTTGAGAGCAAAAAATGAGCACAAACAAGCAGATGAACTGGAGGCTATAATGCAAGGTAGGGGATCTGGACTTCAT
CCTGCTGTCTGTCTGGCCATCCGAATCAACACATTTCTCAGCTGTAGTCAGTATCATAAAATGTATAGAACAGTAAAAGC
TGTCACTGGGAGGCAGATCTTCCAGCCTTTGCATGCTCTTCGCACTGCTGAGAAAGCCCTCCTGCCAGGTTATCACCCAT
TTGAGTGGAAACCTCCCCTGAAAAACGTATCCACTAACACAGAAGTGGGAATTTTAGATGGACTATCAGGACTGCCACTC
TCAATTGATGATTACCCAGTAGACACAATTGCAAAGAGATTCCGATATGATGCAGCCTTGGTTTGTGCCTTAAAGGACAT
GGAGGAGGAGATCTTGGAAGGCATGAAAGCAAAAAACCTGGATGACTATTTGAATGGCCCCTTCACTGTGGTAGTAAAAG
AGTCCTGTGATGGAATGGGAGATGTCAGTGAGAAGCATGGAAGTGGGCCTGCTGTCCCAGAGAAGGCTGTTCGCTTTTCC
TTCACAATCATGAACATTTCTATAGCACATGGGAATGAAAGCAAGAGGATCTTTGAGGAAGTAAAGCCCAATTCAGAGTT
GTGCTGTAAGCCCTTGTGCCTTATGCTGGCTGATGAATCAGATCATGAAACTCTGACAGCAATCCTGAGCCCCCTCATAG
CAGAAAGAGAGGCTATGAAAAACAGTGAACTGCTGCTTGAAATGGGAGGCATCCTGAGAACATTTAGATTTGTCTTTAGG
GGTACAGGATATGATGAGAAACTCTTGCGGGAAGTGGAAGGGCTGGAGGCCTCAGGTTCCACTTATATTTGTACCCTGTG
TGATGCAACCCGCCAGGAGGCATCCCAGAATCTGGTCTTCCACTCCATAACAAGGAGCCATGCTGAAAATCTGGAGCGAT
ATGAAATATGGAGGTCCAACCCATATCACGAGTCTGTTGATGAGCTCCGTGACAGAGTGAAGGGTGTTTCAGCCAAACCT
TTTATTGAGACTGTTCCCTCCATAGATGCATTGCACTGCGACATTGGCAATGCAACAGAATTCTACAGGATCTTCCAGAT
GGAGATTGGTGAACTTTACAAGAATCCTGATGTGTCTAAAGAGGAGAGGAAGAGGTGGCAGTTGACTCTTGACAAACACC

TCAGAAAGAAGATGAACTTGAAGCCTATGCTGAAGATGAGTGGAAATTTTGCTAGAAAGCTCATGTCCAAAGAGACTGTA
GAGGCAGTATGTGAATTAATTAARTGTGAGGAAAGGCATGAAGCCCTCAAAGAACTAATGGACCTTTATCTGAAGATGAA
GCCAGTGTGGCGATCCTCATGCCCTGCCAAGGAGTGCCCAGAACTGCTGTGCCAGTATAGCTTCAATTCACAGCGTTTTG
CAGAGCTCTTATCTACAAAGTTCAAGTACAGATATGAGGGCAAGATTACAAATTATTTCCACAAAACACTTGCTCATGTT
CCTGAAATCATTGAAAGAGATGGGTCCATTGGGGCCTGGGCAAGTGAAGGAAATGAGTCTGGAAACAAACTGTTTAGGAG
GTTCCGAAAAATGAATGCCAGGCAGTCCAAAGTCTATGAGATGGAGGATGTCTTG
Pomatostomus
??
??
??
??
??
??
??
??
??
??
??
??
??ATAAGATGCC
CTGTAAAGGAATGTGATGAAGAGGTCTTGCATGGAAAATATGGCCGACACCTCTCCAGCCACAAGGATATGAAAGATAGA
GAGCTCCATAGCTACACAAATAAAGGTGGCCGACCGAGGCAGCACCTCCTGTCTTTGACAAGGAGAGCTCAGAAACATCG
TCTGAAGGAACTGAAACGTCAAGTGAAGGCTTTTGCTGAGAAAGAAGAGGGTGGTGATATAAAGGCTGTATGCATGACTT
TGTTCCTGCTAGCTCTGAGAGCAAAAAACGAACACAAACAAGCAGATGAACTGGAGGCTATAATGCAAGGGAGGGGATCT
GGACTTCATCCTGCTGTCTGTCTGGCCATCCGAATCAACACATTTCTCAGCTGTAGTCAGTATCACAAAATGTATAGAAC
AGTAAAAGCTGTCACTGGGAGGCAGATCTTCCAGCCTTTGCATGCTCTTCGCACTGCTGAGAAAGCCCTCCTACCGGGTT
ATCACCCCTTTGAGTGGAAACCGCCCTTGAAAAATGTATCCACTAACACAGAAGTGGGAATTATAGATGGGCTATCAGGA
CTGCCACTCTCGATTGATGACTACCCAGTAGACACAATTGCAAAGAGATTTCGATATGATGCAGCCTTGGTTTGTGCCTT
AAAGGACATGGAGGAGGACATCTTGGAAGGCATGAAAGCAAAAAACCTGGATGACTATTTGAATGGCCCCTTCACTGTGG
TAGTAAAAGAGTCCTGTGATGGAATGGGAGATGTCAGTGAGAAGCATGGAAGTGGGCCTGCTGTCCCAGAGAAGGCTGTT
CGCTTTTCTTTCACAGTCATGAACATTTCCATAGCACATGGGAATGAAAGCAAGAAGATCTTTGAGGAAGTAAAGCCCAA
TTCAGAGTTGTGCTGCAAGCCCTTGTGCCTTATGCTGGCT??
??
??
??
??
??
??
??
??
??
??
??
??
Rhipidura
??
??
??
??
??
??
??
??
??
??
??
??
??ATGAGATGCC
CTGTAAAGGAATGTGATGAACAGATCTTGCATGGAAAATATGGCCAACACCTCTCCAGCCACAAGGAGATGAAAGATAGA
GAGCTCTACAGCTACATAAATAAAGGTGGCCGACCGAGGCAGCACCTCCTGTCTTTGACAAGGAGAGCTCAGAAACATCG

TCTGAGGGAGCTGAAACGTCAAGTCAAGGCTTTTGCTGAGAAAGAAGAGGGCGGTGATATAAAGGCTGTATGCATGACTT
TGTTCCTGCTAGCTTTGAGAGCAAAAAATGAACACAAACAAGCAGATGAACTGGAGGCTATAATGCAAGGGAAGGGATCT
GGACTCCATCCTGCTGTCTGTCTGGCCATCAGAATCAACACATTTCTCAGCTGTAGTCAGTATCATAAAATGTATAGAAC
AGTAAAAGCGGTCAGTGGAAGGCAGATCTTCCAGCCTTTGCATGCTCTTCGCACWGCTGAGAAAGCCCTCCTACCAGGTT
ATCACCCATTTGAGTGGAAGCCTCCCTTGAAAAATGTATCCACTAACACAGAAGTGGGAATTATAGATGGACTATCAGGA
CTGCCACTCTCAATTGATGACTACCCAGTAGACACAATTGCAAAGAGATTTCGATATGATGTGGCCTTGGTTTGTGCCTT
AAAGGACATGGAGGAGGAGATCTTGGAAGGCATGAAAGCAAAAAACCTGGATGACTATTTGAATGGTCCCTTCACTGTGG
TAGTAAAGGAGTCCTGTGATGGAATGGGAGATGTCAGTGAGAAGCATGGAAGCGGGCCTGCTGTCCCAGAGAAGGCTGTT
CGCTTTTCTTTCACAGTCATGAACATTTCTATAGCACAGGGGAATGAAAGCAAGAGGATCTTTGAGGAAGTAAAGCCCAA
TTCAGAGTTGTGCTGC??
??
??
??
??
??
??
??
??
??
??
??
??
Pica_pica
??
??
??
??
??
??
??
??
??AAGCATACAACGTGGCAAACGTGTCAAAACCACTGGGGAGCGTGCTCGGCTAAACAGAGGTGTAAAGAAC--------
----
CAAGCACAGATAAACAACAAAAATTTAATGAAGGAGATTGTCAGTTGCAAGGATATACATCTCAGCACCAAGCTGCTTGC
AGTTGATTACCCAGTAGATTTCATTAAATCCATTTCTTGCCAGATTTGCGATCATATTTTGGCAGATCCAGTGGAAACAA
CATGCAGACAYTTGTTTTGCAGAACTTGCATCCTTAAATGTATCAGGGTTATGGGCAGCTATTGCCCCTCCTGCTGGTAT
CCTTGCTTTCCTACTGATCTGGTAACCCCAGTGAAATCCTTCTTGAACATCCTTGATAACCTGAGTATAAGATGCCCTGT
AAAGGAATGTGATGAAGAGATCTTGCATGGAAAATATGGCCAA?????????????????????????????????????
??
??
??
???GTATCATAAAATGYATAGAACAGTA
AAAGCTGTCACTGGGAGGCAGATCTTCCAGCCTTTGCATGCTCTTCGCACTGCTGAGAAAGCCCTCCTACCAGGTTATCA
CCCATTTGAATGGAAACCTCCCTTGAAAAATGTATCCACTAACACAGAAGTGGGAATTATAGATGGACTATCGGGACTGC
CACTCTCAATTGATGACTACCCAGTAGACACAATTGCAAAGAGATTTCGATATGATGCAGCCTTGGTTTGTGCCTTAAAG
GACATGGAGGAGGAGATCTTGGAAGGCATGAAAGCGAAAAACCTGGATGACTACTTGAACGGCCCCTTCACTGTGGTAGT
AAAAGAGTCCTGTGATGGAATGGGAGATGTCAGTGAGAAGCATGGAAGTGGGCCTGCTGTCCCAGAGAAGGCTGTTCGCT
TTTCTTTCACAGTCATGAACATTTCTATAGCACACGGGAATGAAAGCAAGAAGATCTTTGAGGAAGTAAAGCCCAATTCA
GAGTTGTGCTGCAAGCCCTTGTGCCTCATGCTGGCTGATGAATCAGATCATGAAACTCTGACGGCAATCCTGAGCCCCCT
CATA??
??
??AGCCATGCTGAAAATCTGGA
GCGATATGAAATATGGAGGTCCAACCCGTATCATGAATCTGTTGATGAGCTCCGTGACAGAGTGAAGGGTGTTTCTGCCA
AACCTTTTATTGAGACTGTTCCCTCCATTGATGCATTGCACTGTGACATTGGCAATGCAACAGAATTCTACAGGATTTTC
CAGATGGAGATTGGTGAACTTTACAAGAATCCTGATGTGTCTAAAGAGGAGAGGAAGAGGTGGCAGCTGACTCTTGACAA
ACACCTCAGGAAGAAGATGAACTTGAAGCCTATGTTGAAGATGAGTGGAAATTTTGCTAGAAAGCTCATGTCCAAGGAGA
CTGTAGAGGCCGTATGTGAATTAATAAAGTGTGAGGAAAGGCATGAAGCCCTAAAAGAACTAATGGACCTTTATCTGAAG
ATGAAGCCAGTGTGGCGATCCTCATGCCCTGCCAAGGAGTGCCCAGAACTGCTGTGTCAGTATAGCTACAATTCACAGCG
TTTTGCAGAGCTCTTATCTACGAAGTTCAAGTACAGATATGAGGGCAAGATTACAAATTATTTCCACAAAACACTTGCTC

ATGTTCCTGAAATCATTGAAAGAGATGGGTCCAT???
??
Manucodia   TAAATTGCGATCGTTTGAAAAAACACCCT--------------------------
----
CTAATGACAGCCAGCACATAAACAAAGATCAGGCAGAAGAGGCTGTTTCTTCAAACAAAGAATCCATCCTGCATGAAGAC
GAAGCAGTGCCAAGAGGAGAAAAGATGGAGTTAATGGGCAATAGGCAGGGACTTGAGGAAGATGCCCATGCCATGCAAAC
ACAAGACAATAGAGCTCATCAAAACAATTTGAAGGAACTCTGCCGCATCTGTGGAGTTTCATTTAAAACTGATTGTTACA
AGAGGACTTACCCAGTGCATGGGCCAGTGGATGATGAAACTCTGTGGCTTCTGAGAAAGAAAGAAAAAACAGCAACCTCC
TGGCCAGACCTTATTGCTAAGGTTTTCAAGATTGATGTGCGAGGGGATGTTGATAGTATCCATCCCACTCAATTTTGTCA
CAACTGTTGGAGTATTATACATAGAAAATTCAGTAATACTCTATGTGAAGTATATTTTCCTAGGAACAGCACAATGGAGT
GGCAACCGCACTCCCCAAACTGTGACGTGTGCCATACTACCAGACGAGGAGTCAAGAGAAAAAGCCAGCCCCCAAGTGTA
CAACGTGGCAAACGTGCCAAAACCACTGGGGAACGTGCTCGGCTAAACAGAGGTGTAAAGAAG------------
CAAGCACAAATAAACAACAAAAATTTAATGAAGGAGATTGTCAGTTGCAAGGATATACATCTCAGCACCAAGCTGCTTGC
AGTTGATTACCCAGTAGATTTCATTAAATCCATTTCTTGCCAGATTTGCGATCATGTTTTGGCAGATCCAGTGGAAACAA
CATGCAGACACTTATTTTGCAGAACTTGCATCCTTAAATGTATCAGGGTTATGGGCAGCTATTGCCCCTCCTGCTGGTAT
CCTTGCTTTCCTACTGATCTGGTAACCCCAGTGAAATCCTTCCTGAACATCCTTGATAACCTRAGTATAAGATGCCCTGT
AAAGGAATGTGATGAAGACATCTTGCATGGAAAATATGGCCAACACCTCTCCAGCCACAAGGAGATGAAAGATAGAGAGC
TCTATAGCTACATAAATAAAGGTGGCCGACCGAGGCAGCACCTCCTSTCTTTGACGAGGAGAGCTCAGAAACATCGTCTG
AGGGAGCTGAAACGTCAAGTCAAGGCTTTTGCTGAGAAAGAAGAGGGTGGTGATATAAAGGCCGTATGCATGACTTTGTT
CCTGCTAGCTTTGAGAGCAAAAAATGAACACAAACAAGCAGATGAACTGGAGGCTATAATGCAAGGGAGGGGATCTGGAC
TTCATCCTGCTGTCTGTCTGGCCATCCGAATCAACACTTTTCTCAGCTGTAGTCAGTATCATAAAATGTATAGAACAGTA
AAGGCTGTCAGTGGGAGGCAGATCTTCCAGCCTTTGCATGCTCTTCGCACTGCTGAGAAAGCCCTCCTACCAGGTTATCA
CCCATTTGAGTGGAAACCTCCCTTGAAAAATGTATCCACCAACACAGAAGTGGGAATTATAGATGGACTATCAGGACTGC
CACTCTCAATTGATGACTACCCAGTAGACACAATTGCAAAGAGGTTTCGATATGATGCGGCCTTGGTTTGTGCCTTAAAG
GACATGGAGGAGGAGATCTTGGAAGGCATGAAAGCAAAAAACCTGGATGACTACTTGAACGGCCCCTTCACTGTGGTAGT
AAAAGAGTCCTGTGATGGAATGGGAGATGTCAGTGAGAAGCATGGAAGTGGGCCTGCTGTCCCAGAGAAGGCTGTTCGCT
TTTCTTTCACAGTCATGAACATTTCTATAGCACAGGGGAATGAAAGCAAGAGGATCTTTGAGGAGGTAAAGCCCAATTCA
GAGTTGTGCTGCAAGCCCTTGTGCCTTATGCTGGCTGATGAATCAGATCATGAAACTCTGACAGCAATCCTGAGCCCCCT
CATAGCAGAAAGAGAGGCTATGAAAAACAGTGAACTGCTGCTTGAAATGGGAGGCATCCTGAGAACATTTAGATTCATCT
TTAGGGGCACAGGATATGATGAGAAACTCGTGCGGGAAGTGGAAGGGCTGGAGGCCTCAGGTTCCACTTACATTTGTACC
CTGTGCGATGCAACCCGCTTGGAGGCGTCCCAGAATCTGGTCTTCCACTCCATAACCAGGAGCCATGCTGAAAATCTGGA
GCGATATGAAATATGGAGGTCCAACCCATATCACGAATCTGTTGATGAGCTCCGTGACAGAGTGAAGGGTGTTTCAGCCA
AACCTTTTATTGAGACCGTTCCCTCCATAGATGCATTGCACTGCGACATTGGCAATGCAACAGAATTCTACAGGATTTTC
CAGATGGAGATTGGTGAACTTTACAAGAATCCTGACGTGTCTAAAGAGGAGAGGAAGAGGTGGCAGCTGACTCTTGACAA
ACACCTCAGGAAGAAGATGAACTTGAAGCCTATGTTGAAGATGAGTGGAAATTTTGCTAGAAAGCTCATGTCCAAAGAGA
CCGTAGAGGCAGTATGTGAATTAATAAAGTGTGAGGAAAGGCATGAAGCCCTAAAAGAACTAATGGACCTTTATCTGAAG
ATGAAGCCAGTGTGGCGATCCTCATGCCCTGCCAAGGAGTGCCCAGAACTGCTGTGCCAGTATAGCTACAATTCGCAGCG
TTTTGCRGAGCTCTTATCTACAAAGTTCAAATACAGATATGAGGGCAAGATTACAAATTATTTCCACAAAACACTTGCTC
ATGTTCCAGAAATCATTGAAAGAGATGGGTCCATTGGGGCCTGGGCAAGTGAAGGAAATGAGTCTGGAAACAAACTGTTT
AGGAGGTTCCGAAAAATGAACGCCAGGCAGTCCAAAGTCTATGAGATGGAGGATGTCTTG
Corvus_corone
TAAATTGCGATCATTTGAAAAAACACCCTCTGATGACAGCCAGCACATAAACAAAGATCAGGCAGAAGAGGCTGTTTCTT
TGACACCCTCTGATGACAGCCAGCACATAAACAAAGATCAGGCAGAAGAGGCTGTTTCTTCAAACAAAGAATTCATCCTG
CATAAAGATGAAGCAGTGCCAAGAGGAGAAAAGATGGAGTTAATGGGCAATAGGCAGGGACTTGAGGAAGATGCCCATGC
GATGCAAACACAAGACAATAGAGCTCATCAGAACAATTTGAAGGAACTCTGCCGCATCTGCGGAGTTTCATTTAAAACTG
ATTGTTACAAGAGGACTTACCCAGTGCATGGGCCAGTGGATGATGAAACTCTGTGGCTTCTGAGAAAGAAAGAAAAAACA
GCAACCTCTTGGCCAGACCTTATTGCTAAGGTTTTCAAGATTGATGTGCGAGGGGATGTTGATACTATCCACCCCACTCA
ATTTTGTCACAACTGTTGGAGTATTATACATAGAAAATTCAGTAATACTCTRTGTGAAGTATATTTTCCTAGGAACAGCA
CAATGGAGTGGCAACCACACTCCCCAAACTGTAATGTGTGCCGTACTACCAGACRAGGAGTCAAGAGAAAAAGCCAGCCC
CCAAGCGTACAACGTGGAAAACGTGTCAAAACCACTGGGGAGCGTGCTCGGCTAAACAGAGGTGTAAAGAAC--------
----
CAAGCACAGATAAACAACAAAAATTTAATGAAGGAGATTGTCAATTGCAAGAATATACATCTCAGCACCAAGCTGCTTGC
AGTTGATTACCCAATAGATTTCATTAAATCCATTTCTTGCCAGATTTGCGATCATATTTTGGCAGATCCAGTGGAAACAA
CATGCAGACACTTGTTTTGCAGAACTTGCATCCTTAAATGTATCAGGGTTATGGGCAGCTATTGCCCCTCCTGCTGGTAT
CCTTGCTTTCCTACTGATCTGGTAACCCCAGTGAAATCCTTCCTGAACATCCTTGATAACCTGAGTATAAGATGCCCTGT
AAAGGAATGTGATGAAGAGATCTTGCATGGAAAATATGGCCAACACCTCTCCAGCCACAAGGAGATGAAAGATAGAGAGC
TCTATAGCTACATAAATAAAGGTGGCCGACCAAGGCAGCACCTCCTGTCTTTGACGAGGAGAGCTCAGAAACATCGTCTG
AGGGAACTGAAGCGTCAAGTCAAGGCTTTTGCTGAGAAAGAAGAGGGCGGTGATATAAAGGCTGTATGCATGACTTTATT
TCTGCTAGCTTTGAGAGCAAAAAATGAACACAAACAAGCAGATGAACTGGAGGCTATAATGCAAGGGAGGGGATCTGGAC
TTCAYCCTGCTGTCTGTCTGGCCATCCGAATCAACACTTTTCTCAGCTGTAGTCAGTATCATAAAATGTATAGAACAGTA

AAAGCTGTCACTGGGAGGCAGATCTTCCAGCCTTTGCATGCTCTTCGCACTGCTGAGAAAGCCCTCCTACCAGGTTATCA
CCCATTTGAGTGGAAACCTCCCTTGAAAAATGTATCCACTAACACAGAAGTGGGAATTATAGATGGACTATCGGGACTGC
CACTCTCAATTGATGACTACCCAGTAGACACAATTGCAAAGAGATTTCGATATGATGCAGCCTTGGTTTGTGCCTTAAAG
GACATGGAGGAGGAGATCTTGGAAGGCATGAAAGCAAAAAACCTGGATGACTACTTGAACGGCCCCTTCACTGTGGTAGT
AAAAGAGTCCTGTGATGGAATGGGAGATGTCAGTGAGAAGCATGGAAGTGGGCCTGCTGTCCCAGAGAAGGCTGTTCGCT
TTTCTTTCACAGTCATGAACATTTCTATAGCACACGGGAATGAAAACAAGAGGATCTTTGAGGAAGTAAAGGCGAATTCA
GAGTTGTGCTGCAAGCCCTTGTGCCTCATGCTGGCTGATGAATCAGATCATGAAACTCTGACGGCAATCCTGAGCCCCCT
CATAGCAGAAAGAGAGGCTATGAAAAACAGTGAACTGCTGCTTGAAATGGGAGGCATCCTGAGAACATTTAGATTCATCT
TTAGGGGTACAGGATATGATGAGAAACTTGTGCGGGAAGTGGAAGGGCTGGAGGCCTCAGGTTCCACTTACATTTGTACG
CTGTGTGATGCAACCCGCTTGGAGGCATCCCAGAATCTGGTCTTCCACTCCATAACCAGGAGCCATGCTGAAAATCTGGA
GCGATATGAAATATGGAGGTCCAACCCATATCACGAATCTGTTGATGAGCTCCGTGACAGAGTGAAGGGTGTTTCAGCCA
AACCTTTTATTGAGACTGTTCCCTCCATTGATGCATTGCACTGTGACATTGGCAATGCAACAGAATTCTACAGGATTTTC
CAGATGGAGATTGGTGAACTTTACAAGAATCCTGATGTGTCTAAAGAGGAGAGGAAGAGGTGGCAGCTGACTCTTGACAA
ACACCTCAGGAAGAAGATGAACTTGAAGCCTATGTTGAAGATGAGTGGAAATTTTGCTAGAAAGCTCATGTCCAAGGAGA
CTGTAGAGGCCGTATGTGAATTAATAAAGTGTGAGGAAAGGCATGAAGCCCTAAAAGAACTAATGGACCTTTATCTGAAG
ATGAAGCCAGTGTGGCGATCCTCATGCCCTGCCAAGGAGTGCCCAGAACTGCTGTGTCAGTACAGCTACAATTCACAGCG
TTTTGCGGAGCTCTTATCTACAAAGTTCAAGTACAGATATGAGGGCAAGATTACAAATTATTTCCACAAAACACTTGCTC
ATGTTCCTGAAATCATTGAAAGAGATGGGTCCATTGGGGCCTGGGCAAGTGAAGGAAATGAGTCTGGAAACAAACTGTTT
AGGAGGTTCCGAAAAATGAATGCCAGGCAATCCAAAGTCTATGAGATGGAGGATGTCYTG
Vireo
??
??
??
??
??
??
??
??
??
??
??
??
??ATAAGATGCC
CTGTAAAGGAATGTGATGAAGAGATCTTGCATGGAAAATATGGTCAACACCTCTCTGGCCACAAGGAGATGAAAGACAGA
GAGCTTTATAGCTACATAAATAAAGGTGGCCGACCGAGGCAGCACCTCCTGTCTTTGACGAGGAGAGCTCAGAAACATCG
TCTGAGGGAACTGAAACGTCAAGTCAAGGCTTTTGCTGAGAAAGAAGAGGGTGGTGATATAAAGGCTGTATGCATGACTT
TGTTCCTGCTAGCTTTGAGAGCAAAAAATGAACACAAACAAGCAGATGAACTGGAGGCTATAATGCAAGGGAGGGGATCT
GGACTTCATCCTGCCGTCTGTCTGGCCATCCGAATCAACACGTTTCTCAGCTGTAGTCAGTATCATAAAATGTATAGAAC
AGTAAAAGCTGTCACTGGGAGGCAGATCTTCCAGCCTTTGCATGCTCTTCGCACTGCTGAGAAAGCCCTCCTACCAGGTT
ATCACCCATTTGAGTGGAAACCTCCCTTGAAAAATGTATCCACTAACACAGAAGTGGGAATTATAGATGGACTATCAGGA
CTGCCACTCTCAATTGATGACTACCCAGTAGACACAATTGCAAAGAGATTTCGATATGACGCGGCCTTGGTTTGTGCCTT
AAAGGACATGGAGGAGGAGATCTTGGAAGGCATGAAAGCAAAAAACCTGGATGACTATTTGAACGGCCCCTTCACTGTGG
TAGTGAAAGAGTCCTGTGATGGAATGGGAGATGTCAGTGAGAAGCATGGAAGTGGGCCTGCAGTCCCAGAGAAGGCTGTT
CGCTTTTCTTTCACAGTCATGAACATTTCTATAGCACATGGGAATGAAAGCAAGAGGATCTTTGAGGAAATAAAGCCCAA
TTCAGAATTGTGTTGT??
??
??
??
??
??
??
??
??
??
??
??
??
Camptostoma_obsoletum   TAAAGTGCGATCATTTGAAAAAACAR----------------------------
----
TTGATGACAGGCAGCACATAAACAAAGATCAGGCAGAAGGGATTGCTTCTTCAAACAAAGAAATCATACTGCATAAAGAT

AAAGCAGTGTCAAGAGAAGAAAAGATGGAGTTAATGGGCAATAGGCAGGCACTTGAGAAAGAGGCCCATGACATGAAAAC
ACGAGACAATAGAGCTCATCAGAACAATCTGAAGCAACTTTGTCGCATCTGTGGAGTTTCATTTAAAASTGATTGCTACA
AGAGAACTCATCCAGTGCATGGGCCGGTGGAYGATGAAACTCTGTGTCTTCTGAGAAAGAAAGAAAAAAAAGCAACYTCT
TGGCCAGATCTTATCGCTAAGGTGTTCAAGATTGATGTGCGAGGGGATGTTGATACTATTCATCCCACTCGGTTTGTCA
CAACTGTTGGAGTATTATCCATAGAAAATACAGTAGTACTCTATGTGAGGTGTATTTTCCTAGGAACAGCACCATGGAGT
GGCAACCTCACTCCCCAAACTGTGATGTGTGCCGTACTACCAGTCGAGGGGTCAAGAGAAAAAGCCAGCCACCAAGTGTA
CAACACGGCAAACGTGTGAAGACCACTGTGGAACGTGCTCGACTGAACAGAGGTGTAAAGAAC------------
CAGGCACAGATAAACAACAAAAATTTAATGAAAGAGATTGTCAATTGCAAGAATATACAYCTCAGCACCAAGCTGCTTGC
TGTTGATTACCCAGTAGATTTCATTAAATCAATTTCTTGCCAGATTTGTGAGCATATTTTGGCAGATCCAGTGGAAACAA
CGTGCAGACACTTGTTTTGCAGAACTTGCATCCTTAAATGYATCAAGGCTATGGGCAGCTATTGCCCCTCCTGCTGGTAT
CCTTGCTTTCCGACTGATCTGGTAACCCCAGTGAAATCCTTCCTGAACATCCTTGATAGCCTGGGTATAAGATGCCCTGT
AAAGGAATGTGATGAAGAGATTTTGCATGGAAAATATGGCCAACACCTCTCCAACCACAAGGAGATGAAAGACAGAGAGC
TCTATAGCTACGTAAATAAAGGTGGCCGACCAAGGCAGCATCTCCTGTCWTTGACAAGGAGAGCTCAGAAACATCGTCTG
AGGGAACTGAAACGTCAAGTCAAGGCTTTTGCTGAGAAAGAAGAGGGTGGTGATATAAAGGCTGTATGCATGACTTTGTT
CCTTCTAGCTTTAAGAGCGAAAAACGAACACAGACAAGCAGATGAACTGGAGGCTATAATGCAAGGGAGGGGATCTGGAC
TTCATCCTGCTGTCTGTCTGGCAATCCGAGTTAACACGTTTCTCAGCTGTAGCCAGTACCATAAAATGTATAGAACTGTA
AAAGCTGTCACGGGGAGGCAGATCTTCCAGCCCTTGCATGCTCTTCGCACTGCTGAGAAAGCCCTCCTACCAGGTTATCA
CCCATTTGAGTGGAAACCTCCCTTGAAAAATGTATCCACTAATACAGAAGTAGGAATTATAGATGGTCTATCCGGATTGC
CACTTTCAGTTGATGACTACCCAGTAGACACAATTGCAAAGAGATTTCGATATGATGCAGCCTTGGTTTGTGCCTTAAAG
GACATGGAGGAGGAGATCTTGGAAGGCATGAAAGCAAAAAATCTGGAYGACTATTTGAATGGCCCCTTCACTGTGGTAAT
AAAAGAGTCCTGCGATGGAATGGGAGATGTCAGTGAGAAGCATGGAAGTGGGCCTGCTGTCCCAGAGAAGGCAGTTCGCT
TTTCTTTCACAATTATGAACATTGCTATAGCACATGGGAATGAAAGCAAGAGGATCTTTGAGGAAGTAAAGCCAAATTCA
GAGTTGTGTTGCAAACCCTTGTGCCTTATGCTGGCTGATGAATCAGATCATGAAACTCTAACAGCAATCCTGAGCCCCCT
CATGGCAGAAAGAGAGGCTATGAAAAACAGTGAACTGCTGCTTGAAATGGGAGGCATCCTGAGAACATTCAGATTTGTCT
TTAGGGGTACAGGGTATGATGAGAAACTTGTGCGGGAAGTGGAAGGGCTGGAGGCCTCAGGTTCCACTTACATTTGTACY
CTGTGTGATGCAACTCGCTTGGAGGCATCCCAGAATTTGGTCTTCCACTCCATAACCAGGAGCCATGCTGAAAATCTGGA
GCGATATGAAATATGGAGGTCCAACCCATATCATGAATCTGTTGATGAGCTCCGTGACAGAGTGAAGGGTGTTTCAGCCA
AACCTTTTATTGACACCGTTCCCTCCATWGATGCACTGCACTGTGACATTGGCAATGCAACAGAATTCTACAGGATTTTC
CARATGGAGATTGGCGAACTTTACAAGAATCCTGATGTGTCTAAAGAGGAGAGGAAGAAGTGGCAGTTGACTCTTGACAA
ACACCTCAGGAAGAAGATGAACTTGAAGCCTATGATGAAGATGAGTGGAAATTTTGCTAGAAAGCTCATGTCCAAAGAGG
CAGTAGAGGCAGTATGTGAATTAATAAAGTGTGAGGAAAGGCATGAAGCCCTAAAAGAACTAATGGACCTCTATCTGAAG
ATGAAGCCAGTGTGGCGATCCTCATGCCCTGCCAAGGAGTGCCCGGAACTGCTGTGTCAGTATAGCTACAATTCACAGCG
YTTTGCTGAGCTCTTATCTACAAAGTTCAAGTACAGATATGAAGGCAAGATTACAAATTATTTCCACAAAACACTTGCTC
ATGTTCCTGAAATCATTGAAAGAGATGGGTCCATAGGGGCCTGGGCAAGTGAAGGAAATGAATCTGGAAACAAACTGTTT
AGGAGATTCCGAAAAATGAATGCCAGGCAGTCCAAATTCTATGAGATGGAGGATGTCTTG
Promerops_cafer   TAAATTGCGATCATTTGAAAAAACACCCT-------------------------
----
CTGATGACAGCCAGCACATAAACAAAGATCAGGCAGAAGAGGCTGTTTCTTCAAACAAAGAATTCATCCTGCATAAAGAT
GAAGCAGTGCCAAAAGGAGAAAAGATGGAGTTAACAGGCAATAGGCAGGGACTTGAGGAAGATGCCCATGCCATGAAAAC
ACAAGACAATAGAGCTCATCAGAACAATCTGGAGCAACTCTGCCGCATCTGTGGAGTTCCGTTTAAAACTGACTGTTACA
AGAGAACTTACCCAGTGCATGGGCCAGTGGATGATGAAACTCTGTGTCTTCTGAGAAAGAAAGAAAAAACAGCAACCTCT
TGGCCAGATCTTATTGCTAAGGTTTTCAAGATYGATGTGCGAGGGGATGTCGATACTATCCATCCCACTCGATTTTGTCA
CAACTGTTGGAGCATTATACATCGAAAATTCAGTAATACTCTRTGTGAAGTATATTTTCCTAGGAACAGCACAATGGAGT
GGCAACCCCATTCCCCAAACTGTGATGTGTGCCATACTACCAGACGAGGAGTCAAGAGAAAAAGCCAGCCCCCAAGTGTA
CAACGTGGCAAACGTGTCAAAACCACTGGGGAACGTGCTCGGCTAAACAGGGGTGTAAAGAAC------------
AAAGCACAGATAAACAACAAAAATATAATGAAAGAGATTGTCAATTGCAAGGACATACATCTCAGCACCAAACTGCTTGC
AGTTGATTACCCAGTAGATTTCATTAAATCCATTTCTTGTCAGATTTGTGATCATATTTTGGCAGATCCAGTGGAAACAA
CATGCAGACACTTGTTTTGCAGAACTTGCATCCTTAAATGTATCAGGGTTATGGGCAGCTATTGCCCCTCCTGCTGGTAT
CCTTGCTTTCCTACGGATCTGGTAACCCCAGTGAAATCCTTCCTGAACATCCTTGATAACCTGAGTATAAGATGCCCTGT
AAAGGAATGTGATGAAGAGATCTCGCATGGAAAATACGGCCAACACCTCTCCRGCCACAAGGACATGAAAGAGAGAGAGC
TCTAYAGCTACATAAATAAAGGTGGCCGACCGAGGCAGCACCTCCTGTCTTTGACGAGGAGAGCTCAGAAACATCGTCTG
AGGGAACTGAAACGTCAAGTCAAGGCTTTTGCTGAGAAAGAAGAGGGCGGTGATATAAAGGCTGTATGCATGACTCTGTT
YCTGCTAGCTTTGAGAGCAAAAAATGAACACAAACAAGCAGATGAACTGGAGGCTATAATGCAAGGGAGAGGATCTGGAC
TTCATCCTGCTGTCTGTCTGGCCATCCGAGTCAACACGTTTCTCAGCTGTAGTCAGTATCATAAAATGTATAGAACAGTA
AAAGCTGTCACTGGGAGGCAGATCTTCCAACCTTTGCATGCTCTTCGCACTGCTGAGAAAGCCCTCCTGCCAGGTTATCA
CCCATTTGAGTGGAAACCTCCCCTGAAAAATGTATCCACTAACACAGAAGTGGGAATTATAGATGGACTATCAGGACTGC
CACTCTCAGTTGATGATTACCCAGTAGACACAATTGCAAAGAGATTCCGATATGATGCAGCCTTGGTTTGTGCCTTAAAG
GACATGGAGGAGGAGATCTTGGAAGGCATGAAAGAAAAAAACCTGGATGACTATTTGAATGGCCCCTTCACTGTGGTAAT
AAAAGAGTCCTGTGATGGAATGGGAGATGTCAGTGAGAAGCATGGAAGTGGGCCGGCTGTCCCAGAGAAGGCTGTTCGCT
TTTCTTTCACAGTCATGAACATTTCTATAGCACATGGGAATGAAAGCAAGAGGATCTTTGAGGAAGTAAAGCCCAATTCA

GAGTTGTGCTGTAAGCCCTTATGCCTTATGCTGGCTGATGAATCAGATCATGAAACTCTGACAGCAATCCTGAGCCCCCT
CATAGCAGAAAGAGAGGCTATGAAAAACAGTGAACTGCTGCTTGAAATGGGAGGCATCCTAAGAACATTTAGATTCGTCT
TTAGGGGTACAGGATATGATGAGAAACTCGTGCGGGAAGTGGAAGGGCTGGAGGCTTCAGGTTCCACTTATATTTGTACC
CTGTGTGATGCAACCCGCTTGGAGGCATCGCAGAATCTGGTCTTCCACTCCATAACCAGGAGCCATGCTGAAAATCTGGA
GCGATATGAAATATGGAGGTCCAACCCATATCACGAGTCTGTTGATGAGCTCCGTGACAGAGTGAAGGGTGTTTCAGCCA
AACCTTTTATTGAGACTGTTCCCTCCATAGATGCAYTGCACTGTGATATTGGCAATGCAACAGAATTCTACAGGATTTTC
CAGATGGAGATTGGTGAACTTTACAAGAATCCTGACGTGTCTAAAGAGGAGAGGAAGAGGTGGCAGATGACTCTTGACAA
ACACCTCAGGAAGAAGATGAACTTGAAGCCTATGTTGAAGATGAGTGGAAATTTTGCTAGAAAGCTCATGTCCAAAGAGA
CTGTAGAGGCAGTATGTGAATTAATAAAGTGCGAGGAAAGGCATGAAGCCCTAAGAGAACTAATGGACCTTTATCTGAAG
ATGAAGCCAGTGTGGCGATCCTCATGCCCTGCCAAGGAGTGCCCAGAACTGCTGTGCCAGTATAGCTACAATTCACAGCG
TTTTGCAGAGCTGTTGTCTACAAAGTTCAAGTACAGATATGAGGGCAAGATCACAAATTATTTCCACAAAACACTTGCTC
ATGTTCCTGAAATCATTGAAAGAGATGGGTCCATTGGGGCTTGGGCAAGTGAAGGAAATGAGTCTGGAAACAAACTGTTT
AGGAGGTTCCGAAAAATGAATGCCAGGCAGTCCAAAGTTTATGAGATGGAGGATGTCTTG
Oriolus TAAATTGCGATCATTTGAAAAAACGCCCT-------------------------
----
CTGATGACAGCCAGCACATAAACAAAGGTCAGGCAGAAGAGGCTGTTTCTTCAAACAAAGAATTCATCCTGCATAAAGAT
GAAACGGTGCCAAGAGGAGAAAAGATGGAGTTAACCGGCAGTAGGCAGGGACTTGAGGAAGATGCCCATGCCATGGAAAC
ACAAGACAACAGAGCTCATCAGAACAATTTGAAGCAACTCTGCCGCATCTGTGGAGTTTCATTTAAAACTGATTGTTACA
AGAGGACTTACCCAGTGCATGGGCCAGTGGATGATGAAACTCTGCGGCTTCTGAGAAAGAAAGAAAAAACAGCAACCTCT
TGGCCAGACCTTATTGCTAAAGTTTTCAAGATTGATGTGCGAGGGGATGTCGATACTATCCATCCCACTCAATTTTGTCA
CAACTGCCGGAGTATTATACATAGAAAATTCAGTAATACTCTATGTGAAGTATATTTTCCTAGGAACAGCACAATGGAGT
GGCAACCGCACTCCCCAAATTGTGATGTGTGCCATACTACCAGACGAGGAGTCAAGAGAAAAAGCCAGCCCCCAAGTGTG
CAACGTGGCAAACGTGTCAAAACCACTGGGGAACGTGCTCGGCTAAACAGAGGTGTAAAGAAC------------
CAAGCACAGGTTAACAACAAAAATTTAATGAAAGAAATTGTCAATTGCAAGGATATACATCTCAGCACCAAGCTGCTTGC
AGTTGATTACCCAGTAGATTTCATTAAATCCGTTTCTTGCCAGATTTGCGATCATATTTTGGCAGATCCAGTGGAAACAA
CATGCAGACACTTGTTTTGCAGAACTTGCATCCTTAAATGTATCAGGGTTATGGGCAGCTATTGCCCCTCCTGCTGGTAT
CCTTGCTTTCCTACTGATCTGGTAACCCCAGTGAAATCCTTCCTGAACATCCTTGATAACCTGAGTATAAGATGCCCTGT
AAAGGAATGTGATGAAGAGATCTTGCATGGAAAATACGGCCAACACCTCTCTGGCCACAAGGAGATGAAAGATAGAGAGC
TCTATAGCTACATAAATAAAGGTGGCCGACCGAGGCAGCAYCTCCTGTCTTTGACGAGGAGAGCACAGAAACATCGCCTG
AGGGAACTGAAACGTCAAGTCAAGGCTTTTGCTGAGAAAGAAGAGGGCGGTGATATAAAGGCTGTATGCATGACTTTATT
CCTGCTAGCTTTGAGAGCAAAAAATGAACACAAACAAGCAGATGAACTGGAGGCTATACTGCAAGGGAGGGGATCTGGAC
TTCATCCTGCTGTGTGTCTGGCCATCCGAATCAACACGTTTCTCAGCTGTAGTCAGTATCATAAAATGTACAGAACAGTA
AAAGCTGTCACTGGGAGGCAGATCTTCCAGCCTTTGCATGCTCTTCGCACTGCTGAGAAAGCCCTCCTACCAGGATATCA
CCCATTTGAGTGGAAACCTCCCTTGAAAAATGTATCCACTAACACAGAAGTGGGAATTATAGATGGACTATCAGGACTGC
CACTCTCAATTGATGACTACCCAGTAGACACGATTGCAAAGAGATTTCGATATGATGCGGCCTTGGTTTGTGCTTTAAAG
GACATGGAGGAGGAGATCTTGGAAGGCATGAAAGCAAAAAACCTGGACGACTATTTGAACGGCCCCTTCACTGTGGTAGT
AAAAGAGTCCTGTGATGGAATGGGAGACGTCAGTGAGAAGCATGGAAGTGGGCCTGCTGTCCCAGAGAAGGCTGTTCGCT
TTTCTTTCACAGTCATGAACATTTCTATAGCACACGGGAATGAAAGCAAGAGGATCTTTGAGGAAGTAAAGCCCAATTCA
GAGTTGTGCTGCAAGCCCTTGTGCCTTATGCTGGCTGACGAATCAGACCATGAAACTCTGACGGCAATCCTGAGCCCCCT
CATAGCAGAAAGAGAGGCTATGAAAAACAGTGAACTGCTGCTTGAAATGGGAGGCATCCTGAGAACATTTAGATTCGTCT
TTAGGGGTACAGGATATGATGAGAARCTCGTGCGGGAAGTGGAAGGGCTGGAGGCCTCAGGTTCCACTTACATTTGTACC
CTGTGTGATGCAACCCGCTTGGAGGCGTCCCAGAATCTGGTCTTTCACTCCATAACCAGGAGCCATGCTGAAAATCTGGA
GCGATATGAAATATGGAGGTCCAACCCATATCACGAATCTGTTGATGAGCTCCGTGACAGAGTGAAGGGTGTTTCAGCCA
AACCTTTTATTGAGACTGTTCCCTCCATAGATGCGTTGCACTGCGACATTGGCAATGCAACAGAATTCTACAGGATTTTC
CAGATGGAGATTGGTGAACTTTACAAGAATCCTGACGTATCTAAAGAGGAGAGGAAGAGGTGGCAGCTGACRCTTGACAA
ACACCTCAGGAAGAAGATGAACTTGAAGCCTATGTTGAAGATGAGTGGAAACTTTGCTAGAAAGCTCATGTCCAAAGAGA
CCGTAGAGGCAGTGTGTGAATTAATAAAGTGTGAAGAAAGGCATGAAGCCCTAAAAGAACTAATGGACCTGTATCTGAAG
ATGAAGCCAGTGTGGCGATCCTCCTGCCCTGCCAAGGAGTGCCCAGAACTGCTGTGCCAGTATAGCTACAATTCACAGCG
TTTTGCAGAGCTCTTATCTACAAAGTTCAAGTACAGATACGAGGGCAAGATTACAAATTATTTCCACAAAACACTTGCTC
ATGTTCCTGAAATCATTGAAAGAGATGGGTCCATTGGGGCCTGGGCAAGTGAAGGGAATGAGTCGGGAAACAAACTGTTT
AGGAGGTTCCGAAAAATGAATGCCAGGCAGTCCAAAGTCTATGAGATGGAGGATGTCTTG
Dendroica
??
??
??
??
??
??
??
??

??
??
??
??
??
??
??
??
??
??
??
??
??
??
??
??
??
??
??
??
??
??
??
??
??
??
??
??
??
??
Nectarinia
??
??
??
??
??
??
??
??
??
??
??
??
??
??
??
??
??
??
??
??
??
??
??
??
??
??
??
??
??
??
??

??
??
??
??
??
??
??
Amytornis_striatus
??
??
??
??
??
????CCTCTTGGCCAGATCTTATTGCTAAGGTTTTCAAGATTGATGTGCGAGGGGATGTTGATACTATCCATCCCACTCG
ATTCTGTCACAACTGTTGGAGTATTATACATAGAAAATACAGTAATACTCCATGTGAAGTGTATTTTCCTAGGAACAGCA
CAATGGAGTGGCAACCCCACTCCCCAAACTGTGATGTGTGCCATACTACCAGGCGAGGAGTCAAGAGAAAAAGCCAGCCC
CTAAATGTACAACGTGGCAAACGCATCAAAACCACTGGGAACGTGCTCGGCTAAACAGAGGTGCAATAAAC---------
----
CAAGCACAGATAAACAACAAAAATTTAATGAAGGAGATTGTCAATTGCAAGGATATACATCTCAGCACCAAGCTGCTTGC
AGTTGATTACCCAGTAGATTTTATTAAATCCATTTCTTGCCAGATTTGTGAGCATATTTTGGCAGATCCAGTGGAAACAA
CATGCAGGCACCTGTTTTGCAGAACTTGCATCCTTAAGTGTATCAGGGTTATGGGCAGCTATTGCCCTACCTGCTGGTAT
CCTTGCTTTCCTACTGATCTGGTAACCCCAGTGAAATCCTTCCTGAATATCCTTGATAACCTGAGTATAAGATGCCCTGT
AAAGGAATGTGACGAAGAGATCTTGCATGGAAAATATGGCCAGCACCTCTCCAGCCACAAGGAGATGAAAGATAGAGAGC
TCTACAGCTACGTAAACAAAGGYGGCCGACCAAGGCAGCACCTCCTGTCTTTGACGAGGAGAGCTCAGAAACATCGTTTG
AGGGAACTGAAACGTCAGGTCAAGGCTTTTGCTGAGAAAGAAGAGGGCGGTGATATAAAGGCTGTGTGCATGACTTTGTT
CCTGCTAGCTTTGAGAGCRAAAAATGAACACAAACAAGCAGATGAACTGGAAGCTATAATGCAAGGGAGGGGATCTGGCC
TTCATCCTGCTGTYTGTCTGGCAATCCGAGTCAACACATTTCTCAGCTGTAGTCAGTATCACAAAATGTATAGAACAGTA
AAAGCTGTCAGTGGGAGGCAGATCTTCCAGCCTTTGCACGCCCTTCGCAATGCTGAGAAAGCCCTCCTACCAGGTTACCA
CCCATTCGAGTGGAAACCTCCCTTGAAAAATGTATCCACTAACACAGAAGTGGGAATTATAGATGGACTGTCAGGTCTGC
CACTCTCAATTGATGACTACCCAGTAGACACAATTGCAAAGAGATTTCGATATGATGCAGCCCTGGTTTGTGCCTTAAAG
GACATGGAGGAGGAGATCTTGGAAGGCCTGAAAGCAAAAAATCTGGATGACTATTTGAATGGCCCCTTCACTGTGGTAGT
GAAAGAGTCCTGTGATGGAATGGGAGATGTCAGTGAGAAGCATGGAAGTGGGCCCGCTGTCCCAGAGAAGGCTGTTCGCT
TTTCTTTCACAGTCATGAACATTTCTATAGCACATGGGAATGAAAGCACGAGGATCTTTGAGGAAGTAAAGCCCAATTCA
GAGYTGTGCTGT??
??
??
??
??
??
??
??
??
??
??
??
??
Pitta   TAAAGTGCGAACATTTGAAAAAATGCCCT-------------------------
----
CTGACGGCAAGCAGCACATTAACAAAGATCAGGCAGAAGAGGTTGCTTCTTCAAACAAGAAAATC?TACTGCATAAAGAT
GAAGCAGTGCCAAGAGGAGAAAAGATGGAGTTAACGGGCAACAGGCAGGCACTTGAGAAAGATGCCCATGACATGAAAAC
ACGAGACAACAGAGCTCATCAGAACAATCTGAAGCAACTTTGCCGCATCTGTGGAGTTTCATTTAAAACTGATTGTAACA
AGAGAACTCACCCAGTGCATGGGCCGGTGGATGACGAAACTCTGTGGCTTCTGAGAAAGAAAGAAAAAAAAGCAACCTCT
TGGCCGGATCTTATTGCTAAGGTTTTCAAGATTGATGTGCGAGGGGATGTTGATACTATCCATCCCACTCGATTTTGTCA
CAACTGTTGGAGTATAATCCATAGAAAATTCAGTAATAATTTATGTGAAGTATATTTTCCTAGAAATAGCACGATGGAGT
GGCAACCTCACTCCCCAAACTGTGATGTATGCCATACTACCAGTCGAGGGATCAAGAGAAAAAGCCAGCCACCAAGTGTA
CAACATGGAAAACGTGTGAAGACCATTGTGGAACGTGCTCGATTAAACCGAGGTGTAAAGAAC------------
CAAGCACAAATAAACAACAAAAATTTAATGAAAGAGATTGTCAATTGCAAGAATATACATCTCAGCACCAAGCTGCTTGC
AGTTGATTACCCAGTAGATTTCATTAAAGCAATTTCTTGCCAGATTTGTGAGCATATTTTGGCAGATCCAGTGGAAACAG
TATGCAGACACTTGTTTTGCAGAACTTGCATCCTTAAATGTCTCAAGGTTATGGGCAGTTATTGCCCCTCCTGCTGGTAT
CCTTGCTTCCCTACAGATCTGGTAACCCCAGTGAAATCATTCCTGAACATCCTTGATAGCTTGACTATAAAATGCCCTGT

AAAGGAATGTGATGAAGAGATCTTACATGGAAAATATGGCCAACACCTCTCCAGCCACAAGGAGATGAAAGATAGAGAGC
TCTATAGCTACATAAATAAAGGTGGCCGACCAAGGCAGCACCTTCTATCTTTGACAAGGAGAGCTCAGAAACATCGTCTG
AGAGAACTGAAACGTCAAGTCAAGGCTTTTGCTGAGAAAGAAGAGGGTGGTGATATAAAGGCTGTATGCATGACTTTATT
CCTTCTAGCTTTAAGAGCAAAAAATGAACACAAACAAGCAGATGAGTTGGAGGCTATAATGCAAGGGAGGGGATCTGGTC
TTCATCCTGCTGTCTGTCTGGCAATTCGAGTCAACACATTTCTCAGCTGTAGCCAATACCATAAAATGTATAGAACCGTA
AAAGCTGTCACTGGGAGACAGATCTTCCAGCCTTTGCATGCTCTTCGCACTGCTGAGAAAGCCCTTCTACCAGGTTATCA
CCCATTTGAGTGGAAACCTCCCTTGAAAAATGTATCCACCAACACAGAAGTTGGAATTATAGATGGACTATCAGGATTGC
CACTCTCAATTGATGACTACCCAGTAGACACAATTGCAAAGAGATTTCGATATGATGCAGCCTTGGTTTGTGCCTTAAAG
GACATGGAGGAGGAGATCTTGGAAGGCATGAAAGCAAAAAATCTGGATGACTATTTGAATGGTCCTTTCACTGTGGTAGT
AAAAGAGTCCTGTGATGGAATGGGAGATGTCAGTGAGAAGCATGGAAGTGGACCTGCTGTCCCAGAGAAGGCTGTTCGCT
TTTCTTTCACAGTTATGAACATTGCTATAGCACATGGGAATGAAACCAAGAGGATCTTTGAAGAAGTAAAGCCCAATTCG
GAGTTGTGTTGCAAGCCCTTGTGCCTTATGCTGGCTGATGAATCAGATCATGAAACTCTGACGGCAATCCTGAGTCCCCT
CATAGCAGAAAGAGAGGCTATGAAAAACAGTGAACTGCTGCTTGAAATGGGAGGCATCCTGAGAACATTCAGATTCATCT
TTAGGGGTACAGGATATGATGAGAAACTTGTGCGAGAAGTGGAAGGGCTGGAGGCCTCAGGTTCCACTTACATTTGTACC
CTATGTGATGCAACTCGCTTGGAGGCATCACAGAATTTGGTCTTTCACTCCATAACCAGGAGCCATGCTGAGAATCTGGA
GCGATATGAAATATGGAGGTCCAACCCATATCATGAATCTGTTGATGAGCTCCGTGACAGAGTGAAGGGTGTTTCAGCCA
AACCTTTTATTGAGACCGTTCCCTCCATAGATGCACTGCACTGCGACATTGGCAATGCAGCAGAATTCTATAGGATTTTC
CAGATGGAGATTGGTGAACTTTACAAGAATCCCGATGTGTCTAAAGAGGAGAGGAAGAGGTGGCAGTTGACTCTTGACAA
ACACCTCAGGAAGAAGATGAACCTGAAGCCTATGCTGAGGATGAGTGGAAATTTTGCTAGAAAGCTCATGTCCAAAGAGA
CAGTAGAGGCAGTATGTGAATTAATAAAGTGTGAGGAAAGGCACGAAGCCCTAAAAGAACTAATGGACCTTTATCTCAAG
ATGAAACCAGTGTGGCGATCCTCATGCCCTGCCAAAGAATGCCCAGAACTGCTGTGCCAGTATAGCTACAATTCACAGCG
TTTTGCTGAGCTCTTATCTACAAAGTTCAAGTACAGATATGAAGGCAAGATTACAAACTATTTCCACAAAACGCTTGCTC
ATGTTCCTGAAATAATTGAAAGAGATGGGTCCATTGGGGCCTGGGCAAGTGAAGGAAATGAGTCTGGAAACAAACTGTTT
AGAAGGTTCCGAAAAATGAATGCCAGGCAGTCCAAATGCTATGAGATGGAGGATGTCTTG
Toxorhamphus TAAATTGCGATCATTTGAAAAAACACCCT-------------------------
----
CTGATGACAGCCAGCACATCAACAAAGATCAGGCAGAAGAGGCTGTTTCTTCAAACAAAGAACTCATCCTGCATAAAGAT
GAAGCAGTGCCAAGAGGAGAAAAGATGGAGTTAACGGGCAATAGGCAGGGACTTG---
AAGATGCCCATGCCATGAAAACACAAGACAATAGAGCTCATCAGAACAATTTGAAGCAACTCTGCCGCATCTGTGGAGTT
TCATTTAAAACTGATTGTTACAAGAGAACTTACCCAGTGCATGGGCCAGTGGATGATGAAACAGTGGGGCTTCTGAGAAA
GAAAGAAAAAACAGCAACCTCTTGGCCAGACCTTATTGCTAAGGTTTTCAAGATCGATGTGCGAGGGGATGTTGATACTA
TCCATCCCACTCAATTTTGTCACAACTGTCGGAGTATCATACATAGAAAATTCAGCAATACTCTATGTGAAGTATATTTT
CCTAGGAACAGCACAATGGAGTGGCAACCCCACTCCCCAAACTGTGATGTGTGCCATACTACCAGACGAGGAGTCAAGAG
AAAAAGCCAGCCCCCAAGTGTACAACGTGGCAAACGGGTCAAAACCACTGGGGAACGTGCTCGGCTAAACAGAGGTGTAA
AGAAC------------
CAGGCACAGATAAACAACAAAAATCTAATGAAAGAGATTGTCAATTGCAAGGATATACATCTCAGCACTAAGCTGCTTGC
AGTTGATTACCCAGTAGATTTCATTAAATCCCTTTCTTGCCAGATTTGTGATCATATTTTGGCAGATCCTGTGGAAACAA
CATGCAGACACTTGTTTTGCAGAACTTGCATCCTTAAATGTATCAGGGTTATGGGCAGCTATTGCCCCTCCTGCTTGTAT
CCTTGCTTTCCTACTGATCTGGTCACCCCAGTGAAATCCTTCCTGAATATCCTTGATAACCTGAGTATAAGATGCCCTGT
AAAGGAATGTGATGAAGAGATCTTGCATGGAAAATATGGCCAACACCTCTCCAGCCACAAGGAGATGAAAGACAGAGAGC
TCTACAGCTACATAAATAAAGGTGGCCGACCGAGGCAGCACCTCCTGTCTTTGACGCGGAGAGCTCAGAAACATCGTCTG
AGGGAACTGAAACGTCAAGTCAAGGCTTTTGCTGAGAAAGAAGAGGGCGGTGATATAAAGGCTGTATGCATGACTTTGTT
CCTGCTAGCTTTGAGAGCAAAAAATGAACACAAACAAGCAGATGAGCTGGAGGCTATAATGCAAGGGAGGGGATCTGGAC
TTCATCCTGCTGTCTGTCTGGCCATCCGAATCAACACGTTTCTCAGCTGTAGTCAGTATCATAAAATGTATAGAACAGTA
AAAGCTGTCTCTGGGAGGCAGATCTTCCAGCCTTTGCATGCTCTTCGTGCTGCTGAGAAAGCCCTCCTACCAGGTTATCA
CCCATTTGAGTGGAAACCTCCCTTGAAAAATGTATCCACCAACACAGAAGTGGGAATTATAGATGGGCTATCCGGACTAC
CACTCTCAATCGATGACTACCCAGTGGACACAATTGCAAAGAGATTTCGATATGATGCAGCCTTGGTTTGTGCCTTAAAG
GACATGGAGGAAGAGATCTTGGAAGGCATGAAAGCACAAAACCTGGACGATTATTTGAACGGCCCCTTCACTGTGGTACT
AAAAGAGTCCTGTGATGGAATGGGAGATGTCAGTGAGAAGCATGGAAGTGGGCCTGCTGTCCCAGAGAAGGCTGTTCGCT
TTTCTTTCACAGTCATGAACATTTCTGTAGCACATGGGAATGAAAGCAAGAGGATCTTTGAGGAAGTAAAGCCCAATTCA
GAGTTGTGCTGTAAGCCCTTGTGCCTTATGCTGGCTGATGAATCAGATCATGAAACTCTGACGGCAATCCTGAGCCCCCT
CATAGCAGAAAGAGAGGCTATGAAAAACAGTGAACTGCTGCTTGAAATGGGAGGCATCCTGAGAACATTTAGATTTGTCT
TTAGGGGTACAGGATACGATGAGAAACTCGTGCGGGAAGTGGAAGGGCTGGARGCCTCAGGTTCCACTTACATTTGTACC
CTGTGTGATGCAACCCGCTTGGAGGCATCCCAGAATCTGGTCTTCCACTCCATAACCAGGAGCCACGCTGAAAATCTGGA
GCGATATGAAATATGGAGGTCCAACCCATATCACGAGTCTGTTGATGAGCTCCGTGACAGAGTGAAGGGTGTTTCAGCCA
AACCTTTTATTGAGACCGTTCCCTCCATAGATGCATTGCACTGCGACATTGGCAATGCAACAGAATTCTACAAGATTTTC
CAGATGGAGATTGGTGAACTTTACAAGAATCCTGACGTGTCTAAAGAGGAGAGGAAGAGGTGGCAGTTGACTCTTGACAA
ACACCTCAGGAAGAAGATGAACTTGAAGCCTATGTTGAAGATGAGTGGAAATTTTGCTAGAAAGCTCATGTCCAAAGAGA
CTGTAGAGGCAGTGTGTGAATTAATAAAGTGTGAGGAAAGGCATGAAGCACTAAAAGAACTAATGGACCTTTATCTGAAG
ATGAAACCAGTGTGGCGATCCTCATGCCCTGCCAAGGAGTGCCCAGAACTGCTGTGCCAGTATAGCTACAATTCACAGCG

TTTTGCAGAGCTCCTATCTACAAAGTTCAAGTACAGATATGAAGGCAAGATTACAAATTATTTCCACAAAACACTTGCTC
ATGTTCCTGAAATCATTGAAAGAGATGGGTCCATTGGGGCCTGGGCAAGTGAAGGAAATGAGTCTGGAAACAAACTGTTT
AGGAGGTTCCGAAAAATGAATGCCAGGCAGTCCAAAGTCTATGAGATGGAAGATGTCTTG
Orthonyx_temminckii TAAATTGCGATCATTTGAAAAAACACCCT-------------------------
----
CTGATGACAGCCAGCACATAAACAAAGATCAGGCAGAAGAGGCTGCTTATTCAAACAAAGAATTCATCCTGCATAAAGAT
GAAGCARTGCCAAGAGGAGAAAAGATGGAGTTAACGGGCAATAGGCAGGCACTTGAGGAAGATGCCCATGCCATCAAAAC
ACAAGACAGTAGAGCTCATCAGAACAATCTGAAGCAACTCTGCCGCATCTGTGGAGTTTCATTTAAAACTGATTGTTACA
AGAGAACTTACCCAGTGCATGGGCCAGTGGATGATGAAACTCTGTTGCTCCTGAGAAAGAAAGAAAAAACAGCAACCTCT
TGGCCAGATCTCATTGCTAAGGTTTTCAAGATTGATGTGCGAGGGGATGTCGATACTATCCATCCCACTCGATTTTGTCA
CAACTGTTGGAGTATTATACATAGAAAATTCAGTAATACTCTATGTGAAGTRTATTTTCCTAGGAACAGCACAATGGAGT
GGCAACCCCACTCCCCAAACTGTGATGTGTGCCATACTACCAGCCGAGGAGTCAAGAGAAAATGCCAGCCCCCAACTGTA
CAACGTGGCAAACGTGTCAAAACCACTGGGGAACGTGCTCGGCTAAACAGAGGTGTAAAGAAC------------
CAAGCACAGATAAACAACAAAAATTTAATGAAAGAGATTGTCAATTGCAAGGATATACATCTCAGCACCAAGCTGCTTGC
AGTTGATTACCCAGTAGATTTCATTAAATCCATGTCTTGCCAGATTTGTGATCATATTTTAGCAGATCCAGTGGAAACAA
CATGCAGACACTTGTTTTGCAGAACTTGCATCGTTAAATGTATCAGGGTTATGGGCAGCTATTGCCCCTCCTGCTGGTAT
CCTTGCTTTCCTACTGATCTGGTAACCCCAGTGAAATCCTTCCTGAACATCCTTGATAACCTGAGTATAAGATGCCCTGT
AAAGGAATGTGATGAAGAGATCTTGCATGGAAAATATGGCCAACACCTTTCCAGCCACAAGGAGATGAAAGACAGAGAGC
TCTATAGCTACATAAATAAAGGTGGCCGACCGAGGCAGCACCTCCTGTCTTTGACGAGGAGAGCTCAGAAACATCGTCTG
AGGGAACTGAAACGTCAAGTCAAGGCTTTTGCTGAGAAAGAAGAGGGTGGTGATATAAAGGCTGTATGCATGACTTTGTT
CCTGCTAGCTTTGAGAGCAAAAAAATGAACACAAACAAGCAGATGAACTGGAGGCTATAATGCAAGGGAGGGGATCTGGAC
TTCACCCTGCTGTCTGTCTGGCCATCCGGATCAACACGTTTCTCAGCTGTAGTCAATATCACAAAATGTACAGAACAGTA
AAAGCTGTCACTGGGAGGCAGATCTTCCAGCCTTTGCATGCTCTTCGCACTGCTGAGAAAGCCCTCCTACCAGGTTATCA
CCCATTCGAGTGGAAACCTCCCTTGAAAAATGTATCCACTAACACAGAAGTGGGAATTATAGATGGACTGTCAGGGCTGC
CACTCTCAATTGATGACTACCCAGTGGACACAATTGCAAAGAGATTTCGATATGATGCAGCCTTGGTTTGTGCCTTAAAG
GACATGGAAGAGGAGATCTTGGAAGGCATGAAAGCAAAAAACCTGGATGACTATCTGAACGGCCCCTTCACTGTGGTAGT
AAAAGAGTCCTGTGATGGAATGGGAGATGTCAGTGAGAAGCATGGAAGTGGGCCTGCTGTCCCAGAGAAGGCTGTTCGCT
TTTCTTTCACAGTCATGAACATTTCTATAGCTCATGGGGATGAAAGCACGAGGGTCTTCGAAGAAGTAAAGCCCAATTCA
GAGTTGTGCTGCAAGCCCTTGTGCCTTATGCTGGCTGATGAATCAGATCATGAAACTCTGACAGCAATCCTGAGCCCCCT
CATAGCAGAAAGAGAGGCTATGAAAAACAGCGAACTGCTGCTTGAAATGGGAGGCATCCTGAGAACATTTAGATTCGTCT
TTAGGGGTACAGGATATGATGAGAAACTCGTGCGGGAAGTGGAAGGGCTGGAGGCCTCAGGCTCCACTTACATTTGCACC
CTGTGTGATGCAACCCGCTTGGAGGCGTCCCAGAATCTGGTCTTTCACTCCATAACCAGGAGCCATGCAGAAAATCTGCA
GCGATATGAAATATGGAGGTCCAACCCATATCATGAATCTGTAGATGAGCTCCGTGACAGAGTGAAGGGTGTTTCAGCCA
AGCCTTTTATTGAGACCGTTCCCTCCATAGATGCATTGCACTGTGACATTGGCAATGCAACAGAATTCTACAGGATTTTC
CAGATGGAGATTGGTGAACTTTACAAGAATCCTGACGTGTCTAAAGAGGAGAGGAAGAGGTGGCAGTTGACTCTTGACAA
ACACCTCAGGAAGAAGATGAACTTGAAGCCTATGTTGAAGATGAGTGGAAATTTTGCTAGAAAGCTCATGTCCAAAGAGA
CTGTAGAGGCAGTATGTGAATTAATAAAGTGTGAGGAAAGGCATGAAGCCCTAAAAGAACTAATGGACCTTTATCTGAAG
ATGAAGCCAGTGTGGCGATCCTCATGCCCTGCCAAGGAGTGCCCAGAACTGCTGTGCCAGTATAGCTACAATTCACAGCG
TTTTGCGGAGCTCTTATCTACAAAGTTCAAGTACAGATAYGAGGGCAAGATTACAAATTATTTCCACAAAACACTTGCTC
ATGTTCCTGAAATCATTGAAAGAGATGGGTCCATTGGGGCCTGGGCAAGTGAAGGAAATGAGTCTGGAAACAAACTCTTT
AGAAGGTTCCGAAAAATGAATGCCAGGCAGTCCAAAGTCTATGAGATGGAGGATGTCTTG
Sericulus_chrysocephalus
??
??
??
??
??
??
??
??
??
??
??
??
??
??
??
??
??ATCC
GGACTTCATCCTGCTGTCTGTCTGGCAATCCGAGTCAACACGTTTCTCAGCTGTAGTCAGTACCATAAAATGTATAGAAC

AGTAAAAGCTGTCACCGGGAGGCAGATCTTCCAGCCTTTGCATGCCCTTCGCACTGCTGAGAAAGCCCTCCTGCCAGGTT
ACCACCCATTCGAGTGGAAACCTCCCTTGAAAAATGTATCCACTAACACAGAAGTGGGAATTATAGATGGACTATCAGGA
TTGCCACTTTCAATTGATGACTACCCAGTAGACACAATTGCAAAGAGATTTCGATATGATGCAGCCTTGGTTTGTGCCTT
RAAGGACATGGAGGAGGAGATCTTGGAAGGCATGAAAGCAAAAAATCTGGATGACTATTTGAATGGCCCCTTCACAGTGG
TAGTAAAAGAGTCCTGTGATGGAATGGGGGATGTCAGTGAAAAGCATGGAAGTGGGCCTGCTGTCCCAGAGAAGGCTGTT
CGCTTTTCTTTCACAGTCATGAACATTTCTATAGCACATGGGAATGAAAGCAAGAGGATTTTTGAGGAAGTAAAGCCCAA
TTCAGAGTTGTGCTGCAAGCCCTTGTGCCTTATGCTGGCTGATGAATCAGATCATGAAACTCTGACGGCAATCCTGAGCC
CCCTCATAGCAGAAAGAGAGGCTATGAAAAACAGTGAACTGCTGCTTGAAATGGGAGGCATCCTGAGAACATTCAGATTC
ATCTTTAGGGGTACAGGATATGATGAGAAACTTGTGCGGGAAGTGGAAGGGCTGGAGGCCTCGGGTTCCACTTACATTTG
CACCCTGTGTGATGCAACCCGCTTGGAGGCATCCCAGAATTTGGTCTTCCACTCCATAACCAGGAGCCATGCTGAAAATC
TGGAGCGATATGAAATATGGAGGTCCAACCCATATCACGAATCCGTTGATGAGCTCCGTGACAGAGTGAAGGGCGTTTCA
GCCAAACCTTTTATTGAGACYGTTCCCTCCATAGATGCACTGCACTGCGACATTGGCAACGCAACAGAATTCTACAGGAT
CTTCCAGATGGAGATTGGTGAACTTTACAAGAATCCTGATGTRTCTAAAGAGGAGAGGAAGAGGTGGCAGTCGACTCTTG
ACAAACACCTCAGGAAGAAGATGAACTTGAAGCCTATGTTGAAGATGAGTGGAAATTTTGCTAGAAAGCTCATGTCCAAA
GAGACCGTAGAAGCAGTATGTGAATTAATAAAGTGTGAGGAAAGGCATGAAGCCCTAAAAGAACTAATGGACCTTTATCT
GAAGATGAAGCCAGTGTGGCGATCCTCATGCCCTGCCAAGGAGTGCCCAGAACTGCTCTGCCAGTATAGCTACAATTCAC
AGCGTTTTGCAGAGCTCTTATCCACAAAGTTCAAGTACAGATATGAAGGCAAGATTACAAATTACTTCCACAAAACGCTT
GCTCATGTTCCTGAAATCATTGAAAGAGATGGTCCATTGGGGCCTGGCAAGTGAAGGAAATGAGT???????????????
??
Cyclarhis_gujanensis
??
??
??
??
??
??
??
??
??
??
??
??
??
??
??
??
??
??
??
??
??
??
??
??
??
??
??
??
??
??
??
??
??
??
??
??
??
??

[RAG-2]

Polyborus_plancus
??
??
??
??
??
??
??
??
??
??
??
??
??
??
????????????????????????????????
Falco
??
??
??
??
??
??
??
??
??
??
??
??
??
??
????????????????????????????????
Tyrannus
AGATCCTGCCCCACTGGTGTTTTCCTCCTCGATATAAAGCAGAATGAGCTCAAAATGAAACCTGCCTTCTTCTCCAAAGA
CTCATGTTACCTTCCCCCTCTCCGCTACCCTGCTCTTTGCACACATAGAAGCAATGCAAAGTCTGATGAGTACCAGTATA
TCATCCATGGTGGTAAAACACCTAACAATGACCTCTCTGATAAGATTTACTTTATAAGTCTGGTAAGCAAAAGTAGCAAG
AAAATGACATTCCAATGCATTGAGAAAGACCTGGGTGGAGACGTCCCTGAAGCTAGATATGGGCATACAATTAATGTAGT
TCATAGCCGGGGGAAAAGCTTGAGTGTTATATTTGGAGGGAGATCGTATACTCCTCTTGAACAAAGAACCACTGAAAAAT
GGAACAGCGTAGTGGACTGTTTGCCATCTGTGTTTCTTGTTGATTTTGAGTTTGGATGCTGTACATCATACATGCTTCCA
GAGCTTCAGGATGGACTTTCTTTCCATGTTTCAATTGCCAGAGATGATACGATCTACATTTTGGGAGGCCATTCACTTCA
AAATAACACCAGGGRCCCCAACTTGTACAAGCTAAAAGTTGATCTCCCACTGGGCAGCCCAGCTGTGACCTGCACCATCT
TGCCAGGGGGGATATCAGTGTCAAGTGCTATAGTGACTCAGATCAGTGATACTGAATTTGTCCTTGTCGGTGGCTACCAC
TCTGACAACCAGAAACGGTTGGTGTGTAACACCATAGTTCTGGAGGATAGTAAGATAGAGATTGTTGAAAGGGAGAGCCC
AGAGTGGACACCAGATATTAAACACTGCAAAATATGGTTTGGCTGTGATATGGGCAAAGGGTCTGTATTGCTGGGCATTC
CAGGAGCCAACAAACAGTTAATCTCAGATGCAAACTACTTCTACATTTTGAGATGCAAAGGAGAAGAAGAGGACAAGGAA
GAAGAATTGACAACACAGATTTGCAGTCAGACATCAAGTGAAGACCCTGGAGATTCCACTCCATTTGAAGATTCGGAGGA
GTTTTGCTTTAGTGCTGAAGCCAGTAGCTTTGACATTGATGATACTGACACTTACAATGAGGATGATGAAGAAGATGAAT
CAGAAACGGGCTACTGGATCACCTGCTGTGCC
Myiarchus
AGAGCCTGCCCCACTGGTGTTTTCCTCCTCGATATAAAGCAGAATGAGCTCAAAATGAAACCTGCCTTCTTCTCCAAAGA
CTCGTGTTACCTTCCCCCTCTCCGCTACCCTGCTCTTTGCACACTCAGAAACAATGCAAAGTCTGATGAGTACCASTATA
TCATCCATGGTGGTAAAACACCTAACAATGACCTCTCTGATAAGATTTACTTTATACGTCTGGTAAACAAAAGTAGCAAG
AAAATGACTTTCCAATGCACTGAGAAAGACCTGGGTGGAGATGTCCCTGAAGCTAGATATGGGCATACAATTAATGTAGT
TCATAGCCGGGGGAAAAGCATGAGTGTTATATTTGGAGGGAGATCGTATACTCCTCTTGAACAAAGAACCACTGAAAAGT
GGAACAGCGTAGTCGACTGTTTGCCATCTGTGTTTCTTGTTGATTTTGAGTTTGGATGCTGTACATCGTACATGCTTCCG
GAGCTTCAAGATGGACTTTCTTTCCATGTTTCAATCGCCAGAGATGATACGATCTACATTTTAGGAGGCCATTCACTTCA
AAATAACACCAGGTGCCCCAACTTGTACAAGCTAAAAGTTGATCTCCCACTGGGCAGCCCAGCTGTGACCTGCACCATCT
TGCCCGGGGGGATATCAGTGTCAAGTGCTATAGTGACTCAAATCAGTGATACTGAATTTGTCCTTGTCGGTGGCTACCAC
TCCGACAACCAGAAACGGTTGGTGTGTAACACCATAGTTTTGGAGGATAGTAAGATAGAGATTGTTGAAAGGGAGAGCCC
AGAGTGGACACCAGATATTAAACACTGCAAAATATGGTTTGGCTGTGATATGGGCAAAGGGTCTGTATTGCTGGGCATTC

CAGGAGCCAACAAACAGTTAATCTCAGATGCAAACTACTTCTACATTTTGAGATGCAAAGGAGAAGAAGAGGACAAGGAA
GGAGAATTGACAACACAAATTTGCAGTCAGACATCAAGTGAAGACCCTGGAGATTCCACTCCATTTGAAGACTCGGAGGA
GTTTTGCTTTAGTGCTGAAGCCAGTAGCTTTGACATTGATGATACTGACACTTATAATGAGGATGATGAAGAAGATGAAT
CAGAAACGGGCTACTGGATCACTTGCTGTGCC
Hypocnemis_cantator
AGATCCTGCCCCACTGGTGTTTTCCTCCTTGATATAAAGCAGAATGAGCTCAAAATGAAACCTGCCTTCTTCTCCAAAGA
CTCATGTTACCTTCCCCCTCTCCGCTACCCTGCTCTTTGCACACTCCGAAGCAACACAAAGTCTGATGAATACCAGTATA
TCATCCATGGTGGTAAAACACCTAACAATGACCTTTCTGATAAGATATATTTTATGAGTCTGATAAGCAAAAAYAGCAAG
AAAATGATGGTCCGATGCATTGAGAAAGACCTGGGTGGAGATGTCCCTGAAGCTAGATATGGGCATACAATTAATGTAGT
GCATAGCCGGGGAAAAAGCATGAGTGTTATATTTGGAGGGAGATCATAYACTCCTCTTACACAAAGAACCACTGAAAAAT
GGAACAGCGTAGTTGACTGTTTGCCATCTGTGTTTCTTGTTGATTTTGAGTTTGGATGCTGTACATCATACATACTTCCA
GAGCTTCAAGATGGACTTTCTTTCCATGTTTCAATTGCCAGAGATGATACAATCTACATTTTGGGAGGCCATTCACTTCA
AAATAACACCAGGTGCCCCAACCTGTATAAGCTAAAAGTTGATCTCCCACTGGGCAGCCCAGCTGTGACCTGCACCATGT
TGCCAGGGGGAATATCAGTGTCAAGTGCTATAGTGACCCAAATCGGTGATACTGAATTTGTCCTTGTTGGTGGCTACCAC
TCTGACAACCAGAAACGGTTGGTGTGTAACACCATAGTTCTGGAAGATAGTAAGATAGAGATTGTTGAAAGGGTGAGCCC
AGAGTGGACACCAGATATTAAACACTGCAGAATATGGTTTGGCTGTGATATGGGCAAAGGATCTATCTTGCTGGGCATTC
CAGGGGCCAACAAACAGTTAATCTCAGATGCAAACTACTTCTACATTTTGAGATGCAAAGAAGCAGAAGAGGACAAGGAA
GAAGAGTTGACAACACAAATCTGCAGTCAGGCATCAAGTGAAGACCCTGGAGATTCCACTCCATTTGAAGATTCAGAGGA
ATTTTGTTTTGGTGCTGAAGCCAATAGCTTTGATATTGATGATAATGACACTTACAATGAGGATGATGAAGATGATGAAT
CAGAAACGGGCTACTGGATCACCTGCTCTGCC
Phlegopsis
AGATCCTGCCCCACTGGTGTTTTCCTCCTTGATATAAAGCAGAATGAGCTCAAAATGAAACCTGCCTTCTTCTCCAAAGA
CTCATGTTACCTTCCCCCTCTCCGCTACCCTGCTCTTTGCATACTCAGAAGCAACACAAAGTCTGATGAATACCAGTATA
TCATCCATGGTGGTAAAACACCTAACAATGACCTTTCTGATAAGATTTATTTTATGAGTCTGGTAAGCAAAAACAGCAAG
AAAATGATGATCCGATGCATTGAGAAAGACTTGGGTGGAGATGTCCCTGAAGCTAGATATGGGCATACAATTAATGTAGT
GCATAGCCGGGGAAAAAGCATGAGTGTTATATTTGGAGGGAGATCATATACTCCTCTTACACAAAGAACCACTGAAAAAT
GGAACAGCGTAGTTGACTGTTTGCCATCTGTGTTTCTTGTTGATTTTGAGTTTGGATGCTGTACATCATACATACTTCCA
GAGCTTCAAGATGGACTTTCTTTCCATGTTTCAATTGCCAGAGATGATACGATCTACATTTTGGGAGGCCATTCACTTCA
AAATAACACCAGGTGCCCCAACCTGTACAAGCTAAAAGTTGATCTCCCACTGGGCAGCCCAGCTGTGACCTGCACCATGT
TGCCAGGGGGGATATCAGTGTCAAGTGCTATAGTGACCCAAATCGGTGATACTGAATTTGTCCTTGTCGGTGGCTACCAC
TCTGACAACCAGAAACGGTTGGTGTGTAACACCATAGTTCTGGAAGACAGTAAGATAGAGATTGTTGAAAGGGTGAGCCC
AGAGTGGACACCAGATATTAAACACTGCAGAATATGGTTTGGCTGTGATATGGGCAAAGGATCTGTATTGCTGGGCATTC
CAGGGGCCAACAAACAGTTAATCTCAGATGCAAACTACTTCTACATTTTGAGATTCAAAGAAGCAGAAGAGGACAAGGAA
GAAGAGTTGACAACACAAATCTGCAGTCAGACATCAAGCGAAGACCCTGGAGATTCCACTCCATTTGAAGATTCAGAGGA
ATTTTGTTTTGGTGCTGAAGCCAATAGCTTTGATATTGATGATAATGACACTTACAATGAGGATGATGAAGACGATGAAT
CAGAAACAGGCTACTGGATCACCTGCTCTTCC
Manacus_manacus
AGATCCTGCCCCACTGGTGTTTTCCTCCTCGATATAAAGCAGAATGAGCTCAAAATGAAACCTGCCGCCTTCTCCAAAGA
CTCATGTTACCTTCCCCCTCTCCGCTACCCTGCTCTTTGCACACTCAGAAGCAATGCAAAGTCTGATGAGTACCAGTATA
TCATCCATGGTGGTAAAACACCTAACAATGACCTCTCTGATAAGATTTACTTTATAAGTCTGGTAAGCAAAAATAGCAAG
AAAATGACGTTCCAATGCATTGAGAAAGACCTGGGTGGAGATGTCCCTGAAGGTAGATATGGGCATACAATTAGTGTAGT
TCATAGTCGGGGAAAAAGCATGAGTGCTATATTTGGAGGGAGATCGTATACTCCTCTTGCACAAAGAACCACTGAAAAAT
GGAACAGCGTAGTTGAYTGTTTGCCATCTGTGTTTCTTGTTGATTTTGAGTTTGGATGCTGCACATCATACATGCTTCCA
GAGCTTCAAGATGGACTTTCTTTCCACGTTTCAATCGCCAGAGATGATACGATCTACATTTTGGGAGGCCATTCACTTCA
AAATAACACCAGGTGCCCCAACTTGTACAAGCTAAAAGTTGATCTCCCACTGGGCAGCCCATCTGTGACCTGCACCATCT
TGCCAGGGGGGATATCAGTGTCAAGTGCTATAGTGACTCAAATCAGTGATACTGAATTTGTCCTTGTCGGTGGCTACCAC
TCTGACAACGAGAAACGGTTGGTGTGTAACACCATAGTTCTGGAAGACAGTAAGATAAAGATTGTTGAAAGGGAGAGCCC
AGAGTGGACACCAGATATTAAACACTGCAGAATATGGTTTGGCTGTGATATGGGCAAAGGGTCTGTATTGCTGGGCATTC
CAGGGGCCAACAAACAGTTAATCTCAGATGCAAACTACTTCTACAYTTTGAGATGCAAACGAGAAGAAGAGGACAAGGAA
GAAGAATTGACAACACAAATTTGCAGTCAGACATCAAGCRAAGACCCTGGAGATTCCACTCCATTTGAAGACTCGGAGGA
GTTTTGTTTTAGTGCTGAAGCCAGTAGCTTTGACATTGATGATACTGACACTTACAATGAGGATGATGAAGAAGATGART
CAGAAACGGGCTACTGGATCACCTGCTGTGCC
Acanthisitta_chloris
AGATCCTGCCCCACTGGYGTTTTCCTTCTTGATATAACACAGAATGAGCTCAAAATGAAACCTGCCTTCTTCTCTAAAGA
CTCCTGTTACCTTCCCCCTCTCCGCTACCCTGCTCTTTGCACACTCAGAAGCAATGCAAAGTCTGACGAGTACCAGTATA
TCATCCATGGTGGTAAAACCCCTAACAATGACCTTTCTGATAAAATTTACATTATGAGTCTGGTCAGCAAAAATGGTAAG
AAAACCACATTCCAATGTGTTGAGAAAGACCTGGGTGGAGATGTCCCTGCAGCTAGATATGGGCACACAATTAATGTTGT
TCATAGYCGGGGAAAAAGCATGAGTGTTATATTTGGAGGGAGATCATATATTCCTCTTGCACAAAGAACTACTGAAAAAT
GGAACAGTGTAGTTGACTGTTTGCCATCTGTGTTTCTTGTTGATTTTGAGTTTGGATGTTGTACATCATACATACTTCCA
GAGCTTCAAGATGGACTTTCTTTCCATGTTTCAATTGCCAGAGATGATACAGTCTACATCTTGGGAGGCCATTCACTTCA

AAATAACACCAGGTCCCCCAGCTTGTACAAGCTAAAAGTTGATCTTCCACTGGGCAGCCCAGCTGTGACCTGCACCATCT
TGCCAGGGGGGATATCTGTGTCAAGTGCTATAGTGACTCAAATTAGTGATACCGAATTTGTCCTTGTTGGAGGTTACCAC
TCTGACAACCAGAAACGGTTGGTGTGTAACACCATAGTCCTGGAAGACAGTAAGATAGAGATTGTTGAAAGGGTGAGCCC
AGAGTGGACACCAGATATTAAGCACTGCAGAATATGGTTTGGCTGTGATATGGGCAATGGATCTGTATTGCTGGGCATTC
CAGGGGCCAACAAACAGTTACCCTCAGATGCAAACTACTTCTACATTTTGAGATGCAAAAGAGCAGAAGAGGAGAAGGAG
GAAGAACTGACATCACAAATGTGTAGTCAAACATCAACTGAAGACCCTGGAGACTCCACTCCATTTGAAGATTCAGAGGA
GTTTTGTTTTAGTGCTGAAGCCAATAGCTTTGACGTTGATGATACTGACACTTACAACGAAGATGATGAAGAAGATGAAT
CAGAAACAGGCTACTGGATCACCTGCTCTGCC
Regulus
AGATCCTGCCCCACTGGTGTTTTCCTCCTCGATATAAAGAAGAATGAGCTCAAGATGAAACCTGCCTTCTTCTCTAAAGA
CTCGTGTTACCTTCCCCCTCTCCGCTACCCTGCTGTTTGCACGCTCAGAAGTGATGCAAAGGCTGATGAGTACCAGTATA
TCATCCATGGTGGTAAAACACCTAACAATGACCTTTCTGATAAGATGTACTTTATGAATCTGGTAAGCAAAACTAGCAAG
AAAATGACATTCCAATGCATTGAGAAAGACCTGGGTGGAGATGTCCCTGAAGCTAGATATGGGCATACAATTAATGTAGT
TCATAGCCGGGGGAAAAGCATGAGTGTTCTGTTTGGAGGGAGGTCATATACTCCTCTTGCACAAAGAACCACTGAAAAAT
GGAACAGCGTAGTTGACTGTTTGCCATCTGTGGTTCTCATTGATTTTGAGTTTGGATGCTGTACATCATACATACTTCCA
GAGCTTCAAGATGGACTTTCTTTCCATGTTTCAATTGCCAGAGATGATACAATCTACATTTTGGGTGGCCACTCACTTCA
GAATAACACCAGACCCTCCAACTTGTACAAGCTAAAAATTGATCTGCCCCTGGGCAGCCCAGCTGTGACCTGCACCATCC
TGCRAGGGGGGATATCAGTGTCAAGTGCTATAGTGACCCAGATCAGTGACACTGAATTTGTCCTTGTCGGTGGCTATCAC
TCAGACAACCAGAAACGGCTGGCCTGTAACACCATCGTTCTGGAAGATGGTAAGATAGAGATTGYTGAAAGTGTGAGCCC
AGAGTGGACACCAGATATTAAACATAGCAGAATGTGGTTTGGCTGTGATATAGGYAAAGGGTCKGTTTTGCTGGGCATTC
CAGGGGCCAACAAGCAAATAATCCCAGATGCAAACTACTTCTACATTTTGAGATGCAAAGGAGCAGAAGAGGACAAGAAA
GAAGAATTAATAACACAAATTTGCAGTCAGACATCAAGCGAAGACCCTGGRGACTCCACTCCATTTGAAGATTCGGAGGA
GTTTTGTTTTAGTGCTGAAGCCAACAGCTTTGATGCTGACGATGCTGATACTTACAATGAAGATGATGAAGAAGATGAGT
CAGAAACAGGCTACTGGATCACCTGCTGTGCC
Ailuroedus
AGATCCTGCCCCACTGGTGTTTTCCTCCTCGATATAAAGCAGAATGAGCTCAAAATGAAACCTGCCTTCTTCTCTAAAGA
CTCATGTTACCTTCCCCCTCTCCGCTACCCTGCTCTTTGCCCACTCAGAAGCAATG---------
ACGAGTACCAGTATATCATCCATGGGGGTAAAACACCTAACAATGACCTTTCTGATAAGATTTACTTTATGAGCCTAGTA
AGCAAAAATAGCAAGAAAATGATGTTCCAGTGCATTGAGAAAGACCTGGGTGGAGATGTCCCTGAAGCTAGATATGGGCA
TACAATTAATGTAGTTCATAGCCGGGGGAAAAGCATTAGTGTCCTGTTTGGAGGGAGATCATATACTCCTCTCGCACAAA
GAACCACTGAAAAATGGAACAGCGTGGTTGACTGTTTGCCGTCTGTGTTTCTCATCGATTTTGAGTTTGGATGCAGTACA
TCATACATACTTCCAGAGCTTCAAGATGGACTTTCCTTCCATGTTTCAATTGCCAGAGATGATACAATCTACATTTTGGG
AGGCCATTCGCTTCAAAATAACACCAGGTCCCCTAACTTGTACAAGCTAAAAATTGACCTCCCACTGGGCAGTCCAGCTG
TGACCTGCACCGTCATGCCAGGGGGGATATCGGTGTCAAGTGCTATTGTGACTCAGATCAGTGATACTGAATTTGTCCTG
GTTGGTGGCTACCACTCTGACAACCAGAAACGGCTGGTGTGTAACACCATAGTTCTGGAAGACAGTAAGATAGAGATTGG
TGAAAGTGTGAGCCCAGAGTGGACACCAGATATTAAACACTGCAGGATATGGTTTGGCTGTGATATGGGTAAAGGGTCTA
TATTGCTGGGCATTCCAGGGGCCAACAAACAATTAATCCCAGATGCAAACTACTTCTACATTTTGAGATGCAAAGGAGCA
GAAGAGGGCAAGGAAGAAGAACTGACAACACAAATTTGCAGTCAGACGTCAAGTGAAGASCCGGGAGACTCCACTCCGTT
TGAAGATTCGGAGGAGTTTTGTTTTAGTGCTGAAGCCAATAGCTTTGACATGGATGATGCTGACACTTACAATGAAGATG
ATGAAGAAGATGAATCAGAAACAGGCTACTGGATCACCTGCTCTGCC
Zosterops
AGATCCTGCCCCACTGGTGTTTTCCTCCTTGATATAAAGCAGAATGAGCTCAAAATGAAACCTGCTGCCTTCTCTAAAGA
CTCGTGTTACCTTCCCCCTCTCCGCTACCCCGCTATTTGCACGCTCAGGAGCGATGCAGGGGCTGATGAGTACCAGTATG
TCATCCACGGGGGTAAAACACCTAACAATGAACTTTCTGATAAGATTTACTTTATGAGTCTGGTAAGCAAAACTAGCAAG
AAAATAACATTCCAGTGCCTTGAGAAAGACCTGGGTGGAGATGTCCCTGAAGCTAGATATGGGCATACAATTAATGTAGT
TCATAGCCGGGGAAAAAGCATGAGTGTTCTGTTTGGAGGCAGGACATATACTCCTCTTGCACAAAGAACCACTGAAAAAT
GGAACAGTGTAGTCGATTGTTTGCCATCTGTGTTTCTCATTGATTTTGAGTTTGGATGCTGTACATCATACATACTTCCA
GAGCTTCAAGATGGGCTTTCTTTCCACGTGTCACTTGCCAGAGATGATACAATCTACATCTTGGGAGGCCATTCACTTCA
AAATAACACCAGGTCCCCCAGCTTGTACAAGCTAAAAATTGATCTGCCCCTGGGCAGCCCGGCTGTGACCTGCACCATCC
TGCCAGGGGGGATATCAGTGTCAAGTGCTATAGTGACCCAGATCAGTGACACTGAATTCGTCCTTGTTGGTGGCTACCAC
TCAGACAGCCAGAAACGSCTGGCATGTCACACCATAGTTCTGGAAGATAATAAGATAGAGATTGTTGAAAGTGTGAGCCC
AGAGTGGACACCAGATATTAAACACTGCAGAATGTGGTTTGGTTGTGATATGGGTAAAGGGTCTGTTTTGCTGGGCATTC
CGGGGGCCAACAAACAAATAATCCCAGATGCAAACTACTTCTACATTTTGAGATGCAAAGGAGCAGATGAGGACAAGGAA
GAAGAATTGATAACACAAAATTGCAGTCAGACATCAAGTGAAGACCCTGGAGACTCTGCTCCRTTTGAAGATTCAGAGGA
ATTTTGTTTTAGTGCTGAAGCCAATAGCTTTGATGCTGATGATGCTGATACTTACAATGAAGATGATGAAGAGGATGAAT
CAGAAACAGGCTACTGGATCACTTGCTGTGCC
Onychorhynchus
AGATCCTGCCCCACTGGTGTTTTCCTCCTAGATATAAAGCAGAATGAGCTCAAAATGAAACCTGCCTTCTTCTCCAAAGA
CTCATGTTACCTTCCCCCTCTCCGCTACCCTGCTCTTTGCACACTCAGAAGCAATGCAAAGTCTGATGAGTACCAGTATA
TCATCCATGGTGGTAAAACACCTAACAATGACCTCTCTGATAAGATTTACTTTATAAGCCTGGTAAGCAAAAATACCAAG

AAAATGGTGCTTCGATGCATTGAGAAAGACCTGGGTGGAGATGTCCCTGAAGCTAGATATGGGCATACAATTAATGTAGT
TCATAGCCGGGGAAAAAGCATGAGTGTTATATTTGGAGGGAGATCGTATACTCCTCTCGCACAAAGAACCACTGAAAAAT
GGAACAGCGTAGTCGACTGTTTGCCATCTGTGTTTCTTATTGATTTTGAGTTTGGATGCTGTACATCATACATGCTTCCG
GAGCTTCAAGATGGTCTTTCTTTCCATGTTTCAATCGCCAGAGATGATACCATCTACATTTTGGGAGGCCATTCACTTCA
AAATAACACCAGGTGCCCCAACCTGTACAAGCTAAAAGTTGATCTCCCACTGGGCAGCCCAGCTGTGACCTGCACCATCC
TGCCAGGGGGGATATCAGTGTCAAGTGCTATAGTGACCCAAATCAGTGATACTGAATTTGTCCTTATCGGTGGCTACCAC
TCTGACAACCAGAAACGGTTGGTGTGTAACACCATAGTTCTGGAGGATAGTAAGGTAGAGATTGTTGAAAGGGAGAGYCC
GGAGTGGACACCTGATATTAAACACTGCAGGATATGGTTTGGCTGTGATATGGGCAAAGGGTCTGTATTGCTGGGCATTC
CAGGGGCCAACAAACAATTAACCTCAGATGCAAACTACTTCTACATTTTGGGATGCAAAGGAGAAGAAAAGGACAAGGAA
GAAGAATTGACAACACAGATTTGCAGTCAGACATCAAGCGAAGACCCTGGAGATTCCACTCCATTTGAAGATTCGGAGGA
GTTTTGTTTTAGTGCTGAAGCCAATAGCTTTGACATTGATGATGCTGACACTTACAATGAGGATGATGAAGAAGATGAAT
CAGAAACGGGCTACTGGATCACCTGCTGTGCC
Nestor_notabilis
AGGTCCTGTCCCACTGGTGTTTTCCTCCTCGATATAAAGGAGAATGAGCTCAAAATGAAACCTGCCTTTTTCTCTAATGA
CTCCTGTTACCTGCCCCCTCTCCGCTACCCTGCCCTTTGCACACTCAGAGGCAATGCGCAGTCTGATGAGTACCAGTATA
TCATCCATGGTGGAAAAACGCCTAACAATGACCTTTCAGATAAGATTTATATTATGAGTCTGATAAGCAAAAATAGCAAG
AAAACCACATTTCAATGCGTTGAGAAAGACCTGGATGGAGATGTTCCTGAAGCTAGATACGGACATACAATTAACGTAGT
TCATAGCCGTGGAAAAAGCATGAGCGTTATATTTGGAGGGAGATCATATACCCCTCTTGCACAAAGAACCACTGAAAAAT
GGAACAGTGTAGTTGACTGTTTGCCATCTGTGTTTCTCATTGATTTCGAGTTTGGATGCTGTACATCATACATACTTCCA
GAGCTTCAGGATGGACTTGCTTTCCATGTTTCAGTTGCCAGAAATGATACAATCTACATTCTGGGAGGCCATTCACTTCA
AAATAACACCAGGTCCCCCAGCTTGTACAAGCTAAAAGTTGATCTCCCGCTGGGCAGCCCAGCCGTGACCTGCACCATCT
TGGCAGGGGGGATATCTGTGTCGAGTGCTATATTGACTCAAACCAGTGATACCGAATTTGTCCTTGTTGGGGGCTACGAG
TCTGACAAGCAGAAACGGTTGGTATGTAACACCATAGTCCTGGAAGACAGTAATATACAGATTGTTGAAAGGGAGAGCCC
AGACTGGACACCAGATATTAAACACTGCAGGATGTGGTTTGGCAGTGATATGGGCAAAGGATCTGTACTGCTGGGCATTC
CAGGGGCCAACAAACAGTTAATCTCAGATGCAAACTACTTCTACATTTTGAGATGCAAGGGAGCAGAAGAGGACAAGGAG
GAAGAACTGACAGCACAAATTAGCAGTCAGACATCTACTGAAGACCCTGGAGACTCCACTCCATTTGAAGATTCTGAAGA
GTTTTGTTTCAGTGCTGAAGCCAATAGCTTTGATGTTGATGATACTGACACTTACAATGAAGATGATGAAGAAGATGAAT
CAGAAACAGGCTACTGGATCATCTGCTCCACC
Leiothrix_argentauris
AGATCCTGCCCCACTGGTGTTTTCCTCCTCGATATAAAGCAGAATGAACTCAAAATGAAACCTGCCTTCTTCTCTAAAGA
CTCATGTTACCTTCCCCCTCTCCGCTACCCTGCTCTTTGCACGCTCAGAAGCGATGCAAGGGCTGATGAGTACCAGTATA
TCATCCATGGTGGTAAAACACCTAACAATGACCTTTCTGATAAGATTTACTTTATGAGTCTGGTGAGCAAAAGCAGCAAG
AAAATGACATTCCAATGCATTGAGAAAGACCTTGGTGGAGATGTCCCTGAAGCTAGATATGGGCATACGATTAATGTAGT
TCATAGCCGAGGAAAAAGCATGAGTGTTCTGTTTGGAGGGAGGTCGTATACTCCTCTTGCACAAAGAACCACTGAGAAAT
GGAACAGCGTAGTCGACTGTTTGCCATCTGTGTTTCTCATTGATGTTGAGTTTGGGTGCTGTACATCGTATATACTTCCA
GAGCTTCAAGACGGACTTTCTTTCCATGTTTCAATTGCCAGAGGTGATACAATCTACATCTTGGGAGGCCACTCACTTCA
GAATAACACCAGGTCCCCCAACTTGTACAAACTAAAAATTGATCTGCCCCTGGGCAGCCCGGCTGTGACCTGCACCATCC
TGCCAGGGGGGATATCAGTGTCAAGTGCTATAGTGACCCAGATCAGTGACACTGAATTTGTCCTTGTTGGTGGCTACCAC
TCAGACAACCAGAAACGGCTGRCATGCAACACCATAGTTCTGGAAGACAGTAAGATAGAGATTGTCGAAAGTGTGAGCCC
AGAGTGGACACCAGATATTAAACACTGCAGAACATGGTTTGGCTGTGATATGGGAAAAGGKTCTGTTTTGCTGGGCATTC
CAGGGGCCACCAAACAAATAATCCCAGATGCAAACTACTTCTACATTTTGAGATGCAAAGGAGCAGAAGAGGACAAGGAA
GAAGAATTGATTACACAAACTTGCAGTCAGACATCAAGTGAAGACCCTGGAGACTCCACTCCATTTGAAGATTCGGAGGA
GTTTTGTTTTAGTGCTGAAGCCAATAGCTTTGATGCTGATKATGCTGATACTTACAATGAAGATGACGAAGAAGATGAAT
CAGAAACAGGCTACTGGATCACCTGCTGTGCC
Troglodytes
AGATCCTGCCCCACTGGTGTTTTCCTCCTCGATATAAAGCAGAATGAACTCAAAATGAAACCTGCCTTCTTCTCTAAAGA
CTCATGTTACCTTCCCCCTCTCCGCTACCCTGCTCTTTGCACGCTCAGAAGCGATGCAAGGGCTGATGAGTACCAGTATA
TCATCCATGGTGGTAAAACACCTAACAATGACCTTTCTGATAAGATTTACTTTATGAGTCTGGTGAGCAAAAGCAGCAAG
AAAATGACATTCCAATGCATTGAGAAAGACCTTGGTGGAGATGTCCCTGAAGCTAGATATGGGCATACGATTAATGTAGT
TCATAGCCGAGGAAAAAGCATGAGTGTTCTGTTTGGAGGGAGGTCGTATACTCCTCTTGCACAAAGAACCACTGAGAAAT
GGAACAGCGTAGTCGACTGTTTGCCATCTGTGTTTCTCATTGATGTTGAGTTTGGGTGCTGTACATCGTATATACTTCCA
GAGCTTCAAGACGGACTTTCTTTCCATGTTTCAATTGCCAGAGGTGATACAATCTACATCTTGGGAGGCCACTCACTTCA
GAATAACACCAGGTCCCCCAACTTGTACAAACTAAAAATTGATCTGCCCCTGGGCAGCCCGGCTGTGACCTGCACCATCC
TGCCAGGGGGGATATCAGTGTCAAGTGCTATAGTGACCCAGATCAGTGACACTGAATTTGTCCTTGTTGGTGGCTACCAC
TCAGACAACCAGAAACGGCTGRCATGCAACACCATAGTTCTGGAAGACAGTAAGATAGAGATTGTCGAAAGTGTGAGCCC
AGAGTGGACACCAGATATTAAACACTGCAGAACATGGTTTGGCTGTGATATGGGAAAAGGKTCTGTTTTGCTGGGCATTC
CAGGGGCCACCAAACAAATAATCCCAGATGCAAACTACTTCTACATTTTGAGATGCAAAGGAGCAGAAGAGGACAAGGAA
GAAGAATTGATTACACAAACTTGCAGTCAGACATCAAGTGAAGACCCTGGAGACTCCACTCCATTTGAAGATTCGGAGGA
GTTTTGTTTTAGTGCTGAAGCCAATAGCTTTGATGCTGATKATGCTGATACTTACAATGAAGATGACGAAGAAGATGAAT
CAGAAACAGGCTACTGGATCACCTGCTGTGCC

Luscinia_svecica
??
??
??
??
??
??
??
??
??
??
??
??
??
??
????????????????????????????????
Psittacus_erithacus
AGGTCTTGTCCTACTGGTGTTTTCCTCCTCGATATAAAGGAGAATGAGCTCAAAATGAAACCTGCCTTCTTCTCTAATGA
CTCCTGTTACCTGCCTCCTCTCCGCTACCCTGCCATTTGCAAGCTCAGAGGCAATGCAGAGTCTGATGAGTACCAGTATA
TAATCCATGGTGGAAAAACGCCTAACAATGACCTTTCGAATAAGATTTATGTTATGAGTCTGATAAGCAAAAATAGCAAG
AAAACCACATTTCAATGTATTGAGAAAGACCTGTGTGGAGATGTTCCTGAAGCTAGATACGGGCATACAATTAACATAGT
TCATAGTCAGGGAAAAAGCATGAGTGTTATATTTGGAGGGAGATCATATACCCCTCTTGCACAAAGAACCACTGAAAAAT
GGAACAGTGTAGTTGACTGTTTGCCATCTGTGTTTCTCGTTGATTTTGAGTTTGGATGCTGTACATCATACATACTTCCA
GAGCTTCAGGACGGACTTGCTTTCCACGTTTCAGTTGCCAGAAATGATACAATATACATTTTGGGAGGCCATTCAATTCA
AAGTAACACCAGGTCCCCCAGCTTGTACAAGCTAAAAGTTGATCTCCCGCTGGGCAGCCCAGCCGTGACCTGCACCATCT
TGCCAGGGGGGATATCTGTGTCGAGTGCTATAGTGACTCAAACCAGTGATACCGAATTTGTCCTTGTTGGGGGCTACCAG
TCTGACAACCAGAAACGGTTGGTATGTAACACCATAGTTCTGGAAGACAGCAATATACAGATTGTTGAAAGGGAGAGCCC
AGACTGGACACCAGATATTAAACACTGCAGGATGTGGTTTGGCAGTGATATGGGCAAAGGGTCTGTATTGCTGGGCATTC
CAGGGGGCAATAAACAGTTAATCTCAGATGTGAACTACTTTTATATTTTGAGATGCAAAGG---
AGAAAAGGACAAGGAAGAAGAACTGACAGCACAAATTTGCAGTCAGACATTGACTGAAGACCCTACAGACTCCACTCCAT
TTGAAGATTCTGAGGAGTTTTGTTTCAGTGCTGAAGCCAATAGATTTGATGTTGATTATATTGACACTTACCATGAAGAT
GATGAAGAAGATGAATCAGAAACAGGCTACTGGATCACCTGCTCTGCA
Ficedula_hypoleuca
??
??
??
???TACAATTAATGTMGT
TCATAGCCGGGGAAAAAGCATGGCTGTTCTGTTTGGAGGGAGGTCGTATACTCCTCTTGCACAAAGAACCACTGAAAAAT
GGAACAGTGTAGTTGACTGTTTACCATCTGTGTTTCTCATTGATTTTGAGTTTGGATGCTGTACATCATACATACTTCCA
GAGCTTCAAGATGGGCTTTCCTTCCACGTCTCAATTGCCAGAGATGATACAATCTACATCTTGGGAGGCCACTCACTTCA
AAATAACACCAGGTCCCCCAACTTRTACAAGGTAAAAATTGATCTGCCCCTGGGCAGCCCRGCTGTGACCTGCACCATCC
TGCCAGGGGGGRTATCTGTGTCAAGTGCTATAGTGACGCAGATAAGTGATACTGAATTTGTCCTTGTCGGTGGCTACCAC
TCAGACAGCCAGAAACGGCTGGTGTGTAACACCGTAGTTCTGGAAGACAGTAGGATRGAGATTGTTGAAAGTGTGAGCCC
AGAGTGGACACCRGACATTAAACACTGCAGAACGTGGTTTGGCTGTGATATGGGTAAAGGGTCTGTTTTGCTGGGCATTC
CAGGGGCCAACAAACAAATAATCCCAGATGCAAACTACTTCTACATTTTGAGATGCAAAGGGGCAGAAGAGGACAAGGAA
GAAGAATTGATAGCACGAATTTGCAGTCAGACATCAAGTGAAGACCCTGGAGACTCCACTCCATTTGAAGATTCGGAGGA
GTTTTGCTTTAGTGCTGAAGCCAATAGCTTTGATGCTGACGATGCTGATACTTACAATGAAGATGACGAAGAAGATGAAT
CAGAAACAGGCTACTGGATCACCTGCTGTGCC
Menura_novaehollandiae
AGATCCTGCCCCACTGGTGTTTTCCTCCTCGATATAAAGCAGAATGAGCTCAAAATGAAACCTGCCTGCTTCTCTAAAGA
CTCATGTTACCTTCCGCCTCTCCGCTACCCTGCTCTTTGCACGCTCAGAAGCAATGCAAGGTCTGATGAGTACCAGTACA
TCATCCATGGTGGTAAAACACCTAACAATGACCTTTCTGATAAGATTTACTTTATGAGTCTGGTAAGCAAAAATAGCAAG
AAAATGACATTCCAATGCAGTGAGAAAGACCTGGGTGGAGATGTCCCTGAAGCTAGATATGGGCATACAATTAATGTAGT
TCATAGCCGGGGAAGAAGCATGAGTGTTATATTTGGAGGGAGATCATATACTCCTCTTGCACAAAGAACCACTGAAAAAT
GGAACAGTGTAGTTGACTGTTTGCCATCTGTGTTTCTCATTGATTTTGAGTTTGGATGCTGTACTTCATACATACTCCCA
GAGCTTCAAGATGGGCTTTCTTTCCACGTTTCAATTGCCAGAGATGATACAATCTACATTTTGGGAGGCCATTCACTTCA
AAACAACACCAGGTCCCCCAACTTGTACAAGCTAAAAATTGATCTTCCCCTGGGCAGCCCAGCCGTGACCTGCACCATCT
TGCCAGGGGGGATATCAGTGTCGAGTGCTATAGTGACTCAGATCAGTGATACTGAATTTGTCCTTGTTGGTGGCTACCAC
TCTGACAACCAGAAACGGCTGGTGTGTAACACCATAGTTCTGGAAGACAGTAAGATAGAGATTGTTGAAAGTGTGAGCCC
AGAGTGGACACCAGATATTAAACACTGCAGAATGTGGTTTGGCTGTGACATGGGTAAAGGGTCTGTATTGCTGGGCATTC

CAGGGGCCAACAAACAATTAATCCCAGATGCAAACTACTTCTACATTTTGAGATGCAAAGGAGCAGAAGAGGACAAGGAA
GAAGAATTGACAACACAAATTTGCAGTCAGACATCAAGCGAAGACCCTGGAGACTCCACTCCTTTTGAAGATTCGGAGGA
GTTTTGTTTTAGTGCTGAAGCCAATAGCTTTGACATCGATGATACTGACACTTACAATGAAGATGATGAAGAAGATGAAT
CAGAAACGGGCTACTGGATCACCTGCTCTGCC
Pycnonotus
AGATCCTGCCCCACTGGTGTTTTCCTGCTTGATATAAAGCCGAATGAGCTCAAAATGAAACCTGTCTTCTTCTCTAAAGA
CTCATGTTACCTTCCCCCTCTCCGCTACCCTGCTCTTTGCACACTCAGAAGTGATGCAGGGGCTGATGAGTACCAGTATA
TCATCCATGGTGGTAAAACACCCAACAATGAACTTTCTGATAAGATTTACTTTATGAGTCTGGTAAGCAAAACTAGCAAG
AAAATAACATTCCAATGCATTGAGAAAGACCTAGGTGGAGATGTCCCTGAAGCTAGATACGGGCATACAATTAATGTAGT
TCAYAGCCGGGGAAAAAGCATGAGTGTTCTGTTTGGAGGGAGGACACTTGCTCCTCTTGCACAAAGAACCACTGAAAAAT
GGAACAGCGTAGTTGACTGTTTTCCATCTGTGTTTCTCATTGATTTTGAGTTTGGATGCTGTACATCATACATACTTCCA
GAGCTTCAAGATGGACTTTCTTTCCATGTGTCAATTGCCAGAGATGATACCATCTACATCTTGGGAGGCCACTCACTTCA
AAATAACACCAGGTCCCCCAACTTGTACAAGCTAAAAATTGATCTGCCCCTAGGCAGCCCGGCTGTGACCTGCACCATCC
TGCCAGGGGGGATATCAGTGTCAAGTGCTATAGTGACCCAGATCAGTGACACTGAATTTGTCCTTGTTGGTGGCTACCAC
TCAGACAACCAGAAACGGCTGGCATGTAACACCATAGTTGTGGAAGATGATAAGATAGAGATTGTTGAAAGTRTGAGCCC
AGAGTGGACACCAGATATTAAACACTGCAGAATGTGGTTTGGCTGTGATATGGGCAAAGGATCTGTTTTGCTGGGCCTTC
CAGGGGCCAACAAACAAGTAATCCCAGATGCAAACTACTTCTACATTTTGAGATGCAAAGGACCAGAAGAGGACAAGGAA
GAAGAATTGATAACACAAATTTGCAGTCAGACATCAAGTGAAGACCCTGAAGACTCTGCTCCATTTGAAGATTCGGAGGA
GTTTTGTTTTAGTGCAGAAGCCAGTAGCTTTGATGCTGACAATGCTGATACTTACAATGAAGATGATGAAGAGGATGAAT
CAGAAACAGGCTACTGGATCACCTGCTGTGCC
Donacobius_atricapilla
??
??
??
??
??
??
??
??
??
??
??
??
??
??
????????????????????????????????
Hirundo_rustica
AGATCCTGCCCCACTGGTGTTTTCCTCCTCGATATAAAGCAGAATGAGCTCAAAATGAAACCTGCCTTCTTCTCTAAAGA
CTCGTGTTACCTTCCCCCTCTCCGCTACCCTGCTCTTTGCACGCTCAGAAGCGATGTGAAGGCTGATGAGTACCAGTATA
TCATCCATGGTGGTAAAACACCTAACAATGAACTTTCTGATAAGGTTTACTTTATGAGTCTGGTAAGCAAAACTAGCAAG
AAAATAACATTCCAATGCATTGAGAAAGACCTGGGTGGAGATGTCCCTGAAGCTAGATATGGGCATACAATTAATGTAGT
TCATAGCCGGGGGAAAAGCRTGAGTGTTCTGTTTGGAGGGAGGACGTATACTCCTCTTGCACAAAGAACCACTGAGAAAT
GGAACAGCGTAGTCGACTGTGTGCCATCCGTGTTTCTCATTGATTTTGAGTTTGGATGCTGTACATCATACATACTTCCA
GAGCTTCAAGATGGACTTTCTTTCCACGTGTCAATTGCCAGAGATGATACAATCTACATCTTGGGAGGCCACTCACTTCA
GAATAACACCAGGTCCCCCAACTTGTACAAGCTAAAAATTGATCTGCCCCTGGGCAGCCCAGCTGTGACCTGCACCATCC
TGCCAGGGGGGATATCAGTGTCAAGTGCTATAGTGACCCAGATCAGTGACGCTGAATTTGTCCTTGTTGGTGGCTACCAC
TCAGACAACCAGAAACGGCTGGTGTGTAACACCATAGTAATGGAAGATAGTAAGATAGAGATTGTTGAAAGTGTGAGCCC
AGAGTGGACACCAGATATTAAACACTGCAGAATGTGGTTTGGCTGTGATATGGGTAAAGGGTCTGTTTTGCTGGGCATTC
CAGGGGCCAACAAACAAATAATCCCAGATGCAAACTACTTCTACATTTTGAGGTGCAAAAG---
AGAAGAGGATAAGGAAGAAGAATTGAYAACACAAAWTTGCAGCCAGACGTCAAGTGAAGATGCTGGAGACACTGCTCCAC
TTGAAGATTCGGAGGAGTTTTGTTTTAGTGCTGAAGCCAATAGCTTTGATGCTGATGATGCTGATACTTACAATGAAGAT
GATGAAGAGGATGAATCAGAGACAGGCTACTGGATCACCTGTTGTACC
Lichenostomus
??
??
??
??
??
??
??

??
??
??
??
??
??
??
????????????????????????????????
Cnemophilus
AGGTCCTGCCCCACTGGTGTTTTTCTCCTTGATGTAAAGCAGAATGAACTCAAAATGAAACCTGCCTTCTTCTCTAAAGA
CTCGTGTTACCTTCCCCCTCTCCGCTACCCTGCTCTTTGCACTCTCAGAAGCGATGCAAGGGCTGATGAGTACCAGTACA
TCATCCATGGTGGTAAAACACCTAACAATGACCTTTCTGATAAGATTTACTTTATGAGTCTGGTAAGCAAAACTAGCAAG
AAAATGACGTTCCAATGCATTGAGAAAGACCTGGGTGGAGATGTCCCTGAAGCTAGATATGGGCATACAATTAATGTAGT
TCACAGCCGAGGAAAAAGCGTGAGTGTTCTGTTTGGAGGGAGGTCGTATACTCCTCTTGCACAGAGAACCACTGAAAAAT
GGAACAGCGTAGTTGACTGTTTGCCATCTGTGTTTCTCATTGATTTTGAGTTTGGATGCTGTACATCATACATACTTCCA
GAGCTTCAAGATGGACTTTCTTTCCATGTTTCAATTGCTAGAGGTGATACAATCTACATCTTGGGAGGCCACTCACTTCA
AAATAACACCAGGCCCCCCAACTTGTACAAGCTAAAAATTGATCTGCCCCTGGGCAGCCCAGCTGTGACCTGTACCATCC
TGCCGGGAGGGATATCGGTGTCAAGTGCTATAGTGACCCAGATCAGTGACACTGAATTTGTCCTTGTCGGTGGCTACCAC
TCTGACAACCAGAAACGGTTGGTGTGTAACACCATAGTTCTGGAAGACAGTAAGATAGAGATTGTTGAAAGTGTGAGCCC
AGAGTGGACACCAGATATTAAACACTGCAGAATGTGGTTTGGCTGTGATATGGGTAAAGGGTCCGTTTTGCTGGGCATTC
CAGGGGCCAACAAACAATTAATYCCAGATGCAAACTACTTCTACATTTTGAGATGCAAAAGAGAAGAAGAGGACAAGGAA
GAAGAATTGACAACACAAATTTGCAGTCAGACCTCAAGTGAAGATCCTGGAGACTCCACTCCATTTGAAGATTCGGAGGA
GTTTTGTTTTAGTGCTGAAGCCAATAGCTTTGATGTTGATGATGCTGACACTTACAATGAAGATGATGAAGAAGATGAAT
CAGAAACGGGCTACTGGATCACCTGCTGTGCC
Coracina
AGATCCTGCCCCACTGGTGTTTTCCTCCTCGATATAAAGCAGAATGAGCTCAAAATGAAACCTGCCTTCTTCTCTAAAGA
CTCGTGTTACCTTCCCCCTCTCCGCTACCCCGCTCTTTGCACGCTCAGAAGCGATGCAAGGGCTRATGAGTACCAGTATA
TCATCCATGGTGGTAAAACACCTAACAATGACCTTTCTGATAAGATTTACTTTATGAGTCTGGTAAGCAAAACTAGCAAG
AAAATGACGTTCCAATGCATTGAGAAAGACCTGGGTGGAGATGTCCCTGAAGCTAGATATGGGCATACAATTAATGTAGT
TCATAGCCGGGGAAAAAGCATGAGTGTTCTGTTTGGAGGGAGGTCATATACTCCTCTTGCACAAAGAACCACTGAAAAAT
GGAACAGCGTAGTTGACTGTTTGCCATCTGTGTTTCTCATTGATTTTGAGTTTGGATGCTGTACATCATACATACTTCCA
GAGCTTCAAGATGGACTTTCTTTCCACGTTTCAATTGCCAGAGATGATACAATCTACATCTTGGGAGGCCACTCACTTCA
AAATAACACCAGGTCCCCCAACTTGTACAAGATAAAAATTGATCTGCCCCTGGGCAGCCCAGCTGTGACCTGCACCATCC
TGCCAGGGGGGTTATCGGTGTCAAGTGCTATAGTGACCCAGATCAGTGATACTGAATTTGTCCTTGTCGGTGGCTACCAC
TCTGACAACCAGAAACGGTTGGTGTGTAACACCATAGTTCTGGAAGACAGTAAGATAGAGATTGTTGAAAGTGTGAGCCC
AGAGTGGACACCAGATATTAAACACTGCAGAATGTGGTTTGGCTGTGATATGGGTAAAGGGTCTGTTTTGCTGGGCATTC
CAGGGGCCAACAAACAATTAATCCCAGATGCAAACTACTTCTACATTTTGAGATGCAAAGGAGCAGAAGAGGACAAAGAA
GAAGAATCAATAACACAAATTTGCAGTCAGACGTCAAGTGAAGACCCTGGAGACTCCACTCCATTTGAAGACTCGGAGGA
GTTTTGTTTTAGTGCTGAAGCCAATAGCTTTGACGTTGACGATGCTGACACTTACAATGAAGATGATGAAGAAGATGAAT
CAGAAACGGGCTACTGGATCACTTGCTGTGCC
Dicrurus
AGATCGTGCCCCACTGGTGTTTTCCTCCTCGATATAAAGCAGAATGAGCTGAAAATGAAACCGGCCTTCTTCTCTAAAGA
CTCGTGTTACCTTCCCCCTCTCCGCTACCCCGCTCTTTGCACCCTCAGAAGCGATGCAAAGGCTGATGAGTACCAGTATA
TCATCCATGGTGGTAAAACACCTAACAATGACCTTTCTGATAAGATTTACTTTATGAGTCTGGTAAGCAAAGCTAGCAAG
AAAATGACGTTCCAATGCATTGAGAAAGACCTGGGTGGAGATGTCCCTGAAGCTAGATATGGGCATACAATTAATGTAGT
TCATAGCCGGGGAAAAAGCATGAGTGTTCTGTTTGGAGGGAGGTCGTATACTCCTCTTGCACAAAGAACCACTGAAAAAT
GGAACAGTGTAGTTGACTGTTTGCCATCTGTGTTTCTCATTGATTTTGAATTTGGATGCTGTACATCATACATCCTTCCA
GAGCTTCAAGATGGACTTTCTTTCCACGTTTCAGTTGCCAGAGATGATACAATCTACATCTTGGGAGGCCACTCACTTCA
AAATAACACCAGGTCTCCCAACCTGTACAAGCTAAAAATTGATCTGCCCCTGGGCAGCCCAGCTGTGACCTGCACAATCC
TGCCAGGGGGGGTATCGGTGTCAAGCGCTATAGTGACCCAGATCAGCGACACTGAATTTGTCCTTGTCGGTGGCTACCAC
TCTGACAACCAGAAACGGTTGGTGTGTAACACCATAGTTCTGGAAGATAGTAAGATAGAGATTGTTGAAAGTGTGAGCCC
AGAGTGGACACCAGATATTAAACACTGCAGAACGTGGTTTGGCTGTGATATGGGTAAAGGGTCTGTTTTGCTGGGCATTC
CAGGGGCCAACAAACAATTAAGCCCAGATGCAAACTACTTTTACATTTTGAGATGCAAAGGAGCAGACGAGGACAAGGAA
GAAGAATTGATAACACAGATTTGCAGTCAGACATCAAGTGAAGACCCTGGAGACTCCACTCCATTTGAAGACTCGGAGGA
GTTTTGTTTTAGTGCTGAAGCCAATAGCTTTGACGTTGATGATGCTGACACTTACAATGAAGATGATGAAGATGATGAAT
CAGAAACGGGCTACTGGATCACCTGCTGTGCC
Gerygone_fusca
?????????????????????????????GATATAAAGCAGAATGAGCTCAAAATGAAACCTGCCTTCTTCTCCAAAGA
CTCRTGTTACCTTCCCCCTCTCCGTTACCCCGCTCTTTGCACACTCAGAAGAGGTGCAAGGTCTGATGAGTTCCAGTACA
TAATCCATGGTGGTAAAACACCTAACAATGACCTTTCTGATAAGATTTACGTTATGAGTCTGGTAAGCAAAACTAGCAAA

AAAATGATATTCCAATGCATTGAGAAAGACCTGGGTGGGGATGTCCCTGAAGCCAGGTATGGGCATACAATTAATGTAGT
TCATAGCCGGGGAAAAAGCGTGAGTGTTCTATTTGGAGGGAGATCATATACTCCTCTTGCACAAAGAACCACTGAAAAAT
GGAACAGTGTAGTTGACTGTTTGCCATCTGTGTTTCTCATTGATTTTGAGTTTGGATGCTGTACATCGTACATACTKCCA
GAGCTTCAAGATGGGCTTTCTTTCCAYGTTTCAATTGCCAGAGATGATACAATCTACATCTTGGGAGGCCATTCACTCCA
GAATAATACCAGGTCCCCCAACCTGTACAAGCTCAAAATCGATCTGCCCCTGGGCAGCCCAGCTGTGACCTGCACCGTCC
TGCCAGGGGGGATATCGGTGTCAAGTGCTATAGTGACCCAGATCAGTGACACTGAATTTGTCCTTGTCGGTGGCTACCAC
TCTGACAACCAGAAACGRTTGGTATGTAACACCATAGTTCTAGAAGAGAGTAAGATAGAGATTGTTGAAAGTGTGAACCC
AGAGTGGACACCAGATATTAAACACTGCAGAATGTGGTTTGGCTGTGATATGGGGAAAGGGTCTGTATTGCTGGGCATTC
CAGGGGCCAGCAAACARTTAATCCCAGATGCAAACTACTTCTACATTTTGAGATGCAAAGGAGCAGAAGAGGACAAGGAA
GAAGAACTGGTAACACAAATTTGCAGTCAGACATCAAGTGAAGACCCTGGAGACTCCACTCCATTTGAAGATTCAGAGGA
GTTTTGTTTTAGTGCTGAAGCCAATAGCTTTGACATTGATGATGCTGATACT????????????????????????????
????????????????????????????????
Ptiloris_magnificus
AGATCCTGCCCCACTGGTGTTTTCCTCCTCGATATAAAGCAGAATGAGCTCAAAATGAAACCTGCCTTCTTCTCTAAAGA
CTCGTGTTACCTTCCCCCTCTCCGCTACCCCGCCCTTTGCACGCTCAGAAGCGATGCAAAGGCTGATGAGTACCAGTATA
TCATCCATGGTGGTAAAACACCTAACAATGACCTTTCTGATAAGATTTACTTTATGAGTCTGGTAAGCAAAACTAGCAAG
AAAATGACATTCCAATGCATTGAGAAAGACCTGGGTGGAGATGTCCCTGAAGCTAGATATGGGCATACAATTAATGTAGT
TCATAGCCGGGGAAAAAGCATGAGTGTTCTGTTTGGAGGGAGGTCATACACTCCTCTTGCACAAAGAACCACTGAAAAAT
GGAACAGTGTAGTTGACTGTTTGCCATCTGTGTTTCTCATTGATTTTGAGTTTGGATGTTGTACATCATACATCCTTCCA
GAACTTCAAGATGGACTTTCTTTCCACGTTTCAATTGCCAGAGATGATACAATCTACATCTTGGGTGGCCACTCACTTCA
AAATAACACCAGGTCCCCCAACTTGTACAAGGTAAAAATTGATCTGCCCCTGGGCAGCCCAGCTGTGACCTGCACCATCC
TGCCAGGGGGGGTATCGGTGTCAAGTGCTATAGTGACCCAGATCAGTGATACTGAATTTGTCCTTGTCGGTGGCTACCAC
TCTGACAACCAGAAACGGTTGGCGTGTAACACCATAGTTCTAGAAGATAGTAAGATAGAGATTGTTGAAAGTGTGAGCCC
AGAGTGGACACCAGACATTAAACACTGCAGGACGTGGTTTGGCTGTGATATGGGTAAAGGGTCTGTTTTGCTGGGCATTC
CAGGGGCCAACAAACAATTAAGCCCGGATGCAAACTACTTTTACATTTTGAGATGCAAAGGAGCAGAAGAGGACAAGGAA
GAAGAATCGATAACACAAATTTGCAGTCAGACGTCAAGTGAAGACCCTGGAGACTCCACTCCATTTGAAGACTCGGAGGA
GTTTTGTTTTAGTGCTGAAGCCAATAGCTTTGATGTTGACGATGCTGACACTTACAATGAAGATGATGAAGAAGATGAAT
CGGAAACGGGCTACTGGATCACCTGCTGTGCC
Paradisaea_raggiana
AGATCCTGCCCCACTGGTGTTTTCCTCCTCGATATAAAGCAGAATGAGCTCAAAATGAAACCTGCCTTCTTCTCTAAAGA
CTCATGTTACCTTCCCCCTCTCCGCTACCCCGCTCTTTGCACGCTCAGAAGCGATGCAAAGGCTGATGAGTACCAGTATA
TCATCCATGGTGGTAAAACACCTAACAATGACCTTTCTGATAAGATTTACTTTATGAGTCTGGTAAGCAGAACTAGCAAG
AAAATGACGTTCCAATGCATTGAGAAAGACCTGGGTGGAGATGTCCCTGAAGCTAGATATGGGCATACAATTAATGTAGT
TCATAGCCGGGGAAAAAGCATGAGTGTTCTGTTTGGAGGGAGGTCGTACACTCCTCTTGCACAAAGAACCACTGAAAAAT
GGAACAGTGTAGTTGACTGTTTGCCATCTGTGTTTCTCATTGATTTTGAGTTTGGATGTTGTACATCATACATCCTTCCA
GAACTTCAAGATGGACTTTCTTTCCACGTTTCAATTGCCAGAGATGATACAATCTACATCTTGGGTGGCCACTCACTTCA
AAATAACACCAGGTCCCCCAACTTGTACAAGGTTAAAATTGATCTGCCCCTGGGCAGCCCAGCTGTGACCTGCACCATCC
TGCCAGGGGGGGTATCGGTGTCAAGTGCTATAGTGACCCAGATCAGTGATACTGAATTTGTCCTTGTCGGTGGCTACCAC
TCTGACAACCAGAAACGGCTGGTGTGTAACACCATAGTTCTGGAAGACAGTAAGATAGAGATTGTTGAAAGTGTGAGCCC
AGAGTGGACACCAGACATTAAACACTGCAGAACGTGGTTTGGCTGTGATATGGGTAAAGGGTCTGTTTTGCTGGGCATTC
CAGGGGCCAACAAACAATTAAGCCCAGATGCAAACTACTTTTACATTTTGAGATGCAAAGGAGCAGAAGAGGACAAGGAA
GAAGAATCRATAACACAAATTTGCAGTCAGACGTCAAGTGAAGACCCTGGAGACTCCACTCCATTTGAAGACTCGGAGGA
GTTTTGTTTTAGTGCTGAAGCCAATAGCTTTGAYGTTGATGATGCTGACACTTACAATGAAGATGATGAAGAAGATGAAT
CGGAAACGGGCTACTGGATCACCTGCTGTGCC
Cyanocorax_chrysops
??
??
??
??
??
??
??
??
??
??
??
??
??
??
????????????????????????????????

Lonchura
??
??
??
??
??
??
??
??
??
??
?????????????????????????????????TGTGGTTTGGCTGTGATATGGGTAAAGGGTCTGTTTTGCTGGCATTC
CAGGGGCMAGCAAACAAATAATCCCAGATGCAAACTACTTCTACATTTTGAGATGCAAAGGAGCAGAAGAGGACAAGGAA
GAGGAATTGATAACACAAATTTGCAGTCAGACATCAAGTGAAGACCCTGGAGACTCCACTCCATTCGAAGACTCAGAGGA
GTTTTGTTTTAGTGCTGAAGCCAATAGCTTTGATGCTGACGATGCTGATATGTACAATGAAGATGATGAAGAAGATGAAT
CATAA???????????????????????????
Phylloscopus
AGATCCTGCCCCACTGGTGTTTTCCTCCTCGATATAAAGCAGAATGAGCTCAAAATGAAGCCCGCCACCTTCTCTAAAGA
CTCATGTTACCTTCCCCCTCTCCGCTACCCGGCTCTTTGCACGCTCAGAAGCGATGGAAGGGCTGATGAGTACCAGTATA
TCATCCATGGTGGTAAAACACCTAACAATGAACTTTCTGATAAGATTTACTTTATGAGTCTGGTAAGCAAAACTAGCAAG
AAAATAACATTCCAATGCATTGAGAAAGACCTGGGTGGAGATGTCCCTGAAGCTAGATATGGGCATACAATTAATGTAGT
TCATAGCCGGGGAAAAAGCATGAGTGTTCTGTTTGGAGGGAGGATGTATACTCCTCTTGCACAAAGAACCACTGAAAAAT
GGAACAGCGTGGTCGACTGTTTGCCATCTGTGTTTCTCATTGATTTTGAGTTTGGATGCTGTACATCATACATACTTCCA
GAGCTTCAAGATGGACTTTCTTTCCACGTGTCAATTGCCAGAGATGATACAATCTACATCTTGGGAGGCCACTCACTTCA
AAATAACACCAGGTCCCCCAGCTTGTACAAGCTAAAAATTGATCTGCCCCTGGGCAGCCCAGCTGTGACCTGCACCATCC
TGCCAGGGGGGATATCAGTGTCAAGTGCTATAGTGACCCAGATTAGTGACACTGAATTTGTCCTTGTTGGTGGSTACCAC
TCAGACAACCAGAAACGGCTGGCATGTAACACCATAGTTCTGGAAGATAGTAAGATAGAGATAGCTGAAAGTGTGAGCCC
AGAGTGGACACCAGATATTAAACACTGCAGAATGTGGTTTGGCTGTGACATGGGTAAAGGGTCTGTTTTGCTGGGCATTC
CAGGGGCCAACAAACAAATAGTCCCAGATGCRAACTACTTCTACATTTTRARATGCAAAGGAGCAGAAGAGGACAAGGAA
GAAGAACTGATAACACAAATTTGCAGTCAGACATCAAGCGAAGACCCTGGAGACTCTGCTCCATTTGAAGATTCCGAGGA
GTTTTGTTTTAGTGCTGAAGCCAATAGCTTTGATGCAGACGATGCTGATACTTACAATGAAGATGATGAAGAGGATGAAT
CAGAAACAGGCTACTGGATCACCTGCTGTGCC
Turdus
??
??
??
???TACAATTAATGTAGT
TCATAGCCGGGGAAAAAGCGTGAGTGTTCTGTTTGGAGGGAGGTCATACACTCCTCTTGCACAAAGAACCACTGAAAAAT
GGAACAGTGTAGTTGACTGTTTGCCATCTGTGTTTCTCATTGATTTTGAGTTTGGATGCTGTACATCATACATCCTTCCA
GAGCTTCAAGATGGGCTTTCTTTCCACGTTTCAATTGCCAGAGATGATACAATCTACATCTTGGGAGGCCACTCACTTCA
AAATAACACCAGGTCCCCCAACCTGTACAAGCTAAAAATTGATCTGCCCCTGGGCAGCCCAGCTGTGACCTGCACCATCC
TGCCAGGGGGGATATCAGTGTCAAGTGCTATAGTGACCCAGATCAGTGACACTGAGTTTGTCCTGGTGGGTGGCTACCAC
TCAGACAGCCAGAAACGGCTGGCGTGTAACACCATAGTTCTGGAAGATAGTAAGATAGAGATTGTTGAAAGTGCGAGCCC
AGAGTGGACACCAGATATCAAACACTGCAGAACATGGTTCGGCTGTGATATGGGTAAAGGGTCTGTTTTGCTAGGCATTC
CAGGGGCCAACAAACAAATAAGCCCTGATGCAAACTACTTCTACATTTTGAGATGCAAAGGGGCAGAAGAGGACAAGGCA
GAAGAATTGATAACACAAATTTGCAGTCAGACATCAAGTGAAGACCCTGGAGACTCCACTCCATTTGAAGATTCGGAGGA
GTTTTGCTTTAGTGCTGAAGCCAACAGCTTTGATGCTGACGATGCTGATACTTACAATGAAGATGATGAAGAAGATGAAT
CAGAAACAGGCTACTGGATCACCTGCTGTGCC
Acrocephalus
AGATCCTGTCCCACTGGTGTTTTCCTCCTCGATATAAAGCAGAATGAGCTCAAAATGAAACCTGCCTTCTTCTCAAAAGA
TTCGTGTTACCTTCCCCCTCTCCGCTACCCTGCTCTTTGCACKCTCAGAAGCGAGGCAAGGGCTGATGAGTACCAGTATA
TCATCCATGGTGGTAAAACACCTAACAATGAACTTTCTGATAAGATTTACTTTATGAGTCTGGTAAGCAAAACTAGCAAG
AAAATGACGTTCCAATGCGTTGAGAAAGACCTGGGTGGAGATGTCCCTGAAGCTAGATATGGGCATACAATTAATGTAGG
TCATAGCCGGGGAAAAAGCATGAGTGTTCTGTTTGGAGGGAGGACATATACTCCTCTTGCACAAAGAACCACTGAAAAAT
GGAACAGCGTAGTCGACTGTTTGCCATCTGTGTTTCTCATTGATTTTGAGTTTGGATGCTGTACATCATACATACTTCCA
GAGCTTCAAGATGGACTTTCCTTCCATGTGTCAGTTGCCAGAGATGATACAATCTACATCTTGGGAGGCCACTCACTTCA
AAATAACACCAGGTCCCCCAACYTGTACAAGCTAAAAATTGATCTGCCCCTGGGCAGCCCRGCTGTGACTTGCACCATCC
TGCCAGGGGGGATATCAGTGTCAAGTGCTATAGTGACCCAGATCAGTGACACTGAATTTGTCCTTGTTGGTGGCTACCAC
TCAGACAATCAGAAACGGCTGGCATGTAATACCATAGTTCTGGAAGATAGTAAGATAGAGATTGTTGAAAGTGTGAGCCC
AGAGTGGACACCAGATATTAAACACTGCAGAATGTGGTTTGGCTGTGATATGGGTAAAGGGTCTGTTTTGCTGGGCATTC

CGGGGGCCAACAAACAAATAATCCCAGATGCGAACTACTTCTACATTTTGAGATGCAAAGGAGCAGAAGAGGATAARGAA
GAAGAATTGATAACACAAAATTGCAGTCAGACATCAGGTGAAGACCCTGGAGACTCTGCTCCATTTGAAGATTCAGAGGA
ATTTTGTTTTAGTGCTGAAGCCAATAGTTTTGATGCTGACGATGCTGATATTTACAATGAAGATGATGAAGAGGATGAAT
CAGAAACAGGCTACTGGATCACCTGCTGTGCC
Sitta
AGATCCTGCCCCACTGGTGTTTTCCTCCTCGATATAAAGCAGAATGAGCTCAAAATGAAACCTGCCTTCTTCTCTAAAGA
CTCGTGTTACCTCCCCCCTCTCCGCTACCCTGCTCTTTGCATGCTCCGAGGCGATGCAAGGGCTGATGAGTACCAGTATA
TCATCCATGGTGGTAAAACACCTAACAATGACCTTTCTGATAAGATTTACTTTATGAGTCTGGTAAGCAAAACCAGCAAG
AAAATGACGTTCCAATGCATTGAGAAAGACCTGGGCGGAGATGTCCCTGAAGCTAGATATGGGCATACAATTAATGTAAT
TCATAGCCGGGGAAAAAGCATGAGTGTTCTGTTTGGAGGGAGGTCATACACTCCTCTTGCACAAAGAACCACTGAGAAAT
GGAACAGCGTAGTCGACTGTTTGCCATCTGTGTTCCTCATTGATTTTGAGTTTGGGTGCTGTACATCATACATACTTCCA
GAGCTTCAAGATGGACTTTCTTTCCATGTTTCAATTGCCAGAGATGATACAATCTACATCTTGGGAGGCCACTCGCTTGA
AAATAACACCAGGTCCCCCAACTTGTTCAAGCTAAAAATCGACCTGCCCCTGGGCAGCCCGGCTGTGAGCTGCACCATCC
TGCCAGGGGGGATATCAGTGTCGAGTGCCATAGTGACCCAGATCAGCGACACTGAATTTGTCCTTGTCGGTGGCTACCAC
TCAGACAACCAGAAACGGCTGTCCTGCAACACCATAGTTCTGGAAGATAGTAAGATAGAGATTGTAGAAAGTGTGAGCCC
AGAGTGGACACCAGACATTAAACACTGCAGAATGTGGTTTGGCTGTGATATGGGCAAAGGGTCTGTTTTGCTGGGCATTC
CAGGGGCCACCAAACAAATAACCCCAGACGCAAACTACTTCTACATTTTGAGATGCAAAGGAGCAGAAGAGGACAAGGAA
GAAGAGCTGATAACACAAATTTGCAGTCAGACATCAAGTGAAGACCCTGGAGACTCCACCCCTTTTGAAGATTCAGAGGA
GTTCTGTTTTAGTGCTGAAGCCAATAGCTTTGATGCTGACGATGCTGATACTTACAATGAAGATGATGAAGAAGATGAAT
CAGAAACAGGCTACTGGATCACCTGCTCTGCC
Mimus
AGATCCTGCCCCACTGGTGTTTTCCTCCTCGATATAAAGCAGAATGAGCTCAAAATGAAACCTGCCTTCTTCTCTAAAGA
CTCGTGTTACCTTCCCCCTCTCCGCTACCCTGCTCTTTGCACGCTCAGAAGTGATGCAAGGGCTGATGAGTACCAGTATA
TCATCCATGGTGGTAAAACACCTAACAATGACCTTTCTGATAAAACTTACTTTATGAGTCTGGTAAACAAAGCCAGCAAG
AAAATGACATTCCAATGCATTGAGAAAGACCTGGGTGGAGATGTCCCTGAAGCTAGATATGGGCATACAGTCAATGTAGT
TCATAGCCGGGGGAAAAGCGTGACTGTTCTGTTTGGAGGGAGGTCATATACTCCTCTTGCTCAAAGAACCACTGAAAAAT
GGAACAGCGTAGTTGACTGTTTGCCATCTGTGTTTCTCATTGATTTTGAGTTTGGGTGCTGTACATCATACCTACTTCCA
GAGCTTCAAGATGGACTTTCTTTCCACGTTTCAATTGCCAGAGGTGATACAATCTACATCTTGGGAGGCCACTCACTTCA
AAATAACACCAGGTCTCCCAACTTGTACAAGATAAAAATTGATCTGCCCCTGGGCAGCCCGGCTGTGACCTGCACTGTCC
TGCCAGGGGGGATATCAGTGTCAAGTGCTATAGTGACCCAGATCAGTGACACTGAATTTGTCCTTGTTGGTGGCTACCAC
TCAGACAGCCAGAAACGGCTGGCGTGTAACACCATAGTTCTGGAAGATAGTAAGATAGAGATTGTTGAAAGTGTGAGCCC
AGAGTGGACACCAGATATTAAACACTGCAGAACGTGGTTTGGTTGTGATATGGGTAAGGGGTCTGTTCTGCTGGGCATTC
CAGGGGCCAACAAACAAATAATCCCAGATGCAAACTACTTCTACGTTTTGAGATGCAAAGAGGCAGAAGAAGACAAGGAA
GAAGAATTGATAA------
TTTGCAGTCAGACATCAAGTGAAGACCCTGGAGACTCCACTCCATTTGAAGATTCGGAGGAGTTTTGCTTTAGTGCTGAA
GCCAATAGCTTTGATGCTGACGATGCTGATACTTACAATGAAGATGATGAGGAAGACGAATCAGAAACAGGCTACTGGAT
CACCTGCTGTGCC
Sturnus_vulgaris
AGATCCTGCCCCACTGGTGTTTTCCTCCTCGATATAAAGCAGAATGAGCTCAAAATGAAACCTGCCTTCTTCTCTAAAGA
CTCGTGTTACCTTCCCCCTCTCCGCTACCCTGCTCTTTGCAGGCTCAGAAGCGATGCAAGGGCTGATGAGTACCAGTATA
TCATCCATGGTGGTAAAACACCTAACAATGACCTTTCTGATAAAATTTACTTTATGAGTCTGGTAAACAAAGCTAGCAAG
AAAATGACATTCCAGTGCATTGAGAAAGACCTGGGTGGAGATGTCCCTGAAGCTAGATATGGGCACACAATTAATGTAGT
TCATAGCCRGGGAAAAAGCGTGACTGTTCTSTTTGGAGGGAGGTCATATACTCCTCTTGCTCAGAGAACCACTGAAAAAT
GGAACAGTGTAGTCGACTGTTTGCCATCTGTGTTTCTCATTGATTTTGAGTTTGGATGCTGTACATCATACCTGCTTCCA
GAGCTTCAAGATGGACTTTCYTTCCACGTTTCAATTGCCARGGTGATACAATCTATATCTTGGGAGGCCACTCACTTCA
AAATAACACCAGGTCTCCCAACTTGTACAAGATAAAAATTGATCTGCCCCTGGGCAGCCCGGCTGTGACCTGCACCGTCC
TGCCAGGGGGGATATCAGTGTCAAGTGCTATAGTGACCCAGATCAGTGACATTGAATTTGTCCTTGTCGGTGGCTACCAC
TCAGACAGCCAGAAACGGCTGGCGTGTAACACCATAGTTCTGGAAGATAATAAGATAGAGATTGTTGAAAGTGTGAGCCC
GGAGTGGACACCAGATATTAAACACTGCAGAACGTGGTTTGGTTGTGATATGGGTAAGGGGTCTGTTCTGCTGGGCATTC
CAGGGGCCAACAAACAAATAATCCCAGATGCAAACTACTTCTACATTTTGAGATGCAAATGGCCAGAAGAGGACAAGGAA
GAAGAATTGATAACACAGACTTGCAGTCAGACATCAAGTGAAGACCCTGGAGACTCCACTCCATTTGAAGATTCGGAGGA
GTTTTGCTTTAGTGCTGAAGCCAATAGCTTTGATGCTGACGATGCTGACACTTACAATGAAGATGATGAAGAAGATGAAT
CAGAAACAGGCTACTGGATCACCTGCTGTGCT
Creadion_caruncul atus
AGATCCTGCCCCACTGGTGTTTTCCTCCTCGATATAAAGCAGAATGAGCTCAAAATGAAACCTGCCTTCTTCTCTAAAGA
CTCGTGTTACCTTCCCCCTCTCCGCTACCCTGCTCTTTGCACGCTCAGAAGCGATGCAAGGGCTGATGAGTACCAGTATG
TCATCCATGGTGGTAAAACACCAAACAATGACCTTTCTGATAAGATTTACTTTATGAGTCTGGTAAGCAAAACTAGCAAG
AAAATGACGTTCCAATGCATTGAGAAGGACCTGGGTGGAGATGTCCCTGAAGCTAGATACGGGCATACAATTAACGTAAT
TCATAGCCGGGGAAAAAGCRTAAGTGTTCTGTTTGGAGGGAGGTCGTATACTCCTCTTGCACAAAGAACCACTGAAAAAT
GGAACAGCGTAGTTGACTGTTTGCCATCTGTGTTTCTCATTGATTTTGAGTTCGGATGCTGTACATCATACATACTACCA

GAGCTTCAAGATGGACTTTCTTTCCACGTTTCAATTGCCAGAGGTGATACAATCTACATCTTGGGAGGCCACTCACTTCA
AAATAACACCAGGTCCCCCAACTTGTACAAGCTAAAAATTGATCTGCCCCTGGGCAGCCCAGCTGTTACCTGCACCATCC
TGCCAGGGGGGATATCGGTGTCAAGTGCTATAGTGACCCAGATCAGTGATACTGAATTTGTCCTTGTCGGTGGCTACCAC
TCTGACAACCAGAAACGGTTGGTGTGTAACACCATAGTTCTGGAAGACAGTAAGGTAGAGATTGTTGAAAGTCTGAGCCC
AGAGTGGACACCAGATATTAAACACTGCAGAATGTGGTTTGGCTGTGATATGGGTAAAGGGTCTGTTTTGCTGGGCATTC
CAGGGGCCAACAAACAATTAATCCCAGATGCAAACTACTTCTACATTTTGAGATGCAAAGGAGCAGAAGAGGACAAGGAG
GAAGAATTGATAACACAAATTTGCAGTCAGACATCAAGTGAAGACCCTGGAGACTCCACTCCATTTGAAGATTCGGAAGA
GTTCTGTTTTAGTGCTGAAGCCAATAGCTTTGATGCTGACGATGCTGACATTTACAATGAAGATGATGAAGAAGATGAAT
CCGAAACGGGCTACTGGATCACCTGCTGTGCC
Parus
AGATCCTGCCCCACTGGTGTTTTCCTCCTCAATATAAAGCAGAATGAGCTCAAAATGAAACCTGCCTTCTTCTCTAAAGA
CTCGTGTTACCTTCCCCCTCTCCGCTACCCTGCACTTTGCACACTCAGAAGCGATGCAAGGGCTGATGAGTACCAGTATA
TCATCCACGGTGGTAAAACACCTAACAATGACCTTTCTGATAAGATTTACTTTATGAGTCTGGTAAGCAAAGCTAGCAAG
AAAATGACGTTCCAATGCATTGAGAAAGAGCTGGGTGGAGATGTCCCTGAAGCTAGATACGGGCATACAATTAATGTAGT
TCATAGCCGGGGAAAAAGCATGAGTGTTCTGTTTGGAGGGAGGTCGTATACTCCTCTTGAACAAAGAACCACTGAAAAAT
GGAACAGCGTAGTCGACTGTTTGCCGTCTGTGTTTCTTATTGATTTTGAGTTTGGATGCTGTACATCATACATACTTCCA
GAGCTTCAAGATGGACTTTCTTTCCATGTTTCAATTGCCAGAGATGACACTATCTACATCCTGGGAGGCCACTCACTTCA
AAATAACACCAGGTCCCCTAACTTGTACAAGCTAAAAATTGATCTGCCGCTGGGCAGCCCGGCTGTGACCTGCACCATCC
TGCCAGGGGGGATATCAGTGTCAAGTGCTATAGTGACCCAGATCAGTGACAATGAATTTGTCCTTGTCGGTGGCTACCAC
TCAGACAACCAGAAACGACTGGCATGTAAYACCATAGTTCTGGAAGACAGTAAGATAGAGATTGTTGAAAGTGTGAGCCC
AGAGTGGACACCAGATATTAAACACTGCAGAATGTGGTTTGGCTGTGATATGGGTAAGGGGTCTGTTTTGCTGGGCATTC
CAGGGGCCAACAAACAGATAATGCCAGATGCAAACTACTTCTACATTTTGAGATGCAAAGGCGCAGAAGAGGACAAGGAA
GAAGAATTGATAACACAAATTTGCAGTCAGACATCAAGTGAAGACCCTGGAGATTCTGCTCCATTTGAAGATTCAGAGGA
GTTTTGTTTTAGTGCTGAAGCCAACAGCTTTGATGCTGACGATGCTGATACTTACAATGAAGACGATGAAGAAGATGAAT
CAGAAACAGGCTACTGGATCACCTGCTGTGCC
Petroica_rosea
??
??
??
??
??
??
??
??
??
??
??
??
??
??
????????????????????????????????
Eopsaltria_australis
??
??
??
??
??
??
??
??
??
??
??
??
??
??
????????????????????????????????
Serinus
??
??

??
??
??
??
??
??
??
??
??
??
??
??
????????????????????????????????
Icterus
AGATCCTGCCCCACTGGTGTTTTCCTTCTCGATATAAAGCAGAATGAGCTCAAAATGAAACCTGTCTTCTTCTCCAAAGA
CTCATGCTACCTTCCCCCTCTCCGCTACCCTGCTCTTTGCACGCTCAGAAGCAATTCAAGGGCTGATGAGTACCAGTACA
TCATCCATGGTGGTAAAACACCTAACAATGACCTTTCTGATAAGATTTACTTTATAAGTTTGGCAAGCAAAACTAGCAAG
AAAATGACATTCCAATGCATTGAGAAGGACCTGGGTGGAGATGTGCCTGAAGCTAGATATGGGCATACAATTAACGTAGT
TCACAGCCGGGGAAAAAGCATGAGTGTTCTGTTTGGAGGGAGGTCATATACTCCTCTTGCACAGAGAACCACTGAAACAT
GGAACAGTGTAGTCGACTGTTTGCCCTCTGTGTTTCTCATTGATTTTGAGTTTGGATGCTGTACATCATACATGCTTCCA
GAGCTTCAAGATGGACTTTCTTTCCATGTTTCAATTGCCAAAGATGATACAATCTACATCTTGGGGGGCCACTCACTTCA
AAATAACACCAGGTCCCCCAACTTCTACAAGCTAAAAATTGATCTGCCCCTGGGCAGCCCGGCTGTGACCTGCACCATCC
TGCCAGGGGGGATATCAGTGTCAAGTGCYATAGTGACCCAGATCAGTGACACTGAATTTGTCCTTGTTGGCGGCTACCTC
TCAGACAACCAGAAACGGCTGGTATGTAACACCATAGTTTTGGAGGATAATAAGATAGAGATTGTTGAAAGTGTGGGCCC
AGAGTGGACAGCAGATATTAAACACTGCAGAATGTGGTTTGGCTGTGATATGGGTAAAGGGTCTGTTTTGCTGGGCATTC
CAGGGGCCAACAAACAAATAATCCCAGATGCAAACTACTTCTACATTTTGAGATGCAAAGGAGCAGAAGAGGACAAGGAA
GAAGAAATGATAACACAAACTTGCAGTCAGACATCAAGTGAAGACCCTGGAGACTCCACTCCATTTGAAGATTCGGAGGA
GTTTTGTTTTAGTGCTGAAGCCAATAGCTTTGATGCTGACGATGCTGATACTTACAATGAAGAAGATGAAGAAGATGAGT
CAGAAACAGGCTACTGGATCACCTGCTGTGCC
Motacilla
AGATCCTGCCCCACTGGTGTTTTCCTTCTCGATATAAAGCAGAATGAGCTCAAAATGAAACCTGTCTTCTTCTCTAAAGA
CTCATGTTACCTTCCCCCTCTCCGCTACCCCGCTCTTTGCACCCTCAGAAGCGATGCAAGGGCTGATGAGTACCAGTATA
TCATCCATGGTGGTAAAACACCTAACAATGACCTTTCTGATAAGATTTACTTTATAAGTTTGGTAAGCAAAACGAGCAAG
AAAATGACGTTCCAATGCATTGAGAAAGACCTGGGTGGAGAYGTGCCTGAGGCTAGATATGGGCATTCAATTAACGTAGT
TCATAGCCGGGGGAAAAGCATGAGTGTTCTGTTTGGAGGGAGGTCGTATACTCCTCTTGCACAGAGAACCACTGAAACAT
GGAACAGTGTAGTCGACTGTTTGCCATCTGTGTTTCTCATTGATTTTGAGTTTGGATGCTGTACATCATATATGCTTCCA
GAGCTTCAAGACGGACTTTCTTTCCACGTCTCAATTGCCAGAGGTGATACAATCTACATCTTGGGAGGCCACTCACTTCA
AAATAACACCAGGTCCCCYAACTTGTACAAGCTAAAAATTGATCTGCCCCTGGGCAGCCCGGCTGTGACCTGCACCATCC
TGCCAGGGGGGATATCAGTGTCAAGTGCCATAGTGACCCAGGTCAGTGACAATGAATTTGTCCTTGTCGGTGGCTATCAC
TCAGACAACCAAAAACGGCTGGCGTGTAACACCATAGTTCTGGAAGATGGTAAGATAGAGATTGTTGAAAGTGTGGGCCC
AGAGTGGACACCAGATATTAAACACTGCAGGATGTGGTTTGGCTGTGATATGGGTAAAGGGTCTGTGTTGCTGGGCATTC
CAGGAGCCAACAAACAAATAATCCCAGATGCAAACTACTTCTACATTTTGAGATGCAAGGGAGCAGAAGAGGACAAGGAA
GAAGAATTGATAACACAAACTTGCAGTCAGACATCAAGTGAAGACCCTGGAGACTCCACTCCATTTGAAGATTCGGAGGA
GTTTTGTTTTAGTGCTGAAGCCAATAGCTTTGATGCTGACGATGCTGATACTTACAATGAAGATGATGAAGAAGATGAAT
CAGAAACAGGCTACTGGATCACCTGCTGTGCC
Emberiza
AGATCCTGCCCCACTGGTGTTTTCCTTCTCGATATAAAGCAGAATGAGCTCAAAATGAAACCTGTCTCCTTCTCCAGAGA
CTCATGTTACCTTCCCCCTCTCCGCTACCCTGCTCTTTGCACACTCAGAAGTGATGCAAGGGCTGATGAGTACCAGTATA
TCATCCATGGTGGTAAAACACCTAACAATGACCTTTCTGATAAGATTTACTTTATAAATTTGGTAAGCAAAACTAGCAAG
AAAATGACATTCCAATGCATTGAGAAAGACCTAGGTGGAGATGTGCCTGAAGCTAGATATGGGCATACAATTAACGTAGT
TCACAGCCGGGGAAAAAGCATGAGTGTTCTGTTTGGAGGGAGGTCGTATACTCCTCTTGCACAGAGAACCACGGAAACAT
GGAACAGTGTAGTTGACTGTTTGCCATTTGTGTTTCTCATTGATTTTGAGTTTGGATGCTGTACATCATACATGCTTCCA
GAGCTTCAAGATGGACTTTCTTTCCATGTTTCAGTTGCCAGAGGTGATACAATCTACATCTTGGGAGGCCACTCACTTCA
AAATAACACCAGGCCCCCCAACTTGTACAAGCTAAAAATTGATCTGCCCCTGGGCAGCCCGGCTGTGACCTGCACCATCC
TGCCAGGGGGGATATCAGTGTCAAGTGCTATAGTGACCCAGGTCAGTGACACTGAATTTGTCCTTGTCGGTGGCTACCTC
TCAGACAACCAGAAACGGCTGGCATGTAACACCATAGTTCTGGAGGATAATAAGATAGAGATTGTTGAAAGCGTGGGCCC
AGAGTGGACACCAGATATTAAACACTGCAGAATGTGGTTTGGCTGTGATATGGGTAAAGGGTCTGTTTTGCTGGGCATTC
CGGGGGCCAACAAACAAATAATCCCAGATGCAAACTACTTCTACATTTTGAGATGCAAAGGAGCAGAAGAGGATAAGGAA
GAAGAACTGATAACACAAACTTGCAGTCAGACATCAAGTGAAGACCCTGGAGACTCCACTCCATTTGAAGATTCAGAGGA

GTTTTGTTTTAGTGCTGAAGCCAATAGCTTTGATGCTGACAATGCTGATACTTACAATGAAGATGATGAAGAAGATGAGT
CAGAAACAGGCTACTGGATCACCTGCTGTGCC
Pomatostomus
AGATCCTGCCCCACTGGTGTTTTCCTCCTCGATATAAAGCAGAATGAGCTCAAAATGAAGCCTGCCTTTTTCTCTAAAGA
CTCGTGTTACCTTCCCCCTCTCCGCTACCCTGCTCTTTGCACGCTTAGAAGCAATGCAAGGTCTGATGAGTACCAGTATA
TCATCCATGGTGGTAAAACACCTAACAATGACCTTTCTGATAAGATTTACTTTATGAGTCTGGTAAGCAAAACTAGCAAG
AAAATGACATTCCAATGCATGGAGAAAGACCTGGGTGGAGACGTGCCTGAAGCTAGATATGGACATACAATAAACGTAGT
TCATAGCCAGGGAAAAAGTATGAGTGTTCTATTTGGAGGGAGGTCGTATACTCCTCTTGCACTGAGAACCACTGAAAAAT
GGAACAGCGTAGTTGACTGTTTGCCATCTGTGTTTCTCATTGATTTTGAGTTTGGATGCTGTACATCATACATACTTCCA
GAGCTTCAAGATGGACTTTCTTTTCACGTTTCAATTGCCAGAGGTGACACAATCTACATCTTGGGAGGCCATTCACTTCA
AAATAACACCAGGTCCCCCAACTTGTACAAGCTAAAAATTGATATACCCCTGGGCAGCCCAGCTGTGAGCTGCACCATCC
TGCCAGGGGGGATATCAGTGTCAAGTGCTATAGTGACCCAGATCAGTGATACCGAATTTGTCCTGGTCGGTGGCTACCAC
GCTGACAACCAAAAACGGTTGGTGTGTAACACCATAGTTCTGGAAGACAGTAAGATAGAGATTGTTGAAAGCGTGAGCCC
AGAGTGGACACCAGATATTAAACACTGCAGAATGTGGTTTGGCTGTGATATGGGTAAAGGGTCTATTTTGCTGGGCATTC
CAGGGGCCAACAAACAATTAATCCCAGATGCAAACTACTTCTACATTTTGAGATGCAAAGGAGCAGAAGCGGACAAGGAA
GAAAAATTGATGACACAAATTTGCAGTCAGACATCAAGTGAAGACCCTGGAGACTCTACTCCGTTTGAAGATTCGGAGGA
GTTTTGTTTTAGTGCTGAAGCCCATAGCTTTGACGCTGATGATACTGATACTTACAATGAAGATGATGAAGAAGATGAAT
CAGAAACAGGCTACTGGATCACCTGCTGTGCC
Rhipidura
AGATCCTGCCCCACTGGTGTTTTCCTCCTCGATATAAAGCAGAATGAGCTGAAAATGAAACCTGCCTTCTTCTCTAAAGA
CTCGTGTTACCTTCCCCCTCTCCGCTACCCCGCTCTTTGCACGCTCAGAAGCGATGCAAAGGCTGATGAGTACCAGTATA
TCATCCATGGTGGTAAAACACCCAACAATGACCTTTCTGATAAGATTTACTTTATGAGTCTGGTAAGCAAAACTAGCAAG
AAAATGACGTTCCAATGCATTGAGAAAGACCTGGGTGGAGATGTCCCTGAAGCTAGATATGGGCATACAATTAGTGTAGT
TCATAGCCGGGGAAAAAGCATGAGTGTTCTGTTTGGAGGGAGGTCGTATACTCCTCTTGCACAAAGAACCACTGAAAAAT
GGAACAGTGTAGTTGACTGTTTGCCATCTGTGTTTCTCATTGATTTTGAGTTTGGATGCTGTACATCATACATCCTTCCA
GAGCTTCAAGATGGACTTTCTTTCCACGTTTCAATTGCCAGAGATGATACAATCTACATCTTGGGTGGCCACTCACTTCA
AAATAATACCAGGTCCCCCAACCTGTACAAGCTAAAAATTGATCTGCCCCTGGGCAGCCCAGCTGTGACCTGCACCATCC
TGCCAGGAGGGGTATCAGTGTCAAGTGCTATAGTGACCCAGATCAGTGATACTGAATTTGTCCTTGTCGGTGGCTACCAC
TCTGACAACCAGAAACGGYTGGTGTGTAACACCATAGTTCTGGAAGAGAATAAGATAGAAATTGTTGAAAGTGTGAGCCC
AGAGTGGACACCAGATATTAAACACTGCAGAACGTGGTTTGGCTGTGATATGGGTAAGGGATCTGTTTTGCTGGGCATTC
CAGGGGCCAACAAACAACTGAGCCCAGATGCAAACTACTTTTACATTTTGAGATGCAAAGGAGCAGAAGAGGACAAGGAA
GAAGAATCGATAACACAAATTTGCAGTCAGACGTCAAGTGAAGACCCTGGAGACTCCACRCCATTTGAAGACTCGGAGGA
GTTTTGTTTTAGTGCTGAAGCCAATAGCTTTGACATTGACGATGCTGACACTTACAATGAAGATGATGAAGAAGATGAAT
CAGAAACAGGCTACTGGATCACCTGCTGTGCC
Pica_pica
??
??
??
??
??
??
???CTC?CTTCA
GAATAACACCAGGTCCCCCAACTTGTACAAGGTAAAAATTGATCTGCCCCTGGGCAGCCCAGCTGTGACCTGCACCATCC
TGCCAGGGGGGGTATCTGTGTCAAGTGCTATAGTGACACAGATAAGTGATACTGAATTTGTCCTTGTCGGTGGCTACCAC
TCTGACAACCAGAAACGGATGGTGTGTAACACCATAGTTCTGGAAGATAGTAAGATAGAGATTGTTGAAAGTGTGAGCCC
AGAGTGGACACCAGATATTAAACACTGCAGAACGTGGTTTGGCTGTGATATGGGTAAAGGGTCTGTTTTGCTGGGCGTTC
CAGGGGCCAACAAACAATTAAGCCCAGATGCAAACTACTTTTATATTTTGAGATGCAAAGGAGCAGAAGAAGACAAGGAA
GAAGAATCGATAACACAAATTTGCAGTCAGACATCAAGTGAAGACCCTGGAGACTCCACTCCATTTGAAGACTCGGAGGA
GTTTTGTTTTAGCGCTGAAGCCAATAGCTTTGACATTGACGATGCTGGCACTTACAATGAAGATGATGAAGAAGATGAAT
CAGAAACGGGCTACTGGATCACYTGCTGTGCC
Manucodia
AGATCCTGCCCCACTGGTGTTTTCCTCCTCGATATAAAGCAGAATGAGCTCAAAATGAAACCTGCYTTCTTCTCTAAAGA
CTCGTGTTACCTTCCCCCTCTCCGCTACCCTGCTCTTTGCACGCTCAGAAGCGATGCAAAGGCTGATGAGTACCAGTACA
TCATCCATGGTGGTAAAACACCTAACAATGACCTTTCTGATAAGATTTATTTTATGAGTCTGGTAAGCAAAACTAGCAAG
AAAATGACATTCCAATGCATTGAGAAAGACCTGGGTGGAGATGTCCCTGAAGCTAGATATGGGCATACAATTAATGTAGT
TCATAGCCGGGGAAAAAGCATGAGTGTTCTGTTTGGAGGGAGGTCGTATACTCCTCTTGCACAAAGAACCACTGAAAAAT
GGAACAGTGTAGTTGACTGCTTGCCATCTGTGTTTCTCATTGATTTTGAGTTTGGGTGTTGTACATCATACATCCTTCCA
GAGCTTCAAGATGGACTTTCTTTCCACGTTTCAATTGCCAGAGATGATACAATCTACATCTTGGGCGGCCACTCACTTCA
AAATAACACCAGGTCCCCCAACYTGTACAAGGTAAAAATTGATCTGCCCCTGGGCAGCCCAGCTGTGACGTGCACCGTCC
TGCCAGGGGGGGTATCAGTGTCAAGTGCTATAGTGACCCAGATCAGTGATACTGAATTTGTCCTTGTCGGTGGCTACCAC

TCTGACAACCAGAAACGGTTGGTGTGTAACACCATAGTTCTGGAAGATAGTAAGATAGAGATTGTTGAAAGTGTGAGCCC
AGAGTGGACACCAGATATTAAACACTGCAGAACGTGGTTTGGCTGTGATATGGGTAAAGGGTCTGTTTTGCTGGGCATTC
CAGGGGCCAACAAACAATTAAGCCCAGATGCAAACTACTTTTACATTTTGAGATGCAAAGGAGCAGAAGAGGACAAGGAA
GAAGAATCGCTAACACAAATTTGCAGTCAGACGTCAAGTGAAGACCCTGGAGACTCCACTCCATTTGAAGACTCAGAGGA
GTTTTGTTTTAGTGCTGAAGCCAATAGCTTTGACATTGACGATGCTGACACTTACAATGAAGATGATGAAGAAGATGAAT
CGGAAACGGGCTACTGGATCACCTGCTGTGCC
Corvus_corone
AGATCCTGCCCCACTGGTGTTTTCCTCCTCGATATAAAGCAGAATGAGCTGAAAATGAAACCCGCCTTCTTCTCTAAAGA
CTCGTGTTACCTTCCCCCTCTCCGCTATCCCGCTCTTTGCATGCTCAGAAGCGATGCAAAGGCTGATGAGTACCAGTATA
TCATCCATGGTGGTAAAACACCTAACAATGACCTCTCTGATAAGATTTACTTTATGAGTCTCGTAAGCAAAACTAGCAAG
AAAATGACATTCCAATGCATTGAGAAAGACCTGGGTGGAGATGTCCCTGAAGCTAGATATGGGCATACAATTAATGTAGT
TCATAGCCGGGGTAAAAGCATGAGTGTTCTGTTTGGAGGGAGGTCATATACTCCTCTTGCACAAAGAACCACTGAAAAAT
GGAACAGTGTAGTTGACTGCTTGCCATCTGTGTTTCTCATTGATTTTGAGTTTGGATGTTGCACATCATACATCCTTCCA
GAGCTTCAGGATGGACTTTCTTTCCATGTTTCAATTGCCAGAGATGATACAATCTACATCTTGGGTGGCCACTCACTTCA
AAATAACACCAGGTCCCCCAACTTGTACAAGGTAAAAATTGATCTGCCCCTGGGCAGCCCAGCTGTGACCTGCAGCATCC
TGCCAGGGGGGGTATCTGTGTCAAGTGCTATAGTGACGCAGATAAGTGATACTGAATTTGTCCTTGTCGGTGGCTACCAT
TCTGACAACCAGAAACGGTTGGTGTGTAACACCATAGTTCTGGAAGATAGTAAGGTAGAGATTGTTGAAAGTGTGAGCCC
AGAGTGGACACCAGATATTAAACACTGCAGAACGTGGTTTGGCTGTGATATGGGTAAAGGATCTATTTTGCTGGGCGTTC
CAGGGGCCAACAAACAATTAAGCCCAGATGCAAACTACTTTTACATTTTGAGATGCAAAGGAGCAGAAGAGGACAAGGAA
GAAGAATCAATAACACAGATTTGCAGTCAGACATCAAGTGAAGACCCTGGAGACTCCACTCCATTTGAAGACTCGGAGGA
GTTTTGTTTTAGCGCTGAAGCCAATAGCTTTGACGTTGACGATGCTGACACTTACAATGAAGATGATGAAGAAGATGAAT
CAGAAACGGGCTACTGGATCACCTGCTGTGCC
Vireo
AGATCCTGTCCCACTGGTGTTTTCCTCCTCGATGTGAAGCAGAATGAGCTCAAAATGAAACCTGCCTTCTTCTCTAAAGA
CTCGTGTTACCTTCCCCCTCTCCGCTACCCCGCCCTTTGCACACTCAGAAGCGATG---------
ATGTGTACCAGTATATCATCCATGGTGGTAAAACACCTAACAATGACCTTTCCGATAAGATTTACTTTATGAGTCTGGTA
AGCAAAACTAACAAGAAAATGACGTTCCAATGCATTGAGAAAGACCTGGGTGGAGATGTCCCTGAAGCTAGATATGGGCA
TACAATTAATGTAGTTCATAGCCGGGGAAAAAGCATGAGTGTTCTCTTTGGAGGGAGGTCGTATACTCCTCTTGCACAAA
GAACCACTGAAAAATGGAACAGCGTAGTTGACTGTTTGCCATCTGTGTTTCTCATTGATTTTGAGTTTGGATGCTGTACA
TCATACATCCTTCCAGAGCTTCAAGATGGACTTTCTTTCCACGTTTCAATTGCCAGAGATGATACAATCTACATCTTGGG
AGGCTACTCACTTCAAAATAACACCAGGTCSCCCAACTTATACAAGCTAAAAATTGATCTGCCCCTGGGCAGCCCAGCTG
TGACCTGCACCATCCTGCCAGGGGGGATATCAGTGTCAAGTGCTATAGTGACCCAGATCAGTGATGCTGAATTTGTCCTT
GTCGGTGGCTACCACTCTGAGAACCAGAAACGGYTGGTGTGTAACACCATAGTTCTGGATGATAGTAAGATAGAGATTGT
TGAAAGTGTGAGCCCAGAGTGGACACCAGATATTAAACACTGCAGAACATGGTTTGGCTGTGATATGGGTAAAGGGTCTG
TATTACTGGGCATTCCAGGGGCCAACAAACAATTAATCCCAGATGCAAACTACTTCTACATTTTGAGATGCAAAGGAGGA
GAAGAGGACAAGGAAGAAGAATCGCTAATGCAAATTTGCAGTCAGACATCAAGTGAAGACCCTGGAGACTCCACTCCATT
TGAAGACTCAGAGGAGTTTTGTTTTAGTGCTGAAGCMAATCACTTTGATGTTGACGATGCTGACATTTACAATGAAGATG
ATGAAGAAGATGAATCAGAAACAGGCTACTGGATCACCTGCTGTGCC
Camptostoma_obsoletum
AGATCCTGCCCCACTGGTGTTTTCCTCCTCGATATAAAGCAGAATGAGATCAAAATGAAACCTGCCTTCTTCTCCAAAGA
CTCATGTTACCTTCCCCCTCTCCGCTACCCTGCTCTTTGCACACTCAGAAGCAATGCAAAATCTGATGAGTACCAGTATA
TCATCCATGGTGGTAAAACACCTAACAATGACCTCTCTGATAAGATTTACCTTATACGTCTGGTAAGCAAAAGTAGCAAG
AAAATAACGTTCCAATGCATTGAGAAAGACCTGGGTGGAGATGTCCCTGAAGCTAGATATGGGCATACAATTAATGTGGT
TCATAGCCGGGGAAAAAGCATGAGTGTTATATTTGGAGGGAGATCATATACTCCTCTTGAACAAAGAACTACTGAAAAAT
GGAACAGCGTAGTCGACTGTTTGCCATCTGTGTTTCTTGTTGATTTTGAGTTTGGATGCTGTACATCATACATGCTTCCA
GAGCTTCAAGATGGACTTTCTTTCCATGTTTCAATTGCCAGAGATGATACGATCTACATTTTGGGAGGCCATTCACTTCA
AAATAACACCAGGTGCCCCAACTTGTACAAGCTAAAAGTTGATCTCCCACTGGGCAGCCCAGCTGTGACCTGCACCATCT
TGCCAGGGGGGATATCAGTGTCAAGTGCTATAGTGACTCAGACCAGTGATACTGAATTTGTCCTTGTCGGTGGCTACCAC
TCTGACAGCCAGAAACGGTTGGTGTGTAACACCATAGTTCTGGAGGATAGCAAGATAGAGATTGTTGAAAGGGAAAGCCC
AGAGTGGACACCAGATATTAAACACTGCAGAATATGGTTTGGCTGTGATATGGGCAAAGGGTCTGTATTGCTGGGCATTC
CAGGGGCCAACAAACAGTTAATCTCAGATGCAAACTACTTCTACATTTTGAGATGCAAAGGAGAAGAAGAGGACAAGGAA
GAAGAATTGACAACACAAATTTGCAGTCAGACGTCAAGCGAAGACCCTGGAGATTCCACTCCAYTTGAAGATTCRGAGGA
GTTTTGCTTTAGTGCTGAAGCCAGTAGCTTTGACATTGATGATACTGACACTTATAATGAGGATGATGAAGAAGATGAAT
CAGAAACGGGCTACTGGATCACCTGCTGTGCC
Promerops_cafer
AGATCCTGCCCCACTGGTGTTTTCCTCCTCGATATAAAGCAGAAAGAGCTCAAAATGAAACCTGCCTTCTTCTCTAAAGA
CTCATGTTACCTTCCCCCTCTCCGCTACCCTGCTCTTTGCACACTCAGAAGCGATGCAAAGGCTGATGAGTACCAGTATA
TCATCCATGGTGGTAAAACACCCAACAATGACCTTTCTGATAAGCTTTACTTTATGGGTCTGGTAAGCAAAACTAGCAAG
AAAATGACGTTCCAATGCGTTGAGAAAGACCTGGGTGGAGATGTCCCTGAAGCTAGATATGGGCATACAATTAATGTAAT
TCATAGCCGGGGAAAAAGCGTGAGTGTTCTGTTTGGAGGGAGGTCGTATACTCCTCTTGCACAAAGAACCACTGAAAAAT

GGAACAGCGTAGTCGACTGTTTGCCATCTGTGTTTCTTATTGATTTTGAGTTTGGATGCTGTACATCATACATACTTCCA
GAGCTTCAAGATGGACTTTCTTTCCATGTTTCAATTGCCAGAGATGATACAATCTACATCTTGGGAGGTCACTCACTTCA
AAATAACATCAGGTCCCCTAACTTGTACAAGCTAAAAGTTGATCTGCCCCTGGGCAGCCCGGCGGTGACCTGCACCATCC
TGCCAGGGGGGATATCAGTGTCAAGTGCTATAGTGAGCCAGATCAGTGACACTGAATTTGTCCTTGTCGGTGGCTACCAC
TCAGACAACCAGAAACGGCTGGTGTGTAACACCATAGTTTTGGAAGATAGTAAGATAGAGATTGTTGAAAGTGTGGGCCC
AGAGTGGACACCAGATATTAAACACTGCAGAACGTGGTTTGGCTGTGATATGGGTAAAGGGTCTGTTTTGCTGGGCATTC
CAGGGGCCAATAAACAAATAATCCCAGATGCAAACTACTTCTACATTTTGAGATGCAAAGGAGCAGAAGAGGACAAGGAA
GAAGAACTGATAACACAAATTTGCAGTCAGACATCAAGTGAAGACCCYGGAGACTCYACTCCATTTGAAGATTCAGAGGA
GTTTTGTTTTAGTGCTGAAGCCAATAGCTTTGATGCTGACGATGCTGATACTTACAATGAAGATGATGAAGAAGATGAAT
CAGAAACAGGCTACTGGATCACCTGTTGTGCT
Oriolus
AGATCCTGCCCCACTGGTGTTTTCCTCCTTGATATAAAGCAGAATGAGCTCAAAATGAAACCTGCCTTCTTCTCTAAAGA
CTCGTGTTACCTTCCCCCTCTCCGCTACCCCGCGCTTTGCACACTCAGAAGTGATGCAAAGGCTGATGAATACCAGTATA
TCATCCATGGTGGTAAAACACCTAACAATGACCTTTCTGATAAGATTTACTTTATGAGTTTGGTAAACAAAACTAGCAAG
AAAATGACATTCCAATGCATTGAGAAAGATCTGGGTGGAGATGTCCCTGAAGCTAGATATGGGCATACAATTAATGTAGT
TCATAGCCRGGGAAAAAGCATGAGTGTTCTGTTTGGAGGGAGGTCATACACTCCTCTTGCACAAAGAACCACTGAAAAAT
GGAACAGCGTAGTTGACTGTTTGCCATCTGTGTTTCTCATTGATTTTGAGTTTGGATGCTGTACGTCATACATCCTTCCA
GAGCTTCAAGATGGACTTTCTTTCCACGTTTCAATTGCCAGAGATGATACAATCTACATCTTGGGAGGCCACTCACTTCA
AAATAACACCAGGTCCCCCAACCTGTACAAGCTAAAAATTGATCTGCCCCTGGGCAGCCCAGCTGTGACCTGCACCATCC
TGCCAGGGGGCATATCGGTGTCAAGTGCTATAGTGACCCAGGTCAGTGATACTGAATTTGTTCTTGTCGGTGGCTACCAC
TCTGACAACCAGAAAAGGTTGGTGTGCAACAGCATAGTTCTGGAAGACAGTAAGATAGAGATTGTTGAAAGTGTGAGCCC
AGAGTGGACACCAGATATTAAACACTGCAGAACGTGGTTTGGCTGTGACATGGGTAAAGGGTCTGTATTACTGGGMATTC
CAGGGGCCARCAAACAATTAATCCCAGATGCAAACTACTTCTATATTTTGAGATGCAAAGGAGCAGAAGAGGACAAGGAA
GAAGAATCGATAACACAAATTTGCAGTCAGACATCAAGTGAAGACCCTGGAGACTCCACTGCATTTGAAGACTCAGAGGA
GTTTTGTTTTAGTGCTGAAGCCAATAGCTTTGATGTTGACGATGCCGACACTTACAATGAAGATGATGAAGAAGATGAAT
CAGAAACGGGCTACTGGATCACCTGCTGTGCC
Dendroica
??
??
??
??
??
??
??
??
??
??
??
??
??
??
????????????????????????????????
Nectarinia
AGATCTTGTCCAACTGGTGTTTTCCTCCTTGATATAAAGCAGCATGAGCTCAAAATGAAACCTGTSTTCTTCTCTAAAGA
CTCATGTTACCTTCCCCCTCTMCGGTACCCTGCTCTTTGCACACTCAGAAGCAATGCAAGGGCTGATGAGCACCAGTATA
TCATCCATGGTGGTAAAACACCTAACAATGACCTTTCTGATAAGATTTACTTCATGAGTTTGGTAAGCAAAACTAGCAAG
AAAATTACATTCCAATGCATTGAGAAGGACCTGGGTGGAGATGTCCCTGAAGCTAGGTATGGGCATACAATTAATGTAGT
TCATAGCCGGGGAAAAAGYGTGAGTGTTCTGTTTGGAGGGAGGTCGTACACTCCTCTTGCACAAAGAACCACTGAAACAT
GGAACAGCGTAGTCGACTGTTTGCCATCTGTGTTTYTCATTGACTTTGAGTTTGGATGCTGTACATCATACATACTTCCT
GAGCTTCAAGATGGACTTTCTTTCCACGTTTCGATTGCCAGAGATGATACAATCTACATCTTGGGAGGCCACTCACTTCA
AAATAACACCAGGTCCCCCAACTTGTACAAGCTAAAAATTGATCTGCCCCTGGGCAGCCCGGCTGTGACCTGCACCATCC
TGCCAGGGGGGATTCCAGTGTCAAGTGCTATAGTGACCCAGATCAGTGACACTGAATTTGTCCTTGTTGGTGGCTACCAC
TCAGACAACCAGAAACGGCTGGCATGTAACACCATAGTTCTGGAAGACAGTAAGATAGAGATTGTTGAAAGYGTGGGCCC
AGAGTGGACACCAGATATTAAACACTGCAGAACATGGTTTGGCTGTGATATGGGTGAAGGGTCTGTTTTGCTGGGCATTC
CAGGGGCCAACAAACAAATAATCCCAGATGCAAACTACTTCTACATTTTGAGATGCAGAGGAACRGAAGAGGACAAGGAA
GAAGAATTGGTAACACAAACTTGCAGTCAGACATCAAGTGAAGACCCTGGAGACTCCACTCCATTTGAAGATTCGGAGGA
GTTTTGTTTTAGTGCTGAAGCCAATAGCTTTGATGCTGACGATGCTGATACTTACAATGAAGATGATGAAGAAGATGAAT
CAGAAACAGGCTACTGGATCACCTGCTGTGCC
Amytornis_striatus
??

??
??
??
??
??
??
??
??
??
??
??
??
??
????????????????????????????????
Pitta
AGGTCCTGCCCCACTGGTGTTTTCCTCCTTGACATAAAGCAGACTGAGCTCAAAATGAAACCTGCGTTCTTCTCCAAAGA
CTCATGTTACCTTCCCCCTCTCCGCTACCCTGCTCTTTGCACGCTCAGAAGCAATGCAAAGCCTGATGAGTACCAGTACA
TCATACATGGTGGTAAAACACCTAACAATGACCTTTCTGATAAGATTTACTTTATGAGTCTCGTAAGCAAAAATAGCAAG
AAAATGACGTTCCGATGCATTGAGAAAGATCTGGGTGGAGATGTCCCTGAAGCTAGATATGGGCATACCATTAATGTAGT
TCATAGCCGGGGAAAAAGCATGAGTGTTATATTTGGAGGSAGATCATATACTCCTCTTGCACAGAGAACCACTGAAAAAT
GGAACAGTGTAGTYGACTGTTTCCCGTCTGTATTTCTTGTTGATTTTGAGTTTGGATGCTGTACCTCATACATACTTCCA
GAGCTTCAAGATGGACTTTCTTTCCATGTTTCAATTGCCAGAGATGATACCATCTACATTTTAGGAGGCCATTCACTTCA
AAATAACACCAGGTGCCCCAACTTGTAYAAGCTAAAAGTTGATCTCCCACTGGGCAGCCCAGCTGTGACATGCACCATCT
TGCCAGGGGGGATATCTGTGTCAAGTGCTATAGTGACTCAAATCAGTGATACTGAATTTGTCCTTGTTGGTGGCTACCAC
TCTGACAACCAGAAACGGTTGGTGTGTAACACCATAGTTCTAGAAGATAGTAAGATAGAGATTGTTGAAAGGGTGAGCCC
AGAGTGGACACCAGATATTAAACACTGCAGAATATGGTTTGGCTGTGATATGGGCAAAGGGTCCATATTGCTTGGCATTC
CAGGGGCCAACAAACAGTTAATCTCAGATACAAACTACTTCTACATTTTGAGATGCAAAGGAGCASAAGAGGACAAGGAA
GAAGAATTGACAACGCAAATCTGTAGTCAGACATCATCTGAAGACCCTGGAGACTCCATTCCATTTGAAGATTCGGAGGA
GTTTTGTTTTAGTGCTGAAGCCAATAGCTTTGAMATTGATGATAATGACACTTACAATGAGGATGATGAAGACGACGAAT
CAGAAACAGGCTACTGGATCACCTGCTCTTCC
Toxorhamphus
AGATCCTGCCCCAATGGTGTTTTCCTCCTCGATATAAAGCAGAATGAGCTCAAAATGAAACCTGCCTTCTTTTCTAAAGA
CTCGTGTTACCTTCCCCCTCTCCGCTACCCTGCTCTTTGCACGCTCAGAAGTGATGCAAGGGCTGATGAGTACCAGTATA
TCATCCACGGTGGTAAAACACCTAACAATGACCTTTCTGATAAGATTTACTTTATAAGCCTGGTAAGCAAAACTAGCAAG
AAAATTACRTTCCAATGCATTGAGAAGGACTTGGGTGGAGATGTCCCTGAAGCTAGATATGGGCATACAATTAATGTAGT
TCATAGCCGGGGGAAAAGTGTGAGTGTTCTATTTGGAGGGAGGTCGTACACTCCTCTTGCACAAAGAACCACTGAAAAAT
GGAACAGTGTTGTTGACTGTTTGCCATCTGTGTTCCTCATTGATTTTGAGTTTGGATGCTGTACATCATACATACTTCCA
GAGCTTCAAGATGGACTTTCTTTCCATGTTTCAATTGCCAGAGGTGATACAATCTACATCTTGGGAGGCCACTCACTTCA
GAATAACACCAGGTCCCCCAACTTGTACAAGCTAAAAATTGATCTGCCCCTGGGCAGCCCAGCCGTGACCTGCACCATCC
TGCCAGGGGGGAAATCGGTGTCAAGTGCTATAGTGACCCAGGTCAGTGATACTGAATTCGTCCTTGTCGGTGGCTACCAC
TCTGACAACCAGAAACGCTTGGTGTGTAACACCATCATTCTGGAAGATGGTAAGATAGAGATTGTTGAAAGTGTGAGCCC
AGAGTGGACACCAGATATTAAACACTGCAGAATGTGGTTTGGGTGTGATATGGGTAAAGGGTCTGTTTTGCTGGGCATTC
CTGGGGCCAGCAAACAATTAATCCCAGATGCAAACTACTTCTACATCTTGAGATGCAAAGGAGCAGAGGACGACAAGGAA
GAAGAATTGATAACACAAATTTGCAKTCAGACGTCAAGTGAAGACCCTGGAGACTCCACTCCATTTGAAGATTCAGAGGA
GTTTTGTTTTAGTGCTGAAGCCAGCAGCTTTGACGTTGATGATGCTGACACTTACAATGAAGATGATGAAGAAGATGAAT
CAGAAACGGGCTACTGGATCACCTGCTGTGCC
Orthonyx_temminckii
AGATCCTGCCCCACTGGTGTTTTCCTCCTCGATATAAACCAGAGTGAGCTCAAAATGAAACCTGCCTTCTTCTCTAAAGA
CTCATGTTACCTTCCCCCTCTCCGCTACCCTGCTCTTTGCATGCTCAGAAGCGATGCAAAAGCTGATAAGTTCCAGTATA
TCATCCACGGTGGCAAAACACCTAACAATGACCTTTCTGACAAGATTTACTTTATGAGTCTGGTAAGCAAAACTAGCAAG
AAAATGACGTTCCAATGCATTGAGAAAGACCTGGGTGGAGATGTCCCTGAAGCTAGATATGGGCATACAATTAATGTAGT
TCATAGCCGGGGAAAAAGCATGAGTGTTCTATTTGGAGGGAGGTCCTATACTCCCCTTGCACAAAGAACCACTGAAAAAT
GGAACAGCGTAGTTGACTGTTTGCCATCTGTGTTTCTCATTGATTTTGAGTTTGGATGCTGTACATCATACATACTTCCA
GAGCTTCAAGATGGACTTTCTTTCCACGTTTCAATCGCTAGAGATGATACAATCTACATCTTGGGAGGCCATTCACTTCA
AAATAACACCAGGTCCCCCAACTTGTACAAGCTAAAAATTGATCTGCCTCTGGGCAGCCCAGCTGTGACCTGCACCATCC
TGCCAGGAGGATTATCAGTGTCAAGTGCTATAGTGACCCAGATCAGTGACACTGAATTTGTCCTTGTCGGTGGCTACCAC
TCTGACACCCAGAAACGGTTGGTGTGTAACACCATAGTTCTGGAAGATAGTAAGATAGAGATTGTTGAAAGTGTGAGCCC
AGAGTGGACACCAGATATTAAACACTGCAGAACGTGGTTTGGCTGTGATATGGGTAAAGGGTCTGTTTTGCTGGGCATTC
CCGGGGCCAACAAACAACTAATCCCAGATGCAAACTATTTCTACATTTTGAGATGCAAAGGAGCAGTAGAGGACAAGGAA
GAAGAATTGATAACACAAATTTGCAGTCAGACATCAAGTGAAGACCCTGGAGACTCCACTCCGTTTGAAGATTCGGAGGA

GTTTTGTTTTAGTGCTGAAGCCAATAGCTTTGACGTTGATGATGCTGACACTTACAATGAAGATGATGAAGAAGATGAAT
CAGAAACGGGCTACTGGATCACCTGCTGTGCC
Sericulus_chrysocephalus
??
??
??
??
??
??
??
??
??
??
??
??
??
??
????????????????????????????????
Cyclarhis_gujanensis
??
??
??
??
??
??
??
??
??
??
??
??
??
??
????????????????????????????????

[c-myc]
Polyborus_plancus   GCGAATGAATCCGAATC?AGCACAGAATCCAGC---
ACAGAGACGTCAGAAGAGCACAGTAAGCCCCACCACAGCCCGCTGGTTCTCAAACGGTGTCACGTCAACATCCATCAGCA
CAATTATGCCGCGCCTCCCTCCACCAAGGTTGAATACCCAGCTGCAAAAAGGCTAAAGTTGGACAGTGGCAGAGTTCTCA
AACAGATCAGCAACAACCG-AAAATGCTCGAGTCCCC---------
GAACGTCAGATTCGGAAGAGAACGACAAGAGGCGAACGCACAATGTCTTGGAGCGCCAGAGGAGAAACGAGCTGAAGCTG
AGTTTCTTTGCCTTGCGTGACCAGATACCCGAGGTGGCCAACAACGAAAAGGCGCCCAAGGTTGTCATCCTGAAAAAAGC
AACGGAGTACGTTCTTTCCATCCAGTCGGACGAACACAGACTGATCGCAGAGAAAGAGCAGTTGAGGCGGAGGAGAGAAC
AGTTGAAA
Falco   GCGAATGAATCTGAATCCAGCACAGAATCCAGC---
ACAGAGACGTCAGAAGAGCACAGTAAGCCCCACCACAGCCCGCTGGTTCTCAAACGGTGTCACGTCAACATCCATCAGCA
CAATTATGCCGCGCCTCCCTCCACCAAGGTTGAATACCCAGCTGCAAAAAGGCTAAAGTTGGACAGTGGCAGAGTTCTCA
AACAGATCAGCAACAACCG-AAAATGCTCGAGTCCCC---------
GCACGTCAGATTCAGAAGAGAACGACAAGAGGCGAACGCACAATGTCTTGGAGCGCCAGAGGAGAAATGAGCTGAAGTTG
AGTTTCTTTGCCTTGCGTGACCAGATACCCGAGGTGGCCAACAACGAAAAGGCGCCCAAGGTTGTCATCCTGAAAAAAGC
AACGGAGTACGTTCTTTCCATCCAGTCGGACGAACACAGACTGATTGCAGAGAAAGAGCAGTTGAGGCGGAGGAGAGAAC
AGTTGAAA
Tyrannus   TCAAATGAATCCGAATCAAGCACAGAGTCCAGC---
ACAGACACGTCAGAAGAGCACAGTAAGCTCCACCACAGCCCACTAGTTCTCAAACGGTGTCATGTCAACATCCATCAGCA
CAATTATGCCGCTCCTCCCTCTACTAAGGTTGAATACCCAGCTGCAAAAAGGCTAAGGTTGGACAGTGGCAGAGTTCTCA
AACAGATCAGCAACAACCG-AAAATGCTCGAGTCCGC---------
GCACATCAGATTCGGAAGAGAACGACAAGAGGCGAACACACAACGTCTTGGAGCGCCAGAGGAGAAATGAGCTGAAGCTG
AGTTTCTTTGCCTTGCGTGACCAGATACCTGAGGTGGCCAACAATGAGAAGGCACCCAAGGTTGTCATCCTGAAAAAAGC

AACAGAGTACGTTCTTTCCATCCAGTCAGATGAACACAGACTGATTGCAGAGAAAGAGCAGTTGAGGCGGAGGAGAGAAC
AGTTGAAA
Myiarchus GCAAATGAATCCGAATCAAGCACAGAGTCCAGC---
ACAGACACGTCAGAAGAGCACAGTAAGCTCCACCACAGCCCACTAGTTCTCAAACGGTGTCATGTCAACATCCATCAGCA
CAATTATGCCGCTCCTCCCTCTACTAAGGTTGAATACCCCGCTGCAAAAAGGCTAAGGTTGGACAGTGGCAGAGTTCTCA
AACAGATCAGCAACAACCG-AAAATGCTCGAGTCCGC---------
GCACATCAGATTCGGAAGAGAACGACAAGAGGCGAACACACAACGTCTTGGAGCGCCAGAGGAGAAATGAGCTGAAGCTG
AGTTTCTTTGCCTTGCGTGACCAGATACCTGAGGTGGCCAACAATGAGAAGGCACCCAAGGTTGTCATCCTGAAAAAAGC
AACAGAGTACGTTCTTTCCATCCAGTCAGATGAACACAGACTGATTGCAGAGAAAGAGCAGTTGAGGCGGAGGAGAGAAC
AGTTGAAA
Hypocnemis_cantator
??
??
??
??
??
??
??????????
Phlegopsis
??
??
??
??
??
??
??????????
Manacus_manacus GCAAATGAATCTGAATCAAGCACAGAGTCCAGC---
ACAGAGACGTCAGAAGAGCACAGTAAGCTCCACCACAGCCCACTAGTTCTCAAACGGTGTCATGTCAACATCCATCAGCA
CAATTATGCYGCTCCTCCCTCCACCAAGATTGAATACCCAGCTGCAAAAAGGCTAAGGTTGGACAGTGGCAGAGTTCTCA
AACAGATCAGCAACAACCG-AAAATGCTCGAGTCCGC---------
GCACGTCAGATTCAGAAGAGAACGACAAGAGGCGAACACACAACGTCTTGGAGCGCCAGAGGAGAAATGAGCTGAAGCTG
AGTTTCTTTGCCTTGCGTGACCAGATACCTGAGGTGGCCAATAATGAGAAGGCACCCAAGGTTGTCATCCTGAAAAAAGC
AACAGAGTACGTTCTTTCCATCCAGTCAGATGAACACAGACTGATCGCAGAGAAAGAGCAGTTGAGGCGGAGGAGAGAAC
AGTTGAAA
Acanthisitta_chloris GCAAATGAATCTGAATCCAGCACAGAGTCCAGC---
ACAGAGACGTCAGAAGAGCACAGTAAGCCCCACCACAGCCCACTGGTTCTCAAACGGTGTCACGTCAACATCCATCAGCA
CAATTATGCTGCTTCTCCGTCCACCAAGGTTGAATACCCAGCAGCAAAAAGGCTAAAGTTGGACAGTGGCAGAGTTCTCA
AACAGATCAGCAACAACCG-AAAATGCTCGAGTCCGC---------
GCACGTCAGATTCGGAAGAGAACGACAAGAGGCGAACGCACAATGTCTTGGAGCGCCAGAGGAGAAATGAGCTGAAGTTA
AGTTTCTTTGCCTTGCGAGACCAGATACCCGAGGTGGCCAACAACGGGAAGGCGCCCAAGGTTGTCATCCTGAAAAAAGC
AACGGAGTATGTTCTTTCCATCCAGTCAGATGAACACAGACTGATTGCAGAGAAAGAGCAGTTGAGGCGAAGGAGAGAAC
AGTTGAAA
Regulus
TCAAATGAGTATGAATCCAGCACAGAGTCCAGCAGTACAGAGACGTCAGAAGAGCACAGTAAGCCCCACCACAGTCCGCT
GGTCCTCAAACGGTGTCATGTCAACATCCATCAGCACAATTATGCCGCTCCTCCCTCCACCAAGGTTGAATACCCAGCTG
CAAAAAGGCTAAGGTTGGACAGTGGCAGAGTTCTCAAACAGATCAGCAACAACCG-AAAATGCTCCAGTCCGC-------
--
GCACGTCAGATTCGGAAGAGAACGACAAGAGGCGAACACACAACGTCTTGGAGCGCCAGAGGAGAAATGAGCTGAAGTTG
AGTTTCTTTGCCTTGCGTGACCAGATACCCGAGGTGGCCAACAATGAAAAGGCTCCCAAGGTTGTCATCCTGAAAAAAGC
AACAGAGTACGTTCTTTCCATCCAGTCGGATGAACACAGACTGATTGCAGAGAAAGAGCAGTTGAGGCGGAGGAGAGAAC
AATTGAAA
Ailuroedus GCAAATGAATATGAGTCCAGCACAGAGTCCAGC---
ACAGATACGTTAGAAGAACACAGTAAGCCCCACCACAGCCCACTGGTTCTCAAACGGTGTCATGTCAACATCCATCAGCA
CAATTATGCCGCTCCTCCCTCCACCAAGGTTGAATACCCAGCTGCAAAAAGGCTAAGGTTGGACAGTGGCAGAGTTCTCA
AACAGATCAGCAACAACCG-AAAATGCTCCAGTCCAC---------
GCACGTCGGATTCAGAAGAGAACGACAAGAGGCGAACGCACAACGTCTTGGAGCGCCAAAGGAGAAATGAGCTGAAGCTG
AGTTTCTTTGCCTTGCGTGACGAGATACCCGAGGTGGCCAACAACGAAAAGGCTCCCAAGGTTGTCATCCTGAAAAAAGC
AACAGAGTACGTTCTTTCCATCCAGTCGGATGAACACAGACTGATTGCAGAGAAGGAGCAGTTGAGGCGG?GGAGAGAAC
AGTTGAAG

Zosterops
GCAAATGAATATGAATCCAGCACAGAGTCCAGCAGCACAGAGACGTCAGAAGAGCACAGTAAGCCCCACCACAGCCCGCT
GGTTCTCAAACGGTGTCATGTCAACATCCATCAGCACAATTATGCCGCTCCTCCCTCCACCAAGGTTGAATACCCAGCTG
CGAAAAGGCTAAGGTTGGACAGTGGCAGAGTTCTCAAACAGATCAGCAACAACCG-AAAATGCTCCAGTCCGC-------
--
GCACGTCAGATTCGGAGGAGAACGACAAGAGGCGAACGCACAACGTCTTGGAGCGCCAGAGGAGAAACGAGCTGAAGTTG
AGTTTCTTTGCCTTGCGTGACCAGATACCCGAGGTGGCCAACAATGAAAAGGCTCCCAAGGTTGTCATCCTGAAAAAAGC
AACAGAGTACGTTCTTTCCATCCAGTCGGATGAACACAGACTGATTGCAGAGAAAGAGCAGTTGAGGCGGAGGAGAGAAC
AGTTGAAA
Onychorhynchus
??
??
??
??
??
??
??????????
Nestor_notabilis
??
??
??
??
??
??
??????????
Leiothrix_argentauris
??
??
??
??
??
??
??????????
Troglodytes
GCAAATGAATATGAATCCAGCACAGAGTCCAGCAGCACAGAGACGTCAGAAGAGCACAGTAAGCCCCACCACAGCCCACT
GGTTCTCAAACGGTGTCATGTCAACATCCATCAGCACAATTATGCCGCTCCTCCCTCCACCAAGGTTGAATACCCAGCTG
CAAAAAGGCTAAGGTTGGACAGTGGCAGAGTTCTCAAACAGATCAGCAACAACCG-AAAATGCTCCAGTCCGC-------
--
GCACGTCAGATTCGGAAGAGAACGACAAGAGGCGAACACACAACGTCTTGGAGCGCCAGAGGAGAAATGAGCTGAAGTTG
AGTTTCTTTGCCTTGCGTGACCAGATACCCGAGGTGGCCAACAATGAAAAGGCTCCCAAGGTTGTCATCCTGAAAAAAGC
AACAGAGTACGTTCTTTCCATCCAGTCGGATGAACACAGACTGATTGCAGAGAAAGAGCAGTTGAGGCGGAGGAGAGAAC
AGTTGAAA
Luscinia_svecica
??
??
??
??
??
??
??????????
Psittacus_erithacus
??
??
??
??
??
??
??????????
Ficedula_hypoleuca
GCAAATGAATATGAATCCAGCACAGAGTCCAGCAGCACGGAGATGTCAGAAGAGCACAGTAAGCCCCACCACAGCCCACT

GGTTCTCAAACGGTGTCATGTCAACATCCATCAGCACAATTATGCCGCTCCTCCCTCCACCAAGGTTGAATACCCAGCAG
CAAAAAGGCTAAGGTTGGACAGTGGCAGAGTTCTCAAACAGATCAGCAACAACCG-AAAATGCTCCAGCCCGC-------
--
GCACGTCAGACTCGGAGGAGAACGACAAGAGGCGAACGCACAACGTCTTGGAGCGCCAGAGGAGGAATGAGCTGAAGTTG
AGTTTCTTTGCCTTGCGTGACGAGATACCCGAGGTGGCCAACAATGAAAAGGCTCCCAAGGTTGTCATCCTGAAAAAAGC
AACAGAGTACGTTCTTTCCATCCAGTCAGATGAGCACAGACTGATTGCAGAGAAAGAGCAGTTGAGGAGGAGGAGAGAAC
AGTTGAAA
Menura_novaehollandiae   GCGAACGAATATGAATCCAGCACAGAGTCCAGC---
ACAGATACACCAGAAGAGCACAGTAAGCCCCACCACAGCCCGCTGGTTCTCAAAAGGTGTCATGTCAACATCCACCAGCA
CAATTATGCCGCTCCTCCCTCCACTAAGGCTGAATACCCAGCTGCAAAAAGGCTAAGGTTGGACAGTGGCAGAGTTCTCA
AACAGATCAGCAACAACCG-AAAATGCTCCAGTCCGC---------
GCACGTCAGATACGGAAGAGAACGACAAGAGGCGAACGCACAACGTCTTGGAGCGCCAGAGGAGAAATGAGCTGAAGCTG
AGTTTCTTTGCCTTGCGTGACCAGATACCCGAGGTGGCCCACAACGAAAAGGCTCCCAAGGTTGTCATCCTGAAAAAAGC
AACAGAGTACGTTCTTTCCATCCAGTCAGATGAACACAGACTGATTGCAGAGAAAGAGCAGTTGAGACGGAGGAGAGAAC
AGTTGAAA
Pycnonotus
GCAAATGAATATGAATCCAGCACAGAGTACAGCAGCACAGAGACGTCAGAAGAGCACAGTAAGCCCCATCACAGCCCGCT
TGTTCTTAAACGGTGTCATGTCAACATCCATCAGCACAATTATGCCGCTCCTCCCTCCACCAAGGTTGAATACCCAGCAG
CAAAAAGGCTAAGGTTGGACAGTGGCAGAGTTCTCAAACAGATCAGCAACAATCG-AAAATGCTCCAGTCCAC-------
--
GCACATCAGATTCGGAGGAGAACGACAAGAGGCGAACACACAACGTCTTGGAGCGCCAGAGGAGAAATGAGCTGAAGTTG
AGTTTCTTTGCCTTGCGTGACCAGATACCTGAAGTGGCCAACAATGAAAAGGCTCCCAAGGTTGTCATCCTGAAAAAAGC
AACAGAGTACGTTCTTTCCATCCAGTCGGATGAACACAGACTGATTGCAGAGAAAGAGCAGTTGAGACGGAGGAGAGAAC
AGTTGAAA
Donacobius_atricapilla
??
??
??
??
??
??
??????????
Hirundo_rustica
GCAAATGAATATGAATCCAGCACAGAGTCCAGCAGCACAGAGACCTCAGAAGAGCACAGTAAGCCCCACCACAGCCCGCT
GGTTCTCAAACGGTGTCATGTCAACATCCATCAGCACAATTATGCCGCTCCTCCCTCCACCAAGGCTGAATACCCAGCTG
CAAAAAGGCTACGGTTGGACAGTGGCAGAGTTCTCAAACAGATCAGCAACAACCG-AAAATGCTCCAGTCCAC-------
--
GCACGTCAGATTCAGAAGAGAACGACAAGAGGCGAACGCACAATGTCTTGGAGCGCCAGAGAAGAAATGAGCTGAAGTTG
AGTTTCTTTGCCTTGCGTGACCAGATACCCGAGGTGGCCAACAATGAAAAGGCTCCCAAGGTTGTCATCCTGAAAAAAGC
AACAGAGTACGTTCTTTCTATCCAGTCAGATGAACACAGACTGATTGCAGAAAAAGAGCAGTTGAGGCGGAGGAGAGAAC
AGTTGAAA
Lichenostomus
??
??
??
??
??
??
??????????
Cnemophilus
??
??
??
??
??
??
??????????
Coracina
??
??

??
??
??
??
??????????
Dicrurus   GCGAATGAATACGAATCCAGCACAGAGTCCAGC---
ACAGACACGTCGGAAGAGCACAGTAAGCCCCACCACAGCCCACTGGTTCTCAAACGGTGTCATGTCAACATCCATCAGCA
CAATTATGCCGCTCCTCCCTCTACCAAGGTTGAATACCCAGCTGCAAAAAGGCTAAGGTTGGACAGTGCCAGAGTTCTCA
AACAGATCAGCAACAACCG-AAAATGCTCCAGTCCGC---------
GTACATCAGATTCGGAAGAGAATGACAAGAGGCGAACACACAATGTCTTGGAGCGCCAGAGGAGAAATGAGCTGAAGTTG
AGTTTCTTTGCCTTGCGTGACCAGATACCTGAGGTGGCCAACAATGAAAAGGCTCCCAAGGTTGTAATTCTGAAAAAAGC
AACAGAGTATGTTCTTTCCATCCAGTCGGATGAACACAGACTGATTGCAGAGAAAGAGCAGTTGAGGAGGAGGAGAGAAC
AGTTGAAA
Gerygone_fusca
??
??
??
??
??
??
??????????
Ptiloris_magnificus   GCGAACGAATACGAATCCAGCACAGAGTCCAGC---
ACAGACACGTCAGAAGAGCACAGTAAGCCCCATCACAGCCCGCTGGTTCTCAAACGGTGTCATGTCAACATCCATCAGCA
CAATTATGCCGCTCCTCCCTCCACCAAGGTTGAACACCCAGCTGCAAAAAGGCTAAGGTTGGACAGTGGCAGAGTTCTCA
AACAGATCAGCAACAACCG-AAAATGCTCCAGTCCGC---------
GCACATCAGATTCGGAAGAGAACGACAAGAGGCGAACGCACAATGTCTTGGAGCGCCAGAGGAGAAATGAGCTGAAGTTG
AGTTTCTTTGCCTTGCGTGACCAGATACCTGAGGTGGCCAACAATGAAAAGGCTCCCAAGGTTGTCATCCTGAAAAAAGC
AACAGAGTACGTTCTTTCCATCCAGTCGGATGAACACAGACTGATTGCAGAGAAAGAGCAGTTGAGGCGGAGGAGAGAAC
AGTTGAAA
Paradisaea_raggiana
??
??
??
??
??
??
??????????
Cyanocorax_chrysops
??
??
??
??
??
??
??????????
Lonchura
GCAAATGAATATGAATCCAGCACAGAGTCCAGCAGCACAGAGACATCAGAAGAGCACAGTAAGCCCCACCAAAGCCCGCT
GGTTCTCAAACGGTGTCACGTCAACATTCATCAGCACAATTATGCCGCTCCTCCCTCCACCAAGGTTGAATACCCAGCTG
CAAAAAGGCTAAGGTTGGACAGTGGCAGAGTACTCAAACAGATCAGCAACAACCG-AAAATGCTCCAGTCCAC-------
--
GCACTTCAGATTCGGAAGAGAACGACAAGAGGCGAACACACAACGTCTTGGAGCGACAGAGGAGAAATGAGCTGAAGTTG
AGTTTCTTTGCCTTGCGTGACGAGATACCCGAGGTGGCCAACAATGAAAAGGCTCCCAAGGTTGTTATCCTGAAAAAAGC
GACAGAGTACGTTCTTTCCATCCAGTCAGAGGAACACAGACTGATTGCAGAGAAAGAGCAGTTGAGGCGGAGGAGAGAAC
AGTTGAAA
Phylloscopus
??
??
??
??
??

??
??????????
Turdus
GCAAATGAATATGAATCCAGCACAGAGTCCAGCAGCACAGAGATGTCAGAAGAGCACAGTAAGCCCCACCACAGCCCGCT
GGTTCTCAAACGGTGTCATGTCAACATTCATCAGCACAATTATGCTGCTCCTCCCTCCACCAAGGTTGAATACCCAGCTG
CAAAAAGGCTAAGGTTGGACAGTGGCAGAGTTCTCAAACAGATTAGCAACAACCG-AAAATGCTCCAGTCCAC-------
--
GCACGTCAGATTCGGAAGAGAATGACAAGAGGCGAACACACAATGTCTTGGAGCGCCAGAGGAGAAATGAGCTCAAGTTG
AGTTTCTTTGCCTTGCGTGACGAGATACCCGAGGTGGCCAACAATGAAAAGGCTCCCAAGGTTGTCATCCTGAAAAAAGC
AACAGAGTACGTTCTTTCTATCCAGTCGGATGAACACAGACTGATTGCAGAGAAAGAGCAGTTGAGGAGGAGGAGAGAAC
AGTTGAAA
Acrocephalus
??
??
??
??
??
??
??????????
Sitta
GCAAATGAATATGAATCCAGCACAGAGTCCAGCAGCACAGAGACGTCAGAAGAGCACAGTAAGCCCCACCACAGCCCGCT
GGTTCTCAAACGGTGTCATGTCAACATCCATCAGCACAATTATGCCGCTCCTCCCTCTACCAAGGTTGAATACCCAGCTG
CAAAAAGGCTAAGGTTGGACAGTGGCAGAGTTCTCAAACAGATCAGCAACAACCG-AAAATGCTCCAGTCCGC-------
--
GCACATCAGATTCGGAAGAGAACGACAAGAGGCGAACACACAATGTCTTGGAGCGCCAGAGGAGAAATGAGCTGAAGTTG
AGTTTCTTTGCCTTGCGTGACCAGATACCCGAGGTGGCCAACAATGAAAAGGCTCCCAAGGTTGTCATCCTGAAAAAAGC
AACAGAGTACGTTCTTTCCATCCAGTCGGATGAACACAGACTGATTGCAGAGAAAGAGCAGTTGAGGCGGAGGAGAGAAC
AGTTGAAA
Mimus
GCAAATGAATATGAATCCAGCACAGAGTCCAGCAGCACAGAGATGTCAGAAGAGCACAGTAAGCCCCACCACAGCCCTCT
GGTTCTCAAACGGTGTCATGTCAACATCCATCAGCACAATTATGCCGCTCCTCCCTCCACCAAGGTTGAATACCCAGCTG
CAAAAAGGCTAAGGTTGGACAGTGGCAGAGTTCTCAAACAGATCAGCAACAACCG-AAAATGCTCCAGTCCAC-------
--
GCACGTCAGATTCGGAAGAGAACGACAAGAGGCGAACACACAATGTCTTGGAGCGCCAGAGGAGGAATGAGCTGAAGTTG
AGTTTCTTTGCCTTGCGTGACGAGATACCTGAGGTGGCCAACAATGAAAAGGCTCCCAAGGTTGTCATCCTGAAAAAAGC
AACAGAGTACGTTCTTTCCATCCAGTCGGATGAACACAGACTGATTGCAGAGAAAGAGCAGTTGAGGAGGAGGAGAAAAC
AGTTGAAA
Sturnus_vulgaris
GCAAATGAATATGAATCCAGCACAGAGTCCAGCAGCACAGAGATGTCAGAAGAGCACAGTAAGCCCCACCACAGCCCGCT
GGTTCTCAAACGGTGTCATGTCAACATCCATCAGCACAATTATGCCGCTCCTCCCTCCACCAAGGTTGAATACCCAGCTG
CAAAAAGGCTAAGGTTGGACAGTGGCAGAGTTCTCAAACAGATCAGCAACAACCG-AAAATGCTCCAGTCCAC-------
--
GCACATCAGATTCGGAAGAGAACGACAAGAGGCGAACACACAATGTCTTGGAGCGCCAGAGGAGAAATGAGCTGAAGTTG
AGTTTCTTTGCCTTGCGTGATGAGATACCCGAGGTAGCCAACAATGAAAAGGCTCCCAAGGTTGTCATTCTGAAAAAAGC
AACAGAGTACGTTCTTTCCATCCAGTCAGATGAACACAGACTGATTGCAGAGAAAGAGCAGTTGAGGAGGAGGAGAGAAC
AGTTGAAA
Creadion_carunculatus
??
??
??
??
??
??
??????????
Parus
GCAAATGAATATGAATCCAGCACAGAGTCCAGCAGCACAGAGACGTCAGAGGAGCACAGTAAGCCCCACCACAGCCCGCT
GGTTCTCAAACGGTGTCATGTCAACATCCATCAGCACAATTATGCCGCTCCTCCCTCCACCAAGGTTGCATACCCAGCTG
CAAAAAGGCTAAGGTTGGACAGTGGCAGGGTTCTCAAACAGATCAGCAACAACCG-AAAATGCTCCAGTCCGC-------
--
GCACATCAGATTCGGAGGAGAACGACAAGAGGCGAACACACAATGTCTTGGAGCGCCAGAGGAGGAATGAGCTCAAGTTG

AGTTTCTTTGCCTTGCGTGACGAGATACCCGAGGTGGCCAACAATGAAAAGGCTCCCAAGGTTGTCATCCTGAAAAAAGC
AACAGAGTACGTTCTTTCCATCCAGTCGGATGAGCACAGACTGATTGCAGAGAAAGAGCAGTTGAGGCGGAGGAGAGAAC
AGTTGAAA
Petroica_rosea GCGAATGAGTATGAATCCAGCACGGAGTCCAGC---
ACAGACACGTCAGAAGAGCACAGTAAGCCCCACCACAGCCCACTGGTTCTCAAACGGTGTCATGTCAACATCCATCAGCA
CAATTATGCCGCTCCTCCCTCCACCAAGGTTGAATACCCAGCTGCAAAAAGGCTAAGGTTGGACAGTGGCAGAGTTCTCA
AACAGATCAGCAACAACCG-AAAATGCTCCAGTCCAC---------
GCACGTCAGATTCGGAAGAGAACGACAAGAGGCGAACACACAATGTCTTGGAGCGCCAGAGGAGAAATGAGCTGAAGTTG
AGTTTCTTTGCCTTGCGTGACGAGATACCCGAGGTTGCCAACAATGAAAAGGCTCCCAAGGTTGTCATCCTGAAAAAAGC
AACAGAATACGTTCTTTCCATCCAGTCAGATGAACACAGACTGATTGCAGAGAAAGAGCAGTTGAGGCGGAGGA????AC
AGTTGAAA
Eopsaltria_australis TCGAATGAATATGAATCCAGCACAGAGTCCAGC---
ACAGACACATCAGAAGAGCACAGTAAGCCCCACCACAGCCCGCTGGTTCTCAAACGGTGTCATGTCAACATCCATCAGCA
CAATTACGCTGCTCCTCCCTCCACCAAGGTTGAATACCCAGCTGCGAAAAGGCTAAGGTTGGACAGTGGCAGAGTTCTCA
AACAGATCAGCAACAACCG-AAAATGCTCCAGTCCGC---------
GCACGTCAGATTCGGAAGAGAATGACAAGAGGCGAACACACAACGTCTTGGAGCGCCAGAGGAGAAATGAGCTGAAGTTG
AGTTTCTTTGCCTTGCGTGACGAGATACCCGAGGTGGCCAACAATGAAAAGGCTCCCAAGGTTGTCATCCTGAAAAAAGC
AACAGAGTACGTTCTTTCTATCCAGTTGGATGAGCACAGACTGATTGCAGAGAAAGAGCAGTTGAAGCGGAGGAGAGAAC
AGTTGAAA
Serinus
GCAAATGAGTACGAATCCAGCACAGAGTCCAGCAGCACAGAGACATCAGAAGAGAACAGTAAGCCCCACCAAAGCCCGCT
AGTTCTCAAAAGGTGTCATGTCAACATCCATCAGCACAATTATGCTGCTCCTCCCTCCACCAAGGTTGAATACCCAGCTG
CAAAAAGGCTAAGGTTGGACAGTGGCAGAGTTCTCAAACAGATCAG-
AACAACCGAAAAATGCTCCAGTCCGCGCACGTCAGGCTCGTCAGATTCGGAAGAGAACGACAAGAGGCGAACACACAATG
TCTTGGAGCGCCAGAGGAGAAATGAGCTGAAGTTGAGTTTCTTTGCCTTGCGTGACGAAATACCCGAGGTGGCCAACAAT
GAAAAGGCTCCCAAGGTTGTCATCCTGAAAAAAGCAACGGAGTATGTTCTTTCCATCCAGTCAGAGGAACACAGACTGAT
TGCAGAGAAAGAGCAGTTGAGACGGAGGAGAGAACAGTTGAAA
Icterus
??
??
??
??
??
??
??????????
Motacilla
GCAAATGAATATGAATCTAGCACAGAGTCCAGCAGCACAGAAACATCAGAAGAGCACAGTAAGCCCCACCAAAGCCCGCT
GGTTCTCAAACGGTGTCACGTCAACATCCATCAGCACAATTATGCCGCTCCTCCCTCCACCAAGGTTGAATACCCAGCTG
CAAAAAGGCTAAGGTTGGACAGTGGCAGAGTTCTCAAACAGATCAGCAACAACCG-
AAAATGCTCCAGTCCGCGCACGTCAGGCACGTCAGATTCGGAAGAGAACGACAAGAGGCGAACCCACAATGTCTTGGAGC
GCCAGAGGAGAAATGAGCTGAAGTTGAGTTTCTTTGCCTTGCGTGACGAGATACCCGAGGTGGCCAACAATGAAAAGGCT
CCCAAGGTTGTCATCCTGAAAAAAGCGACAGAGTACGTTCTTTCCATCCAGTCAGAGGAACACAGACTGATTGCAGAGAA
AGAGCAGTTGAGGCGGAGGAGAGAACAGTTGAAA
Emberiza
GCAAATGAGTATGAATCCAGCACAGAGTCCAGCAGCACAGAGACATCAGAAGAGCACAGTAAGCCCCACCAAAGCCCGCT
GGTCCTCAAACGGTGTCATGTCAACATCCATCAGCACAATTACGCCGCTCCTCCCTCCACCAAGGTTGAATACCCAGCTG
CAAAAAGGCTAAGGTTGGAAAGTGGCAGAGTTCTCAAACAGATCAGCAACAACCG-
AAAATGCTCCAGTCCGCGCACGTCAGGCTCRTCAGATTCGGAAGAGAACGACAAGAGGCGAACGCACAATGTCTTGGAGC
GCCAGAGGAGAAATGAGCTGAAGTTGAGTTTCTTTGCCTTGCGTGACGAGATACCCGAGGTGGCCAACAATGAAAAGGCT
CCCAAGGTTGTCATCCTGAAAAAAGCGACAGAGTACGTTCTTTCCATCCAGTCGGAAGAGCACAGACTGATTGCAGAGAA
AGAGCAGTTGAGGCGGAGGAGAGAACAGTTGAAA
Pomatostomus GCAAATGAATATGAGTCCAGCACGGACTTCAGC---
ACAGACACATCAGAAGAGCACAGTAAGCCCCACCACAGCCCGCTGGTTCTCAAACGGTGTCATGTCAACATCCATCAGCA
CAATTATGCCGCTCCTCCCTCCACCAAGGTTGAATACCCAGCTGCGAAAAGGCTAAGATTGGACAGTGGCAGAGTTCTCA
AACAGATCAGCAACAACCG-AAAATGCTCCAGTCCGC---------
GCACGTCAGATTCAGAAGAGAACGACAAGAGGCGAACGCACAATGTCTTGGAGCGCCAGAGGAGAAATGAGCTGAAGTTG
AGTTTCTTTGCCTTGCGTGACGAGATACCCGAGGTGGCCAACAATGAAAAGGCTCCCAAGGTTGTCATCCTGAAAAAGGC
AACAGAGTACATTCTTTCCATCCAGTCGGATGAACACAGACTGATTGCAGAGAAAGAGCAGTTGAGGCGGAGGAGAAAAC
AGTTGAAA

```
Rhipidura
????????????????????????????????????????????????????????????????????????????????
????????????????????????????????????????????????????????????????????????????????
????????????????????????????????????????????????????????????????????????????????
????????????????????????????????????????????????????????????????????????????????
????????????????????????????????????????????????????????????????????????????????
????????????????????????????????????????????????????????????????????????????????
??????????
Pica_pica                 GCGAATGAATACGAATCCAGCACAGAGTCCAGC---
ACAGACACGTCAGAAGAGCACAGTAAGCCCCACCACAGCCCGCTGGTTCTCAAACGGTGTCATGTCAACATCCATCAGCA
CAATTATGCCGCTCCTCCCTCCACCAAGGTTGAATACCCAGCTGCAAAAAGGCTAAGGTTGGACAGTGGCAGAGTTCTCA
AACAGATCAGCAACAATCG-AAAATGTTCCAGTCCGC---------
GCACATCAGATTCGGAAGAGAACGACAAGAGGCGAACACACAACGTCTTGGAGCGCCAGAGGAGAAATGAGCTGAAGTTG
AGTTTCTTTGCCTTGCGTGACCAGATACCTGAGGTGGCCAACAACGAAAAGGCGCCCAAGGTTGTCATCCTGAAAAAAGC
AACAGAGTACGTTCTTTCCATCCAGTCAGATGAACACAGACTGATTGCAGAGAAAGAGCAGTTGAGACGGAGGAGAGAAC
AGTTGAAA
Manucodia                 GCGAATGAATACGAATCCAGCACAGAGTCCAGC---
ACAGACACGTCAGAAGAGCACAGTAAGCCCCACCACAGCCCGCTGGTTCTCAAACGGTGTCATGTCAACATCCATCAGCA
CAATTATGCCGCTCCTCCCTCCACCAAGGTTGAATACCCAGCTGCAAAAAGGCTAAGGTTGGACAGTGGCAGAGTTCTCA
AACAGATCAGCAACAACCG-AAAATGCTCCAGTCCGC---------
GCACATCAGATTCGGAAGAGAACGACAAGAGGCGAACGCACAATGTCTTGGAGCGCCAGAGGAGAAATGAGCTGAAGTTG
AGTTTCTTTGCCTTGCGTGACCAGATACCTGAGGTGGCCAACAATGAAAAGGCTCCCAAGGTTGTCATCCTGAAAAAAGC
AACAGAGTACGTTCTTTCCATCCAGTCGGATGAACACAGACTGATTGCAGAGAAAGAGCAGTTGAGGCGGAGGAGAGAAC
AGTTGAAA
Corvus_corone             GCGAATGAATATGAATCCAGCACAGAGTCCAGC---
ACAGACACATCAGAAGAGCACAGTAAGCCCCACCACAGCCCGCTGGTTCTCAAACGGTGTCATGTCAACATCCATCAGCA
CAATTATGCTGCTCCTCCCTCCACCAAGGTTGAATACCCAGCTGCAAAAAGGCTAAGGTTGGACAGTGGCAGAGTTCTCA
AGCAGATCAGCAACAACCG-AAAATGTTCCAGTCCGC---------
GCACATCAGATTCGGAAGAGAACGACAAGAGGCGAACGCACAATGTCTTGGAGCGCCAGAGGAGAAATGAGCTGAAGTTG
AGTTTCTTTGCCTTGCGTGACCAGATACCTGAGGTGGCCAACAATGAAAAGGCTCCCAAGGTTGTCATCCTGAAAAAAGC
AACAGAGTACGTTCTTTCCATCCAGTCAGATGAACACAGACTGATTGCAGAGAAAGAGCAGTTGAGACGGAGGAGAGAAC
AGTTGAAA
Vireo
????????????????????????????????????????????????????????????????????????????????
????????????????????????????????????????????????????????????????????????????????
????????????????????????????????????????????????????????????????????????????????
????????????????????????????????????????????????????????????????????????????????
????????????????????????????????????????????????????????????????????????????????
????????????????????????????????????????????????????????????????????????????????
??????????
Camptostoma_obsoletum
????????????????????????????????????????????????????????????????????????????????
????????????????????????????????????????????????????????????????????????????????
????????????????????????????????????????????????????????????????????????????????
????????????????????????????????????????????????????????????????????????????????
????????????????????????????????????????????????????????????????????????????????
????????????????????????????????????????????????????????????????????????????????
??????????
Promerops_cafer
????????????????????????????????????????????????????????????????????????????????
????????????????????????????????????????????????????????????????????????????????
????????????????????????????????????????????????????????????????????????????????
????????????????????????????????????????????????????????????????????????????????
????????????????????????????????????????????????????????????????????????????????
????????????????????????????????????????????????????????????????????????????????
??????????
Oriolus                   GCGAATGAATACGAATCCAGCACAGAGTACAGC---
ACAGACACGTCAGAAGAGCACAGTAAGCCCCACCACAGCCCACTAGTTCTCAAACGGTGTCATGTCAACAGCCATCAGCA
CAATTATGCCGCTCCTCCCTCCACCAAGGTTGAATACCCAGCTGCAAAAAGGCTAAGGTTGGACAGTGGCAGAGTTCTCA
AACAGATCAGCAACAACCG-CAAATGCTGCAGTCCGC---------
```

GCACATCAGATTCGGAAGAGAACGACAAGAGGCGAACACACAATGTCTTGGAGCGCCAGAGGAGAAATGAGCTGAAGTTG
AGTTTCTTTGCCTTGCGTGATGAGATACCTGAGGTGGCCAACAATGAAAAGGCTCCCAAGGTTGTCATCCTGAAAAAAGC
AACAGAGTATGTTCTTTCCATCCAGTCGGATGAACACAGACTGATTGCAGAGAAAGAGCAGTTGAGGCGGAGGAGAGAAC
AGTTGAAA
Dendroica
??
??
??
??
??
??
??????????
Nectarinia
TCAAATGAATATGAATCCAGCACAGAGTCCAGCAGCACAGAGACGTCAGAAGAGCACAGTAAGCCCCACCAAAGCCCGCT
GGTTCTCAAACGGTGTCATGTCAACATCCATCAGCACAATTATGCCGCTCCTCCCTCCACCAAGGTTGAATACCCAGCTG
CAAAAAGGCTAAGGTTGGACAGTGGCAGAGTTCTCAAACAGATCAGCAACAACCG-AAAATGCTCCAGTCCGC-------
--
GCACGTCAGATTCGGAAGAGAATGACAAGAGGCGAACGCACAACGTCTTGGAGCGCCAGAGGAGGAACGAGCTGAAGTTG
AGTTTCTTTGCCTTGCGTGACGAGATACCTGAGGTGGCCAACAATGAAAAGGCTCCCAAGGTTGTCATCCTGAAAAAAGC
AACAGAGTACGTTCTTTCCATCCAGTCGGATGAACACAGACTGATTGCAGAGAAAGAGCAGTTGAGGCGGAGGAGAGAAC
AGTTGAAA
Amytornis_striatus
??
??
??
??
??
??
??????????
Pitta   GCAAATGAATCTGAATCAAGCACAGAGTCCAGC---
ACAGAGACATCAGAAGAGCACAGTAAGCCCCACCACAGCCCACTAGTTCTCAAACGGTGTCATGTCAACATCCATCAGCA
CAATTATGCTGCTCCTCCCTCCACCAAGGTTGAATACCCAGCTGCAAAAAGGCTAAGGTTGGACAGTGGCAGAGTTCTCA
AACAAATTAGCAACAACCG-AAAATGCTCGAGTCCGC---------
GCACGTCAGATTCGGAAGAGAACGACAAGAGGCGAACACACAATGTCTTGGAGCGCCAGAGAAGAAATGAGCTGAAGCTG
AGTTTCTTTGCATTGCGTGACCAGATACCTGAGGTAGCTAACAACGAAAAGGCACCCAAGGTTGTCATCCTGAAAAAAGC
AACAGAGTATGTTCTTTCTATTCAGTCAGATGAACACAGACTGATTGCAGAGAAAGAGCAATTGAGGCAGAGGAGAGAAC
AGTTGAAA
Toxorhamphus
??
??
??
??
??
??
??????????
Orthonyx_temminckii   GCGGCTGAATACGAATCCAGCACAGAGTCCAGC---
ATAGACGTATCAGAAGAGCACAGTAAGCCCCACCACAGCCCGCTGGTTCTCAAACGGTGTCATGTCAACATCCATCAGCA
CAATTATGCCGCTCCTCCCTCCACCAAGGTTGAATATCCAGCTGCAAAAAGGCTAAGGTTGGACAGTGGCAGAGTTCTCA
AACAGATTAGCAACAACCG-AAAATGCTCCAGTCCGC---------
GCACGTCAGATTCGGAAGAGAATGACAAGAGGCGAACGCACAACGTCTTGGAGCGCCAGAGGAGAAATGAGCTGAAGTTG
AGTTTCTTTGCCTTGCGTGACGAGATACCCGAGGTGGCCAACAATGAAAAGGCTCCCAAGGTTGTCATCCTAAAAAAAGC
AACAGAGTATGTTCTTTCCATCCAGTCAGATGAACACAGACTGATTGCAGAGAAGGAGCAGTTGAGGCGGAGGAGAGAAC
AGTTGAAA
Sericulus_chrysocephalus
??
??
??
??
??

??
??????????
Cyclarhis_gujanensis
??
??
??
??
??
??
??????????

[TGFß2]
Polyborus_plancus
??
??
??
??
??
??
??
??
?????????????
Falco   CCTATTGTTTTAGGTAACTATGCATCC---
GTATTCAGTATACTTACGCAGTAGTACCAGCCTGTGAGGTGTAATGCAGTTG-
CTTATCCTGTTTCTTAACAGTTGGCATTTGTACAGAGCATGGACCCTCATTTGTAGACCTACAGGGTCTGTGTGCAAAGA
GTACCTGCCCAGCAGTCTCCAGTCCCACAGAGACG-GTGGCAGAT-
GAGGAAGATTGTGGCCTTAGAAAGCAGAAGCCGAGT----TCCATCTGGGATGATTCCCAGACCCATCAGCCCCATAAT-
-T-ACAGCCAAATTATCACTTCCACTGTGCTCACCTATTCAGAGAGGG--CACTC--AGTAGGACCTGGAGAAAA-
ATACT------AGTCCTTCAGGGAAACCGTTAGCTTTTTCTGTATAAGCAAGTCTTGCCATGTTTAAGCATCT-------
-----GAGTTCAGGTG---
CAGTATAAACTACTGTCAATGTAATTTTTTTTGTAAATTTTTGGGACTTGGATTTGCCTGCCTGTAAAGCATCATT--
CAGAGAGCAAG--CAACCGGATTATTTTTAT----------------------
TCCCTGTGTTGCCTTTCTTATGACCGATGTGTTTGTCTGTATTTTATTTCCCAGGAAC
Tyrannus   CCTATTGTTTTAGGTAACTATRCATCT---
GTATTCAATATAMTTACATAATCATACCAACCTGTGAGGTGTCTTACAGTTG-
TTTATCATGTTTCTTTACAGTTGGTATTTTTACAGAACGCACCCCCTCATTTGCAGACCCACGGGGTCTGTG--
CAGCAGGTGTCTGCCCAGTGGTCTCCAGTCCCACAGAGACA-GTGGCAGATGAGGGA-
GGTTGTGACCTTAGACAACAGAGGCTGART----
TCCATCTGGGATGATTCCCAGATGCGTTAGTCCCATAATAATTACAGCCAAATTATCACCTCCACTTTGCTCACCTACTC
AGAGAGTC--CCCTC--TGTAGGACCTGACGAAAA-
AAACTTGACTAAGTGCTCCAGAGAAATCTTTAGCTTTGTCTCTA-AAGCAAGTCCTGCCATGTCTRGGCGTCC-------
-----AGTTTTAGTTA---CAGTATAAGCTACTGTTGATTCAA-
TTTGGTTGAGAATTTTTGGGACTCAGGCTTGCCTGCCTGTAAAGCATCATT--CAGGGGGTGAG--
CARCTGGATTATTTTCAT----------------------
TCCCTGTGTTGTTTTTATTATGACCGATGGGTTTGTCTGTACTTTATTTCCAAGGAAT
Myiarchus
??
??
??
??
??
??
??
??
?????????????
Hypocnemis_cantator
??
??
??

??
??
??
??
??
?????????????
Phlegopsis
??
??
??
??
??
??
??
??
?????????????
Manacus_manacus
??
?????????????????????????????????????GAACTCATCCCCTCATTTGCAGACCCACGGGGTCTGTG--
CAGCAGGTGTCTGCCCAGTGGTCTCCAATCCCGCAGAGACA-GTGGCAGATGAGAGA-
GGTTGTGACTTTAGGCAGCACAGGCTGA-
TGAGTTCCATCTGAGATGATTCCCAAGTGCATTAGTCCCATAATAATTACAACCAAATTATCACCTCCGCTTTGCTCACC
TACTCATAGAGTC--CCCTC--TGTAGAACCTGGGGAAAA-
AGACTTGACTAAGTCCTCCAGAGAAACCTTTAGCTTTGTCTCTA-AAGCAAGTCCTGCCATGTCTAGGTATCC-------
-----AATTTCAGTTA---CAGTATAAGCTACTATTGATTTGA-
TTTGATTGAGAATTTTTGGGACTCAGACTTGCCTGCCTGTA???????????????????????????????????????
??
????
Acanthisitta_chloris CCTATTGTTTTAGGTAACTATGCATCC---
AGATTCAATATACTTACATAATAGTACCAGCCTGTGAGGTGTAATACAGTTA-
CCTACCATGTTTCTTTACAGTTGGCATTTATGCAGGACGCAGACCCTCATTCGTAGACCCATGGGGTCTGTGTGCAGTAG
GTGTCTGCCCAGTGGTCTCCAATCCCACAGAGATA-GGGGCAGGTGAGGGA-
GGTTGTGACCTTGGACAACAGAAGCTGAAT----TCCATCTGGGATGATTCCCAGATGCATTAGCCCCATAAT--T-
ACAGCCAAATTATCGCCTCCACTGTGCTTACCTACTCAGAGAGTCAGTCCCCTTCACAGGACCTGGAGAAAA-
ACACTTGACT-AGTCTTCCAGGGAAACAATTAGCTTTGTCTGTATAAGCAAGTCCTGCCATGTCTAGGCATCT-------
-----GATTTCAGTTA---CAGTATAAGATACTATTGATTTGA-
TTTGGTTGTGAATTTTTGAAACTCAGACTTGCCTGCCTGTAAAGCATCATT--CAGGAGGCAAG--
CAACTGGATTGTTTTTAT----------------------
TCCCTATGTTGTCTTCATTATGACTGATATGTTTGTCTGTCTTTTATTTCCCAGGAAT
Regulus CCTATTGTTTTAGGTAACTATGCCTGC---
ATATCCAGTATTCTTACATGGTCCTACCAGTCTGGGAGGTGTCATGCAGTTG-
TTTATCATGTTTCTTTACAGTTGGCATTTGTACAGAATGCAGACCCTCATTCTCAGACCCA--
GGGTCTATGTGCAGCAGATCTCTGCCCGGCGGTCTCCAGTCCCACGGAGACA-GGGGCAGAT-------
GGCTGTGACCTTAGACAACAGAAGCTCAGT----TCCATCTGGGATGATTCCCAGATACATTAGCCCCATAGT--T-
AGAGACAAATTATCACCTTCACTTTGTTCTCCTACTCAGAG--TC--TCCTC--TGTAGGACCTGGAGAAAA-
ACACTTGAGT-
ACTCCTCCAGGGAAGCCATTAGCTTTGTCTGTATGAGCACATCRTGCCATGTCTAAGCATCCTGTAATAACTCTAATTTC
ACTTA---CAGTATAAGCTACTATTGATCTGA-
TTTAGTTGAGAATTTTTGTGTCTCAGACCTGCCTGCCTGTAAAGGATCATT--CAGGGAGCTAG--
CAACTGGATTATTTTTAT----------------------TCCAGGTGTTGTCTTAGTTATGATCAATGTGTTTG----
TATTTTATTTATCAGGAAT
Ailuroedus
??
??
??
??
??
??
??

??
?????????????
Zosterops CCTATTGTTTTAGGTAACTATGCCTGC---
ACACCCAATATTCTTACACAGTCCTACCAGCCTGGGAGGTGTCATGCAGTTG-
TTTACCATGTTTCTTTACAGTTGACATTTATACAGAATGCACACCCTCATTGTCAGACCCA--
GGGTCTGTGTGCAGCAAGTCTCTGCCTGGCAGTCTCCAGTCCTACAGAGACA-GGGGTAGAT-------
TGTTGTGACCATAGACAGCAGAAGTTCAGT----TCCATCTGAGGTGATTCCCACATGCATTAGCCCCATAGT--
TAAGAGCCAAATTATC------ACTTTGTTCCCCTACTCAGAG--TC--TCCTC--TGTAGGACCTGGAGAAAT-
ACACTTGAGT-
AGTCCTCCAGGGAAGCCATTAGCTTTGTCTGTATGGGCAAGTCCTGCCATGTCTAAGCATCCTGTAATAGTTGTCATTTC
AGTTA---CAGCATAAGTTACTATTGATTTGC-T---
GTTGAGAATTTTTGTGACTCAGACTTGCCTGCCTGTAGAGGATCATT--TAGGGAACAAG--
CAACAGGATTGTTTTTAT----------------------TCCCGGTGTTGTCTTTGTTATGACCAGTGTGTTTG----
TATTTTATTTCCCAGGAAT
Onychorhynchus
??
??
??
??
??
??
??
??
?????????????
Nestor_notabilis CCTATTGTTTTAGGTAACTATGCATCT---ATATTCCATATACTT----
AGTAGTACCAGCCTGTGAGGTGTAATGCAGTTG-
CTTATGATGTTTCTTTATGGTTGGCATTTATACAGAGGTCAGACCCTCATTTGTGGACCCACGGGGTCTGCGTGCAGCAG
GTATCTGCCTGTCAGTCTCCAGTCTCACAGAGACA-GTGGCAGAT-
GGGGAAGGTTGTGGCCTTAGAAAGCTGAAGCTGAGT----TCTGTCTGGGATGATTCCCAGACTCATTAGCCCCATAAT-
-G-ACAGCCAAATTATCGCCTCTATTCTGCTCACCTACTCAGAAAGGC--CACTT--GGTAGGACCTGGAGTAAA-
ATACT------AATCCTCCAGGGAAACCATTAGCT--GTCTGTGTAAACAAGTCCTGCCATATATAAGGATTT-------
-----GATTCCAGTTA---CAGTATAAGCTACTGTTTATGTGA-
TTTGGTTGTGAGTTCTTGGGACTCAGACTTGCCTGCCTGTAAAGCATCATT--CAGGGAGCAAG--
AAACTGGATTATCTTTAT----------------------
TCCCTGTGTTGTCTTTCTTATGACCAGTGTGTTTGTCTGCATTTTATTTCCCAGGAAC
Leiothrix_argentauris
??
??
??
??
??
??
??
??
?????????????
Troglodytes
??
??
??
??
??
??
??
??
?????????????
Luscinia_svecica
??
??
??
??

??
??
??
??
?????????????
Psittacus_erithacus   CCTATTGTTTTAGGTAACTATGCATCT---
ATATTCCGTATACTTACTCAGTAGTACCAGTCTGTGAGGTGTAATGCTGTTG-
CTTATGATGTTTCTTTACGGTTGGCATGTATACAGAGCTTGCACCCTCATTTATGGACCCATGGGGTCTATGTGCAGCAG
GCATCTGCCCAGCAGTCTCCAGTCTCACAGAGACA-GTGCCAGAT--
GGGAGGCTTGTGGCCTTAGAAGGCAGAAGTTGAGT----TCTATCTGGGATGATTCCCAGACTCATCAGCCCCATAAT--
G-ACAGCCAAATTATCRCCTCTGTTCTGCTCACCTGTTCAGAGAGGC--CACTC--AGTAGGACCTGGAGAAAA-
ACACT------GGTCCTCCAGGGAAACCATTAGCTATGTCTGTATAAACAAGCCCTGCGACATCTAAGCATTT-------
-----GATTTCAGTTA---CAGTATAAGCTACTGTTTATGTGA-
TTTGTTTGTGAGTTCTTGGGACTCAAACCTACCTGCCTGCAAARCATCATT--CAGGGAGCAAG--
CAACTGGATTATCTTTAT----------------------
TCCCTGTGTTGCCTTTGTTATGACCAATGTGTTTGTCTGCATTTTATTTCCCAGGAAC
Ficedula_hypoleuca
CCTATTGTTTTAGGTAACTATGCCTGCCATACATCCAGTGTTCTTGCACAGTCCTGCCAGCCTGGGAGGTGTCATGCAGT
TG-TTTATCATGTTTCTTTGCAGTTGGCATTTATGCAGAGTGCAGACCCACATTCTCAGACCCA--
GGGTCTGTGTGCAGCAGGTCTCTGCCCTGCGGTCTCCAGTCCCACAGAGAT---GGGCAGAC-------
TGTGGTGACCTTAGACAGCAGAAGCTCAGT----TCCATCTGGGATGATTCCCAGATGTATCAGCCCCATAGT--T-
AGAGCCAAATTATCACCTTCACTTTATTCACCTACCCAGAG--TC--TCCCT--
TGTAGGACCTGGAGAAACTACACTTGGGT-AGTGCTCCAGGGAGGCCATGAGCTTTG-
CTGTGTGAGCAAGTCCTGCCATGTCTGAGCATCCTGGAATAACTGTAATTTCAGTTA---
CAGCATAAGCTGCTATTGATTTGA-CTTAGTTGAGAATTTTTGGGACTCAGACTTGCCTGCCTGTAAAGGATCATT--
CAGGAAGCAAGCACAACTGGATTATTTTTAT----------------------
TCCTGGTGTTGTCTTTGTTATGACCAATGTGTTTG----TATTTTATTTCCCAGGAA-
Menura_novaehollandiae   CCTATTGTTTTAGGTAACTATGCCTCC---
ACATTCAATATACTTACACAGTCTTACCAGCCTGGGAGGTGTCATGCAGTTGCTTTATCATGTTTCTTTACAGTTGGCAT
TTATACAGAACACAGACCCTCATTCTCAGACGTAGGGG-----------------------------
TCTCCAATCCCACAGAGACAGGGGGCAGATGAGAGA-GGTTGTGACCTTAGACAACAGAAGCTCAGT----
TCCATCTGGGATGGTTCCCAGGTGCATTAGGCCCATCAT--T-
ACAGCCAAATTATCACCTCCACTTTGCTCACCTACTCAGAGAGTC--CCCTT--TGTAGAACCTGGAGAAAA-
ACACTTGACA-GGTCCTCCAGGGAAACCACTAGCTTTGTCTGTGTAAGCAAGTCCTGCCATGTCTAGGCATCC-------
-----AGTTTCAATTA---CAGCATAAGCTGCTGTTGATTTGA-
TTCAGTTGAGAATTTCTGTGACTCAGCCTTGCCTGCCTGTAAAGCATCATT--CAGGGAGCAAG--
CAACTGGATTATTTTTAT----------------------
TCCTTCTGTTGTCTTTGTTATGACCAATGTGTTTGTCTGTATTTTATTTCCCAGGAAT
Pycnonotus   CCTATTGTTTTAGGTAACTATGCCTGC---
ACATCCAGTATTCTTACACAGTCCTACCAGCCTGGGAGGTGTCATGCACTTG-
TTTATCATGTTTCTTTACAGCTGGCATTTATACAGAATGCACACCCTCATTCTCAGACCCA--
GGGTCTGTGTGCAGCAGGTCTCTGCCCGGCAGTCTCCAGTCTGACAGAGACA-GGAGCAGAC-------
TGTTCTGACCTTAGA---------------------CCTCTGGGATGATTCCCAGATGCAGTAGCTCCATAGT--T-
AGAGCCAAATTGTCACCTTCACTTTGTTTGCCTACTCAGAG--TC--TCCTC--TGTAGGACCTGGTGAAAA-
ACAGTTGAGT-
AGTCCTCCAGGAAAGTCATTACCTTTGTCTGTATGAGCAAGTCCTGCCATGTCTAAGCATCCTGTAACAGCTGTCATTTC
AGTTA---CAGCATAAGCTACTATTGATTTGA-
TTTAGTTGAGAATTTTTGTGACTCAGACTTGCCTGCCTGTAAAGGATCATT--CAGGGAGCAAG--
CAACAAGATTATTTTTAT----------------------TCCCAGTGTTGTCTTTGTTATGACCGATGTGTTTG----
TATTTTATTTCCCAGGAAT
Donacobius_atricapilla
??
??
??
??
??
??
??
??
?????????????

Hirundo_rustica
??
??
??
??
??
??
??
??
?????????????
Lichenostomus
??
??
??
??
??
??
??
??
?????????????
Cnemophilus
??
??
??
??
??
??
??
??
?????????????
Coracina
??
??
??
??
??
??
??
??
?????????????
Dicrurus   CCTATTGTTTTAGGTAACTATGCCTCC---
ACATCCAATATTCTTACACAGTCCTCCCCGCCTGGAAGGTGTCATGCAGTTG-
TTTATCATGTTTCTTTACAGTTGGCATTTACACAGAACGCAGACCCTCATTCTCAGACCCA-
GGGGCCTGTGTGCCACATGTCTCTGCCTGGCAGTGTCCAATCCCACAGAGACA-GGGGCAGAT-------
TGTTGTGACCTTAGACAGCAGT------------TCCAGCTGGGATGATTCCCAGATGCATTAGCCCCA---T--T-
AGAGCCAAATTATCACCTTCACTTTGCTCACCTACTCAGAG--TC--TCCTC--TGTAGGACCTGGAGAAAA-
ACACTTGACT-AATCCTCCAGGGAAGCCGCTAGCTTTGTCTGTATGAGCAAGTCCTGCCATGTCTAAGCATCC-------
-----AATTTCAGTTA---CAGCACAAGCTACAATTTATTTGA-
TTTAGTTGAGAACTTTTGTGACTCAGACTTGCCTGCCTGTAAAGGATCATT--CAGAGAGCAAG--
CAACTGGATTATCTTTAT----------------------TCCCTGTGTTGTCTTTGTTATGACCAATGTGTTTG----
CATTTTATTTCCCAG????
Gerygone_fusca
??
??
??
??
??
??
??

```
????????????????????????????????????????????????????????????????????????????????
?????????????
Ptiloris_magnificus
????????????????????????????????????????????????????????????????????????????????
????????????????????????????????????????????????????????????????????????????????
????????????????????????????????????????????????????????????????????????????????
????????????????????????????????????????????????????????????????????????????????
????????????????????????????????????????????????????????????????????????????????
????????????????????????????????????????????????????????????????????????????????
????????????????????????????????????????????????????????????????????????????????
????????????????????????????????????????????????????????????????????????????????
?????????????
Paradisaea_raggiana
????????????????????????????????????????????????????????????????????????????????
????????????????????????????????????????????????????????????????????????????????
????????????????????????????????????????????????????????????????????????????????
????????????????????????????????????????????????????????????????????????????????
????????????????????????????????????????????????????????????????????????????????
????????????????????????????????????????????????????????????????????????????????
????????????????????????????????????????????????????????????????????????????????
????????????????????????????????????????????????????????????????????????????????
?????????????
Cyanocorax_chrysops        CCTATTGTTTTAGGTAACTATGCCTCC---
ACATCCAATACTCTTACACAGTCCTGCCAGCCTGAGAGGTGTCATGCAGTTG-
TTTATGATGTTTCTTTACAGTTGGCATTTATACAGAACGCAGACCCTCATTCTCAGACCCAGGGGGTCTTTGTGCAGCAG
GTCTCTGCCTGGCGGTCTCCAATCCCACAGAGGCA-GGGACAGAT-------
TGCTGTGACCTTCGACAGCAGAAGCTCRGT----TCTATCTGGGATGATTCCCAGATGCATTAGCCCCATAGT--T-
AGAGCCAAATTAACACCTTCACTTTGCTCACCTACTCAGAG--TC--TCCTC--TGTAGGACCTGGAGAAAA-
ACACTTGACT-AATCCTCCAGGGAAGCCGTTAGCTT----TGTATGAGCAAGTGCTGCCATGTCTAAGCATCC-------
-----AGTTTCAGTTA---CAGCACAAGCTACAATTTATTGGA-
TTTAGCTGAGAACTTTTGTGACTCAGACTTGCCTGCCTGTAAAGGATCATT--CAGGGAGCAAG--
CAACTGGATTATCTTTAT----------------------TCCCTGTGTTGTCTTTGTTATGACCGATGTGTTTG----
CATTTTATTTGCCAGGAAT
Lonchura
????????????????????????????????????????????????????????????????????????????????
????????????????????????????????????????????????????????????????????????????????
????????????????????????????????????????????????????????????????????????????????
????????????????????????????????????????????????????????????????????????????????
????????????????????????????????????????????????????????????????????????????????
????????????????????????????????????????????????????????????????????????????????
????????????????????????????????????????????????????????????????????????????????
????????????????????????????????????????????????????????????????????????????????
?????????????
Phylloscopus
????????????????????????????????????????????????????????????????????????????????
????????????????????????????????????????????????????????????????????????????????
????????????????????????????????????????????????????????????????????????????????
????????????????????????????????????????????????????????????????????????????????
????????????????????????????????????????????????????????????????????????????????
????????????????????????????????????????????????????????????????????????????????
????????????????????????????????????????????????????????????????????????????????
????????????????????????????????????????????????????????????????????????????????
?????????????
Turdus                     CCTATTGTTTTAGGTAACTATGCCTGC---
ACATCCAGTGTTCTTACACAGTCCTGCCAGCCTGGGAGGTGTCATGCA-TTG-
TTTATCATGTTTCTTTGCAGTTGGCATTTATCCTGAATGCAGACCCTCAT-CTCAGACCC--
GGGGTCTGTGTGCAGCAGGTCTCTGCCCGGCGCTCTCCAGTCCCACAGAGACA-GGGACAGAT-------
TGTYGTGACCTTAGACAACAGAAGCTCAGT----TCCATTTGGGATGATTCCCAGATGCATTAGCCCCATAGT--T-
AGAGCCAAATTATCACCTTCACTTTGTTCACCTACCCAGAG--TC--TCCCT--TGTAGGACCTGGAGAAAA-
ACACTTGAGT-
```

AGTCCTCCAGGGAAGCCATTAGCTTTGTGTGTATGAGCAAGTCCTGCCATGTCTGAGCATCCTGTAATTGCTGTAATTTC
AGTTA---CAGCATAAGCTACTATTGATTTGA-
TTTAGTTGAGAATTTTTGTGACTCAGACTTGCCTGCCTGTAAAGGATCATT--GAGGGAGCAAG--
CAAYTGAATTGTTTTTAT----------------------TCCTGGTTTTGTCTTTGTTATGACCGATGTGTTTG----
TATTTTATTTCTCAGGAAT
Acrocephalus
??
??
??
??
??
??
??
??
?????????????
Sitta
??
??
??
??
??
??
??
??
?????????????
Mimus   CCTATTGTTTTAGGTAACTATGCCTGC---
ACATCCAATATTCTTACACACTCCTACCAGCCTGGGAGGTGTCATGCAGTTG-
TTTATCACGTTTCTTTACAGTTGGCATTTAAACCCAGGGCAGACCCTCATTATCAGATCCA--
GGGTCTGTGTGCAGCAGGTCTCTGCCCGGCGCTCTCCAGTCCCACAGAGACA-GGGGCAGAT-------
TGTTGTGRCCTTAGACAGCATAAGCTCAGT----TCCATCTGGGATGATTCCCAGATGCATTAGCCCCATAGT--T-
AGAGCCAAATTATCACCTTCACTTTGTTCACCTACCCAGAG--TC--TCCCT--TGAAGGACCTGGAGAAAA-
ACACTGGAGT-
AGTCCCACAGGGAAGCCATTAGCTGTGTTTGTGTGAGCAAGTCCTGCCGTGTCAAAGCATCCTGTAATAACTGTAATTTC
AGTTA---CTGAATAAACTCCTATTGATTTGA-
TTTAGTTRAGAATTTTTGTGACTCAGACTTGCCTGCCTGTAAAGGATCATT--CAGGGAGCAAG--
CAACTGGATTATTTTTAT----------------------
TCCCAGTGTTGTCTTTGTTATGACCG????????????????????????????????
Sturnus_vulgaris   CCTATTGTTTTAGGTAACTATGCCTGC---
ACATCCAGTATTCTTACACACTCCTCCCAGCCYGGGAGGTGTCATGCACTTG-
TTTATCATGTTTCTTTACAGTTGGCATTTGTACCCAATGCAGACCCTCTTTCTCAGACCCA--
GGRTCTGYGTGCAGCAGGTCTCTGCCCGGCGCTCTCCCGTCCCACAGAGACA-GGGGCAGAT-------
TGCTGTGGCCTTAGACAGCAGAAGCTCAGT----TCCATCTGGGRTGATTCCCAGATGCATTAGCCCCATAGT--T-
AGAGCCAAATTATCATCTYCACTTTGTTCACCTACCCAGAG--TC--TCCCT--TGTAGGATCTGGAGAAAA-
ACACTTGAGT-
AGTCCTACAGGGAAGCCATTAGCTTTGTCTGTGTGAGCAAGTCCTGCCATGTCTAAGCATCCTGTAATAACTGTAATTTC
AGTTA---CTGAATAAGCTACTATTGATTTGA-
TTTAGTTGAGAATTTTTGTGACTCAGACTTGCCGGCCTGTAAAGGATCATT--CAGGAAGCAAG--
CAACTGGATTATTTTTAT----------------------
TCCCAGTGTTGTCTTTGTTCTGACCA????????????????????????????????
Creadion_carunculatus
??
??
??
??
??
??
??
??
?????????????

```
Parus
????????????????????????????????????????????????????????????????????????????????
????????????????????????????????????????????????????????????????????????????????
????????????????????????????????????????????????????????????????????????????????
????????????????????????????????????????????????????????????????????????????????
????????????????????????????????????????????????????????????????????????????????
????????????????????????????????????????????????????????????????????????????????
????????????????????????????????????????????????????????????????????????????????
????????????????????????????????????????????????????????????????????????????????
?????????????
Petroica_rosea
????????????????????????????????????????????????????????????????????????????????
????????????????????????????????????????????????????????????????????????????????
????????????????????????????????????????????????????????????????????????????????
????????????????????????????????????????????????????????????????????????????????
????????????????????????????????????????????????????????????????????????????????
????????????????????????????????????????????????????????????????????????????????
????????????????????????????????????????????????????????????????????????????????
????????????????????????????????????????????????????????????????????????????????
?????????????
Eopsaltria_australis
????????????????????????????????????????????????????????????????????????????????
????????????????????????????????????????????????????????????????????????????????
????????????????????????????????????????????????????????????????????????????????
????????????????????????????????????????????????????????????????????????????????
????????????????????????????????????????????????????????????????????????????????
????????????????????????????????????????????????????????????????????????????????
????????????????????????????????????????????????????????????????????????????????
????????????????????????????????????????????????????????????????????????????????
?????????????
Serinus                   CCTATTGTTTTAGGTAACTATGCCTGC---
ACATCCAATATTCTTACACAGTGGTGGGAGACTA-GAGGTGTCATGCAGTTG-
TTTATCATGTTTCTTTGCAGTTAGCCTTTATACAGAATGCAGACCCTCATTCTCAGACCCG--
GGGTCTGTGTGCAGCAGGTCTCTGCCCAGCGGTCACCAGTCCCACAGAGACA-GGGGCAGAT-------
TGTTGTGACCTTAGACAGTAGAAGCTCAGT----TCCATGTGGATGATTCCCAGATGCATTAGCCCCATAGT--T-
AGAGCAAAATTATCACCTTCACTTTGTTCACCTATTCAGAG--TC--TCCTC--TGTAGGACCTGGAGAAAA-
ACACTTGAGT-
AGTCCTCCAGGGAAGCCTTAAGCTTTGTTGGTATGAGCAAGTCCTGCCATGTCCAAGCATCCTGCAATAACTGTAATTTC
AGTTA---CAGCATAAGCTACTATTGATCTGA-
TTTAGTTGAGAATGTTTGTGACTCAGACCTGCCTGTCTGTAAAGGATCATT--CAGGGAG-AAG--
CAACTGGATTATTTTTAT----------------------TCCCAGTGTTGTCTGTGTT--GACCAATGTGTTTG----
TATTTTATTTCCCAGGAAT
Icterus
????????????????????????????????????????????????????????????????????????????????
????????????????????????????????????????????????????????????????????????????????
????????????????????????????????????????????????????????????????????????????????
????????????????????????????????????????????????????????????????????????????????
????????????????????????????????????????????????????????????????????????????????
????????????????????????????????????????????????????????????????????????????????
????????????????????????????????????????????????????????????????????????????????
????????????????????????????????????????????????????????????????????????????????
?????????????
Motacilla                 ?????????????GTAACTGTGCCTGC---
ACATCCAATATTCTTACACAGTGGTGGGAGACT-GGAGGTATCATGCAGTTG-
TTTATCATGTTTCTTTACAGTTGGCATTTATAGAGAATGCAGATCCTCATTCTCAGACCCA--
GGGTCTGTGTGCCGCAGGTCTCTGCCCAGTGGTCTCCAATCCCACAGAGAGA-GGGGCAGAT-------
TGTTGTGACCTTAGACAGTAGAAGCTCAGT----TCCACCTGGGATGATTCCCAGATGCATTAGCCCCATAGT--T-
AGAGCAAAATTATCATCTTCACTTTGTTCACCTACTCAGAG--TC--TCCTC--TGTAGGACCTGGAGAAAA-
ACACTTGAGT-
AGTCCTCCAGGGAAGCCATTAGCTTTGTCTGTATGAGCAAATCCTGCCATGTCCAAGCATCCTGCAATAACTGTAATTTC
```

AGTTA---CAGCATAAGCTACTATTGATCTGA-
TTTAGTTGAGAATTTTTGTGACTCAGACCTGCCTGTCTGTAAAGGATCATT--CAGGGAGCCAG--
CAACTGGATTATTTTTAT----------------------TCCCAGTATTGTCTTTGTTATGACCAATGTGTTTG----
CATTTTATTTCCCAG????
Emberiza
??
??
??
??
??
??
??
??
?????????????
Pomatostomus
??
??
??
??
??
??
??
??
?????????????
Rhipidura   CCTATTGTTTTAGGTAACTATGCCTCC---
ACACCCAATATTCTTACACAGTCCTGCCAGCCTGGGAGGTGTCATGCAGTTG-
TTCATCATGTTTCTTTACAGCTGGTCTTTATACAGAACGCAGACCCTCATTCTCAGCCCCAGGGGGTGTGTGTGCAGCRG
GTCTCTGCCTGGCAGTCTCCAATCCCACAGAGACA-GGGGCAGAG-------
AGTTGTGACCTTAGAAAGCAGAAGCTGAGT----TCTGTTTGGGATGATTCCCAGATGCATTAGCCCCATAGT--T-
AGAGCCAAATTTTCACCTTCACTTTGCTCATCTACTCAGAG--TC--TCCTC--TGTAGGGCCTGGAGAAAA-
ACACTTGGCC-AATCCTCCAGGGAAGCCTTTAGCTTTGTCTGTATGAGCAAGTCCTGCCATGTCTAAGCATCC-------
-----AGTTTCAGTTA---CAGCACAAGCTACAATTGATTTGA-
TTTAGTTGAGAACTTTTTTGACTCAGACTTGCATGCCTGTAAAGGATCATT--CAGGGAGCAAG--
CAACTGGATGATCCTTAT----------------------TCCCAGTGTTGTCTTTGTTATGACCGATGTGTTTG--
TGGGGTTTTTTTCCCAG????
Pica_pica
??
??
??
??
??
??
??
??
?????????????
Manucodia
??
??
??
??
??
??
??
??
?????????????
Corvus_corone   CCTATTGTTTTAGGTAACTATGCCTCC---
ACATCCAATACTCTTACACAGTCCTGCCAGCCT--GAGGTGTCATGCAGTTG-
TTTATCATGTTTCTTTACAGTTGGCATTTATACAGAACACAGACCCTCATTCTCAGACCCAGGGGGTCTGTGTGCAGCAG
GTCTCTGCCTGACGGTCTCCAATCCCACAGAGACA-GGGACAGAT-------
TGCTGTGACCTTCGACAGCAGAAGCTCAGT----TCCATCTGGGATGATTCCCAGATGCATTAGCCCCATAGT--T-

AGAGCCAAATTAACACCTTCACTTTGCTCACCTACTCAGAG--TC--TCCTC--TGTAGGACCTGGAGAAAA-
ACACTTGACT-AATCCTCCAGGGAAGCCGTTAGCTT----TGTATGAGCAAGTCCTGCCATGTCTAAGCATCC-------
-----AGTTTCAGTTA---CAGCACAAGCTACAATTTATTGGA-
TTTAGTTGAGAACTTTTGTGACTCAGACTTGCCTGCCTGTAAAGGATCATT--CAGGGAGCAAG--
CAACTGGATTATCTTTAT----------------------TCCCTATGTTGTCTTTGTTATGACCGATGTGTTTG----
TATTTTGTTTCCCAGGAAT
Vireo
??
??
??
??
??
??
??
??
?????????????
Camptostoma_obsoletum
??
??
??
??
??
??
??
??
?????????????
Promerops_cafer
??
??
??
??
??
??
??
??
?????????????
Oriolus
??
??
??
??
??
??
??
??
?????????????
Dendroica ??????????????????TATGCCTGC---
ACATCCAATATTCTTACACAGTGGTGGGAGACT-GGAGGTGTCATGCAGTTG-
TTTATCATGTTTCTTTACAGTTAGCCTTTATACAGAATGCAGACCCTCA-------------
GGGGTCTGTGTGCAGCAGGTCTCTGCCCAGCGGTCTCCAATCCCACAGAGACA-------GAT-------
TGTTGTGACCTTAGACAGTAGAAGCTCAGT----TCCATGTGGGATGATTCCCCGGTGCATTAGCCCCATAGT--T-
AGAGCAAAATTATCACCTTCACTTTGTTCCC-TACTCA-AGAGTC--TCCTC--TGTAGAACCTGGAGAAAA-
ACACTTGAGT-AGTCCTCCAGAGAAGTCATTAGCT----CT--
ATGAGCAAGTTCTGCCATGTCCAAGCATCCTGCAATAACTGTAATTTCAGWTA---
CAGCATAAGCTACTATTGATCTGA-
TTTAGTTGAGAATTTTTGTGACTCAGACCTACCTTTCTGTAAAGGATGATTTCCAGGGAGCAAG--
CAACTGGATTATTTTTAT----------------------TCCCAGCGTTGTCTTTGTTATGACCGATGTGTTTG----
TATTTTATTTCCC??????
Nectarinia ????????????????????????????????ATCCAATATTTT-
ACACAGTCCTACCAGCCTGGGAGGTTTCATGCAGTTG-

TTTATCACGTTTCTTTACAGTTGGCATTGATACAGAATGCAGACCTTCATTTTCAGACCCA--
GGGTCTGTGTGCAGCGGGTCTC----CAGT-----CCCA---CAGAGACAGG-AGCAGATTG---------
TTGTGACCTTAGACA---GAAGCTCAGT----TCTGTCTGGGATTATTCCCAGATGCATTAGCCCCATAGT--T-
AGAGACAAATTATCACCTACACTTCGTTCAGCTACTCAGAG--TC--TCCTC--TGTAGAACCTGGAGAAAA-
ACGCTTGAGT-
AGTGCTCCAGGGAAGCCATTAGCTTTGTCTGTATGAGCAAGTCCTGCCATGTCCAAGCATCCTATAATAACTGTAATTTC
AGTTA---CAGCATAAGCTACTATTGATTTGA-
CTTAGTTGAGAATTTTTGTGACTCAGACTTGCCTGCCTGTAAAGGGTCATT--CAGGGAGCAAG--
CAACTGGATTATTTTTAT----------------------
TCCCAGTGTTGTCTTTGTTATG????????????????????????????????????
Amytornis_striatus CCTATTGTTTTAGGTAATTATGCCTCC---ACATTCAATATTCTTTCAC-
GTCCTACCATCCTGGGAGGTGTCATGCAGTTG-
TTTGTCATGTTTCTTTACAGTTGGCATTTACACAGAATGCAGACCCTCGCTCTCAGA----------------------
GGTTGCTGCGTGGCAGTCTCCAGTCCCACAGAGAG--GGGGCAGAT-------TGCTGTGACCTTAGA--
GCAGAAGCTCAGT----TCCATCTGGGATGGTTACCAGATGCATCAGCCCCATACT--T-
ACGGCCAAATTATCACCTTCACTTTGCTCACCTACTCAGAGAGTC--TCCTC--TGTAGGACCTGGAGAAAA-
GCACTTGACT-AGTCCTCCAGGGAAACTGGTCACTT----TGTATGAGCATGTCCTGCCATGTCTAAGCATCC-------
-----AATTTCAGTTG---CAGCATAAGCTACTACTGATTTGA-
TTTTGTTGAGAATTTTTGTGCCTCAGACTTGCCTGCCTGTAAAGGATCATT--CAGGGAGCAAG--
CGCCTGGATTATTTTTAT----------------------
TCCCTGTGTTGTC???
Pitta CCTATTGTTTTAGGTAACTATGCATCC---
ATATTCAATGTACTTACATAATCATACCAAACTGTGAGGTGTCATGCAGTTG-
CTTACCATGTTTCTTTACAGTTGRCATTCATACAGAATGCAGACCTTCATTTGCAGACCCATGGKACCT--
GTGCAGCAGG--------------TCTCCAGTACTGCAGAGACA-GTGGTAGATGAGGGA-
GGTTGTGACACCAGGCAACAGAAGCTGATT----
TCCATCTGAGATGGTTCCCAGATGCATTAGCCCCATAATAATTACAGCCAAATTGTCACCTGCACTTTGGTAACCTACTC
AGAGAGTC--CCCTC--CATAGGACCTGGAGAAAA-
ACACCTGACTAAGTCCACCAAGGAAACCATTAGCTTGGTCTGTATAATCAAGTCCTGCCATTTCTAGGCATCG-------
-----GATTTCAGTTA---CAGTATAAACTATTATTGATTTG------
GTTGAGAACTTTTGGGACTCAGACTTGTCTGCCAGTAAAACATCATT--CAGGGAGCAAG--
CAGGTGGATTATTTTTATGTTGCCTTTATTAAGACTGATATATGTATGCTGCCTTTGTTAAGACTGATGTGTTTGTCTGT
ACTTTATTTCCAAGGAAT
Toxorhamphus
??
??
??
??
??
??
??
??
?????????????
Orthonyx_temminckii
??
??
??
??
??
??
??
??
?????????????
Sericulus_chrysocephalus
???????????????????????????????CATTGAGTGTGCTTACACAGTCCTACCAGCCTGGCAGGTCTCATGCAGT
TG-
CTTATCATGTTTCTTTACAGTTGGCATTTATACAGAACACAGACCCTCATTCTCAGACCCAGGGGGTCTCTGTGCAGCAG
TTCTCTGCCC??
??
??

??
??
??
??????????
Cyclarhis_gujanensis
??
??
??
??
??
??
??
??
?????????????

;
End;
